# A general approach for activity-based protein profiling of oxidoreductases with redox-differentiated diarylhalonium warheads†

**DOI:** 10.1039/d4sc08454c

**Published:** 2025-03-11

**Authors:** Leo Krammer, Barbara Darnhofer, Marko Kljajic, Laura Liesinger, Matthias Schittmayer, Dmytro Neshchadin, Georg Gescheidt, Alexander Kollau, Bernd Mayer, Roland C. Fischer, Silvia Wallner, Peter Macheroux, Ruth Birner-Gruenberger, Rolf Breinbauer

**Affiliations:** a Institute of Organic Chemistry, Graz University of Technology Stremayrgasse 9 8010 Graz Austria breinbauer@tugraz.at; b Diagnostic and Research Institute of Pathology, Medical University of Graz Stiftingtalstraße 6 8036 Graz Austria; c Institute of Chemical Technologies and Analytics, Technische Universität Wien Getreidemarkt 9 1060 Vienna Austria ruth.birner-gruenberger@tuwien.ac.at; d Institute of Physical and Theoretical Chemistry, Graz University of Technology Stremayrgasse 9 8010 Graz Austria; e Institute of Pharmaceutical Sciences, University of Graz Humboldtstraße 46 8010 Graz Austria; f Institute of Inorganic Chemistry, Graz University of Technology Stremayrgasse 9 8010 Graz Austria; g Institute of Biochemistry, Graz University of Technology Petersgasse 12 8010 Graz Austria

## Abstract

Activity-based protein profiling (ABPP) is a unique proteomic tool for measuring the activity of enzymes in their cellular context, which has been well established for enzyme classes exhibiting a characteristic nucleophilic residue (*e.g.*, hydrolases). In contrast, the enzyme class of oxidoreductases has received less attention, as its members rely mainly on cofactors instead of nucleophilic amino acid residues for catalysis. ABPP probes have been designed for specific oxidoreductase subclasses, which rely on the oxidative conversion of the probes into strong electrophiles. Here we describe the development of ABPP probes for the simultaneous labeling of various subclasses of oxidoreductases. The probe warheads are based on hypervalent diarylhalonium salts, which show unique reactivity as their activation proceeds *via* a reductive mechanism resulting in aryl radicals leading to covalent labeling of liver proteins at several different amino acids in close proximity to the active sites. The redox potential of the probes can be tuned by isosteric replacement varying the halonium central atom. ABPP experiments with liver using 16 probes differing in warhead, linker, and structure revealed distinct overlapping profiles and broad substrate specificities of several probes. With their capability of multi oxidoreductase subclass labeling – including rare examples for the class of reductases – and their unique design, the herein reported probes offer new opportunities for the investigation of the “oxidoreductome” of microorganisms, plants, animal and human tissues.

## Abstract 2

A general chemoproteomic profiling approach for oxidoreductases with conceptually novel probes based on diarylhalonium salts is reported.

## Graphical abstract

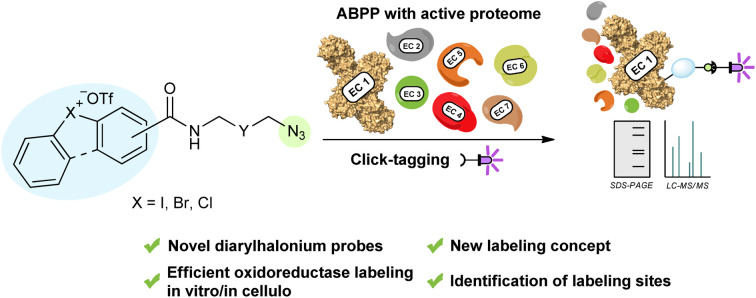

## Introduction

Since its introduction by Cravatt in 1999,^1^ activity-based protein profiling (ABPP) has emerged as an indispensable chemical proteomic technique,^2^ as it allows the exclusive analysis of the catalytically active proteome in a cell or living organism. While originally designed for hydrolases (EC 3),^1,3^ ABPP was soon expanded to other enzyme classes bearing a characteristic nucleophilic residue,^4^ thereby becoming a valuable tool for the investigation of the mode of action and selectivity profile of drugs^5^ and natural products.^6^ However, enzyme classes with more complex catalytic activities still have received much less attention. One of these are oxidoreductases (EC 1), which are involved in various important metabolic processes ranging from drug metabolism, over amino/fatty acid synthesis and degradation to synthesis of secondary metabolites. As these enzymes mostly rely on cofactors (*e.g.*, NAD(P)H, flavins, pyridoxal phosphate (PLP), heme, *etc.*) for their catalytic action, it is more difficult to annotate their function on a gene sequence level, as the encoded amino acids do not fulfil a distinct catalytic function. Therefore, ABPP provides the means to overcome these limitations by direct mechanism-based functional profiling.

**Fig. 1:**
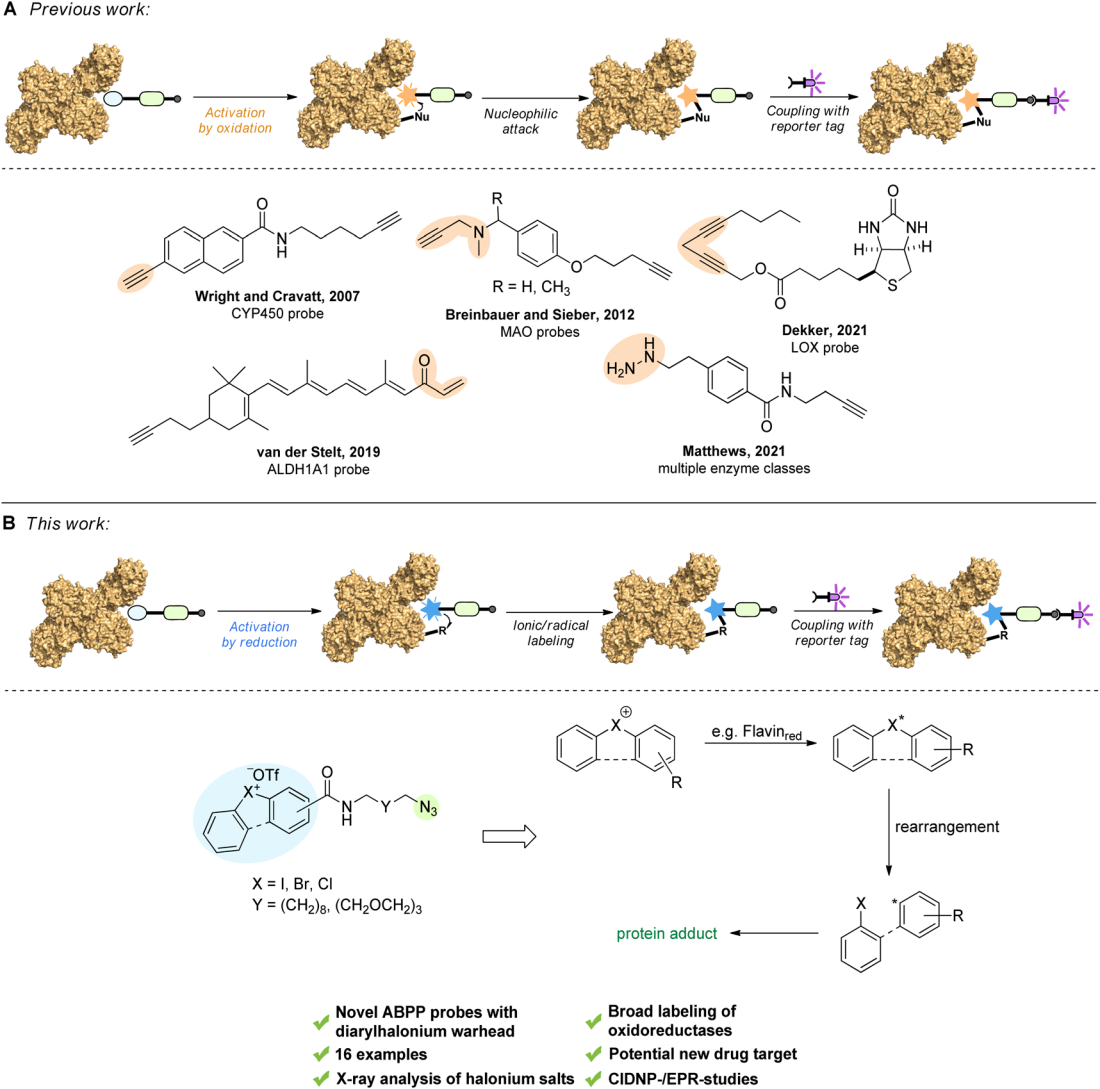
(A) Previous work based on the concept of “activation by oxidation” and selected examples thereof. (B) This work, based on the concept of “activation by reduction” and proposed, simplified mechanism for the mode of action *via* radical formation upon enzymatic reduction. Warheads are highlighted in orange and blue, the reporter tag click handle is highlighted in green.

The most decisive element for the specificity of an activity-based probe is the reactive group or warhead.^7^ Two principles are pursued: (a) probes, which bear a reactive electrophilic group prone to be attacked by a nucleophilic residue of the enzyme class under investigation, or (b) a latent functional group, which under activation by the protein class of interest is transformed into a highly reactive – typically electrophilic – species. A reporter tag or click-handle is attached to the warhead *via* a flexible linker. The labeled proteome can be analyzed by gel-electrophoresis or quantitative LC-MS/MS analysis for target protein identification. In general, probes are either designed as universal probes that react with most members of an enzyme family – an approach used for activity profiling of its members and comparative ABPP addressing the target-specificity of small-molecule inhibitors – or highly specific probes that target one specific protein of interest, which also allow the investigation of the mode of action and drug target profile of small molecule inhibitors. The design of probes for ABPP of oxidoreductases usually follows the same principles mentioned above. The warhead is in most cases activated by oxidation by the protein of interest and converted into an electrophile, which can be attacked by a nucleophile in close proximity forming a covalent linkage (Fig. 1A). This principle has led to probes specific for their target enzyme class.^8^ For example, the group of Cravatt has developed alkyne-probes (converted into a highly reactive ketene) for ABPP studies with CYP450s,^9^ which can also be targeted by heterocycles like benzofuran or thiophene.^10^ Alkyne warheads have also been used for Fe(ii)/α-ketoglutarate dependent dioxygenases.^11^ Monoamine oxidases have been selectively addressed by propargylamine warheads that are converted into propargyl iminium species (Fig. 1A), or cyclopropyl amines.^12^ The group of Dekker used a bispropargylic warhead (Fig. 1A) in their studies with lipoxygenases, whereupon a highly reactive allene radical is formed after single-electron oxidation by the enzyme.^13^ In a different line of research, the group of van der Stelt has developed an ALDH1A1 probe containing an electrophilic vinyl ketone, which is attacked by a nucleophilic Cys in the active site (Fig. 1A).^14^ However, there is also the possibility of oxidoreductases already bearing an electrophilic unit. These can be attacked by nucleophilic warheads like hydrazine, as shown in the ABPP labeling of lentil seedling diamine oxidase (LSDAO) by the group of Jakobsche.^15^ Most recently, Matthews and co-workers showed that their supernucleophilic hydrazine warhead designed for profiling of electrophilic protein sites (Fig. 1A) was oxidized by certain enzymes to a diazo intermediate and then converted into an alkyl radical upon N_2_-extrusion. With these two modes of probe reactivity they were able to modify different classes of enzymes, including several oxidoreductases (*e.g.*, MAO A/B, ALDH2, COX17, *etc.*).^16,17^ Also of note, Sieber has developed a clickable PLP-analogue, which enabled the labeling of PLP-dependent enzymes.^18^ While these examples demonstrate the progress in the field of oxidoreductase subclass specific probes, we herein report a new class of ABPP probes for oxidoreductases that engage in broad oxidoreductase subclass labeling.

Diaryliodonium salts and their lighter halogen homologues display higher valence state halogens. Postulated in 1937 by Roberts and Kimball as putative intermediates in the halogenation of ethylenes,^19^ various halonium salts have been successfully synthesized, with diaryliodonium species representing the majority,^20–23^ which have also shown their potential as inhibitors of certain oxidoreductases.^24–28^ The subclass of diarylhalonium species includes both diphenyl- and diphenylene-based derivatives (acyclic *vs.* cyclic). While acyclic diarylhalonium salts have been known for a longer time and also have found application as useful catalysts (*e.g.*, photoinitiators)^29,30^ and arylating agents,^31^ cyclic diarylhalonium ions gained interest only very recently.^32–34^ Based on their unique redox properties, we propose diarylhalonium probes^35^ which undergo activation by reduction forming aryl ions/radicals which can covalently react with several amino acid residues or bound cofactors (Fig. 1B), thereby overcoming the necessity of suitably positioned nucleophilic residues for the established probes, which require activation by oxidation (Fig. 1A).

## Results and discussion

### Development of activity-based probes

Although intrinsically reactive, diarylhalonium salts are in general bench-stable compounds for which several synthetic methods and purification protocols have been established over time.^23,36^ Diaryliodonium salts are used as photoinitiators for polymerization as under irradiation with UV light the photo-excited molecule is activated, thereby creating radicals and radical cations.^29,30^ Diphenyliodonium chloride (DIP)^27^ and diphenyleneiodonium chloride (DPI) have shown broad *in vitro* and *in vivo* activity for several NAD(P)-dependent oxidases.^24–26^ The mode of action is believed to involve single electron reduction by the flavin cofactor of the oxidoreductase and subsequent dissociation/radical formation of diarylhalonium species, which react with amino acid residues.^26,27,37^ Massey has noted in his studies of DIP inhibition of several enzymes, that the redox potential of the flavoenzyme, in particular that of the Fl_semiquinone_/Fl_red_ couple has influence on the inhibition by DIP.^37^ Ideally it should be lower than that of the Ph_2_I^+^/Ph_2_I˙ couple. Within flavoproteins enzyme active sites exhibit flavin redox potential ranging from +100 mV to −400 mV enabling the catalysis of a variety of redox reactions.^38^ It has also been proposed, that even the stereochemical outcome of reactions with flavoenzymes is correlated with their redox potential.^39^

We envisioned that a warhead based on the diaryliodonium moiety should also be applicable to other oxidoreductases and different cofactors as long as the activation *via* reduction will take place. This should apply to both oxidases as well as reductases, since, in their respective catalytic cycles, reduced states or reduced cofactors are involved or produced. In order to address the different role of the potential of the various oxidoreductases we planned to synthesize diarylbromonium and diarylchloronium warheads as well. As ionization potential and electronegativity increases going from iodine to chlorine, a difference in reactivity is to be expected. This would allow a fine-tuning of redox reactivity of the probes in analogy to the reactivity tuning of electrophilic warheads.

**Scheme 1:**
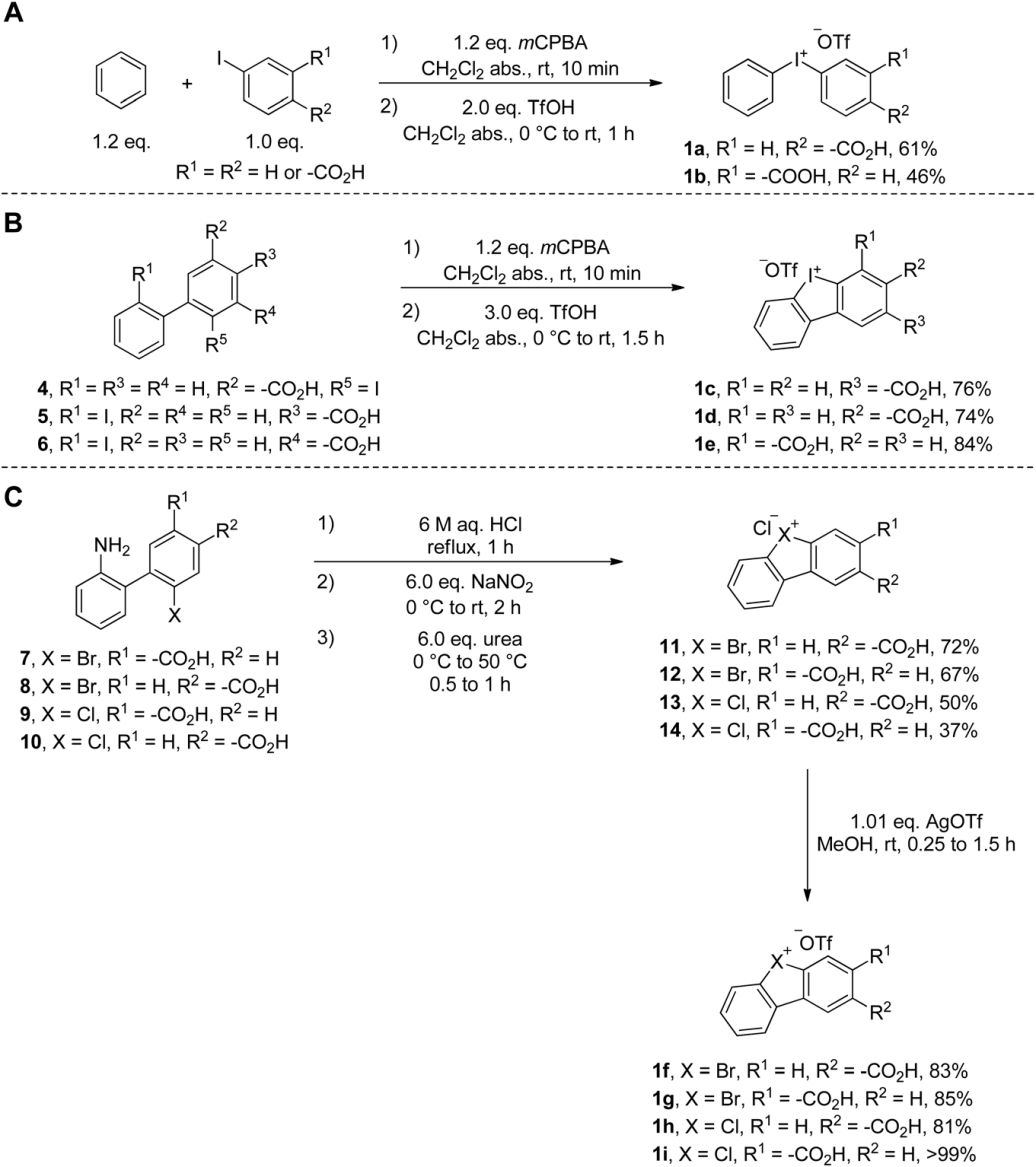
Synthesis of the warheads. Diphenyliodonium salts (A), diphenyleneiodonium salts (B) and diphenylbromonium and –chloronium salts (C).

Based on these considerations we started our warhead design with the synthesis of diphenyliodonium salts bearing a free carboxylic acid moiety on one of the phenyl rings *meta* or *para* to the hypervalent iodine substituent as a ligation handle (Scheme 1A).^40^ Trifluoromethanesulfonate (triflate) was chosen as the counter anion because of its weakly coordinating character and the good solubility properties of the corresponding salts in organic solvents. The synthesis of salts 1a–b succeeded with conditions adapted from Bielawski *et al.*,^22^ starting from the corresponding iodobenzoic acids. Diphenyleneiodonium salts 1c–e could be synthesized under the same conditions in 74–84% yield (Scheme 1B). As unsymmetrical acyclic diarylhalonium probes might lack selectivity in terms of which radical is ultimately formed (with/without linker and click handle) and labeling the enzyme and synthesis of acyclic diarylbromonium and –chloronium salts is rather difficult, only diphenylene-based λ^3^-bromanes and λ^3^-chloranes were used as warheads in this study.

**Fig. 2:**
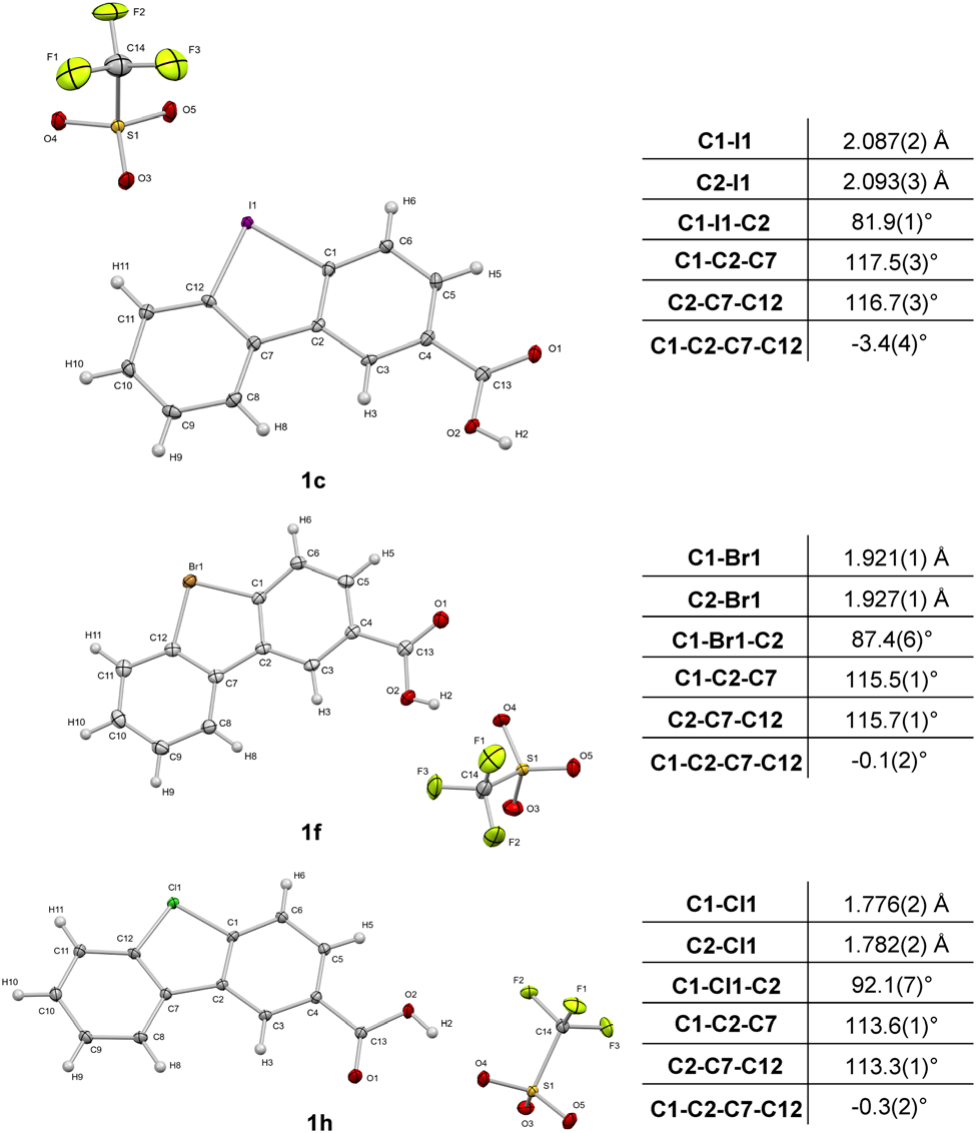
Crystallographic analysis of 1c (CCDC 2145617), 1f (CCDC 2149555) and 1h (CCDC 2145616).

Diphenylenebromonium triflate salts 1f–g and diphenylchloronium triflate salts 1h–i bearing the carboxylate ligation handle were synthesized according to a procedure adapted from the literature,^21,33^ starting from the corresponding amino-halo-biphenyl *via* diazonium-formation and subsequent N_2_-extrusion (Scheme 1C). Single crystal X-ray analyses of 1c, 1f, and 1h were conducted, corroborating the structural trends in the order from I(iii) to Cl(iii) presented recently by Wencel-Delord.^34^ Although it is known that the atomic radius increases going from chlorine to iodine, the crucial T-shaped structure of the three-center-four-electron feature is maintained in all three λ^3^-hypervalent compounds (Fig. 2). Moreover, the “general size” of the compounds, measured as the distance between atoms C5 and C10, differs only marginally. Going from iodonium salt 1c (7.041 Å) to bromonium salt 1f (7.010 Å) and chloronium salt 1h (6.969 Å), the difference between the largest and smallest structure is 0.072 Å (or ∼1%). The isosteric character of the probes suggests that the differences in the warheads' reactivities should therefore be of electronic origin. With the desired diarylhalonium salts in hand, we set out to synthesize the activity-based probes. For this purpose, different linkers, bearing a terminal azide as a versatile click-handle for the attachment of fluorophores for visualization or biotin for enrichment studies, were attached to the diarylhalonium salts at the carboxylate moiety *via* amide formation.

**Scheme 2:**
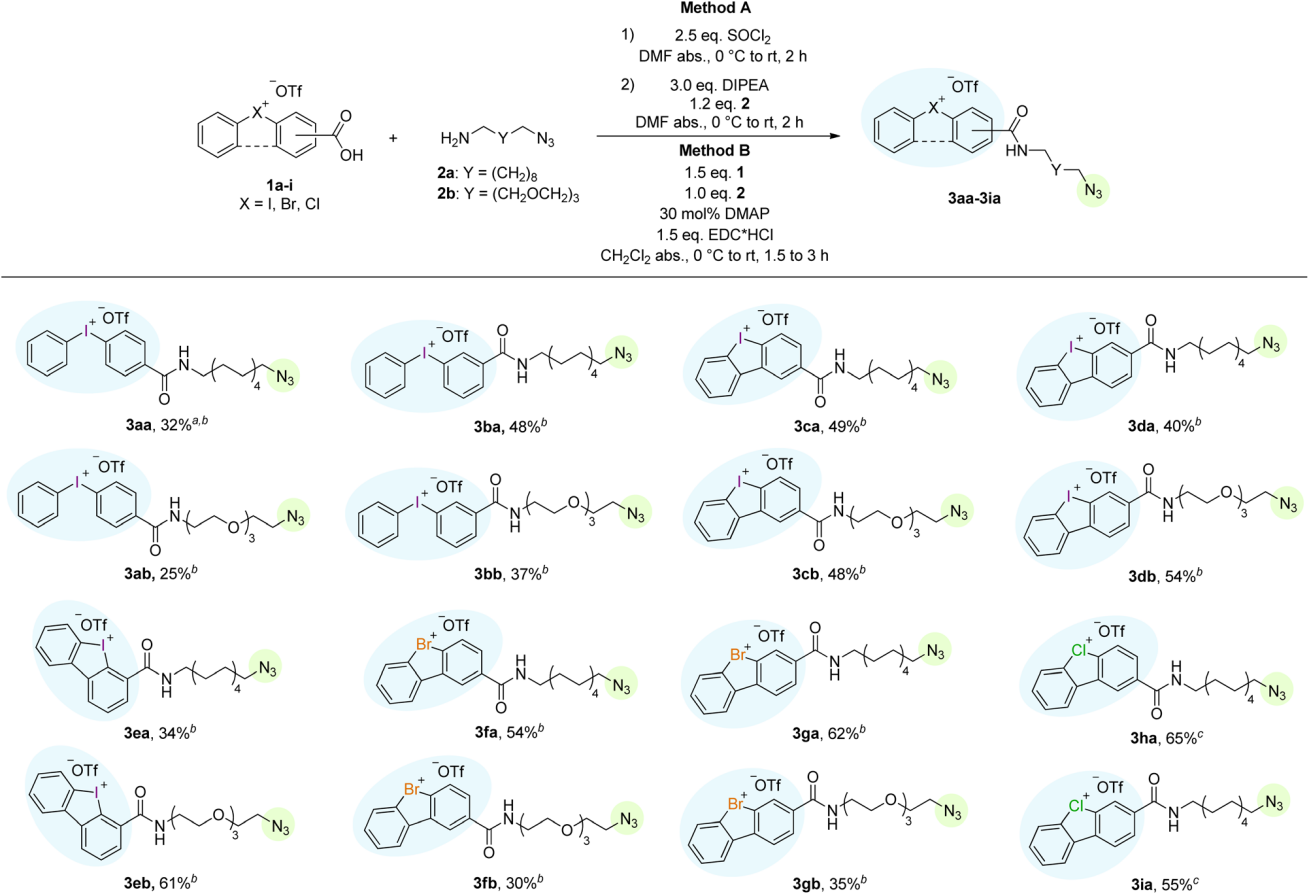
Synthesis of activity-based probes from diarylhalonium salts and azido-amine linkers. ^*a*^ Isolated yields after column chromatography. ^*b*^ Method A was used. ^*c*^ Method B was used. Warheads are highlighted in blue, reporter tag click-handles are highlighted in green.

In preliminary studies, probes with longer linkers performed better compared to short-linker-probes, therefore only aliphatic linkers with chain length ≥10 atoms were used in this study and synthesized according to standard amide coupling procedures.^41^ The best coupling method for the diaryliodonium and diarylbromonium salts was the conversion of the carboxylic acid into an intermediary acyl chloride with a slight excess of SOCl_2_ in *N*,*N*-dimethylformamide (DMF), which upon removal of volatiles could react with the azido-amines 2a and 2b with *N*,*N*-diisopropylethylamine (DIPEA) as base in DMF (Scheme 2). Remarkably, the compounds proved to be stable during flash column chromatography, yielding the purified, desired diarylhalonium-based AB probes 3aa–3gb in moderate to good yields. Other coupling methods (*e.g.*, using PyBOP, HATU, HBTU or DCC with DIPEA) turned out to be unsuccessful. However, for the synthesis of the diarylchloronium-based probes a different method was applied, as the corresponding intermediary acyl chlorides proved to be unstable under the standard conditions. Therefore, the azido-amine was reacted with a slight excess of the corresponding chloronium salt carboxylic acid and EDC·HCl under DMAP catalysis in CH_2_Cl_2_. Diarylchloronium-based probes 3ha and 3ia could be isolated in good yields after column chromatography (Scheme 2). It should be noted that only the azidoalkylamine 2a was used in this method, as separation from the other highly polar reactants on the flash column was unachievable for the oligoethyleneglycolether reagent 2b. The nomenclature of the probes follows the principle of a modular system, in which the first small letter indicates the used halonium warhead, whereas the used linker is indicated by the second letter.

### Warhead characterization and control experiments

**Fig. 3:**
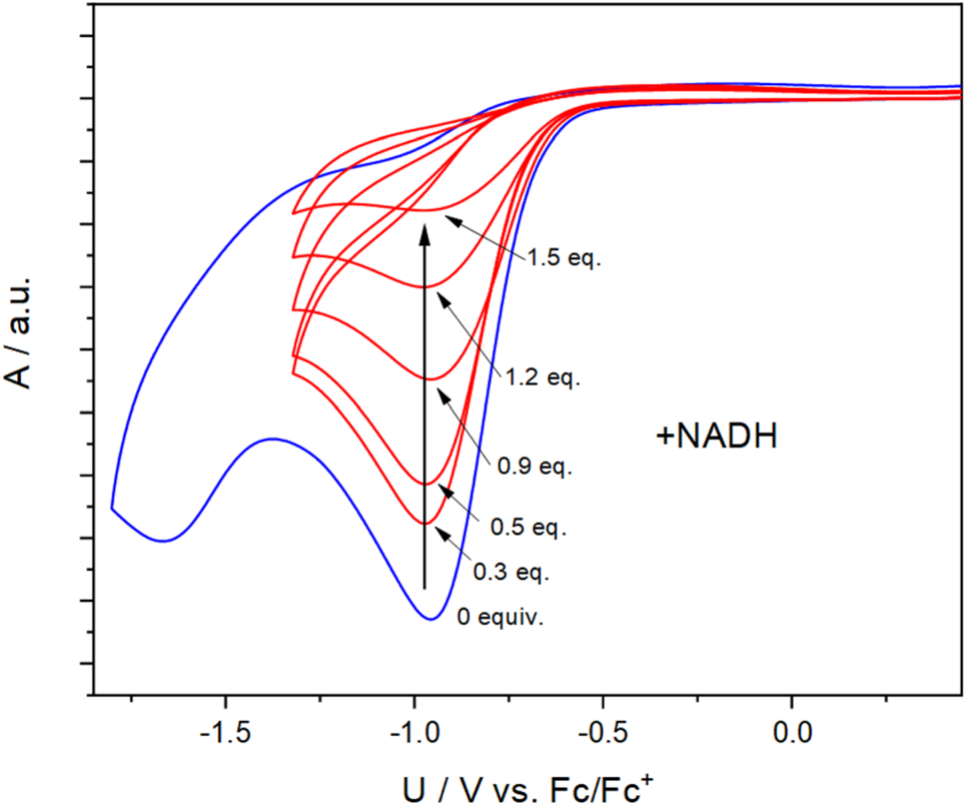
Cyclovoltammogram of 1c (blue curve, 30 mM concentration, solvent: MeCN, scan rate: 500 mV s^−1^, supporting salt: Bu_4_NClO_4_ (0.1 M), working electrode: Pt). The red curves indicate the decrease of the reduction peak upon addition of NADH.

**Scheme 3:**
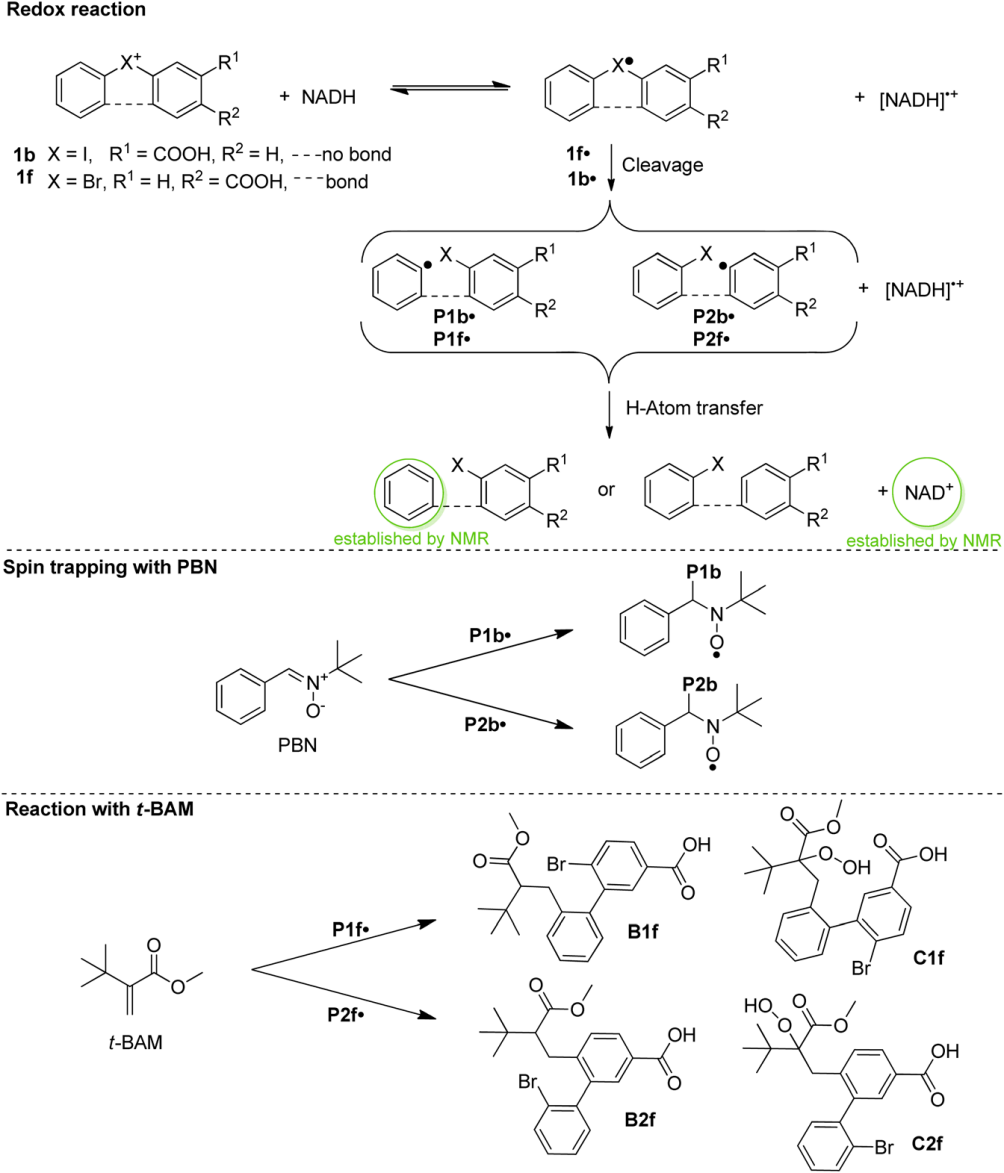
Electron transfer reaction, spin trapping with PBN, and quenching with methyl *t*-butylacrylate (*t*-BAM).

**Fig. 4:**
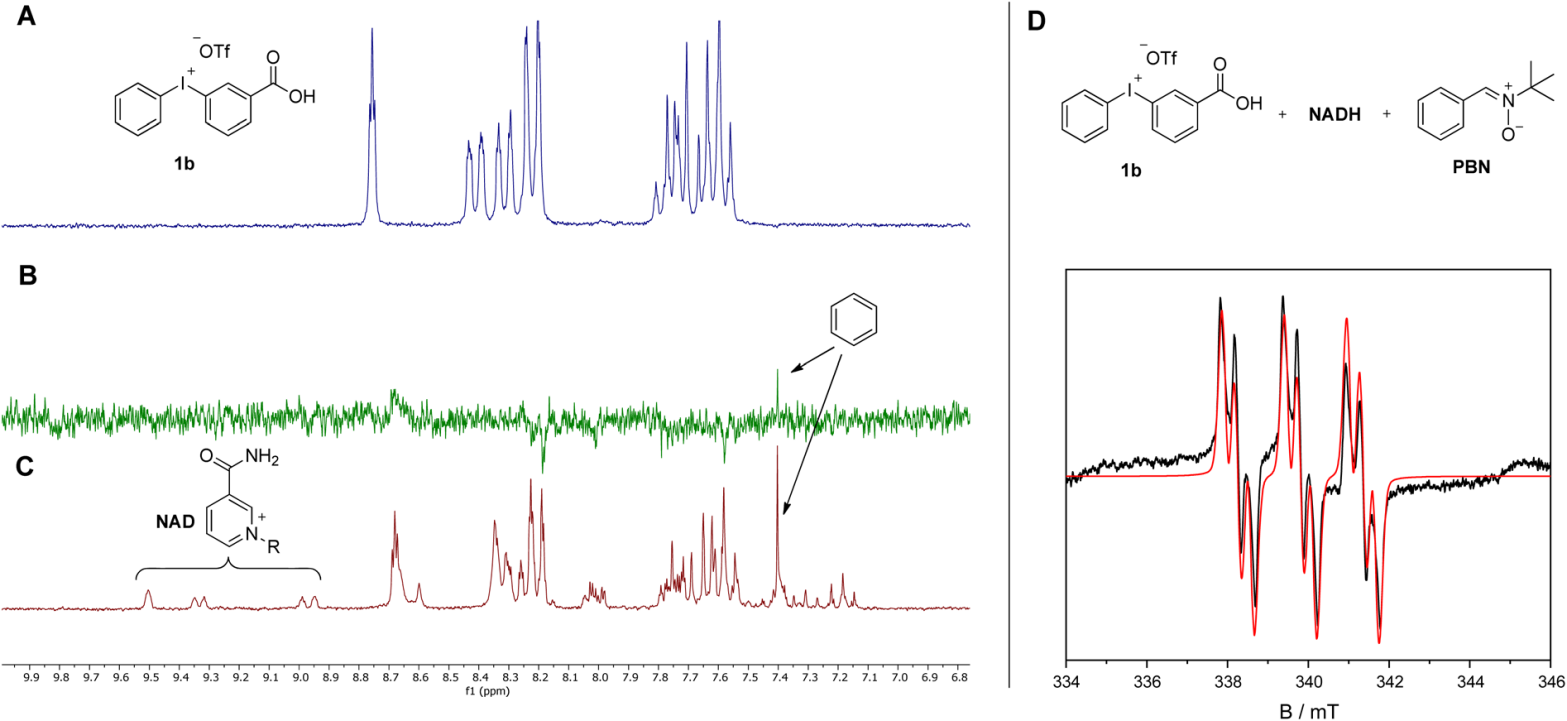
(A) ^1^H-NMR spectrum of 1b (methanol-*d*_4_/D_2_O at 200 MHz). (B) ^1^H-CIDNP spectrum recorded 30 min after mixing 1b and NADH. (C) ^1^H-NMR recorded 30 min after the spectrum B. (D) CW-EPR during the reaction of 1b, NADH, and PBN overlaid with the simulated spectrum.

To obtain insight into the reactivity of the iodonium, bromonium, and chloronium warheads upon one-electron reduction, we determined the reduction potentials of 1b–1d and 1f–1i (Table 1). The differences between the *E*_pc_ values likely originate from the nature of the halogen atom and the differing solvation/character of the ion pairs [(diaryl halonium)^+^ OTf^−^] (see also Fig. S1†).^42^ The cyclovoltammograms generally show irreversible shapes (scan rates 100–500 mV s^−1^) with peak potentials, *E*_p_, between *ca.* −1.3 to −0.7 V *vs.* Fc/Fc^+^ in line with published data on related iodonium salts.^43^ As an example, Fig. 3 (blue curve) shows the cyclovoltammogram of 1c (see Fig. S2–S7 for a complete set†) pointing to a rapid decomposition of the primarily formed radical 1c˙ generated by one-electron reduction of the salt 1c (see Scheme 3 for the analogous formation of 1b˙). To establish that NADH, serving as a representative cofactor for oxidoreductases, is able to accomplish the one-electron reduction of the halonium warheads, we have gradually added NADH to the solution of 1c used for the cyclovoltammetric experiment. This led to a steady decrease of the reduction peak of 1c with no additional peaks emerging in the voltammogram (Fig. 3, red curves). This indicates that NADH is a suitable reducing agent for 1c. To provide insight into the mechanistic background of the reduction, we reacted 1b, 1g, and 1h with NADH (1.5 equiv.) in methanol-*d*_4_/D_2_O and recorded the corresponding ^1^H-NMR spectra; a representative example is shown in Fig. 4A for 1b. The blue trace shows the spectrum of parent 1b. Immediately after the addition of NADH we have saturated the signals (7.1–9.6 ppm, parent 1b; for experimental details see ESI†). This procedure led to the emergence of a weak singlet peak at 7.4 ppm just outside the range of the NMR signals attributed to 1b (green trace, Fig. 4B). This peak is well attributable and corresponds to benzene protons. We ascribe this enhanced absorption to a thermal CIDNP effect^44^ based on the redox reaction between NADH (reducing agent) and 1b generating the primary radical pair NADH˙^+^/1b˙ (Scheme 3) and its spin-dependent follow-up reactions.^45^ This observation is in line with the formation of benzene radical P1˙ upon reduction by NADH followed by cleavage and [NADH]˙^+^ is converted to NAD^+^ (these radical-pair-based reactions cause a non-Boltzmann population of magnetic states resulting in enhanced absorption (observed in our case) or emission). Benzoic acid, based on radical P2˙ is not detectable by CIDNP because the multiplet nature of its NMR spectrum spreads the signal intensity among four lines between *ca.* 7.5 and 8.2 ppm being below our detection limit. After *ca.* 30 min, several new/superimposed signals emerge. The five resonances between 8.9 and 9.6 ppm (red trace in Fig. 4C) are attributable to the aromatic protons of NAD^+^.^46^ The intense singlet at 7.4 ppm can straightforwardly be assigned to benzene protons. Additional evidence for the formation of radicals based on the reductive cleavage of the iodonium, bromonium, and chloronium warheads follows from spin-trap experiments. When we reacted the diarylhalonium salts with NADH in the presence of PBN (alpha-phenyl *N-tert*-butyl nitrone) as the spin trap, we could record well distinguishable EPR spectra. Fig. 4D displays the EPR spectrum recorded upon mixing 1b, NADH, and PBN in methanol/H_2_O (see Fig. S8 and S9† for spectra of analogous reactions with 1g and 1h). It consists of two components, one attributable to the PBN adduct of P1b˙ (^14^N hyperfine coupling constant, hfc 1.54 mT, ^1^H hfc = 0.22 mT the other of P2b˙ (^14^N hfc = 1.52 mT, ^1^H hfc = 0.23 mT), P1b˙/P2b˙ = 47 : 53 in line with published data).^47^ In addition, we performed the redox reaction between 1f and NADH in the presence of an excess of freshly prepared (for experimental details see ESI†) methyl *t*-butyl acrylate (*t*-BAM). It is established that phenyl-type radicals not only abstract hydrogens (Scheme 3) but also can add to double bonds initiating a radical polymerization. The use of the bulky monomer *t*-BAM causes a termination of the potential polymerization already after the first addition of the radical to the monomer. This allows analyzing low-molecular weight products with mass spectrometry. Indeed, the radicals P1f˙ and P2f˙ are the end groups of the major products, B1f and B2f, as well as the hydroperoxide-adducts C1f and C2f (deriving from quenching by airborne/dissolved oxygen), respectively (Scheme 3 and Fig. S10†). Analogous experiments performed with chloronium warhead 1h and iodonium warhead 1a revealed matching results confirming the reductive cleavage of the diarylhalonium warheads and the formation of radical intermediates (Fig. S11 and S12†). Altogether, these experiments give strong evidence for the generation of radical intermediates upon reductive activation of our arylhalonium warheads.

**Table 1:** Peak reduction potentials (in V; *E*_p_/V *vs.* Fc/Fc^+^) of 1b–d and 1g–i

Compound	Halogen	Solvent	
		MeOH	MeCN
1b	I^+^	−0.72	−0.78
1c	I^+^	−0.72	−0.91
1d	I^+^	−0.97	−0.88
1f	Br^+^	−1.28	−1.19
1g	Br^+^	−1.07	−1.12
1h	Cl^+^	n. d.^a^	−0.95
1i	Cl^+^	−0.79	−0.92

a n. d. = not detectable.

An alternative – and for our endeavor undesired – mode of reactivity could be the reaction of the electrophilic arylhalonium warheads with nucleophilic amino acid side chains. We therefore tested the susceptibility of our halonium warheads to thiol nucleophiles in a model experiment with glutathione – the most abundant thiol nucleophile in cells (Fig. S13†).^48^ Members of each diphenylenehalonium salt class were reacted under near physiological conditions (aqueous environment, neutral pH, 40 °C reaction temperature) with the Cys-containing oligopeptide glutathione (GSH). However, even after 3 h reaction time – which is even longer than the incubation time during the ABPP experiments – no GSH-halonium-adducts but only unreacted starting materials could be detected by HPLC. Based on these results, the warheads do not seem to be highly susceptible to thiols from common cysteine residues. However, an electrophile–nucleophile reactivity cannot be fully ruled out. Due to the p*K*_a_ of their free thiol (∼8–9) and the resulting enhanced ionization probability compared to other nucleophile bearing amino acids, cysteines exhibit an intrinsically high nucleophilicity.^49^

### *In vitro* ABPP experiments with isolated oxidoreductases

**Fig. 5:**
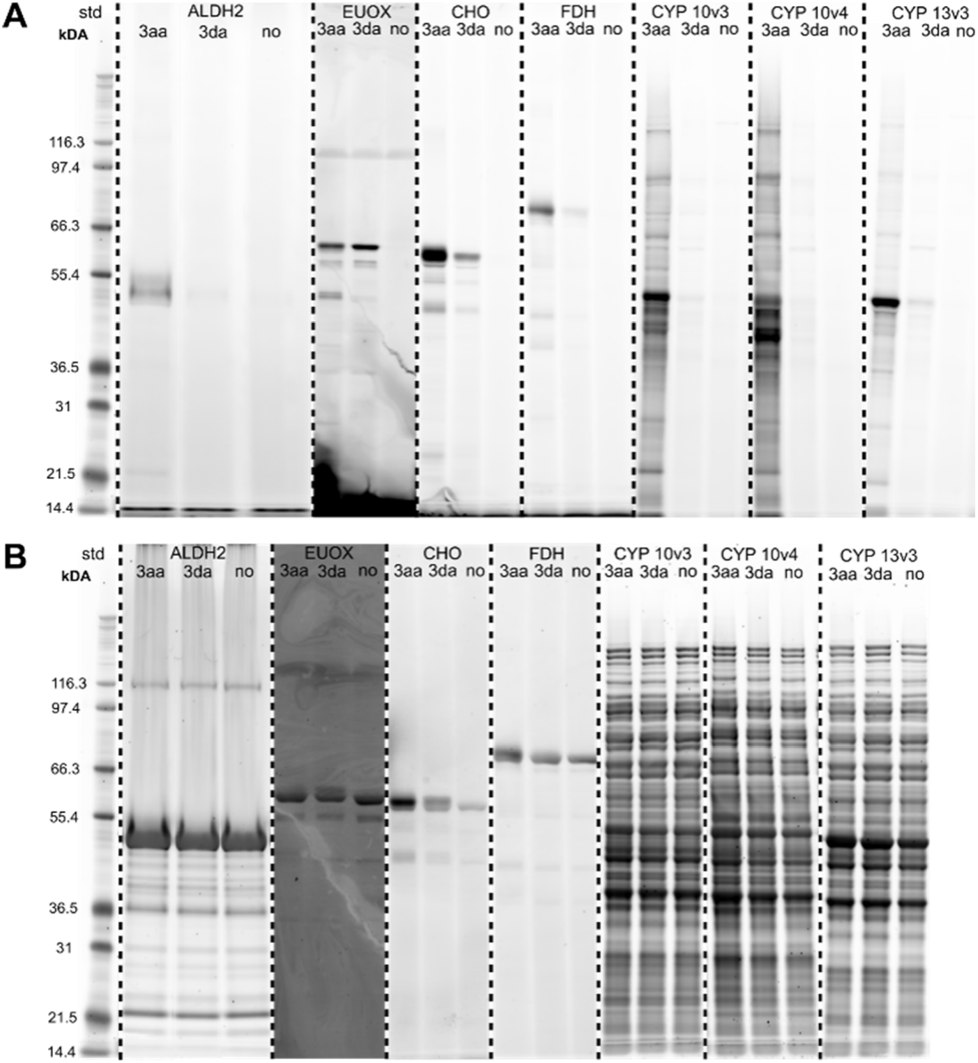
(A) Fluorescent SDS-PAGE analysis of the labeling of various recombinant enzymes with probes 3aa and 3da (30 μM, 1 h; no = no probe added). Protein bands were visualized by fluorescent staining. (B) Total protein stains of activity-based gels. Proteins were visualized with Krypton fluorescent protein stain. ALDH2 = aldehyde dehydrogenase 2 (recombinant human), EUOX = eugenoloxidase (*Rhodococcus jostii*), CHO = choline oxidase (*Arthrobacter nicotianae*), FDH = formate dehydrogenase (*Clostridium carboxidivorans*), CYP 10v3/10v4 = cytochrome P450 10v3/10v4 (*Phenylobacterium zucineum*), CYP 13v3 = cytochrome P450 13v3 (linalool 8-monooxygenase; *Mycobacterium intracellulare*). CYP enzymes were used as crude cell lysates. Mark 12™ Unstained Standard (Thermo Fisher Scientific) was used as protein standard (=std).

After having collected strong evidence that our warheads form radicals during redox activation, we tested in a first series of ABPP experiments probes 3aa and 3da in the labeling of recombinant enzymes. We observed that 3aa – with 3da to a lesser extent – led to the labeling of several of the tested oxidoreductases featuring different cofactors, such as choline oxidase (FAD), eugenol oxidase (FAD), lactate dehydrogenase (NAD), ALDH2 (NAD), linalool 8-monoxygenase (heme), CYP10V3 (heme) (Fig. 5), thereby confirming the ability of our probe design for the covalent labeling of diverse oxidoreductases. Notably, the acyclic probe 3aa led to efficient labeling although the radical could also be formed on the aryl group without linker attachment. Moreover, upon denaturation of selected enzymes with heat (+1% SDS) prior to incubation with probe 3aa no or much less intense lanes could be observed on the activity-based gel (Fig. S14†), which further confirms the ability of the probe to only react with the enzymes being in their active state. In all cases, proteins were incubated with the probes for 1 h to ensure sufficient labeling and probes were used in 30 μM concentration, which was found to be ideal in probe concentration-dependent ABPP experiments with the reductase NAD(P)H dehydrogenase [quinone] 1 (NQO1) and iodonium probe 3aa (Fig. S15†).

In additional *in vitro* ABPP experiments with NQO1 and all diarylhalonium probes (3aa–3ia; 40 μM, 1 h), we could observe labeling for several different probes throughout all subclasses (Fig. S16 and S17†). Again, without the addition of NADH prior to the addition of the probe or upon preliminary denaturation with 2% SDS we observed complete loss of the labeling (Fig. S18†), which further supports our postulation that only by active enzymatic reduction the probes are activated. Notably, pre-incubation with dicoumarol – a known pharmacological inhibitor of NQO1 (ref. 50) – resulted in a substantially decreased labeling of NQO1 with probe 3aa (Fig. S15†). Concerning the labeling capability of the probes for NQO1, their accessibility to the flavin active site seems to be the most important factor, which is mainly influenced by the linker position. NQO1 was chosen as the ideal candidate, as it was found to promote exclusively reductions of several compounds responsible for quinone levels, thereby minimizing opportunities for generation of reactive oxygen intermediates by redox cycling, and for depletion of intracellular thiol pools.^51,52^ Furthermore, in the FAD-dependent NQO1 electrons are sequentially transferred from NAD(P)H to the flavin cofactor and then from the reduced flavin to the substrate.^52,53^ Only in its active and uncompromised state NQO1 can accept electrons from NAD(P)H and interferences in this process will result in loss of function.^54^ The loss of enzyme activity upon labeling by our probes was further confirmed in spectrophotometric activity assays with NQO1 and probe 3aa (Fig. S19†).

We also looked at the reaction of the flavin cofactor of NQO1 with selected probes (3aa, 3ga, and 3ha) in UV-Vis spectroscopy experiments (Fig. S20 and S21†), which revealed a direct interaction of the cofactor with our probes and formation of flavin-probe adducts.

### *In cellulo* ABPP experiments with murine liver

As an ABPP probe also needs to show its value with samples of higher complexity and with proteins of natural abundance, we therefore tested all synthesized activity-based probes (3aa–3ia) for their performance in ABPP studies using fresh murine liver, which was chosen as the object of investigation as it is the central organ in mammals for metabolic oxidation processes and therefore encompasses a high diversity and high expression levels of oxidoreductases. When performing SDS-PAGE and in-gel fluorescence scanning after initial incubation of intact small pieces of mouse liver with PBS containing either probes or no probe (negative control),^40^ we found that several probes effectively labeled the liver proteome confirming their membrane permeability and reactivity. The selectivity of the probes varied depending on the specific probe designs (Fig. S22†). There was already a clear trend visible by fluorescent gel electrophoresis regarding the polarity and position of the linker, as well as the halonium ion in the warhead. Probes 3ea and 3eb showed almost no labeling activity, which might be caused by either steric problems imposed by the shape of the probes in reaching the active site or by the impossibility of the attached linkers to reach outside of the binding pocket of the enzymes, which is important for the follow-up attachment of the fluorophore.

### Enrichment studies/target protein identification

**Fig. 6:**
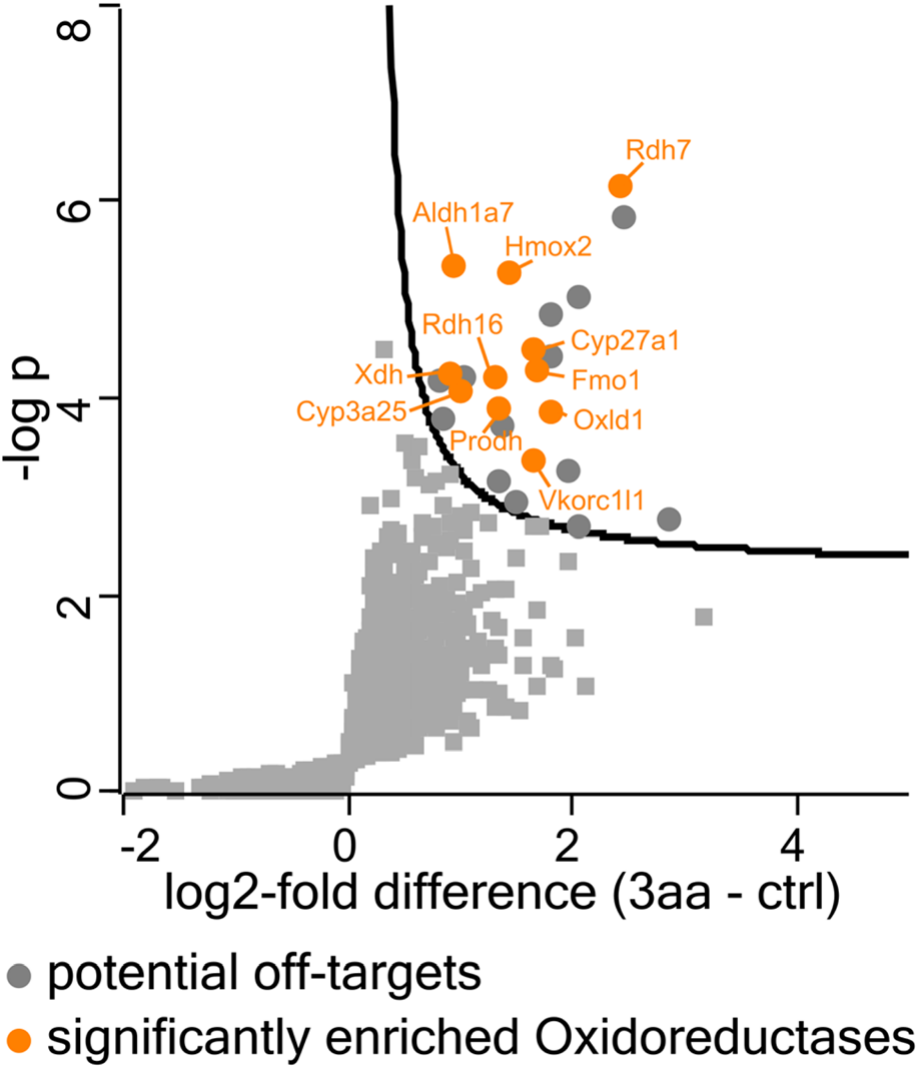
One-sided volcano plot for the labeling of murine liver with diphenyliodonium probe 3aa (30 μM, 1 h) showing the distribution of quantified proteins according to –log *p* value and log2-fold difference of proteins in probed (with probe 3aa) *vs.* non-probed samples (*n* = 6). Proteins above the line are considered statistically significant (adjusted *p*-value <0.01). Oxidoreductases are annotated.

In order to evaluate whether the diarylhalonium-based probes do in fact label oxidoreductases we conducted protein enrichment assays with all 16 synthesized probes. After incubation of ABPP probes with murine liver pieces (*n* = 6), whereby each individual mouse liver tissue was treated with all probes (30 μM probe concentration, 1 h incubation time) including a non-probed control to rule out bias resulting from phenotypic differences between individual mice. The labeled, precipitated, resolubilized, and reduced protein was reacted with a dibenzocyclooctyne (DBCO)-TEV-biotin strain-promoted click linker (Fig. S23; for full experimental and statistical details see ESI and ESI Tables†). After the click-reaction was performed, labeled proteins were enriched on streptavidin–agarose resin. Following washing steps and on-bead tryptic digest, the protein digests were analyzed by LC-trapped ion mobility-MS/MS, employing data-independent acquisition, and subsequent search of the data with the Swiss-Prot murine database and common contaminants for identification and label free relative quantitation. First, we performed statistical analysis of probed *vs.* non-probed samples using multiple comparison corrected pairwise test (FDR = 5%) in Perseus. The results strikingly underline the usefulness of our diarylhalonium-based probes for the ABPP of oxidoreductases. Several different proteins were significantly enriched with our probes 3aa–3ia (see Fig. 6 for a representative example, Fig. S24–S38 for the complete set†) compared to the unprobed samples, with the majority being annotated as oxidoreductases (see Table S1 for comparison of significant fold change values†). Three out of our 16 probes proved to be highly versatile. Diphenyliodonium probe 3aa (Fig. 6) labeled a total of 24 proteins, of which 10 are annotated as oxidoreductases of different subclasses (plus one oxidoreductase-like protein) and 12 currently unannotated proteins. The annotated oxidoreductases include the flavin-containing monooxygenase 1 (FMO1), aldehyde dehydrogenase cytosolic 1 (ALDH1A7), cytochrome P450 monooxygenases (CYP3A25, CYP27A1), retinol dehydrogenases (RDH7, RDH16), or heme oxygenase 2 (HMOX2).

Diphenylenebromonium probe 3fb, also bearing the PEG-linker, engaged in the significant labeling of 19 proteins (Fig. S34†), of which eight proteins are not assigned to a specific enzyme class. Notable oxidoreductases (*n* = 9) are several CYP450s (CYP27A1, CYP2D10, CYP3A25, CYP2D26), STEAP4, an integral membrane metalloreductase, which uses both iron and copper as substrates and plays a role in the cellular uptake of these metals,^55^ NADH dehydrogenase [ubiquinone] flavoprotein 1 (NDUFV1), which belongs to complex I of the respiratory chain,^56^ and monoamine oxidase A (MAOA), with the last two represented exclusively for this probe (Table S1†). The third highly versatile probe is diphenylenechloronium-based probe 3ha. With this probe a total of 22 enzymes could be significantly enriched, of which eight are already annotated as oxidoreductases and 11 are currently unannotated (Fig. S37†). These are, in particular, different aldehyde dehydrogenase family members (ALDH1A7, ALDH8A1, and ALDH3A2), CYP450s (CYP2D10, CYP27A1), choline dehydrogenase (CHDH), which is similar to the bacterial choline oxidase used in our recombinant enzyme studies, carotenoid-cleaving dioxygenase (BCO2), and heme oxygenase 2 (HMOX2).

Diphenyleneiodonium-based probe 3da significantly enriched only 7 proteins (Fig. S29†), of which, however, five are oxidoreductases (CYP27A1, CYP2D10, CYP3A25, HMOX2, and AOX1) and the other two are unannotated (BOLA3, MCAM). Three of these oxidoreductases (CYP27A1, CYP2D10, and HMOX2) could also be significantly enriched by the structural isomer 3ca (Fig. S27 and Table S1†), with no other proteins enriched, resulting in the fact that this probe seems to work exclusively with oxidoreductases. Almost as exclusively for oxidoreductases worked diphenylenebromonium-based probe 3ga, with two significantly enriched CYP450s (CYP27A1, CYP2D10) and glycerol-3-phosphate dehydrogenase mitochondrial (GPD2) out of four proteins in total (Fig. S35 and Table S1;† unannotated BOLA3 was enriched as well). All other probes engaged in either significant labeling of off-targets (*i.e.* EC 2–7), proteins that are currently not assigned to an enzyme class (they could, however, be on-targets), or no significant labeling at all under our statistical constraints.

Although some oxidoreductases (*e.g.*, CYP27A1, CYP2D10) or unannotated proteins (*e.g.*, BOLA3, MCAM) could be enriched with all kind of probes (iodonium, bromonium, or chloronium cation; aliphatic or PEG-linker; see Table S1†), for others the reduction potential of the halonium ion might play a role. For example, HMOX2 was significantly enriched by diaryliodonium probes 3aa, 3ca, and 3da, as well as by diarylchloronium probe 3ha, a structural analogue of 3ca. On the other hand, GPD2 was exclusively enriched by diarylbromonium probe 3ga, and MAOA exclusively by diarylbromonium probe 3fb. The subtle solvent effects observed in the cyclic voltammetry measurements of the warheads (Table 1 and Fig. S1†) might point to implications of the respective microenvironment within different proteins on the redox potential of the individual probes.

Also, the type of linker and the site of attachment seems to play an important role for the reactivity of the probes. Probes bearing the PEG-type linker (Scheme 2, 3ab–3gb; with the exception of 3fb) performed insufficiently compared to their aliphatic linker analogues (Scheme 2, 3aa–3ia; see also Table S1†), and some proteins were only significantly enriched with probes bearing the aliphatic linker (*e.g.*, HMOX2) or PEG-linker (*e.g.*, URB1), respectively (Table S1†). Moreover, probes bearing the linker in *ortho*-position to the bridging halonium ion (*i.e.*3ea and 3eb) seemed unable to engage in significant labeling as shown already by SDS-PAGE, which was confirmed in the enrichment experiments.

**Fig. 7:**
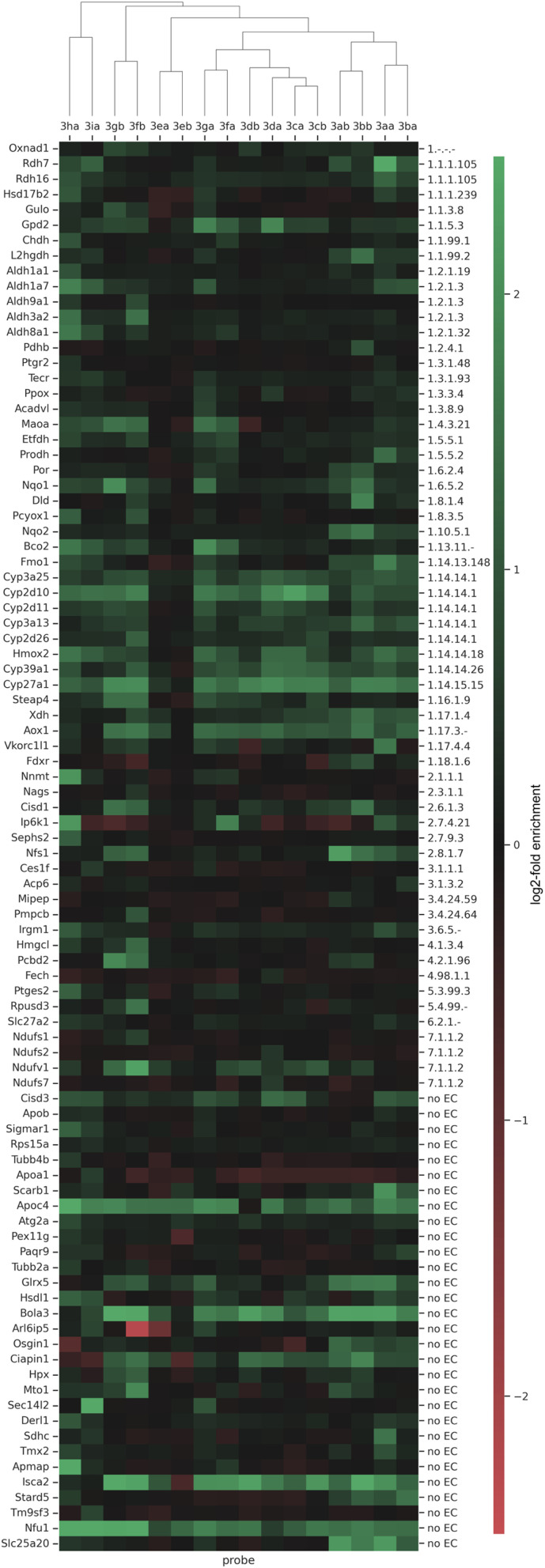
Hierarchical clustering and heat map, demonstrating the clusters with an abundance-scale (log2-fold enrichment). A total of 92 significant proteins (OXNAD1 to SLC25A20) are represented. Proteins present in higher amounts are shown in green, while those in lower amounts appear in red. The column tree shows the clustering of the probes 3aa–3ia (Scheme 2) based on their different profiles of enriched proteins.

In order to even further compare the differences of the probes in the enrichment of the proteins we performed a hierarchical cluster analysis with Euclidean distance of ANOVA significant proteins (Fig. 7). With this, a total of 92 significant proteins could be identified in significant pairs of probe x *vs.* probe y or probe x *vs.* no probe, including 41 annotated oxidoreductases (EC 1) and 30 proteins not assigned to an enzyme class, which might possess a currently undisclosed oxidoreductase activity. In the depicted dendrogram, probes sharing an earlier node point also exhibit more similar labeling activity – information, which can be gathered from the heat map. Notably, we found that our probes, which are per definition automatically clustered based on their labeling activity with statistically significant proteins, are also clustered based on their design, where the type of linker, as well as the position, seem to have a large influence.

Acyclic diaryliodonium probes are initially paired based on the linker design (aliphatic linker: 3aa/3ba, PEG-linker: 3ab/3bb), but are subsequently merged in the second level of hierarchy. Together, the acyclic diaryliodonium probes exhibit similar labeling activity. For example, they all labeled CYP450 isozymes CYP3A13 to CYP27A1, FMO1, and, very interestingly, ribosyldihydronicotinamide dehydrogenase [quinone] (NQO2). The FAD-containing NQO2 was the first (and remains until now the only) example of a true reductase (*i.e.* in nature exclusively catalyzing a reduction of a substrate using electrons from cofactors such as NADH, *etc.*) being targeted by an ABPP approach using hydrazine probes.^16,57^ In addition, we could also identify NAD(P)H dehydrogenase [quinone] 1 (NQO1), which now marks the second example of a true reductase being targeted by an ABPP approach, suggesting that our probes are indeed activated in a reductive manner.

Furthermore, the two retinol dehydrogenases RDH7 and RDH16 were found to be labeled by probe 3aa very well. The cyclic diaryliodonium probes were clustered based on the position of the linker. Probes 3ca and 3cb, which bear their respective linker in the *para*-position to the iodonium cation, are the most similar regarding their respective labeled proteome (mainly CYP450 isozymes, yet more different potential off-targets compared to the acyclic probes), followed by cyclic diaryliodonium probes 3da and 3db (respective linker in *meta*-position). Cyclic diaryliodonium probes 3ea and 3eb, which bear their linkers in the position *ortho* to the iodonium cation, were clustered separately, with no distinct labeling activity observable. This is in accordance with the observations in the SDS-PAGE analysis and the data from the pairwise analyses of the probed *vs.* non-probed samples, where they already did not show any significant labeling/enrichment activity. In the next level above the cyclic diaryliodonium probes, cyclic diarylbromonium analogues 3fa and 3ga join the hierarchical branch, which seem to label protein clusters more similar to all diaryliodonium probes (3aa–3db) and less similar to their PEG-linker-bearing counterparts (3fb and 3gb). In addition to all CYP450s and glycerol-3-phosphate dehydrogenase [NAD^+^] 2 (GPD2), they were also found to label the enzymes in the cluster from TECR to NQO1.

Diarylbromonium probes bearing the PEG-linker (3fb and 3gb) are clustered more separately from all already discussed probes. This might be attributed to the combination of them bearing the PEG-linker and the bromonium core-unit, which was found to exhibit a slightly lower peak reduction potential compared to the iodonium and chloronium analogues (Table 1). Again, apart from many CYP450s, they also labeled several enzymes in the aldehyde dehydrogenase cluster (ALDH1A1 to ALDH3A2) and MAOA. However, the main difference compared to the other diarylbromonium and diaryliodonium probes seems to stem from the labeling of off-targets (*e.g.*, PCPB2 or NDUFV1) and unannotated proteins (*e.g.*, HPX, MTO1). As can be seen in the heat map and hierarchical clustering (Fig. 7), the diarylchloronium probes 3ha and 3ia were found to overall label most differently from all other probes. In the oxidoreductase section, many small clusters are visible. These include the CYP-, ALDH-, and RDH-clusters, and enzymes like HSD17B2, BCO2, or PCYOX1. Especially probe 3ha therefore seems to be one of the most versatile probes in our set. In addition to our oxidoreductase targets (EC 1), our probes also labeled off-targets of other enzyme classes (EC 2–7) and many proteins without EC-annotation (Fig. 7). Some of these proteins (*e.g.*, BOLA3, ISCA2, NFU1) were found to be significantly labeled by more or less all probes. Interestingly, a large number of these non-oxidoreductase proteins (including the mitochondrial membrane respiratory chain NADH dehydrogenase subunits NDUFS2 to NDUFV1) contain Fe–S clusters. Fe–S clusters, like ferredoxin, act in enzymes for example as electron-transfer reactants.^58^ Hence, these clusters, if in a reduced form, could potentially catalyze a single electron transfer to the probe warhead, which would lead to the formation of a radical (as proposed by our mechanistic studies) and subsequent non-selective binding of the probe. Thus our probes may complement chemoproteomic approaches targeting Fe–S cysteine occupancy for identification of Fe–S proteins.^59^ Generally, one could hypothesize, that our probes might also engage in the labeling of off-target proteins when being present in very close vicinity to an active oxidoreductase where electrons could be transferred between molecules.

### Role of nucleophilic amino acid residues

Although our previously discussed experiments did not show any electrophilic reactivity with glutathione as a nucleophile, we still consider this mechanistic principle as a possible competing pathway of labeling since Kumari *et al.* recently proposed that the attack of a Cys residue of the target protein on the iodonium group may lead to formation of the adduct.^17,35^ Cravatt and Baker have adopted a strategy called isotopic tandem orthogonal proteolysis – activity-based protein profiling (isoTOP-ABPP) in order to quantify the reactivity of cysteines in proteomes.^60^ With their approach they were able to identify ‘hyper-reactive’ cysteines, which showed complete labeling even at low concentrations of an iodoacetamide probe. By comparing the gene names in the large dataset (>1000 human and >160 murine Cys-containing proteins) created in their study with the gene names of proteins labeled and enriched with the diarylhalonium probes in our experiments we found that only one ‘hyper-reactive’ cysteine – (*R*_10:1_ ≤ 2, as termed by Cravatt and Baker^60^) and 7 other reactive cysteine (*R*_10:1_ 2–13) containing proteins (oxidoreductases and other enzyme classes) were indeed targeted (see Table S2†). However, the majority (*n* = 6) are proteins that were only slightly enriched and are not found in the main probe clusters. We also checked for highly reactive lysine residues among our enriched proteins. Again, by comparing a huge dataset (>4000 human Lys-containing proteins) – also generated by the group of Cravatt in a follow-up study^61^ – we found only two enriched proteins containing lysine residues with high reactivity (*R*_10:1_ ≤ 2), but 13 proteins with Lys of only medium to low reactivity (2 < *R*_10:1_ ≤ 10) (see Table S3†). Also here, the majority of these proteins were only slightly enriched with our probes and are again not found in the main probe clusters.

As there is only little overlap between the proteins identified with our arylhalonium probes and the data from reactive Cys and Lys, we assume that a large share of the targeted oxidoreductases has been labeled by the proposed reactivity mode (*i.e.* activation by reduction and labeling *via* radical mechanism).

### Identification of labeling sites

Eventually, we also endeavored to identify the modified sites in the labeled proteins to further enhance our understanding of the probes' mechanism of action. For this purpose, the labeled, precipitated and resolubilized protein samples from our previous enrichment studies with murine liver (*n* = 6) and probes 3aa, 3ga, and 3ha*versus* no probe were reacted with a dialkoxydiphenylsilane (DADPS)-biotin alkyne click linker using copper click procedure (Fig. S39A; for full experimental and statistical details see ESI and ESI Tables†). After the click-reaction was performed, labeled proteins were enriched on streptavidin-agarose resin. Following washing steps and on-bead tryptic digest, the labeled peptide fragments were efficiently released under mild conditions (10% formic acid) – thereby only leaving a small molecular fragment of the linker on the labeled peptides (Fig. S39B†) – and analyzed by nano-HPLC-MS/MS, following database search using FragPipe with integrated MSFragger^62^ to identify modified peptides and assign the masses of respective modifications to reactive amino acids.

Modifications could be identified on overall 17 different amino acids in a total of 44 different proteins (see ESI Table 2†). Most importantly, amino acids of all side chain types (*e.g.*, aliphatic, aromatic, nucleophilic, cationic, *etc.*) were shown to be labeled, which corroborates our proposal of radicals to be involved in the mechanism of action, as they would readily react with any kind of molecule in close proximity. Cys, which has already been described to be a labeling site in the proteome for hypervalent iodine reagents,^63^ was found to be a potential labeling site in many of the identified proteins. However, its nucleophilic character clearly makes it a rather easy target for *C*-radical species as well. Furthermore, Cys-labeling could also be the result of *C*-radical disulfide bond labeling, as recently reported by Kielkowski and co-workers.^64^ Recently, the group of Hacker published a proteome-wide study with 54 different electrophilic probes, out of which a set of 17 probes was considered ideal for selectively and globally profiling nine different amino acid residues (Arg, Asp, Cys, Glu, His, Lys, Met, Trp, and Tyr).^65^ While some of these residues were also labeled by our probes, we could additionally identify modifications on amino acids not addressed by their probes (Ala, Asn, Gln, Gly, Ile, Leu, Pro, Ser, Sec, Thr, and Val).

**Fig. 8:**
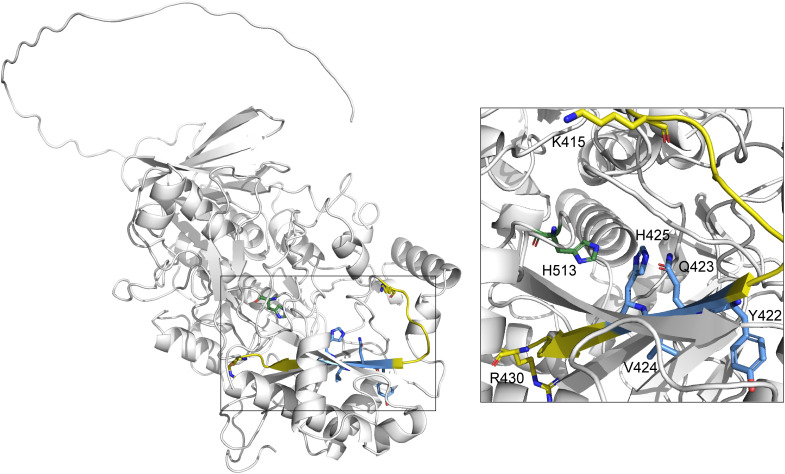
Side and zoomed views of the predicted AlphaFold structure of mCHDH (PDB Q8BJ64) depicting the potentially labeled amino acids (blue, shown in sticks) on the modified peptide fragment (yellow, first and last AA shown in sticks) and important active site residue (green, shown in sticks).

Nine of the proteins for which we could identify the labeling sites were also found to be significantly enriched by our probes in the *in cellulo* enrichment experiments (Table S4†). Together with these proteins and other on-targets (*i.e.* GAPDH, ALDH6A1, ALDH1L1, and MAOB) as well as potential off-targets (MGST1) we had a closer look at the labeling sites on a 3D structural level (crystal structures and AlphaFold^66^ predicted structures) to ascertain whether the labeling occurs indeed in or very close to the protein active site. Choline dehydrogenase (mCHDH), for example, was potentially labeled at residues Y422/Q423/V424/H425 (Fig. 8), which are located directly opposite of the active site residue H513. In the crystal structure of glyceraldehyde-3-phosphate dehydrogenase (mGAPDH) it became evident that the potentially labeled residues are even next to the NAD^+^/NADH binding site right in the active site of the enzyme (Fig. S40†). Examples for nine other enzymes, including oxidoreductases RDH7 and MAOB, as well as different ALDHs, are also in line with a reductive activation taking place in the active site of the respective enzyme followed by labeling in close proximity (Fig. S41–S49†).

To further validate our findings, we had a closer look at the distinct isotopic patterns of MS1 spectra for the hits of bromonium probe 3ga and chloronium probe 3ha. As molecules bearing a bromide- or chloride-residue generally exhibit very unique isotopic patterns, we compared the patterns found in the acquired MS1 spectra with simulations. In fact, the acquired and simulated patterns were found to be identical for the majority of probe-peptide adducts (Fig. S50–S74†), thereby validating our labeling site findings. Only in some cases (*e.g.*, HMGCL, Fig. S57†) acquired and simulated patterns slightly differed, which may be attributed to an isotopic overlap with other molecules.

### Clinical relevance

Throughout the 91 significantly enriched proteins many enzymes play a role in pathophysiological mechanisms. To name a few examples, choline dehydrogenase (CHDH) is associated with choline deficiency^67^ and male fertility.^68^ Aldehyde dehydrogenases (ALDHs) are a family of detoxifying enzymes often upregulated in cancer cells and associated with therapeutic resistance.^69^ Loss of monoamine oxidase A (MAO-A) enzyme function is associated with Brunner syndrome, a disorder characterized by intellectual disability and impulsive, aggressive behavior.^70^ Proline dehydrogenase (PRODH) has been implicated to play a role in hyperprolinemia and schizophrenia.^71^ Nicotinamide adenine dinucleotide (NADH):ubiquinone oxidoreductase (complex I, NDUFV1) enzyme deficiencies account for a significant proportion of mitochondrial disorders, including Leigh Syndrome.^72^ β,β-Carotene-9′,10′-oxygenase 2 (BCO2), which catalyzes the asymmetrical cleavage of carotenoids, is associated with macular degeneration and hepatic steatosis.^73^ Variants of the CYP27A1 gene, which encodes sterol-26-hydroxylase, cause cerebrotendinous xanthomatosis, a rare and underdiagnosed inherited neurometabolic disorder.^74^ Cytochrome P450 2D6 (CYP2D6, an ortholog of murine CYP2D10 and CYP2D26) metabolizes many important drugs and its activity ranges from complete deficiency to ultrafast metabolism depending on at least 16 different known alleles.^75^ Vitamin K epoxide reductase complex subunit 1 like 1 (VKORC1L1) plays a role in blood coagulation and is a target of the anticoagulant warfarin.^76^ Another one of the detected enzymes is xanthine dehydrogenase (XDH). Previous studies have already demonstrated that DIP is an inhibitor of XDH,^37,77^ which was confirmed by us since our DIP-derived probe 3aa labeled XDH with good potency (Fig. 6). This enzyme is responsible for the oxidation of hypoxanthine to xanthine and further to uric acid.^78^ A deficiency of this enzyme (and also of aldehyde oxidase 1 (AOX1), which was also targeted by our probes) can be found in patients suffering from a rare genetic disorder called *xanthinuria*.^79^ Nowadays, xanthine oxidase inhibitors (*e.g.*, allopurinol, tisopurine, febuxostat) are used to treat said conditions. As more or less all approved drugs exhibit either side effects or reduced activity in some patients, the development of new drugs is still ongoing.

The last examples highlight already existing targets for treatment or monitoring of diseases. However, we hypothesize that our new ABPP approach for oxidoreductases with diarylhalonium-based probes could facilitate the identification of new oxidoreductase drug targets in different (native) tissues or in pathogens, as shown recently for carbapenem-resistant *Acinetobacter baumannii*,^35^ and might also help in drug target validation, where other proteomic techniques fail to provide functional information on the actual enzyme activity.

## Conclusions

In conclusion, we have introduced novel diarylhalonium-based probes for the use in activity-based protein profiling of oxidoreductases. A set of 16 probes could be synthesized with bioisosteric warheads of different reduction potential by variation of the central halogen atom (I, Br, Cl). In contrast to the majority of existing ABPP probes for oxidoreductases, these new probes exhibit a more general character, allowing for the simultaneous detection of proteins from different oxidoreductase subclasses, a feature which is currently underrepresented by established probes. Importantly, the new probes were found to work also with “pure” reductases (*e.g.*, NQO2), and engaged in activity-based labeling of clinically relevant oxidoreductases. The reductive formation of aryl radicals could be confirmed *via* CIDNP-NMR, EPR-spectroscopy using spin traps, and in reactions of halonium salts with the bulky monomer *t*-BAM. The reductive activation of the probes and proposed labeling mechanism was further validated in ABPP experiments with recombinant oxidoreductases (*e.g.*, ALDH2, NQO1) and UV-Vis spectroscopy revealed a direct interaction of the probes with flavin cofactors, which constitutes an additional labeling modality for flavin-dependent oxidoreductases. Markedly, the arylhalonium probes could be applied for the *in cellulo* ABPP in murine liver, which is the central organ in mammals for metabolic oxidation processes and therefore encompasses a high diversity and high expression levels of oxidoreductases. Indeed, enrichment studies showed a large share of oxidoreductases among the labeled proteins. The labeling sites of the probes could be characterized for several enzymes *via* MS/MS analysis, showing their location in the active sites. Notably, a large diversity of different amino acid residues including non-nucleophilic amino acids such as Ala, Gly, Ile, Leu, Pro, and Val were labeled by the new arylhalonium probes corroborating the proposed radical mechanism after initial reduction. We see considerable potential for these probes in characterizing the “oxidoreductome” of various species and in the search for new oxidoreductases in biocatalysis, which cannot be annotated by gene sequencing alone.

## Data availability

Experimental details and procedures including biological and synthetic methods, characterization data, whole-protein gel images, protein enrichment data, CIDNP data, CV data, EPR spectra, NMR spectra, X-ray crystallographic data, and data .xlsx files for protein enrichment statistical analyses and labeling site analysis are provided in the ESI.† Full proteomic data are available *via* ProteomeXchange Consortium^80^ partner repository with the dataset identifier PXD043873 and reviewer account details (reviewer_pxd043873@ebi.ac.uk, pw: 785SJoUH). The included data can be obtained free of charge *via*https://www.ebi.ac.uk/pride/archive/ or https://proteomecentral.proteomexchange.org/cgi/GetDataset. CCDC 2145616 (1h), CCDC 2145617 (1c), and CCDC 2149555 (1f) contain the supplementary crystallographic data for this paper. These data can be obtained free of charge *via*http://www.ccdc.cam.ac.uk/data_request/cif, or by emailing data_request@ccdc.cam.ac.uk, or by contacting The Cambridge Crystallographic Data Centre, 12 Union Road, Cambridge CB2 1EZ, UK; fax: +44 1223 336033.

## Author contributions

L. K. and B. D. contributed equally to this work. L. K. and M. K. performed the synthetic work and spectroscopic analyses. L. K., B. D., M. S., and L. L. carried out all proteomic experiments and collected the data. D. N. and G. G. performed CIDNP, CV, and EPR experiments and collected the data. A. K. and B. M. provided experimental and conceptual support for the proof-of-concept experiments. R. C. F. carried out the X-ray diffraction analyses. L. K., S. W., and P. M. designed and performed the flavin spectroscopy experiments. L. K. and R. B. wrote the manuscript with input from all authors. R. B.-G. and R. B. conceived and supervised the project.

## Conflicts of interest

There are no conflicts to declare.

## Supplementary Material

SC-016-D4SC08454C-s001

SC-016-D4SC08454C-s002

SC-016-D4SC08454C-s003

SC-016-D4SC08454C-s004
