# Supplementary material for: A general approach for activity-based protein profiling of oxidoreductases with redox-differentiated diarylhalonium warheads
Source: Chem Sci. 2025 Mar 11;16(15):6240–56. doi: 10.1039/d4sc08454c (PMC11912224; doi:10.1039/d4sc08454c)
Supplement: SC-016-D4SC08454C-s001 [file SC-016-D4SC08454C-s001.pdf]

Supplementary Information (SI) for Chemical Science.
This journal is © The Royal Society of Chemistry 2025

Supporting Information

A General Approach for Activity-based Protein Profiling of Oxidoreductases with Redox-differentiated Diarylhalonium Warheads

Leo Krammer,[‡,a] Barbara Darnhofer,[‡,b] Marko Kljajic,[a] Laura Liesinger,[c] Matthias Schittmayer,[c] Dmytro Neshchadin,[d] Georg Gescheidt,[d] Alexander Kollau,[e] Bernd Mayer,[e] Roland C. Fischer,[f] Silvia Wallner,[g] Peter Macheroux,[g] Ruth Birner-Gruenberger,*[,b,c] Rolf Breinbauer*[,a]

[a.] Institute of Organic Chemistry, Graz University of Technology, Stremayrgasse 9, 8010 Graz, Austria.

[b.] Diagnostic and Research Institute of Pathology, Medical University of Graz, Stiftingtalstraße 6, 8036 Graz, Austria.

[c.] Institute of Chemical Technologies and Analytics, Technische Universität Wien, Getreidemarkt 9, 1060 Vienna, Austria.

[d.] Institute of Physical and Theoretical Chemistry, Graz University of Technology, Stremayrgasse 9, 8010 Graz, Austria.

[e.] Institute of Pharmaceutical Sciences, University of Graz, Humboldtstraße 46, 8010 Graz, Austria.

[f.] Institute of Inorganic Chemistry, Graz University of Technology, Stremayrgasse 9, 8010 Graz, Austria.

[g.] Institute of Biochemistry, Graz University of Technology, Petersgasse 12, 8010 Graz, Austria.

‡ These authors contributed equally.

*Correspondence to: ruth.birner-gruenberger@tuwien.ac.at and breinbauer@tugraz.at

Table of Contents

Supplementary Figures, Schemes and Tables 5

General Information 50

Dry Solvents 50
High Performance Liquid Chromatography with Mass Spectrometry (HPLC-MS) 51
Gas Chromatography with Mass Spectrometry (GC-MS) 51
Nuclear Magnetic Resonance Spectroscopy 52
High Resolution Mass Spectrometry (HRMS) 52
Determination of Melting Points 53
Thin Layer Chromatography 53
Flash Column Chromatography 53
X-ray Diffraction 53

Experimental Procedures 55

Overview 55
Synthesis of Azidoamine Linkers 60
1,10-Diazidodecane (**15**) 60
10-Azidodecan-1-amine (**2a**) 60
Tetraethylene glycol di(*p*-toluenesulfonate) (**16**) 61
1-Azido-2-(2-(2-(2-azidoethoxy)ethoxy)ethoxy)ethane (**17**) 62
2-(2-(2-(2-Azidoethoxy)ethoxy)ethoxy)ethan-1-amine (**2b**) 63
Synthesis of Biphenylcarboxylic Acids 63
General Procedure for the 3-Iodination of 4-Halobenzoic acids 63
4-Bromo-3-iodobenzoic acid (**18**) 64
4-Chloro-3-iodobenzoic acid (**19**) 64
Methyl 4-bromo-3-iodobenzoate (**20**) 65
Methyl 4-chloro-3-iodobenzoate (**21**) 66
Methyl 3-bromo-4-iodobenzoate (**22**) 67
Methyl 3-chloro-4-iodobenzoate (**23**) 67
2'-Iodo-[1,1'-biphenyl]-4-carboxylic acid (**5**) 68
2'-Iodo-[1,1'-biphenyl]-3-carboxylic acid (**6**) 69
6-Amino-[1,1'-biphenyl]-3-carboxylic acid (**24**) 70
6-Iodo-[1,1'-biphenyl]-3-carboxylic acid (**4**) 71
Methyl 2'-amino-6-bromo-[1,1'-biphenyl]-3-carboxylate (**25**) 72
Methyl 2'-amino-2-bromo-[1,1'-biphenyl]-4-carboxylate (**26**) 73
2'-Amino-2-bromo-[1,1'-biphenyl]-4-carboxylic acid (**8**) 75
Methyl 2'-amino-6-chloro-[1,1'-biphenyl]-3-carboxylate (**27**) 76

Methyl 2'-amino-2-chloro-[1,1'-biphenyl]-4-carboxylate (**28**) 77
2'-Amino-6-chloro-[1,1'-biphenyl]-3-carboxylic acid (**9**) 78
2'-Amino-2-chloro-[1,1'-biphenyl]-4-carboxylic acid (**10**) 79
Synthesis of Diarylhalonium Salts 80
(4-Carboxyphenyl)(phenyl)iodonium trifluoromethanesulfonate (**1a**) 80
(3-Carboxyphenyl)(phenyl)iodonium trifluoromethanesulfonate (1**b**) 81
2-Carboxydibenzo[*b*,*d*]iodol-5-ium trifluoromethanesulfonate (**1c**) 81
3-Carboxydibenzo[*b*,*d*]iodol-5-ium trifluoromethanesulfonate (**1d**) 82
4-Carboxydibenzo[*b*,*d*]iodol-5-ium trifluoromethanesulfonate (**1e**) 83
2-Carboxydibenzo[*b*,*d*]bromol-5-ium chloride (**11**) 84
2-Carboxydibenzo[*b*,*d*]bromol-5-ium trifluoromethanesulfonate (**1f**) 85
3-Carboxydibenzo[*b*,*d*]bromol-5-ium chloride (**12**) 87
3-Carboxydibenzo[*b*,*d*]bromol-5-ium trifluoromethanesulfonate (**1g**) 88
2-Carboxydibenzo[*b*,*d*]chlorol-5-ium chloride (**13**) 89
2-Carboxydibenzo[*b*,*d*]chlorol-5-ium trifluoromethanesulfonate (**1h**) 89
3-Carboxydibenzo[*b*,*d*]chlorol-5-ium chloride (**14**) 90
3-Carboxydibenzo[*b*,*d*]chlorol-5-ium trifluoromethanesulfonate (**1i**) 91
Synthesis of Diarylhalonium ABPP probes 92
General Procedure A for the Synthesis of Iodonium- and Bromonium-based Probes via Acyl chloride 92
General Procedure B for the Synthesis of Chloronium-based Probes via Carbodiimide-mediated Amidation 92
(4-((10-Azidodecyl)carbamoyl)phenyl)(phenyl)iodonium trifluoromethanesulfonate (**3aa**) 93
(4-((2-(2-(2-(2-Azidoethoxy)ethoxy)ethoxy)ethyl)carbamoyl)phenyl)(phenyl)iodonium trifluoromethanesulfonate (**3ab**) 94
(3-((10-Azidodecyl)carbamoyl)phenyl)(phenyl)iodonium trifluoromethanesulfonate (**3ba**) 95
(3-((2-(2-(2-(2-Azidoethoxy)ethoxy)ethoxy)ethyl)carbamoyl)phenyl)(phenyl)iodonium trifluoromethanesulfonate (**3bb**) 96
2-((10-Azidodecyl)carbamoyl)dibenzo[*b*,*d*]iodol-5-ium trifluoromethanesulfonate (**3ca**) 97
2-((2-(2-(2-(2-Azidoethoxy)ethoxy)ethoxy)ethyl)carbamoyl)dibenzo[*b*,*d*]iodol-5-ium trifluoromethanesulfonate (**3cb**) 98
3-((10-Azidodecyl)carbamoyl)dibenzo[*b*,*d*]iodol-5-ium trifluoromethanesulfonate (**3da**) 99
3-((2-(2-(2-(2-Azidoethoxy)ethoxy)ethoxy)ethyl)carbamoyl)dibenzo[*b*,*d*]iodol-5-ium trifluoromethanesulfonate (**3db**) 100
4-((10-Azidodecyl)carbamoyl)dibenzo[*b*,*d*]iodol-5-ium trifluoromethanesulfonate (**3ea**) 101
4-((2-(2-(2-(2-Azidoethoxy)ethoxy)ethoxy)ethyl)carbamoyl)dibenzo[*b*,*d*]iodol-5-ium trifluoromethanesulfonate (**3eb**) 102
2-((10-Azidodecyl)carbamoyl)dibenzo[*b*,*d*]bromol-5-ium trifluoromethanesulfonate (**3fa**) 103
2-((2-(2-(2-(2-Azidoethoxy)ethoxy)ethoxy)ethyl)carbamoyl)dibenzo[*b*,*d*]bromol-5-ium trifluoromethanesulfonate (**3fb**) 104

3-((10-Azidodecyl)carbamoyl)dibenzo[*b*,*d*]bromol-5-ium trifluoromethanesulfonate (**3ga**) 105

3-((2-(2-(2-(2-Azidoethoxy)ethoxy)ethoxy)ethyl)carbamoyl)dibenzo[*b*,*d*]bromol-5-ium trifluoromethanesulfonate (**3gb**) 106

2-((10-Azidodecyl)carbamoyl)dibenzo[*b*,*d*]chlorol-5-ium trifluoromethanesulfonate (**3ha**) 107

3-((10-Azidodecyl)carbamoyl)dibenzo[*b*,*d*]chlorol-5-ium trifluoromethanesulfonate (**3ia**) 108

Synthesis of Methyl *t*-butacrylate (*t*-BAM) 110

Methyl 3,3-dimethyl-2-oxobutanoate (**29**) 110

Methyl 3,3-dimethyl-2-methylenebutanoate (*t*-BAM) (**30**) 111

Mechanistic Experiments 112

Cyclic Voltammetry 112

Spin-trapping EPR 112

^{1}H NMR and CIDNP 112

HPLC-MS Analysis of *t*-BAM Experiments 112

UV-Vis Spectrophotometric Experiments 113

Biochemical Experiments 114

Recombinant Enzyme Experiments 114

Expression of NQO1 and Corresponding Amino Acid Sequence 115

In vitro Labeling Experiments of Mouse Liver Tissues 115

Activity-based Gels 116

Protein Enrichment Experiments 116

LC-MS/MS Analysis and Bioinformatics for Protein Enrichment Experiments 117

Probes Site Analysis 118

LC-MS/MS Analysis and Bioinformatics for Probed Site Analysis 118

References 121

Crystallographic Data 123

NMR Appendix 124

Supplementary Figures, Schemes and Tables

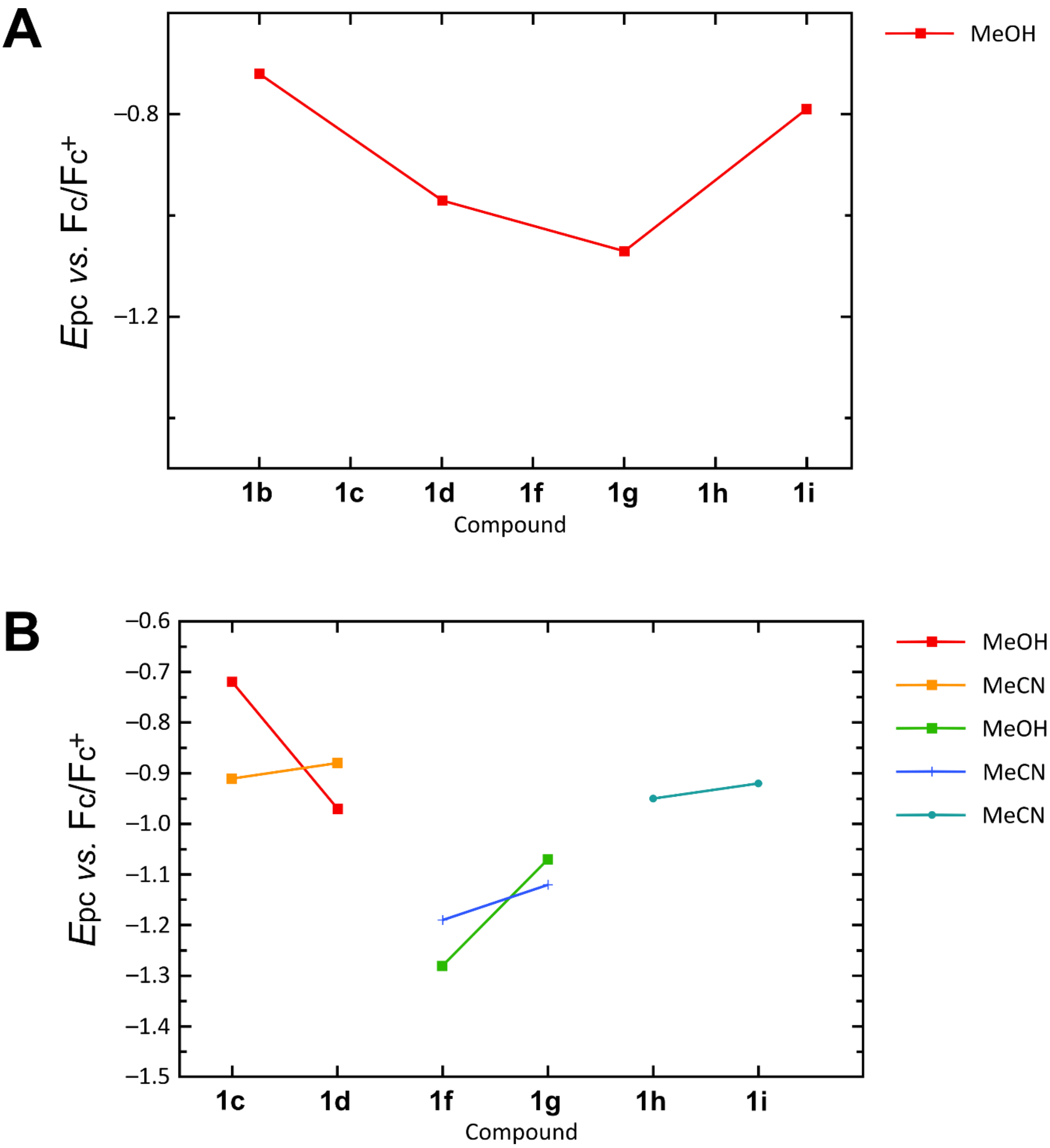

Figure S1. (A) Dependence of the E_{pc} (in MeOH) on the halonium cation in *meta*-substituted compounds **1b**, **1d**, **1g**, and **1i**. (B): Dependence of the E_{pc} on the substitution pattern (*meta* vs. *para*) and solvent (MeOH vs. MeCN). The differences are based on the following factors: i) The electronic effect of the carboxylic group; going from *meta* to *para* substitution the E_{pc} is shifted by ca. 200 mV in MeOH (**1d** → **1c**, **1g** → **1f**). In MeCN, these shifts are less pronounced. ii) Ion pairing/solvation; this is the ruling factor for the different E_{pc} values. In going from MeOH to MeCN, the E_{pc} values alter by ca. 100 mV, however this change is not uniform in going from I^+ to Br^+ to Cl^+. This can be traced back to the differing solvation/character of the ion pairs [(diaryl halonium)$^+$ OTf^-].[1]

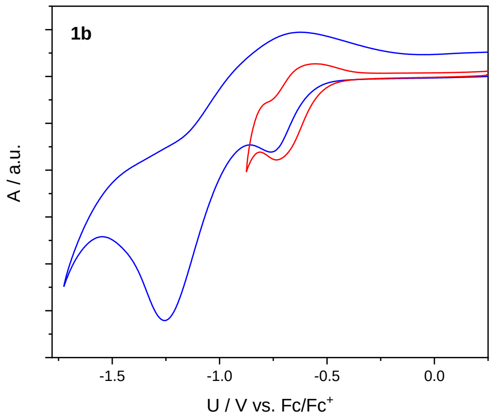

Figure S2. Cyclovoltammogram of **1b** (blue curve, 30 mM concentration, solvent: MeCN, scan rate: 500 mVs^{-1}, supporting salt: Bu_4NClO_4 (0.1 M), working electrode: Pt). The red curves indicate the decrease of the reduction peak upon addition of NADH.

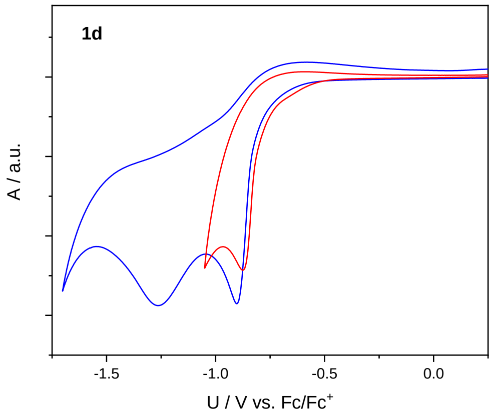

Figure S3. Cyclovoltammogram of **1d** (blue curve, 30 mM concentration, solvent: MeCN, scan rate: 500 mVs^{-1}, supporting salt: Bu_4NClO_4 (0.1 M), working electrode: Pt). The red curves indicate the decrease of the reduction peak upon addition of NADH.

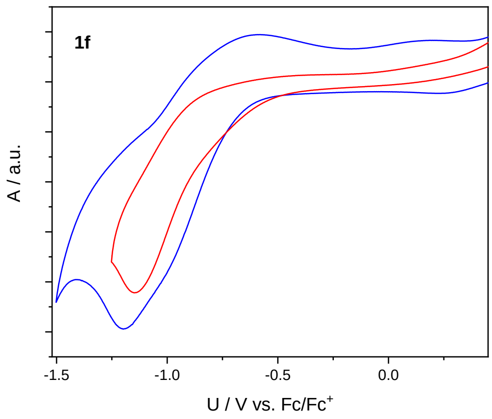

Figure S4. Cyclovoltammogram of **1f** (blue curve, 30 mM concentration, solvent: MeCN, scan rate: 500 mVs^{-1}, supporting salt: Bu_4NClO_4 (0.1 M), working electrode: Pt). The red curves indicate the decrease of the reduction peak upon addition of NADH.

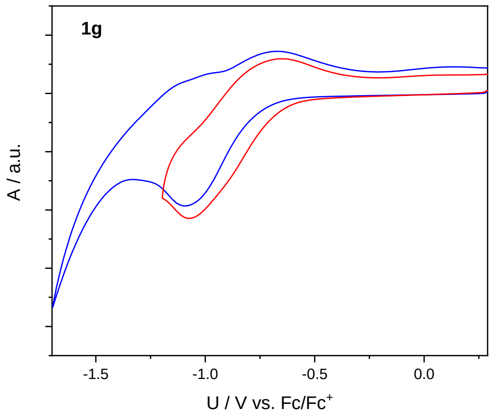

Figure S5. Cyclovoltammogram of **1g** (blue curve, 30 mM concentration, solvent: MeCN, scan rate: 500 mVs^{-1}, supporting salt: Bu_4NClO_4 (0.1 M), working electrode: Pt). The red curves indicate the decrease of the reduction peak upon addition of NADH.

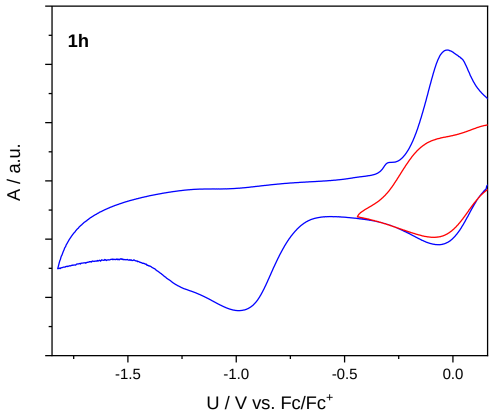

Figure S6. Cyclovoltammogram of **1h** (blue curve, 30 mM concentration, solvent: MeCN, scan rate: 500 mVs^{-1}, supporting salt: Bu_4NClO_4 (0.1 M), working electrode: Pt). The red curves indicate the decrease of the reduction peak upon addition of NADH.

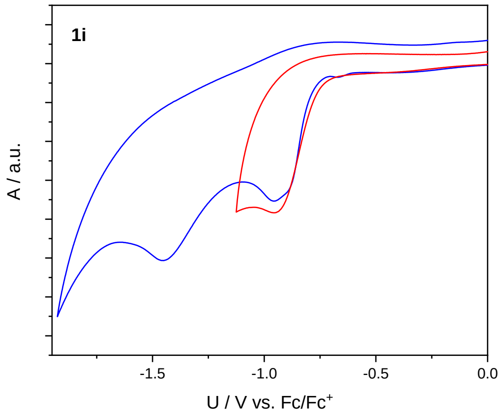

Figure S7. Cyclovoltammogram of **1i** (blue curve, 30 mM concentration, solvent: MeCN, scan rate: 500 mVs^{-1}, supporting salt: Bu_4NClO_4 (0.1 M), working electrode: Pt). The red curves indicate the decrease of the reduction peak upon addition of NADH.

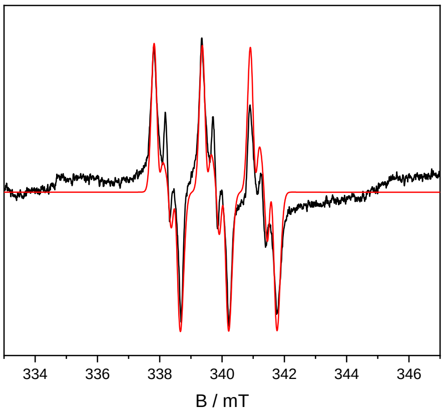

Figure S8. CW-EPR during the reaction of **1g**, **NADH**, and **PBN** overlaid with the simulated spectrum.

Parameters of simulation:

Radical 1: ^{14}N hfc = 15.5, ^{1}H hfc = 4.1

Radical 2: ^{14}N hfc = 15.4, ^{1}H hfc = 4.1

Ratio 41/59

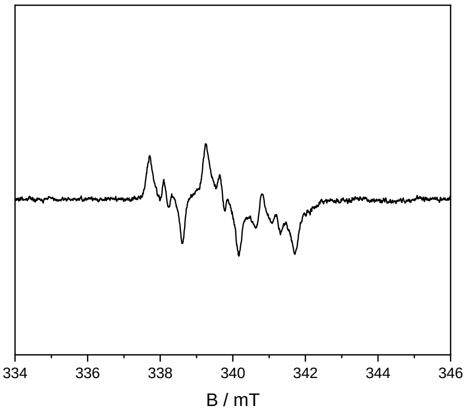

Figure S9. CW-EPR during the reaction of **1h**, NADH, and PBN.

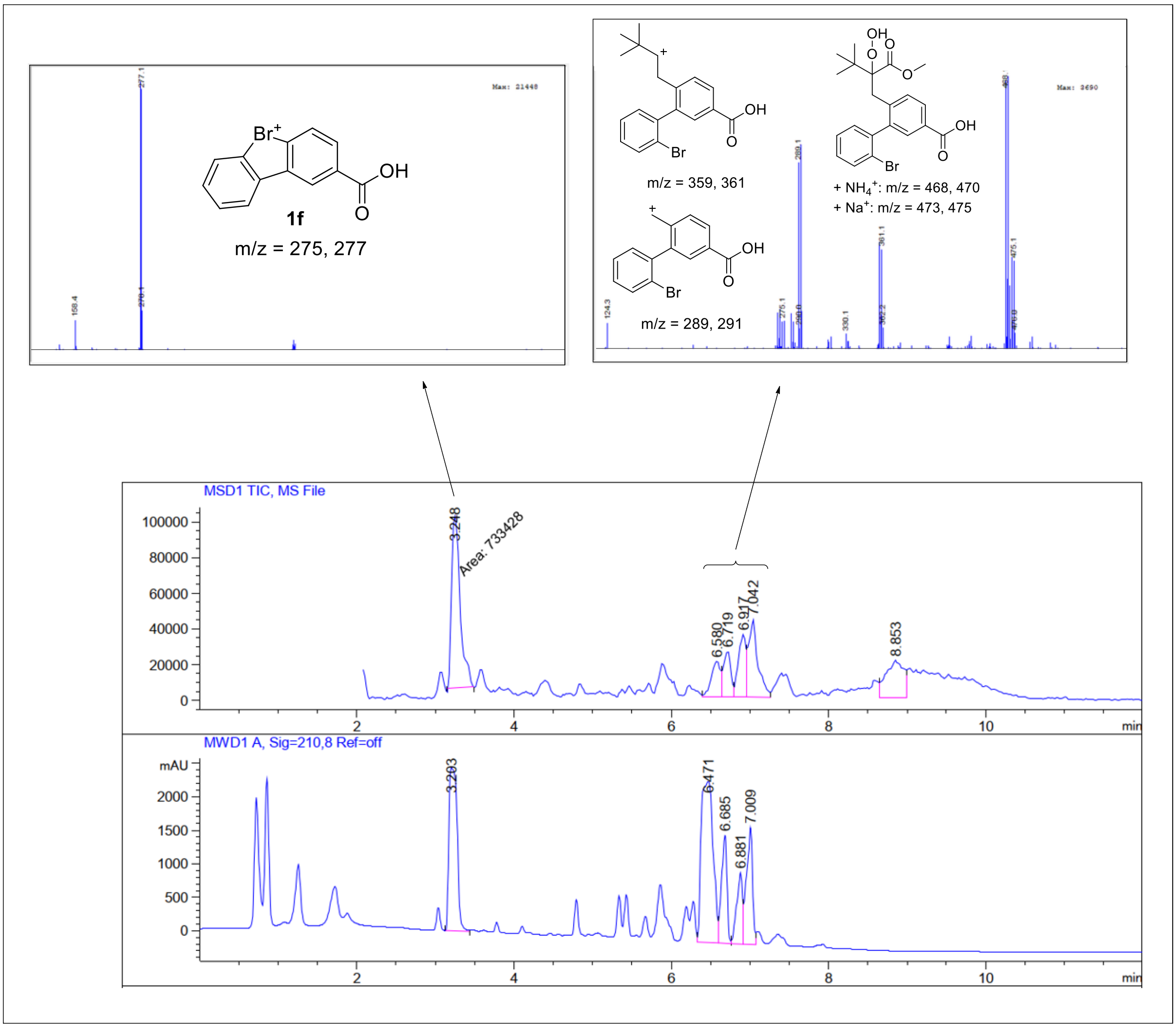

Figure S10. HPLC-Chromatogram and mass spectra of selected peaks of redox reaction with NADH and diarylbromonium salt **1f** using *t*-BAM (**30**) as a non-polymerizable monomer. Intact diarylbromonium salt (top left) as well as hydroperoxide-adduct/fragmentation products of *t*-BAM adducts (top right) could be detected. Please note that the modifications could also occur on the bromophenyl ring.

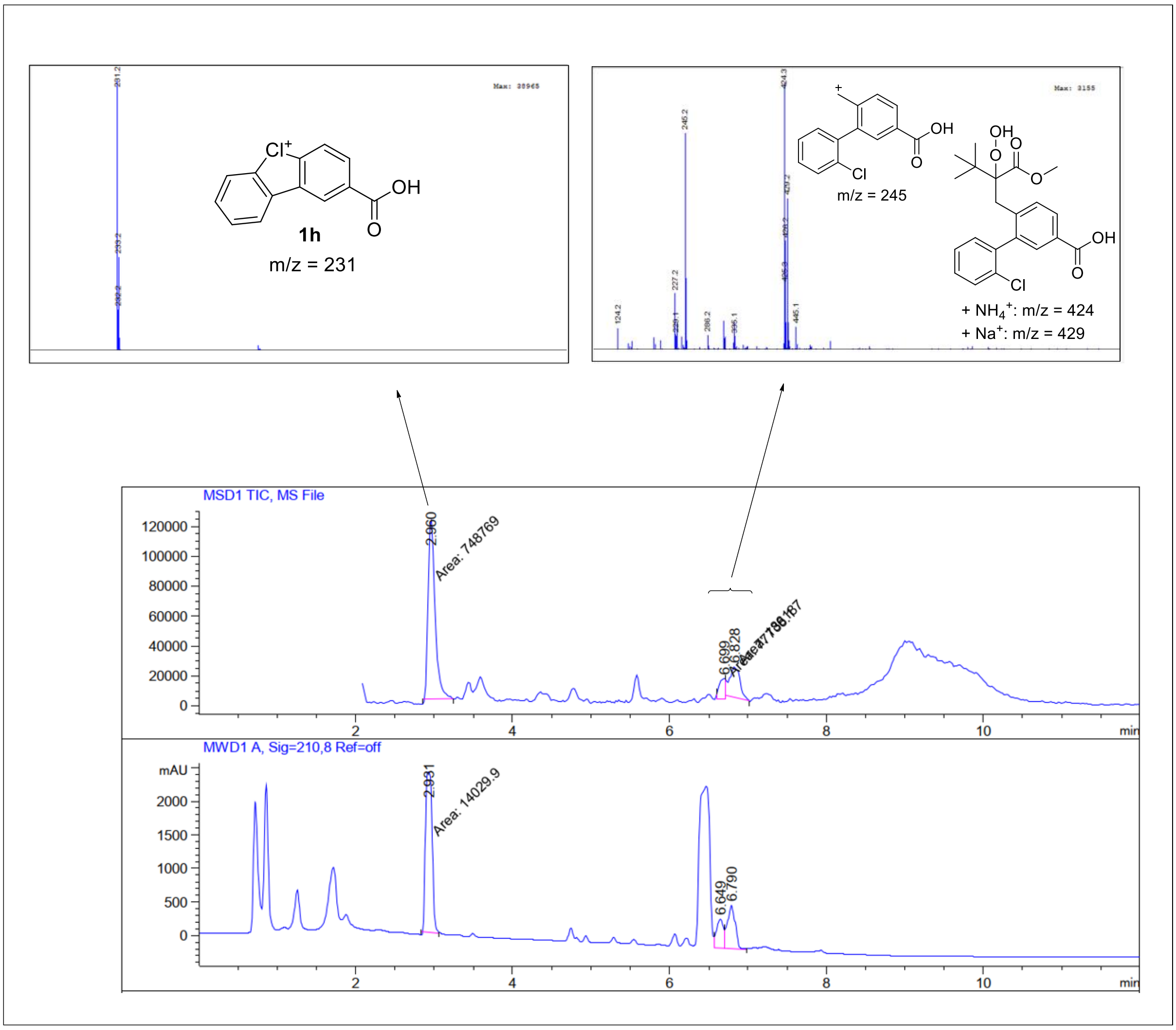

Figure S11. HPLC-Chromatogram and mass spectra of selected peaks of redox reaction with NADH and diarylchloronium salt **1h** using t-BAM (**30**) as a non-polymerizable monomer. Intact diarylchloronium salt (top left) as well as hydroperoxide-adduct/fragmentation products of t-BAM adducts (top right) could be detected. Please note that the modifications could also occur on the chlorophenyl ring.

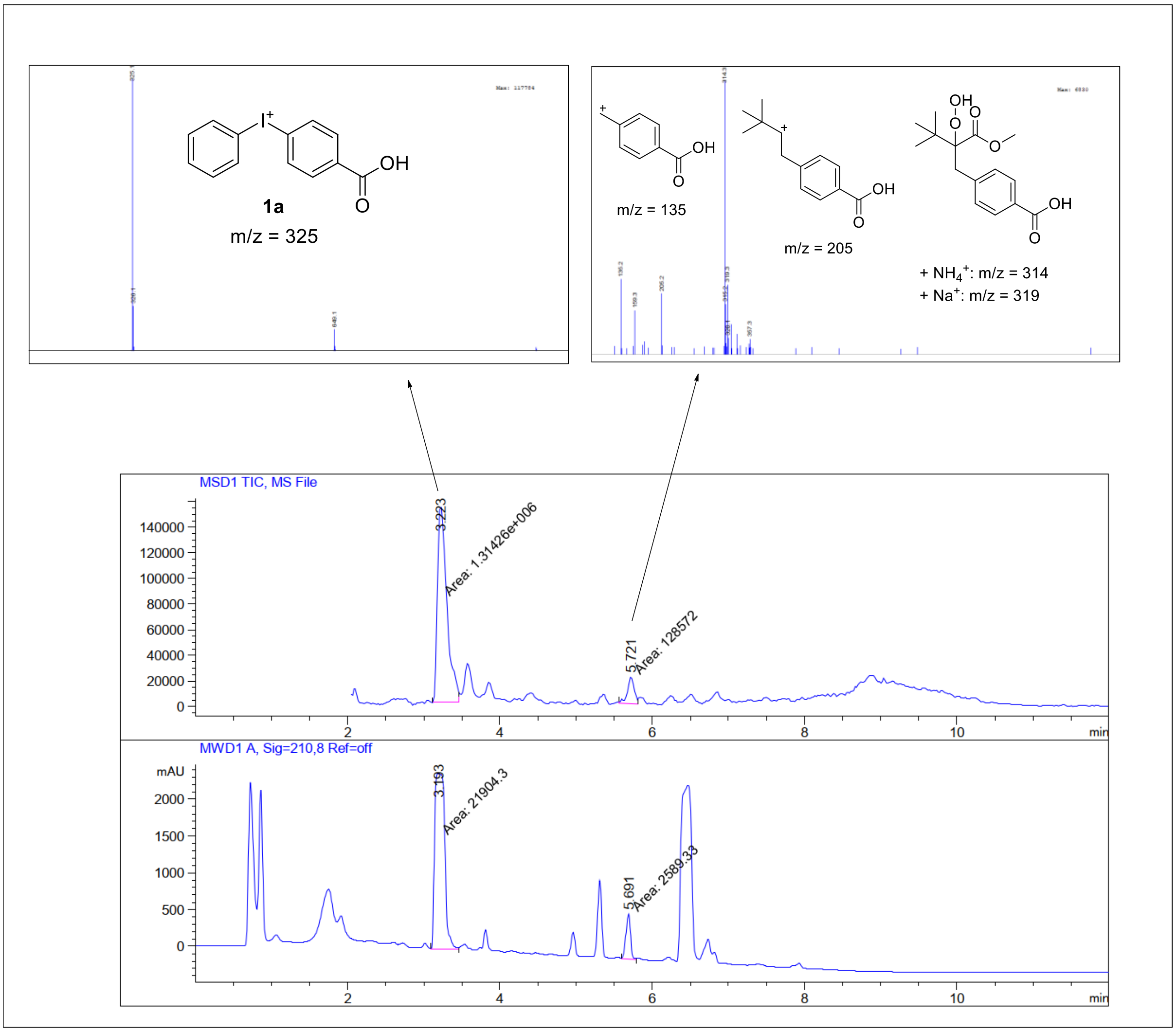

Figure S12. HPLC-Chromatogram and mass spectra of selected peaks of redox reaction with NADH and diaryliodonium salt **1a** using t-BAM (**30**) as a non-polymerizable monomer. Intact diaryliodonium salt (top left) as well as hydroperoxide-adduct/fragmentation products of t-BAM adducts (top right) could be detected. Please note that the modifications could also occur on the phenyl ring.

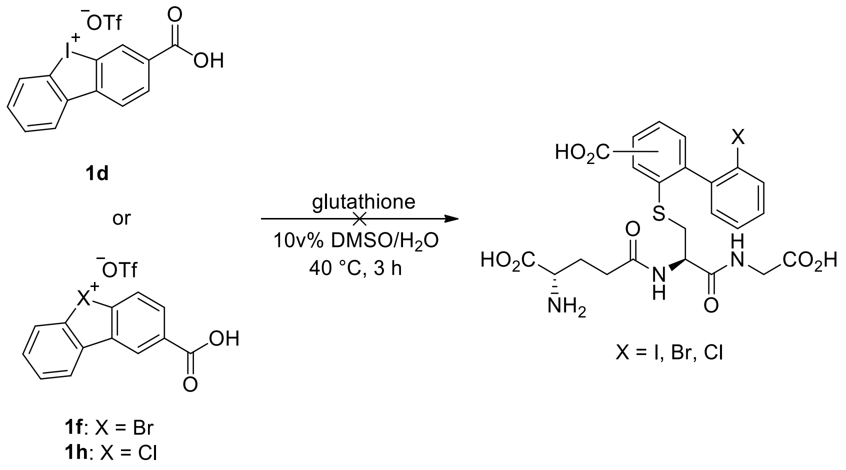

Figure S13. Control experiment with selected diphenylenehalonium salts and glutathione. Glutathione (11.1 mM in H_2O, 450 µL, 5.0 µmol, 1.0 eq.) was warmed to 40 °C in HPLC-Vial, halonium salt **1d**/**1f**/**1h** (120 mM in DMSO, 50 µL, 6.0 µmol, 1.2 eq.) was added at 40 °C and the mixture stirred at 40 °C for 3 h. Samples were analyzed by HPLC-MS.

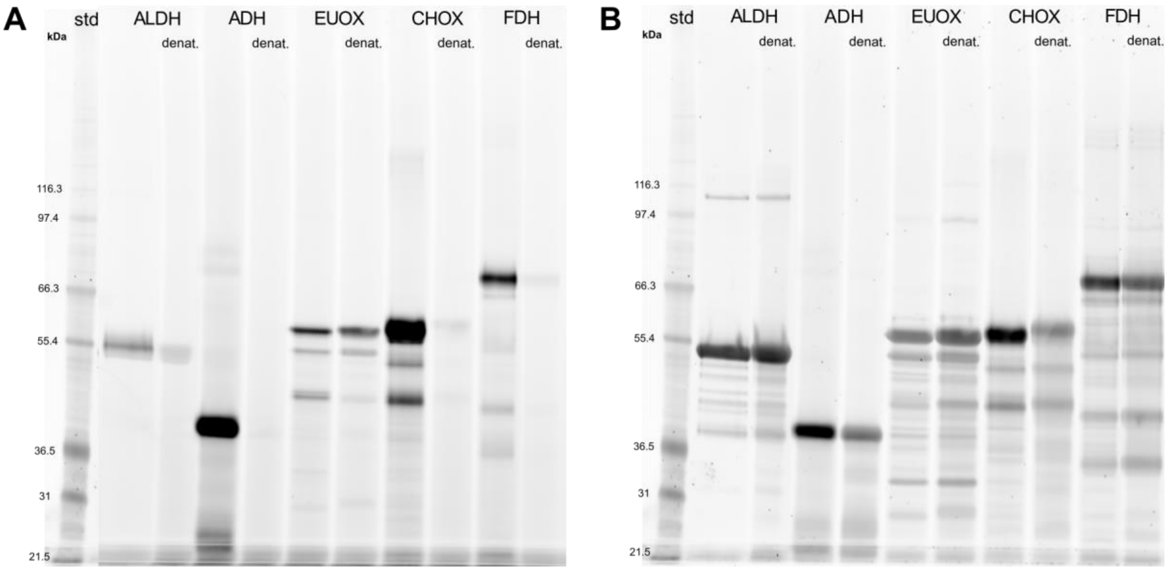

Figure S14. (A) Fluorescent SDS-PAGE analysis of the labeling of different recombinant enzymes with probe **3aa** (30 µM, 1 h) without/with prior denaturation (= denat.) with heat + 1% SDS. Protein bands were visualized by fluorescent staining. (B) Total protein stain of activity-based gel. Proteins were visualized with Krypton fluorescent protein stain. Mark 12™ Unstained Standard (Thermo Fisher Scientific) was used as protein standard (= std). ALDH = aldehyde dehydrogenase 2 (recombinant human), ADH = alcohol dehydrogenase (yeast), EUOX = eugenoloxidase (*Rhodococcus jostii*), CHOX = choline oxidase (*Arthrobacter nicotianae*), FDH = formate dehydrogenase (*Clostridium carboxidivorans*). Mark 12™ Unstained Standard (Thermo Fisher Scientific) was used as protein standard (= std).

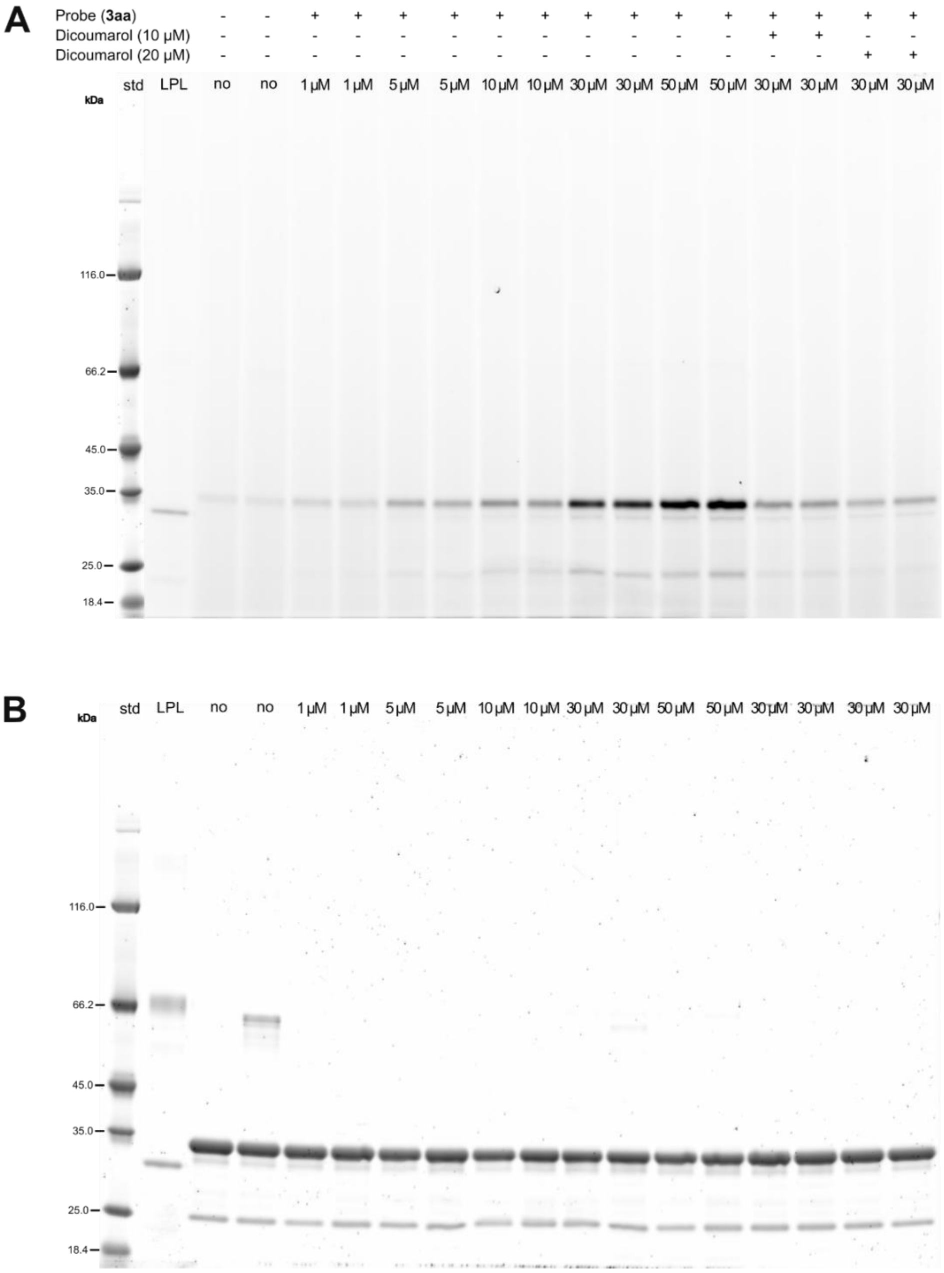

Figure S15. (A) Fluorescent SDS-PAGE analysis of the labeling of NQO1 with probe **3aa (**1 – 50 µM, 1 h; no = no probe added) with NADH and with or without inhibitor (dicoumarol, 10 or 20 µM, 15 min) added. All experiments were performed in duplicates. Protein bands were visualized by fluorescent staining. Lipoprotein lipase (LPL) was used as a positive control. (B) Total protein stain of activity-based gel. Proteins were visualized with Krypton fluorescent protein stain. Pierce™ Unstained Protein MW Marker (Thermo Fisher Scientific) was used as protein standard (= std).

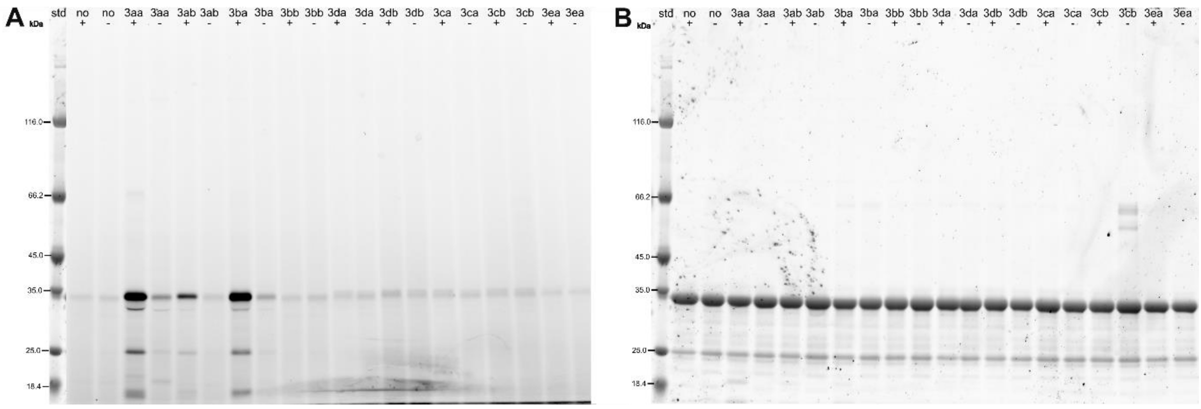

Figure S16. (A) Fluorescent SDS-PAGE analysis of the labeling of NQO1 with probes **3aa**-**3ea** (40 µM, 1 h; no = no probe added) with (+) or without (–) NADH added. Protein bands were visualized by fluorescent staining. (B) Total protein stain of activity-based gel. Proteins were visualized with Krypton fluorescent protein stain. Pierce™ Unstained Protein MW Marker (Thermo Fisher Scientific) was used as protein standard (= std).

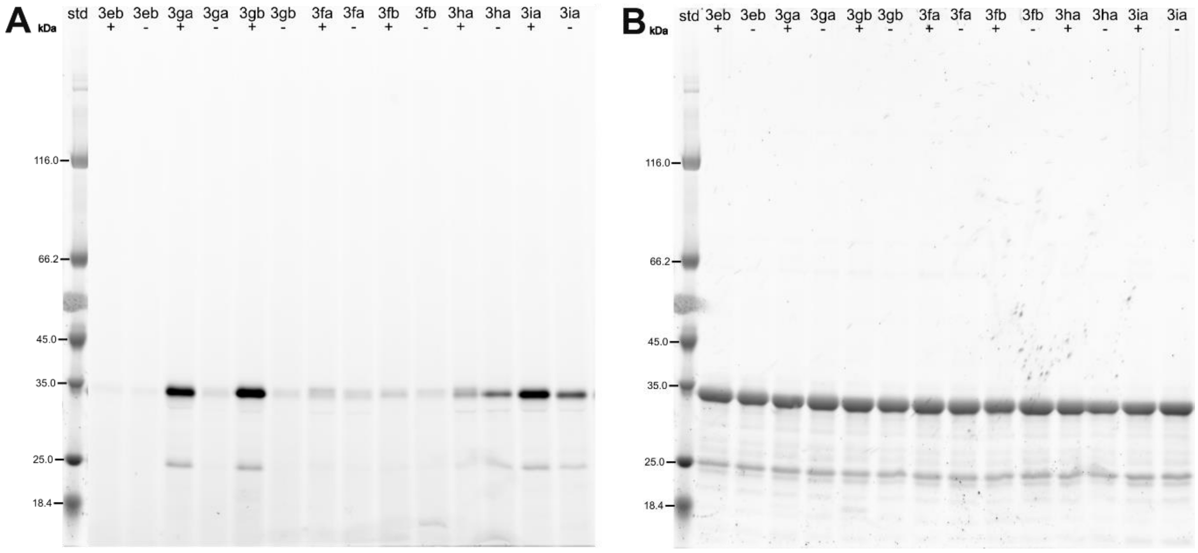

Figure S17. (A) Fluorescent SDS-PAGE analysis of the labeling of NQO1 with probes **3eb**-**3ia (**40 µM, 1 h) with (+) or without (–) NADH added. Protein bands were visualized by fluorescent staining. (B) Total protein stain of activity-based gel. Proteins were visualized with Krypton fluorescent protein stain. Pierce™ Unstained Protein MW Marker (Thermo Fisher Scientific) was used as protein standard (= std).

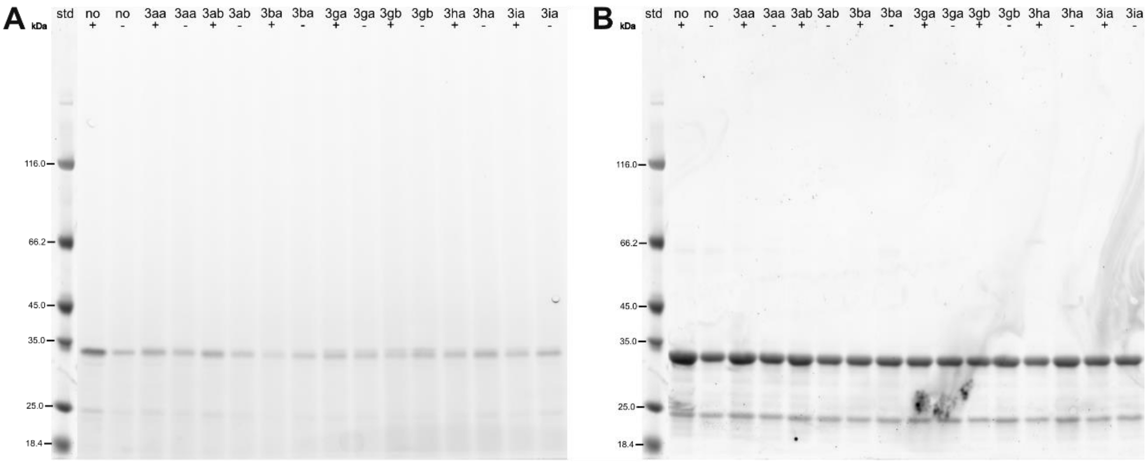

Figure S18. (A) Fluorescent SDS-PAGE analysis of the labeling of denatured (2% SDS + heat) NQO1 with selected probes (40 µM, 1 h; no = no probe added) with (+) or without (–) NADH added. Protein bands were visualized by fluorescent staining. (B) Total protein stain of activity-based gel. Proteins were visualized with Krypton fluorescent protein stain. Pierce™ Unstained Protein MW Marker (Thermo Fisher Scientific) was used as protein standard (= std).

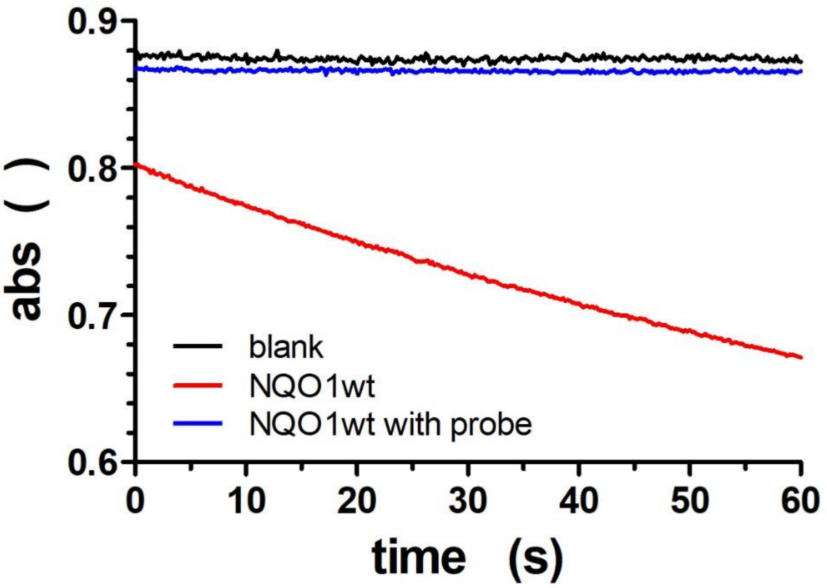

Figure S19. Enzymatic activity of NQO1wt with and without iodonium probe **3aa**. NQO1wt (10 nM) was mixed with 200 µM NADH and 200 µM menadione as substrate and the consumption of NADH was followed at 365 nm as an indicator for enzymatic activity. The red solid line shows the consumption of NADH in the activity assay performed with NQO1wt, the blue and black solid lines were measured when NQO1wt was pretreated with iodonium probe **3aa** and when no enzyme was added to the reaction mixture, respectively. The experiments were performed in triplicates (mean values are shown).

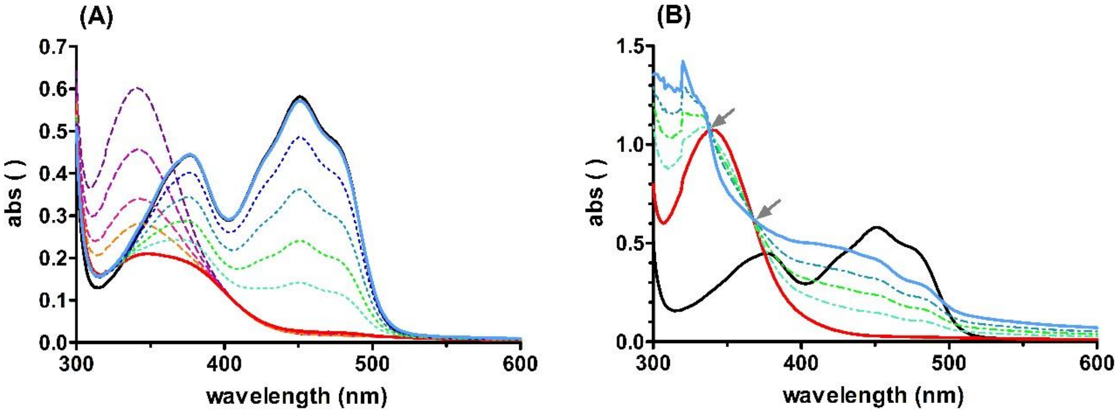

Figure S20. (A) Reduction und reoxidation of NQO1 with NADH and oxygen. UV-vis absorption spectra were recorded during the reduction of 40 µM NQO1 in 50 mM HEPES, pH 7.0 with a 4-fold excess of NADH. The solid black line shows the spectrum of the oxidized flavin cofactor of NQO1 with the two characteristic absorption maxima at 375 and 450 nm. The dashed colored lines (magenta to orange) were recorded immediately after addition of 160 µM NADH and after 3, 6, and 8 min and show the reduced flavin cofactor (complete loss of absorption at 450 nm) and a characteristic peak of NADH at 340 nm. The solid red line shows the reduced flavin cofactor of NQO1 10 min after the addition of NADH, when almost all reducing agent was used up. The dotted lines (cyan to dark blue) represent the spectra during reoxidation of the flavin cofactor with oxygen and the final spectrum of the reoxidized flavin is shown as a solid light blue line. (B) Reaction of reduced NQO1 with iodonium probe **3aa**. The solid black line represents the spectrum of 40 µM NQO1 in 50 mM HEPES, pH 7.0. The solid red line was recorded 2 min after the addition of 160 µM NADH and prior to the addition of 400 µM **3aa**. The dashed-dotted lines show selected spectra during the reaction of reduced NQO1 with **3aa**. The solid light blue line corresponds to the final spectrum after reaction of NQO1 with **3aa**. Note that the reaction of the reduced flavin with **3aa** proceeds with two isosbestic points at 338 and 368 nm (grey arrows).

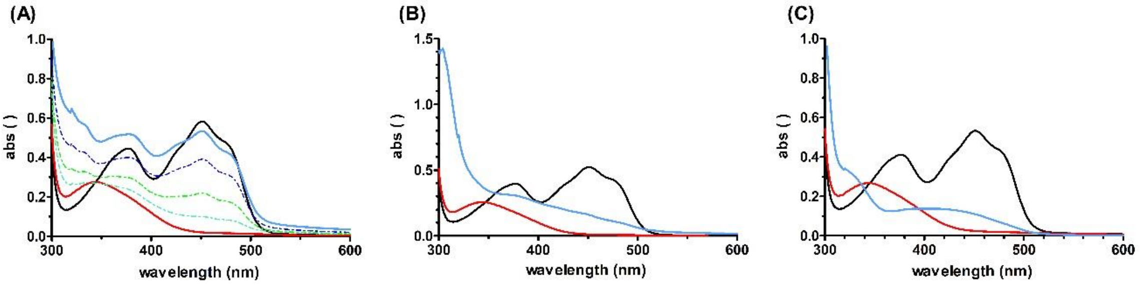

Figure S21. (A) Reaction of reduced NQO1 with iodonium probe **3aa**. The solid black line represents the spectrum of 40 µM NQO1 in 50 mM HEPES, pH 7.0. The solid red line was recorded 2 min after the addition of 80 µM NADH and prior to the addition of 400 µM **3aa**. The lines in dash-dotted style show selected spectra during the reaction of reduced NQO1 with **3aa**, respectively. The solid light blue line corresponds to the final spectrum after reaction of NQO1 with **3aa**. (B) Reaction of reduced NQO1 with bromonium probe **3ga**. The solid black line represents the spectrum of 40 µM NQO1 in 50 mM HEPES, pH 7.0. The solid red line was recorded 2 min after the addition of 80 µM NADH and prior to the addition of 400 µM **3ga**. The final spectrum after the fast reaction of NQO1 with **3ga** is drawn as solid light blue line. (C) Reaction of reduced NQO1 with chloronium probe **3ha**. The solid black line represents the spectrum of 40 µM NQO1 in 50 mM HEPES, pH 7.0. The solid red line was recorded 2 min after the addition of 80 µM NADH and prior to the addition of 400 µM **3ha**. The final spectrum after the fast reaction of NQO1 with **3ha** is drawn as solid light blue line.

Acquisition of spectra prior to and after addition of NADH show the spectral changes induced by the reduction of the flavin. Without the addition of probe, the flavin will undergo complete reoxidation to its initial state under aerobic conditions (Figure S20A). However, upon addition of probes **3aa**, **3ga**, and **3ha** after complete reduction of NQO1 with 2 eq. NADH, the flavin is "trapped" in its reductive state and reoxidation to the initial state is suppressed (Figure S21). The reactions of bromonium probe **3ga** and chloronium probe **3ha** with reduced NQO1 proceeded very fast. Almost no competing reoxidation

by airborne oxygen could be observed, as the characteristic flavin absorption maxima at 375 and 450 nm (Figure S21B-C, solid black lines), respectively, were not visible at all after the reactions (Figure S21B-C, solid light blue lines). Iodonium probe **3aa**, on the other hand, reacted more slowly with reduced NQO1. The final spectrum recorded upon complete reaction after preliminary reduction of NQO1 with 2 eq. NADH and addition of 10 eq. **3aa** (Figure S21A, solid light blue line), as well as some spectra recorded during the reaction (Figure S21A, dash-dotted lines), feature the characteristic flavin absorption maxima, indicating that NQO1 had already been partially reoxidized by oxygen before it was able to react with iodonium probe **3aa**. However, the discrepancy between the initial (Figure S21A, solid black line) and final spectrum (Figure S21A, solid light blue line) can unequivocally be attributed to a reaction between the flavin of NQO1 and **3aa**. Upon adding more reducing agent (4 eq. NADH instead of 2 eq.) prior to the addition of iodonium probe **3aa**, a significant change could be observed (Figure S20B). As more reduced NQO1 was available over the course of the reaction, due to a constant further reduction after reoxidation by airborne oxygen, more probe was able to react with and label the flavin cofactor of NQO1, resulting in a final spectrum with less prominent flavin peaks and more similar to the ones acquired from the reaction with bromonium and chloronium probes **3ga** and **3ha** (Figure S21B-C, solid light blue lines). Interestingly, two clear isosbestic points appeared during this reaction (Figure S20B), indicating that the reaction proceeds *via* a single species, which is from reduced flavin to the flavin-probe adduct. Isosbestic points were not visible in the reactions with 2 eq. NADH (Figure S21A-C) because in the reaction with iodonium probe **3aa** the reduced flavin of NQO1 formed two different products (i.e. reoxidized flavin and flavin-probe adduct) and the reactions with the bromonium and chloronium probe proceeded merely too fast. Notably, without preceding reduction by NADH, addition of the probes did not have significant influence on the flavin spectra. This observation once again substantiates our "activation by enzymatic reduction" proposal. As already proposed by Tew[2] and Massey et al.,[3] flavin-halonium adduct formation can occur either via nucleophilic attack of the flavin anion or via radical intermediates. The latter mechanism, however, has not been found throughout all flavoenzymes tested.[3]

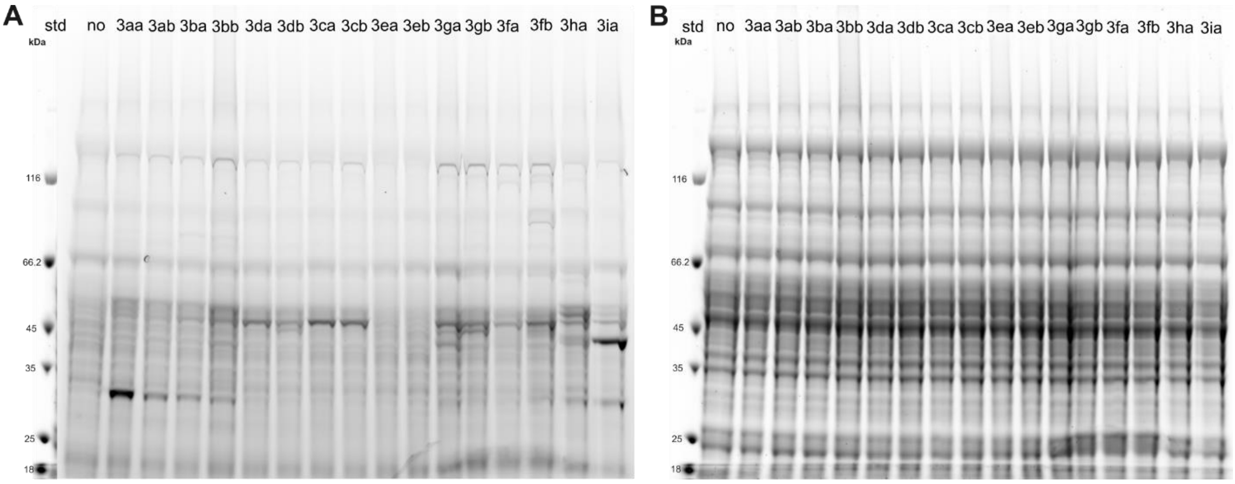

Figure S22. (A) Fluorescent SDS-PAGE analysis of the labeling of fresh mouse liver pieces with probes **3aa**-**3ia** (30 µM, 1 h; no = no probe added). Protein bands were visualized by fluorescent staining. (B) Total protein stain of activity-based gel. Proteins were visualized with Krypton fluorescent protein stain. Pierce™ Unstained Protein MW Marker (Thermo Fisher Scientific) was used as protein standard (= std).

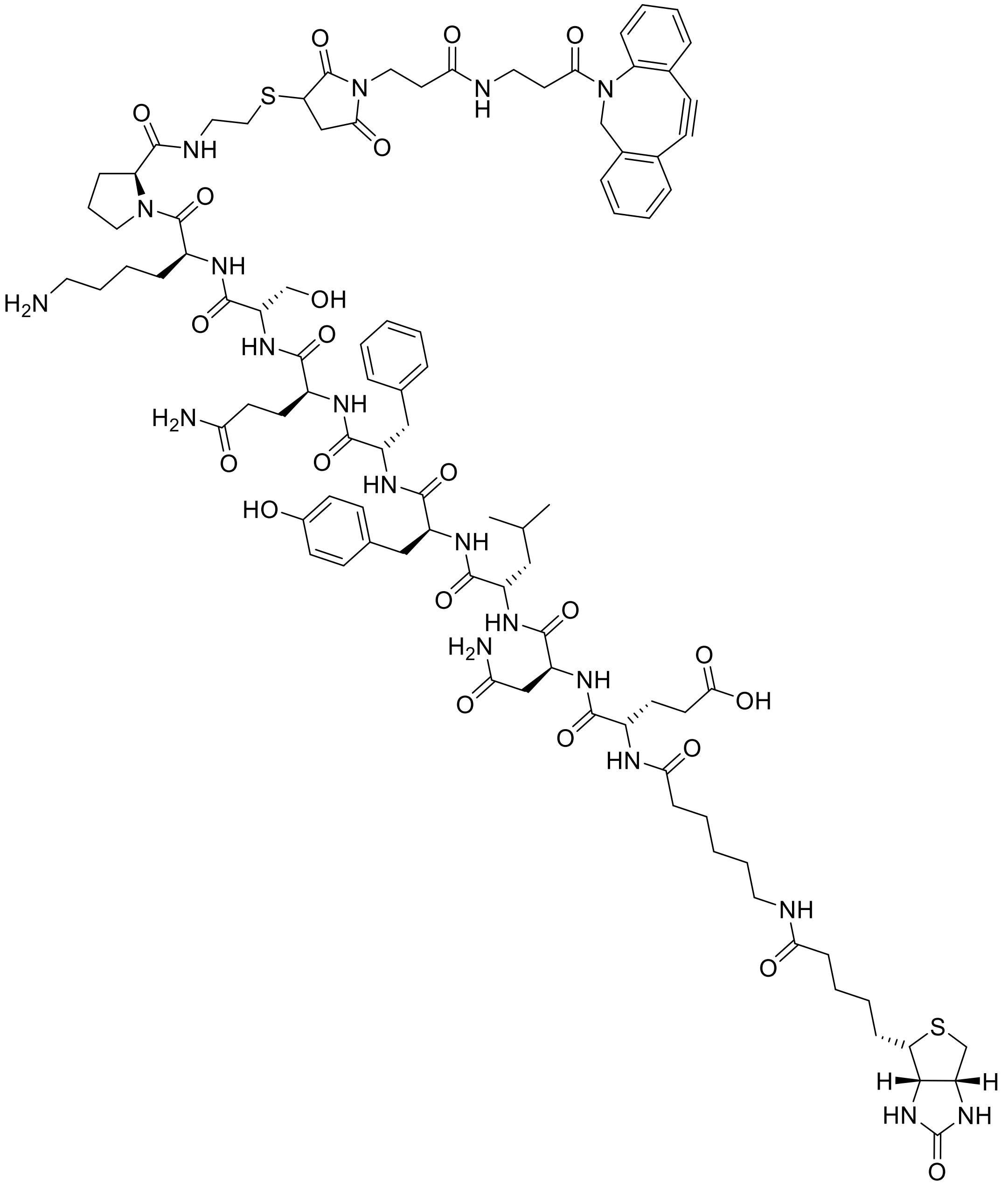

Figure S23. Structure of dibenzocyclooctyne (DBCO)-TEV-Biotin strain-promoted click linker (PiChem, Austria).

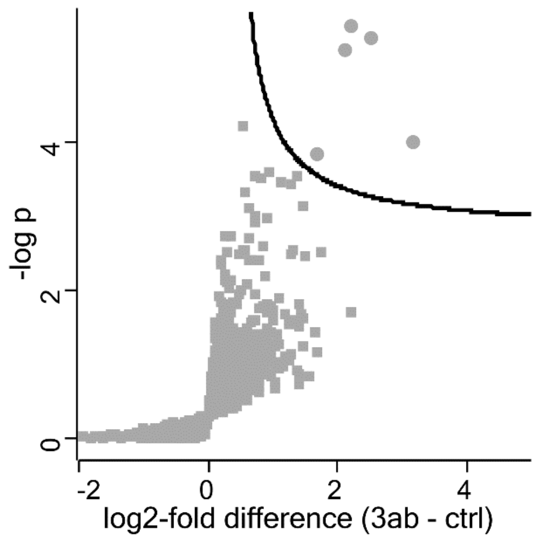

Figure S24. One-sided volcano plot for the labelling of murine liver with diphenyliodonium probe **3ab** (30 µM, 1 h) showing the distribution of quantified proteins according to –log p value and difference of proteins in probed (with probe **3ab**) vs. non-probed samples. Proteins above the line are considered statistically significant (p-value <0.01). ● = oxidoreductase, ● = potential off-target.

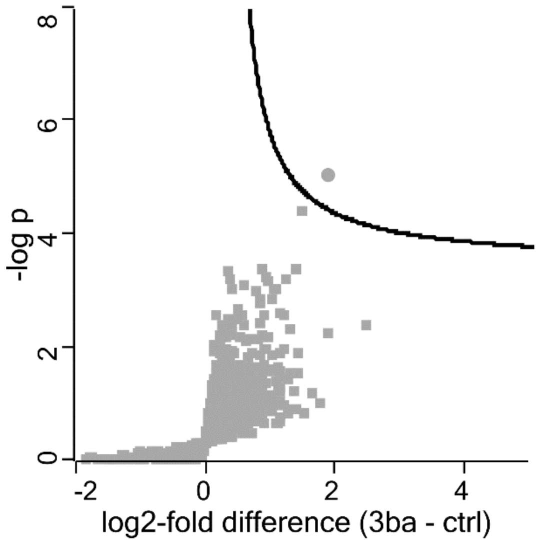

Figure S25. One-sided volcano plot for the labelling of murine liver with diphenyliodonium probe **3ba** (30 µM, 1 h) showing the distribution of quantified proteins according to –log p value and difference of proteins in probed (with probe **3ba**) vs. non-probed samples. Proteins above the line are considered statistically significant (p-value <0.01). ● = oxidoreductase, ● = potential off-target.

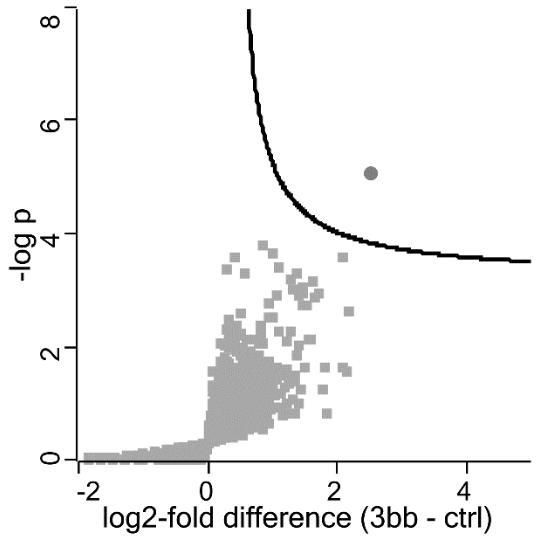

Figure S26. One-sided volcano plot for the labelling of murine liver with diphenyliodonium probe **3bb** (30 µM, 1 h) showing the distribution of quantified proteins according to –log p value and difference of proteins in probed (with probe **3bb**) vs. non-probed samples. Proteins above the line are considered statistically significant (p-value <0.01). ● = oxidoreductase, ● = potential off-target.

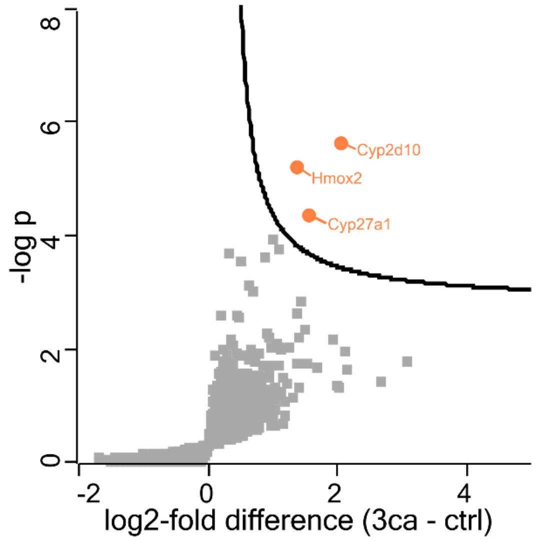

Figure S27. One-sided volcano plot for the labelling of murine liver with diphenyleneiodonium probe **3ca** (30 µM, 1 h) showing the distribution of quantified proteins according to –log p value and difference of proteins in probed (with probe **3ca**) vs. non-probed samples. Proteins above the line are considered statistically significant (p-value <0.01). ● = oxidoreductase, ● = potential off-target.

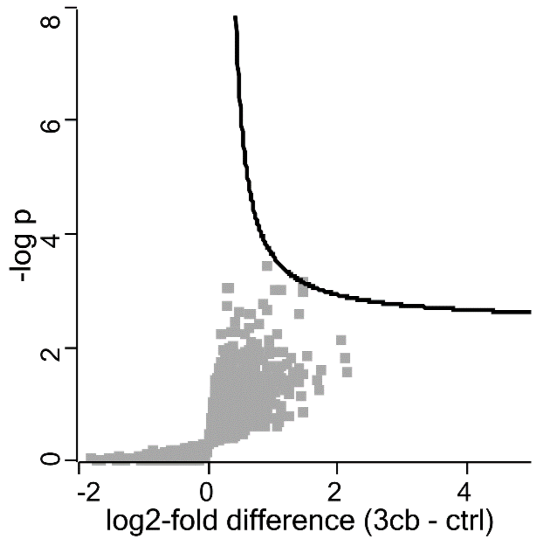

Figure S28. One-sided volcano plot for the labelling of murine liver with diphenyleneiodonium probe **3cb** (30 µM, 1 h) showing the distribution of quantified proteins according to –log p value and difference of proteins in probed (with probe **3cb**) vs. non-probed samples. Proteins above the line are considered statistically significant (p-value <0.01). ● = oxidoreductase, ● = potential off-target.

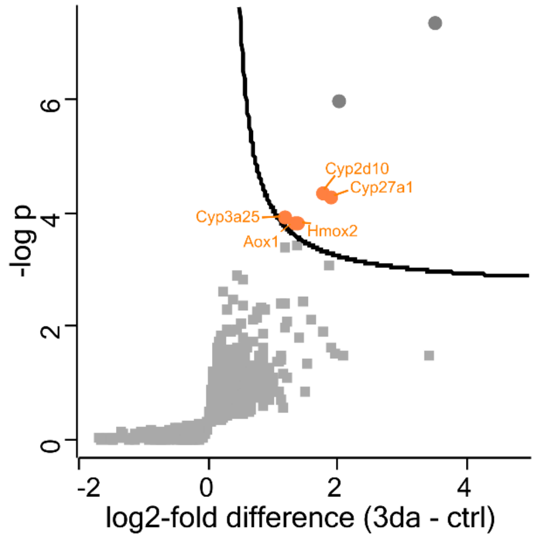

Figure S29. One-sided volcano plot for the labelling of murine liver with diphenyleneiodonium probe **3da** (30 µM, 1 h) showing the distribution of quantified proteins according to –log p value and difference of proteins in probed (with probe **3da**) vs. non-probed samples. Proteins above the line are considered statistically significant (p-value <0.01). ● = oxidoreductase, ● = potential off-target.

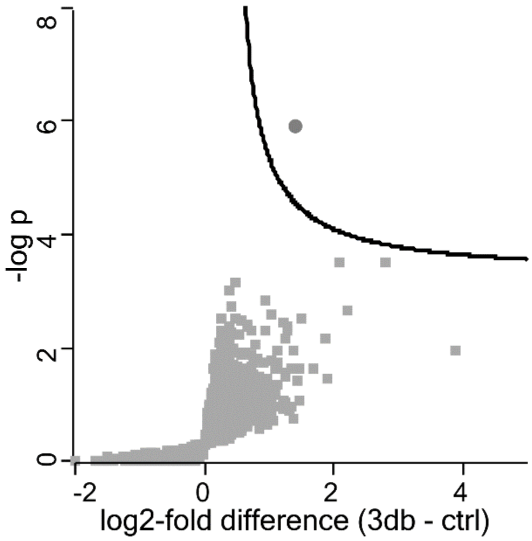

Figure S30. One-sided volcano plot for the labelling of murine liver with diphenyleneiodonium probe **3db** (30 µM, 1 h) showing the distribution of quantified proteins according to –log p value and difference of proteins in probed (with probe **3db**) vs. non-probed samples. Proteins above the line are considered statistically significant (p-value <0.01). ● = oxidoreductase, ● = potential off-target.

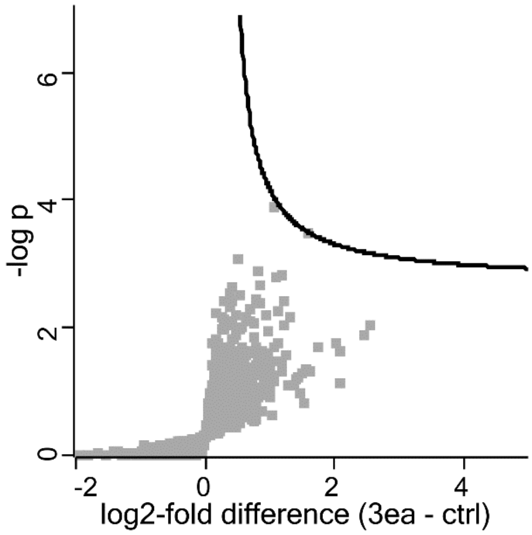

Figure S31. One-sided volcano plot for the labelling of murine liver with diphenyleneiodonium probe **3ea** (30 µM, 1 h) showing the distribution of quantified proteins according to –log p value and difference of proteins in probed (with probe **3ea**) vs. non-probed samples. Proteins above the line are considered statistically significant (p-value <0.01). ● = oxidoreductase, ● = potential off-target.

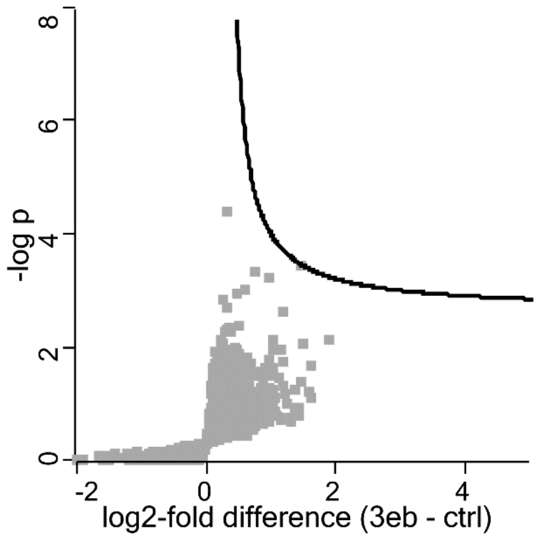

Figure S32. One-sided volcano plot for the labelling of murine liver with diphenyleneiodonium probe **3eb** (30 µM, 1 h) showing the distribution of quantified proteins according to –log p value and difference of proteins in probed (with probe **3eb**) vs. non-probed samples. Proteins above the line are considered statistically significant (p-value <0.01). ● = oxidoreductase, ● = potential off-target.

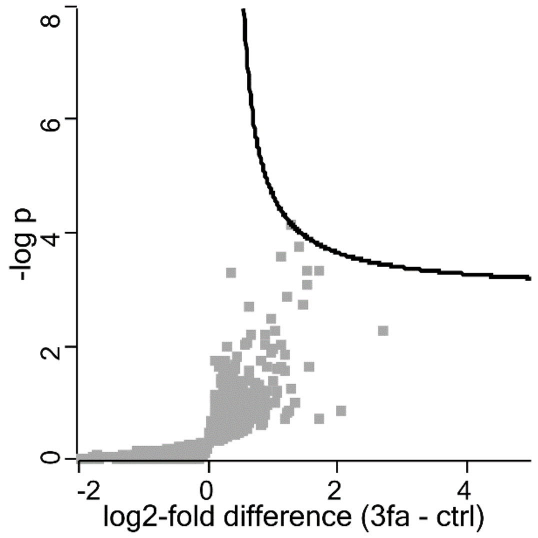

Figure S33. One-sided volcano plot for the labelling of murine liver with diphenylenebromonium probe **3fa** (30 µM, 1 h) showing the distribution of quantified proteins according to –log p value and difference of proteins in probed (with probe **3fa**) vs. non-probed samples. Proteins above the line are considered statistically significant (p-value <0.01). ● = oxidoreductase, ● = potential off-target.

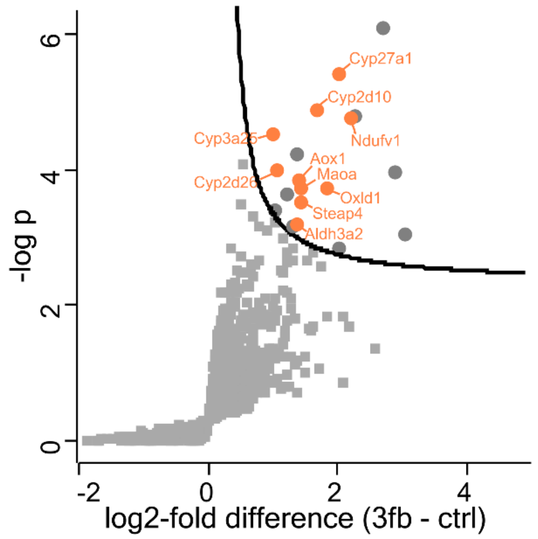

Figure S34. One-sided volcano plot for the labelling of murine liver with diphenylenebromonium probe **3fb** (30 µM, 1 h) showing the distribution of quantified proteins according to –log p value and difference of proteins in probed (with probe **3fb**) vs. non-probed samples. Proteins above the line are considered statistically significant (p-value <0.01). ● = oxidoreductase, ● = potential off-target.

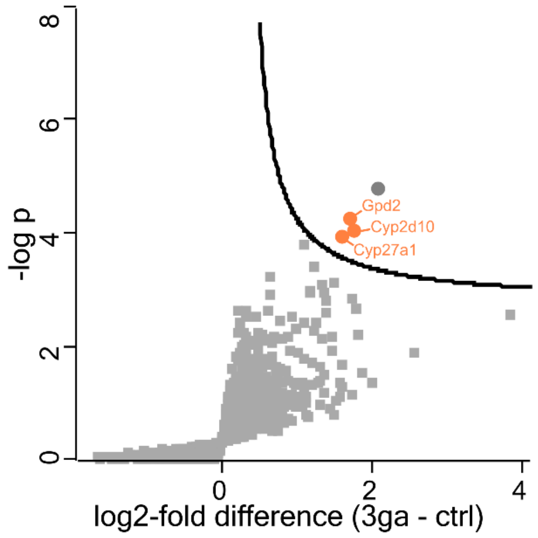

Figure S35. One-sided volcano plot for the labelling of murine liver with diphenylenebromonium probe **3ga** (30 µM, 1 h) showing the distribution of quantified proteins according to –log p value and difference of proteins in probed (with probe **3ga**) vs. non-probed samples. Proteins above the line are considered statistically significant (p-value <0.01). ● = oxidoreductase, ● = potential off-target.

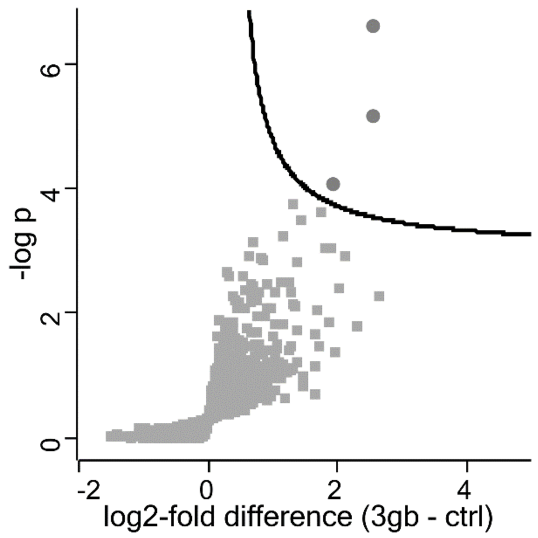

Figure S36. One-sided volcano plot for the labelling of murine liver with diphenylenebromonium probe **3gb** (30 µM, 1 h) showing the distribution of quantified proteins according to –log p value and difference of proteins in probed (with probe **3gb**) vs. non-probed samples. Proteins above the line are considered statistically significant (p-value <0.01). ● = oxidoreductase, ● = potential off-target.

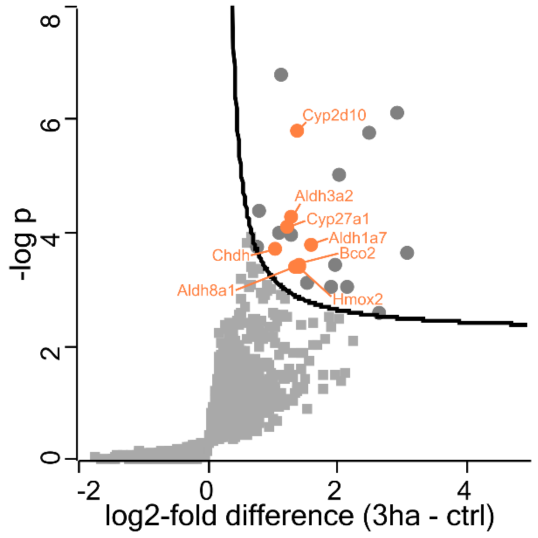

Figure S37. One-sided volcano plot for the labelling of murine liver with diphenylenechloronium probe **3ha** (30 µM, 1 h) showing the distribution of quantified proteins according to –log p value and difference of proteins in probed (with probe **3ha**) vs. non-probed samples. Proteins above the line are considered statistically significant (p-value <0.01). ● = oxidoreductase, ● = potential off-target.

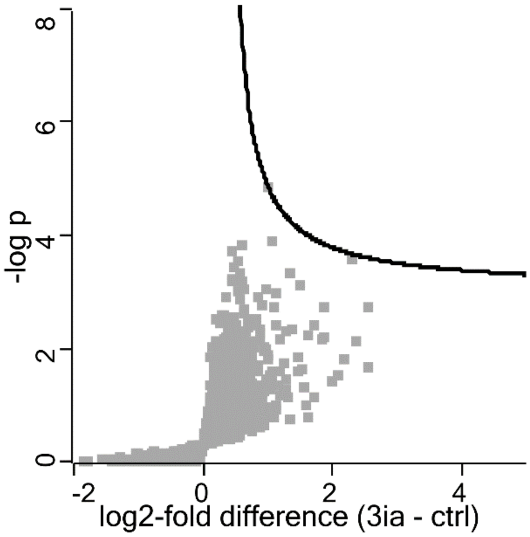

Figure S38. One-sided volcano plot for the labelling of murine liver with diphenylenechloronium probe **3ia** (30 µM, 1 h) showing the distribution of quantified proteins according to –log p value and difference of proteins in probed (with probe **3ia**) vs. non-probed samples. Proteins above the line are considered statistically significant (p-value <0.01). ● = oxidoreductase, ● = potential off-target.

Table S1. Fold change values for all significantly enriched proteins vs. control (no probe added) with probes **3aa-3ia** from multiple comparison corrected pairwise test (FDR = 5%).[a] Fold change values highlighted in orange represent values for proteins significantly enriched by the respective probe.

Protein		Probe															
Gene name	EC[b]	**3aa**	**3ab**	**3ba**	**3bd**	**3ca**	**3cb**	**3da**	**3db**	**3ea**	**3eb**	**3fa**	**3fb**	**3ga**	**3gb**	**3ha**	**3ia**
Rdh16	1.1.1.105	2.48	1.14	1.49	1.26	1.15	1.21	1.14	1.19	0.94	1.15	1.21	1.09	1.40	1.20	1.73	1.40
Rdh7	1.1.1.105	5.38	1.90	2.07	1.33	1.12	1.02	1.09	1.04	1.02	1.05	1.09	1.05	1.41	1.10	1.71	2.13
Gpd2	1.1.5.3	1.41	1.07	1.52	1.34	2.13	1.48	3.66	1.33	0.84	1.03	2.33	1.69	3.28	1.58	1.31	1.38
Chdh	1.1.99.1	1.20	1.00	1.10	1.04	1.01	0.96	0.97	1.02	1.01	1.08	1.37	1.04	1.06	0.96	2.06	1.08
Aldh8a1	1.2.1.-	1.15	1.01	1.07	1.00	1.04	1.08	1.09	1.02	1.02	1.03	1.35	1.45	1.24	1.07	2.52	1.61
Aldh1a7	1.2.1.3	1.92	1.15	2.25	1.20	1.03	1.01	1.10	1.04	1.11	1.09	1.17	1.34	2.25	1.32	3.02	1.85
Aldh3a2	1.2.1.3	1.20	1.09	1.06	1.07	1.06	1.09	1.05	1.05	0.97	1.00	1.19	2.61	1.19	1.23	2.46	1.29
Maoa	1.4.3.4	1.52	1.08	1.18	0.96	0.88	0.93	0.98	0.82	0.77	0.78	1.99	2.70	2.80	2.37	2.06	1.52
Prodh	1.5.5.2	2.56	1.02	1.41	1.17	1.20	1.10	0.95	1.03	0.86	1.01	1.40	1.04	1.23	0.90	1.06	1.04
Ndufv1	1.6.5.3;1.6.99.3	1.07	1.27	0.99	1.61	1.72	1.94	1.42	2.04	1.55	1.06	1.75	4.67	1.06	2.44	1.58	0.95
Bco2	1.13.11.71	1.20	0.91	1.11	1.01	1.02	1.11	1.01	1.30	0.88	0.95	2.20	1.65	3.47	1.36	2.68	1.96
Cyp27a1	1.14.13.15	3.12	2.60	2.64	3.10	2.92	2.65	3.70	2.82	1.40	1.03	2.66	4.05	3.05	3.34	2.31	1.78
Fmo1	1.14.13.8	3.22	1.59	1.79	1.51	1.01	1.03	1.07	1.02	0.82	0.89	1.12	0.92	1.61	1.05	1.60	1.41
Cyp3a25	1.14.14.1	2.02	1.66	1.85	2.03	2.12	1.73	2.30	1.72	1.20	1.15	1.54	2.02	2.14	1.79	1.60	1.30
Cyp2d10	1.14.14.1	1.71	1.38	1.86	2.08	4.19	2.74	3.44	2.04	1.22	1.13	2.44	3.19	3.37	2.51	2.58	2.56
Cyp2d26	1.14.14.1	1.20	1.10	1.23	1.28	1.39	1.56	1.19	1.17	0.97	1.06	1.09	2.07	1.22	1.21	1.20	1.23
Hmox2	1.14.99.3	2.69	1.28	1.98	1.79	2.58	1.60	2.58	1.43	1.00	1.00	1.71	1.49	2.59	1.47	2.66	2.10
Steap4	1.16.1.-	1.23	1.11	1.10	1.22	1.09	1.60	1.40	1.63	0.94	0.90	1.42	2.69	1.93	2.57	1.35	1.34
Xdh	1.17.1.4;1.17.3.2	1.86	1.77	1.98	2.45	1.56	1.59	1.24	1.17	1.30	1.03	1.25	1.60	1.15	1.08	1.10	1.04
Aox1	1.17.3.-;1.2.3.1	1.69	1.88	2.39	2.46	1.85	1.81	2.52	2.32	1.76	1.06	2.17	2.65	2.55	2.74	1.25	1.01
Vkorc1l1	1.17.4.4	3.15	1.11	1.15	0.91	1.14	1.16	1.36	0.91	1.17	1.19	1.55	1.61	1.86	1.34	1.49	1.11
Nnmt	2.1.1.1	1.01	0.97	0.96	1.03	1.08	0.98	1.00	0.97	0.96	0.94	1.62	1.16	1.47	0.95	4.46	1.48
Ip6k1	2.7.4.21	1.32	0.63	1.37	0.85	0.74	0.94	0.66	1.43	0.87	0.77	2.79	0.73	1.17	0.59	3.88	0.68
Nfs1	2.8.1.7	2.03	4.37	1.72	2.63	1.06	1.09	0.94	1.22	1.04	1.03	0.97	2.62	1.08	2.17	1.07	1.15
Irgm1	3.6.5.-	1.78	1.01	1.34	1.18	1.14	1.10	1.08	1.09	0.91	1.02	1.10	1.19	1.27	1.12	1.81	1.37
Hmgcl	4.1.3.4	1.03	1.15	1.01	1.02	1.01	0.91	1.06	0.92	1.13	0.96	1.10	2.32	1.08	1.50	1.54	1.01
Pcbd2	4.2.1.96	1.05	1.54	1.05	1.54	1.00	0.92	1.02	0.99	1.19	0.98	1.09	2.49	1.08	3.84	1.03	0.90
Cisd3	n.a.	2.12	0.95	1.91	1.00	2.08	1.38	2.19	1.34	1.27	1.26	1.84	1.43	2.14	1.19	2.15	1.79
Sigmar1	n.a.	1.08	0.98	0.99	1.13	0.94	1.02	0.96	1.01	0.97	0.85	0.94	1.03	1.26	0.97	2.45	1.44
Urb1	n.a.	1.66	1.66	2.15	1.64	2.00	1.87	1.70	2.63	1.78	1.94	1.48	2.06	1.55	1.88	1.81	1.28
Scarb1	n.a.	3.52	1.10	1.92	1.06	0.82	1.08	0.86	0.98	0.43	1.18	1.02	0.96	1.04	0.89	1.10	1.29
Apoc4	n.a.	2.83	2.51	1.92	1.77	1.59	1.80	3.00	1.14	1.79	2.80	2.89	2.42	2.49	3.53	7.57	2.83
Rasl2-9	n.a.	4.18	1.80	2.70	2.00	2.05	2.10	2.20	1.56	1.09	1.26	1.56	1.59	2.75	1.31	4.06	3.09
Atg2a	n.a.	1.38	1.13	1.33	1.07	1.15	1.17	1.09	1.33	1.04	1.29	1.10	1.03	1.09	1.03	1.74	1.25
Wdr43	n.a.	1.19	1.00	1.68	1.47	1.79	1.43	0.98	1.40	0.98	1.52	1.40	1.40	1.36	1.06	3.77	1.22
Glrx5	n.a.	3.50	3.22	1.89	2.98	1.15	1.49	1.11	1.36	1.17	1.52	1.10	1.95	2.34	1.96	1.03	1.14
Coq4	n.a.	2.60	1.08	1.42	1.34	0.67	0.83	1.26	1.42	1.19	1.45	1.06	1.10	1.30	1.09	1.88	1.00
Bola2	n.a.	1.21	2.70	1.89	2.47	1.14	2.14	1.22	1.89	1.09	1.58	1.22	4.05	2.17	4.38	1.89	1.26
Bola3	n.a.	5.54	5.73	3.77	5.74	1.91	2.79	4.11	4.25	2.32	1.16	2.89	6.47	4.23	5.90	1.18	1.62
Stard7	n.a.	3.91	1.00	1.83	1.51	0.93	1.47	1.54	1.23	1.47	0.81	0.82	1.35	1.56	0.95	1.86	1.09
Mcam	n.a.	7.25	9.02	5.60	3.48	4.30	4.16	11.32	6.95	5.81	3.78	6.52	7.37	5.93	6.28	8.49	4.25
Saraf	n.a.	1.53	0.90	0.94	1.27	0.67	0.72	1.23	0.79	0.67	0.51	0.89	0.93	1.68	0.92	2.91	1.23
Hpx	n.a.	1.12	1.34	1.08	1.54	1.01	1.39	0.97	1.51	0.98	0.96	0.93	2.49	1.21	1.84	1.16	0.97
Derl1	n.a.	1.54	1.04	1.26	1.07	1.07	1.15	1.18	1.16	1.18	1.08	1.17	1.00	1.40	1.08	2.17	1.41
Oxld1	n.a.	3.54	1.44	1.57	1.77	2.71	1.53	2.16	1.34	0.85	0.98	2.31	3.56	2.52	2.11	2.73	1.86
Sdhc	n.a.	2.55	0.95	1.11	1.11	1.01	0.96	1.08	0.95	0.75	0.99	0.80	0.93	1.13	0.90	1.04	1.42
Tmx2	n.a.	1.87	1.28	1.17	1.01	0.90	1.08	1.05	0.96	1.22	0.97	1.10	1.06	1.16	1.07	1.70	1.14
Apmap	n.a.	1.19	0.99	1.01	1.02	0.85	0.95	0.90	0.97	0.92	0.97	1.41	1.01	1.15	1.06	5.59	1.37
Isca2	n.a.	3.18	2.42	1.76	4.29	2.10	2.40	2.79	3.68	1.62	0.94	3.28	4.88	3.35	5.84	1.28	1.18
Trmt112	n.a.	0.87	1.10	1.10	1.29	1.18	1.02	1.12	0.98	0.96	0.81	1.13	1.24	1.35	1.21	4.09	1.01
Stard5	n.a.	1.80	1.45	2.84	1.99	0.82	0.93	0.86	0.77	0.89	0.93	0.92	1.01	0.82	0.94	1.42	0.87
Nfu1	n.a.	1.99	1.85	1.91	2.53	1.73	2.91	1.18	2.79	1.26	1.84	1.75	8.29	3.05	3.69	6.20	5.86
Slc25a20	n.a.	4.14	4.66	2.11	2.78	1.02	1.04	1.06	0.94	0.92	1.11	0.88	1.20	1.30	1.10	1.37	1.37

[a]Please note: the threshold for significance (from pairwise test) differs throughout the different probes.

[b]n.a. = EC not annotated.

Table S2. List of significantly enriched proteins bearing at least one reactive cysteine residue (as termed and listed by Weerapana, Cravatt and co-workers).[4] All reactive cysteines of the proteins are given. $R_{10:1}$ < 2 state highly reactive cysteine residues. n.a. = not annotated.

SwissprotID	Gene name	EC	$R_{10:1}$
Q80Y14	Glrx5	n.a.	5.9 (human)<br>5.28 (murine)<br>6.22 (m)
Q9QZ23	Nfu1	n.a.	7.56 (h)
O08749	Dld	1.8.1.4	4.51 (m)<br>8 (h)
Q9CXT8	Pmpcb	3.4.24.64	5.9 (h)<br>4.77 (m)<br>4.96 (m)
Q9JLJ2	Aldh9a1	1.2.1.3<br>1.2.1.47	0.92 (h)<br>1.82 (h)
Q8BP40	Acp6	3.1.3.2	2.99 (m)
Q8VDQ1	Ptgr2	1.3.1.48	4.52 (m)
A6H611	Mipep	3.4.24.59	7.45 (h)

Table S3. List of significantly enriched proteins bearing at least one reactive lysine residue (as termed and listed by Hacker, Cravatt and co-workers).[5] All reactive lysines of the proteins are given. $R_{10:1}$ < 2: highly reactive lysine, 2 < $R_{10:1}$ < 5: Lys with medium reactivity, $R_{10:1}$ > 5: Lys with low reactivity. n.a. = not annotated.

SwissprotID	Gene name	EC	$R_{10:1}$
Q80Y14	Glrx5	n.a.	10
Q8WTY4	Ciapin1	n.a.	10
O70252	Hmox2	1.14.99.3	4.29<br>4.46<br>10
Q9CZL5	Pcbd2	4.2.1.96	3.82
Q91WS0	Cisd1	n.a.	0.21<br>9.21
P37040	Por	1.6.2.4	8.53
O08749	Dld	1.8.1.4	4.22<br>10 (5 residues)
Q9CQF9	Pcyox1	1.8.3.5	10 (2 residues)
Q9CY27	Tecr	1.3.1.93	2.95<br>4.17<br>10 (3 residues)
P50544	Acadvl	1.3.8.9	4.01<br>4.79<br>5.72<br>8.94<br>9.05<br>10 (3 residues)

Q9DC70	Ndufs7	1.6.5.3<br>1.6.99.3	10
Q9JLJ2	Aldh9a1	1.2.1.3<br>1.2.1.47	0.82<br>3.84<br>10 (2 residues)
P68372	Tubb4b	n.a.	8.27<br>9.59<br>10 (7 residues)
P62245	Rps15a	n.a.	10 (3 residues)
Q9D051	Pdhb	n.a.	10 (2 residues)

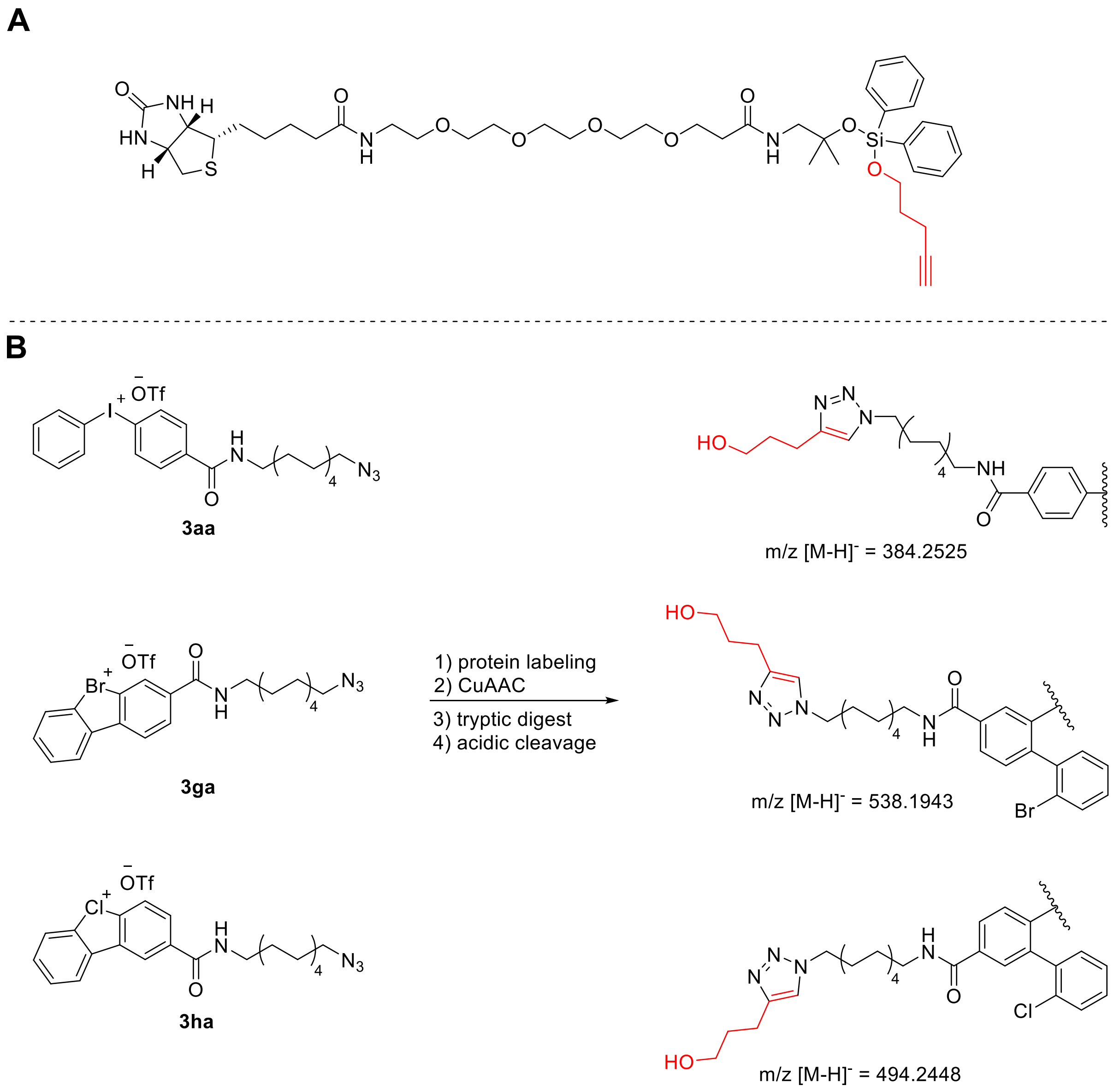

Figure S39. (A) Structure of dialkoxydiphenylsilane(DADPS)-Biotin alkyne click linker. The structural fragment remaining on the labeled protein after click reaction and mild acidic cleavage (10% formic acid) is depicted in red. (B) Structures and masses of the corresponding probe-linker adducts after labeling, click reaction, tryptic digest, and mild acidic cleavage.

Table S4. List of enzymes found in both *in cellulo* ABPP studies and active site studies, as well as corresponding probes responsible for their labeling and identification.

**Protein name**	**Probes *in cellulo*** pairwise test	**Probes *in cellulo*** ANOVA	**Probes active site search**
Rdh7	3aa	3aa (3ab, 3ba, 3ia, 3ha)[a]	3aa, 3ha
Chdh	3ha	3ha (3fa)	3ha
Aox1	3da, 3fb	All (except 3eb + 3ia)	3aa
Aldh8a1	3ha	3ha (3ia, 3fb, 3ga, 3fa)	3ga
Aldh1a1	-	3aa, 3ab, 3ha	3aa
Nfs1	3aa, 3ab, 3fb	3aa, 3ab, 3bb, 3fb (3ba, 3gb)	3aa
Isca2	3fb, 3gb	All (except 3eb + 3ia)	3aa
Tubb4b	-	3ha	3ha
Hmgcl	3fb	3fb (3gb, 3ha)	3ha

[a] Probes in grey induced enrichment that was less prominent when compared to the ones in black.

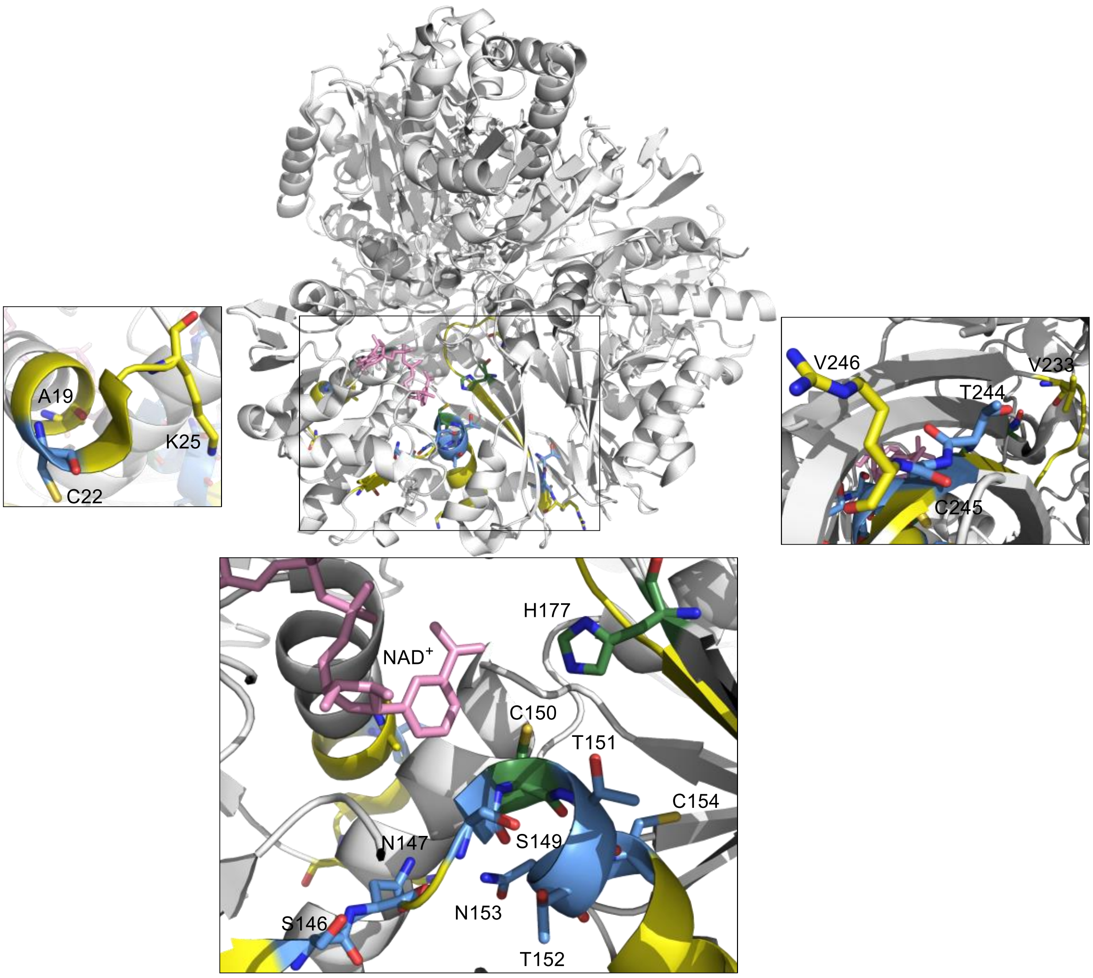

Figure S40. Side and zoomed views of the crystal structure (PDBE 6lgj) of mGAPDH (PDB P16858) depicting the potentially labeled amino acids (blue, shown in sticks) on the modified peptide fragments (yellow, first and last AA shown in sticks) and important active site residues (green, shown in sticks). Please note that active site residue C150 was potentially labeled as well. Co-crystallized NAD^+ is shown in pink sticks.

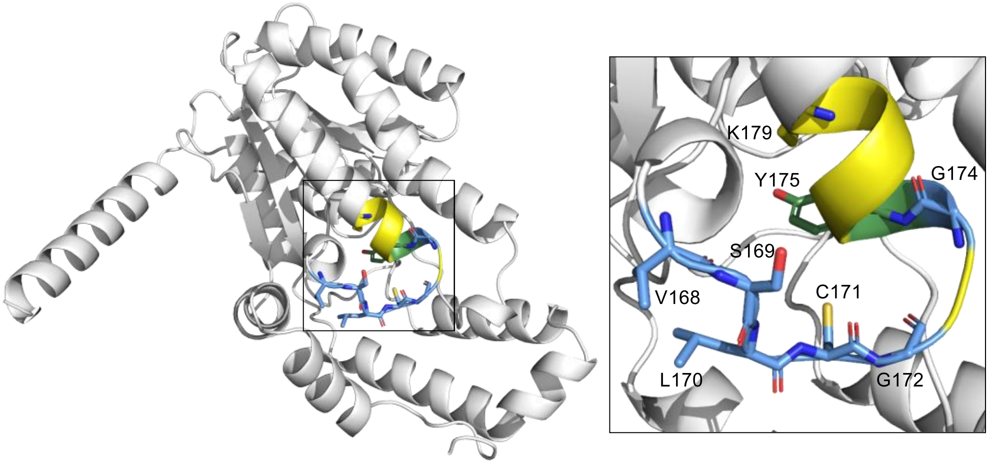

Figure S41. Side and zoomed views of the predicted AlphaFold structure of mRDH7 (PDB O88451) depicting the potentially labeled amino acids (blue, shown in sticks) on the modified peptide fragment (yellow, first and last AA shown in sticks) and the important active site residue (green, shown in sticks).

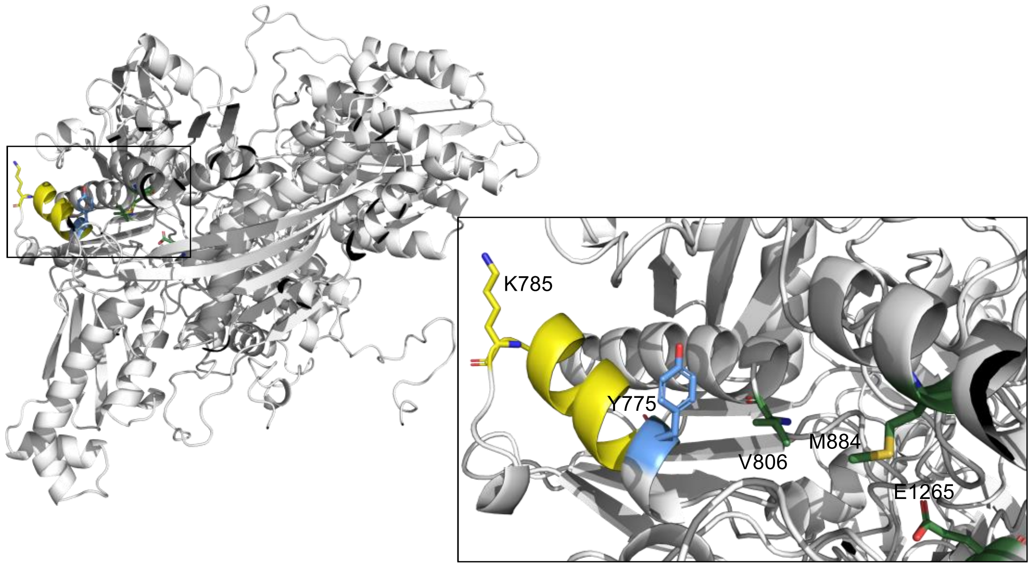

Figure S42. Side and zoomed views of the predicted AlphaFold structure of mAOX1 (PDB O54754) depicting the potentially labeled amino acid (blue, shown in sticks) on the modified peptide fragment (yellow, first and last AA shown in sticks) and important active site residues (green, shown in sticks).

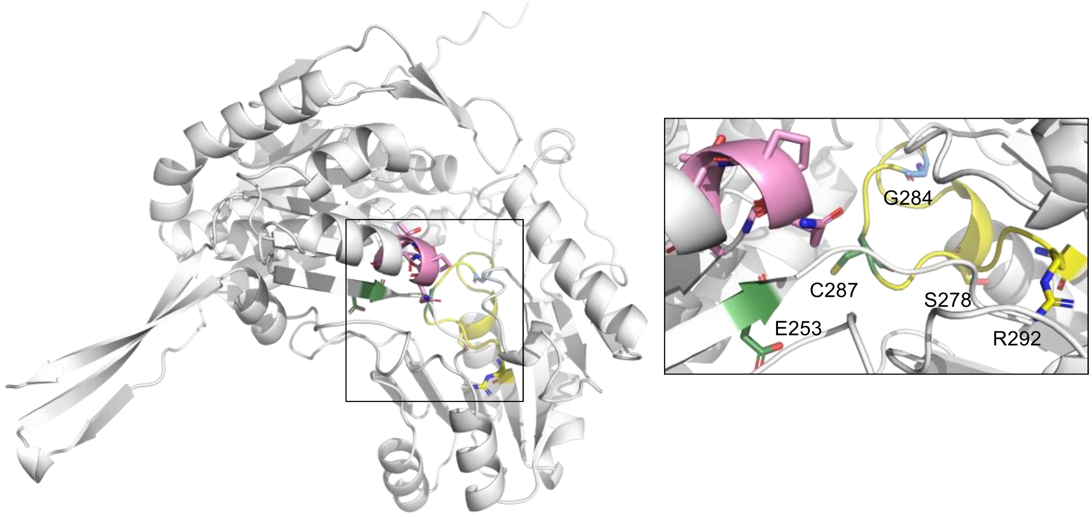

Figure S43. Side and zoomed views of the predicted AlphaFold structure of mALDH8A1 (PDB Q8BH00) depicting the potentially labeled amino acids (blue, shown in sticks) on the modified peptide fragment (yellow, first and last AA shown in sticks) and important active site residues (green, shown in sticks). Please note that active site residue C287 was potentially labeled as well. The cofactor (NAD^+) binding site is shown in pink sticks.

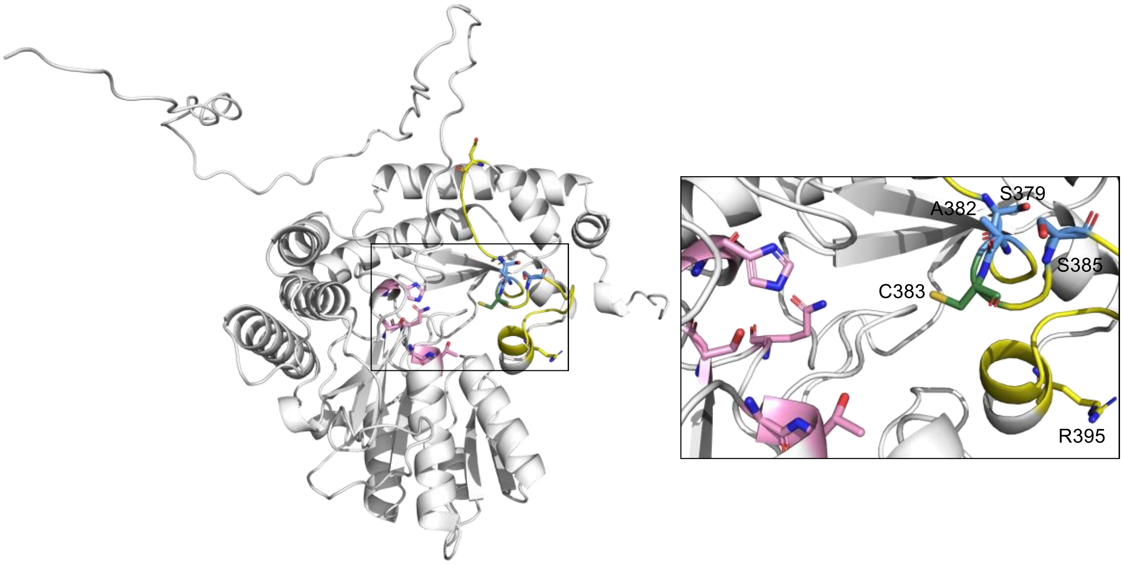

Figure S44. Side and zoomed views of the predicted AlphaFold structure of mNFS1 (PDB Q9Z1J3) depicting the potentially labeled amino acids (blue, shown in sticks) on the modified peptide fragment (yellow, first and last AA shown in sticks) and the important active site residue (green, shown in sticks; also responsible for [2Fe-2S] cluster binding). Please note that active site residue C383 was potentially labeled as well. The cofactor (PLP) binding sites are shown in pink sticks.

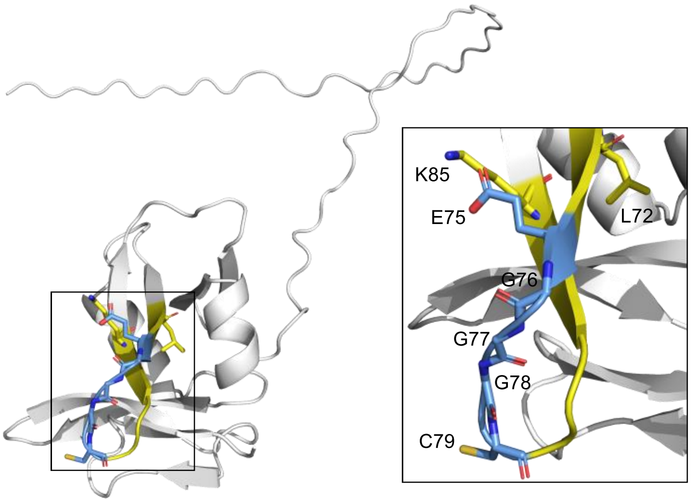

Figure S45. Side and zoomed views of the predicted AlphaFold structure of mISCA2 (PDB Q9DCB8) depicting the potentially labeled amino acids (blue, shown in sticks) on the modified peptide fragment (yellow, first and last AA shown in sticks). Residues E75 to C79 belong to a highly conserved sequence region in the Fe-S biosynthesis domain of ISCA2.[6]

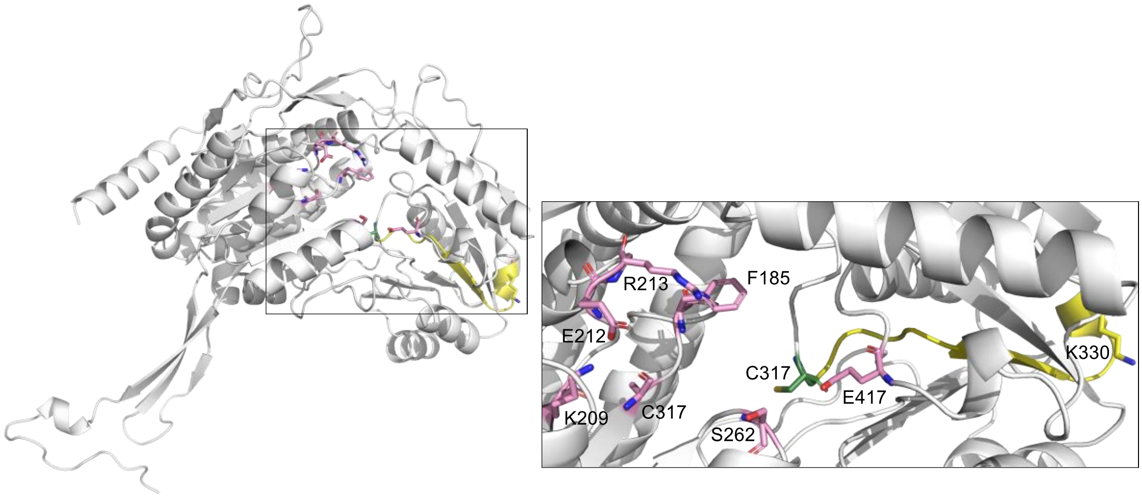

Figure S46. Side and zoomed views of the predicted AlphaFold structure of mALDH6A1 (PDB Q9EQ20) depicting the labeled active site amino acid (green, shown in sticks) on the modified peptide fragment (yellow, first and last AA shown in sticks. Residues responsible for cofactor (NAD^+) binding are shown in pink sticks.

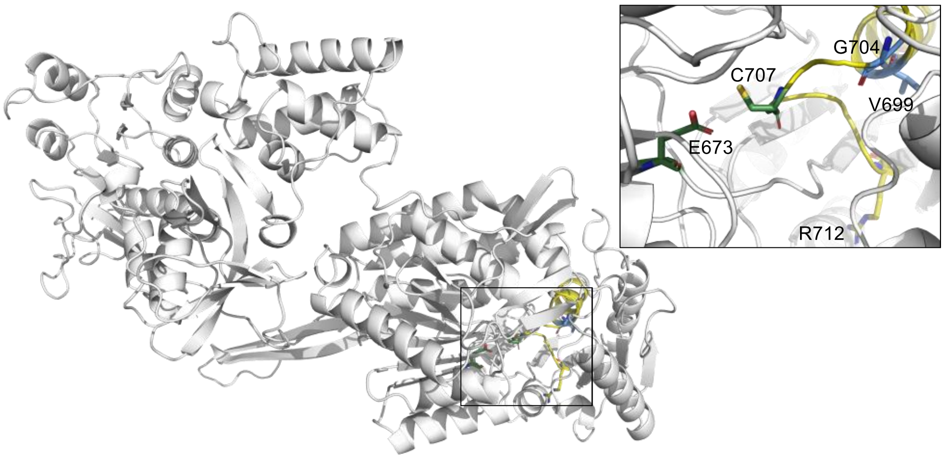

Figure S47. Side and zoomed views of the predicted AlphaFold structure of mALDH1L1 (PDB Q8R0Y6) depicting potentially labeled amino acids (blue, shown in sticks) on the modified peptide fragment (yellow, first and last AA shown in sticks) and important active site residues (green, shown in sticks). Please note that active site residue C707 was potentially labeled as well.

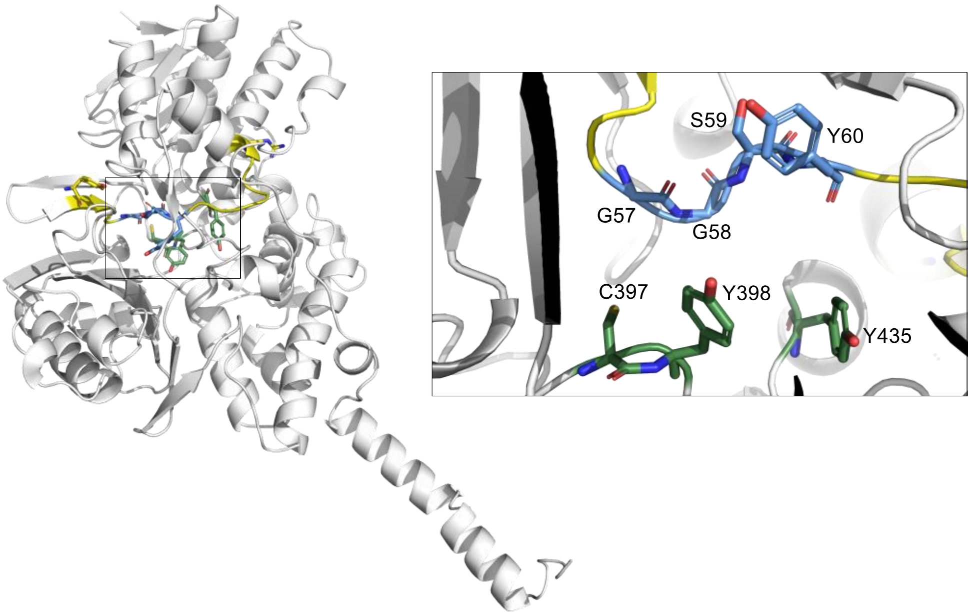

Figure S48. Side and zoomed views of the predicted AlphaFold structure of mMAOB (PDB Q8BW75) depicting the potentially labeled amino acids (blue, shown in sticks) on the modified peptide fragment (yellow, first and last AA shown in sticks) and the important active site residue (green, shown in sticks). C397 is responsible for the binding of the FAD-cofactor at the end of the substrate cavity and two nearly parallel tyrosyl residues (Y398 and Y435) form the so-called “aromatic cage”, which also plays a functional role in the catalytic mechanism of mMAOB.[7]

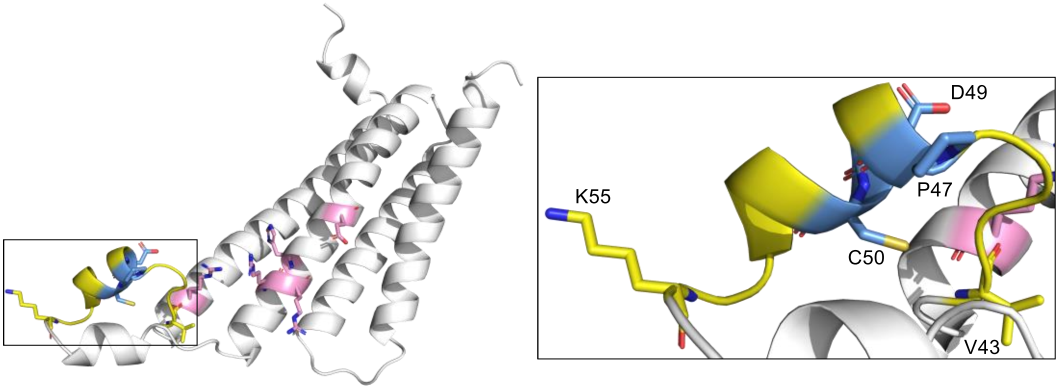

Figure S49. Side and zoomed views of the predicted AlphaFold structure of mMGST1 (PDB Q91VS7) depicting the potentially labeled amino acids (blue, shown in sticks) on the modified peptide fragment (yellow, first and last AA shown in sticks). Residues responsible for GSH binding in the active site are shown in pink sticks.[8]

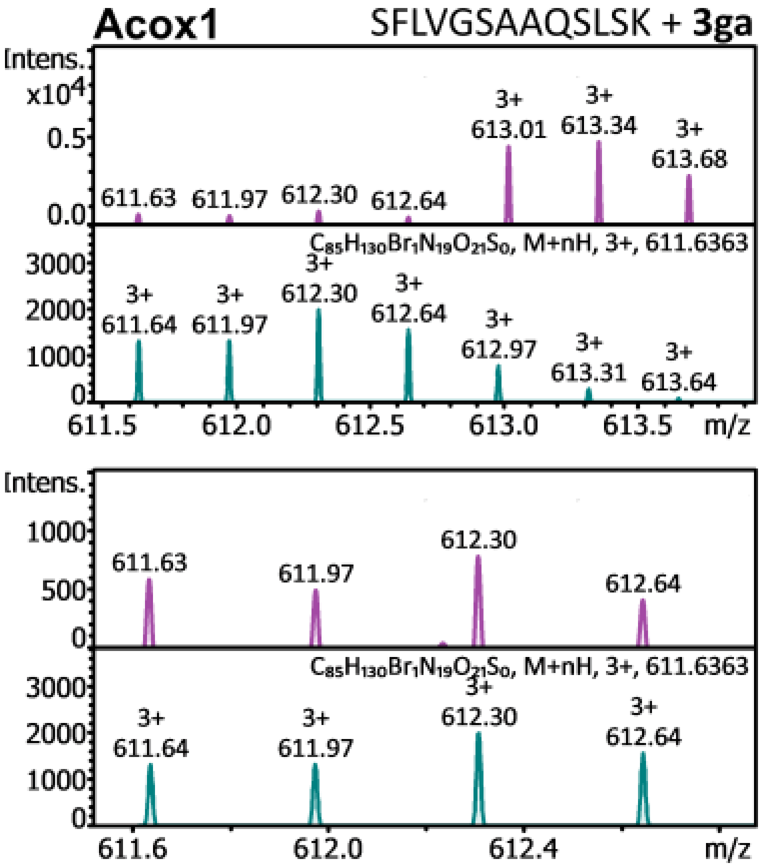

Figure S50. Measured (purple) and simulated (turquoise) isotope pattern of the peptide fragment SFLVGSAAQSLSK of Acox1 labeled by probe **3ga**.

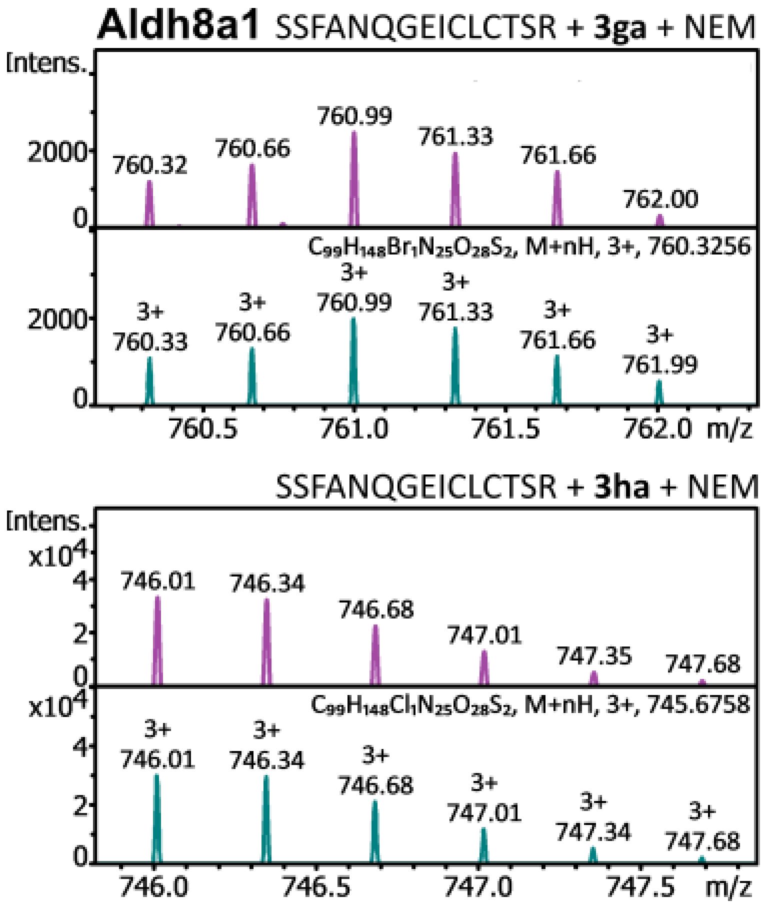

Figure S51. Measured (purple) and simulated (turquoise) isotope pattern of the peptide fragment SSFANQGEICLCTSR of Aldh8a1 labeled by probes **3ga** (top) and **3ha** (bottom). NEM = *N*-ethylmaleimide.

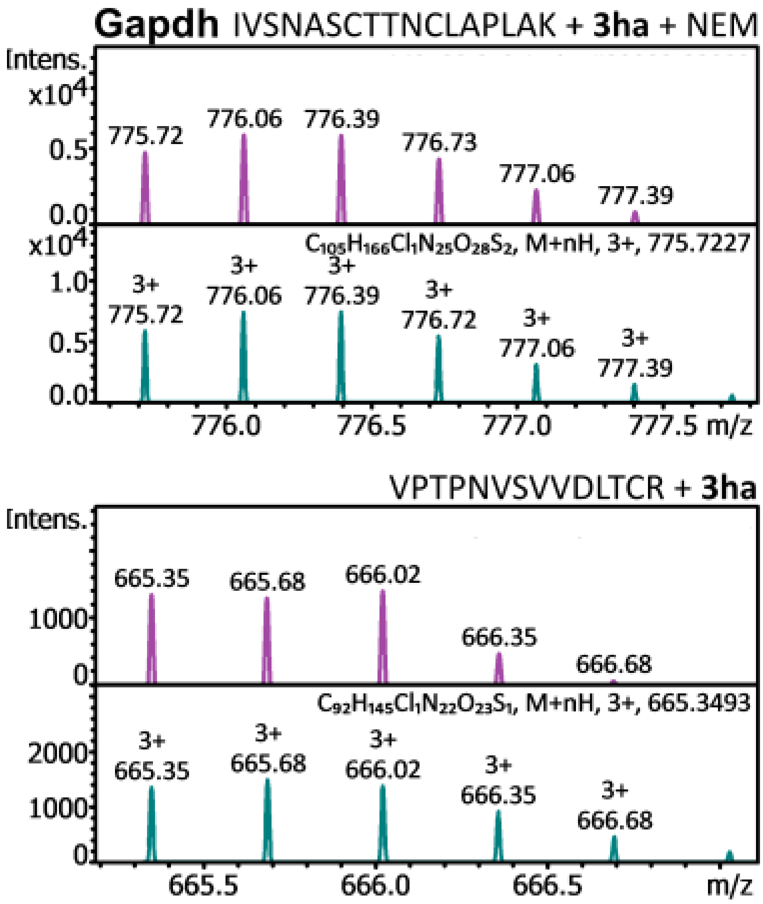

Figure S52. Measured (purple) and simulated (turquoise) isotope pattern of the peptide fragments IVSNASCTTNCLAPLAK (top) and VPTPNVSVVDLTCR (bottom) of Gapdh labeled by probe **3ha**. NEM = *N*-ethylmaleimide.

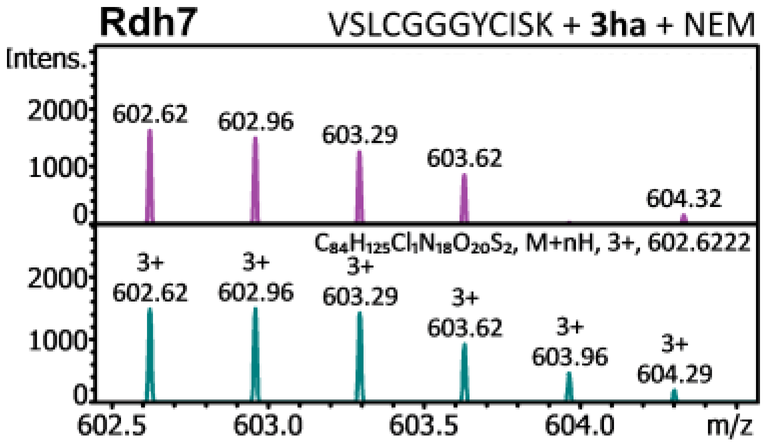

Figure S53. Measured (purple) and simulated (turquoise) isotope pattern of the peptide fragment VSLCGGGYCISK of Rdh7 labeled by probe **3ha**. NEM = *N*-ethylmaleimide.

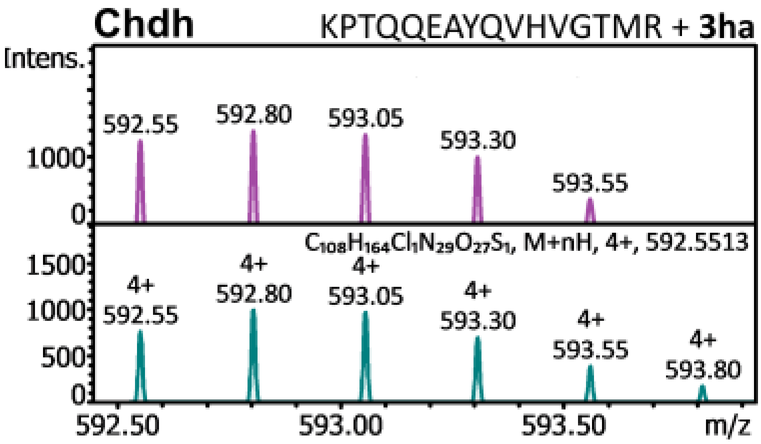

Figure S54. Measured (purple) and simulated (turquoise) isotope pattern of the peptide fragment KPTQQEAYQVHVGTMR of Chdh labeled by probe **3ha**.

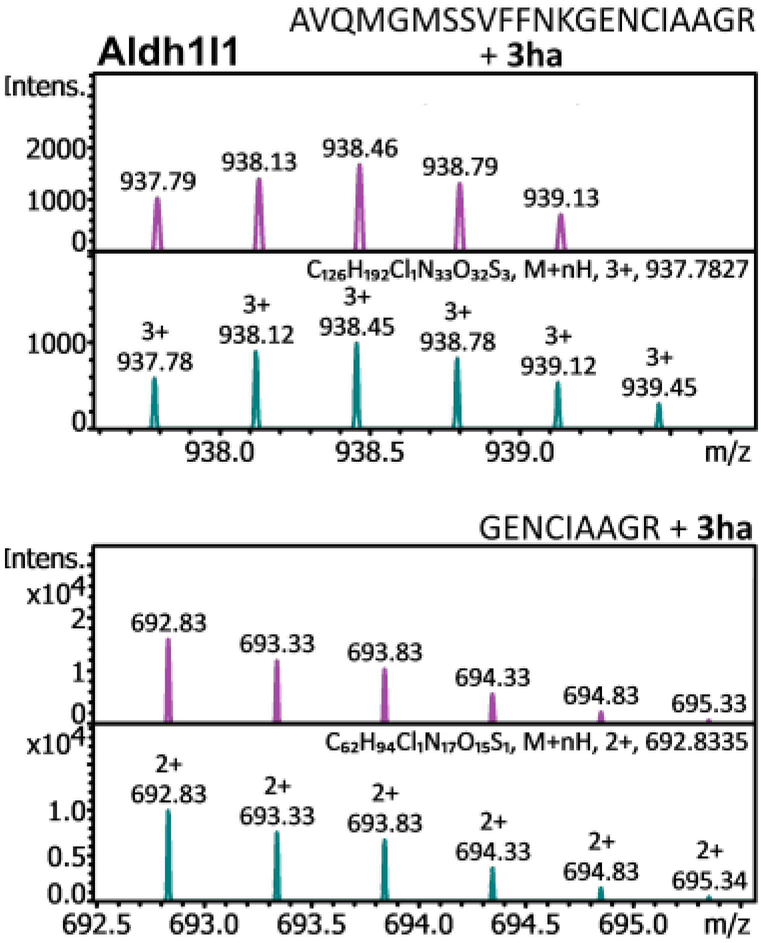

Figure S55. Measured (purple) and simulated (turquoise) isotope pattern of the peptide fragments AVQMGMSSVFFNKGENCIAAGR (top) and GENCIAAGR (bottom) of Aldh1l1 labeled by probe **3ha**.

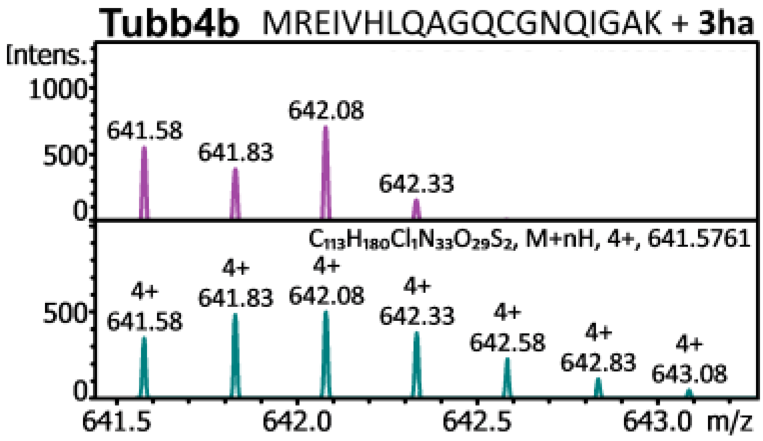

Figure S56. Measured (purple) and simulated (turquoise) isotope pattern of the peptide fragment MREIVHLQAGQCGNQIGAK of Tubb4b labeled by probe **3ha**. Please note that this fragment features an additional oxidation (m/z +16) on the methionine residue.

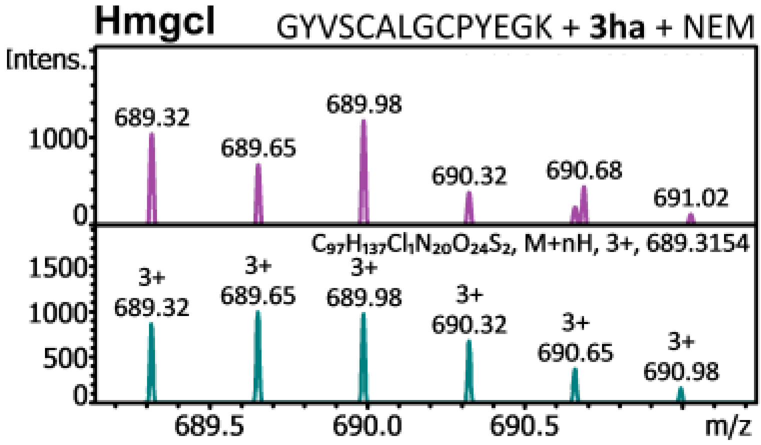

Figure S57. Measured (purple) and simulated (turquoise) isotope pattern of the peptide fragment GYVSCALGCPYEGK of Hmgcl labeled by probe **3ha**. NEM = *N*-ethylmaleimide.

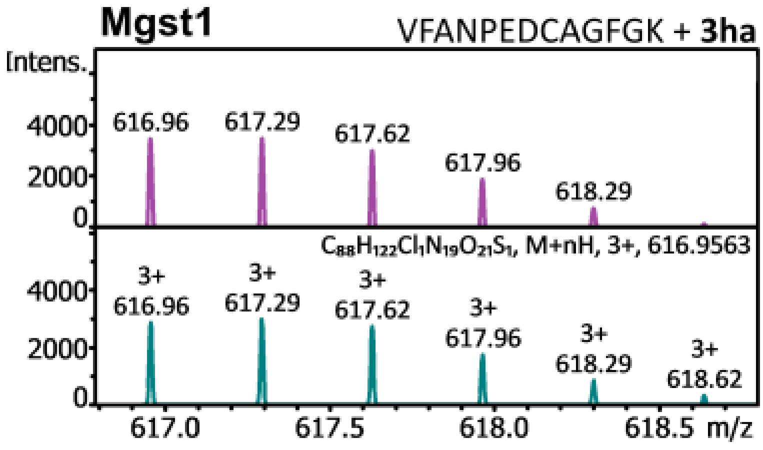

Figure S58. Measured (purple) and simulated (turquoise) isotope pattern of the peptide fragment VFANPEDCAGFGK of Mgst1 labeled by probe **3ha**.

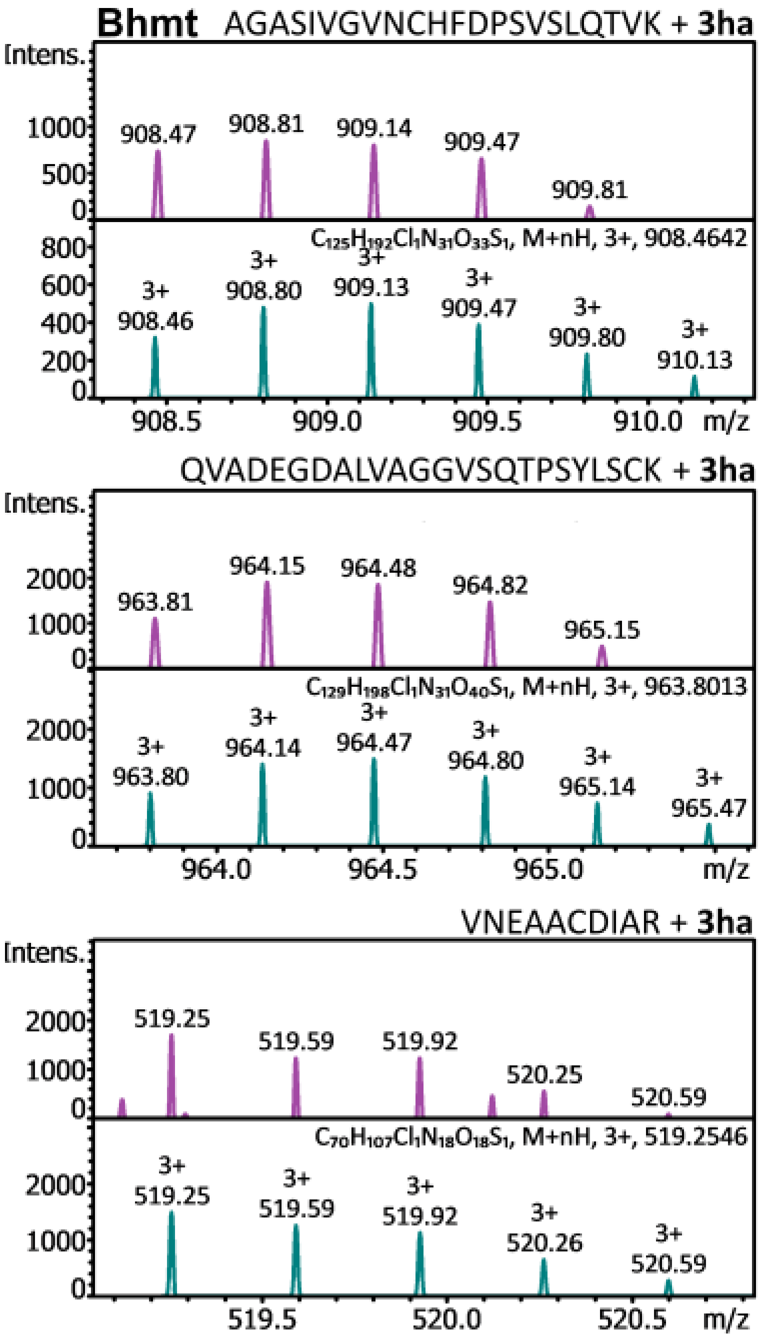

Figure S59. Measured (purple) and simulated (turquoise) isotope pattern of the peptide fragments AGASIVGVNCHFDPSVSLQTVK (top), QVADEGDALVAGGVSQTPSYLSCK (middle), and VNEAACDIAR (bottom) of Bhmt labeled by probe **3ha**.

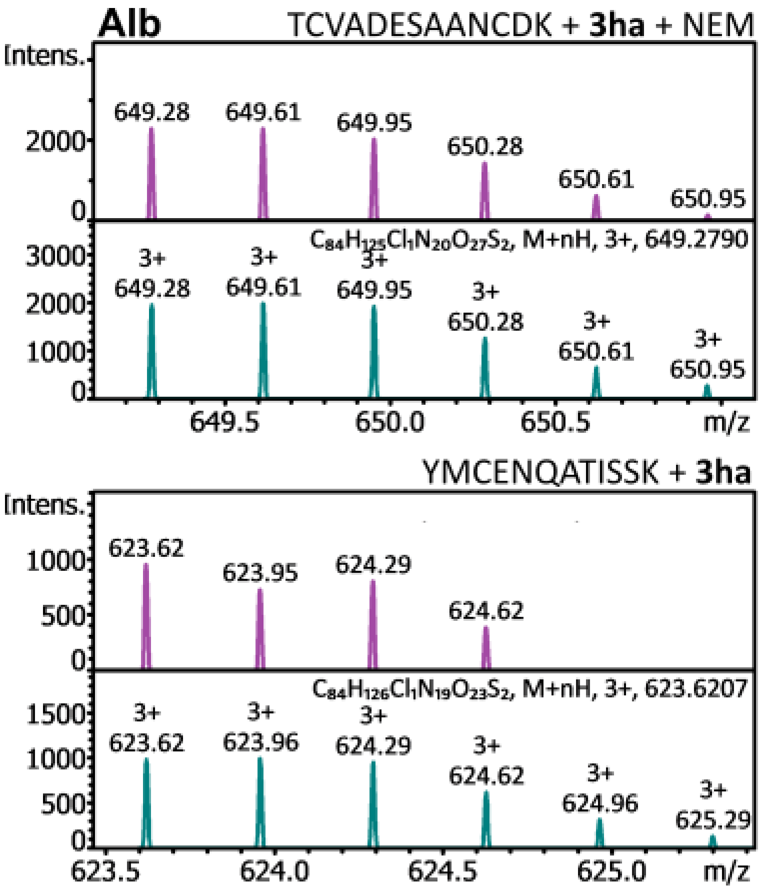

Figure S60. Measured (purple) and simulated (turquoise) isotope pattern of the peptide fragments TCVADESAANCDK (top) and YMCENQATISSK (bottom) of Alb labeled by probe **3ha**. NEM = *N*-ethylmaleimide.

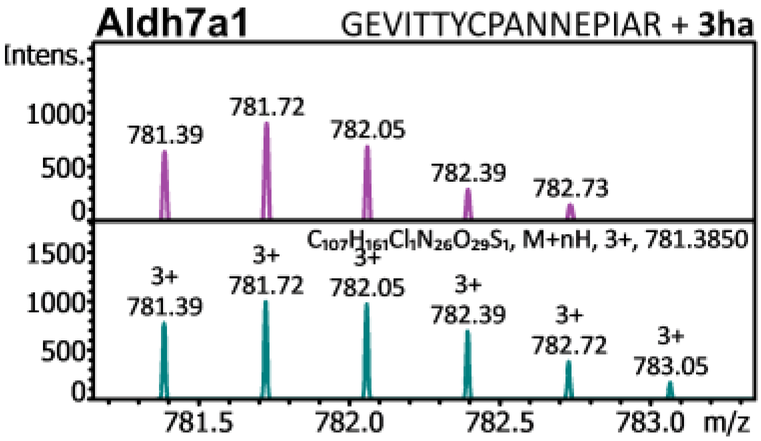

Figure S61. Measured (purple) and simulated (turquoise) isotope pattern of the peptide fragment GEVITTYCPANNEPIAR of Aldh7a1 labeled by probe **3ha**.

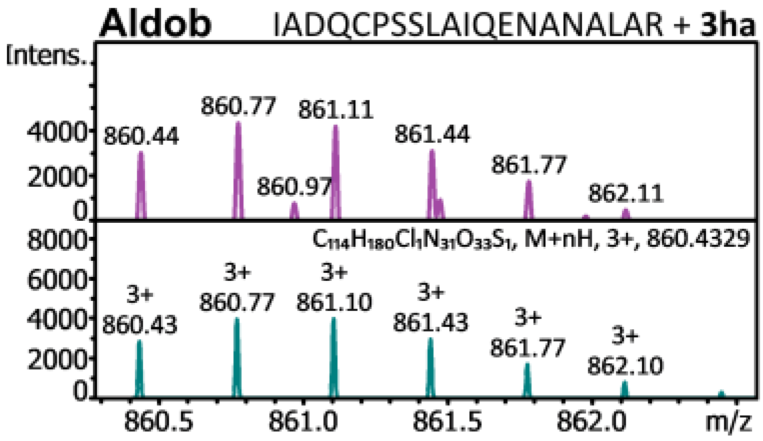

Figure S62. Measured (purple) and simulated (turquoise) isotope pattern of the peptide fragment IADQCPSSLAIQENANALAR of Aldob labeled by probe **3ha**.

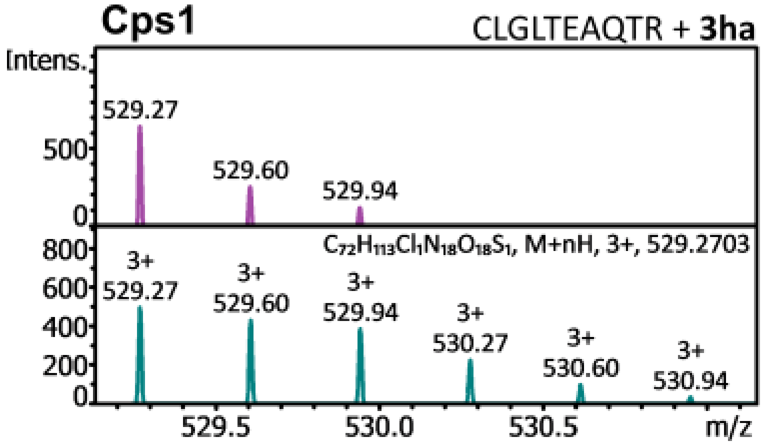

Figure S63. Measured (purple) and simulated (turquoise) isotope pattern of the peptide fragment CLGLTEAQTR of Cps1 labeled by probe **3ha**.

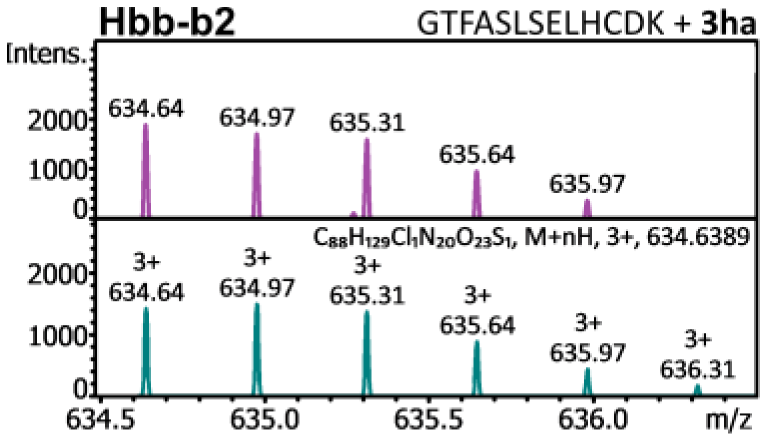

Figure 64. Measured (purple) and simulated (turquoise) isotope pattern of the peptide fragment GTFASLSELHCDK of Hbb-b2 labeled by probe **3ha**.

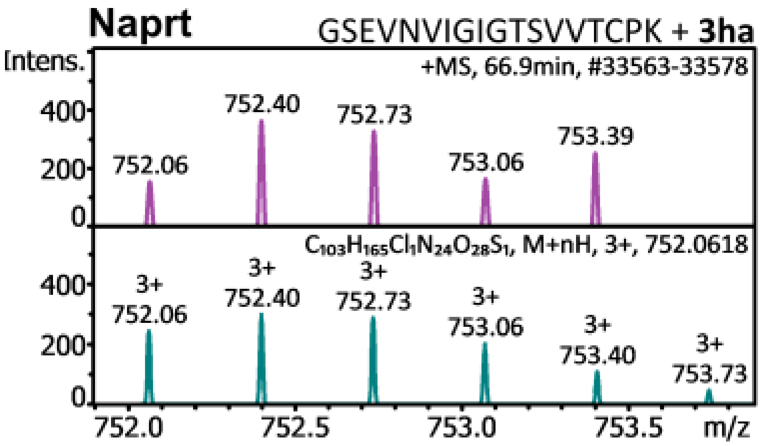

Figure S65. Measured (purple) and simulated (turquoise) isotope pattern of the peptide fragment GSEVNVIGIGTSVVTCPK of Naprt labeled by probe **3ha**.

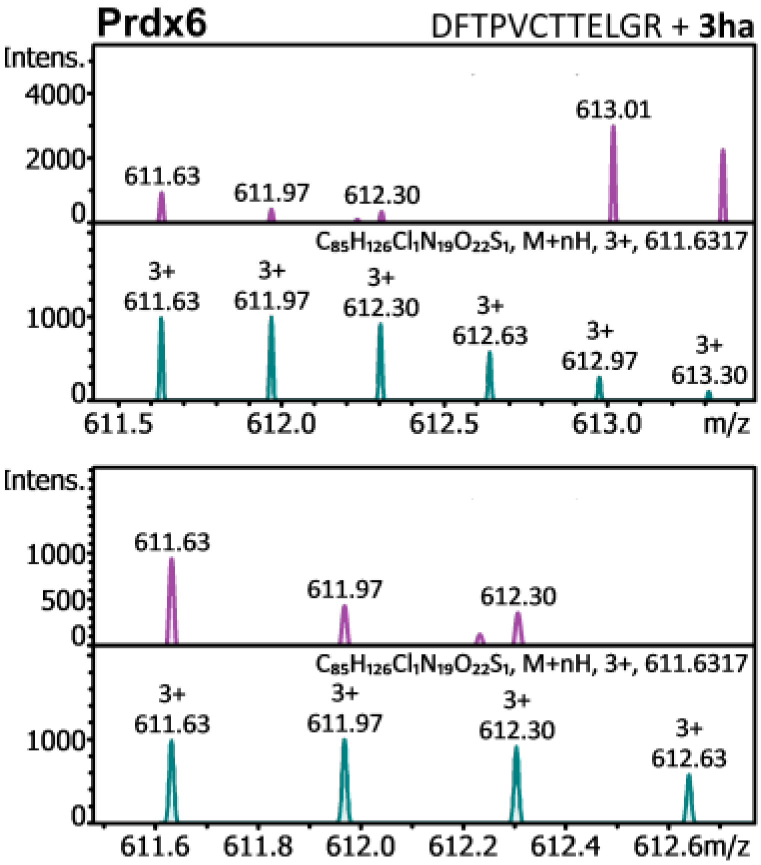

Figure S66. Measured (purple) and simulated (turquoise) isotope pattern of the peptide fragment DFTPVCTTELGR of Prdx6 labeled by probe **3ha**.

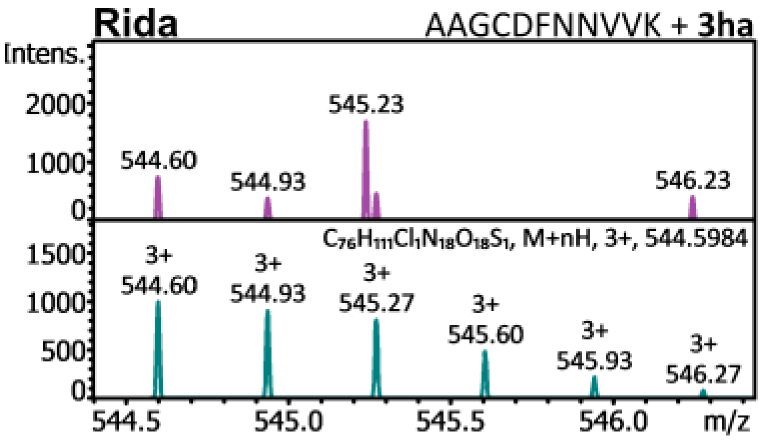

Figure S67. Measured (purple) and simulated (turquoise) isotope pattern of the peptide fragment AAGCDFNNVVK of Rida labeled by probe **3ha**.

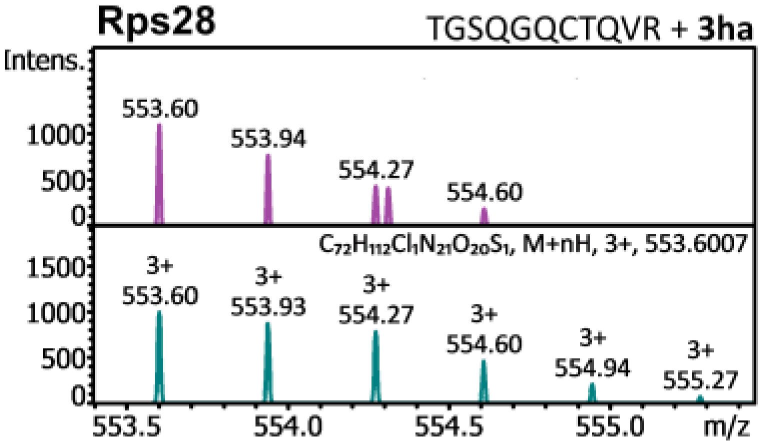

Figure S68. Measured (purple) and simulated (turquoise) isotope pattern of the peptide fragment TGSQGQCTQVR of Rps28 labeled by probe **3ha**.

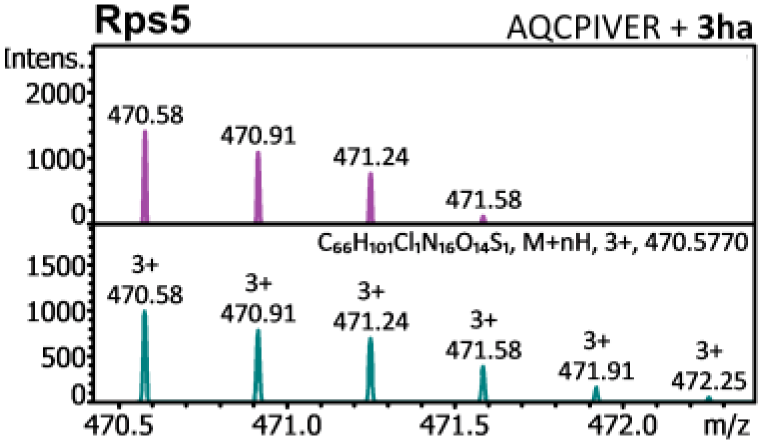

Figure S69. Measured (purple) and simulated (turquoise) isotope pattern of the peptide fragment AQCPIVER of Rps25 labeled by probe **3ha**.

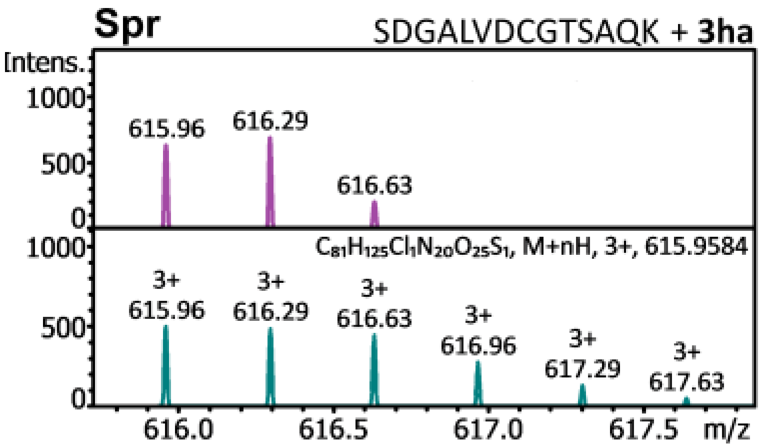

Figure S70. Measured (purple) and simulated (turquoise) isotope pattern of the peptide fragment SDGALVDCGTSAQK of Spr labeled by probe **3ha**.

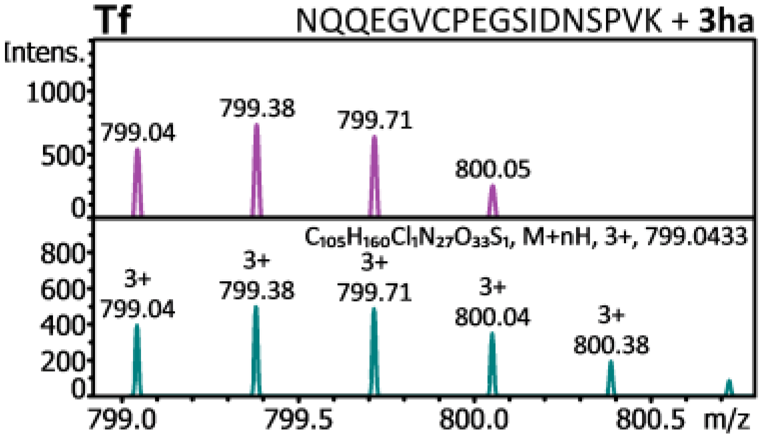

Figure S71. Measured (purple) and simulated (turquoise) isotope pattern of the peptide fragment NQQEGVCPEGSIDNSPVK of Tf labeled by probe **3ha**.

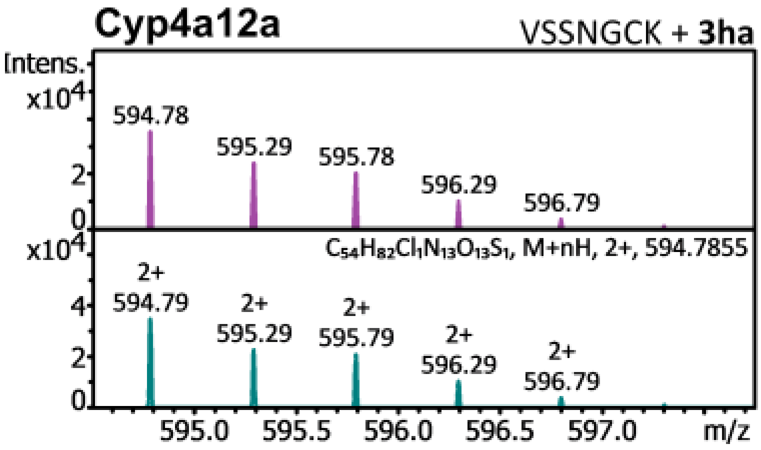

Figure S72. Measured (purple) and simulated (turquoise) isotope pattern of the peptide fragment VSSNGCK of Cyp4a12a labeled by probe **3ha**.

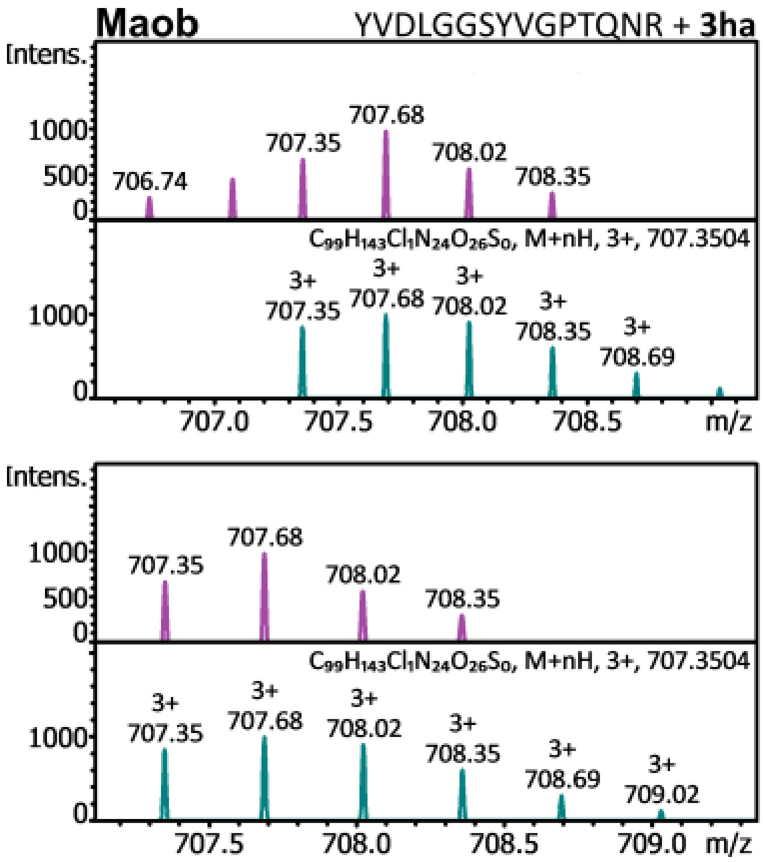

Figure S73. Measured (purple) and simulated (turquoise) isotope pattern of the peptide fragment YVDLGGSYVGPTQNR of Cyp4a12a labeled by probe **3ha**.

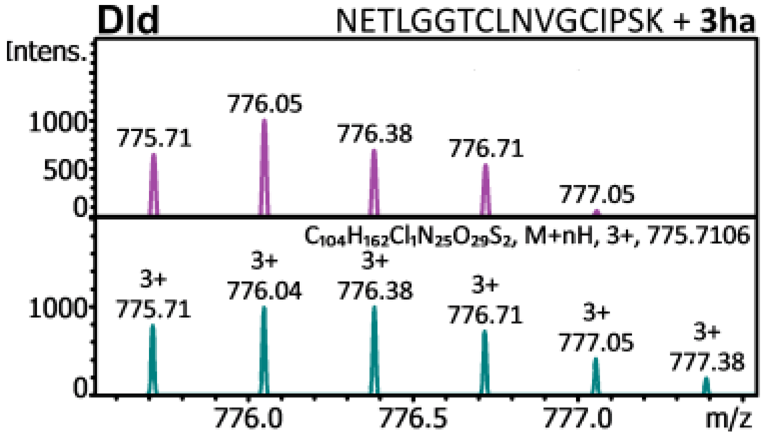

Figure S74. Measured (purple) and simulated (turquoise) isotope pattern of the peptide fragment NETLGGTCLNVGCIPSK of Dld labeled by probe **3ha**.

General Information

All commercially available chemicals and solvents were purchased from abcr, Acros Organics, Alfa Aesar, Fluka, Honeywell, Merck, Roth, Sigma Aldrich, TCI, Thermo Fisher Scientific, VWR and used without further purification, unless otherwise stated. Experiments were usually carried out under air with non-dry solvents unless otherwise mentioned. When applying Schlenk techniques the glass apparatus was dried under oil pump vacuum by heating with a heat gun, cooled to RT, and flushed with inert gas. In general, when high vacuum (*in vacuo*) was stated in experimental procedures, typically a vacuum of 10^{-2}-10^{-3} mbar was applied. Dry solvents were prepared by the below-mentioned procedures and afterwards stored under inert gas atmosphere (argon) over molecular sieves. In some cases, when explicitly mentioned, dry solvents were received from the listed suppliers. All reagents were added in a counter stream of inert gas to keep the inert atmosphere. All reactions were stirred with Teflon-coated magnetic stirring bars.

The stated temperatures generally refer to the oil bath or the cooling bath temperature. Temperatures were measured externally if not otherwise stated. When working at a temperature of 0 °C, an ice-water bath served as the cooling medium. Lower temperatures were achieved by using an acetone/dry ice cooling bath. Reactions, which were carried out at higher temperatures than rt, were heated in a silicon oil bath on a heating plate (RCT basic IKAMAG® safety control, 0-1500 rpm) equipped with an external temperature controller. The water bath temperature of the rotary evaporator was usually set to 40 °C unless otherwise noted.

Molecular sieves (Sigma-Aldrich, beads with 8-12 mesh) were activated in a round-bottom flask with a gas outlet adapter by heating them carefully in a heating mantle at level 1 at least for 24 h under high vacuum until complete dryness was obtained. These activated molecular sieves were stored at rt under argon atmosphere.

Dry Solvents

Dichloromethane (CH_2Cl_2): Dichloromethane (stabilized with EtOH) was purchased from Fisher Scientific, dried over phosphorus pentoxide, distilled and heated under reflux over CaH_2 for 24 h. It was distilled into an amber 1 L Schlenk bottle and stored over 4 Å molecular sieves under argon atmosphere.

<u>N,N-Dimethylformamide (DMF):</u> Anhydrous *N,N*-dimethylformamide was purchased from Sigma Aldrich in 99.8% purity. It was transferred into an amber 1 L Schlenk bottle and stored over activated 4 Å molecular sieves under argon atmosphere.

<u>Dimethylsulfoxide (DMSO):</u> Anhydrous dimethylsulfoxide was purchased from Sigma Aldrich in 99.9% purity in 1 L glass bottles and stored over 4 Å molecular sieves in a brown glass bottle under Ar atmosphere.

High Performance Liquid Chromatography with Mass Spectrometry (HPLC-MS)

Analytical HPLC-MS measurements were performed on an Agilent Technologies 1200 Series system (G1379 Degasser, G1312 Binary Pump, G1367C HiP ALS SL Autosampler, G1330B FC/ALS Thermostat, G1316B TCC SL column compartment, G1365C MWD SL multiple wavelength detector (deuterium lamp, 190-400 nm)) equipped with a single quadrupole LCMS detector “6120 LC/MS” using electrospray ionization source (ESI in positive and negative mode). Separations were carried out on a C-18-Reversed-Phase column of the type „Poroshell® 120 SB-C18, 3.0 x 100 mm, 2.7 µm“ by Agilent Technologies. Flow: Constant flow rate 0.7 mL/min, T = 35 °C. The following method was used:

MeCN_2_100: 0.0 – 0.1 min, isocratic, 2% MeCN (98% H_2O + 0.05% TFA); 0.1 – 8.0 min, linear, 2% to 100% MeCN (98% to 0% H_2O + 0.05% TFA); 8.0 – 11.1 min, isocratic, 100% MeCN; 11.1 – 11.3 min, linear, 100% to 2% MeCN (0% to 98% H_2O + 0.05 % TFA); 11.3 – 12.0 min, isocratic, 2% MeCN (98% H_2O + 0.05% TFA).

Gas Chromatography with Mass Spectrometry (GC-MS)

GC-MS analyses were performed on an Agilent Technologies 7890A GC system equipped with a 5975C mass selective detector (inert MSD with Triple Axis Detector system) by electron-impact ionization (EI) with a potential of E = 70 eV. Herein, the samples were separated depending on their boiling point and polarity. The desired crude materials or pure compounds were dissolved, and the solutions were injected by employing the autosampler 7683B in a split mode 1/20 (inlet temperature: 280 °C; injection volume: 0.2 µL). Separations were carried out on an Agilent Technologies J&W GC HP-5MS capillary column ((5%-phenyl)methylpolysiloxane, 30 m x 0.2 mm x 0.25 µm) with a constant helium flow rate (He

5.0 (Air Liquide), 1.085 mL·min-1, average velocity: 41.6 cm·s^{-1}). The following method was used:

MT_50_S: initial temperature: 50 °C for 1 min; linear increase to 300 °C (40 °C·min^{-1}); hold for 5 min; 1 min post-run at 300 °C; detecting range: 50.0-550.0 amu; solvent delay: 2.60 min.

Nuclear Magnetic Resonance Spectroscopy

NMR spectra were recorded on a Bruker Avance III 300 spectrometer (^{1}H: 300.36 MHz; ^{13}C: 75.53 MHz) with autosampler, or a Varian Unity Inova 500 spectrometer (^{1}H: 499.87 MHz; ^{13}C: 125.69 MHz; ^{19}F: 470.53 MHz), or a Jeol JNM-ECZL 400 MHz NMR Spectrometer (^{19}F: 376.17 MHz), or another Bruker Avance III 300 spectrometer (^{1}H: 300.13 MHz; ^{13}C: 75.47 MHz; ^{19}F: 282.40 MHz) with autosampler.

Chemical shifts δ are referenced to the residual proton and carbon signal of the deuterated solvent ($CDCl_3$: δ = 7.26 ppm (^{1}H), 77.16 ppm (^{13}C); CD_3OD: δ = 3.31 ppm (^{1}H), 49.00 ppm (^{13}C); DMSO-d_6: δ = 2.50 ppm (^{1}H), 39.52 ppm (^{13}C); D_2O: δ = 4.79 ppm (^{1}H)).[9] Chemical shifts δ are given in ppm (parts per million) and coupling constants *J* in Hz (Hertz). If necessary, 1D spectra (APT) as well as 2D spectra (H,H-COSY, HSQC, HMBC) were recorded for the identification and confirmation of the structure. Signal multiplicities are abbreviated as s (singlet), br s (broad singlet), d (doublet), t (triplet), q (quartet), quint (quintet), m (multiplet), dd (doublet of doublets), td (triplet of doublets), dt (doublet of triplets), and qd (quartet of doublets). Deuterated solvents for nuclear resonance spectroscopy were purchased from euriso-top®.

High Resolution Mass Spectrometry (HRMS)

High-resolution mass spectra (LC-ESI-MS/MS) were acquired by data-dependent high-resolution tandem mass spectrometry on a QExactive Focus (Thermo Fisher Scientific, Germany). The electrospray ionization potential was set to +3.5 or -3.0 kV, the sheath gas flow was set to 20, and an auxiliary gas flow of 5 was used. Samples were diluted with an appropriate solvent (methanol or chloroform) and 1 µL was injected on a SeQuant® ZIC®-pHILIC HPLC column (Merck, 100 x 2.1 mm; 5 µm; 100 Å; peek coated; equipped with a guard column) or on a RP-column (Waters, ACQUITY UPLC HSS T3 150 x 2.1 mm; 1.8 µm with VanGuard column). The separation solvent (pHILIC: A: CH_3CN, B: 25 mM NH_4HCO_3; RP: A: 0.1% HCOOH, B: 0.1% HCOOH in CH_3CN) was delivered through an Ultimate 3000 HPLC system

(Thermo Fisher Scientific, Germany) with a flow rate of 100 µL·min^{-1} and appropriate gradients were used for proper sample elution.

Determination of Melting Points

Melting points were determined on a Mel-Temp® melting point apparatus from Electrothermal with an integrated microscopical support. They were measured in open capillary tubes with a mercury-in-glass thermometer and were not corrected.

Thin Layer Chromatography

Analytical thin layer chromatography (TLC) was carried out on Merck TLC silica gel aluminum sheets (silica gel 60, F254, 20 x 20 cm). All separated compounds were visualized by UV light (λ = 254 nm and/or λ = 366 nm) and by the listed staining reagents followed by development in heat.

$KMnO_4$: 3.0 g $KMnO_4$ and 20 g K_2CO_3 were dissolved in 300 mL H_2O and afterwards 5.0 mL 5% aq. NaOH were added.

Flash Column Chromatography

Flash column chromatography was performed on silica gel 60 from Acros Organics with particle sizes between 35 µm and 70 µm. Depending on the problem of separation, a 30 to 100-fold excess of silica gel was used with respect to the dry amount of crude material. The dimension of the column was adjusted to the required amount of silica gel and formed a pad between 10 cm and 30 cm. In general, the silica gel was mixed with the eluent and the column was equilibrated. Subsequently, the crude material was dissolved in the eluent and loaded onto the top of the silica gel and the mobile phase was forced through the column using a rubber bulb pump. The volume of each collected fraction was adjusted between 20% and 30% of the silica gel volume.

X-ray Diffraction

For single crystal X-ray diffractometry all suitable crystals were covered with a layer of silicone oil. A single crystal was selected, mounted on a glass rod on a copper pin, and placed in the cold N_2 stream provided by an Oxford Cryosystems cryometer (T = 100 K), if not otherwise

stated. XRD data collection was performed on a Bruker APEX II diffractometer with use of Mo Kα radiation (λ = 0.71073 Å) from an IμS microsource and a CCD area detector. Empirical absorption corrections were applied using SADABS.[10] The structures were solved with use of either direct methods or the Patterson option in SHELXS. Structure refinement was carried out using SHELXL.[11] CIF files were edited, validated and formatted with the program OLEX2.[12] The space group assignments and structural solutions were evaluated using PLATON.[13] All non-hydrogen atoms were refined anisotropically. All hydrogen atoms were placed in calculated positions corresponding to standard bond lengths and angles using riding models.

Experimental Procedures

Overview

▪ Synthesis of Azidoamine Linkers

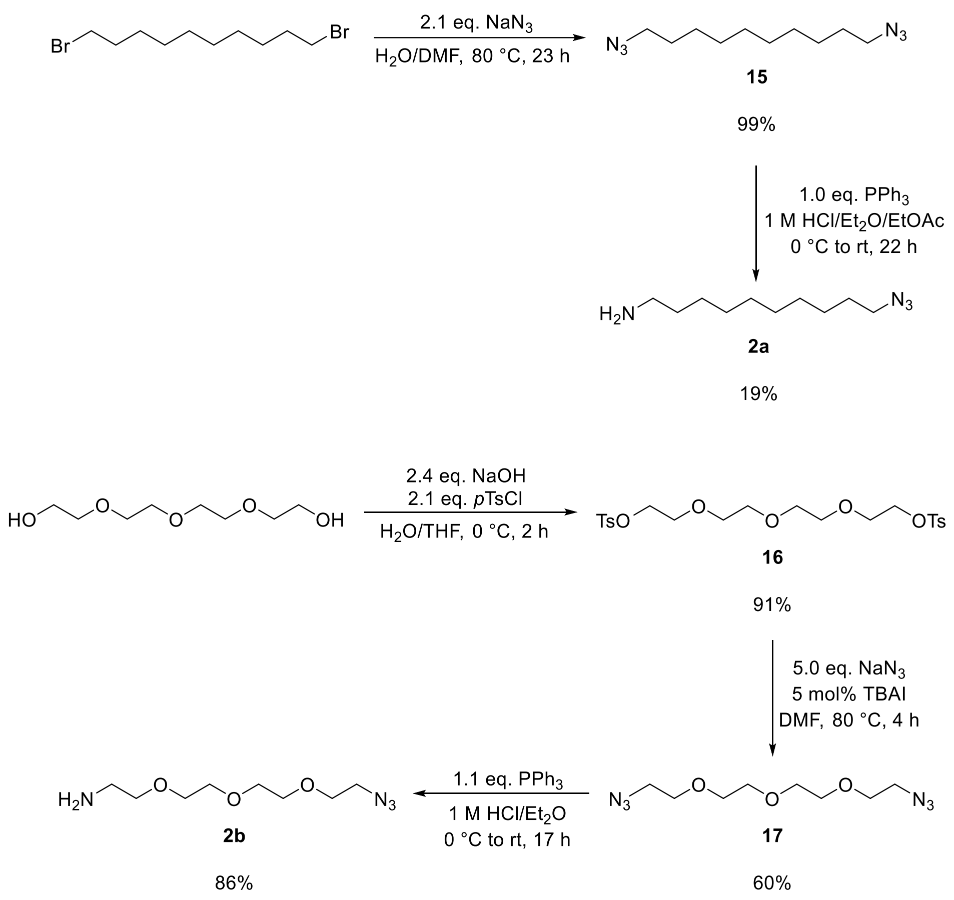

▪ **Synthesis of Biphenylcarboxylic Acids**

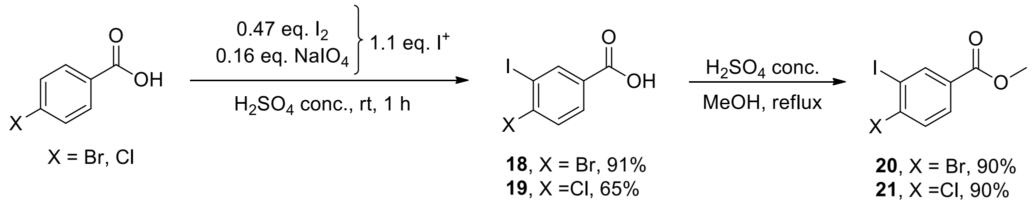

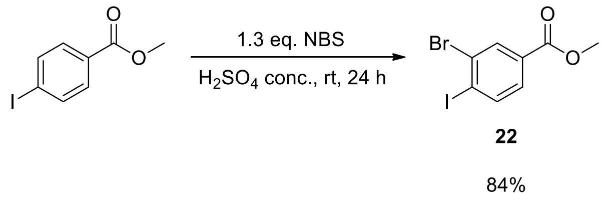

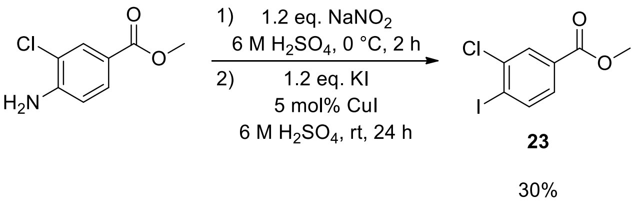

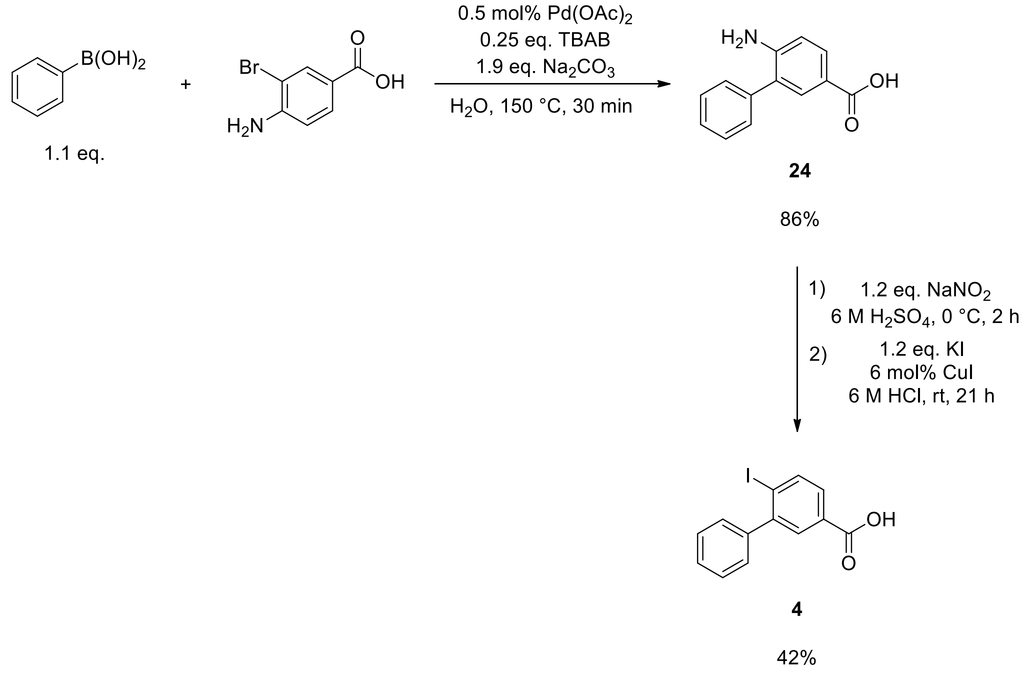

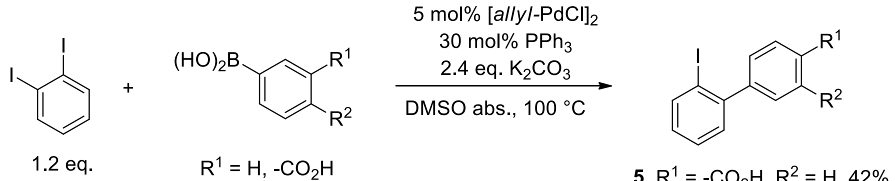

R^1 = H, $-CO_2H$
R^2 = H, $-CO_2H$

5, R^1 = $-CO_2H$, R^2 = H, 42%
6, R^1 = H, R^2 = $-CO_2H$, 45%

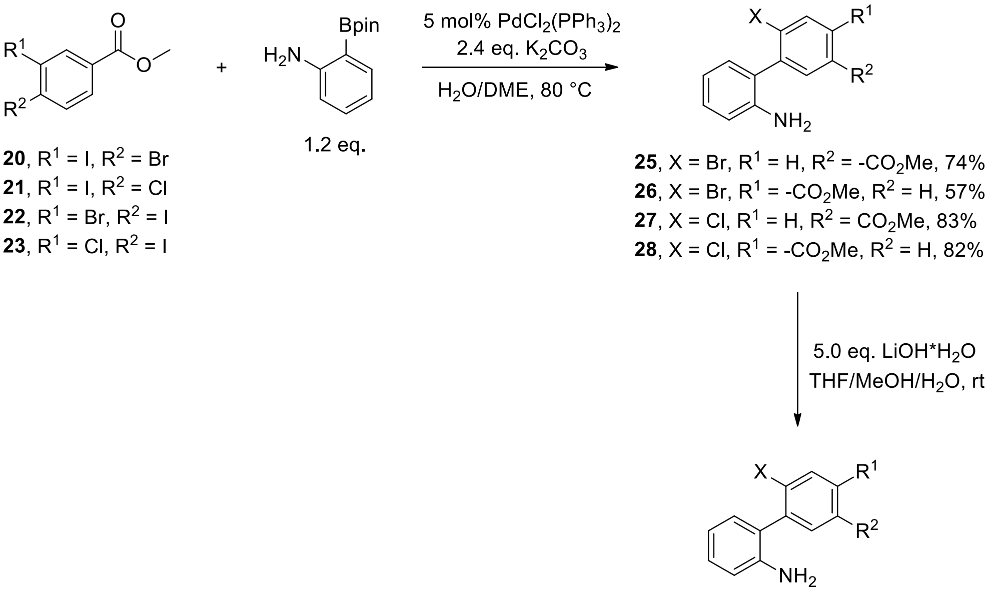

20, R^1 = I, R^2 = Br
21, R^1 = I, R^2 = Cl
22, R^1 = Br, R^2 = I
23, R^1 = Cl, R^2 = I

25, X = Br, R^1 = H, R^2 = $-CO_2Me$, 74%
26, X = Br, R^1 = $-CO_2Me$, R^2 = H, 57%
27, X = Cl, R^1 = H, R^2 = CO_2Me, 83%
28, X = Cl, R^1 = $-CO_2Me$, R^2 = H, 82%

7, X = Br, R^1 = H, R^2 = $-CO_2H$, >99%
8, X = Br, R^1 = $-CO_2H$, R^2 = H, 99%
9, X = Cl, R^1 = H, R^2 = CO_2H, 97%
10, X = Cl, R^1 = $-CO_2H$, R^2 = H, 75%

▪ Synthesis of Acyclic Diaryliodonium Salts

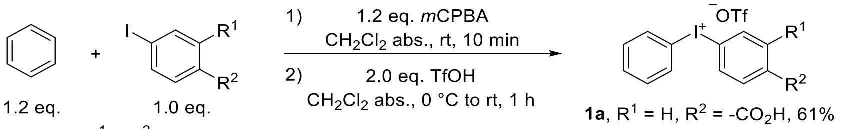

R^1 = R^2 = H or $-CO_2H$

1a, R^1 = H, R^2 = $-CO_2H$, 61%
1b, R^1 = -COOH, R^2 = H, 46%

▪ Synthesis of Cyclic Diaryliodonium Salts

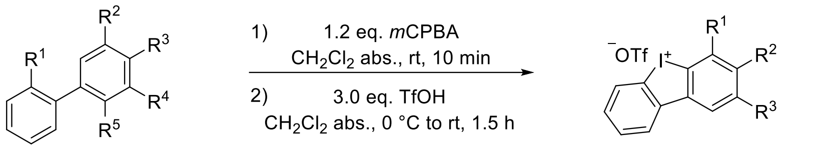

4, $R^1 = R^3 = R^4 = H$, $R^2 = -CO_2H$, $R^5 = I$
5, $R^1 = I$, $R^2 = R^4 = R^5 = H$, $R^3 = -CO_2H$
6, $R^1 = I$, $R^2 = R^3 = R^5 = H$, $R^4 = -CO_2H$

1c, $R^1 = R^2 = H$, $R^3 = -CO_2H$, 76%
1d, $R^1 = R^3 = H$, $R^2 = -CO_2H$, 74%
1e, $R^1 = -CO_2H$, $R^2 = R^3 = H$, 84%

▪ Synthesis of Cyclic Diarylbromonium and Diarylchloronium Salts

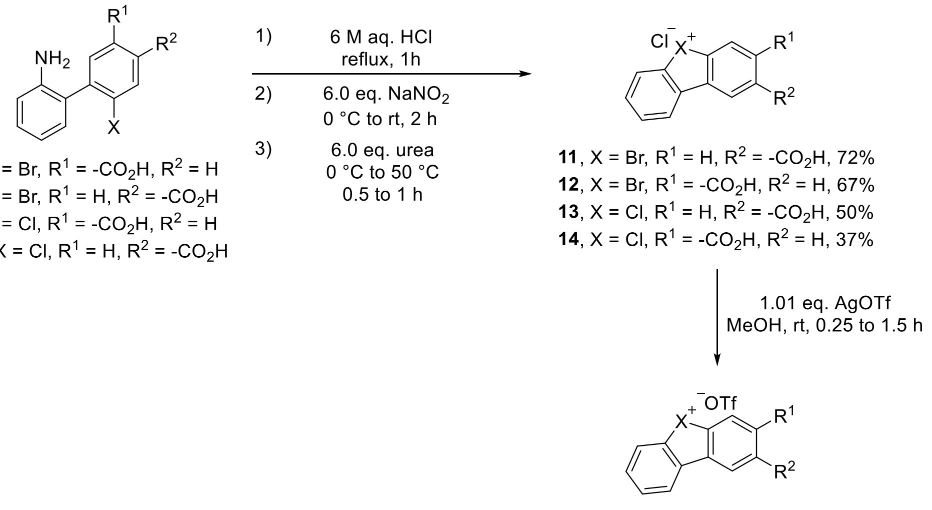

7, X = Br, $R^1 = -CO_2H$, $R^2 = H$
8, X = Br, $R^1 = H$, $R^2 = -CO_2H$
9, X = Cl, $R^1 = -CO_2H$, $R^2 = H$
10, X = Cl, $R^1 = H$, $R^2 = -CO_2H$

11, X = Br, $R^1 = H$, $R^2 = -CO_2H$, 72%
12, X = Br, $R^1 = -CO_2H$, $R^2 = H$, 67%
13, X = Cl, $R^1 = H$, $R^2 = -CO_2H$, 50%
14, X = Cl, $R^1 = -CO_2H$, $R^2 = H$, 37%

1f, X = Br, $R^1 = H$, $R^2 = -CO_2H$, 83%
1g, X = Br, $R^1 = -CO_2H$, $R^2 = H$, 85%
1h, X = Cl, $R^1 = H$, $R^2 = -CO_2H$, 81%
1i, X = Cl, $R^1 = -CO_2H$, $R^2 = H$, >99%

▪ Synthesis of Activity-based Probes

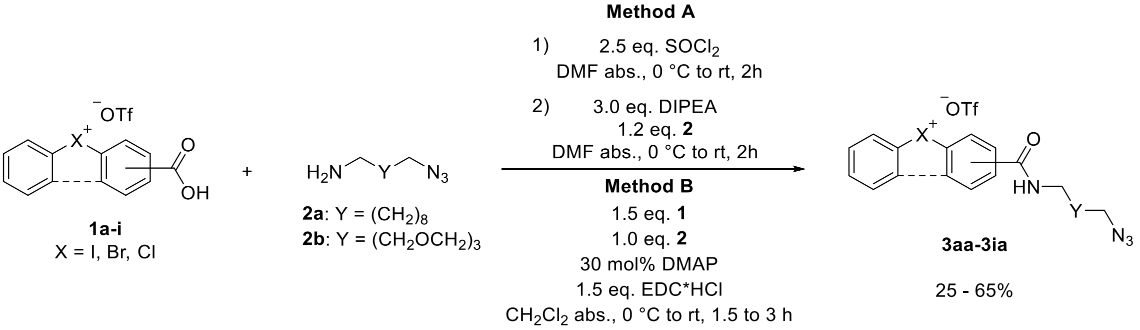

1a-i
X = I, Br, Cl

2a: Y = $(CH_2)_8$
2b: Y = $(CH_2OCH_2)_3$

3aa-3ia
25 - 65%

▪ Synthesis of Methyl *tert*-butacrylate (*t*-BAM)

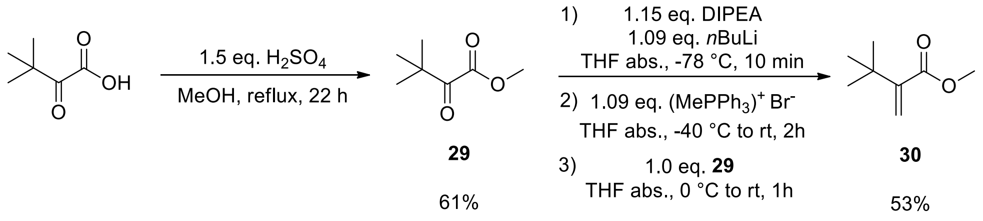

Synthesis of Azidoamine Linkers

1,10-Diazidodecane (15)

This compound was prepared according to a procedure described by Roe et al.[14]

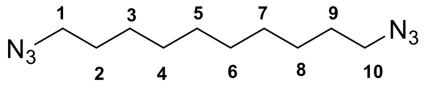

15

In a 100 mL round-bottom flask 1,10-dibromodecane (11.0 g, 36.7 mmol) was dissolved in 50 mL DMF. NaN_3 (5.00 g, 77.0 mmol) was dissolved in 25 mL H_2O and added to the DMF solution. The reaction mixture was heated to 80 °C for 23 h. After cooling to rt, the reaction mixture was taken up in 150 mL brine and the product was extracted with CH_2Cl_2 (4 x 100 mL). The combined organic layers were dried over Na_2SO_4, filtered, and concentrated under reduced pressure.

Yield 8.21 g (36.6 mmol, 99%), colorless liquid, $C_{10}H_{20}N_6$ [224.31]

^{1}H NMR (300.36 MHz, $CDCl_3$) δ = 3.25 (t, $^3J_{H,H}$ = 6.9 Hz, 4H, H-1, H-10), 1.66 – 1.52 (m, 4H, H-2, H-9), 1.30 (s, 12H, H-3 to H-8) ppm.

^{13}C NMR (75.53 MHz, $CDCl_3$) δ = 51.6 (CH_2, C-1, C-10), 29.5 (CH_2, C-2, C-9), 29.2 (CH_2, C-5, C-6), 29.0 (CH_2, C-4, C-7), 26.8 (CH_2, C-3, C-8) ppm.

The recorded spectra are in accordance with the reported in literature.[14]

10-Azidodecan-1-amine (2a)

This compound was prepared according to a procedure described by Roe et al.[14]

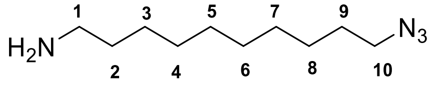

2a

In a 100 mL round-bottom flask 1,10-diazidodecane (**15**) (3.38 g, 15.0 mmol) was dissolved in 20 mL Et_2O/EtOAc (1/1, v/v). 30 mL 1 M HCl were added and the reaction mixture was cooled to 0 °C via an ice/water bath. PPh_3 (3.95 g, 15.1 mmol) was added in small portions over a period of 3 h at 0 °C. The reaction mixture was allowed to warm to rt and was stirred for 22 h

at rt. After separation of the layers, the aqueous phase was washed with Et_2O (3 x 20 mL), basified to pH 12 with 3 M NaOH, and subsequently extracted with CH_2Cl_2 (3 x 50 mL). The combined organic layers were dried over Na_2SO_4, filtered, and concentrated under reduced pressure. The crude, colourless oil was purified *via* flash column chromatography (100 mL silica gel, 9 x 4 cm, CH_2Cl_2/MeOH/NH_3 = 100/20/1, fraction size: 20 mL).

Yield 569 mg (2.87 mmol, 19%), pale yellow liquid, $C_{10}H_{22}N_4$ [198.31]

TLC R_f = 0.17 (CH_2Cl_2/MeOH/NH_3 = 100/20/1; UV and ninhydrin)

^{1}H NMR (300.36 MHz, $CDCl_3$) δ = 3.23 (t, $^3J_{H,H}$ = 6.9 Hz, 2H, H-10), 2.69 (t, $^3J_{H,H}$ = 7.1 Hz, 2H, H-1), 2.43 (s, 2H, N-H), 1.63 – 1.51 (m, 2H, H-9), 1.50 – 1.38 (m, 2H, H-2), 1.27 (s, 12H, H-3 to H-8) ppm.

^{13}C NMR (75.53 MHz, $CDCl_3$) δ = 51.6 (CH_2, C-10), 42.7 (CH_2, C-1), 33.1 (CH_2, C-2), 29.6 (CH_2), 29.5 (CH_2), 29.5 (CH_2), 29.2 (CH_2), 28.9 (CH_2), 26.9 (CH_2), 26.8 (CH_2) ppm.

The recorded spectra are in accordance with the reported in literature.[14]

Tetraethylene glycol di(*p*-toluenesulfonate) (16)

This compound was prepared according to a procedure described by Jain et al.[15]

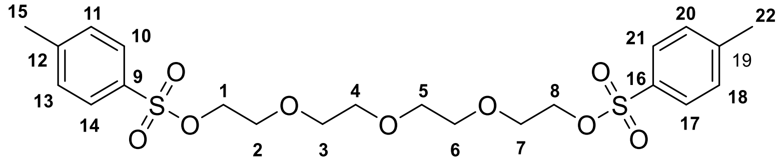

16

In a 250 mL round-bottom flask a solution of NaOH (1.49 g, 37.3 mmol) in 9 mL H_2O was added to a mixture of tetraethylene glycol (3.05 g, 15.7 mmol) in 5 mL THF. After cooling to 0 °C, a solution of *p*-toluenesulfonyl chloride (6.16 g, 32.3 mmol) in 35 mL THF was added dropwise over 1 h at 0 °C and the yellowish reaction solution was then stirred at 0 °C for 1 h until full conversion was detected by TLC (cyclohexane/EtOAc = 2/3; UV). The reaction solution was then diluted with 20 mL ice water and extracted with CH_2Cl_2 (3 x 50 mL). The combined organic layers were washed with brine (1 x 100 mL), dried over Na_2SO_4, filtered and the solvent was removed under reduced pressure. The crude product was used without further purification.

Yield 7.21 g (14.3 mmol, 91%), pale yellow liquid, $C_{22}H_{30}O_9S_2$ [502.59]

TLC R_f = 0.47 (cyclohexane/EtOAc = 2/3; UV)

^{1}H NMR (300.36 MHz, $CDCl_3$) δ = 7.78 (d, $^3J_{H,H}$ = 8.2 Hz, 4H, H-10, H-14, H-17, H-21), 7.33 (d, $^3J_{H,H}$ = 8.0 Hz, 4H, H-11, H-13, H-18, H-20), 4.19 – 4.10 (m, 4H), 3.66 (m, 4H), 3.56 (s, 8H), 2.44 (s, 6H, H-15, H-22) ppm.

^{13}C NMR (75.53 MHz, $CDCl_3$) δ = 144.9 (C_q, C-9, C-16), 133.1 (C_q, C-12, C-19), 130.0 (CH, C-11, C-13, C-18, C-20), 128.1 (CH, C-10, C-14, C-17, C-21), 70.9 (CH_2), 70.7 (CH_2), 69.4 (CH_2), 68.8 (CH_2), 21.8 (CH_3, C-15, C-22) ppm.

The recorded spectra are in accordance with the reported in literature.[15]

1-Azido-2-(2-(2-(2-azidoethoxy)ethoxy)ethoxy)ethane (17)

This compound was prepared according to a procedure described by Longo et al.[16]

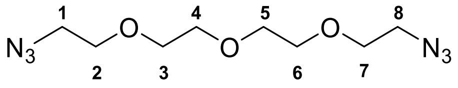

17

In a 100 mL round-bottom flask tetraethylene glycol di(*p*-toluenesulfonate) (**16**) (5.00 g, 9.96 mmol) was dissolved in 30 mL DMF. NaN_3 (3.24 g, 49.8 mmol) and TBAI (180 mg, 0.487 mmol) were added and the yellow suspension was stirred at 80 °C for 4 h until quantitative conversion was detected by TLC (cyclohexane/EtOAc = 2/3; UV + $KMnO_4$). After cooling to rt, the reaction solution was taken up in 150 mL brine and extracted with EtOAc (3 x 50 mL). The combined organic layers were washed with sat. NH_4Cl solution (1 x 50 mL), dried over Na_2SO_4, filtered and the solvent was removed under reduced pressure. The crude product was purified via column chromatography (100 mL silica gel, cyclohexane/EtOAc = 1/1, fraction size: 20 mL).

Yield 1.46 g (5.96 mmol, 60%), orange liquid, $C_8H_{16}N_6O_3$ [244.26]

TLC R_f = 0.60 (cyclohexane/EtOAc = 1/2; $KMnO_4$)

^{1}H NMR (300.36 MHz, $CDCl_3$) δ = 3.73 – 3.59 (m, 12H, H-2 to H-7), 3.45 – 3.32 (m, 4H, H-1, H-8) ppm.

^{13}C NMR (75.53 MHz, $CDCl_3$) δ = 70.8 (CH_2), 70.2 (CH_2), 50.8 (CH_2, C-1, C-8) ppm.

The recorded spectra are in accordance with the reported in literature.[17]

2-(2-(2-(2-Azidoethoxy)ethoxy)ethoxy)ethan-1-amine (2b)

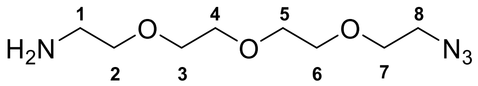

2b

In a 100 mL round-bottom flask 1-azido-2-(2-(2-(2-azidoethoxy)ethoxy)ethoxy)ethane (**17**) (1.01 g, 4.13 mmol) was dissolved in 16 mL 1 M aq. HCl. After cooling to 0 °C via an ice/water bath, a solution of PPh_3 (1.18 g, 4.48 mmol) in 16 mL Et_2O was added dropwise over 1 h. The ice/water bath was then removed, and the colorless reaction solution was stirred for 17 h at rt. After separation of the layers, the aqueous phase was washed with Et_2O (2 x 10 mL), basified to pH 12 with 2 M NaOH, and subsequently extracted with CH_2Cl_2 (4 x 50 mL). The combined organic layers were dried over Na_2SO_4, filtered, and concentrated under reduced pressure. The crude product was purified via column chromatography (50 mL silica gel, 10 x 2.5 cm, CH_2Cl_2/MeOH/NH_3 = 10/2/0.1, fraction size: 10 mL).

Yield 768 mg (3.52 mmol, 86%), pale yellow liquid, $C_8H_{18}N_4O_3$ [218.26]

TLC R_f = 0.23 (CH_2Cl_2/MeOH/NH_3 = 10/2/0.1; ninhydrin)

^{1}H NMR (300.36 MHz, $CDCl_3$) δ = 3.71 – 3.58 (m, 10H), 3.50 (t, $^3J_{H,H}$ = 5.2 Hz, 2H), 3.41 – 3.34 (m, 2H, H-8), 2.86 (t, $^3J_{H,H}$ = 5.2 Hz, 2H, H-1), 1.74 (s, 2H, N-H) ppm.

^{13}C NMR (75.53 MHz, $CDCl_3$) δ = 71.2 (CH_2), 70.7 (CH_2), 70.7 (CH_2), 70.7 (CH_2), 70.3 (CH_2), 70.1 (CH_2), 50.9 (CH_2, C-8), 41.2 (CH_2, C-1) ppm.

The recorded spectra are in accordance with the reported in literature.[17]

Synthesis of Biphenylcarboxylic Acids

General Procedure for the 3-Iodination of 4-Halobenzoic acids

According to a literature procedure from Kraszkiewicz et al.[18]

In a 100 mL round-bottom flask I_2 (4.73 mmol) and $NaIO_4$ (1.59 mmol, 1.1 eq of total reactive I^+-species) were added to 30 mL 96% (w/w) H_2SO_4 and the reaction mixture was stirred for 30 min at rt until a dark brown solution was obtained. The corresponding 4-halobenzoic acid

(10.0 mmol) was added and the dark brown suspension was stirred for 1 h at rt. The reaction mixture was then poured onto 300 mL ice water and the product was collected via filtration, washed with H_2O (6 x 100 mL), and dried via suction on the frit. The crude product was suspended in 50 mL CH_2Cl_2 and collected via filtration. This process was repeated five times to remove remaining I_2. The final product was eventually dried under high vacuum.

4-Bromo-3-iodobenzoic acid (18)

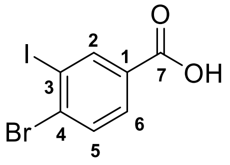

18

Starting from 4-bromobenzoic acid (2.01 g, 10.0 mmol) iodination was executed as described in general procedure.

Yield 2.99 g (9.15 mmol, 91%), pale pink solid, $C_7H_4BrIO_2$ [326.92]

M.p. 235 - 239 °C

^{1}H NMR (300.36 MHz, DMSO-d6) δ = 8.36 (s, 1H, H-2), 7.83 (m, $^3J_{H,H}$ = 8.2 Hz, 2H, H-5, H-6) ppm.

^{13}C NMR (75.53 MHz, DMSO-d6) δ = 165.44 (C_q, C-7), 140.5 (CH, C-2), 134.2 (C_q, C-4), 133.0 (CH, C-6), 131.4 (C_q, C-1), 130.4 (CH, C-5), 102.2 (C_q, C-3) ppm.

4-Chloro-3-iodobenzoic acid (19)

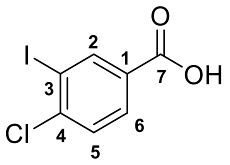

19

Starting from 4-chlorobenzoic acid (1.57 g, 10.0 mmol) iodination was executed as described in general procedure.

Yield 1.83 g (6.46 mmol, 65%), pale pink solid, $C_7H_4ClIO_2$ [282.46]

M.p. 205 – 215 °C

^{1}H NMR (300.36 MHz, DMSO-d6) δ = 8.38 (s, 1H, H-2), 7.91 (d, $^3J_{H,H}$ = 7.5 Hz, 1H, H-6), 7.69 (d, $^3J_{H,H}$ = 8.1 Hz, 1H, H-5) ppm.

^{13}C NMR (75.53 MHz, DMSO-d6) δ = 165.2 (C_q, C-7), 142.0 (C_q, C-4), 140.6 (CH, C-2), 130.9 (C_q, C-1), 130.5 (CH, C-6), 129.6 (CH, C-5), 99.0 (C_q, C-3) ppm.

Methyl 4-bromo-3-iodobenzoate (20)

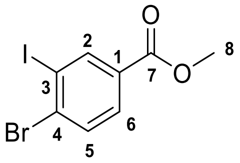

20

In a 100 mL round-bottom flask equipped with a reflux condenser 4-bromo-3-iodobenzoic acid (**18**) (1.18 g, 3.61 mmol) was suspended in 30 mL methanol. H_2SO_4 conc. (400 µL) was added and the reaction mixture was heated for 7 h under reflux until full conversion was detected by TLC. The yellow solution was cooled to rt, diluted with 20 mL H_2O and extracted with EtOAc (3 x 50 mL). The combined organic layers were washed with brine (1 x 100 mL), dried over Na_2SO_4, filtered, and the solvent was removed under reduced pressure. The crude product was purified *via* silica filtration (25 mL silica gel, CH_2Cl_2/MeOH = 10/1).

Yield 1.11 g (3.26 mmol, 90%), colorless solid, $C_8H_6BrIO_2$ [340.94]

TLC R_f = 0.63 (cyclohexane/EtOAc = 4/1; UV + $KMnO_4$)

M.p. 65 - 66 °C

^{1}H NMR (300.36 MHz, DMSO-d6) δ = 8.37 (d, $^4J_{H,H}$ = 1.8 Hz, 1H, H-2), 7.84 (m, $^3J_{H,H}$ = 8.3, 5.0 Hz, 2H, H-5, H-6), 3.85 (s, 3H, H-8) ppm.

^{13}C NMR (75.53 MHz, DMSO-d6) δ = 164.4 (C_q, C-7), 140.2 (CH, C-2), 134.7 (C_q, C-4), 133.1 (CH, C-6), 130.2 (CH, C-5), 130.0 (C_q, C-1), 102.5 (C_q, C-3), 52.6 (CH_3, C-8) ppm.

The recorded spectra are in accordance with the reported in literature.[19]

Methyl 4-chloro-3-iodobenzoate (21)

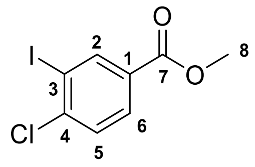

21

In a 50 mL round-bottom flask 4-chloro-3-iodobenzoic acid (**19**) (460 mg, 1.63 mmol) was suspended in 15 mL methanol. H_2SO_4 conc. (177 µL, 2.0 eq.) was added and the reaction mixture was heated for 4 h under reflux until full conversion was detected by HPLC. The colorless solution was cooled to rt, diluted with 15 mL H_2O and extracted with EtOAc (3 x 15 mL). The combined organic layers were washed with brine (1 x 30 mL), dried over Na_2SO_4, filtered, and the solvent was removed under reduced pressure. The crude product was purified via silica filtration (25 mL silica gel, cyclohexane/EtOAc = 3/1).

Yield 435 mg (1.47 mmol, 90%), colorless solid, $C_8H_6ClIO_2$ [296.49]

TLC R_f = 0.69 (cyclohexane/EtOAc = 3/1; UV + $KMnO_4$)

HPLC-MS t_R = 6.94 min (method: *2-100-MeCN*); *m/z* (ESI+) = 297 $[M+H]^+$

M.p. 48 – 50 °C

^{1}H NMR (300.36 MHz, $CDCl_3$) δ = 8.51 (d, $^4J_{H,H}$ = 1.8 Hz, 1H, H-2), 7.94 (dd, $^3J_{H,H}$ = 8.3, $^4J_{H,H}$ = 1.8 Hz, 1H, H-6), 7.51 (d, $^3J_{H,H}$ = 8.3 Hz, 1H, H-5), 3.92 (s, 3H, H-8) ppm.

^{13}C NMR (75.53 MHz, $CDCl_3$) δ = 165.0 (C_q, C-7), 143.7 (C_q, C-4), 141.5 (CH, C-2), 130.5 (CH, C-6), 130.0 (C_q, C-1), 129.4 (CH, C-5), 98.0 (C_q, C-3), 52.7 (CH_3, C-8) ppm.

The recorded spectra are in accordance with the reported in literature.[20]

Methyl 3-bromo-4-iodobenzoate (22)

This compound was prepared according to a procedure described by Vu et al.[21]

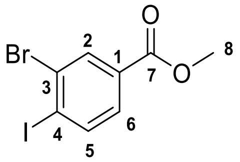

22

In a 50 mL round-bottom flask methyl 4-iodobenzoate (5.00 g, 19.1 mmol) was dissolved in 25 mL H_2SO_4 conc. *N*-Bromosuccinimide (4.37 g, 24.6 mmol) was added in one portion and the reaction mixture was stirred for 24 h at rt until full conversion was detected by HPLC. The orange solution was poured onto 200 mL ice-cold water and extracted with EtOAc (3 x 30 mL). The combined organic layers were washed with sat. $NaHCO_3$ solution (1 x 50 mL) and brine (1 x 50 mL), dried over Na_2SO_4 and the solvent was removed under reduced pressure. The product was directly used without further purification.

Yield 5.48 g (16.1 mmol, 84%), pale pink solid, $C_8H_6BrIO_2$ [340.94]

^{1}H NMR (300.36 MHz, DMSO-d6) δ = 8.15 – 8.05 (m, 2H, H-2, H-5), 7.61 (dd, $^3J_{HH}$ = 8.1 Hz, $^4J_{HH}$ = 1.8 Hz, 1H, H-6), 3.85 (s, 3H, H-8) ppm.

^{13}C NMR (75.53 MHz, DMSO-d6) δ = 164.7 (C_q, C-7), 140.8 (CH, C-5), 132.4 (CH, C-2), 131.1 (C_q, C-3), 129.5 (C_q, C-1), 129.0 (CH, C-6), 109.1 (C_q, C-4), 52.6 (CH_3, C-8) ppm.

The recorded spectra are in accordance with the reported in literature.[22]

Methyl 3-chloro-4-iodobenzoate (23)

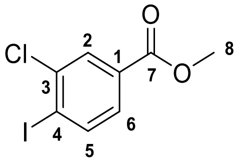

23

In a 250 mL round-botom flask methyl 4-amino-3-chlorobenzoate (1.01 g, 5.38 mmol) was suspended in 50 mL 6 M aq. H_2SO_4. After stirring the yellowish reaction mixture for 1 h at rt, it was cooled to 0 °C via an ice/water bath and a solution of $NaNO_2$ (443 mg, 6.42 mmol) in 2.5 mL H_2O was added and stirred for 1 h at 0 °C. Subsequently, CuI (51.8 mg, 0.272 mmol)

and KI (1.07 g, 6.46 mmol) were added and the dark brown suspension was stirred for 24 h at rt. The solid was collected by filtration through a sintered glass frit and washed with H_2O (3 x 50 mL). Subsequently, the solid was dissolved in 100 mL EtOAc and the organic layer was washed with sat. $NaHCO_3$ solution (1 x 50 mL) and brine (1 x 100 mL), dried over $NaSO_4$, filtered, and concentrated under reduced pressure. The crude, dark-brown oil was purified via column chromatography (70 mL silica gel, 10 x 3 cm, cyclohexane/EtOAc = 10/1, fraction size: 15 mL).

Yield 491 mg (1.66 mmol, 30%), colorless solid, $C_8H_6ClIO_2$ [296.49]

TLC R_f = 0.53 (cyclohexane/EtOAc = 10/1; UV + $KMnO_4$)

M.p. 68 – 70 °C

^{1}H NMR (300.36 MHz, $CDCl_3$) δ = 8.07 (s, 1H, H-6), 7.95 (d, $^3J_{HH}$ = 8.2 Hz, 1H, H-4), 7.58 (d, $^3J_{HH}$ = 10.0 Hz, 1H, H-3), 3.92 (s, 3H, H-8) ppm.

^{13}C NMR (75.53 MHz, $CDCl_3$) δ = 165.7 (C_q, C-7), 140.6 (CH, C-3), 139.2 (C_q, C-1), 131.8 (C_q, C-5), 130.1 (CH, C-6), 128.6 (CH, C-4), 104.6 (C_q, C-2), 52.7 (CH_3, C-8) ppm.

The recorded spectra are in accordance with the reported in literature.[23]

2'-Iodo-[1,1'-biphenyl]-4-carboxylic acid (5)

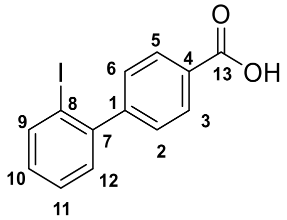

5

In a flame-dried and argon-flushed 80 mL Schlenk flask 4-carboxyphenylboronic acid (630 mg, 3.80 mmol), allylpalladium(II) chloride dimer (70.9 mg, 0.194 mmol) and PPh_3 (302 mg, 1.15 mmol) were dissolved in 30 mL abs. DMSO. 1,2-Diiodobenzene (600 µL, 1.51 g, 4.59 mmol) and K_2CO_3 (1.27 g, 9.15 mmol) were added to the yellow suspension and heated to 100 °C for 66 h. After cooling to rt, the reaction mixture was quenched by the addition of 100 mL 6 M HCl and the product was extracted with CH_2Cl_2 (3 x 100 mL). The combined organic layers were washed with 6 M HCl (2 x 100 mL), H_2O (1 x 100 mL), and sat. $Na_2S_2O_3$

solution (2 x 100 mL) and dried over Na_2SO_4. After filtration, the solvent was removed under reduced pressure. The crude, orange-brown solid was adsorbed onto 2 g silica gel and purified *via* column chromatography (250 mL silica gel, 13 x 5 cm, cyclohexane/EtOAc = 4/1 to 2/1, fraction size: 50 mL).

Yield 515 mg (1.59 mmol, 42%), pale orange solid, $C_{13}H_9IO_2$ [324.12]

TLC R_f = 0.23 (cyclohexane/EtOAc = 1/1; UV)

M.p. 235 - 242 °C decomp.

^{1}H NMR (300.36 MHz, DMSO-d6) δ = 13.04 (s, 1H, O-H), 8.08 – 7.92 (m, 3H, H-aromatic), 7.54 – 7.41 (m, 3H, H-aromatic), 7.35 (dd, $^3J_{H,H}$ = 7.6 Hz, $^4J_{H,H}$ = 1.5 Hz, 1H, H-aromatic), 7.16 (td, $^3J_{H,H}$ = 7.7 Hz, $^4J_{H,H}$ = 1.6 Hz, 1H H-aromatic) ppm.

^{13}C NMR (75.53 MHz, DMSO-d6) δ = 167.0 (C_q, C-13), 147.8 (C_q, C-7), 145.1 (C_q, C-1), 139.4 (CH, C-9), 130.0 (CH, C-aromatic), 129.7 (CH, C-aromatic), 129.4 (CH, C-aromatic), 129.1 (CH, C-aromatic), 128.5 (CH, C-aromatic), 98.1 (C_q, C-8) ppm.

HRMS calcd (M/z) for $C_{13}H_8IO_2^-$ $[M-H]^-$: 322.9574; found: 322.9572.

The recorded spectra are in accordance with the reported in literature.[24]

2'-Iodo-[1,1'-biphenyl]-3-carboxylic acid (6)

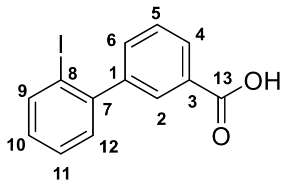

6

In a flame-dried and argon-flushed 80 mL Schlenk flask 3-carboxyphenylboronic acid (602 mg, 3.63 mmol), allylpalladium(II) chloride dimer (66.7 mg, 0.182 mmol) and PPh_3 (289 mg, 1.10 mmol) were dissolved in 25 mL abs. DMSO. 1,2-Diiodobenzene (710 µL, 1.79 g, 5.43 mmol) and K_2CO_3 (1.21 g, 8.78 mmol) were added to the yellow suspension and heated to 100 °C for 48 h. After cooling to rt, the reaction mixture was quenched by the addition of 100 mL 6 M HCl and the product was extracted with CH_2Cl_2 (3 x 100 mL). The combined organic layers were washed with 6 M HCl (1 x 100 mL) and H_2O (1 x 150 mL), dried over

Na_2SO_4, filtered and concentrated under reduced pressure to a volume of around 50 mL. The dark red solution was washed with sat. $Na_2S_2O_3$ solution (1 x 50 mL) and dried over Na_2SO_4. After filtration, the solvent was removed under reduced pressure. The crude, dark-brown solid was adsorbed onto 2 g silica gel and purified *via* column chromatography (250 mL silica gel, 13 x 5 cm, cyclohexane/EtOAc = 4/1, fraction size: 50 mL).

Yield 530 mg (1.59 mmol, 45%), grey-brownish solid, $C_{13}H_9IO_2$ [324.12]

TLC R_f = 0.14 (cyclohexane/EtOAc = 1/1; UV)

M.p. 170 - 175 °C decomp.

^{1}H NMR (300.36 MHz, DMSO-d6) δ = 13.09 (s, 1H, OH), 7.99 (m, $^3J_{H,H}$ = 10.7, 5.0 Hz, 2H, H-4, H-9), 7.87 (s, 1H, H-2), 7.63 – 7.55 (m, 2H, H-5, H-6), 7.49 (td, $^3J_{H,H}$ = 7.5 Hz, $^4J_{H,H}$ = 1.0 Hz, 1H, H-11), 7.38 (dd, $^3J_{H,H}$ = 7.5 Hz, $^4J_{H,H}$ = 1.5 Hz, 1H, H-12), 7.16 (td, $^3J_{H,H}$ = 7.7 Hz, $^4J_{H,H}$ = 1.6 Hz, 1H, H-10) ppm.

^{13}C NMR (75.53 MHz, DMSO-d6) δ = 167.0 (C_q, C-13), 144.9 (C_q, C-7), 143.8 (C_q, C-1), 139.3 (CH, C-9), 133.5 (CH, C-6), 130.6 (C_q, C-3), 130.1 (CH, C-12), 129.8 (CH, C-2), 129.6 (CH, C-10), 128.6 (CH, C-11), 128.5 (CH, C-5), 128.5 (CH, C-4), 98.5 (C_q, C-8) ppm.

HRMS calcd (M/z) for $C_{13}H_8IO_2^-$ [M-H]$^-$: 322.9574; found: 322.9571.

6-Amino-[1,1'-biphenyl]-3-carboxylic acid (24)

This compound was prepared according to a procedure described by Neuthe et al.[25]

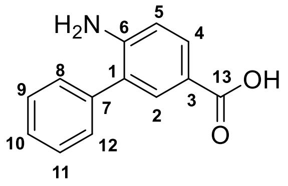

24

A 30 mL pressure reaction vessel was charged with 4-amino-3-bromobenzoic acid (1.03 g, 4.77 mmol), phenylboronic acid (621 mg, 5.09 mmol), sodium carbonate (972 mg, 9.17 mmol), TBAB (368 mg, 1.14 mmol), and palladium(II) acetate (5.4 mg, 0.024 mmol). 10 mL H_2O were added, the reaction vessel sealed, and the reaction mixture was stirred at 150 °C for 30 min in a preheated oil bath. After cooling to rt, the dark brown reaction mixture was poured into

50 mL 1 M aq. HCl and extracted with EtOAc (2 x 60 mL). The combined organic layers were washed with H_2O (1 x 50 mL) and brine (1 x 75 mL), dried over Na_2SO_4, filtered, and the solvent was eventually removed under reduced pressure. The crude, off-white solid was purified via column chromatography (100 mL silica gel, 10.5 x 3.5 cm, cyclohexane/EtOAc = 4/1 to 1/1, fraction size: 20 mL).

Yield 873 mg (4.09 mmol, 86%), off-white solid, $C_{13}H_{11}NO_2$ [213.24]

TLC R_f = 0.26 (cyclohexane/EtOAc = 1/1; UV)

M.p. 163 - 172 °C decomp.

^{1}H NMR (300.36 MHz, DMSO-d6) δ = 12.14 (s, 1H, O-H), 7.64 (dd, $^3J_{H,H}$ = 8.4 Hz, $^4J_{H,H}$ = 1.9 Hz, 1H, H-4), 7.56 (d, $^4J_{H,H}$ = 1.9 Hz, 1H, H-2), 7.51 – 7.33 (m, 5H, H-8 to H-12), 6.78 (d, $^3J_{H,H}$ = 8.5 Hz, 1H, H-5), 5.57 (s, 2H, N-H) ppm.

^{13}C NMR (75.53 MHz, DMSO-d6) δ = 167.4 (C_q, C-13), 149.6 (C_q, C-6), 138.7 (C_q, C-7), 132.0 (CH, C-4), 130.1 (CH, C-2), 128.9 (CH, C-aromatic), 128.6 (CH, C-aromatic), 127.2 (CH, C-10), 124.6 (C_q, C-1), 118.0 (C_q, C-3), 114.1 (CH, C-5) ppm.

HRMS calcd (M/z) for $C_{13}H_{11}NO_2$ $[M]^+$: 213.0790; found: 213.0785.

6-Iodo-[1,1'-biphenyl]-3-carboxylic acid (4)

This compound was prepared according to a modified procedure described by Kunz et al.[26]

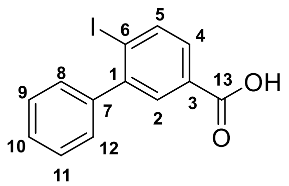

4

In an argon-flushed 80 mL Schlenk-flask flask 6-amino-[1,1'-biphenyl]-3-carboxylic acid (**24**) (987 mg, 4.63 mmol) was suspended in 18 mL H_2SO_4 conc./H_2O (1/2 v/v) and cooled to 0 °C via an ice/water bath. A solution of $NaNO_2$ (392 mg, 5.68 mmol, in 3 mL H_2O) was added dropwise over 1 h at 0 °C and the resulting yellow suspension was then stirred for 1 h at 0 °C. After addition of CuI (49.9 mg, 0.262 mmol), a solution of KI (919 mg, 5.54 mmol, in 3 mL H_2O) was added dropwise over 10 min at 0 °C. The ice/water bath was removed and the brown, sluggish reaction mixture was stirred overnight while warming to rt. Full conversion was

detected by HPLC-MS after 21 h. The reaction mixture was extracted with EtOAc (3 x 50 mL) and the combined organic layers were washed with H_2O (1 x 100 mL), sat. $Na_2S_2O_3$ solution (1 x 100 mL) and brine (1 x 100 mL), and dried over Na_2SO_4. After filtration, the solvent was removed under reduced pressure. The crude, orange solid was purified via column chromatography (120 mL silica gel, 12 x 3.5 cm, cyclohexane/EtOAc = 1/1, fraction size: 25 mL).

Yield 626 mg (1.93 mmol, 42%), off-white solid, $C_{13}H_9IO_2$ [324.12]

TLC R_f = 0.11 (cyclohexane/EtOAc = 1/1; UV)

HPLC-MS t_R = 6.41 min (method: *2-100-MeCN*); *m/z* (ESI+) = 325 $[M+H]^+$

M.p. > 220 °C decomp.

^{1}H NMR (300.36 MHz, DMSO-d6) δ = 13.18 (s, 1H, O-H), 8.13 (d, $^3J_{H,H}$ = 8.2 Hz, 1H, H-5), 7.77 (d, $^4J_{H,H}$ = 1.6 Hz, 1H, H-2), 7.63 (dd, $^3J_{H,H}$ = 8.1 Hz, $^4J_{H,H}$ = 1.8 Hz, 1H, H-4), 7.52 – 7.40 (m, 3H, H-9, H-10, H-11), 7.39 – 7.31 (m, 2H, H-8, H-12) ppm.

^{13}C NMR (75.53 MHz, DMSO-d6) δ = 166.7 (C_q, C-13), 146.3 (C_q, C-1), 142.9 (C_q, C-7), 139.9 (CH, C-5), 131.0 (C_q, C-3), 130.1 (CH, C-2), 129.4 (CH, C-4), 128.9 (CH, C-8, C-12), 128.2 (CH, C-9, C-11), 128.0 (CH, C-10), 104.9 (C_q, C-6) ppm.

HRMS calcd (M/z) for $C_{13}H_8IO_2^-$ $[M-H]^-$: 322.9574; found: 322.9572.

Methyl 2'-amino-6-bromo-[1,1'-biphenyl]-3-carboxylate (25)

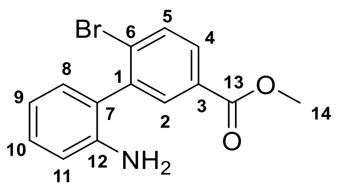

25

An argon-flushed 30 mL Schlenk flask was charged with methyl 4-bromo-3-iodobenzoate (**20**) (691 mg, 2.03 mmol), 2-aminophenylboronic acid pinacol ester (540 mg, 2.46 mmol), K_2CO_3 (691 mg, 5.00 mmol) and $PdCl_2(PPh_3)_2$ (71.8 mg, 0.102 mmol). DME (6 mL) and H_2O (1 mL) were added and the yellow suspension was stirred for 6 d at 80 °C until full conversion was detected by HPLC-MS. The orange reaction solution was diluted with 20 mL H_2O and extracted

with EtOAc (3 x 20 mL). The combined organic layers were dried over Na_2SO_4, filtered and the solvent was removed under reduced pressure. The crude, orange oil was purified via column chromatography (200 mL silica gel, 11 x 5 cm, cyclohexane/EtOAc = 10/1 to 5/1, fraction size: 50 mL).

Yield 461 mg (1.51 mmol, 74%), orange solid, $C_{14}H_{12}BrNO_2$ [306.16]

TLC R_f = 0.41 (cyclohexane/EtOAc = 4/1; UV + $KMnO_4$)

HPLC-MS t_R = 6.04 min (method: *2-100-MeCN*); *m*/*z* (ESI+) = 306 $[M+H]^+$

M.p. 110 – 112 °C

^{1}H NMR (300.36 MHz, DMSO-d6) δ = 7.93 – 7.76 (m, 3H, H-2, H-4, H-5), 7.14 – 7.05 (m, 1H, H-10), 6.90 – 6.84 (m, 1H, H-8), 6.75 (d, $^3J_{H,H}$ = 8.0 Hz, 1H, H-11), 6.61 (t, $^3J_{H,H}$ = 7.3 Hz, 1H, H-9), 4.69 (s, 2H, N-H), 3.85 (s, 3H, H-14) ppm.

^{13}C NMR (75.53 MHz, DMSO-d6) δ = 165.6 (C_q, C-13), 145.1 (C_q, C-12), 140.6 (C_q, C-1), 133.5 (CH, C-5), 132.3 (CH, C-2), 129.7 (CH, C-8), 129.6 (C_q, C-6), 129.5 (CH, C-4), 129.3 (C_q, C-3), 129.0 (CH, C-10), 124.2 (C_q, C-7), 115.9 (CH, C-9), 115.0 (CH, C-11), 52.3 (CH_3, C-14) ppm.

HRMS calcd (M/z) for $C_{14}H_{13}BrNO_2^+$ $[M+H]^+$: 306.0124; found: 306.0125.

Methyl 2'-amino-2-bromo-[1,1'-biphenyl]-4-carboxylate (26)

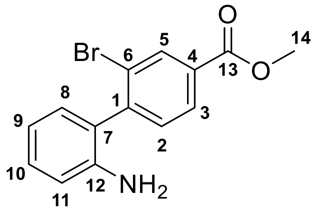

26

An argon-flushed 30 mL Schlenk flask was charged with methyl 3-bromo-4-iodobenzoate (**22**) (1.07 g, 3.13 mmol), 2-aminophenylboronic acid pinacol ester (803 mg, 3.67 mmol), K_2CO_3 (1.05 g, 7.63 mmol) and $PdCl_2(PPh_3)_2$ (111 mg, 0.158 mmol). DME (9 mL) and H_2O (1.5 mL) were added and the dark orange suspension was stirred for 40 h at 80 °C until full conversion was detected by HPLC-MS. The orange reaction solution was diluted with 20 mL H_2O and extracted with EtOAc (3 x 20 mL). The combined organic layers were dried over Na_2SO_4,

filtered and the solvent was removed under reduced pressure. The crude, orange oil was purified via column chromatography (200 mL silica gel, 11 x 5 cm, cyclohexane/EtOAc = 10/1, fraction size: 40 mL).

Yield 551 mg (1.80 mmol, 57%), orange solid, $C_{14}H_{12}BrNO_2$ [306.16]

TLC R_f = 0.41 (cyclohexane/EtOAc = 4/1; UV + $KMnO_4$)

HPLC-MS t_R = 6.08 min (method: *2-100-MeCN*); *m/z* (ESI+) = 306 $[M+H]^+$

M.p. 112 – 117 °C

^{1}H NMR (300.36 MHz, DMSO-d6) δ = 8.20 (d, $^4J_{H,H}$ = 1.4 Hz, 1H, H-5), 7.98 (dd, $^3J_{H,H}$ = 7.9, $^4J_{H,H}$ = 1.3 Hz, 1H, H-3), 7.45 (d, $^3J_{H,H}$ = 7.9 Hz, 1H, H-2), 7.09 (t, $^3J_{H,H}$ = 7.0 Hz, 1H, H-10), 6.89 – 6.82 (m, 1H, H-8), 6.75 (d, $^3J_{H,H}$ = 8.0 Hz, 1H, H-11), 6.61 (t, $^3J_{H,H}$ = 7.3 Hz, 1H, H-9), 4.68 (s, 2H, N-H), 3.89 (s, 3H, H-14) ppm.

^{13}C NMR (75.53 MHz, DMSO-d6) δ = 165.0 (C_q, C-13), 145.1 (C_q, C-1), 145.0 (C_q, C-12), 133.2 (CH, C-5), 132.4 (CH, C-2), 130.3 (C_q, C-4), 129.4 (CH, C-8), 129.1 (CH, C-10), 128.6 (CH, C-3), 124.2 (C_q, C-7), 124.0 (C_q, C-6), 115.8 (CH, C-9), 115.0 (CH, C-11), 52.5 (CH_3, C-14) ppm.

HRMS calcd (M/z) for $C_{14}H_{13}BrNO_2^+$ $[M+H]^+$: 306.0124; found: 306.0125.

2'-Amino-6-bromo-[1,1'-biphenyl]-3-carboxylic acid (7)

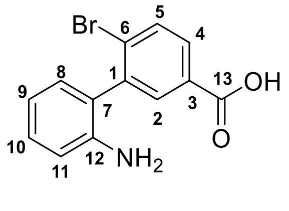

7

In a 25 mL round-bottom flask methyl 2'-amino-6-bromo-[1,1'-biphenyl]-3-carboxylate (**25**) (440 mg, 1.44 mmol) was dissolved in 5 mL THF. A solution of $LiOH\cdot H_2O$ (245 mg, 5.84 mmol) in 7 mL H_2O was added and the reaction mixture was stirred for 45 min at rt until full conversion was detected by HPLC-MS. The dark-green emulsion was diluted with 5 mL H_2O, adjusted to pH ~ 2 with 2 M aq. HCl and extracted with EtOAc (4 x 15 mL). The

combined organic layers were dried over Na_2SO_4, filtered and the solvent was removed under reduced pressure.

Yield 421 mg (1.44 mmol, quant.), light brown solid, $C_{13}H_{10}BrNO_2$ [292.13]

HPLC-MS t_R = 4.97 min (method: *2-100-MeCN*); *m/z* (ESI+) = 292 $[M+H]^+$

M.p. 235 – 240 °C decomp.

^{1}H NMR (300.36 MHz, DMSO-d6) δ = 7.83 (dt, $^3J_{H,H}$ = 8.4, 5.1 Hz, 2H, H-4, H-5), 7.76 (d, $^4J_{H,H}$ = 1.7 Hz, 1H, H-2), 7.09 (t, $^3J_{H,H}$ = 6.9 Hz, 1H, H-10), 6.91 – 6.83 (m, 1H, H-8), 6.75 (d, $^3J_{H,H}$ = 7.9 Hz, 1H, H-11), 6.61 (t, $^3J_{H,H}$ = 7.3 Hz, 1H, H-9) ppm.

^{13}C NMR (75.53 MHz, DMSO-d6) δ = 166.6 (C_q, C-13), 145.2 (C_q, C-12), 140.4 (C_q, C-1), 133.3 (CH, C-5), 132.5 (CH, C-2), 130.5 (C_q, C-3), 129.8 (CH, C-4, C-8), 129.1 (C_q, C-6), 128.9 (CH, C-10), 124.4 (C_q, C-7), 115.9 (CH, C-9), 114.9 (CH, C-11) ppm.

HRMS calcd (M/z) for $C_{13}H_{11}BrNO_2^+$ $[M+H]^+$: 291.9968; found: 291.9967.

2'-Amino-2-bromo-[1,1'-biphenyl]-4-carboxylic acid (8)

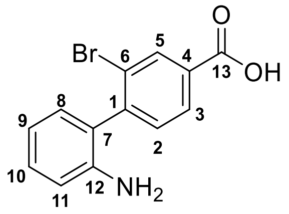

8

In a 25 mL round-bottom flask methyl 2'-amino-2-bromo-[1,1'-biphenyl]-4-carboxylate (**26**) (501 mg, 1.64 mmol) was dissolved in 5 mL THF. A solution of $LiOH\cdot H_2O$ (276 mg, 6.58 mmol) in 7 mL H_2O was added and the reaction mixture was stirred for 50 min at rt until full conversion was detected by HPLC-MS. The olive-colored emulsion was adjusted to pH ~ 2 with 2 M aq. HCl and extracted with EtOAc (3 x 15 mL). The combined organic layers were dried over Na_2SO_4, filtered and the solvent was removed under reduced pressure.

Yield 473 mg (1.62 mmol, 99%), brown solid, $C_{13}H_{10}BrNO_2$ [292.13]

HPLC-MS t_R = 4.96 min (method: *2-100-MeCN*); *m/z* (ESI+) = 292 $[M+H]^+$

M.p. 159 – 161 °C

^{1}H NMR (300.36 MHz, DMSO-d6) δ = 8.19 (d, $^4J_{H,H}$ = 1.4 Hz, 1H, H-5), 7.96 (dd, $^3J_{H,H}$ = 7.9, $^4J_{H,H}$ = 1.5 Hz, 1H, H-3), 7.42 (d, $^3J_{H,H}$ = 7.9 Hz, 1H, H-2), 7.15 – 7.05 (m, 1H, H-10), 6.86 (dd, $^3J_{H,H}$ = 7.4, $^4J_{H,H}$ = 1.2 Hz, 1H, H-8), 6.75 (d, $^3J_{H,H}$ = 7.9 Hz, 1H, H-11), 6.61 (t, $^3J_{H,H}$ = 7.3 Hz, 1H, H-9) ppm.

^{13}C NMR (75.53 MHz, DMSO-d6) δ = 166.0 (C_q, C-13), 145.0 (C_q, C-12), 144.6 (C_q, C-1), 133.4 (CH, C-5), 132.2 (CH, C-2), 131.6 (C_q, C-4), 129.5 (CH, C-8), 129.0 (CH, C-10), 128.7 (CH, C-3), 124.4 (C_q, C-7), 123.8 (C_q, C-6), 115.8 (CH, C-9), 115.0 (CH, C-11) ppm.

HRMS calcd (M/z) for $C_{13}H_{11}BrNO_2^+$ $[M+H]^+$: 291.9968; found: 291.9968.

Methyl 2'-amino-6-chloro-[1,1'-biphenyl]-3-carboxylate (27)

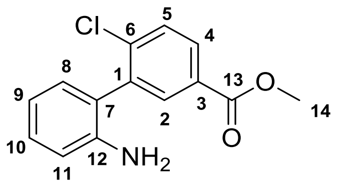

27

An argon-flushed 30 mL Schlenk flask was charged with methyl 4-chloro-3-iodobenzoate (**21**) (412 mg, 1.39 mmol), 2-aminophenylboronic acid pinacol ester (368 mg, 1.68 mmol), K_2CO_3 (476 mg, 3.44 mmol) and $PdCl_2(PPh_3)_2$ (50.2 mg, 71.5 µmol). DME (6 mL) and H_2O (1 mL) were added and the orange suspension was stirred for 15 h at 80 °C until full conversion was detected by HPLC. The brown reaction solution was diluted with 20 mL H_2O and extracted with EtOAc (3 x 15 mL). The combined organic layers were washed with brine (1 x 30 mL), dried over Na_2SO_4, filtered and the solvent was removed under reduced pressure. The crude, dark-orange oil was purified via column chromatography (100 mL silica gel, 10.5 x 3.5 cm, cyclohexane/EtOAc = 10/1, fraction size: 20 mL).

Yield 301 mg (1.15 mmol, 83%), pale orange solid, $C_{14}H_{12}ClNO_2$ [261.71]

TLC R_f = 0.31 (cyclohexane/EtOAc = 4/1; UV + $KMnO_4$)

HPLC-MS t_R = 5.80 min (method: *2-100-MeCN*); *m/z* (ESI+) = 262 $[M+H]^+$

M.p. 89 – 91 °C

^{1}H NMR (300.36 MHz, DMSO-d6) δ = 7.93 (dd, $^{3}J_{H,H}$ = 8.3, $^{4}J_{H,H}$ = 2.1 Hz, 1H, H-4), 7.82 (d, $^{4}J_{H,H}$ = 2.0 Hz, 1H, H-2), 7.71 (d, $^{3}J_{H,H}$ = 8.4 Hz, 1H, H-5), 7.16 – 7.03 (m, 1H, H-10), 6.89 (dd, $^{3}J_{H,H}$ = 7.4, $^{4}J_{H,H}$ = 1.2 Hz, 1H, H-8), 6.76 (d, $^{3}J_{H,H}$ = 7.9 Hz, 1H, H-11), 6.61 (t, $^{3}J_{H,H}$ = 7.0 Hz, 1H, H-9), 4.70 (s, 2H, N-H), 3.85 (s, 3H, H-14) ppm.

^{13}C NMR (75.53 MHz, DMSO-d6) δ = 165.5 (C_q, C-13), 145.5 (C_q, C-12), 138.5 (C_q, C-1), 138.3 (C_q, C-6), 132.6 (CH, C-2), 130.3 (CH, C-5), 129.9 (CH, C-8), 129.5 (CH, C-4), 129.1 (CH, C-10), 128.7 (C_q, C-3), 122.2 (C_q, C-7), 115.9 (CH, C-9), 115.0 (CH, C-11), 52.3 (CH_3, C-14) ppm.

HRMS calcd (M/z) for $C_{14}H_{13}ClNO_2^+$ $[M+H]^+$: 262.0629; found: 262.0630.

Methyl 2'-amino-2-chloro-[1,1'-biphenyl]-4-carboxylate (28)

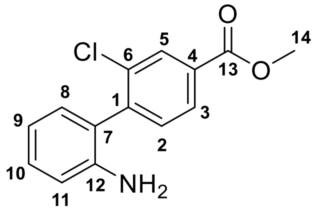

28

An argon-flushed 30 mL Schlenk flask was charged with methyl 3-chloro-4-iodobenzoate (**23**) (811 mg, 2.74 mmol), 2-aminophenylboronic acid pinacol ester (724 mg, 3.30 mmol), K_2CO_3 (945 mg, 6.84 mmol) and $PdCl_2(PPh_3)_2$ (96.9 mg, 138 µmol). DME (12 mL) and H_2O (2 mL) were added and the orange suspension was stirred for 16 h at 80 °C until full conversion was detected by HPLC. The dark red reaction solution was diluted with 30 mL H_2O and extracted with EtOAc (3 x 20 mL). The combined organic layers were dried over Na_2SO_4, filtered and the solvent was removed under reduced pressure. The crude, dark-orange oil was purified via column chromatography (125 mL silica gel, 10 x 4 cm, cyclohexane/EtOAc = 10/1, fraction size: 20 mL).

Yield 586 mg (2.24 mmol, 82%), pale pink solid, $C_{14}H_{12}ClNO_2$ [261.71]

TLC R_f = 0.32 (cyclohexane/EtOAc = 4/1; UV + $KMnO_4$)

HPLC-MS t_R = 6.15 min (method: *2-100-MeCN*); *m/z* (ESI+) = 262 $[M+H]^+$

M.p. 96 – 100 °C

^{1}H NMR (300.36 MHz, DMSO-d6) δ = 8.03 (s, 1H, H-11), 7.94 (d, $^{3}J_{H,H}$ = 7.9 Hz, 1H, H-6), 7.47 (d, $^{3}J_{H,H}$ = 7.9 Hz, 1H, H-8), 7.10 (t, $^{3}J_{H,H}$ = 7.9 Hz, 1H, H-2), 6.88 (d, $^{3}J_{H,H}$ = 7.4 Hz, 1H, H-9), 6.76 (d, $^{3}J_{H,H}$ = 8.0 Hz, 1H, H-3), 6.62 (t, $^{3}J_{H,H}$ = 7.3 Hz, 1H, H-1), 4.71 (s, 2H, H4), 3.89 (s, 3H, H-14) ppm.

^{13}C NMR (75.53 MHz, DMSO-d6) δ = 165.1 (C_q, C-13), 145.2 (C_q, C-4), 143.1 (C_q, C-7), 133.4 (C_q, C-10), 132.6 (CH, C-8), 130.2 (C_q, C-12), 130.1 (CH, C-11), 129.6 (CH, C-6), 129.1 (CH, C-2), 128.0 (CH, C-9), 122.3 (C_q, C-5), 115.8 (CH, C-1), 114.9 (CH, C-3), 52.5 (CH_3, C-14) ppm.

HRMS calcd (M/z) for $C_{14}H_{13}ClNO_2^+$ $[M+H]^+$: 262.0629; found: 262.0630.

2'-Amino-6-chloro-[1,1'-biphenyl]-3-carboxylic acid (9)

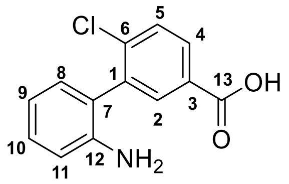

9

In a 10 mL round-bottom flask methyl 2'-amino-6-chloro-[1,1'-biphenyl]-3-carboxylate (**27**) (275 mg, 1.05 mmol) was dissolved in 3 mL THF. A solution of $LiOH{\cdot}H_2O$ (176 mg, 4.20 mmol) in 4 mL H_2O was added and the reaction mixture was stirred for 50 min at rt until full conversion was detected by HPLC. The dark-orange emulsion was diluted with 10 mL H_2O, adjusted to pH ~ 2 with 2 M aq. HCl and extracted with EtOAc (3 x 10 mL). The combined organic layers were dried over Na_2SO_4, filtered and the solvent was removed under reduced pressure.

Yield 251 mg (1.02 mmol, 97%), brown solid, $C_{13}H_{10}ClNO_2$ [247.68]

HPLC-MS t_R = 4.73 min (method: *2-100-MeCN*); *m/z* (ESI+) = 248 $[M+H]^+$

M.p. 218 – 220 °C decomp.

^{1}H NMR (300.36 MHz, DMSO-d6) δ = 7.91 (dd, $^{3}J_{H,H}$ = 8.3, $^{4}J_{H,H}$ = 2.0 Hz, 1H, H-4), 7.80 (d, $^{4}J_{H,H}$ = 1.9 Hz, 1H, H-2), 7.68 (d, $^{3}J_{H,H}$ = 8.3 Hz, 1H, H-5), 7.14 – 7.05 (m, 1H, H-10), 6.93 – 6.86 (m, 1H, H-8), 6.76 (d, $^{3}J_{H,H}$ = 7.9 Hz, 1H, H-11), 6.62 (t, $^{3}J_{H,H}$ = 7.3 Hz, 1H, H-9) ppm.

^{13}**C NMR** (75.53 MHz, DMSO-d6) δ = 166.5 (C_q, C-13), 145.4 (C_q, C-12), 138.3 (C_q, C-1), 137.8 (C_q, C-6), 132.8 (CH, C-2), 130.1 (CH, C-5), 130.0 (C_q, C-3), 129.9 (CH, C-8), 129.7 (CH, C-4), 129.0 (CH, C-10), 122.5 (C_q, C-7), 115.9 (CH, C-9), 115.0 (CH, C-11) ppm.

HRMS calcd (M/z) for $C_{13}H_{11}ClNO_2^+$ $[M+H]^+$: 248.0473; found: 248.0473.

2'-Amino-2-chloro-[1,1'-biphenyl]-4-carboxylic acid (10)

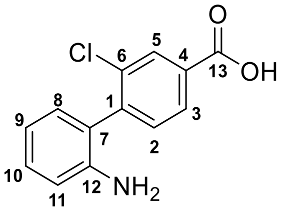

10

In a 100 mL round-bottom flask methyl 2'-amino-2-chloro-[1,1'-biphenyl]-4-carboxylate (**28**) (502 mg, 1.92 mmol) was dissolved in 20 mL THF/MeOH/H_2O (5/4/1 v/v/v). LiOH·H_2O (325 mg, 7.73 mmol) was then added, and the reaction mixture was stirred for 1 h at rt until full conversion was detected by HPLC. The dark-green solution was diluted with 20 mL H_2O, adjusted to pH ~ 2 with 2 M aq. HCl and extracted with EtOAc (3 x 30 mL). The combined organic layers were dried over Na_2SO_4, filtered and the solvent was removed under reduced pressure.

Yield 358 mg (1.44 mmol, 75%), off-white solid, $C_{13}H_{10}ClNO_2$ [247.68]

HPLC-MS t_R = 4.92 min (method: *2-100-MeCN*); *m/z* (ESI+) = 248 $[M+H]^+$

M.p. 215 – 219 °C decomp.

^{1}**H NMR** (300.36 MHz, DMSO-d6) δ = 8.01 (s, 1H, H-11), 7.93 (d, $^3J_{H,H}$ = 7.7 Hz, 1H, H-6), 7.44 (d, $^3J_{H,H}$ = 7.9 Hz, 1H, H-9), 7.09 (t, $^3J_{H,H}$ = 7.1 Hz, 1H, H-2), 6.89 (d, $^3J_{H,H}$ = 6.8 Hz, 1H, H-8), 6.75 (d, $^3J_{H,H}$ = 7.9 Hz, 1H, H-3), 6.61 (t, $^3J_{H,H}$ = 7.2 Hz, 1H, H-1) ppm.

^{13}**C NMR** (75.53 MHz, DMSO-d6) δ = 166.1 (C_q, C-13), 145.3 (C_q, C-4), 142.6 (C_q, C-7), 133.2 (C_q, C-12), 132.4 (CH, C-8), 131.5 (C_q, C-10), 130.3 (CH, C-11), 129.7 (CH, C-6), 129.0 (CH, C-2), 128.2 (CH, C-9), 122.5 (C_q, C-5), 115.9 (CH, C-1), 115.0 (CH, C-3) ppm.

HRMS calcd (M/z) for $C_{13}H_{11}ClNO_2^+$ $[M+H]^+$: 248.0473; found: 248.0474.

Synthesis of Diarylhalonium Salts

(4-Carboxyphenyl)(phenyl)iodonium trifluoromethanesulfonate (1a)

This compound was prepared according to a modified procedure described by Bielawski et al.[27]

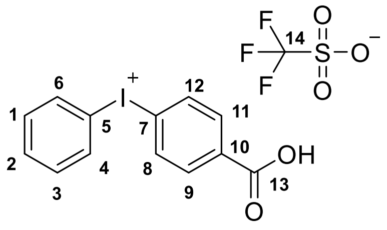

1a

In a flame-dried and argon-flushed 80 mL Schlenk flask 4-iodobenzoic acid (514 mg, 2.07 mmol), mCPBA (77 w%) (545 mg, 2.43 mmol) and benzene (220 µL, 194 mg, 2.48 mmol) were added to 20 mL abs. CH_2Cl_2 and the colorless suspension was stirred at rt for 10 min. After cooling to 0 °C via an ice/water bath, triflic acid (360 µL, 616 mg, 4.10 mmol) was added dropwise over 10 min. The resulting dark green suspension was stirred for 30 min at 0 °C and then 30 min at rt. After concentration of the reaction mixture under reduced pressure, the remaining residue was taken up in 50 mL Et_2O and stirred for 20 min at rt. The product was collected by filtration, washed with Et_2O (3 x 10 mL) and dried under high vacuum.

Yield 595 mg (1.25 mmol, 61%), light brown solid, $C_{14}H_{10}F_3IO_5S$ [474.19]

M.p. 191 - 193 °C decomp.

^{1}H NMR (300.36 MHz, DMSO-d6) δ = 8.34 (d, $^3J_{H,H}$ = 8.4 Hz, 2H, H-8, H-12), 8.27 (d, $^3J_{H,H}$ = 7.7 Hz, 2H, H-4, H-6), 8.00 (d, $^3J_{H,H}$ = 8.4 Hz, 2H, H-9, H-11), 7.68 (t, $^3J_{H,H}$ = 7.3 Hz, 1H, H-2), 7.54 (t, $^3J_{H,H}$ = 7.7 Hz, 2H, H-1, H-3) ppm.

^{13}C NMR (75.53 MHz, DMSO-d6) δ = 166.2 (C_q, C-13), 135.4 (CH, C-4, C-6, C-8, C-12), 133.9 (C_q, C-10), 132.3 (CH, C-2), 132.1 (CH, C-9, C-11), 131.9 (CH, C-1, C-3), 120.8 (C_q, C-7), 116.6 (C_q, C-5) ppm.

^{19}F NMR (470.53 MHz, DMSO-d^6) δ = -77.7 ppm.

HRMS calcd (M/z) for $C_{13}H_{10}IO_2^+$ $[M\text{-}OTf]^+$: 324.9720; found: 324.9720.

The recorded spectra are in accordance with the reported in literature.[27]

(3-Carboxyphenyl)(phenyl)iodonium trifluoromethanesulfonate (1b)

This compound was prepared according to a modified procedure described by Bielawski et al.[27]

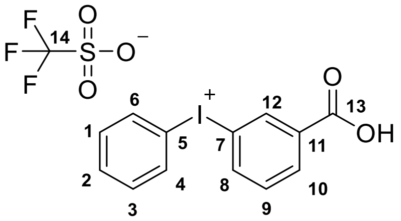

1b

In a flame-dried and argon-flushed 80 mL Schlenk flask 3-iodobenzoic acid (508 mg, 2.05 mmol), mCPBA (77 w%) (552 mg, 2.47 mmol) and benzene (220 µL, 194 mg, 2.48 mmol) were added to 20 mL abs. CH_2Cl_2 and the light brown suspension was stirred at rt for 10 min. After cooling to 0 °C via an ice/water bath, triflic acid (360 µL, 616 mg, 4.10 mmol) was added dropwise over 5 min. The resulting yellow-orange emulsion was stirred for 30 min at 0 °C and then 3 h at rt. After concentration of the reaction mixture under reduced pressure, the remaining residue was taken up in 40 mL Et_2O and stirred for 20 min at rt. The product was collected by filtration, washed with Et_2O (3 x 10 mL) and dried under high vacuum.

Yield 446 mg (0.941 mmol, 46%), light brown solid, $C_{14}H_{10}F_3IO_5S$ [474.19]

M.p. 145 - 148 °C decomp.

^{1}H NMR (300.36 MHz, DMSO-d6) δ = 8.76 (s, 1H, H-12), 8.45 (d, $^3J_{H,H}$ = 7.5 Hz, 1H, H-8), 8.30 (d, $^3J_{H,H}$ = 7.5 Hz, 2H, H-4, H-6), 8.16 (d, $^3J_{H,H}$ = 7.6 Hz, 1H, H-10), 7.73 – 7.61 (m, 2H, H-2, H-9), 7.54 (t, $^3J_{H,H}$ = 7.4 Hz, 2H, H-1, H-3) ppm.

^{13}C NMR (75.53 MHz, DMSO-d6) δ = 165.5 (C_q, C-13), 139.1 (CH, C-8), 135.7 (CH, C-12), 135.4 (CH, C-4, C-6), 133.7 (C_q, C-11), 132.5 (CH, C-10), 132.2 (CH, C-2), 132.0 (CH, C-9), 131.8 (CH, C-1, C-3), 116.6 (C_q, C-5), 116.5 (C_q, C-7) ppm.

^{19}F NMR (470.53 MHz, DMSO-d^6) δ = -77.7 ppm.

HRMS calcd (M/z) for $C_{13}H_{10}IO_2^+$ [M-OTf]$^+$: 324.9725; found: 324.9717.

2-Carboxydibenzo[*b*,*d*]iodol-5-ium trifluoromethanesulfonate (1c)

This compound was prepared according to a modified procedure described by Bielawski et al.[27]

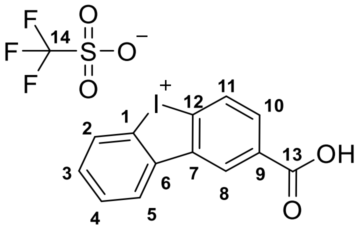

1c

In a flame-dried and argon-flushed 30 mL Schlenk flask 6-iodo-[1,1'-biphenyl]-3-carboxylic acid (**4**) (493 mg, 1.52 mmol) and mCPBA (77 w%) (413 mg, 1.84 mmol) were added to 5-10 mL abs. CH_2Cl_2 and the pale orange suspension was stirred at rt for 10 min. After cooling to 0 °C via an ice/water bath, triflic acid (410 µL, 701 mg, 4.67 mmol) was added dropwise and the resulting grey suspension was stirred for 10 min at 0 °C prior to warming to rt. After stirring for 1.5 h at rt, the reaction mixture was concentrated under reduced pressure. The remaining residue was taken up in 30 mL Et_2O and stirred for 30 min at rt. The product was collected by filtration, washed with Et_2O (3 x 5 mL) and dried under high vacuum.

Yield 546 mg (1.16 mmol, 76%), light grey solid, $C_{14}H_8F_3IO_5S$ [472.17]

M.p. > 265 °C decomp.

^{1}H NMR (300.36 MHz, DMSO-d6) δ = 13.62 (bs, 1H, O-H), 8.88 (s, 1H, H-8), 8.62 (d, $^3J_{H,H}$ = 7.6 Hz, 1H, H-5), 8.35 (d, $^3J_{H,H}$ = 8.5 Hz, 1H, H-11), 8.22 (t, $^3J_{H,H}$ = 8.9 Hz, 2H, H-2, H-10), 7.92 – 7.82 (m, 1H, H-4), 7.75 (t, $^3J_{H,H}$ = 7.5 Hz, 1H, H-3) ppm.

^{13}C NMR (75.53 MHz, DMSO-d6) δ = 166.4 (C_q, C-13), 142.3 (C_q, C-7), 140.9 (C_q, C-6), 133.4 (C_q, C-9), 131.5 (CH, C-3), 131.0 (CH, C-4, C-10), 130.9 (CH, C-11), 130.5 (CH, C-2), 127.5 (CH, C-5), 127.0 (CH, C-8), 126.2 (C_q, C-12), 122.1 (C_q, C-1) ppm.

^{19}F NMR (282.40 MHz, DMSO-d^6) δ = -77.8 ppm.

HRMS calcd (M/z) for $C_{13}H_8IO_2^+$ [M-OTf]$^+$: 322.9563; found: 322.9562.

3-Carboxydibenzo[*b*,*d*]iodol-5-ium trifluoromethanesulfonate (1d)

This compound was prepared according to a modified procedure described by Bielawski et al.[27]

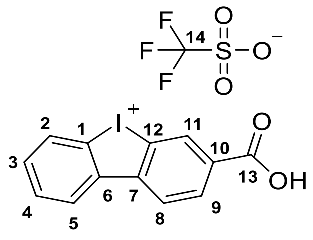

1d

In a flame-dried and argon-flushed 30 mL Schlenk flask 2'-iodo-[1,1'-biphenyl]-4-carboxylic acid (**5**) (299 mg, 0.922 mmol) and mCPBA (77 w%) (244 mg, 1.09 mmol) were added to 6 mL abs. CH_2Cl_2 and the orange suspension was stirred at rt for 10 min. After cooling to 0 °C via an ice/water bath, triflic acid (240 µL, 410 mg, 2.73 mmol) was added dropwise and the resulting light brown suspension was stirred for 10 min at 0 °C prior to warming to rt. After stirring for 1.5 h at rt, the reaction mixture was concentrated under reduced pressure. The remaining residue was taken up in 30 mL Et_2O and stirred for 20 min at rt. The product was collected by filtration, washed with Et_2O (3 x 5 mL) and dried under high vacuum.

Yield 320 mg (0.677 mmol, 74%), off-white solid, $C_{14}H_8F_3IO_5S$ [472.17]

M.p. >320 °C decomp.

^{1}H NMR (300.36 MHz, DMSO-d6) δ = 13.57 (s, 1H, O-H), 8.77 (d, $^4J_{H,H}$ = 1.2 Hz, 1H, H-11), 8.56 (t, $^3J_{H,H}$ = 7.2 Hz, 2H, H-aromatic), 8.31 (dd, $^3J_{H,H}$ = 8.2 Hz, $^4J_{H,H}$ = 1.3 Hz, 1H, H-9), 8.24 (d, $^3J_{H,H}$ = 7.6 Hz, 1H, H-aromatic), 7.89 (t, $^3J_{H,H}$ = 7.1 Hz, 1H, H-aromatic), 7.77 (t, $^3J_{H,H}$ = 7.2 Hz, 1H, H-aromatic) ppm.

^{13}C NMR (75.53 MHz, DMSO-d6) δ = 165.6 (C_q, C-13), 145.5 (C_q, C-7), 140.7 (C_q, C-6), 132.6 (C_q, C-10), 132.0 (CH, C-aromatic), 131.6 (CH, C-11), 131.3 (CH, C-9), 130.9 (CH, C-aromatic), 130.7 (CH, C-aromatic), 127.8 (CH, C-aromatic), 126.8 (CH, C-aromatic), 122.7 (C_q, C-aromatic), 121.8 (C_q, C-aromatic) ppm.

^{19}F NMR (282.40 MHz, DMSO-d^6) δ = -77.8 ppm.

HRMS calcd (M/z) for $C_{13}H_8IO_2^+$ [M-OTf]$^+$: 322.9563; found: 322.9562.

4-Carboxydibenzo[*b*,*d*]iodol-5-ium trifluoromethanesulfonate (1e)

This compound was prepared according to a modified procedure described by Bielawski et al.[27]

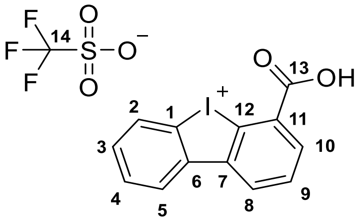

1e

In a flame-dried and argon-flushed 30 mL Schlenk flask 2'-iodo-[1,1'-biphenyl]-3-carboxylic acid (**6**) (403 mg, 1.24 mmol) and mCPBA (77 w%) (337 mg, 1.50 mmol) were added to 8 mL abs. CH_2Cl_2 and the brown solution was stirred at rt for 10 min. After cooling to 0 °C via an ice/water bath, triflic acid (320 µL, 547 mg, 3.64 mmol) was added dropwise and the resulting grey suspension was stirred for 10 min at 0 °C prior to warming to rt. After stirring for 1.5 h at rt, the reaction mixture was concentrated under reduced pressure. The remaining residue was taken up in 40 mL Et_2O and stirred for 20 min at rt. The product was collected by filtration, washed with Et_2O (3 x 5 mL) and dried under high vacuum.

Yield 491 mg (1.04 mmol, 84%), off-white solid, $C_{14}H_8F_3IO_5S$ [472.17]

M.p. 214 - 218 °C decomp.

^{1}H NMR (300.36 MHz, DMSO-d6) δ = 8.75 (d, $^3J_{H,H}$ = 7.5 Hz, 1H, H-8), 8.56 (d, $^3J_{H,H}$ = 7.2 Hz, 1H, H-5), 8.29 (d, $^3J_{H,H}$ = 7.8 Hz, 2H, H-2, H-10), 8.05 (t, $^3J_{H,H}$ = 7.6 Hz, 1H, H-9), 7.89 (t, $^3J_{H,H}$ = 7.4 Hz, 1H, H-4), 7.75 (t, $^3J_{H,H}$ = 7.3 Hz, 1H, H-3) ppm.

^{13}C NMR (75.53 MHz, DMSO-d6) δ = 167.2 (C_q, C-13), 141.5 (C_q, C-7), 139.9 (C_q, C-6), 132.0 (CH, C-9), 131.3 (CH, C-3), 130.7 (CH, C-4), 130.6 (CH, C-aromatic), 130.3 (CH, C-aromatic), 130.3 (CH, C-8), 129.8 (C_q, C-11), 127.3 (CH, C-5), 122.8 (C_q, C-1), 120.5 (C_q, C-12) ppm.

^{19}F NMR (470.53 MHz, DMSO-d^6) δ = -77.8 ppm.

HRMS calcd (M/z) for $C_{13}H_8IO_2^+$ [M-OTf]$^+$: 322.9563; found: 322.9561.

2-Carboxydibenzo[*b*,*d*]bromol-5-ium chloride (11)

This compound was prepared according to a modified procedure described by Yoshida et al.[28]

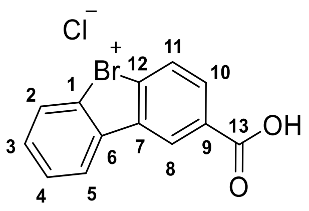

11

In a 100 mL round-bottom flask 2'-amino-6-bromo-[1,1'-biphenyl]-3-carboxylic acid (**7**) (340 mg, 1.16 mmol) was suspended in 15 mL 6 M aq. HCl and stirred for 1 h at 100 °C. After cooling to 0 °C via an ice/water bath, a solution of $NaNO_2$ (482 mg, 6.99 mmol) in 3 mL H_2O was added and the brown suspension was stirred for 2 h while warming to room temperature. After subsequent cooling to 0 °C, urea (418 mg, 6.96 mmol) was added in one portion and the reaction mixture was stirred for 1 h at 50 °C until gas evolution ceased and full conversion was detected by HPLC-MS. The product was collected by filtration using a sintered glass frit, washed with ice water (2 x 10 mL) and Et_2O (2 x 10 mL) and dried under high vacuum.

Yield 261 mg (0.838 mmol, 72%), off-white solid, $C_{13}H_8BrClO_2$ [311.56]

HPLC-MS t_R = 3.30 min (method: *2-100-MeCN*); *m/z* (ESI+) = 275, 277 $[M\text{-}Cl]^+$

M.p. 205 – 210 °C

^{1}H NMR (300.36 MHz, DMSO-d6) δ = 8.96 (s, 2H, H-, H-8, H-11), 8.90 (d, $^3J_{H,H}$ = 7.7 Hz, 1H, H-2), 8.70 (d, $^3J_{H,H}$ = 5.6 Hz, 1H, H-5), 8.28 (d, $^3J_{H,H}$ = 7.8 Hz, 1H, H-10), 7.89 (d, $^3J_{H,H}$ = 6.5 Hz, 1H, H-4), 7.84 (d, $^3J_{H,H}$ = 7.0 Hz, 1H, H-3) ppm.

^{13}C NMR (75.53 MHz, DMSO-d6) δ = 166.1 (C_q, C-13), 140.8 (C_q, C-12), 138.2 (C_q, C-1), 135.9 (C_q, C-7), 134.6 (C_q, C-6), 133.7 (C_q, C-9), 131.7 (CH, C-3), 131.5 (CH, C-10), 131.1 (CH, C-2), 126.5 (CH, C-aromatic), 126.4 (CH, C-aromatic), 126.3 (CH, C-aromatic), 126.2 (CH, C-aromatic) ppm.

HRMS calcd (M/z) for $C_{13}H_8BrO_2^+$ $[M\text{-}Cl]^+$: 274.9702; found: 274.9700.

2-Carboxydibenzo[*b*,*d*]bromol-5-ium trifluoromethanesulfonate (1f)

This compound was prepared according to a modified procedure described by Yoshida et al.[28]

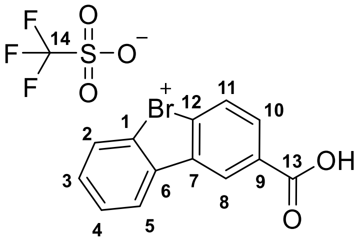

1f

In a 25 mL round-bottom flask 2-carboxydibenzo[*b*,*d*]bromol-5-ium chloride (**11**) (260 mg, 0.835 mmol) was suspended in 20 mL MeOH and a solution of silver trifluoromethanesulfonate (220 mg, 0.856 mmol) in 5 mL MeOH was added. After stirring for 1.5 h at rt, the brown suspension was filtered, and the filtrate was evaporated to dryness. The residue was taken up in 5 mL Et_2O and the product was collected by filtration, washed with Et_2O (3 x 5 mL) and dried under high vacuum.

Yield 293 mg (0.689 mmol, 83%), light brown solid, $C_{14}H_8BrF_3O_5S$ [425.17]

HPLC-MS t_R = 3.28 min (method: *2-100-MeCN*); *m/z* (ESI+) = 275, 277 $[M\text{-}OTf]^+$

M.p. > 225 °C decomp.

^{1}H NMR (300.36 MHz, DMSO-d6) δ = 13.78 (s, 1H, O-H), 9.03 (d, $^4J_{H,H}$ = 1.6 Hz, 1H, H-8), 8.84 – 8.73 (m, 1H, H-5), 8.59 (d, $^3J_{H,H}$ = 8.9 Hz, 1H, H-11), 8.49 (d, $^3J_{H,H}$ = 8.3 Hz, 1H, H-2), 8.34 (dd, $^3J_{H,H}$ = 8.9, $^4J_{H,H}$ = 1.7 Hz, 1H, H-10), 7.95 (t, $^3J_{H,H}$ = 7.2 Hz, 1H, H-4), 7.88 (m, 1H, H-3) ppm.

^{13}C NMR (75.53 MHz, DMSO-d6) δ = 166.0 (C_q, C-13), 139.7 (C_q, C-12), 137.2 (C_q, C-1), 136.0 (C_q, C-7), 134.7 (C_q, C-6), 134.0 (C_q, C-9), 132.3 (CH, C-3), 132.0 (CH, C-10), 131.3 (CH, C-4), 126.9 (CH, C-5), 126.7 (CH, C-8), 125.9 (CH, C-11), 125.5 (CH, C-2) ppm.

^{19}F NMR (282.40 MHz, DMSO-d^6) δ = -77.8 ppm.

HRMS calcd (M/z) for $C_{13}H_8BrO_2^+$ $[M\text{-}OTf]^+$: 274.9702; found: 274.9700.

3-Carboxydibenzo[*b,d*]bromol-5-ium chloride (12)

This compound was prepared according to a modified procedure described by Yoshida et al.[28]

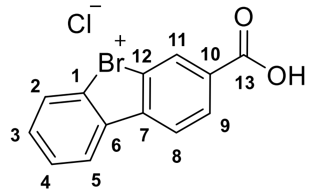

12

In a 100 mL round-bottom flask 2'-amino-2-bromo-[1,1'-biphenyl]-4-carboxylic acid (**8**) (402 mg, 1.38 mmol) was suspended in 15 mL 6 M aq. HCl and stirred for 1 h at 100 °C. After cooling to 0 °C via an ice/water bath, a solution of $NaNO_2$ (567 mg, 8.22 mmol) in 3 mL H_2O was added. The ice bath was removed, and the brown suspension was stirred for 2 h at rt. Subsequently, urea (494 mg, 8.22 mmol) was added in one portion at 0 °C (ice bath). The resulting light brown suspension was then slowly warmed to 50 °C and full conversion could be detected by HPLC after 40 min. The reaction mixture was again cooled to 0 °C and the product was collected by filtration using a sintered glass frit, washed with ice water (2 x 10 mL) and Et_2O (2 x 10 mL) and dried under high vacuum.

Yield 289 mg (0.928 mmol, 67%), off-white solid, $C_{13}H_8BrClO_2$ [311.56]

HPLC-MS t_R = 3.35 min (method: *2-100-MeCN*); *m/z* (ESI+) = 275, 277 $[M-Cl]^+$

M.p. 208 – 212 °C

^{1}H NMR (300.36 MHz, DMSO-d6) δ = 13.70 (s, 1H, O-H), 9.39 (s, 1H, H-11), 8.88 (d, $^3J_{H,H}$ = 8.2 Hz, 1H, H-2), 8.64 (t, $^3J_{H,H}$ = 7.6 Hz, 2H, H-5, H-8), 8.38 (d, $^3J_{H,H}$ = 8.0 Hz, 1H, H-9), 7.95 (t, $^3J_{H,H}$ = 7.4 Hz, 1H, H-4), 7.87 (t, $^3J_{H,H}$ = 7.3 Hz, 1H, H-3) ppm.

^{13}C NMR (75.53 MHz, DMSO-d6) δ = 165.4 (C_q, C-13), 139.0 (C_q, C-7), 138.6 (C_q, C-1), 137.4 (C_q, C-12), 134.4 (C_q, C-6), 133.1 (C_q, C-10), 132.3 (CH, C-3), 131.5 (CH, C-9), 131.2 (CH, C-4), 127.2 (CH, C-11), 126.7 (CH, C-5), 126.3 (CH, C-2), 125.7 (CH, C-8) ppm.

HRMS calcd (M/z) for $C_{13}H_8BrO_2^+$ $[M-Cl]^+$: 274.9702; found: 274.9701.

3-Carboxydibenzo[*b,d*]bromol-5-ium trifluoromethanesulfonate (1g)

This compound was prepared according to a modified procedure described by Yoshida et al.[28]

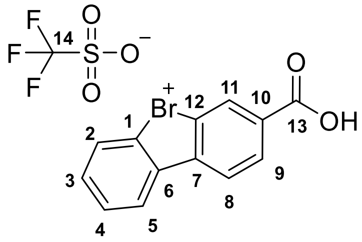

1g

In a 25 mL round-bottom flask 3-carboxydibenzo[*b,d*]bromol-5-ium chloride (**12**) (235 mg, 0.754 mmol) was suspended in 20 mL MeOH and a solution of silver trifluoromethanesulfonate (196 mg, 0.762 mmol) in 5 mL MeOH was added. After stirring for 1 h at rt, the light brown suspension was filtered, and the filtrate was evaporated to dryness. The residue was taken up in 10 mL Et_2O and the product was collected by filtration, washed with Et_2O (3 x 5 mL) and dried under high vacuum.

Yield 272 mg (0.640 mmol, 85%), light brown solid, $C_{14}H_8BrF_3O_5S$ [425.17]

M.p. > 275 °C decomp.

^{1}H NMR (300.36 MHz, DMSO-d6) δ = 13.84 (s, 1H), 9.05 (s, 1H, H-11), 8.68 (t, $^3J_{H,H}$ = 7.4 Hz, 2H, H-5, H-8), 8.51 (d, $^3J_{H,H}$ = 8.4 Hz, 1H, H-2), 8.41 (d, $^3J_{H,H}$ = 8.1 Hz, 1H), 7.99 (t, $^3J_{H,H}$ = 7.4 Hz, 1H, H-4), 7.95 – 7.87 (m, 1H, H-3) ppm.

^{13}C NMR (75.53 MHz, DMSO-d6) δ = 165.3 (C_q, C-13), 139.1 (C_q, C-7), 137.7 (C_q, C-1), 136.4 (C_q, C-12), 134.5 (C_q, C-6), 133.4 (C_q, C-10), 132.8 (CH, C-3), 131.7 (CH, C-9), 131.4 (CH, C-4), 127.1 (CH, C-5), 126.6 (CH, C-11), 126.2 (CH, C-8), 125.7 (CH, C-2) ppm.

^{19}F NMR (282.40 MHz, DMSO-d^6) δ = -77.8 ppm.

HRMS calcd (M/z) for $C_{13}H_8BrO_2^+$ [M-OTf]$^+$: 274.9702; found: 274.9701.

2-Carboxydibenzo[*b*,*d*]chlorol-5-ium chloride (13)

This compound was prepared according to a modified procedure described by Yoshida et al.[28]

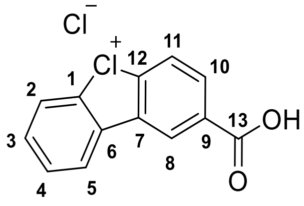

13

In a 100 mL round-bottom flask 2'-amino-6-chloro-[1,1'-biphenyl]-3-carboxylic acid (**9**) (648 mg, 2.62 mmol) was suspended in 15 mL 6 M aq. HCl and stirred for 1 h at 100 °C. After cooling to 0 °C via an ice/water bath, a solution of $NaNO_2$ (1.08 g, 15.7 mmol) in 6 mL H_2O was added and the brown suspension was stirred for 2.5 h while warming to room temperature. After subsequent cooling to 0 °C, urea (946 mg, 15.8 mmol) was added in one portion and the reaction mixture was stirred for 30 min at 50 °C until gas evolution ceased and full conversion was detected by HPLC-MS. The product was collected by filtration using a sintered glass frit, washed with ice water (2 x 10 mL) and Et_2O (2 x 10 mL), dried under high vacuum, and directly used for the next step.

Yield 353 mg (1.32 mmol, 50%), brown solid, $C_{13}H_8Cl_2O_2$ [267.11]

HPLC-MS t_R = 3.26 min (method: *2-100-MeCN*); *m/z* (ESI+) = 231 $[M\text{-}Cl]^+$

2-Carboxydibenzo[*b*,*d*]chlorol-5-ium trifluoromethanesulfonate (1h)

This compound was prepared according to a modified procedure described by Yoshida et al.[28]

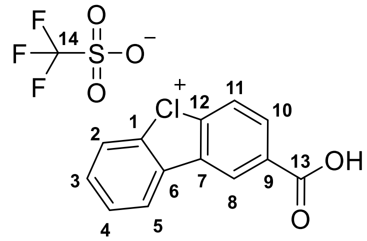

1h

In a 25 mL round-bottom flask 2-carboxydibenzo[*b*,*d*]chlorol-5-ium chloride (**13**) (353 mg, 1.32 mmol) was dissolved in 15 mL MeOH and silver trifluoromethanesulfonate (339 mg,

1.32 mmol) was added. After stirring for 15 min at rt, the brown suspension was filtered through a sintered glass frit, and the filtrate was evaporated to dryness. The residue was taken up in 5 mL Et_2O and the product was collected by filtration, washed with Et_2O (3 x 5 mL) and dried under high vacuum.

Yield 409 mg (1.07 mmol, 81%), pale brown solid, $C_{14}H_8ClF_3O_5S$ [380.72]

M.p. 152 – 160 °C decomp.

^{1}H NMR (300.36 MHz, DMSO-d6) δ = 13.87 (bs, 1H, O-H), 9.16 (d, $^4J_{H,H}$ = 1.8 Hz, 1H, H-8), 8.87 (dd, $^3J_{H,H}$ = 7.3, $^4J_{H,H}$ = 1.8 Hz, 1H, H-5), 8.77 (d, $^3J_{H,H}$ = 9.0 Hz, 1H, H-11), 8.71 – 8.63 (m, 1H, H-2), 8.44 (dd, $^3J_{H,H}$ = 9.0, $^4J_{H,H}$ = 1.8 Hz, 1H, H-10), 8.06 – 7.91 (m, 2H, H-3, H-4) ppm.

^{13}C NMR (75.53 MHz, DMSO-d6) δ = 165.7 (C_q, C-13), 142.7 (C_q, C-12), 140.9 (C_q, C-1), 134.3 (C_q, C-9), 132.7 (C_q, C-aromatic), 132.4 (CH, C-3), 132.3 (CH, C-10), 131.8 (CH, C-4), 131.5 (C_q, C-aromatic), 126.1 (CH, C-5, C-8), 123.3 (CH, C-11), 122.9 (CH, C-2) ppm.

^{19}F NMR (470.53 MHz, DMSO-d^6) δ = -77.7 ppm.

HRMS calcd (M/z) for $C_{13}H_8ClO_2^+$ $[M\text{-}OTf]^+$: 231.0207; found: 231.0208.

3-Carboxydibenzo[*b*,*d*]chlorol-5-ium chloride (14)

This compound was prepared according to a modified procedure described by Yoshida et al.[28]

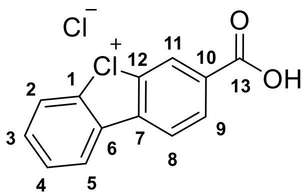

14

In a 50 mL round-bottom flask 2'-amino-2-chloro-[1,1'-biphenyl]-4-carboxylic acid (**10**) (310 mg, 1.25 mmol) was suspended in 15 mL 6 M aq. HCl and stirred for 1 h at 100 °C. After cooling to 0 °C via an ice/water bath, a solution of $NaNO_2$ (524 mg, 7.59 mmol) in 3 mL H_2O was added and the yellowish suspension was stirred for 1.5 h while warming to room temperature. After subsequent cooling to 0 °C, urea (453 mg, 7.54 mmol) was added in one portion and the reaction mixture was stirred for 30 min at 50 °C until gas evolution ceased. The

product was collected by filtration using a sintered glass frit, washed with ice water (2 x 10 mL) and Et_2O (2 x 10 mL), dried under high vacuum, and directly used for the next step.

Yield 125 mg (0.468 mmol, 37%), brown solid, $C_{13}H_8Cl_2O_2$ [267.11]

3-Carboxydibenzo[*b,d*]chlorol-5-ium trifluoromethanesulfonate (1i)

This compound was prepared according to a modified procedure described by Yoshida et al.[28]

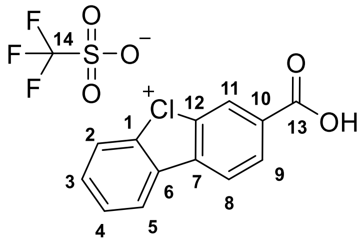

1i

In a 25 mL round-bottom flask 3-carboxydibenzo[*b,d*]chlorol-5-ium chloride (**14**) (125 mg, 0.468 mmol) was dissolved in 10 mL MeOH and silver trifluoromethanesulfonate (122 mg, 0.474 mmol) was added. After stirring for 15 min at rt, the grey suspension was filtered, and the filtrate was evaporated to dryness. The residue was taken up in 5 mL Et_2O and the product was collected by filtration, washed with Et_2O (3 x 5 mL), and dried under high vacuum.

Yield 177 mg (0.465 mmol, quant.), pale pink solid, $C_{14}H_8ClF_3O_5S$ [380.72]

M.p. 207 – 210 °C decomp.

^{1}H NMR (300.36 MHz, DMSO-d6) δ = 9.26 (d, $^4J_{H,H}$ = 1.0 Hz, 1H, H-11), 8.80 – 8.67 (m, 3H, H-2, H-5, H-8), 8.47 (dd, $^3J_{H,H}$ = 8.1, $^4J_{H,H}$ = 1.0 Hz, 1H, H-9), 8.09 – 7.96 (m, 2H, H-3, H-4) ppm.

^{13}C NMR (75.53 MHz, DMSO-d6) δ = 165.1 (C_q, C-13), 141.2 (C_q, C-12), 140.0 (C_q, C-1), 135.7 (C_q, C-7), 133.7 (C_q, C-10), 132.9 (CH, C-3), 132.2 (CH, C-9), 131.9 (CH, C-4), 131.2 (C_q, C-6), 126.2 (CH, C-5 or C-8), 125.4 (CH, C-5 or C-8), 124.1 (CH, C-11), 123.1 (CH, C-2) ppm.

^{19}F NMR (470.53 MHz, DMSO-d^6) δ = -77.7 ppm.

HRMS calcd (M/z) for $C_{13}H_8ClO_2^+$ [M-OTf]$^+$: 231.0207; found: 231.0207.

Synthesis of Diarylhalonium ABPP probes

General Procedure A for the Synthesis of Iodonium- and Bromonium-based Probes via Acyl chloride

In a flame-dried and argon-flushed 20 mL Schlenk flask the corresponding iodonium/bromonium compound (0.422 mmol) was dissolved in 4 mL abs. DMF and cooled to 0 °C via an ice/water bath. $SOCl_2$ (76.6 µL, 126 mg, 1.06 mmol) was added dropwise to the solution at 0 °C. The stopper was exchanged against a bubbler and the resulting suspension was stirred for 2 h at rt. The solvent and the volatiles were then removed under oil pump vacuum at 40 °C with a cooling trap. The residue was taken up in 2 mL CH_2Cl_2 and again all volatiles were removed under reduced pressure with a cooling trap. This process was repeated twice. The crude intermediate was taken up in 4 mL abs. DMF and cooled to 0 °C via an ice/water bath. DIPEA (220 µL, 163 mg, 1.26 mmol) was added and the reaction mixture was stirred for 10 min at 0 °C. Subsequently, the corresponding azido amine (0.506 mmol) was added to the reaction mixture and the resulting suspension/solution was stirred for 10 min at 0 °C and then 2 h at rt. After removal of the solvent and volatiles under reduced pressure, the crude product was adsorbed onto 500 mg silica gel and purified via column chromatography (120 mL silica gel, 12 x 3.5 cm, CH_2Cl_2/MeOH = 50/1 to 10/1, fraction size: 25 mL). After column chromatography, remaining DIPEA was removed by dissolving the product in 20 mL CH_2Cl_2 and washing this solution with 1 M aq. HCl (2 x 20 mL). The organic layer was dried over Na_2SO_4, filtered and the solvent was removed under reduced pressure. The product was then collected via precipitation from EtOAc/cyclohexane or CH_2Cl_2/Et_2O.

General Procedure B for the Synthesis of Chloronium-based Probes via Carbodiimide-mediated Amidation

In an argon-flushed 30 mL Schlenk flask the corresponding azido amine (0.151 mmol) was dissolved in 5 mL abs. CH_2Cl_2 and cooled to 0 °C via an ice/water bath. The corresponding chloronium compound (0.227 mmol) and DMAP (45.0 µmol) were added and the suspension was stirred for 10 min at 0 °C. Subsequently, EDC hydrochloride (0.227 mmol) was added, the ice-water bath removed and the reaction mixture was stirred while warming to rt until full conversion was detected by HPLC-MS (1.5 – 3 h). The solvent was removed under reduced

pressure and the crude product was purified via column chromatography (50 mL silica gel, 7 x 3 cm, CH_2Cl_2/MeOH = 10/1, fraction size: 10 mL).

(4-((10-Azidodecyl)carbamoyl)phenyl)(phenyl)iodonium trifluoromethanesulfonate (3aa)

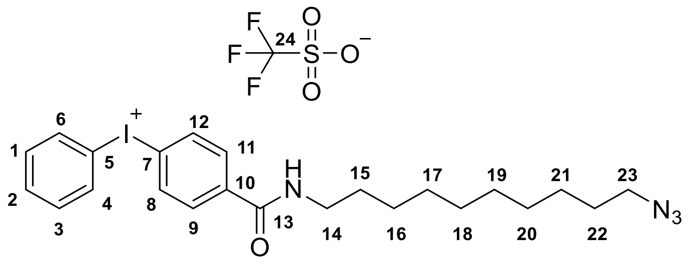

3aa

Starting from (4-carboxyphenyl)(phenyl)iodonium trifluoromethanesulfonate (**1a**) (201 mg, 0.424 mmol) and 10-azidobutan-1-amine (**2a**) (107 mg, 0.540 mmol) synthesis of (4-((10-azidodecyl)carbamoyl)phenyl)(phenyl)iodonium trifluoromethanesulfonate (**3aa**) was executed as described in general procedure A.

Yield 90 mg (0.138 mmol, 32%), pale yellow solid, $C_{24}H_{30}F_3IN_4O_4S$ [654.49]

TLC R_f = 0.31 (CH_2Cl_2/MeOH = 7/1; UV and $KMnO_4$)

M.p. 75 - 82 °C decomp.

^{1}H NMR (300.36 MHz, DMSO-d^6) δ = 8.59 (t, 1H, N-H), 8.23 (d, $^3J_{H,H}$ = 8.3 Hz, 2H, H-8, H-12), 8.16 (d, $^3J_{H,H}$ = 7.5 Hz, 2H, H-4, H-6), 7.82 (d, $^3J_{H,H}$ = 8.3 Hz, 2H, H-9, H-11), 7.58 (t, $^3J_{H,H}$ = 7.3 Hz, 1H, H-2), 7.46 (t, $^3J_{H,H}$ = 7.6 Hz, 2H, H-1, H-3), 3.32 – 3.16 (m, 4H, H-14, H-23), 1.49 (m, $^3J_{H,H}$ = 6.2 Hz, 4H, H-15, H-22), 1.25 (s, 12H, H-16 to H-21) ppm.

^{13}C NMR (75.53 MHz, DMSO-d^6) δ = 164.9 (C_q, C-13), 137.1 (C_q, C-10), 134.8 (CH, C-4, C-6), 134.7 (CH, C-8, C-12), 131.3 (CH, C-1, C-3), 131.2 (CH, C-2), 129.7 (CH, C-9, C-11), 122.9 (C_q, C-7), 120.3, 50.6 (CH_2, C-23), 28.9 (CH_2), 28.8 (CH_2), 28.7 (CH_2), 28.5 (CH_2), 28.2 (CH_2), 26.4 (CH_2), 26.1 (CH_2) ppm.

^{19}F NMR (470.53 MHz, DMSO-d^6) δ = -77.7 ppm.

HRMS calcd (M/z) for $C_{23}H_{30}IN_4O^+$ [M-OTf]$^+$: 505.1459; found: 505.1458.

(4-((2-(2-(2-(2-Azidoethoxy)ethoxy)ethoxy)ethyl)carbamoyl)phenyl)(phenyl)iodonium trifluoromethanesulfonate (3ab)

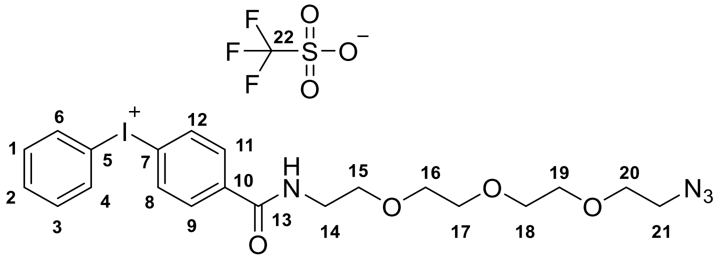

3ab

Starting from (4-carboxyphenyl)(phenyl)iodonium trifluoromethanesulfonate (**1a**) (204 mg, 0.429 mmol) and 2-(2-(2-(2-azidoethoxy)ethoxy)ethoxy)ethan-1-amine (**2b**) (113 mg, 0.520 mmol) synthesis of (4-((2-(2-(2-(2-azidoethoxy)ethoxy)ethoxy)ethyl)carbamoyl)phenyl)(phenyl)iodonium trifluoromethanesulfonate (**3ab**) was executed as described in general procedure A. The crude product purified via column chromatography (100 mL silica gel, CH_2Cl_2/MeOH = 24/1, fraction size: 20 mL).

Yield 73 mg (0.109 mmol, 25%), off-white amorphous solid, $C_{22}H_{26}F_3IN_4O_7S$ [674.43]

TLC R_f = 0.31 (CH_2Cl_2/MeOH = 7/1; UV and $KMnO_4$)

^{1}H NMR (300.36 MHz, DMSO-d^6) δ = 8.71 (s, 1H, N-H), 8.26 (d, $^3J_{H,H}$ = 8.4 Hz, 2H, H-8, H-12), 8.18 (d, $^3J_{H,H}$ = 7.4 Hz, 2H, H-4, H-6), 7.85 (d, $^3J_{H,H}$ = 8.4 Hz, 2H, H-9, H-11), 7.60 (t, $^3J_{H,H}$ = 7.3 Hz, 1H, H-2), 7.48 (t, $^3J_{H,H}$ = 7.6 Hz, 2H, H-1, H-3), 3.60 – 3.47 (m, 12H, H-15 to H-20), 3.44 – 3.36 (m, 4H, H-14, H-21) ppm.

^{13}C NMR (75.53 MHz, DMSO-d^6) δ = 165.2 (C_q, C-13), 136.8 (C_q, C-10), 134.8 (CH, C-4, C-6), 134.8 (CH, C-8, C-12), 131.3 (CH, C-1, C-3), 131.3 (CH, C-2) 129.7 (CH, C-9, C-11), 122.9 (C_q, C-7), 120.2 (C_q, C-5), 69.8 (CH_2), 69.7 (CH_2), 69.6 (CH_2), 69.2 (CH_2), 68.7 (CH_2), 50.0 (CH_2, C-21) ppm.

^{19}F NMR (470.53 MHz, DMSO-d^6) δ = -77.7 ppm.

HRMS calcd (M/z) for $C_{21}H_{26}IN_4O_4^+$ $[M-OTf]^+$: 525.0993; found: 525.0993.

(3-((10-Azidodecyl)carbamoyl)phenyl)(phenyl)iodonium trifluoromethanesulfonate (3ba)

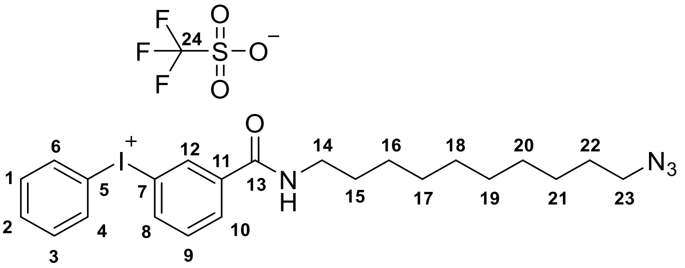

3ba

Starting from (3-carboxyphenyl)(phenyl)iodonium trifluoromethanesulfonate (**1b**) (199 mg, 0.420 mmol) and 10-azidobutan-1-amine (**2a**) (105 mg, 0.540 mmol) synthesis of (3-((10-azidodecyl)carbamoyl)phenyl)(phenyl)iodonium trifluoromethanesulfonate (**3ba**) was executed as described in general procedure A.

Yield 133 mg (0.203 mmol, 48%), colorless solid, $C_{24}H_{30}F_3IN_4O_4S$ [654.49]

TLC R_f = 0.34 (CH_2Cl_2/MeOH = 7/1; UV and $KMnO_4$)

M.p. 125 - 135 °C decomp.

^{1}H NMR (300.36 MHz, DMSO-d^6) δ = 8.66 (s, 1H, N-H), 8.60 (s, 1H, H-12), 8.30 (d, $^3J_{H,H}$ = 7.8 Hz, 1H, H-8), 8.18 (d, $^3J_{H,H}$ = 7.7 Hz, 2H, H-4, H-6), 8.02 (d, $^3J_{H,H}$ = 7.7 Hz, 1H, H-10), 7.58 (m, $^3J_{H,H}$ = 13.9, 7.4 Hz, 2H, H-2, H-9), 7.48 (m, $^3J_{H,H}$ = 15.4, 7.6 Hz, 2H, H-1, H-3), 3.32 – 3.22 (m, $^3J_{H,H}$ = 15.3, 6.4 Hz, 4H, H-14, H-23), 1.50 (s, 4H, H-15, H-22), 1.26 (s, 12H, H-16 to H-21) ppm.

^{13}C NMR (75.53 MHz, DMSO-d^6) δ = 164.1 (C_q, C-13), 137.1 (CH, C-8), 134.9 (CH, C-4, C-6), 133.8 (CH, C-12), 131.3 (CH, C-1, C-3), 131.3 (CH, C-2), 131.1 (CH, C-9), 129.5 (CH, C-10), 120.3 (C_q, C-5), 120.1 (C_q, C-7), 50.6 (CH_2, C-23), 39.4 (CH_2, C-14), 28.9 (CH_2), 28.8 (CH_2), 28.7 (CH_2), 28.5 (CH_2), 28.2 (CH_2), 26.4 (CH_2), 26.1 (CH_2) ppm.

^{19}F NMR (470.53 MHz, DMSO-d^6) δ = -77.7 ppm.

HRMS calcd (M/z) for $C_{23}H_{30}IN_4O^+$ [M-OTf]$^+$: 505.1459; found: 505.1459.

(3-((2-(2-(2-(2-Azidoethoxy)ethoxy)ethoxy)ethyl)carbamoyl)phenyl)(phenyl)iodonium trifluoromethanesulfonate (3bb)

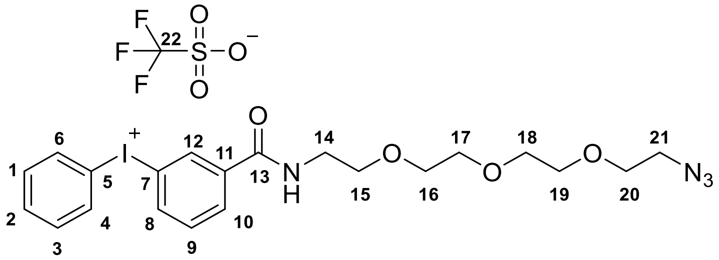

3bb

Starting from (3-carboxyphenyl)(phenyl)iodonium trifluoromethanesulfonate (**1b**) (198 mg, 0.418 mmol) and 2-(2-(2-(2-azidoethoxy)ethoxy)ethoxy)ethan-1-amine (**2b**) (111 mg, 0.508 mmol) synthesis of (3-((2-(2-(2-(2-azidoethoxy)ethoxy)ethoxy)ethyl)carbamoyl)phenyl)(phenyl)iodonium trifluoromethanesulfonate (**3bb**) was executed as described in general procedure A.

Yield 105 mg (0.156 mmol, 37%), off-white amorphous solid, $C_{22}H_{26}F_3IN_4O_7S$ [674.43]

TLC R_f = 0.28 (CH_2Cl_2/MeOH = 7/1; UV and $KMnO_4$)

^{1}H NMR (300.36 MHz, DMSO-d^6) δ = 8.76 (s, 1H), 8.62 (s, 1H, H-12), 8.31 (d, $^3J_{H,H}$ = 7.9 Hz, 1H, H-8), 8.18 (d, $^3J_{H,H}$ = 7.7 Hz, 2H, H-4, H-6), 8.03 (d, $^3J_{H,H}$ = 7.7 Hz, 1H, H-10), 7.57 (dt, $^3J_{H,H}$ = 12.0, 7.6 Hz, 2H, H-2, H-9), 7.46 (t, $^3J_{H,H}$ = 7.7 Hz, 2H, H-1, H-3), 3.61 – 3.48 (m, 12H, H-15 to H-20), 3.45 – 3.40 (m, 2H, H-14), 3.37 (m, 2H, H-21) ppm.

^{13}C NMR (75.53 MHz, DMSO-d^6) δ = 164.3 (C_q, C-13), 137.2 (CH, C-8), 136.8 (C_q, C-), 134.8 (CH, C-4, C-6), 133.8 (CH, C-12), 131.3 (CH, C-1, C-3), 131.2 (CH, C-2), 131.1 (CH, C-9), 129.6 (CH, C-10), 120.4 (C_q, C-aromatic), 120.2 (C_q, C-aromatic), 69.8 (CH_2), 69.7 (CH_2), 69.6 (CH_2), 69.2 (CH_2), 68.7 (CH_2), 50.0 (CH_2, C-21), 39.4 (CH_2, C-14) ppm.

^{19}F NMR (470.53 MHz, DMSO-d^6) δ = -77.7 ppm.

HRMS calcd (M/z) for $C_{21}H_{26}IN_4O_4^+$ [M-OTf]$^+$: 525.0993; found: 525.0992.

2-((10-Azidodecyl)carbamoyl)dibenzo[*b,d*]iodol-5-ium trifluoromethanesulfonate (3ca)

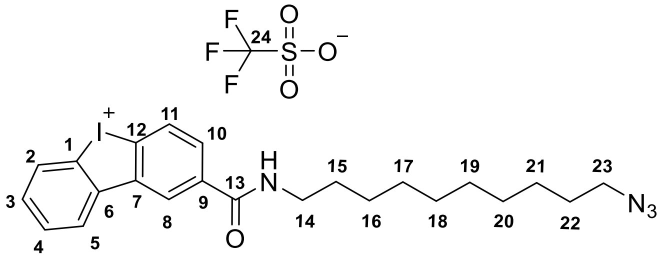

3ca

Starting from 2-carboxydibenzo[*b,d*]iodol-5-ium trifluoromethanesulfonate (**1c**) (202 mg, 0.428 mmol) and 10-azidodecan-1-amine (**2a**) (109 mg, 0.550 mmol) synthesis of 2-((10-azidodecyl)carbamoyl)dibenzo[*b,d*]iodol-5-ium trifluoromethanesulfonate (**3ca**) was executed as described in general procedure A.

Yield 137 mg (0.210 mmol, 49%), colorless solid, $C_{24}H_{28}F_3IN_4O_4S$ [652.47]

TLC R_f = 0.27 (CH_2Cl_2/MeOH = 10/1; UV and $KMnO_4$)

M.p. 200 – 204 °C

^{1}H NMR (300.36 MHz, DMSO-d^6) δ = 8.79 (s, 1H, H-8), 8.75 (s, 1H, N-H), 8.60 (d, $^3J_{H,H}$ = 8.1 Hz, 2H, H-2, H-11), 8.44 (d, $^3J_{H,H}$ = 7.3 Hz, 1H, H-5), 8.04 (d, $^3J_{H,H}$ = 8.1 Hz, 1H, H-10), 7.84 (t, $^3J_{H,H}$ = 7.1 Hz, 1H, H-4), 7.68 (t, $^3J_{H,H}$ = 7.1 Hz, 1H, H-3), 3.33 – 3.23 (m, 4H, H-14, H-23), 1.55 (d, $^3J_{H,H}$ = 5.9 Hz, 2H, H-15), 1.50 (d, $^3J_{H,H}$ = 6.2 Hz, 2H, H-22), 1.25 (s, 12H, H-16 to H-21) ppm.

^{13}C NMR (75.53 MHz, DMSO-d^6) δ = 164.9 (C_q, C-13), 141.4 (C_q, C-7), 140.8 (C_q, C-6), 136.6 (C_q, C-9), 130.7 (CH, C-3, C-11), 130.5 (CH, C-2), 130.3 (CH, C-4), 128.8 (CH, C-10), 126.9 (C_q, C-12), 126.4 (CH, C-5), 124.5 (C_q, C-1), 124.3 (CH, C-8), 50.6 (CH_2, C-23), 29.0 (CH_2), 28.8 (CH_2), 28.7 (CH_2), 28.5 (CH_2), 28.2 (CH_2), 26.5 (CH_2), 26.1 (CH_2) ppm.

^{19}F NMR (376.17 MHz, DMSO-d^6) δ = -77.7 ppm.

HRMS calcd (M/z) for $C_{23}H_{28}IN_4O^+$ [M-OTf]$^+$: 503.1302; found: 503.1298.

2-((2-(2-(2-(2-Azidoethoxy)ethoxy)ethoxy)ethyl)carbamoyl)dibenzo[*b,d*]iodol-5-ium trifluoromethanesulfonate (3cb)

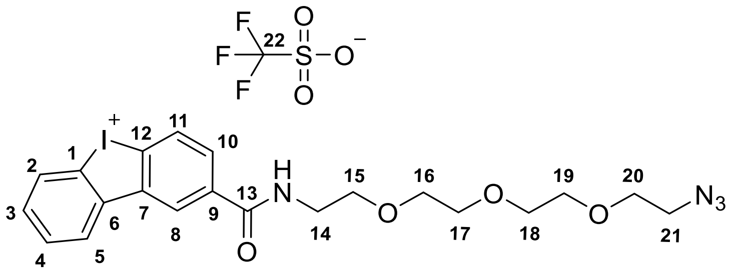

3cb

Starting from 2-carboxydibenzo[*b*,*d*]iodol-5-ium trifluoromethanesulfonate (**1c**) (201 mg, 0.426 mmol) and 2-(2-(2-(2-azidoethoxy)ethoxy)ethoxy)ethan-1-amine (**2c**) (111 mg, 0.509 mmol) synthesis of 2-((2-(2-(2-(2-azidoethoxy)ethoxy)ethoxy)ethyl)carbamoyl)dibenzo[*b*,*d*]iodol-5-ium trifluoromethanesulfonate (**3cb**) was executed as described in general procedure A.

Yield 137 mg (0.204 mmol, 48%), off-white solid, $C_{22}H_{24}F_3IN_4O_7S$ [672.41]

TLC R_f = 0.29 (CH_2Cl_2/MeOH = 10/1; UV and $KMnO_4$)

M.p. 162 – 165 °C

^{1}H NMR (300.36 MHz, DMSO-d^6) δ = 8.87 (s, 1H, N-H), 8.82 (s, 1H, H-8), 8.66 – 8.56 (m, 2H, H-2, H-11), 8.44 (d, $^3J_{H,H}$ = 7.8 Hz, 1H, H-5), 8.05 (d, $^3J_{H,H}$ = 8.4 Hz, 1H, H-10), 7.84 (t, $^3J_{H,H}$ = 7.4 Hz, 1H, H-4), 7.69 (t, $^3J_{H,H}$ = 7.4 Hz, 1H, H-3), 3.63 – 3.47 (m, 14H), 3.36 (s, 2H, H-21) ppm.

^{13}C NMR (75.53 MHz, DMSO-d^6) δ = 165.1 (C_q, C-13), 141.4 (C_q, C-7), 140.8 (C_q, C-6), 136.3 (C_q, C-9), 130.7 (CH, C-3, C-11), 130.6 (CH, C-2), 130.3 (CH, C-4), 128.8 (CH, C-10), 127.1 (C_q, C-12), 126.3 (CH, C-5), 124.6 (C_q, C-1), 124.4 (CH, C-8), 69.8 (CH_2), 69.7 (CH_2), 69.2 (CH_2), 68.9 (CH_2), 49.9 (CH_2, C-21) ppm.

^{19}F NMR (376.17 MHz, DMSO-d^6) δ = -77.7 ppm.

HRMS calcd (M/z) for $C_{21}H_{24}IN_4O_4^+$ $[M-OTf]^+$: 523.0837; found: 523.0840.

3-((10-Azidodecyl)carbamoyl)dibenzo[*b,d*]iodol-5-ium trifluoromethanesulfonate (3da)

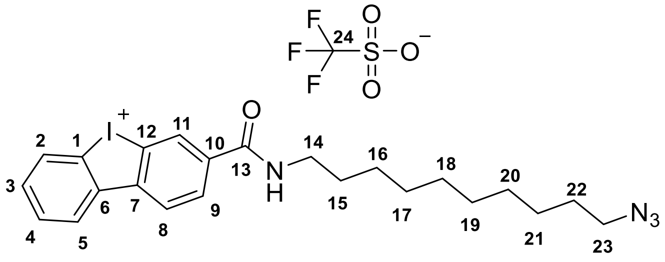

3da

Starting from 3-carboxydibenzo[*b,d*]iodol-5-ium trifluoromethanesulfonate (**1d**) (201 mg, 0.425 mmol) and 10-azidodecan-1-amine (**2a**) (101 mg, 0.511 mmol) synthesis of 3-((10-azidodecyl)carbamoyl)dibenzo[*b,d*]iodol-5-ium trifluoromethanesulfonate (**3da**) was executed as described in general procedure A.

Yield 111 mg (0.170 mmol, 40%), off-white solid, $C_{24}H_{28}F_3IN_4O_4S$ [652.47]

TLC R_f = 0.40 (CH_2Cl_2/MeOH = 10/1; UV and $KMnO_4$)

M.p. 194 - 198 °C decomp.

^{1}H NMR (300.36 MHz, DMSO-d^6) δ = 8.90 (d, $^4J_{H,H}$ = 1.1 Hz, 1H, H-11), 8.74 – 8.63 (m, 2H, N-H, H-2), 8.50 (d, $^3J_{H,H}$ = 8.1 Hz, 2H, H-5, H-8), 8.22 – 8.12 (m, 1H, H-9), 7.83 (t, $^3J_{H,H}$ = 7.1 Hz, 1H, H-4), 7.70 (t, $^3J_{H,H}$ = 7.2 Hz, 1H, H-3), 3.32 – 3.23 (m, 4H, H-14, H-23), 1.51 (d, $^3J_{H,H}$ = 5.8 Hz, 4H, H-15, H-22), 1.26 (s, 12H, H-16 to H-21) ppm.

^{13}C NMR (75.53 MHz, DMSO-d^6) δ = 164.8 (C_q, C-13), 143.3 (C_q, C-7), 140.7 (C_q, C-6), 136.5 (C_q, C-10), 130.9 (CH, C-3), 130.8 (CH, C-2), 130.4 (CH, C-4), 130.2 (CH, C-11), 128.3 (CH, C-9), 126.9 (CH, C-8), 125.8 (CH, C-5), 125.0 (C_q, C-aromatic), 124.3 (C_q, C-aromatic), 50.6 (CH_2, C-23), 29.0 (CH_2), 28.9 (CH_2), 28.9 (CH_2), 28.7 (CH_2), 28.5 (CH_2), 28.2 (CH_2), 26.5 (CH_2), 26.1 (CH_2) ppm.

^{19}F NMR (376.17 MHz, DMSO-d^6) δ = -77.7 ppm.

HRMS calcd (M/z) for $C_{23}H_{28}IN_4O^+$ [M-OTf]$^+$: 503.1302; found: 503.1299.

3-((2-(2-(2-(2-Azidoethoxy)ethoxy)ethoxy)ethyl)carbamoyl)dibenzo[*b,d*]iodol-5-ium trifluoromethanesulfonate (3db)

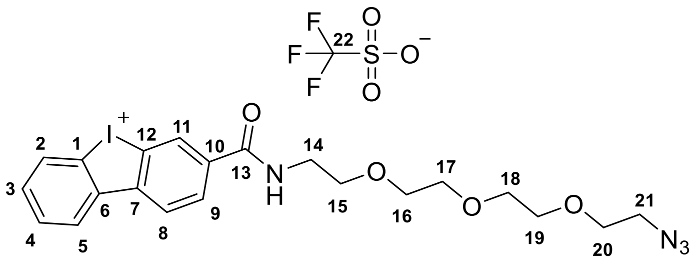

3db

Starting from 3-carboxydibenzo[*b,d*]iodol-5-ium trifluoromethanesulfonate (**1d**) (200 mg, 0.424 mmol) and 2-(2-(2-(2-azidoethoxy)ethoxy)ethoxy)ethan-1-amine (**2b**) (111 mg, 0.508 mmol) synthesis of 3-((2-(2-(2-(2-azidoethoxy)ethoxy)ethoxy)ethyl)carbamoyl)dibenzo[*b,d*]iodol-5-ium trifluoromethanesulfonate (**3db**) was executed as described in general procedure A.

Yield 155 mg (0.231 mmol, 54%), off-white solid, $C_{22}H_{24}F_3IN_4O_7S$ [672.41]

TLC R_f = 0.34 (CH_2Cl_2/MeOH = 10/1; UV and $KMnO_4$)

M.p. 125 – 130 °C

^{1}H NMR (300.36 MHz, DMSO-d^6) δ = 8.92 (s, 1H, H-11), 8.78 (s, 1H, N-H), 8.66 (d, $^3J_{H,H}$ = 8.0 Hz, 1H, H-2), 8.54 – 8.45 (m, 2H, H-5, H-8), 8.19 (d, $^3J_{H,H}$ = 7.8 Hz, 1H, H-9), 7.83 (t, $^3J_{H,H}$ = 7.1 Hz, 1H, H-4), 7.70 (t, $^3J_{H,H}$ = 7.3 Hz, 1H, H-3), 3.54 (m, 12H, H-15 to H-20), 3.46 (m, 2H, H-14), 3.37 (m, 2H, H-21) ppm.

^{13}C NMR (75.53 MHz, DMSO-d^6) δ = 165.0 (C_q, C-13), 143.5 (C_q, C-7), 140.6 (C_q, C-6), 136.1 (C_q, C-10), 130.9 (CH, C-3), 130.8 (CH, C-2), 130.4 (CH, C-4), 130.3 (CH, C-11), 128.4 (CH, C-9), 126.9 (CH, C-5), 125.8 (CH, C-8), 125.1 (C_q, C-1), 124.4 (C_q, C-12), 69.8 (CH_2), 69.7 (CH_2), 69.6 (CH_2), 69.2 (CH_2), 68.8 (CH_2), 50.0 (CH_2, C-21), 39.5 (CH_2, C-14) ppm.

^{19}F NMR (470.53 MHz, DMSO-d^6) δ = -77.7 ppm.

HRMS calcd (M/z) for $C_{21}H_{24}IN_4O_4^+$ [M-OTf]$^+$: 523.0837; found: 523.0840.

4-((10-Azidodecyl)carbamoyl)dibenzo[*b,d*]iodol-5-ium trifluoromethanesulfonate (3ea)

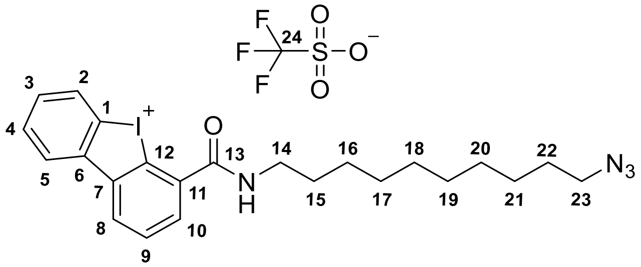

3ea

Starting from 4-carboxydibenzo[*b*,*d*]iodol-5-ium trifluoromethanesulfonate (**1e**) (198 mg, 0.420 mmol) and 10-azidodecan-1-amine (**2a**) (110 mg, 0.555 mmol) synthesis of 4-((10-azidodecyl)carbamoyl)dibenzo[*b,d*]iodol-5-ium trifluoromethanesulfonate (**3ea**) was executed as described in general procedure A. The crude product was purified via column chromatography (120 mL silica gel, CH_2Cl_2/MeOH = 24/1, fraction size: 20 mL).

Yield 93 mg (0.143 mmol, 34%), off-white solid, $C_{24}H_{28}F_3IN_4O_4S$ [652.47]

TLC R_f = 0.34 (CH_2Cl_2/MeOH = 10/1; UV and $KMnO_4$)

M.p. >170 °C decomp.

^{1}H NMR (300.36 MHz, DMSO-d^6) δ = 9.70 (s, 1H, N-H), 8.82 (d, $^3J_{H,H}$ = 7.9 Hz, 1H, H-2), 8.69 (d, $^3J_{H,H}$ = 7.6 Hz, 1H, H-8), 8.53 (d, $^3J_{H,H}$ = 7.5 Hz, 1H, H-5), 8.45 (d, $^3J_{H,H}$ = 7.6 Hz, 1H, H-10), 8.03 (t, $^3J_{H,H}$ = 7.7 Hz, 1H, H-9), 7.85 (t, $^3J_{H,H}$ = 7.2 Hz, 1H, H-4), 7.72 (t, $^3J_{H,H}$ = 7.2 Hz, 1H, H-3), 3.42 (dd, $^3J_{H,H}$ = 12.1, 6.4 Hz, 2H, H-14), 3.28 (t, $^3J_{H,H}$ = 6.8 Hz, 2H, H-23), 1.60 (m, 2H, H-15), 1.53 – 1.44 (m, 2H, H-22), 1.28 (m, 12H, H-16 to H-21) ppm.

^{13}C NMR (75.53 MHz, DMSO-d^6) δ = 163.7 (C_q, C-13), 141.6 (C_q, C-7), 139.3 (C_q, C-6), 131.2 (CH, C-9), 131.0 (CH, C-2), 130.6 (CH, C-3), 130.4 (CH, C-4), 129.4 (C_q, C-11), 129.2 (CH, C-8), 127.4 (CH, C-10), 126.6 (CH, C-5), 122.3 (C_q, C-aromatic), 122.2 (C_q, C-aromatic), 50.6 (CH_2, C-23), 40.5 (CH_2, C-14), 28.8 (CH_2), 28.6 (CH_2), 28.5 (CH_2), 28.5 (CH_2), 28.2 (CH_2), 26.4 (CH_2), 26.1 (CH_2) ppm.

^{19}F NMR (470.53 MHz, DMSO-d^6) δ = -77.7 ppm.

HRMS calcd (M/z) for $C_{23}H_{28}IN_4O^+$ [M-OTf]$^+$: 503.1302; found: 503.1302.

4-((2-(2-(2-(2-Azidoethoxy)ethoxy)ethoxy)ethyl)carbamoyl)dibenzo[*b,d*]iodol-5-ium trifluoromethanesulfonate (3eb)

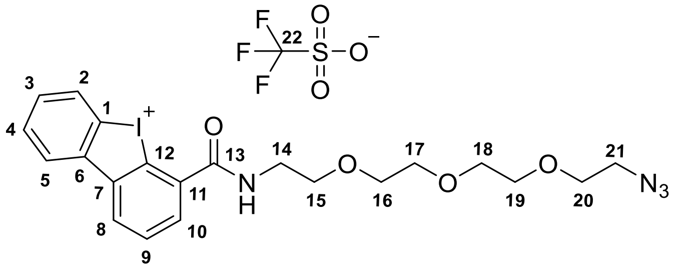

3eb

Starting from 4-carboxydibenzo[*b,d*]iodol-5-ium trifluoromethanesulfonate (**1e**) (205 mg, 0.425 mmol) and 2-(2-(2-(2-azidoethoxy)ethoxy)ethoxy)ethan-1-amine (**2b**) (116 mg, 0.511 mmol) synthesis of 4-((2-(2-(2-(2-azidoethoxy)ethoxy)ethoxy)ethyl)carbamoyl)dibenzo[*b,d*]iodol-5-ium trifluoromethanesulfonate (**3eb**) was executed as described in general procedure A.

Yield 178 mg (0.265 mmol, 61%), off-white solid, $C_{22}H_{24}F_3IN_4O_7S$ [672.41]

TLC R_f = 0.40 (CH_2Cl_2/MeOH = 10/1; UV and $KMnO_4$)

M.p. 148 – 150 °C

^{1}H NMR (300.36 MHz, DMSO-d^6) δ = 9.81 (s, 1H, N-H), 8.77 (d, $^3J_{H,H}$ = 8.0 Hz, 1H, H-2), 8.69 (d, $^3J_{H,H}$ = 7.6 Hz, 1H, H-8), 8.52 (d, $^3J_{H,H}$ = 7.5 Hz, 1H, H-5), 8.47 (d, $^3J_{H,H}$ = 7.6 Hz, 1H, H-10), 8.03 (t, $^3J_{H,H}$ = 7.6 Hz, 1H, H-9), 7.85 (t, $^3J_{H,H}$ = 7.3 Hz, 1H, H-4), 7.72 (t, $^3J_{H,H}$ = 7.5 Hz, 1H, H-3), 3.71 – 3.46 (m, 14H, H-14 to H-20), 3.33 (s, 2H, H-21) ppm.

^{13}C NMR (75.53 MHz, DMSO-d^6) δ = 164.1 (C_q, C-13), 141.6 (C_q, C-7), 139.2 (C_q, C-6), 131.3 (CH, C-9), 131.0 (CH, C-2), 130.7 (CH, C-3), 130.5 (CH, C-4), 129.3 (CH, C-8), 129.3 (C_q, C-11), 127.5 (CH, C-10), 126.6 (CH, C-5), 122.2 (C_q, C-12), 122.1 (C_q, C-1), 69.8 (CH_2), 69.7 (CH_2), 69.2 (CH_2), 68.3 (CH_2), 49.9 (CH_2, C-21), 40.5 (CH_2, C-14) ppm.

^{19}F NMR (470.53 MHz, DMSO-d^6) δ = -77.7 ppm.

HRMS calcd (M/z) for $C_{21}H_{24}IN_4O_4^+$ [M-OTf]$^+$: 523.0837; found: 523.0835.

2-((10-Azidodecyl)carbamoyl)dibenzo[*b*,*d*]bromol-5-ium trifluoromethanesulfonate (3fa)

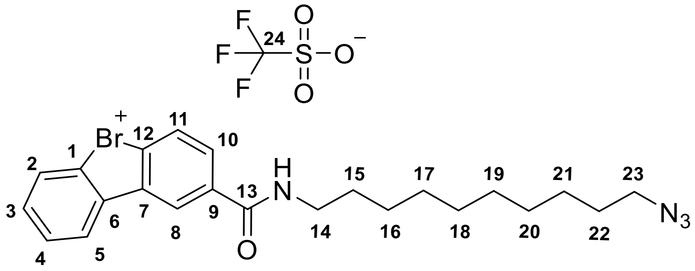

3fa

Starting from 2-carboxydibenzo[*b*,*d*]bromol-5-ium trifluoromethanesulfonate (**1f**) (180 mg, 0.423 mmol) and 10-azidodecan-1-amine (**2a**) (97.0 mg, 0.489 mmol) synthesis of 2-((10-azidodecyl)carbamoyl)dibenzo[*b*,*d*]bromol-5-ium trifluoromethanesulfonate (**3fa**) was executed as described in general procedure A. The crude product was purified via column chromatography (120 mL silica gel, 12 x 3.5 cm, CH_2Cl_2/MeOH = 10/1, fraction size: 20 mL).

Yield 139 mg (0.230 mmol, 54%), ochre amorphous solid, $C_{24}H_{28}BrF_3N_4O_4S$ [605.47]

TLC R_f = 0.39 (CH_2Cl_2/MeOH = 7/1; UV and $KMnO_4$)

^{1}H NMR (300.13 MHz, DMSO-d^6) δ = 8.95 (d, $^4J_{H,H}$ = 1.2 Hz, 1H, H-8), 8.84 (t, $^3J_{H,H}$ = 5.2 Hz, 1H, N-H), 8.71 (t, $^3J_{H,H}$ = 8.8 Hz, 2H, H-2, H-11), 8.59 (d, $^3J_{H,H}$ = 7.2 Hz, 1H, H-5), 8.19 (dd, $^3J_{H,H}$ = 8.8, $^4J_{H,H}$ = 1.2 Hz, 1H, H-10), 7.96 (t, $^3J_{H,H}$ = 7.4 Hz, 1H, H-4), 7.85 (t, $^3J_{H,H}$ = 7.5 Hz, 1H, H-3), 3.32 – 3.25 (m, 4H, H-14, H-23), 1.61 – 1.46 (m, 4H, H-15, H-22), 1.29 (m, 12H, H-16 to H-21) ppm.

^{13}C NMR (75.47 MHz, DMSO-d^6) δ = 164.4 (C_q, C-13), 138.6 (C_q, C-12), 137.7 (C_q, C-1), 137.4 (C_q, C-9), 135.5 (C_q, C-7), 134.9 (C_q, C-6), 131.9 (CH, C-3), 131.2 (CH, C-4), 130.1 (CH, C-10), 126.2 (CH, C-5), 125.9 (CH, C-aromatic), 125.8 (CH, C-aromatic), 124.4 (CH, C-8), 50.6 (CH_2, C-23), 29.0 (CH_2), 28.9 (CH_2), 28.8 (CH_2), 28.5 (CH_2), 28.2 (CH_2), 26.5 (CH_2), 26.2 (CH_2) ppm.

^{19}F NMR (282.40 MHz, DMSO-d^6) δ = -77.8 ppm.

HRMS calcd (M/z) for $C_{23}H_{28}BrN_4O^+$ [M-OTf]$^+$: 455.1441; found: 455.1444.

2-((2-(2-(2-(2-Azidoethoxy)ethoxy)ethoxy)ethyl)carbamoyl)dibenzo[*b,d*]bromol-5-ium trifluoromethanesulfonate (3fb)

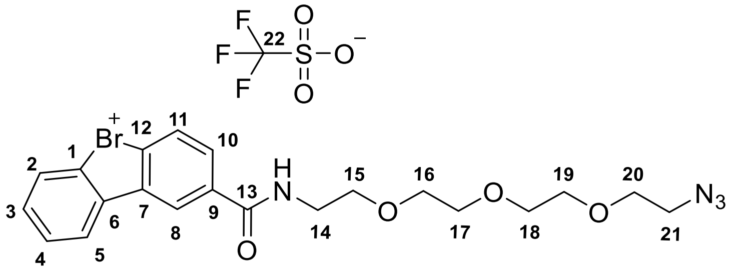

3fb

Starting from 2-carboxydibenzo[*b,d*]bromol-5-ium trifluoromethanesulfonate (**1f**) (180 mg, 0.423 mmol) and 2-(2-(2-(2-azidoethoxy)ethoxy)ethoxy)ethan-1-amine (**2b**) (112 mg, 0.513 mmol) synthesis of 2-((2-(2-(2-(2-azidoethoxy)ethoxy)ethoxy)ethyl)carbamoyl)dibenzo[*b,d*]bromol-5-ium trifluoromethanesulfonate (**3fb**) was executed as described in general procedure A. The crude product was purified by dissolving it in 20 mL CH_2Cl_2 and washing the resulting solution with 1 M aq. HCl (8 x 20 mL) to remove remaining DIPEA. The combined aqueous layers were then extracted with CH_2Cl_2 (3 x 50 mL). The combined organic layers were dried over Na_2SO_4, filtered and the solvent was removed under reduced pressure.

Yield 78 mg (0.125 mmol, 30%), off-white amorphous solid, $C_{22}H_{24}BrF_3N_4O_7S$ [625.41]

HPLC-MS t_R = min (method: *2-100-MeCN*); *m/z* (ESI+) = 475, 477 $[M\text{-}OTf]^+$

^{1}H NMR (300.13 MHz, DMSO-d^6) δ = 9.01 (s, 2H, H-8, N-H), 8.87 (t, $^3J_{H,H}$ = 8.8 Hz, 2H, H-2, H-11), 8.58 (d, $^3J_{H,H}$ = 7.5 Hz, 1H, H-5), 8.19 (dd, $^3J_{H,H}$ = 8.8, $^4J_{H,H}$ = 1.3 Hz, 1H, H-10), 7.94 (t, $^3J_{H,H}$ = 7.4 Hz, 1H, H-4), 7.83 (t, $^3J_{H,H}$ = 7.9 Hz, 1H, H-3), 3.56 (m, 14H, H-14 to H-20), 3.36 (d, $^3J_{H,H}$ = 5.3 Hz, 2H, H-21) ppm.

^{13}C NMR (75.47 MHz, DMSO-d^6) δ = 164.7 (C_q, C-13), 139.2 (C_q, C-12), 138.1 (C_q, C-1), 136.9 (C_q, C-9), 135.5 (C_q, C-7), 134.8 (C_q, C-6), 131.6 (CH, C-3), 131.1 (CH, C-4), 129.9 (CH, C-10), 126.2 (CH, C-aromatic), 126.1 (CH, C-aromatic), 126.0 (CH, C-aromatic), 124.4 (CH, C-8), 69.8 (CH_2), 69.7 (CH_2), 69.2 (CH_2), 68.8 (CH_2), 50.0 (CH_2, C-21) ppm.

^{19}F NMR (282.40 MHz, DMSO-d^6) δ = -77.8 ppm.

HRMS calcd (M/z) for $C_{21}H_{24}BrN_4O_4^+$ [M-OTf]$^+$: 475.0975; found: 475.0975.

3-((10-Azidodecyl)carbamoyl)dibenzo[*b,d*]bromol-5-ium trifluoromethanesulfonate (3ga)

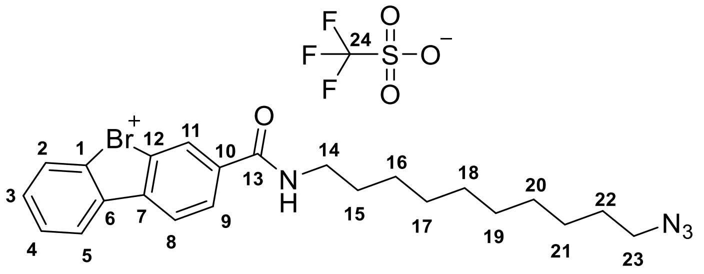

3ga

Starting from 3-carboxydibenzo[*b,d*]bromol-5-ium trifluoromethanesulfonate (**1g**) (180 mg, 0.423 mmol) and 10-azidodecan-1-amine (**2a**) (97.4 mg, 0.491 mmol) synthesis of 3-((10-azidodecyl)carbamoyl)dibenzo[*b,d*]bromol-5-ium trifluoromethanesulfonate (**3ga**) was executed as described in general procedure A. The crude product was purified via column chromatography (120 mL silica gel, 12 x 3.5 cm, CH_2Cl_2/MeOH = 10/1, fraction size: 20 mL).

Yield 158 mg (0.261 mmol, 62%), ochre gum, $C_{24}H_{28}BrF_3N_4O_4S$ [605.47]

TLC R_f = 0.37 (CH_2Cl_2/MeOH = 10/1; UV and $KMnO_4$)

^{1}H NMR (300.13 MHz, DMSO-d^6) δ = 8.97 (d, $^4J_{H,H}$ = 1.0 Hz, 1H, H-11), 8.87 (t, $^3J_{H,H}$ = 5.3 Hz, 1H, N-H), 8.68 (d, $^3J_{H,H}$ = 8.3 Hz, 1H, H-8), 8.65 (dd, $^3J_{H,H}$ = 7.9, 1.3 Hz, 1H, H-5), 8.56 (d, $^3J_{H,H}$ = 8.1 Hz, 1H, H-2), 8.35 (dd, $^3J_{H,H}$ = 8.2, $^4J_{H,H}$ = 1.0 Hz, 1H, H-9), 7.97 (t, $^3J_{H,H}$ = 7.2 Hz, 1H, H-4), 7.92 – 7.84 (m, 1H, H-3), 3.30 (t, $^3J_{H,H}$ = 6.9 Hz, 4H, H-14, H-23), 1.61 – 1.45 (m, 4H, H-15, H-22), 1.27 (s, 1H, 12H, H-16 to H-21) ppm.

^{13}C NMR (75.47 MHz, DMSO-d^6) δ = 163.8 (C_q, C-13), 137.5 (C_q, C-aromatic), 137.5 (C_q, C-aromatic), 137.3 (C_q, C-aromatic), 136.9 (C_q, C-aromatic), 134.7 (C_q, C-6), 132.4 (CH, C-3), 131.3 (CH, C-4), 129.1 (CH, C-9), 126.8 (CH, C-5), 125.9 (CH, C-8), 125.8 (CH, C-2), 125.4 (CH, C-11), 50.6 (CH_2, C-23), 29.0 (CH_2), 28.9 (CH_2), 28.9 (CH_2), 28.7 (CH_2), 28.5 (CH_2), 28.2 (CH_2), 26.5 (CH_2), 26.2 (CH_2) ppm.

^{19}F NMR (282.40 MHz, DMSO-d^6) δ = -77.8 ppm.

HRMS calcd (M/z) for $C_{23}H_{28}BrN_4O^+$ [M-OTf]$^+$: 455.1441; found: 455.1444.

3-((2-(2-(2-(2-Azidoethoxy)ethoxy)ethoxy)ethyl)carbamoyl)dibenzo[*b,d*]bromol-5-ium trifluoromethanesulfonate (3gb)

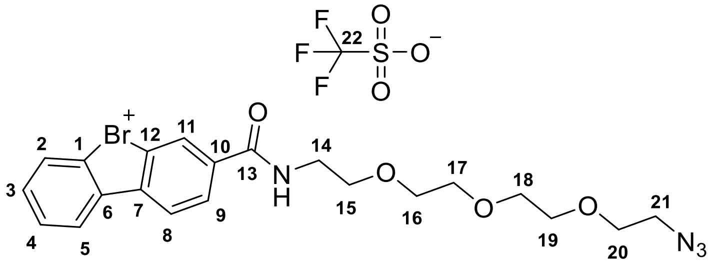

3gb

Starting from 3-carboxydibenzo[*b,d*]bromol-5-ium trifluoromethanesulfonate (**1g**) (180 mg, 0.423 mmol) and 2-(2-(2-(2-azidoethoxy)ethoxy)ethoxy)ethan-1-amine (**2b**) (106 mg, 0.486 mmol) synthesis of 2-((2-(2-(2-(2-azidoethoxy)ethoxy)ethoxy)ethyl)carbamoyl)dibenzo[*b,d*]bromol-5-ium trifluoromethanesulfonate (**3gb**) was executed as described in general procedure A. The crude product was purified via column chromatography (50 mL silica gel, 10 x 2.5 cm, CH_2Cl_2/MeOH = 10/1 to 4/1, fraction size: 10 mL).

Yield 92 mg (0.147 mmol, 35%), off-white solid, $C_{22}H_{24}BrF_3N_4O_7S$ [625.41]

HPLC-MS t_R = 4.11 min (method: *2-100-MeCN*); *m/z* (ESI+) = 475, 477 [M-OTf]$^+$

TLC R_f = 0.26 (CH_2Cl_2/MeOH = 7/1; UV and $KMnO_4$)

M.p.

^{1}H NMR (300.13 MHz, DMSO-d^6) δ = 9.19 (s, 1H, H-11), 8.96 (t, $^3J_{H,H}$ = 5.2 Hz, 1H, N-H), 8.90 (d, $^3J_{H,H}$ = 8.5 Hz, 1H, H-2), 8.64 (d, $^3J_{H,H}$ = 8.2 Hz, 1H, H-8), 8.61 (d, $^3J_{H,H}$ = 8.0 Hz, 1H, H-5), 8.33 (d, $^3J_{H,H}$ = 8.1 Hz, 1H, H-9), 7.93 (t, $^3J_{H,H}$ = 7.4 Hz, 1H, H-4), 7.84 (t, $^3J_{H,H}$ = 7.3 Hz, 1H, H-3), 3.62 – 3.52 (m, 12H, H-15 to H-20), 3.51 – 3.45 (m, 2H, H-14), 3.38 (s, 2H, H-21) ppm.

^{13}C NMR (75.47 MHz, DMSO-d^6) δ = 164.3 (C_q, C-13), 138.2 (C_q, C-1), 137.8 (C_q, C-12), 137.5 (C_q, C-7), 137.0 (C_q, C-10), 134.7 (C_q, C-6), 131.9 (CH, C-3), 131.2 (CH, C-4), 129.1 (CH, C-9), 126.5 (CH, C-5), 126.3 (CH, C-2), 125.8 (CH, C-11), 125.5 (CH, C-8), 69.8 (CH_2), 69.7 (CH_2), 69.7 (CH_2), 69.3 (CH_2), 68.8 (CH_2), 50.0 (CH_2, C-21) ppm.

^{19}F NMR (282.40 MHz, DMSO-d^6) δ = -77.8 ppm.

HRMS calcd (M/z) for $C_{21}H_{24}BrN_4O_4^+$ [M-OTf]$^+$: 475.0975; found: 475.0977.

2-((10-Azidodecyl)carbamoyl)dibenzo[*b,d*]chlorol-5-ium trifluoromethanesulfonate (3ha)

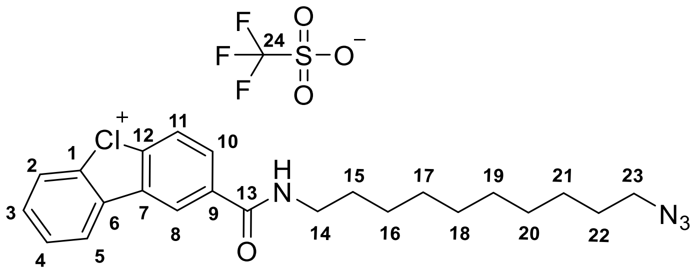

3ha

Starting from 2-carboxydibenzo[*b,d*]chlorol-5-ium trifluoromethanesulfonate (**1h**) (86.4 mg, 0.227 mmol) and 10-azidodecan-1-amine (**2a**) (30.0 mg, 0.151 mmol) synthesis of 2-((10-azidodecyl)carbamoyl)dibenzo[*b,d*]chlorol-5-ium trifluoromethanesulfonate (**3ha**) was executed as described in general procedure B.

Yield 55.3 mg (98.6 µmol, 65%), off-white solid, $C_{24}H_{28}ClF_3N_4O_4S$ [561.02]

HPLC-MS t_R = 6.15 min (method: *2-100-MeCN*); *m/z* (ESI+) = 411 [M-OTf]$^+$

TLC R_f = 0.23 (CH_2Cl_2/MeOH = 7/1; UV and $KMnO_4$)

M.p. 115 – 118 °C

^{1}H NMR (300.36 MHz, $CDCl_3$) δ = 8.76 (d, $^4J_{H,H}$ = 1.3 Hz, 1H, H-8), 8.40 (t, $^3J_{H,H}$ = 9.8 Hz, 3H, H-2, H-5, H-10), 8.24 (dd, $^3J_{H,H}$ = 9.1, $^4J_{H,H}$ = 1.5 Hz, 1H, H-10), 7.92 (t, $^3J_{HH}$ = 4.9 Hz, 1H, N-H), 7.79 – 7.64 (m, 2H, H-3, H-4), 3.43 (dd, $^3J_{H,H}$ = 12.5, 6.2 Hz, 2H, H-14), 3.25 (d, $^3J_{H,H}$ = 7.0 Hz, 2H, H-23), 1.66 (d, $^3J_{H,H}$ = 6.4 Hz, 2H, H-15), 1.63 – 1.51 (m, 2H, H-22), 1.32 (m, 12H, H-16 to H-21) ppm.

^{13}C NMR (75.53 MHz, $CDCl_3$) δ = 164.2 (C_q, C-13), 140.2 (C_q, C-12), 139.9 (C_q, C-1), 139.1 (C_q, C-9), 132.7 (CH, C-3 or C-4), 132.4 (CH, C-3 or C-4), 131.9 (CH, C-10), 131.9 (C_q, C-aromatic), 131.5 (C_q, C-aromatic), 126.3 (CH, C-aromatic), 124.4 (CH, C-8), 122.7 (CH, C-

aromatic), 51.6 (CH_2, C-23), 40.9 (CH_2, C-14), 29.6 (CH_2), 29.4 (CH_2), 29.3 (CH_2), 29.2 (CH_2), 29.0 (CH_2), 27.2 (CH_2), 26.8 (CH_2) ppm.

^{19}F NMR (470.53 MHz, $CDCl_3$) δ = -78.3 ppm.

HRMS calcd (M/z) for $C_{23}H_{28}ClN_4O^+$ [M-OTf]$^+$: 411.1952; found: 411.1936.

3-((10-Azidodecyl)carbamoyl)dibenzo[*b,d*]chlorol-5-ium trifluoromethanesulfonate (3ia)

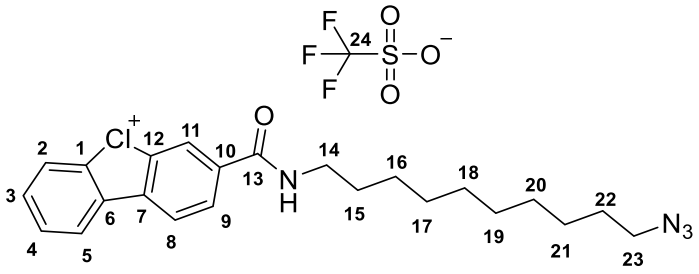

3ia

Starting from 3-carboxydibenzo[*b,d*]chlorol-5-ium trifluoromethanesulfonate (**1i**) (63.2 mg, 0.166 mmol) and 10-azidodecan-1-amine (**2a**) (30.0 mg, 0.151 mmol) synthesis of 3-((10-azidodecyl)carbamoyl)dibenzo[*b,d*]chlorol-5-ium trifluoromethanesulfonate (**3ia**) was executed as described in general procedure B.

Yield 47.0 mg (83.8 µmol, 55%), off-white solid, $C_{24}H_{28}ClF_3N_4O_4S$ [561.02]

HPLC-MS t_R = 6.11 min (method: *2-100-MeCN*); *m/z* (ESI+) = 411 [M-OTf]$^+$

TLC R_f = 0.34 (CH_2Cl_2/MeOH = 7/1; UV and $KMnO_4$)

M.p. 152 – 155 °C

^{1}H NMR (300.36 MHz, DMSO-d^6) δ = 9.14 (s, 1H, H-11), 8.90 (s, 1H, N-H), 8.76 (d, $^3J_{H,H}$ = 8.2 Hz, 1H, H-8), 8.73 – 8.67 (m, 2H, H-2, H-5), 8.41 (d, $^3J_{H,H}$ = 7.4 Hz, 1H, H-9), 8.09 – 7.90 (m, 2H, H-3, H-4), 3.39 – 3.25 (m, 4H, H-14, H-23), 1.68 – 1.42 (m, 4H, H-15, H-22), 1.28 (s, 12H, H-16 to H-21) ppm.

^{13}C NMR (75.53 MHz, DMSO-d^6) δ = 163.5 (C_q, C-13), 140.9 (C_q, C-12), 140.3 (C_q, C-1), 137.7 (C_q, C-10), 134.1 (C_q, C-7), 132.6 (CH, C-3 or C-4), 131.8 (CH, C-3 or C-4), 131.4 (C_q, C-6), 129.6 (CH, C-9), 126.0 (CH, C-5), 125.1 (CH, C-8), 123.1 (CH, C-2), 122.7 (CH, C-

11), 50.6 (CH_2, C-23), 28.9 (CH_2), 28.9 (CH_2), 28.8 (CH_2), 28.7 (CH_2), 28.5 (CH_2), 28.2 (CH_2), 26.4 (CH_2), 26.1 (CH_2) ppm.

^{19}F NMR (470.53 MHz, DMSO-d^6) δ = -77.7 ppm.

HRMS calcd (M/z) for $C_{23}H_{28}ClN_4O^+$ [M-OTf]$^+$: 411.1952; found: 411.1937.

Synthesis of Methyl *t*-butacrylate (*t*-BAM)

Methyl 3,3-dimethyl-2-oxobutanoate (29)

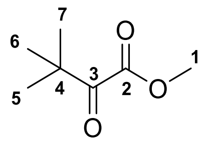

29

In a 100 mL round-bottom flask H_2SO_4 conc. (2.33 mL, 42.8 mmol) was added to a solution of 3,3-dimethyl-2-oxobutanoic acid (3.71 g, 28.5 mmol) in 25 mL MeOH and the mixture was heated for 22 h under reflux until full conversion was detected by GC-MS. Subsequently, the reaction solution was cooled to rt, concentrated under reduced pressure, poured into 25 mL ice water, and extracted with Et_2O (4 x 15 mL). The combined organic layers were washed with sat. $NaHCO_3$ solution (1 x 25 mL) and brine (1 x 25 mL), dried over Na_2SO_4, filtered and the solvent was removed under reduced pressure. The crude product was purified via Kugelrohr distillation (50 mbar, 75 – 105 °C).

Yield 2.51 g (17.4 mmol, 61%), colorless liquid, $C_7H_{12}O_3$ [144.17]

GC-MS t_R = 3.22 min (method: *MT_50_S*); m/z (%) = 144 (2), 85 (25), 57 (100)

^{1}H NMR (300.36 MHz, $CDCl_3$) δ = 3.84 (s, 3H, H-1), 1.25 (s, 9H, H-5 to H-7) ppm.

^{13}C NMR (75.53 MHz, $CDCl_3$) δ = 201.9 (C_q, C-3), 164.2 (C_q, C-2), 52.3 (CH_3, C-1), 42.7 (C_q, C-4), 25.8 (CH_3, C-5 to C-7) ppm.

The recorded spectra are in accordance with the reported in literature.[29]

Methyl 3,3-dimethyl-2-methylenebutanoate (*t*-BAM) (30)

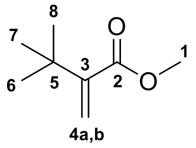

30

In a flame-dried and argon-flushed 250 mL two-neck round-bottom flask diisopropylamine (2.40 mL, 1.73 g, 17.1 mmol) was dissolved in 100 mL THF abs. and cooled to -78 °C (acetone/dry ice). Subsequently, *n*-BuLi (2.5 M in hexanes, 6.45 mL, 16.1 mmol) was added. After stirring at -78 °C for 10 min, methyl triphenylphosphonium bromide (5.75 g, 16.1 mmol) was added in one portion and the yellow solution was stirred for 2 h at -40 °C and 1.5 h at rt. The reaction mixture was cooled to 0 °C and methyl 3,3-dimethyl-2-oxobutanoate (**29**) (2.14 g, 14.8 mmol) was added dropwise. After stirring for 1 h at rt, full conversion was detected by GC-MS and the reaction mixture was quenched by the addition of 15 mL 1 M aq. HCl. The layers were separated and the aqueous layer was extracted with Et_2O (3 x 20 mL). The combined organic layers were washed with H_2O (2 x 25 mL) and brine (1 x 100 mL), dried over Na_2SO_4, filtered, and the solvent was removed under reduced pressure. The residue was taken up in 20 mL *n*-pentane, the resulting precipitates were filtered off using a sintered glass frit and the filtrate was concentrated under reduced pressure. The crude product was purified via Kugelrohr distillation (48 mbar, 65 – 95 °C).

Yield 1.12 g (7.79 mmol, 53%), colorless liquid, $C_8H_{14}O_2$ [142.20]

GC-MS t_R = 3.20 min (method: *MT_50_S*); m/z (%) = 142 (11), 127 (67), 111 (36), 95 (96), 83 (85), 73 (91), 67 (100), 55 (67)

^{1}H NMR (300.36 MHz, $CDCl_3$) δ = 5.92 (s, 1H, H-4a), 5.52 (s, 1H, H-4b), 3.73 (s, 3H, H-1), 1.20 (s, 9H, H-6 to H-8) ppm.

^{13}C NMR (75.53 MHz, $CDCl_3$) δ = 168.4 (C_q, C-2), 150.2 (C_q, C-3), 121.2 (CH_2, C-4), 51.5 (CH_3, C-1), 35.0 (C_q, C-5), 29.5 (CH_3, C-6 to C-8) ppm.

The recorded spectra are in accordance with the reported in literature.[29]

Mechanistic Experiments

Cyclic Voltammetry

Cyclovoltammograms were recorded at rt using an Autolab PGSTAT12 potentiostat in a three-electrode cell with platinum disc, platinum wire, and silver wire as working (WE), counter (CE) and reference electrodes (RE), respectively. A 0.1 M solution of Bu_4NClO_4 in dry acetonitrile served as the supporting electrolyte. Dry nitrogen gas was bubbled through the solutions for 5 min prior to electrochemical experiments. Ferrocene was used as the internal reference to determine peak potentials.

Spin-trapping EPR

EPR spectra were recorded by a Magnettech Miniscope MS 300 EPR spectrometer under nitrogen atmosphere. The spin trap (PBN) with no background EPR signal was dissolved together with the corresponding halonium salts in MeOH/H_2O. A saturated solution of NADH was rapidly added to those mixtures and resulting EPR spectra were recorded within 1 min after mixing.

^{1}H NMR and CIDNP

^{1}H NMR and CIDNP spectra were recorded using a Bruker Avance 200 MHz NMR spectrometer. NADH was added to a solution of halonium salt in CD_3OD/D_2O in the NMR tube. After rapid mixing, CIDNP spectrum of the ongoing reaction was recorded using the following NMR pulse sequence: presaturation of the background (waltz16) – 3 µs delay (to create CIDNP polarizations) – RF pulse (4 µs) – acquisition. This should allow detection of pure CIDNP polarizations with minimal contamination by the background.

HPLC-MS Analysis of *t*-BAM Experiments

Sample solutions from redox reaction of **1f** or **1h**, respectively, with NADH and *t*-BAM (**30**) in D_2O/MeCN-*d3* were directly injected into the HPLC-MS system and analyzed with the standard method (*MeCN_2_100*; see section Materials & Methods). Cationic fragmentation products of *t*-BAM (**30**) adducts could be detected.

Spectrophotometric Activity Assays

Changes in the activity of NQO1 with or without pre-incubation with probe **3aa** were investigated by using a dual-beam spectrophotometer (Specord 200Plus, Analytic Jena, Jena, Germany) at 25 °C. All samples were prepared in 50 mM HEPES, pH 7.0 and the final reaction mixture contained 200 µM NADH, 200 µM menadione (dissolved in EtOH), and 10 nM NQO1. In the experiments with labeled enzyme, NQO1 (40 µM) was pre-incubated with 800 µM **3aa** (dissolved in DMSO) and 800 µM NADH for 1 h at 37 °C. The reaction mixtures were first incubated for 2 min at 25 °C, whereupon the reaction was started by the addition of labeled/unlabeled enzyme. The decrease in absorption caused by the breakdown of NADH was measured for 60 s at 365 nm. In all cases the reference cuvette was filled with 50 mM HEPES, pH 7.0.

UV-Vis Spectrophotometric Experiments

Changes in the flavin spectrum of NQO1 during reduction with NADH and reaction with different probes were recorded using a dual-beam spectrophotometer (Specord 200Plus, Analytic Jena, Jena, Germany). All samples were prepared in 50 mM HEPES, pH 7.0 and spectra were recorded from 300 to 800 nm.

Probes were dissolved in DMSO and then diluted to a final concentration of 100 mM.

The enzyme NQO1 was diluted to a final concentration of 40 µM in in 50 mM HEPES, pH 7.0 and the initial spectrum of the flavin cofactor was recorded from 300 to 800 nm. Afterwards, NADH was added in the desired concentration (2-fold or 4-fold excess, 80 or 160 µM, respectively) and spectra were recorded in regular intervals (every 60 s). Representative spectra of the reduction of the flavin cofactor were selected and plotted.

The sample with the reduced NQO1 was either left under aerobic conditions and the reoxidation of the flavin cofactor after consumption of the entire NADH was followed from 300 to 800 nm, or the protein sample with the reduced flavin cofactor was used for testing the reaction of NQO1 with different probes. Therefore, 400 µM (10 eq.) of the respective probe were added to the measurement cuvette after addition of NADH and after verification of the reduced flavin spectrum. The reaction of the reduced flavin cofactor with the probes was again monitored by measuring the resulting spectra in regular time intervals (every 60 s).

In all cases the reference cuvette was filled with 50 mM HEPES, pH 7.0 containing 0.4% DMSO. All samples were centrifuged at 13000 rpm for 5 min before recording the final spectrum to remove turbidities that would otherwise cause baseline drift.

Biochemical Experiments

Recombinant Enzyme Experiments

Alcohol dehydrogenase from *Saccharomyces cerevisiae* (ADH) was purchased from Sigma-Aldrich (Merck, Darmstadt, Germany). Recombinant *Rhodococcus jostii* eugenol oxidase (EUOX) was a kind gift of Wolfgang Kroutil, University of Graz, Austria, and recombinant human aldehyde dehydrogenase 2 (ALDH2) of Alexander Kollau and Bernd Mayer, University of Graz, Austria. Doris Ribitsch, University of Natural Resources and Life Science, Tulln an der Donau, Austria, kindly provided recombinant *Athrobacter nicotianae* choline oxidase (CHO) and *Clostridium carboxidivorans* formate dehydrogenase (FDH). Recombinant cytochrome P450 enzymes (CYP 10v3/10v4 (*Phenylbacterium zucineum*); CYP 13v3 (linalool 8-monooxygenase; *Mycobacterium intracellulare*)) were kind gifts of Anton Glieder, TU Graz, Austria. 10 µg of pure protein solution was incubated with 1.5 nmol probe (0.15 µL 10 mM solution in DMSO; 30 – 40 µM final concentration), and no probe as control for 1 h at 37 °C. For the concentration-dependent labeling experiments with NQO1, 10 µg of pure protein solution was incubated with 0.03 – 1.5 nmol **3aa** (0.15 µL 0.2 – 10 mM solution in DMSO; 1 – 50 µM final concentration), and no probe as control for 1 h at 37 °C. For the inhibition experiments with NQO1, 10 µg of pure protein solution was pre-incubated with 300 or 600 pmol dicoumarol (0.15 or 0.30 µL 2 mM solution in DMSO; 10 or 20 µM final concentration) for 15 min at 30 °C, followed by incubation with 0.9 nmol **3aa** (0.15 µL 6 mM solution in DMSO; 30 µM final concentration) for 1 h at 30 °C. Reduction and alkylation was performed by adding the same volume of a mixture of 4 M urea, 20 mM tris(2-carboxyethyl)phosphine (TCEP), and 60 mM *N*-ethylmaleimide (NEM) to the enzyme/probe solution and incubation for 10 min at 95 °C. 1 nmol Dibac-atto633 (1 µL 1 mM in DMSO) was added to a solution containing 1 µg reduced and alkylated protein and then the mixture was incubated for 1 h at 37 °C in the dark. Then, samples were prepared for SDS-PAGE by adding 3.5 µL of a 5/2 (v/v) mixture of Invitrogen™ NuPAGE™ LDS Sample Buffer (4X) (Thermo Fisher Scientific) and Invitrogen™ NuPAGE™ Sample Reducing Agent (10X) (Thermo Fisher Scientific) followed by incubation for 5 min at 95 °C. The prepared samples were then loaded

on Invitrogen™ NuPAGE™ Bis-Tris Midi Protein Gels (4 to 12%, 1.0 mm) (Thermo Fisher Scientific) in 3-(*N*-morpholino)propanesulfonic acid (MOPS) buffer and SDS-PAGE was performed using an Invitrogen™ ZOOM® Dual Power Supply with the following settings: 200 V, 400 mA, 200 W. The protein gel was subsequently scanned for active proteins with Cy5 settings on a Biorad ChemiDoc MP imaging system. Further, the whole protein stain was performed with Krypton fluorescent protein stain (Thermo Fisher Scientific) and visualized with Cy3 settings on a Biorad ChemiDoc MP imaging system. Used buffers for probe labeling for the different pure enzymes: *ALDH2*: 50 mM TrisHCl pH 8.5, 4 mM NAD^+; *EUOX*: 20 mM TrisHCl pH 7.5; *CHO*: 10 mM TrisHCl pH 7.5, 1 mM NADH; *FDH*: 20 mM $NaPO_4$ pH 6.5; *Cyp*: 100 mM KH_2PO_4; *NQO1*: Dulbecco's Phosphate Buffered Saline (DPBS), 100 µM NADH were added directly before addition of the probe. In the denaturation gel, the denaturation samples were adjusted to 1% w/w SDS concentration by the addition of 20% w/w aq. SDS-solution and incubated for 10 min at 95 °C prior to probe labeling.

Expression of NQO1 and Corresponding Amino Acid Sequence

Human NQO1 WT was produced in *E. coli* BL21 [pET28a – NQO1] with a hexahistidine tag (underlined) at the *C*-terminus.

Amino Acid Sequence:

MGSSHHHHHHSSGLVPRGSHMVGRRALIVLAHSERTSFNYAMKEAAAAALKKKGW
EVVESDLYAMNFNPIISRKDITGKLKDPANFQYPAESVLAYKEGHLSPDIVAEQKKLE
AADLVIFQFPLQWFGVPAILKGWFERVFIGEFAYTYAAMYDKGPFRSKKAVLSITTG
GSGSMYSLQGIHGDMNVILWPIQSGILHFCGFQVLEPQLTYSIGHTPADARIQILEGWK
KRLENIWDETPLYFAPSSLFDLNFQAGFLMKKEVQDEEKNKKFGLSVGHHLGKSIPT
DNQIKARK

In vitro Labeling Experiments of Mouse Liver Tissues

C57bl6 mice were housed in a clean and temperature-controlled environment with unlimited access to food and water on a regular 12 h/12 h light/dark cycle. Male and female mice aged 8-10 weeks were sacrificed in *ad libitum* fed state and livers were harvested for further analyses. The experiment was performed in accordance with the European Directive 2010/63/EU.

Murine fresh liver was cut into small pieces using a scalpel. A few parts (3-4 mm^3) were incubated either with 150 µL PBS containing 30 µM probe or without probe as negative control (n = 6). After 1 h of incubation at 37 °C, each sample was separately homogenized by sonication. The cell pellet was removed by centrifugation at 7000 *g* and the protein content of the supernatant was determined with the BCA protein assay kit (Thermo Fisher Scientific) according to the manufacturer's instruction. Activity-based gel electrophoresis was performed as stated below.

Activity-based Gels

30 - 50 µg of already reduced and NEM-ylated protein extract were incubated for 1 h with the fluorophore DIBAC-atto633. The samples were then prepared for SDS PAGE by adding a 5/2 (v/v) mixture of Invitrogen™ NuPAGE™ LDS Sample Buffer (4X) (Thermo Fisher Scientific) and Invitrogen™ NuPAGE™ Sample Reducing Agent (10X) (Thermo Fisher Scientific) followed by incubation for 5 min at 95 °C. The prepared samples were then loaded on Invitrogen™ NuPAGE™ Bis-Tris Midi Protein Gels (4 to 12%, 1.0 mm) (Thermo Fisher Scientific) in MOPS buffer and SDS-PAGE was performed using an Invitrogen™ ZOOM® Dual Power Supply with the following settings: 200 V, 400 mA, 200 W. The protein gel was scanned for active proteins with Cy5 settings on a Biorad ChemiDoc MP imaging system. Afterwards, whole protein was stained with Krypton fluorescent protein stain (Thermo Fisher Scientific) and visualized with Cy3 settings on a Biorad Chemidoc MP.

Protein Enrichment Experiments

500 µg protein was subjected to acetone precipitation. Re-solubilization and reduction were performed in 100 µL 100 mM TrisHCl, pH = 8.5, with 1% SDS w/v and 10 mM TCEP for 10 min at 95 °C. Alkylation was performed with NEM at a final concentration of 30 mM NEM at 37 °C for 30 min. After addition of 1200 pmol dibenzocyclooctyne (DBCO)-TEV-Biotin strain promoted click linker (Figure S23, PiChem, Austria), the click reaction was carried out under shaking at 37 °C and 500 rpm overnight. Samples were diluted to < 0.1% SDS concentration and excess linker was removed employing 3 kDa cut-off filters. The retentate was washed twice with 500 µL 8 M urea, 100 mM Tris-HCl, pH = 8.5 and once with 2 M urea, 100 mM TrisHCl, pH = 8.5. Enrichment was performed using 12 µL streptavidin-agarose resin (Thermo Fisher Scientific) on an overhead rotator in plugged spin columns (Thermo Fisher Scientific) for 4 h. The beads were washed once with 2 M urea, 100 mM TrisHCl, pH = 8.5,

and once with 100 mM TrisHCl, pH = 8.5, 2 M urea, 0.5 mM ethylenediaminetetraacetic acid (EDTA), 1 mM DTT, and once again with 2 M urea, 100 mM Tris-HCl, pH = 8.5, before incubation with 200 µL 100 mM Tris-HCl, pH = 8.5, containing 400 ng trypsin (Promega), for 10 h. The supernatant of the on-bead digest was harvested by centrifugation (500 *g*, 5 min).

LC-MS/MS Analysis and Bioinformatics for Protein Enrichment Experiments

One third of the on-bead digest was separated on the Evosep One equipped with an IonOpticks Aurora Series UHPLC C18 column (15 cm x 75 µm ID, 1.7 µm) (IonOpticks, Australia). The LC-method Whisper_40SPD was used with solvent A being 0.1% formic acid in water and solvent B acetonitrile containing 0.1% formic acid while maintaining the column at 40 °C. The timsTOF Pro mass spectrometer (Bruker Daltonics, Germany) was operated in positive mode with enabled trapped ion mobility spectrometry (TIMS) at 100% duty cycle (100 ms ramp time). Source capillary voltage was set to 1500 V and dry gas flow to 3 $L \cdot min^{-1}$ at 180 °C. Scan mode was set to data independent parallel accumulation–serial fragmentation (diaPASEF) using parameters previously optimised with py_diAID.[30] In brief, 24 isolation windows from m/z 300 to 1,200 and $1/K_0$ 0.7 to 1.35 were defined. After MS1 scan, two isolation windows were fragmented per TIMS ramp resulting in an overall DIA cycle time of 1.38 s. The mass spectrometry proteomics data were deposited to the ProteomeXchange[31,32] Consortium *via* the PRIDE[33] partner repository with the dataset identifier PXD043873 and reviewer account details: reviewer_pxd043873@ebi.ac.uk, pw: 785SJoUH.

The LC-MS/MS data were analyzed by DIANN 1.8.1 by searching the public SwissProt murine database containing all common contaminants. Cysteine NEM-ylation was set as fixed modification and methionine oxidation was considered as variable. Digestion enzyme was trypsin with a maximum of one missed-cleavage sites allowed. Acceptance parameters for identification were set to 1% false discovery rate (FDR) for peptide-spectrum matching (PSM), protein as well as site decoy fraction. Mass accuracies and scan windows size for match between runs was determined automatically by the algorithm.

Data processing was performed with Perseus software version 2.0.1.0. Data was filtered for decoy hits and contaminants. After log2 transformation, proteins were filtered for containing at least 4 valid values in probed or control groups. Missing values were imputed with random numbers that are drawn from a Gaussian distribution. The values were optimized to simulate a typical abundance region that the missing values would have if they had been measured.

Pairwise statistical analysis of proteins in probed vs. non-probed samples was performed employing a one-sided Student's t-test. The entire dataset was also analyzed by ANOVA and Tukey's Post-Hoc test to determine differences between probes. For each statistical analysis in Perseus, multi-testing correction was done in with permutation-based FDR with parameters S_0 = 0.1 to 2 and FDR = 0.05.

Cluster analysis was performed in Python 3.7.7 employing the packages matplotlib 3.5.3, pandas 1.3.3, numpy 1.21.6, and seaborn 0.12.2, linkage complete, distance Euclidean.

Probes Site Analysis

500 µg protein was reduced in 50 mM TrisHCl, pH = 8.5, with 1% SDS w/v and 10 mM TCEP for 10 min at 95 °C and subsequently alkylated with NEM at a final concentration of 30 mM at 37 °C for 30 min before acetone precipitation. Samples were re-solubilized in 100 mM TrisHCl, pH = 8.5, with 1% SDS, and Cu-catalyzed click reaction was performed with 21 µM dialkoxydiphenylsilane (DADPS)-Biotin alkyne linker (Figure S39, Vector Laboratories) using 7.8 mM THPTA (tris-hydroxypropyltriazolylmethylamine) (both Vector Laboratories), 1.9 mM copper(II) sulfate and 19 mM sodium ascorbate for 60 min at rt. After 1:12 dilution with 8 M urea, 100 mM Tris-HCl, pH = 8.5, excess linker was removed employing 3 kDa cut-off filters. Retentate was washed once with 400 µL 8 M urea, 100 mM Tris-HCl, pH = 8.5 and twice with 2 M urea, 100 mM TrisHCl, pH = 8.5. Enrichment was performed using 15 µL streptavidin-agarose resin (Thermo Scientific) on an overhead rotator in plugged spin columns (Thermo Scientific) for 4 h. The beads were then washed three times with 2 M urea, 100 mM TrisHCl, pH = 8.5, before overnight incubation with 200 µL 2 M urea, 100 mM TrisHCl, pH = 8.5 containing 800 ng Trypsin/Lys-C Mix (Promega). After collection of the supernatant of the on-bead digest by centrifugation (300 *g*, 5 min), the beads were washed with phosphate buffered saline (1 x 120 µL) and 5% MeCN in Milli-Q® H_2O (3 x 120 µL). The residual bound peptides were eluted using 20 µl 10% formic acid for 30 min at rt, followed by a 5 min bead washing step with 20 µl 4% MeCN in Milli-Q® H_2O while collecting both fractions in the same tube. 5 µl of the resulting eluate was subjected to LC-MS/MS analysis.

LC-MS/MS Analysis and Bioinformatics for Probed Site Analysis

Chromatography was carried out on an Ultimate 3000 RSLCnano System (ThermoFisher Scientific, MA, USA) equipped with an Ionopticks Aurora Series UHPLC C18 column (250 mm x 75 µm, 1.7 µm) (Ionopticks, Australia). Solvent A was 0.1% formic acid in H_2O

and solvent B MeCN + 0.1% formic acid. Total LC-MS/MS run time per sample was 94 min. Flow: Constant flow rate 400 nL/min, T = 40 °C. The following gradient was used: 0 – 13 min, isocratic, 2% B; 13 – 33 min, linear, 2% to 10% B; 33 – 53 min, linear, 10% to 25% B; 53 – 63 min, linear, 25% to 37% B; 63 – 73 min, linear, 37% to 80% B; 73 – 83 min, isocratic, 80% B; 83 – 84 min, linear, 80% to 2% B; 84 – 94 min, isocratic, 2% B.

The timsTOF HT mass spectrometer (Bruker Daltonics, Germany) was operated in positive mode with enabled Trapped Ion Mobility Spectrometry (TIMS) at 100% duty cycle (100 ms ramp and accumulation time). Source capillary voltage was set to 1600 V and dry gas flow to 3 L/min at 180 °C. Scan mode was set to parallel accumulation-serial fragmentation (PASEF) for the scan range of 100-1700 m/z. Precursor selection was based on their intensity (data dependent acquisition) and the precursors were allowed to accumulate for the total of four ramps per PASEF cycle, bringing the total cycle time to 0.53 s. The mass spectrometry proteomics data were deposited to the ProteomeXchange[31,32] Consortium *via* the PRIDE[33] partner repository with the dataset identifier PXD043873 and reviewer account details: reviewer_pxd043873@ebi.ac.uk, pw: 785SJoUH.

The LC-MS/MS data were analyzed using FragPipe 22.0 software[34] with incorporated MSFragger version 4.1, IonQuant version 1.10.27 and Philosopher version 5.1.1 searching FASTA entries from *Mus musculus* and common contaminants (17335 Swiss-Prot entries, downloaded on 10.06.2024 from UniProt). Starting with the FragPipe LFQ-MBR workflow the following search criteria were changed and applied, respectively: enzyme ‘stricttrypsin’, max. missed cleavage sites: 2; *N*-ethylmaleimide (NEM) on Cys, oxidation on Met, and the used probe plus cleaved DADPS biotin linker allowed on any amino acid were set as variable modifications. More precisely, the used modification mass of the cleaved linker-probe conjugate was 384.2525 Da for **3aa**, 538.1943 Da for **3ga**, and 494.2448 Da for **3ha**. The precursor and fragment mass tolerance were set to 20 ppm. Identification acceptance criteria included a max. 1% false discovery rate (FDR) on PSMs, peptides and proteins using razor peptides for protein FDR scoring. Additionally, MSBooster was omitted and three ions with a minimum frequency of 0.5 were used for protein quantification.

The resulting peptide tables were used for investigating the identified peptides containing the target probe modification. The hits of the **3ga** and **3ha** probes were further verified through comparison of their distinct MS1 spectra to simulated isotopic patterns unique to chlorine and bromine containing probes using Bruker Compass DataAnalysis software (version 6.1).

References

1 a) M. J. Bird, T. Iyoda, N. Bonura, J. Bakalis, A. J. Ledbetter and J. R. Miller, *J. Electroanal. Chem.*, 2017, **804**, 107; b) G. C. Greenacre and R. N. Young, *J. Chem. Soc., Perkin Trans. 2*, 1975, 1661; c) T. E. H. Esch and J. Smid, *J. Am. Chem. Soc.*, 1966, **88**, 307;

2 D. G. Tew, *Biochemistry*, 1993, **32**, 10209.

3 S. Chakraborty and V. Massey, *J. Biol. Chem.*, 2002, **277**, 41507.

4 E. Weerapana, C. Wang, G. M. Simon, F. Richter, S. Khare, M. B. D. Dillon, D. A. Bachovchin, K. Mowen, D. Baker and B. F. Cravatt, *Nature*, 2010, **468**, 790.

5 S. M. Hacker, K. M. Backus, M. R. Lazear, S. Forli, B. E. Correia and B. F. Cravatt, *Nat. Chem.*, 2017, **9**, 1181.

6 M. Eidi and M. Garshasbi, *BMC Neurol.*, 2019, **19**, 153.

7 D. E. Edmondson, C. Binda and A. Mattevi, *Arch. Biochem. Biophys.*, 2007, **464**, 269.

8 P. J. Holm, P. Bhakat, C. Jegerschöld, N. Gyobu, K. Mitsuoka, Y. Fujiyoshi, R. Morgenstern and H. Hebert, *J. Mol. Biol.*, 2006, **360**, 934.

9 H. E. Gottlieb, V. Kotlyar and A. Nudelman, *J. Org. Chem.*, 1997, **62**, 7512.

10 a) R. H. Blessing, *Acta Crystallogr. A*, 1995, **51 (Pt 1)**, 33; b) G. M. Sheldrick, SADABS Version 2.0 Siemens Area Detector Correction, Universität Göttingen, 2003;

11 a) G. M. Sheldrick, SHELXTL Version 6.1 Bruker AXS, Inc., 2002; b) G. M. Sheldrick, GM SHELXS97 and SHELXL97, Universität Göttingen, 2002;

12 O. V. Dolomanov, L. J. Bourhis, R. J. Gildea, J. A. K. Howard and H. Puschmann, *J. Appl. Crystallogr.*, 2009, **42**, 339.

13 a) A. L. Spek, *J. Appl. Cryst.*, 2003, **36**, 7; b) A. L. Spek, *Acta Cryst. D*, 2009, **65**, 148;

14 S. Roe, M. Gunaratnam, C. Spiteri, P. Sharma, R. D. Alharthy, S. Neidle and J. E. Moses, *Org. Biomol. Chem.*, 2015, **13**, 8500.

15 A. K. Jain, A. Paul, B. Maji, K. Muniyappa and S. Bhattacharya, *J. Med. Chem.*, 2012, **55**, 2981.

16 B. Longo, C. Zanato, M. Piras, S. Dall'Angelo, A. D. Windhorst, D. J. Vugts, M. Baldassarre and M. Zanda, *Bioconjug. Chem.*, 2020, **31**, 2201.

17 L. N. Goswami, Z. H. Houston, S. J. Sarma, S. S. Jalisatgi and M. F. Hawthorne, *Org. Biomol. Chem.*, 2013, **11**, 1116.

18 L. Kraszkiewicz, M. Sosnowski and L. Skulski, *Synthesis*, 2006, 1195.

19 S. Djurdjevic, F. Yang and J. R. Green, *J. Org. Chem.*, 2010, **75**, 8241.

20 E. M. F. Billaud, A. Maisonial-Besset, L. Rbah-Vidal, A. Vidal, S. Besse, J.-B. Béquignat, C. Decombat, F. Degoul, L. Audin, J.-B. Deloye, F. Dollé, B. Kuhnast, J.-C. Madelmont, S. Tarrit, M.-J. Galmier, M. Borel, P. Auzeloux, E. Miot-Noirault and J.-M. Chezal, *Eur. J. Med. Chem.*, 2015, **92**, 818.

21 C. B. Vu, E. G. Corpuz, T. J. Merry, S. G. Pradeepan, C. Bartlett, R. S. Bohacek, M. C. Botfield, C. J. Eyermann, B. A. Lynch, I. A. MacNeil, M. K. Ram, M. R. van Schravendijk, S. Violette and T. K. Sawyer, *J. Med. Chem.*, 1999, **42**, 4088.
22 U. Fluch, B. D. McCarthy and S. Ott, *Dalton Trans.*, 2018, **48**, 45.
23 M. Cigl, R. Jurok, F. Hampl, J. Svoboda, N. Podoliak and V. Novotná, *Liq. Cryst.*, 2020, **47**, 768.
24 R. Papagna, E. Engelage, H. Wieske, M. Erdelyi and S. Huber, *Self-complementary Dimers Based on Zwitterionic Halogen Bond Donors*, 2022.
25 K. Neuthe, C. S. Popeney, K. Bialecka, A. Hinsch, A. Sokolowski, W. Veurmann and R. Haag, *Polyhedron*, 2014, **81**, 583.
26 T. Kunz and P. Knochel, *Angew. Chem. Int. Ed.*, 2012, **51**, 1958.
27 M. Bielawski, M. Zhu and B. Olofsson, *Adv. Synth. Catal.*, 2007, **349**, 2610.
28 Y. Yoshida, S. Ishikawa, T. Mino and M. Sakamoto, *Chem. Commun.*, 2021, **57**, 2519.
29 B. Seidl and R. Liska, *Macromol. Chem. Phys.*, 2007, **208**, 44.
30 P. Skowronek, M. Thielert, E. Voytik, M. C. Tanzer, F. M. Hansen, S. Willems, O. Karayel, A.-D. Brunner, F. Meier and M. Mann, *Mol. Cell. Proteomics*, 2022, **21**, 100279.
31 E. W. Deutsch, N. Bandeira, Y. Perez-Riverol, V. Sharma, J. J. Carver, L. Mendoza, D. J. Kundu, S. Wang, C. Bandla, S. Kamatchinathan, S. Hewapathirana, B. S. Pullman, J. Wertz, Z. Sun, S. Kawano, S. Okuda, Y. Watanabe, B. MacLean, M. J. MacCoss, Y. Zhu, Y. Ishihama and J. A. Vizcaíno, *Nucleic Acids Res.*, 2023, **51**, D1539-D1548.
32 J. A. Vizcaíno, E. W. Deutsch, R. Wang, A. Csordas, F. Reisinger, D. Ríos, J. A. Dianes, Z. Sun, T. Farrah, N. Bandeira, P.-A. Binz, I. Xenarios, M. Eisenacher, G. Mayer, L. Gatto, A. Campos, R. J. Chalkley, H.-J. Kraus, J. P. Albar, S. Martinez-Bartolomé, R. Apweiler, G. S. Omenn, L. Martens, A. R. Jones and H. Hermjakob, *Nat. Biotechnol.*, 2014, **32**, 223.
33 Y. Perez-Riverol, J. Bai, C. Bandla, D. García-Seisdedos, S. Hewapathirana, S. Kamatchinathan, D. J. Kundu, A. Prakash, A. Frericks-Zipper, M. Eisenacher, M. Walzer, S. Wang, A. Brazma and J. A. Vizcaíno, *Nucleic Acids Res.*, 2022, **50**, D543-D552.
34 a) A. T. Kong, F. V. Leprevost, D. M. Avtonomov, D. Mellacheruvu and A. I. Nesvizhskii, *Nat. Methods*, 2017, **14**, 513; b) F. Yu, S. E. Haynes and A. I. Nesvizhskii, *Mol. Cell. Proteomics*, 2021, **20**, 100077; c) F. Yu, S. E. Haynes, G. C. Teo, D. M. Avtonomov, D. A. Polasky and A. I. Nesvizhskii, *Mol. Cell. Proteomics*, 2020, **19**, 1575; d) F. Yu, G. C. Teo, A. T. Kong, S. E. Haynes, D. M. Avtonomov, D. J. Geiszler and A. I. Nesvizhskii, *Nat. Commun.*, 2020, **11**, 4065;

Crystallographic Data

Table S5. Crystallographic data of diarylhalonium salts **1c**, **1f**, and **1h**.

**Compound**	**1c**	**1f**	**1h**
CCDC number	2145617	2149555	2145616
Empirical formula	$C_{14}H_8F_3IO_5S$	$C_{14}H_8F_3BrO_5S$	$C_{14}H_8F_3ClO_5S$
Formula weight [g·mol^{-1}]	472.16	425.17	380.71
Temperature [K]	100.03	100(2)	99.99
Crystal system	triclinic	monoclinic	triclinic
Space group	$P\bar{1}$	$P2_1/n$	$P\bar{1}$
a [Å]	7.0091(7)	6.4902(3)	6.6746(4)
b [Å]	10.6673(11)	18.8943(9)	8.0822(6)
c [Å]	11.7572(12)	12.3367(6)	13.7840(10)
α [°]	63.230(5)	90	95.756(2)
β [°]	75.790(5)	95.462(2)	99.723(2)
γ [°]	85.689(5)	90	97.231(2)
Volume [Å^3]	760.20(14)	1505.95(12)	721.43(9)
Z	2	4	2
ρ_{calc} [g·cm^{-3}]	2.063	1.875	1.753
μ [mm^{-1}]	2.301	2.926	0.469
F(000)	456	840	384
Radiation	MoKα (λ = 0.71073)	MoKα (λ = 0.71073)	MoKα (λ = 0.71073)
2Θ range for data collection [°]	1.997 to 30.161	1.978 to 30.158	1.511 to 30.189
Index ranges	$-9 \leq h \leq 9$, $-15 \leq k \leq 15$, $-16 \leq l \leq 16$	$-9 \leq h \leq 8$, $-26 \leq k \leq 26$, $-17 \leq l \leq 17$	$-9 \leq h \leq 9$, $-11 \leq k \leq 11$, $-19 \leq l \leq 19$
Reflections collected	40453	61285	39301
Independent reflections	4407 [R_{int} = 0.1267]	4391 [R_{int} = 0.0558]	4150 [R_{int} = 0.0496]
Data/restraints/parameters	4407/0/221	4391/0/219	4150/0/219
Goodness-of-fit on F^2	1.053	1.011	1.091
Final R indexes [I>=2σ (I)]	R_1 = 0.0430, wR_2 = 0.0535	R_1 = 0.0237, wR_2 = 0.0606	R_1 = 0.0349, wR_2 = 0.0912
Final R indexes [all data]	R_1 = 0.0783, wR_2 = 0.0609	R_1 = 0.0265, wR_2 = 0.0620	R_1 = 0.0369, wR_2 = 0.0925
Largest diff. peak/hole [e·Å^{-3}]	3.136/-2.844	0.514/-0.468	0.593/-0.537

NMR Appendix

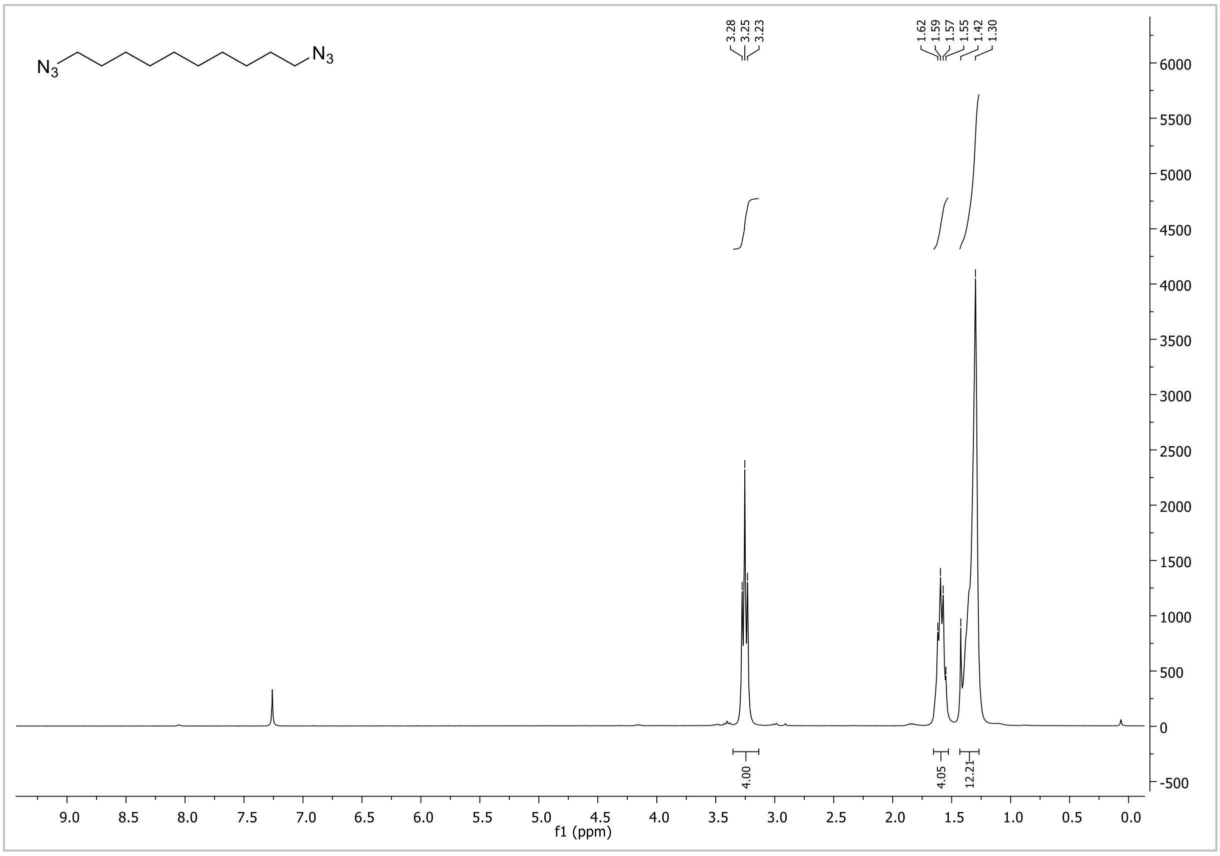

Figure S75. ^{1}H-NMR (300.36 MHz, $CDCl_3$) of compound **15**.

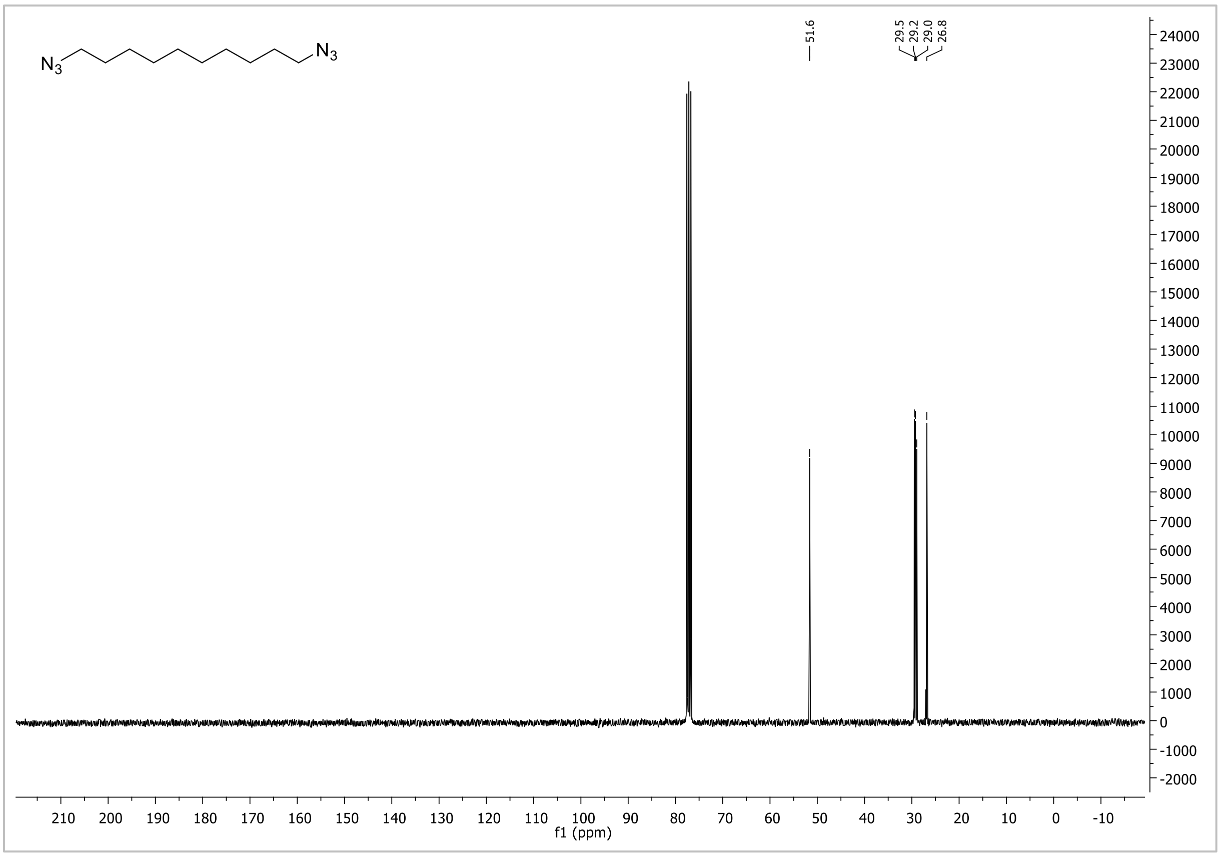

Figure S76. ^{13}C-NMR (75.53 MHz, $CDCl_3$) of compound **15**.

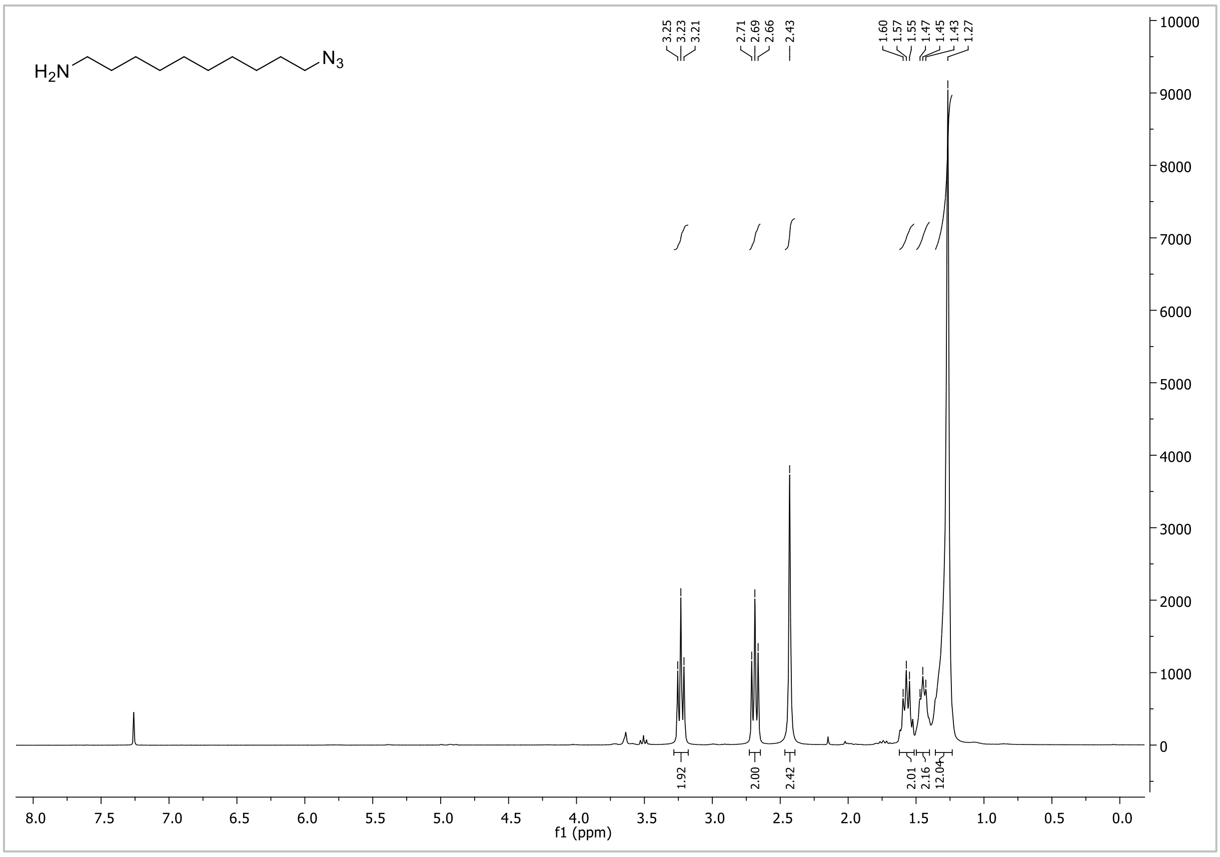

Figure S77. ^{1}H-NMR (300.36 MHz, $CDCl_3$) of compound **2a**.

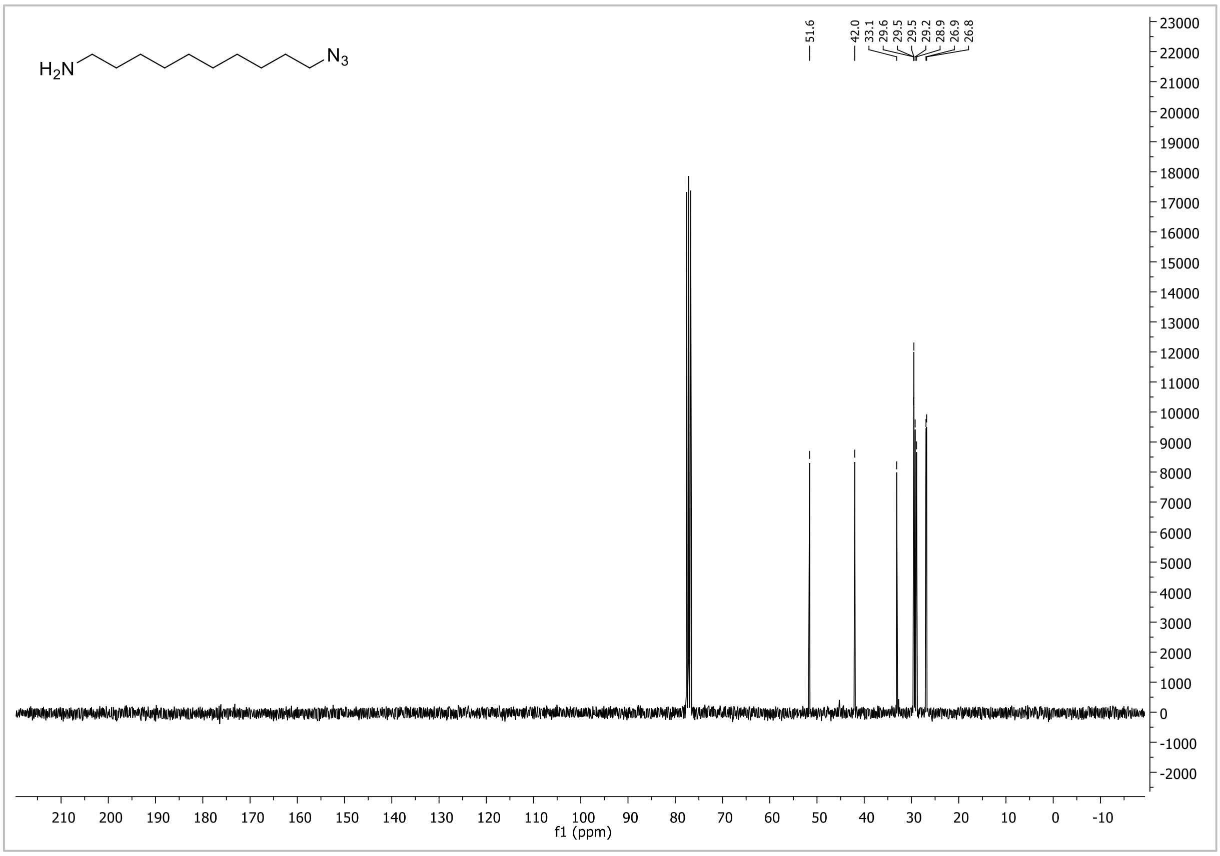

Figure S78. ^{13}C-NMR (75.53 MHz, $CDCl_3$) of compound **2a**.

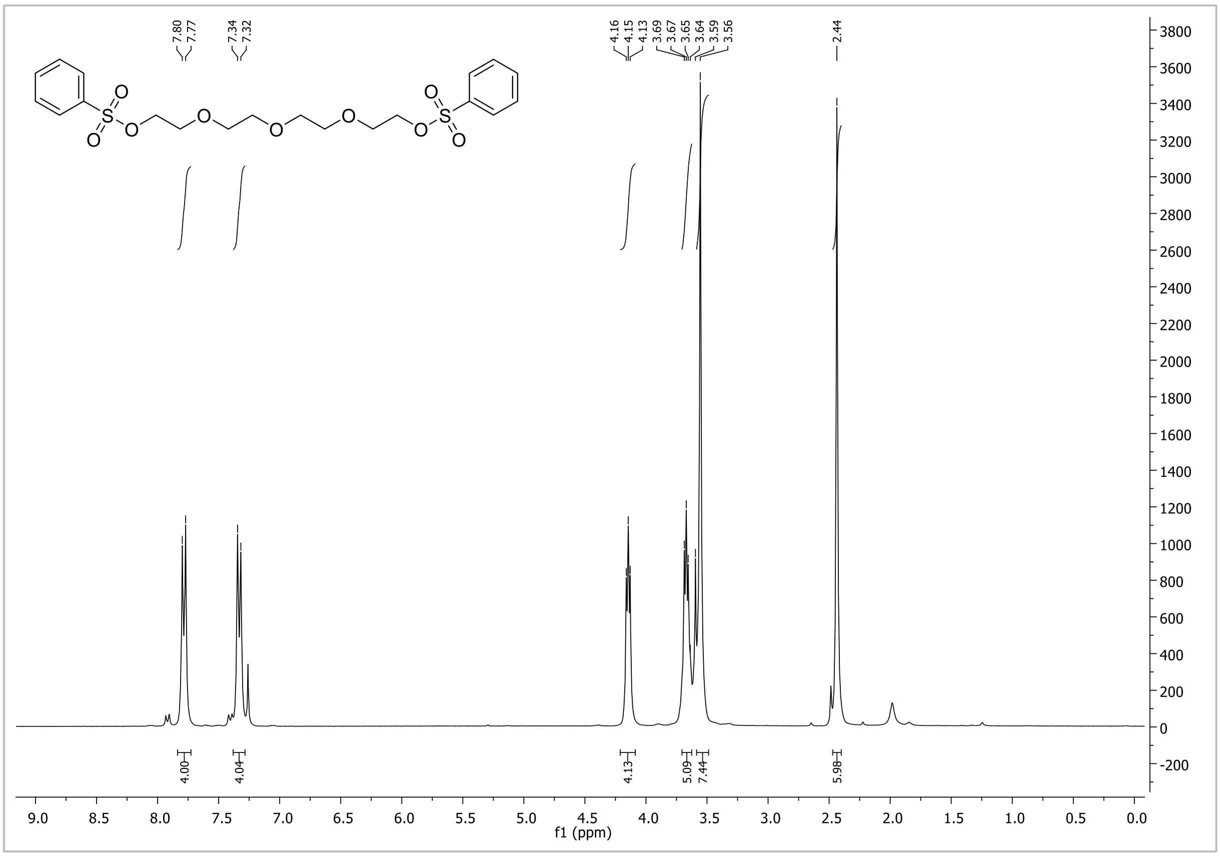

Figure S79. ^{1}H-NMR (300.36 MHz, $CDCl_3$) of compound **16**.

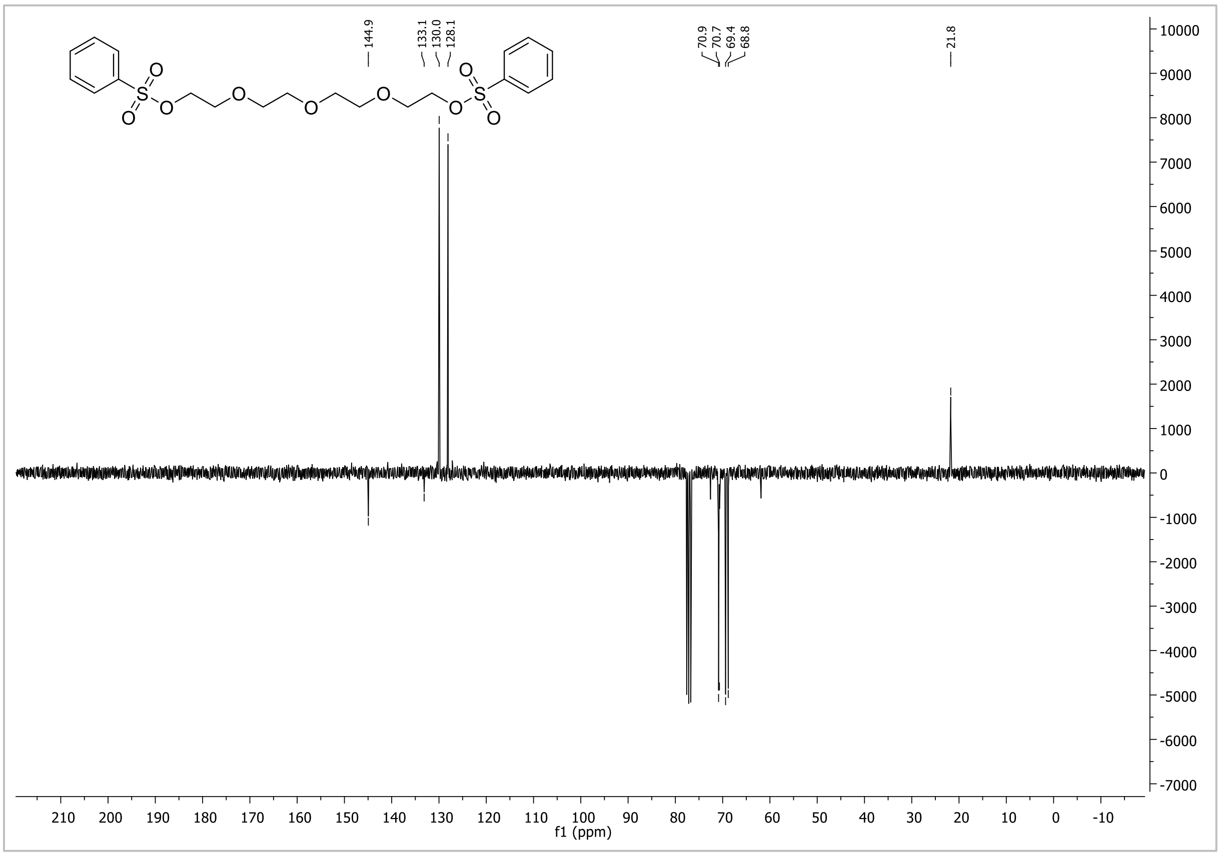

Figure S80. ^{13}C-NMR (75.53 MHz, $CDCl_3$) of compound **16**.

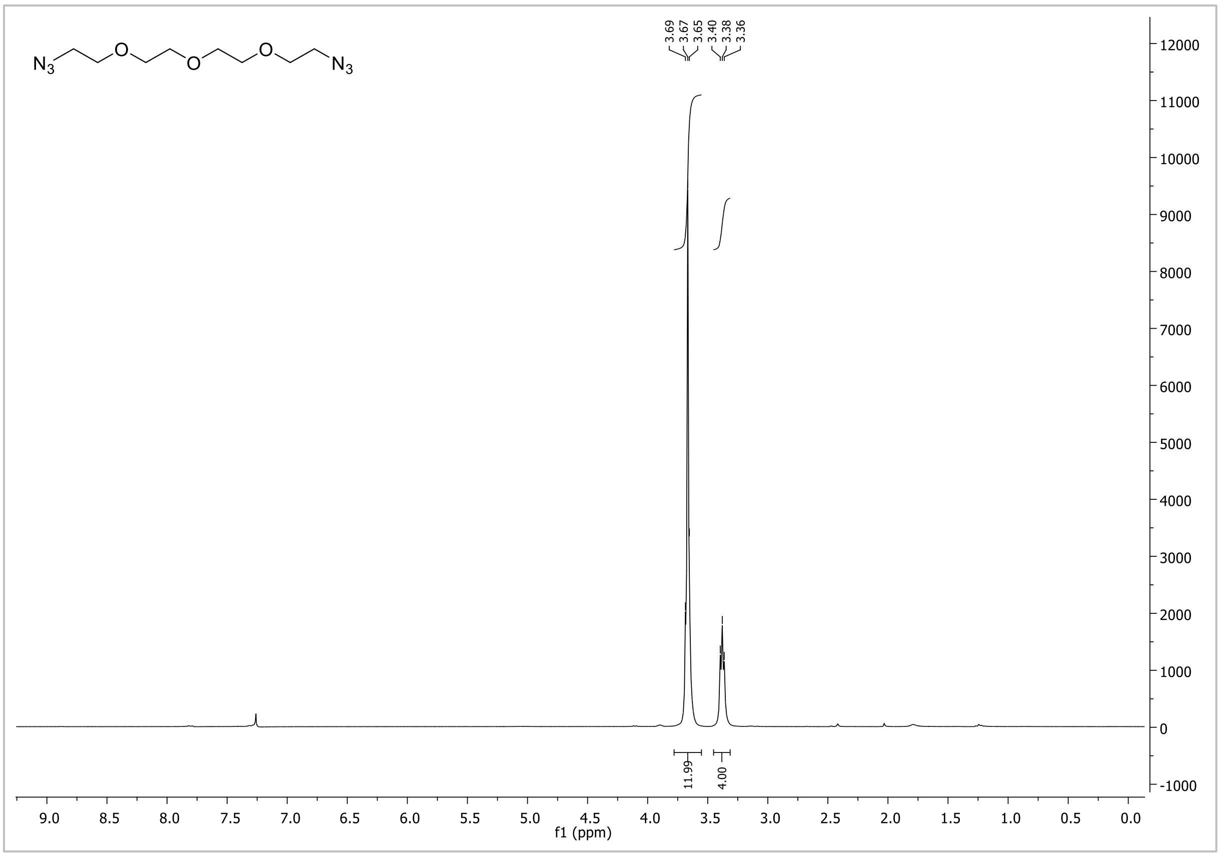

Figure S81. ^{1}H-NMR (300.36 MHz, $CDCl_3$) of compound **17**.

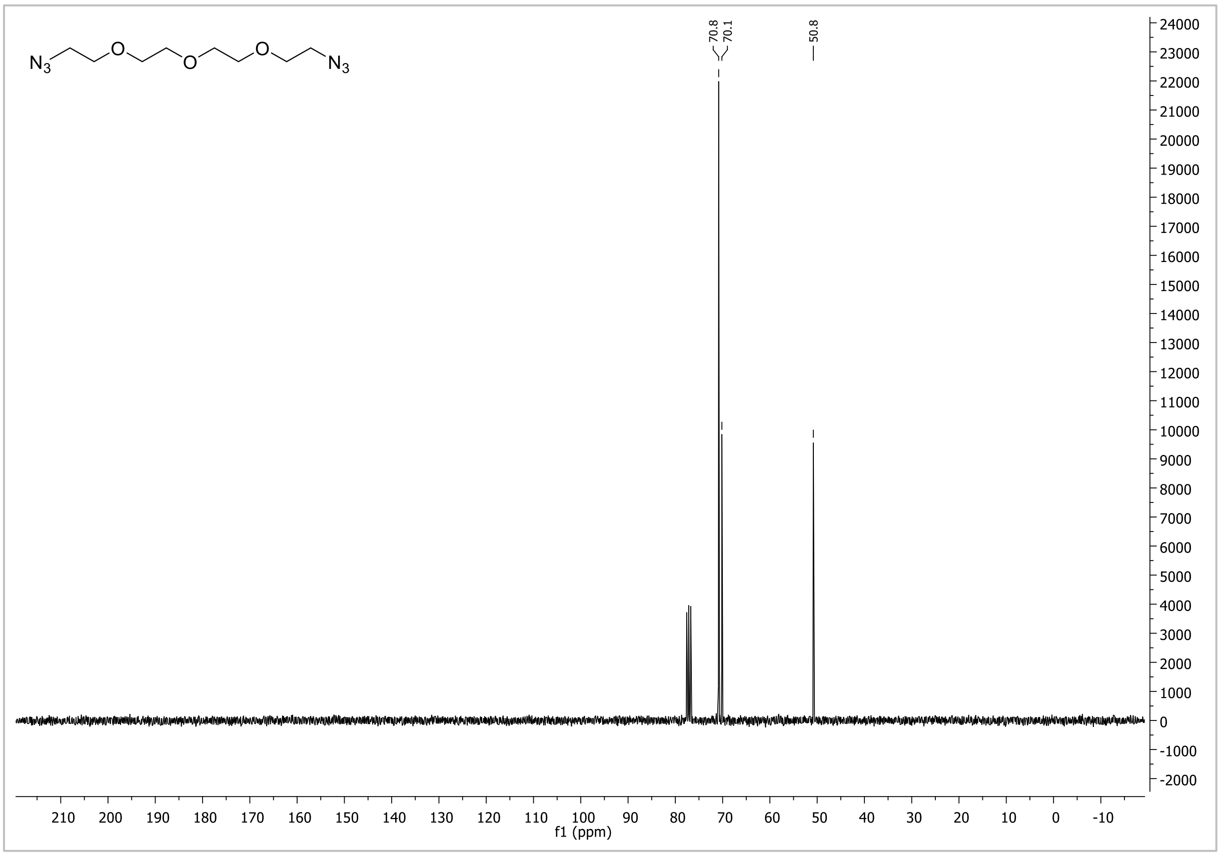

Figure S82. ^{13}C-NMR (75.53 MHz, $CDCl_3$) of compound **17**.

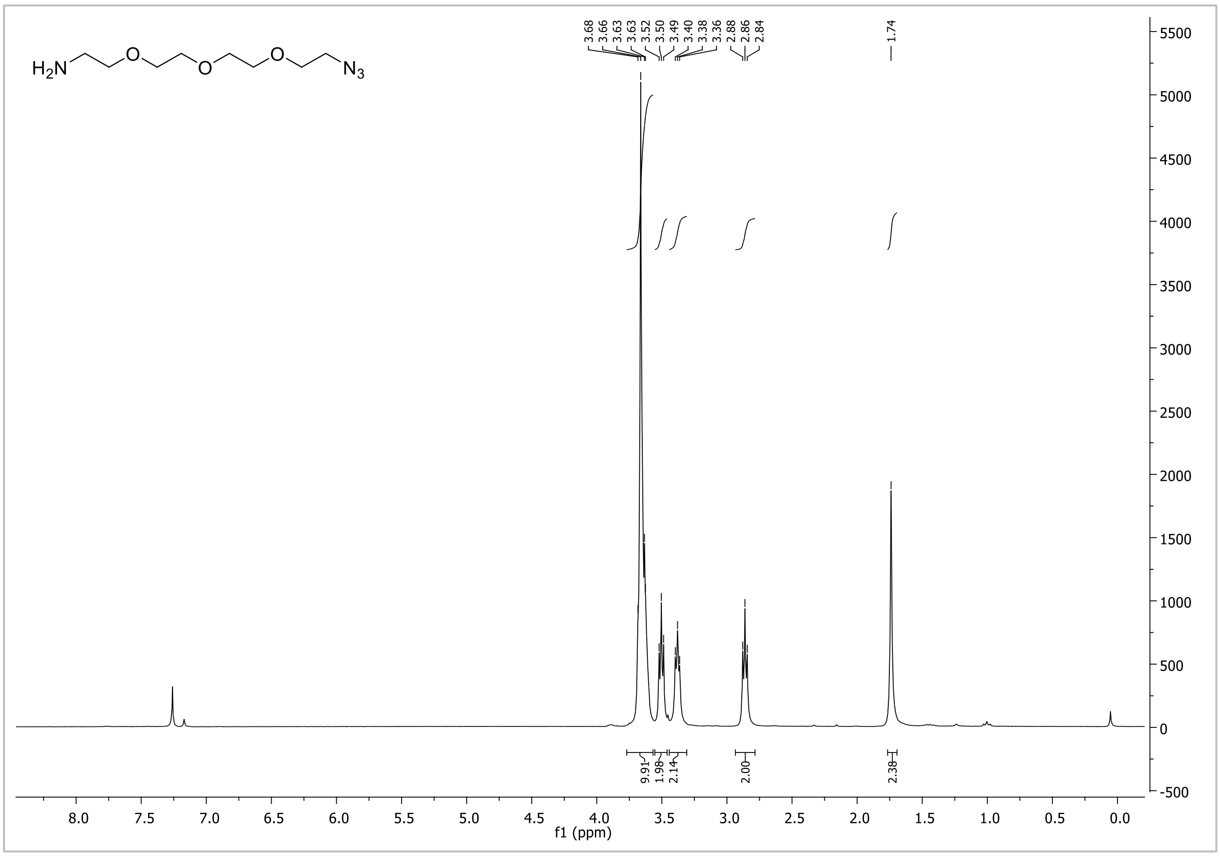

Figure S83. ^{1}H-NMR (300.36 MHz, $CDCl_3$) of compound **2b**.

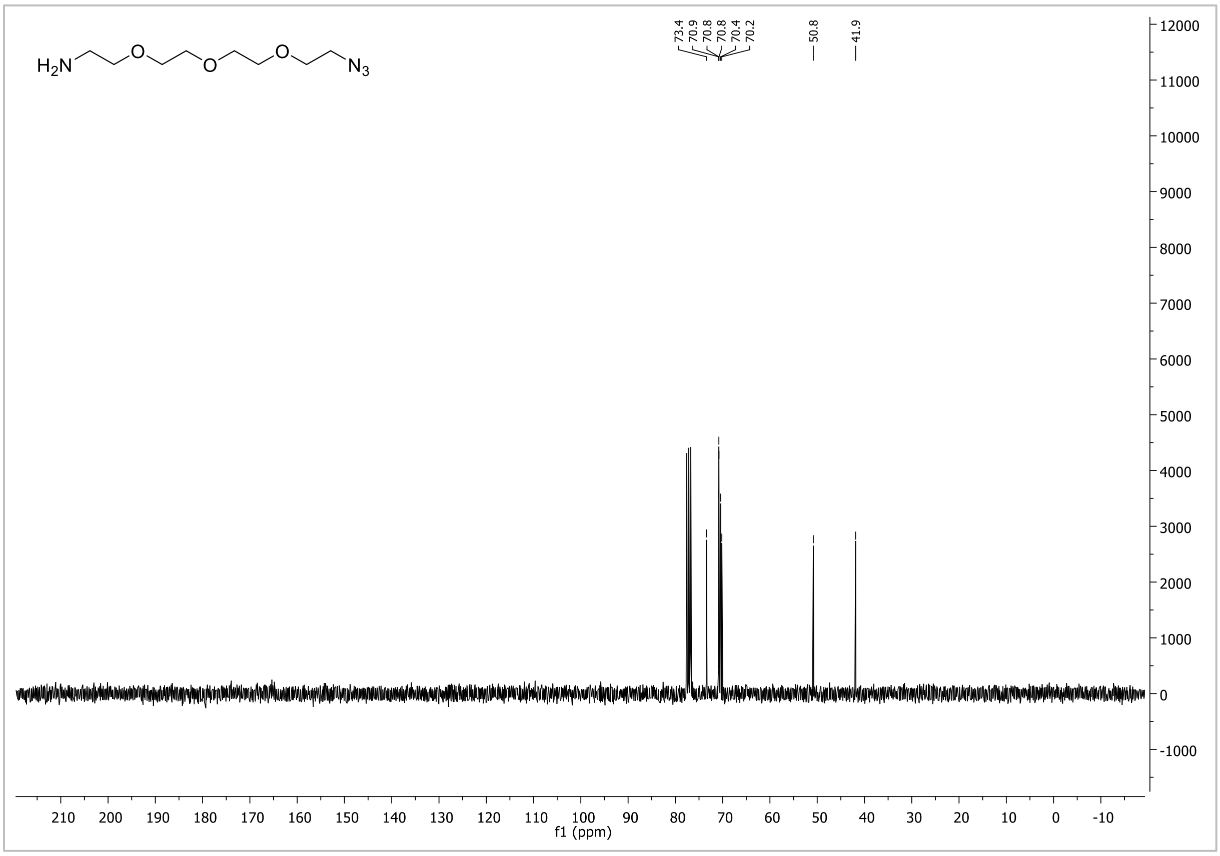

Figure S84. ^{13}C-NMR (75.53 MHz, $CDCl_3$) of compound **2b**.

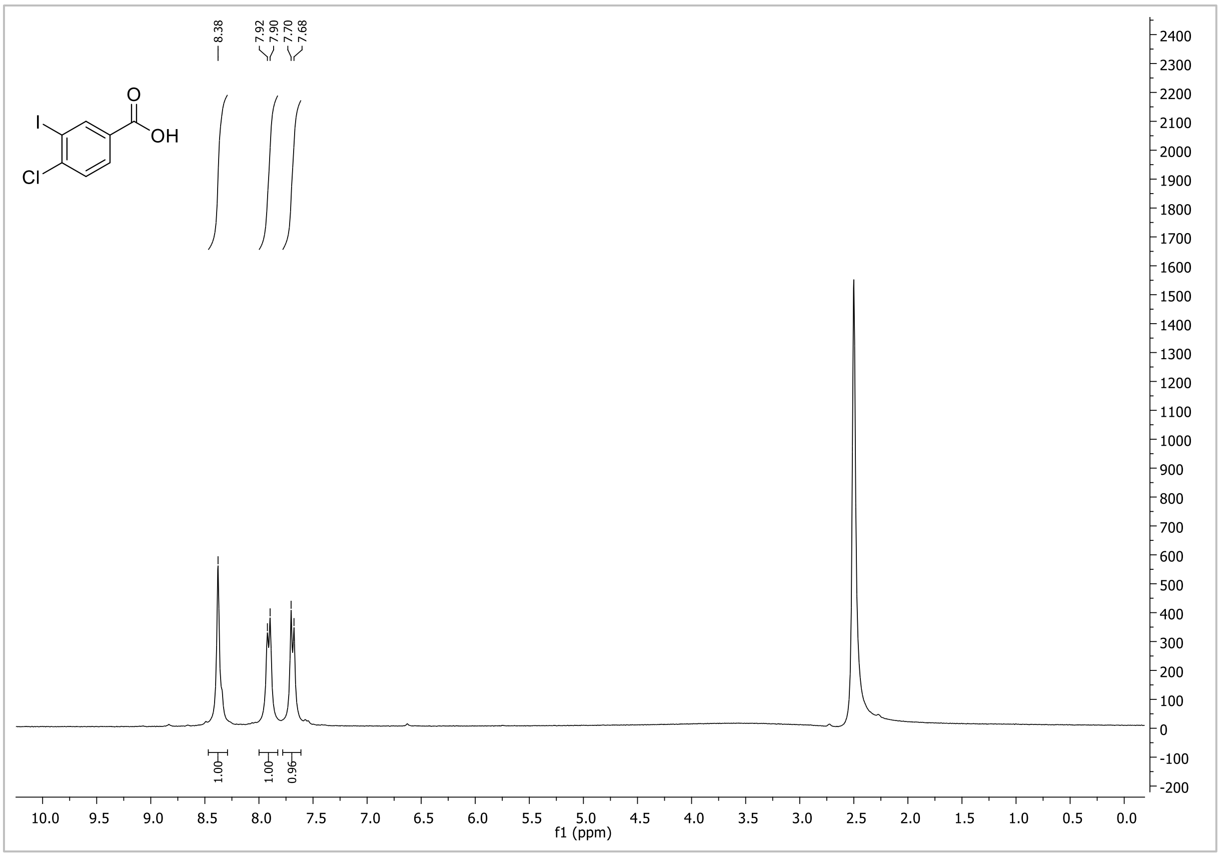

Figure S85. ^{1}H-NMR (300.36 MHz, DMSO-d_6) of compound **19**.

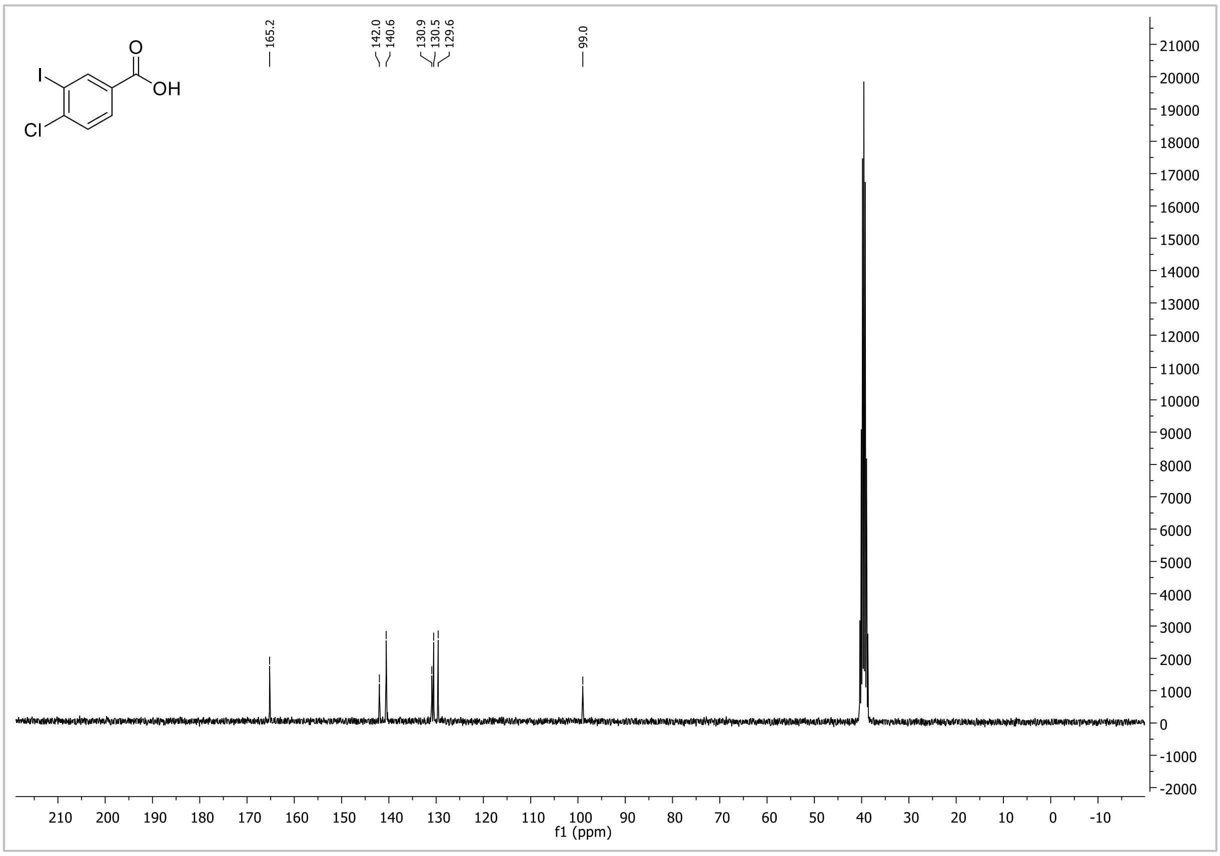

Figure S86. ^{13}C-NMR (75.53 MHz, DMSO-d_6) of compound **19**.

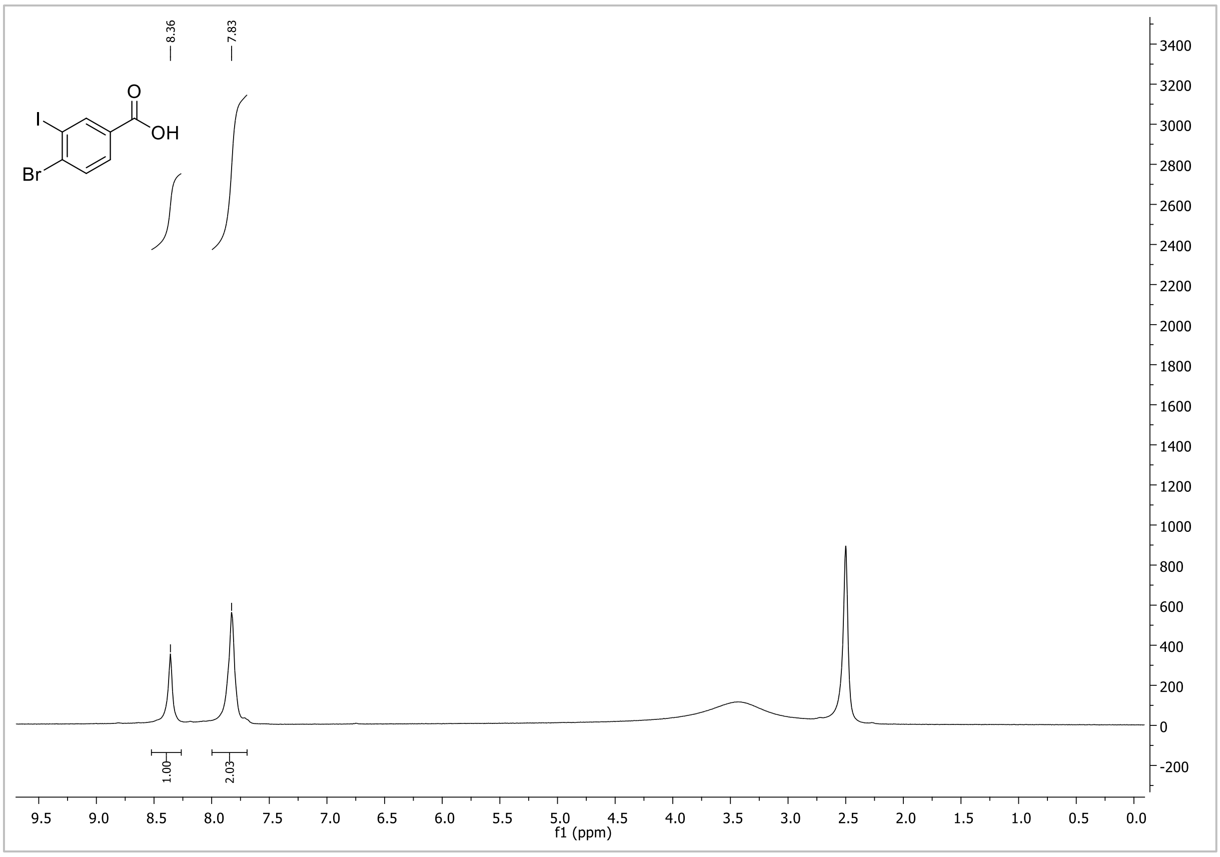

Figure S87. ^{1}H-NMR (300.36 MHz, DMSO-d_6) of compound **18**.

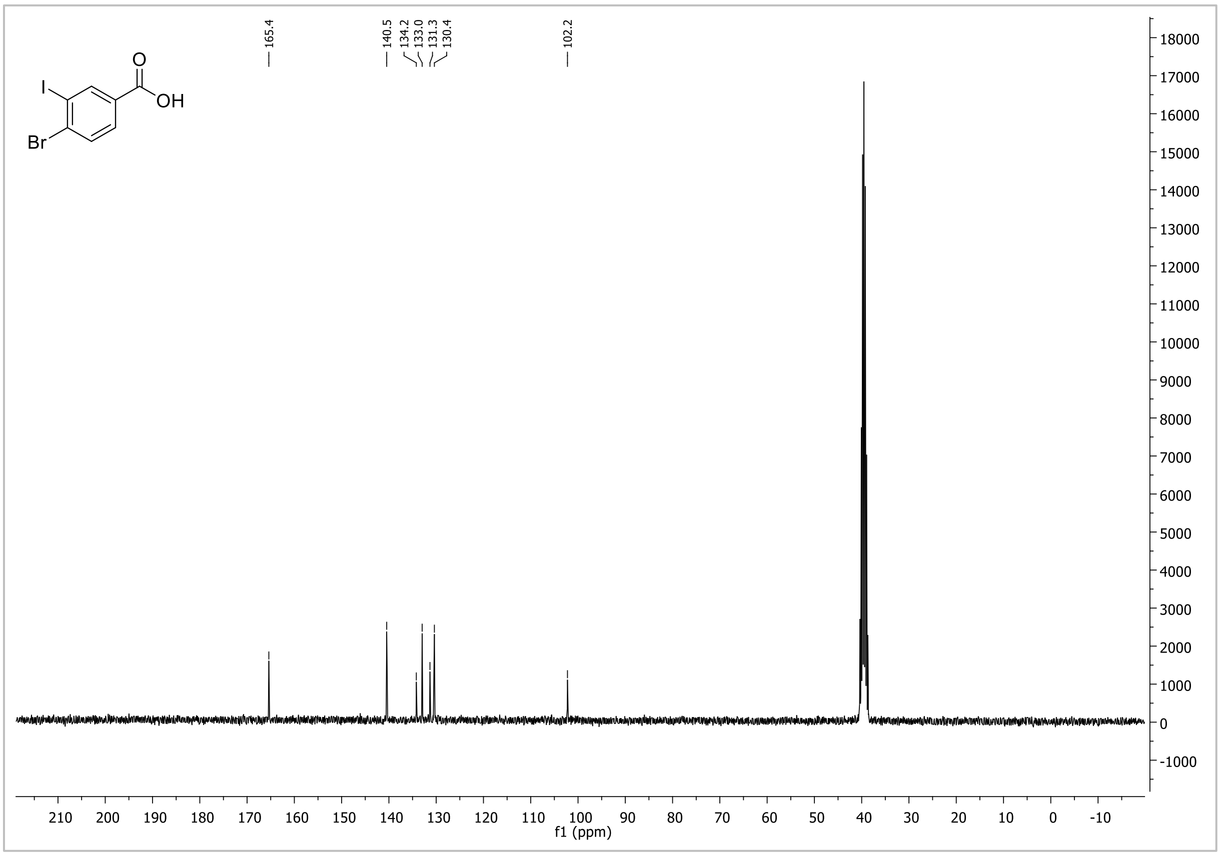

Figure S88. ^{13}C-NMR (75.53 MHz, DMSO-d_6) of compound **18**.

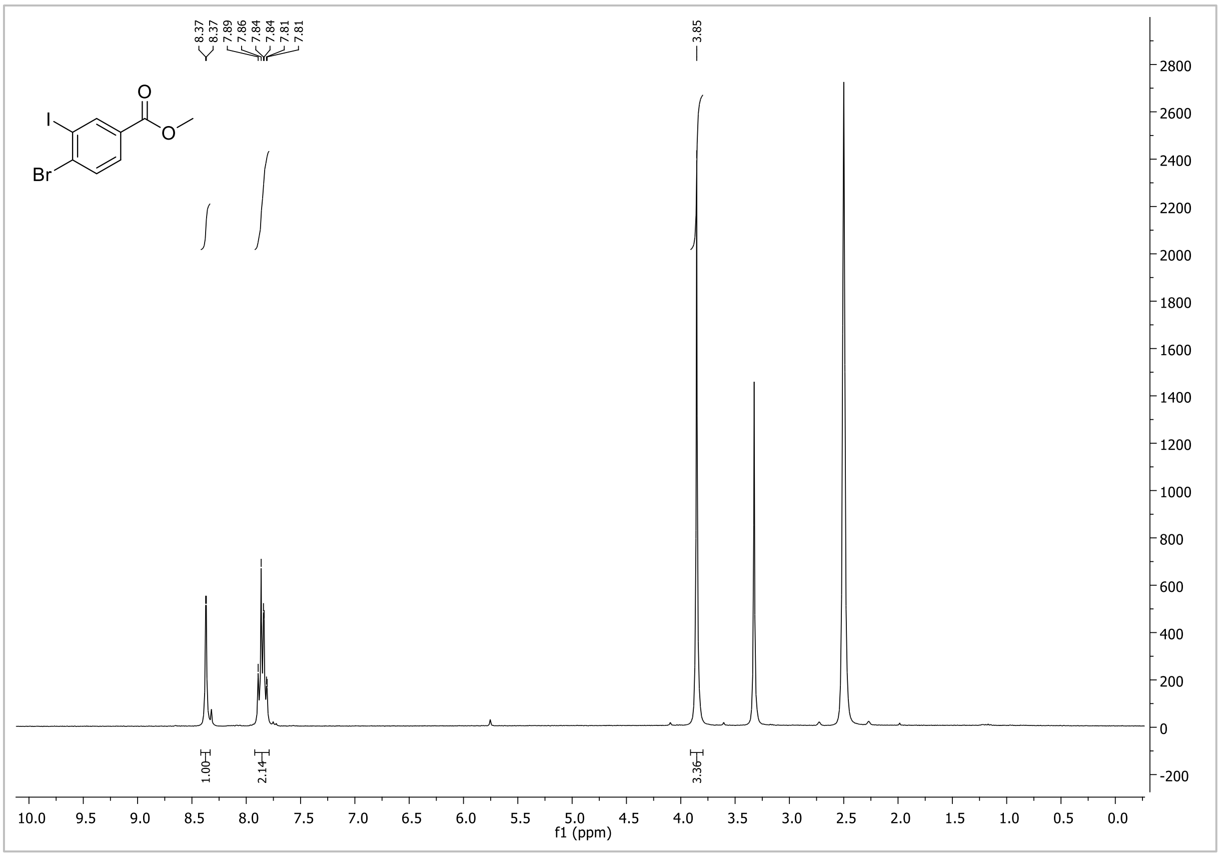

Figure S89. ^{1}H-NMR (300.36 MHz, DMSO-d_6) of compound **20**.

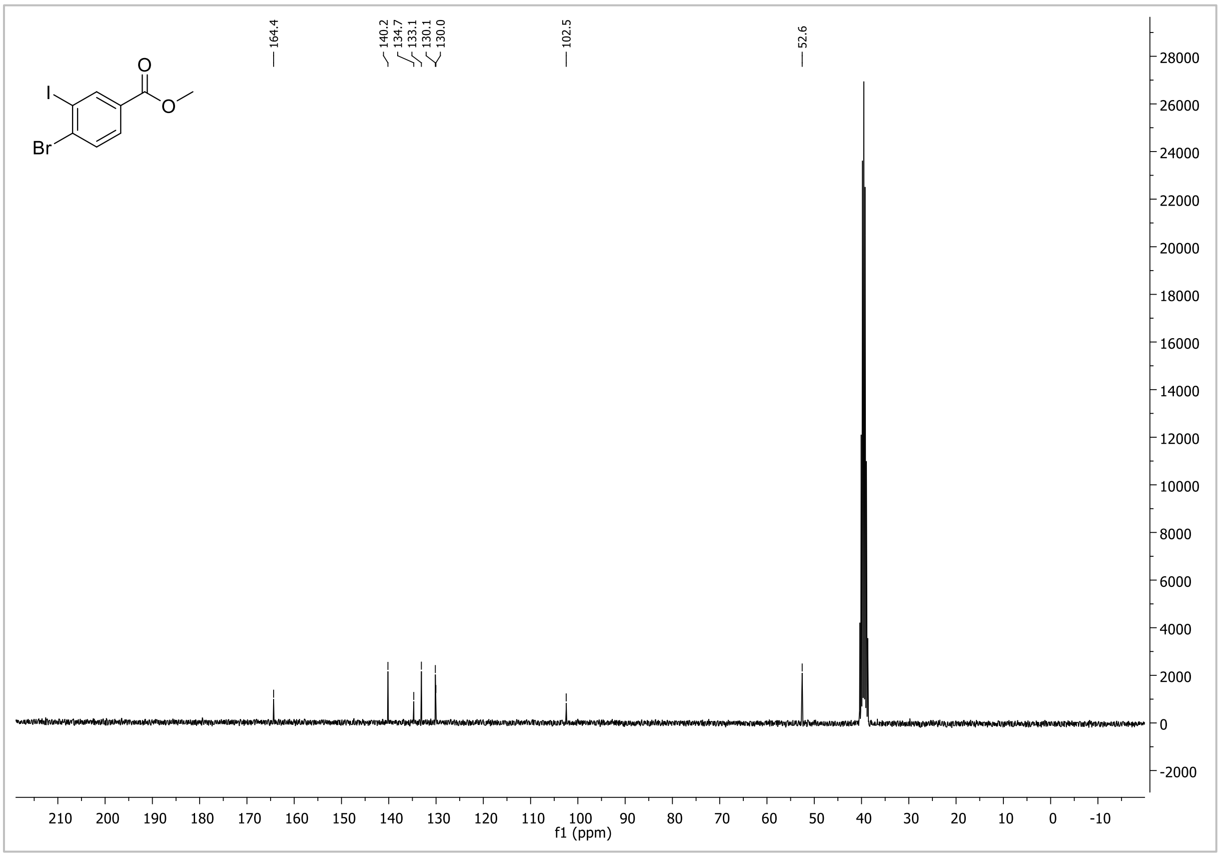

Figure S90. ^{13}C-NMR (75.53 MHz, DMSO-d_6) of compound **20**.

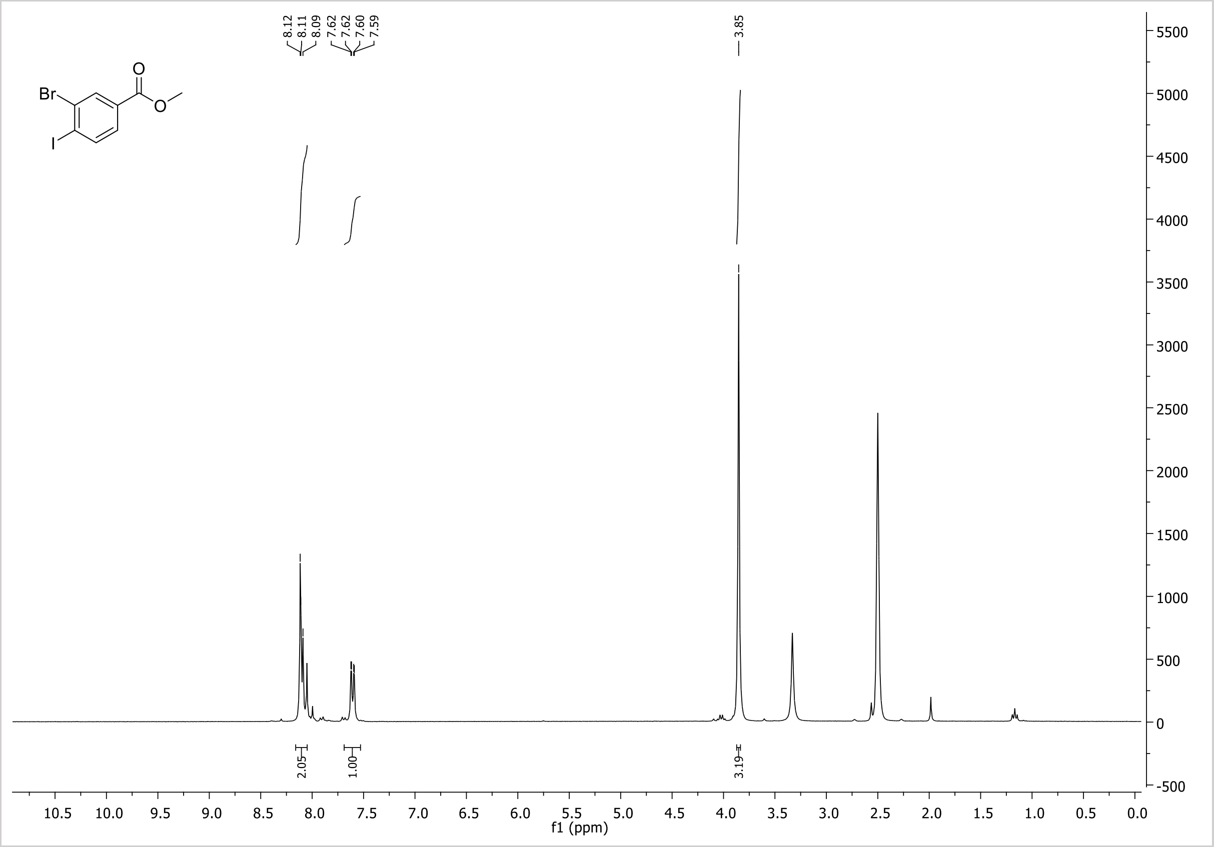

Figure S91. ^{1}H-NMR (300.36 MHz, DMSO-d_6) of compound **22**.

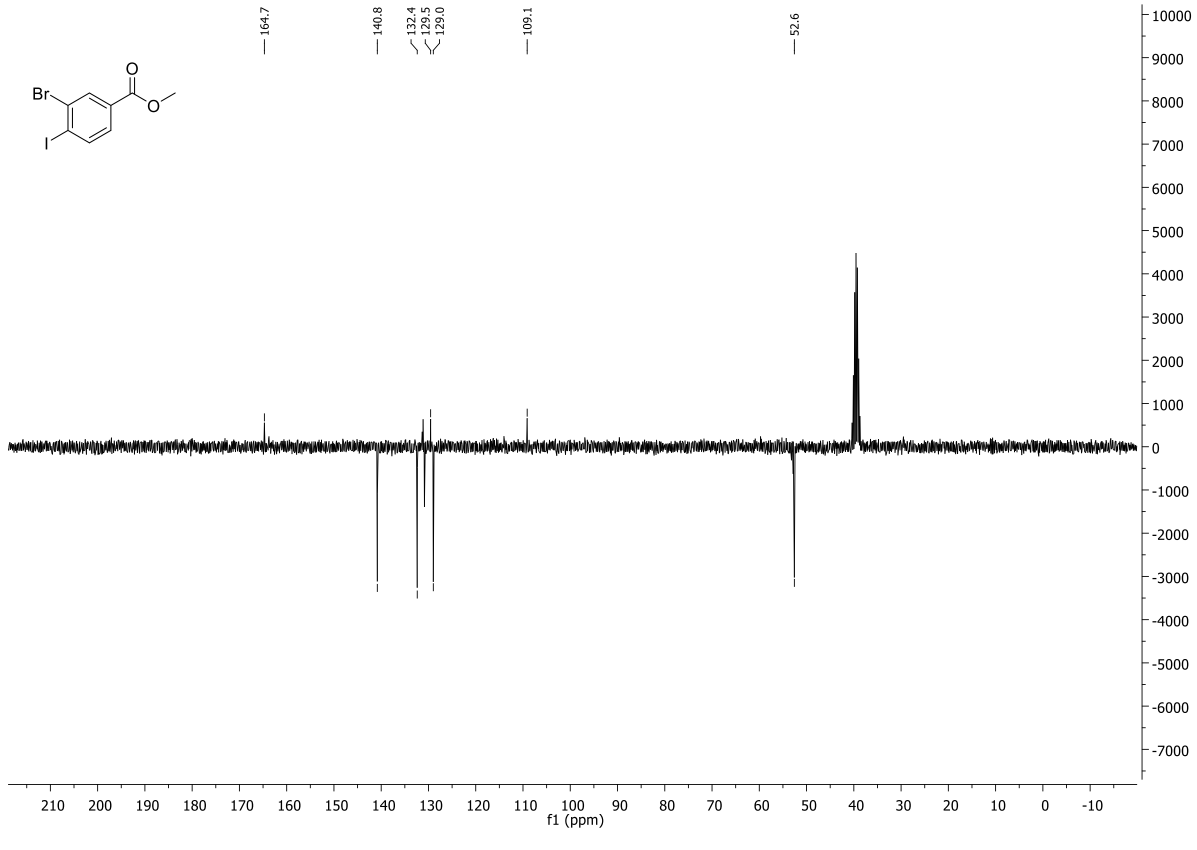

Figure S92. ^{13}C-NMR (75.53 MHz, DMSO-d_6) of compound **22**.

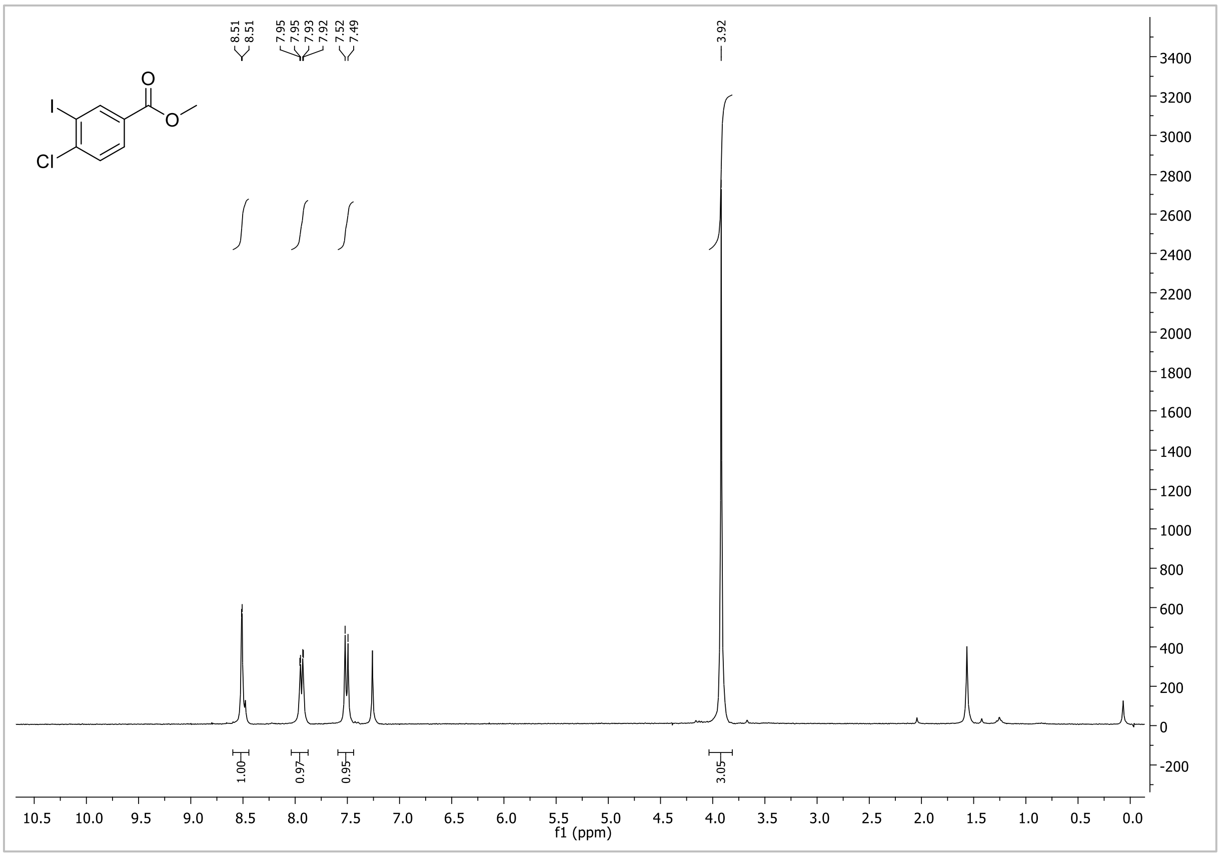

Figure S93. ^{1}H-NMR (300.36 MHz, $CDCl_3$) of compound **21**.

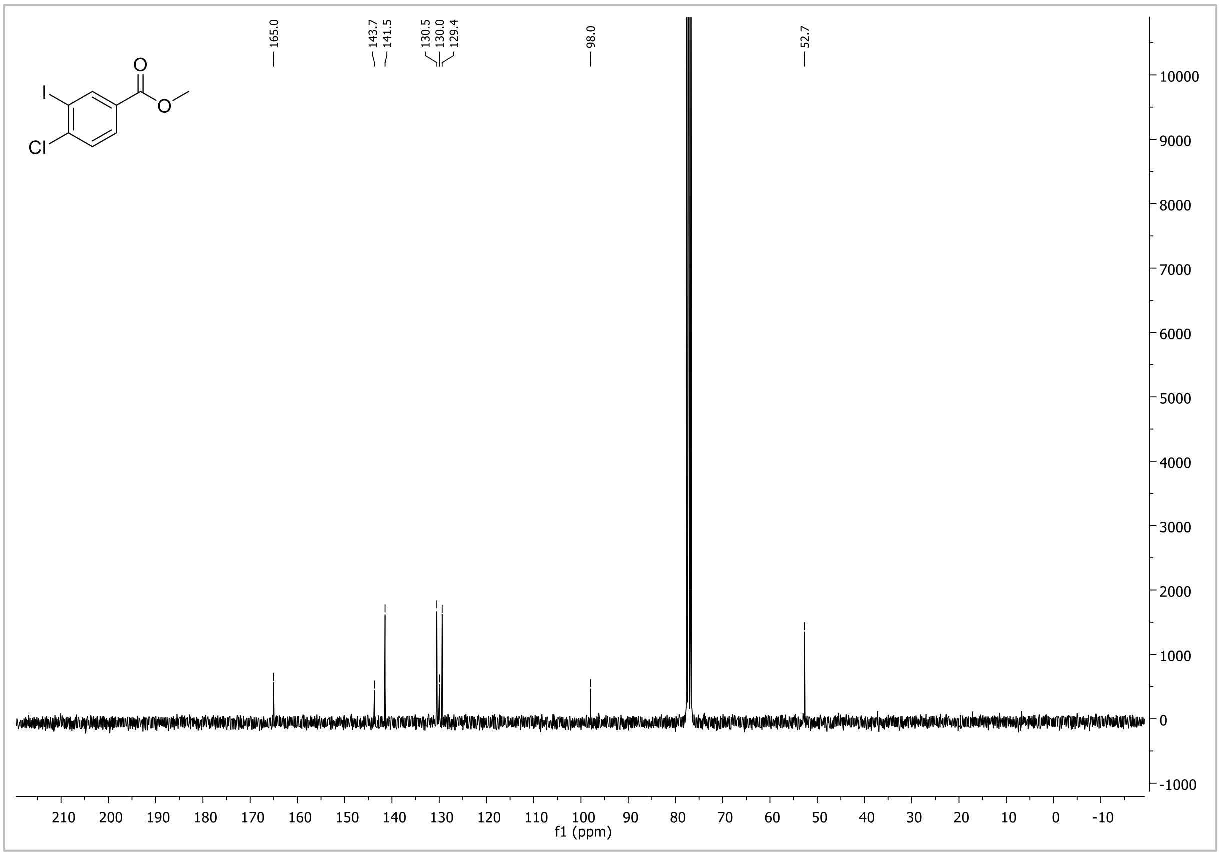

Figure S94. ^{13}C-NMR (75.53 MHz, $CDCl_3$) of compound **21**.

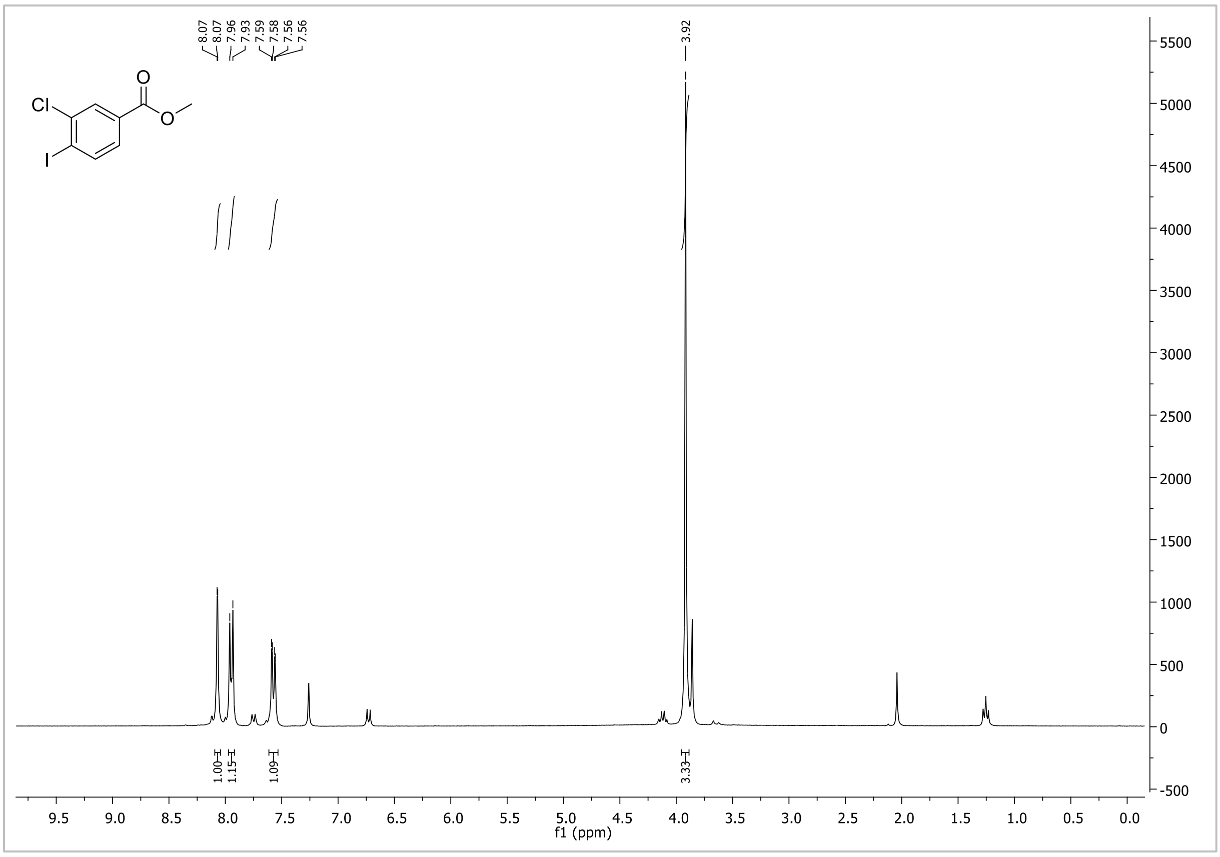

Figure S95. ^{1}H-NMR (300.36 MHz, $CDCl_3$) of compound **23**.

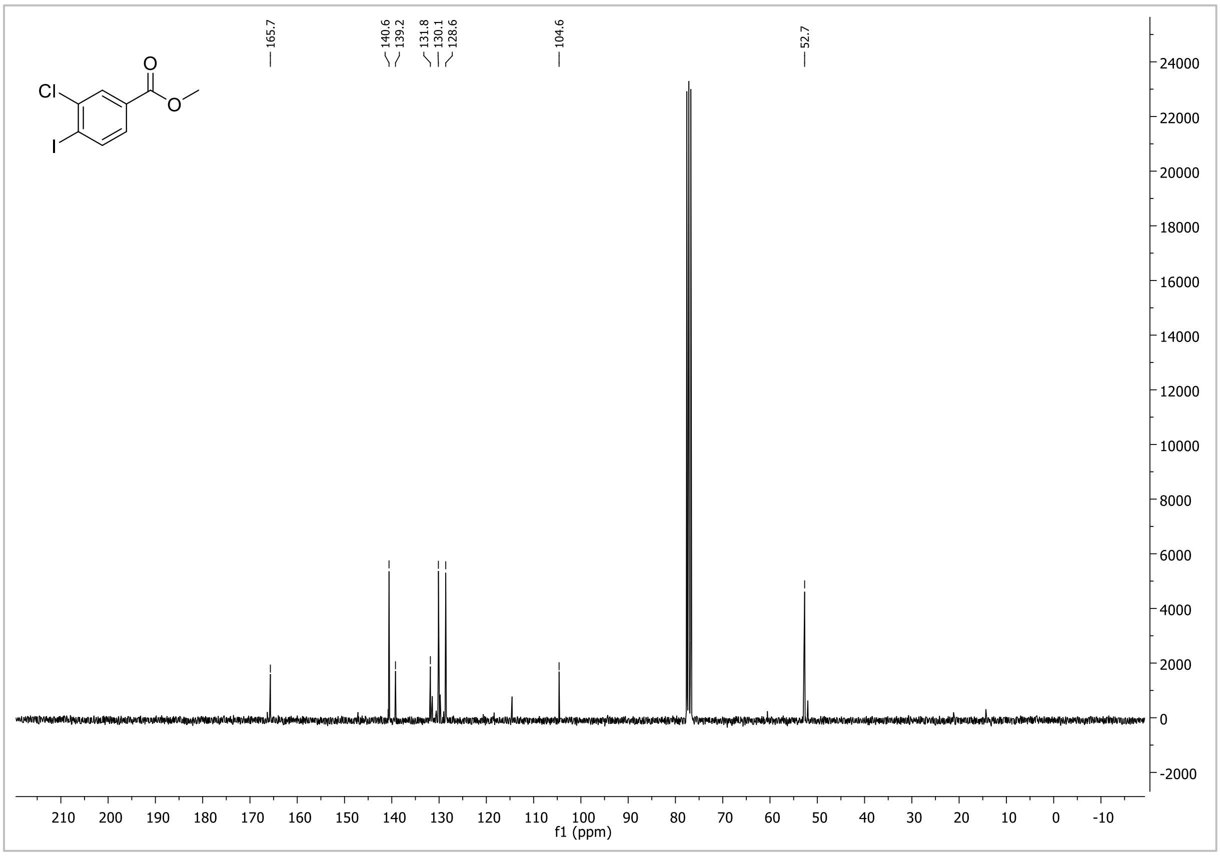

Figure S96. ^{13}C-NMR (75.53 MHz, $CDCl_3$) of compound **23**.

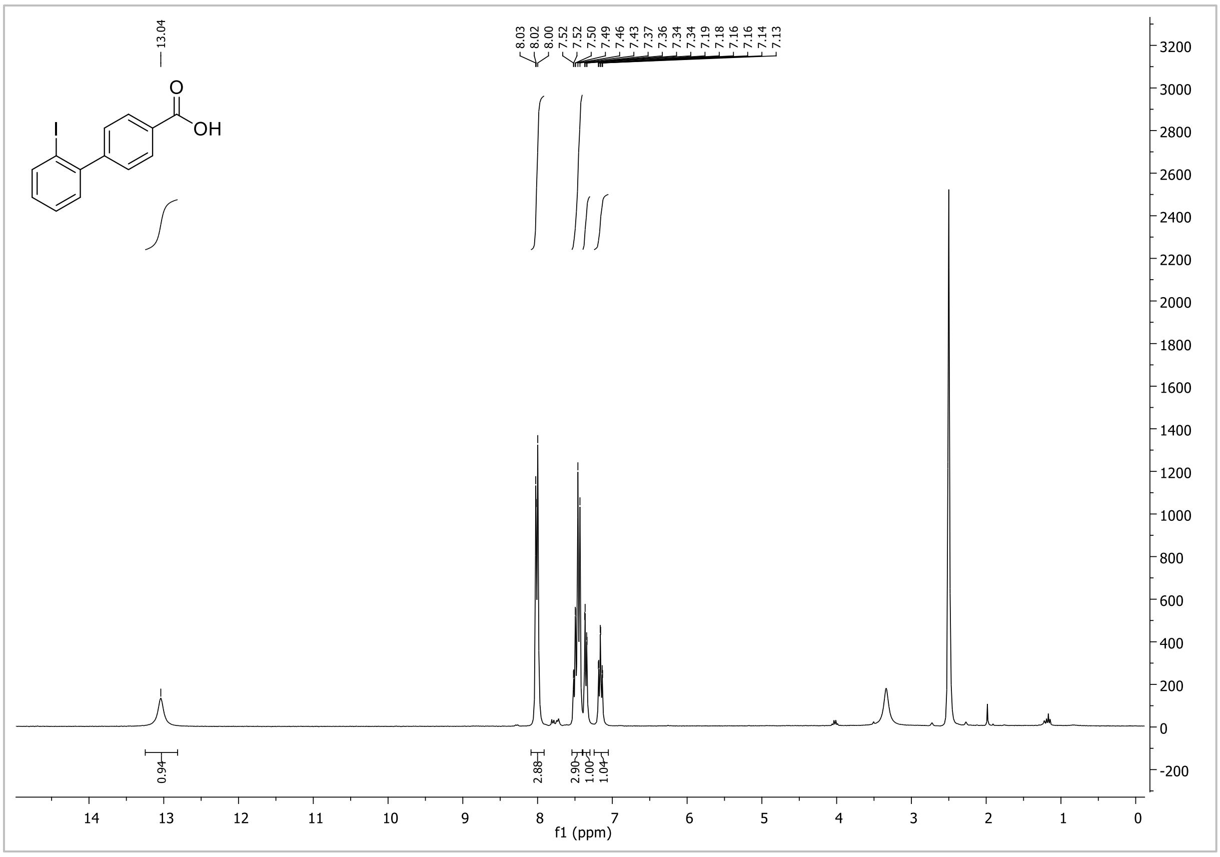

Figure S97. ^{1}H-NMR (300.36 MHz, DMSO-d_6) of compound **5**.

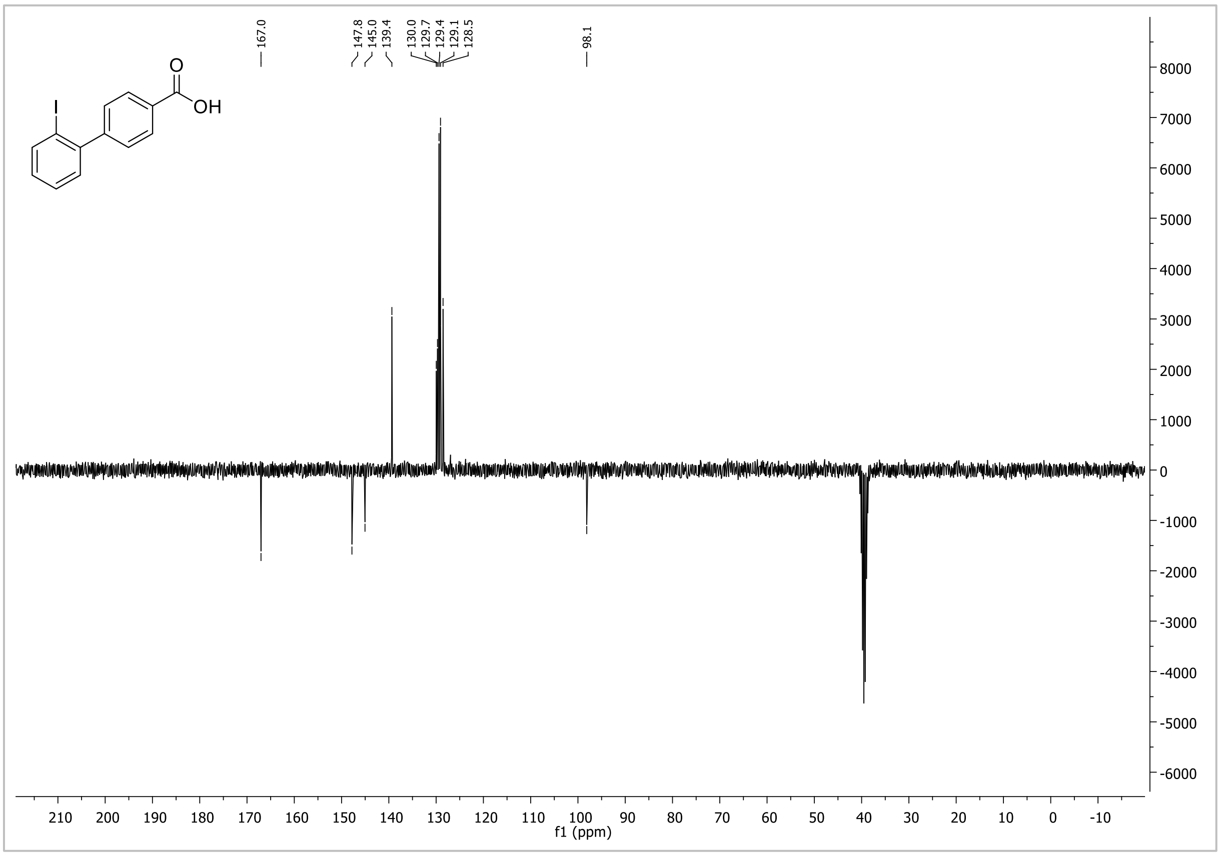

Figure S98. ^{13}C-NMR (75.53 MHz, DMSO-d_6) of compound **5**.

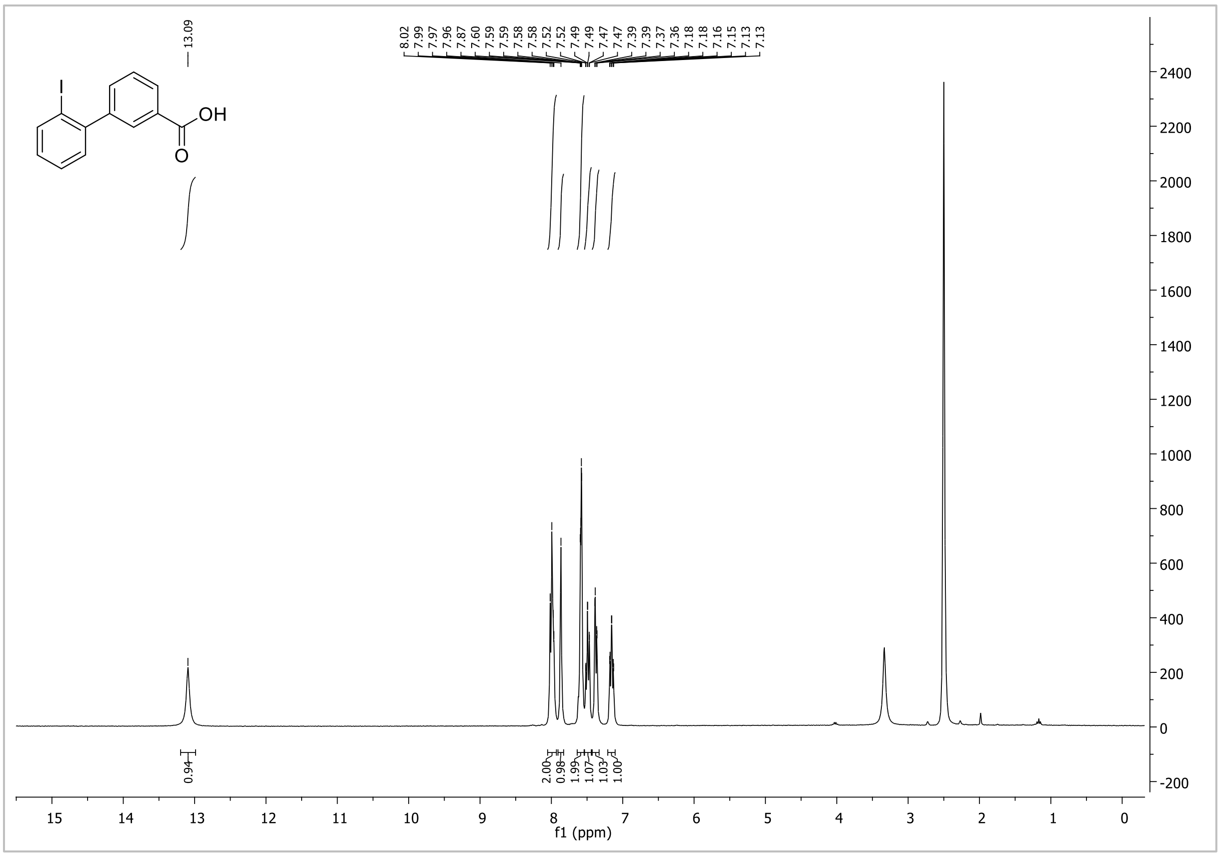

Figure S99. ^{1}H-NMR (300.36 MHz, DMSO-d_6) of compound **6**.

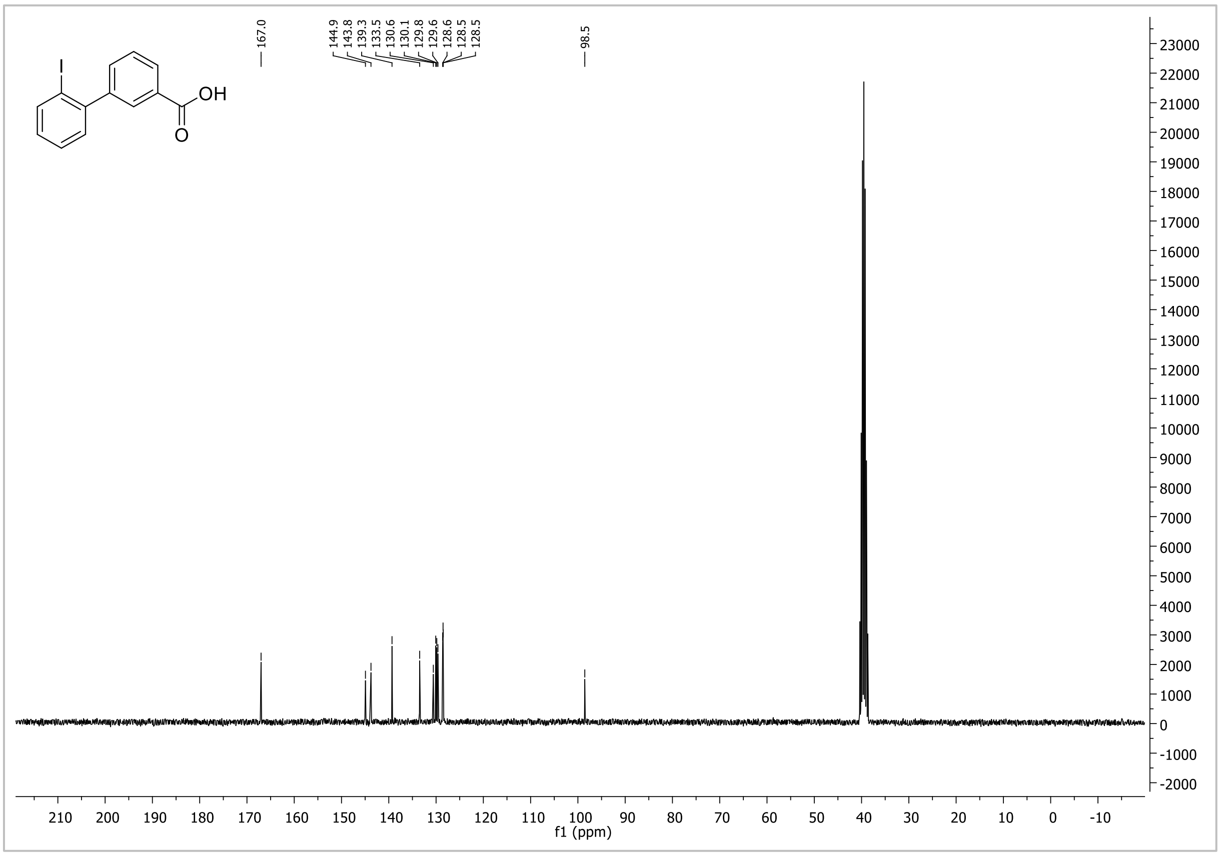

Figure S100. ^{13}C-NMR (75.53 MHz, DMSO-d_6) of compound **6**.

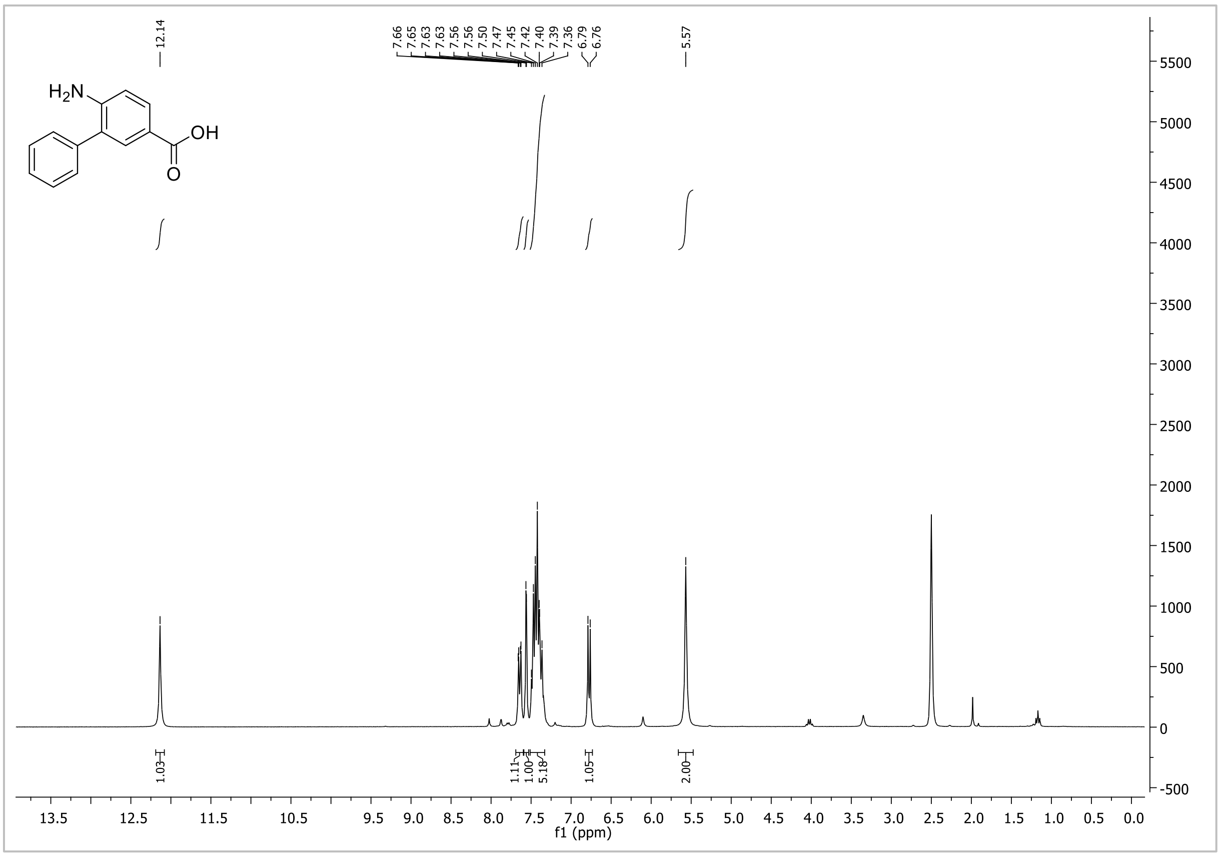

Figure S101. ^{1}H-NMR (300.36 MHz, DMSO-d_6) of compound **24**.

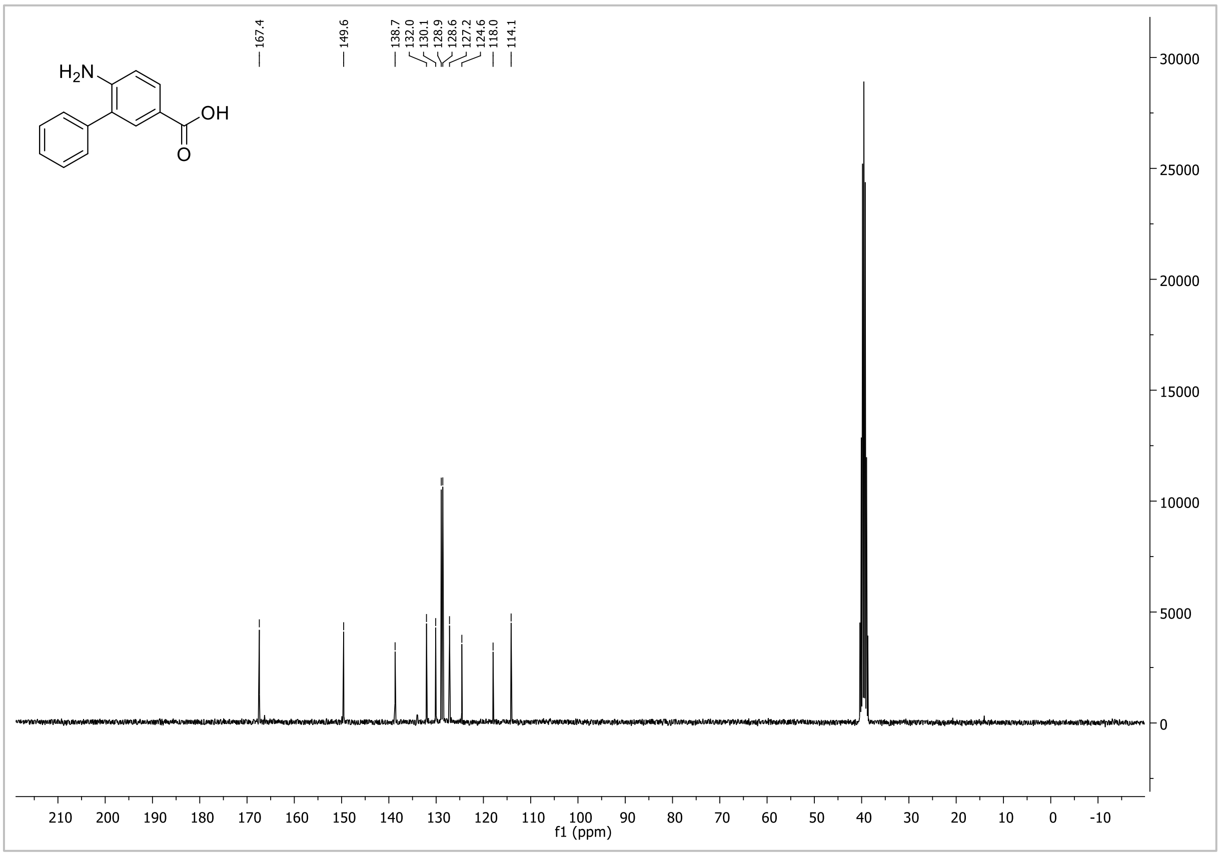

Figure S102. ^{13}C-NMR (75.53 MHz, DMSO-d_6) of compound **24**.

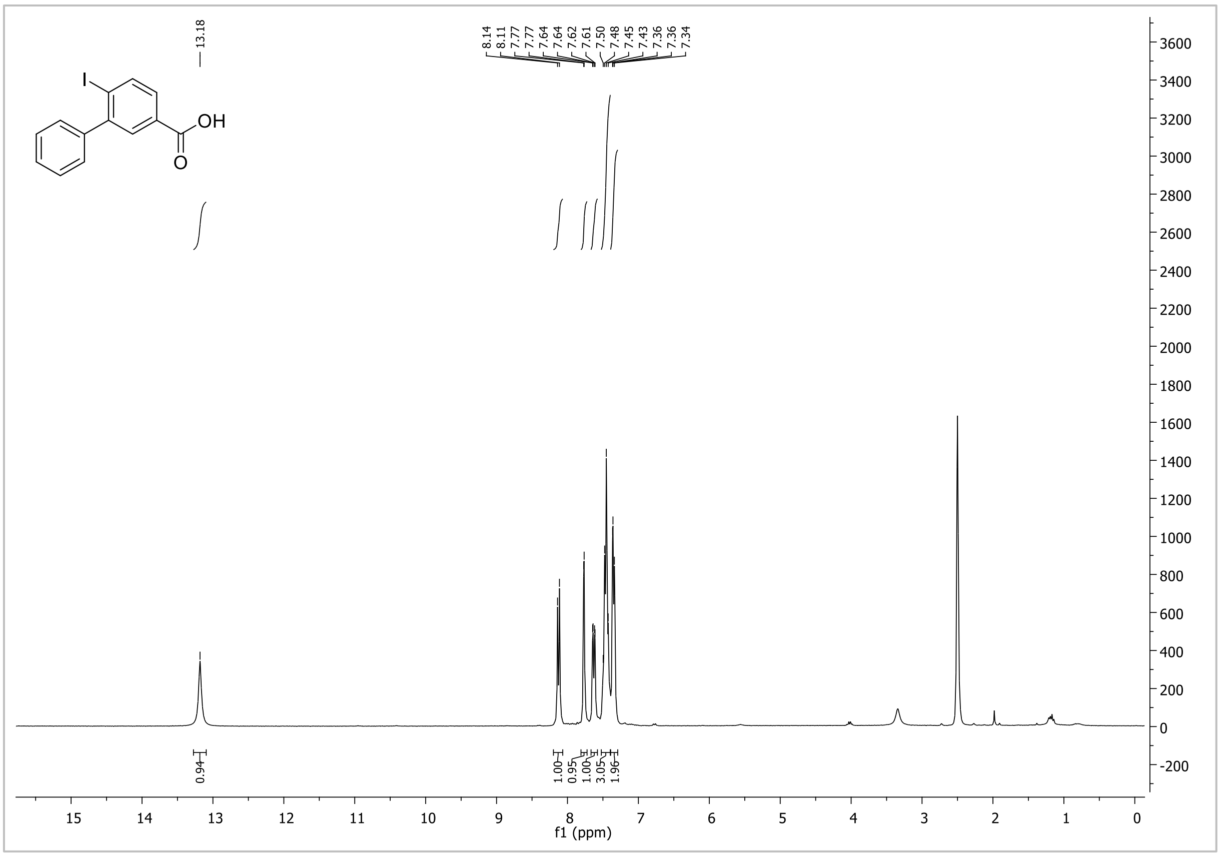

Figure S103. ^{1}H-NMR (300.36 MHz, DMSO-d_6) of compound **4**.

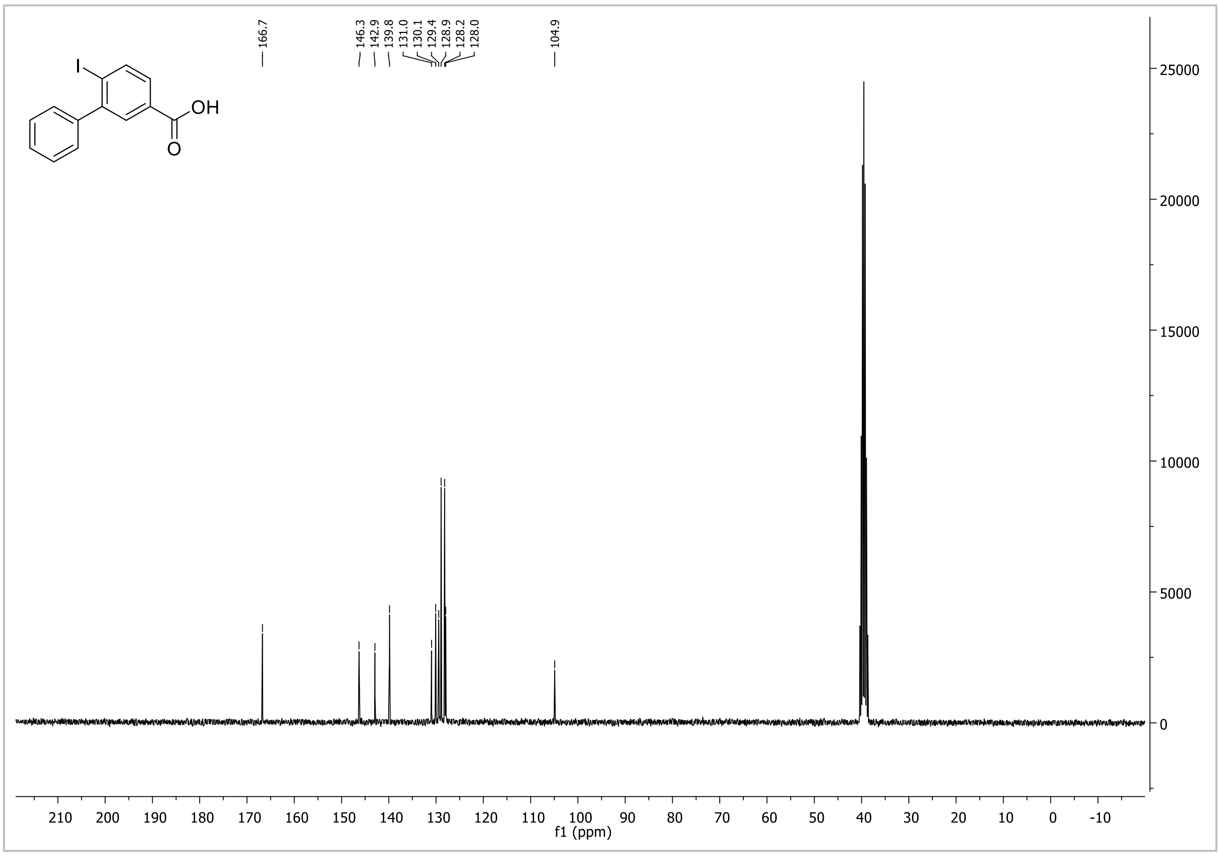

Figure S104. ^{13}C-NMR (75.53 MHz, DMSO-d_6) of compound **4**.

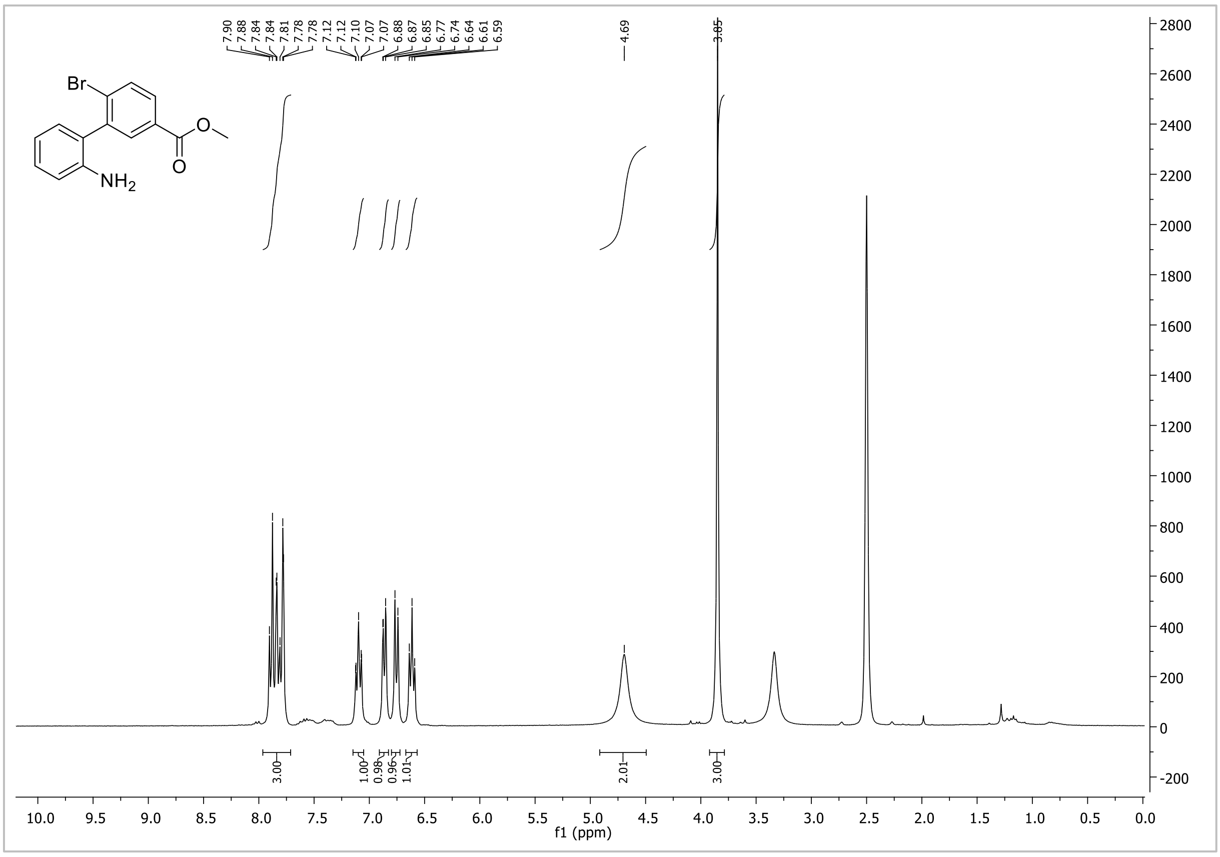

Figure S105. ^{1}H-NMR (300.36 MHz, DMSO-d_6) of compound **25**.

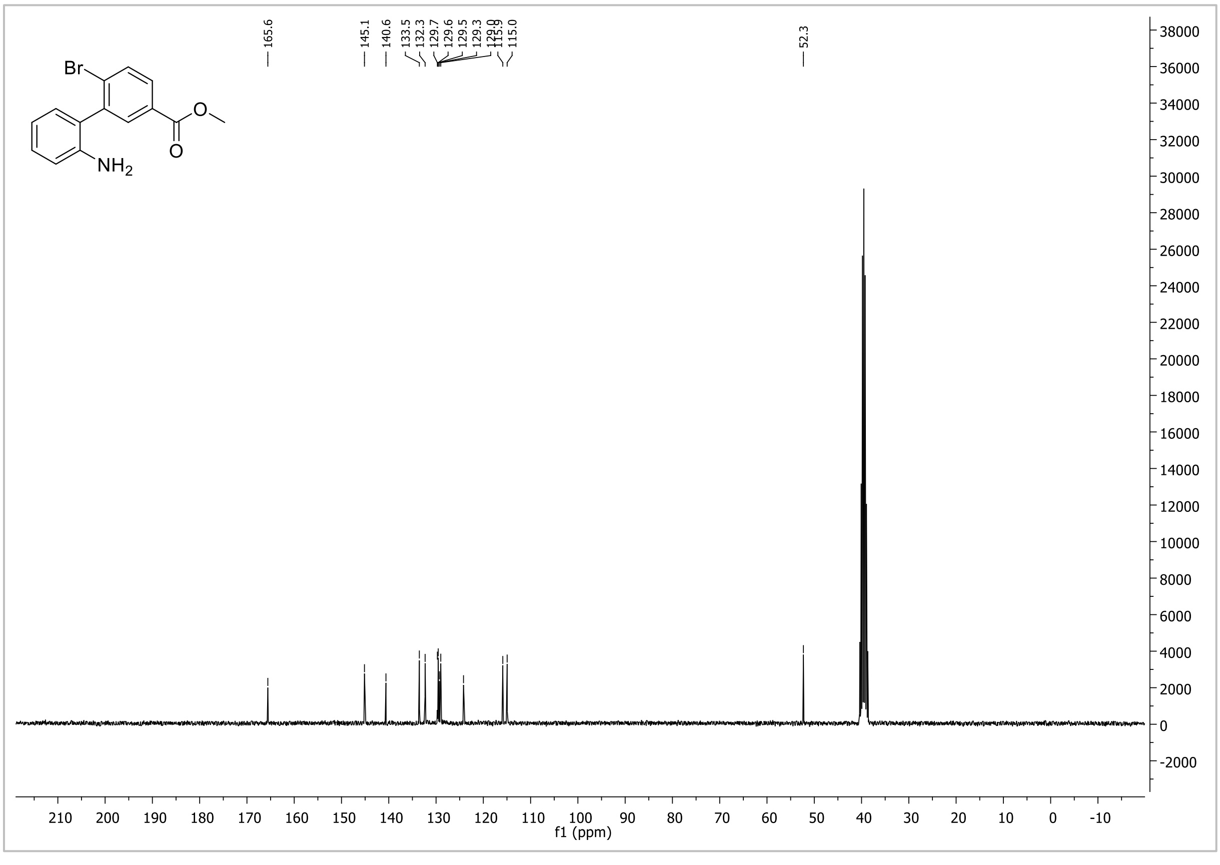

Figure S106. ^{13}C-NMR (75.53 MHz, DMSO-d_6) of compound **25**.

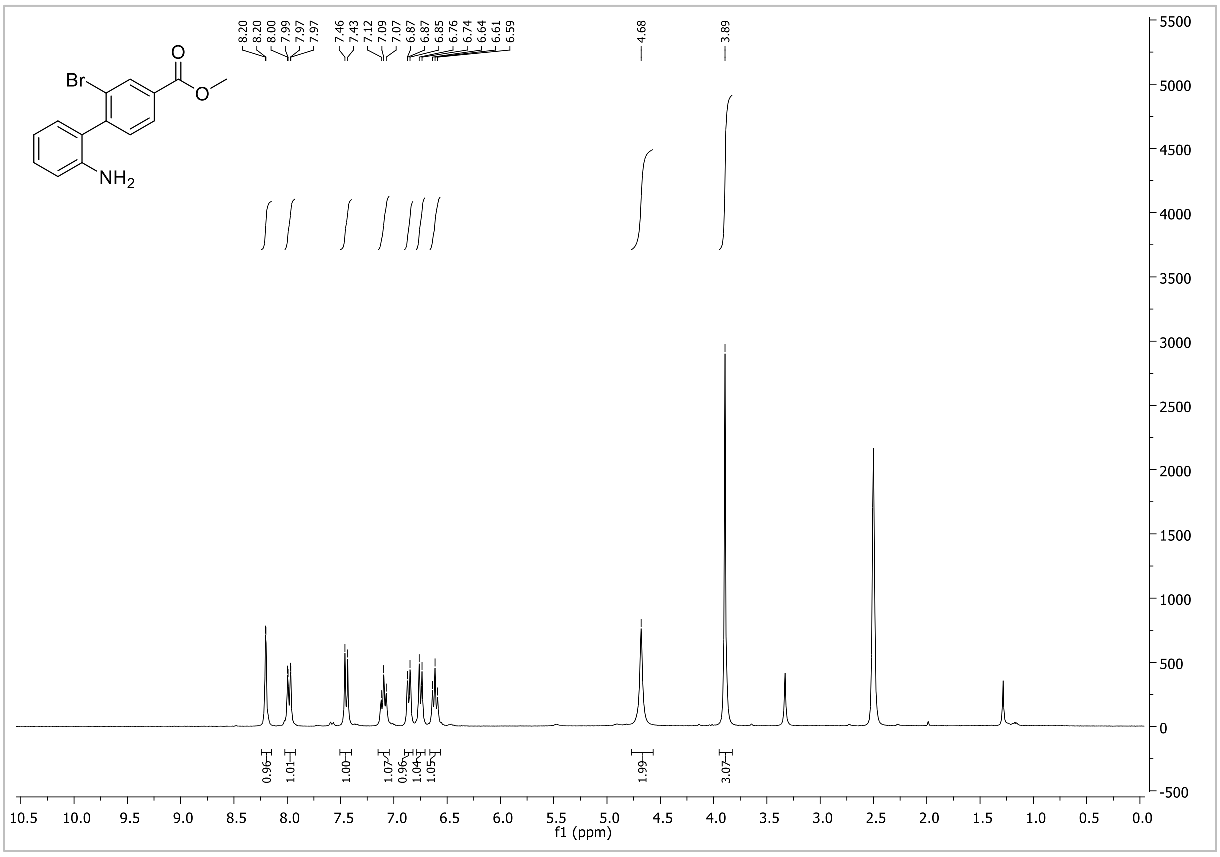

Figure S107. ^{1}H-NMR (300.36 MHz, DMSO-d_6) of compound **26**.

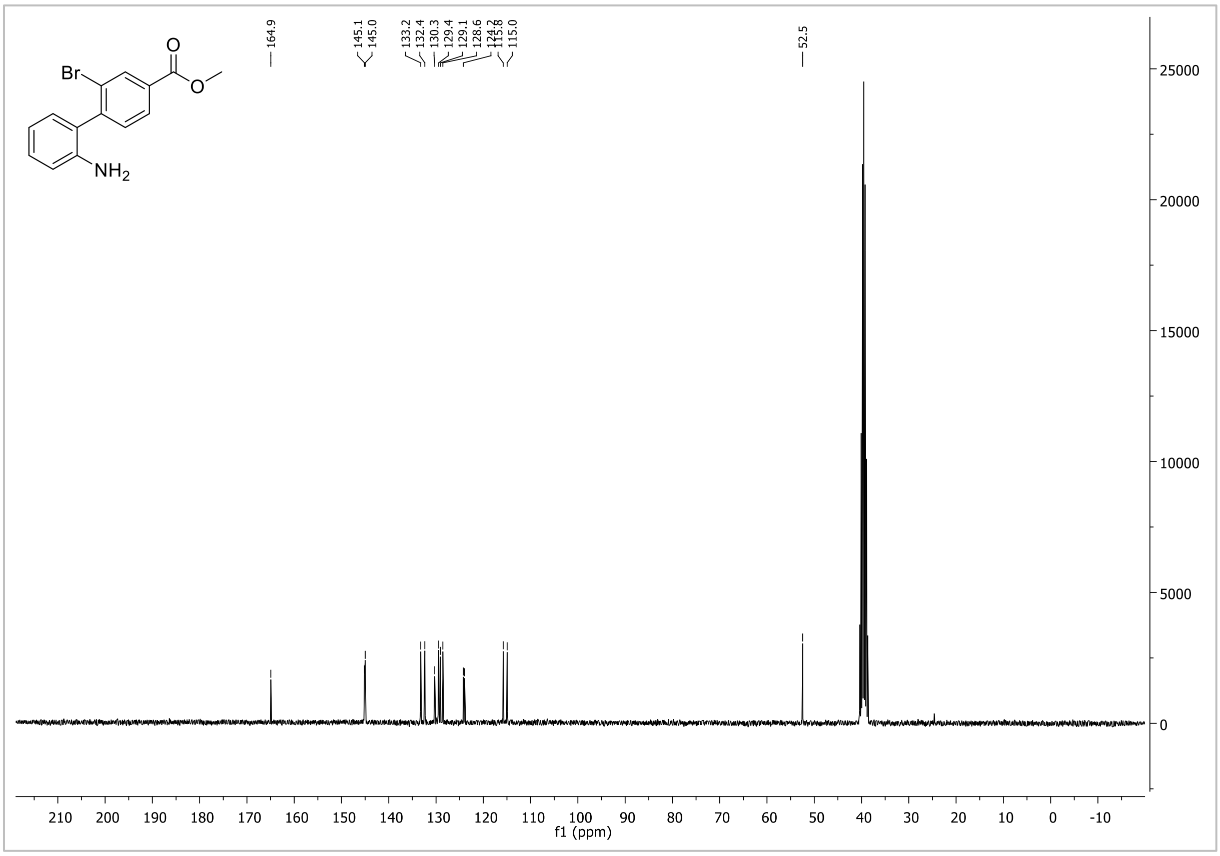

Figure S108. ^{13}C-NMR (75.53 MHz, DMSO-d_6) of compound **26**.

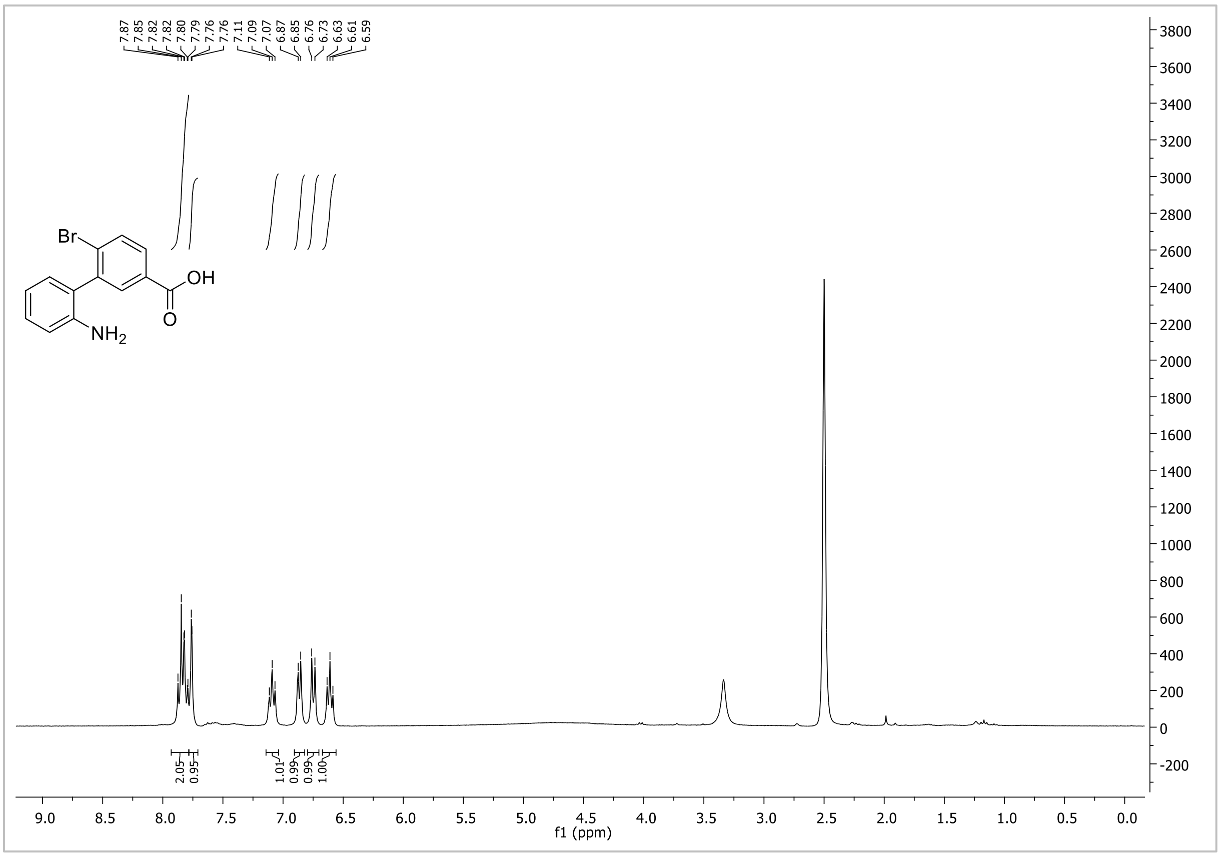

Figure S109. ^{1}H-NMR (300.36 MHz, DMSO-d_6) of compound **7**.

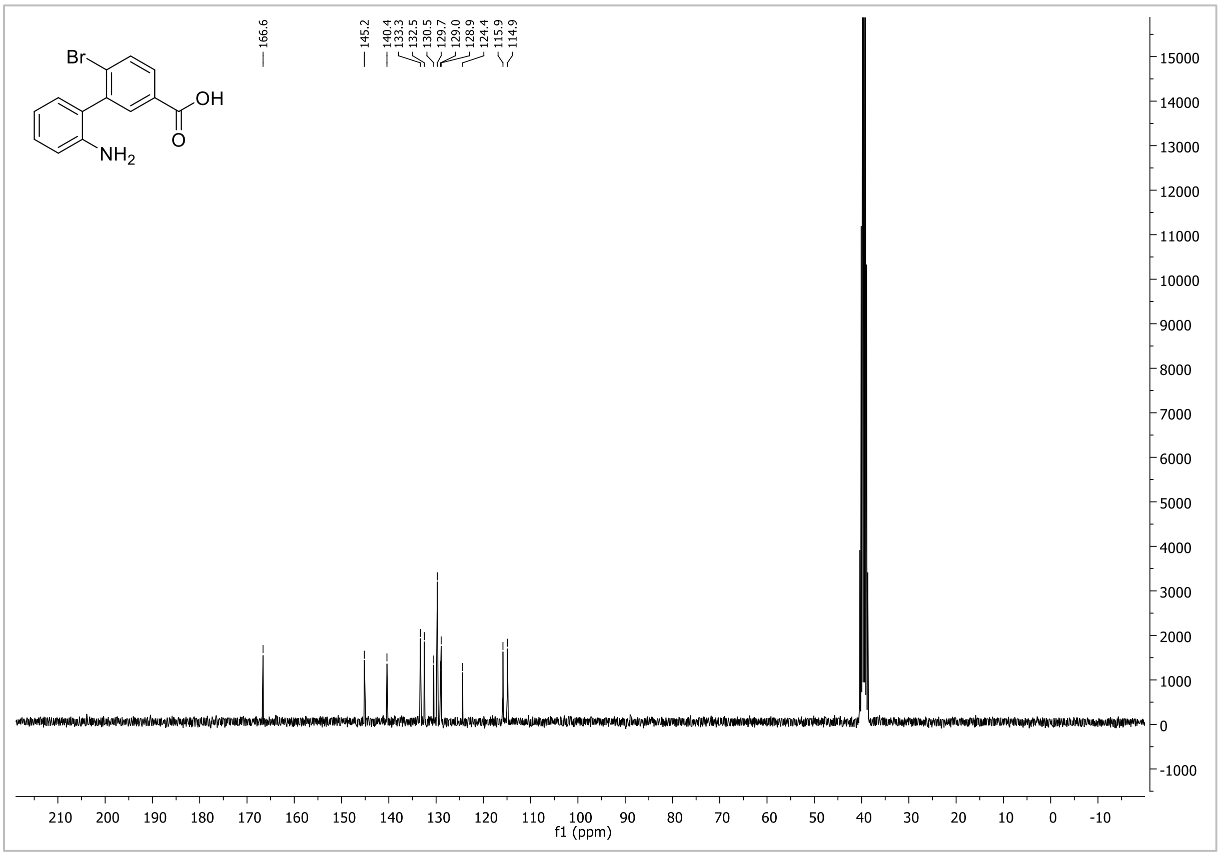

Figure S110. ^{13}C-NMR (75.53 MHz, DMSO-d_6) of compound **7**.

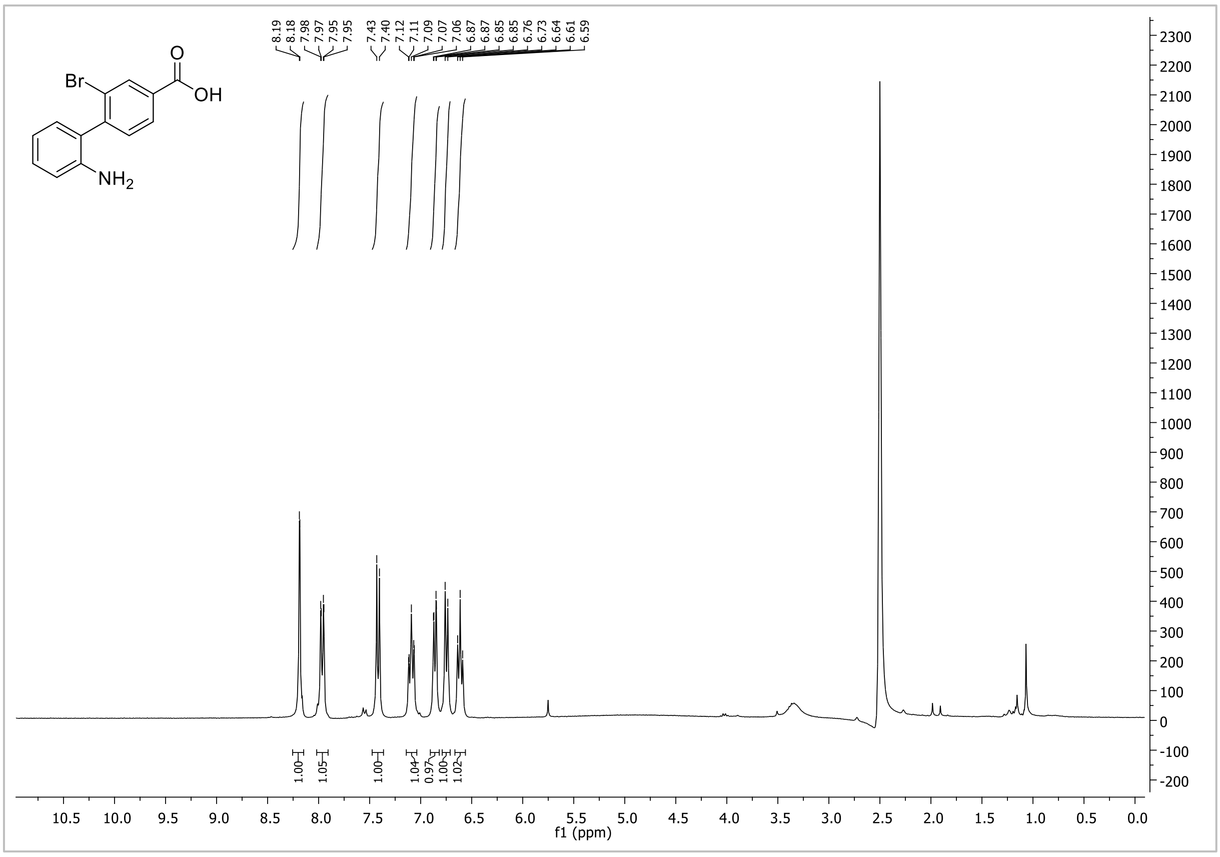

Figure S111. ^{1}H-NMR (300.36 MHz, DMSO-d_6) of compound **8**.

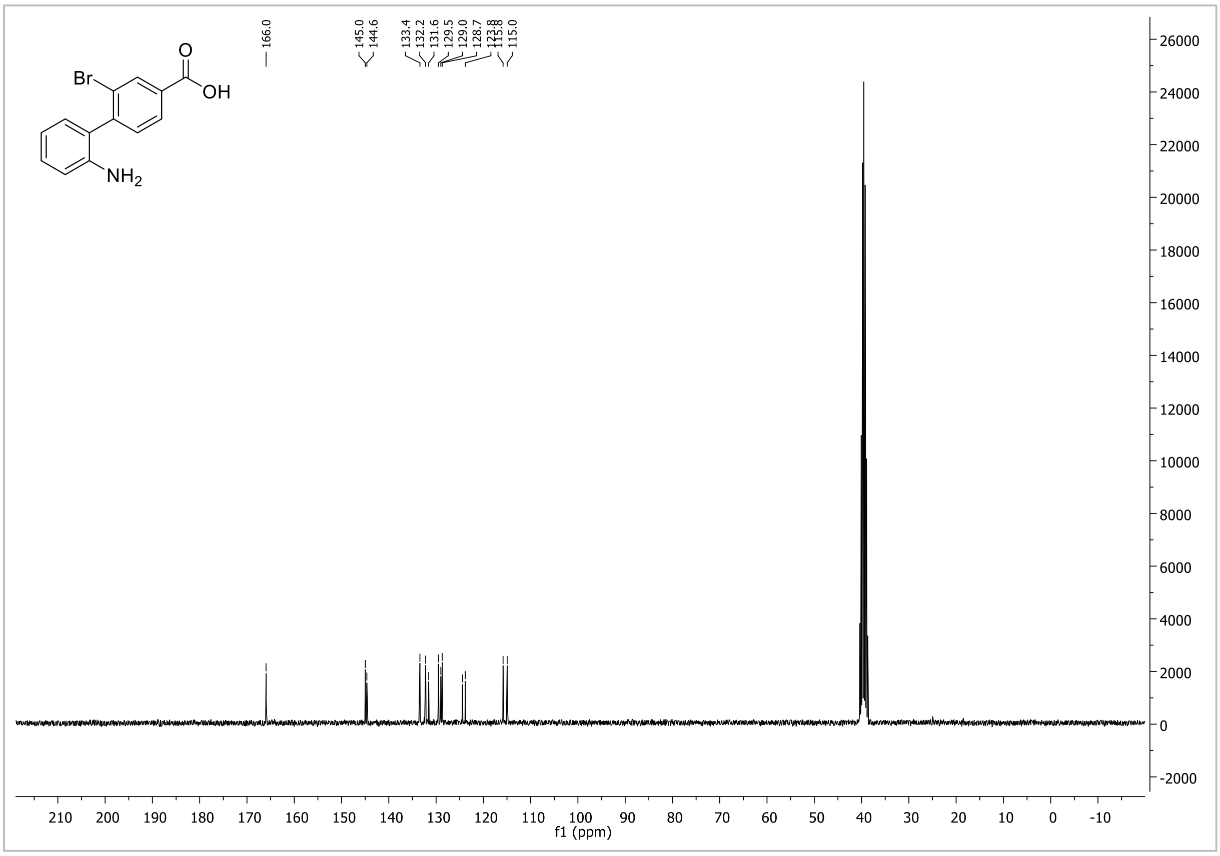

Figure S112. ^{13}C-NMR (75.53 MHz, DMSO-d_6) of compound **8**.

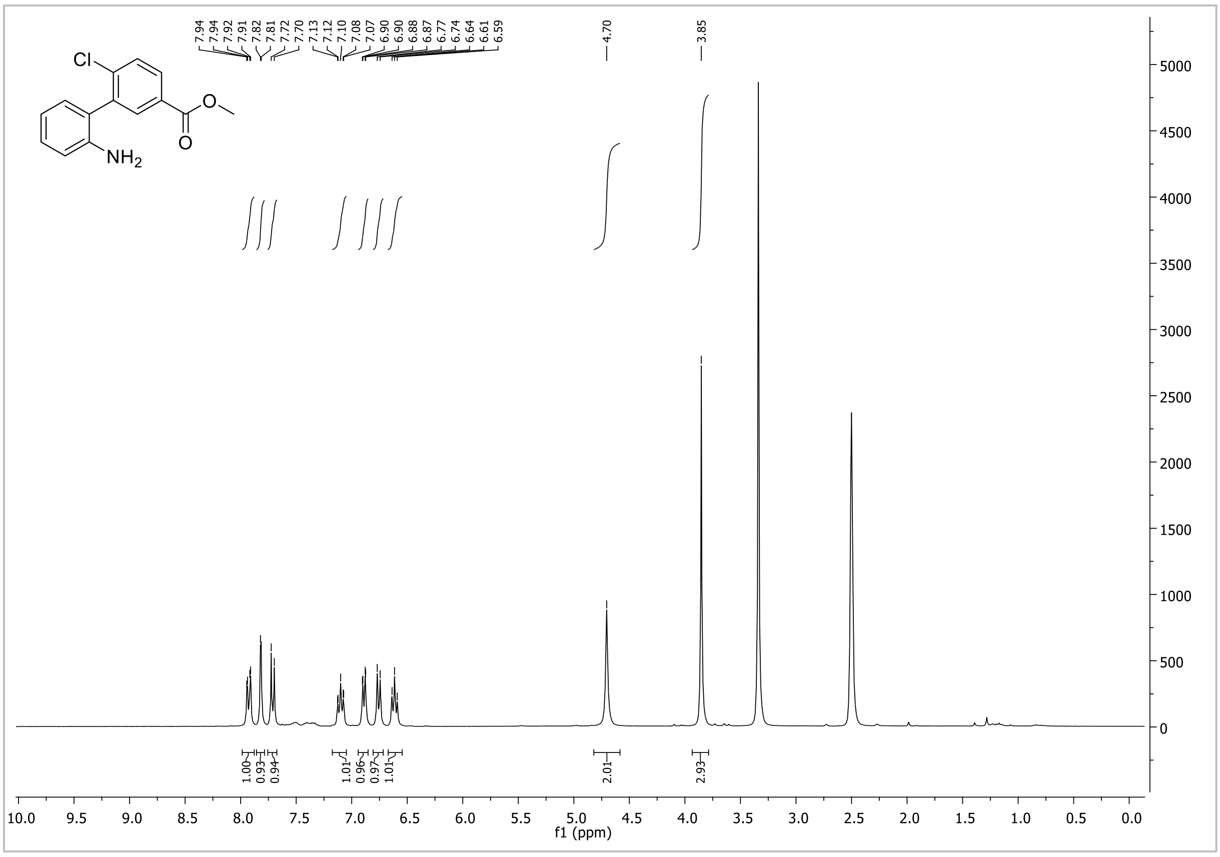

Figure S113. ^{1}H-NMR (300.36 MHz, DMSO-d_6) of compound **27**.

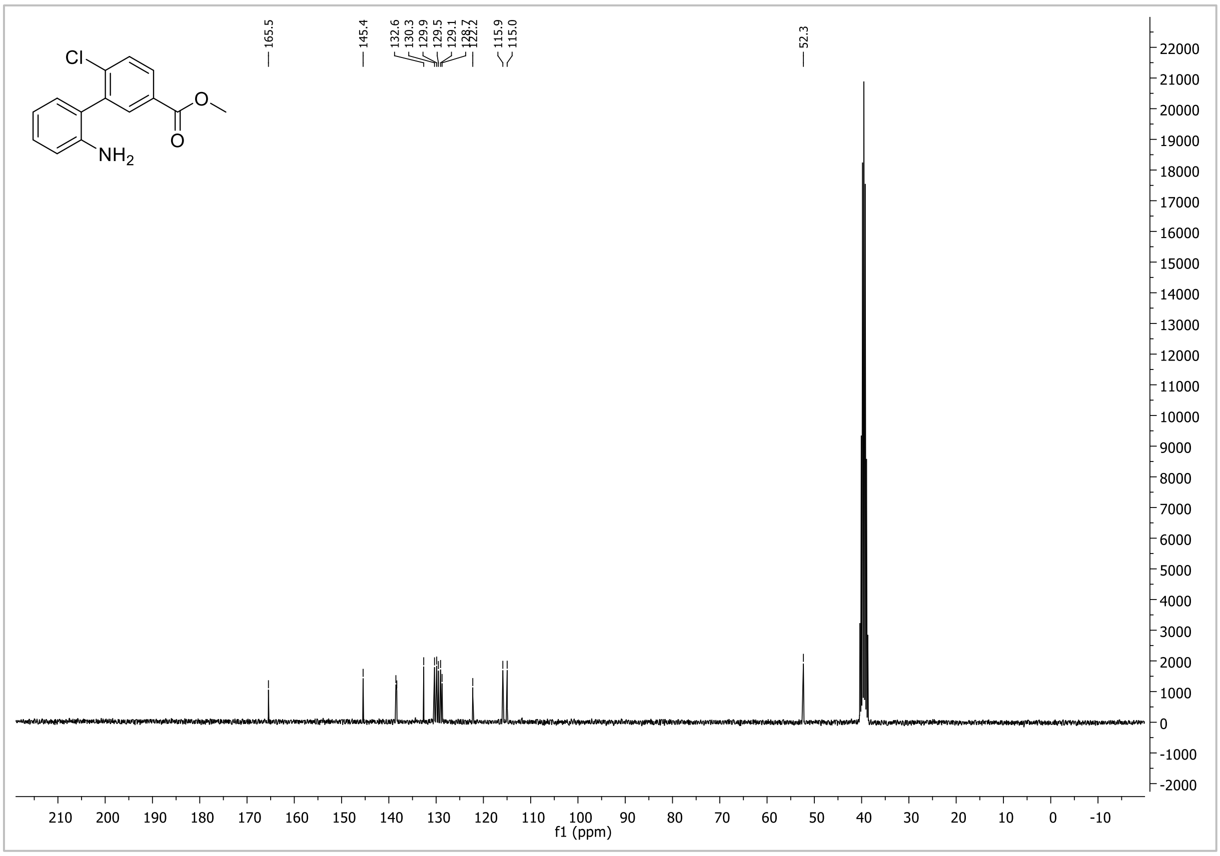

Figure S114. ^{13}C-NMR (75.53 MHz, DMSO-d_6) of compound **27**.

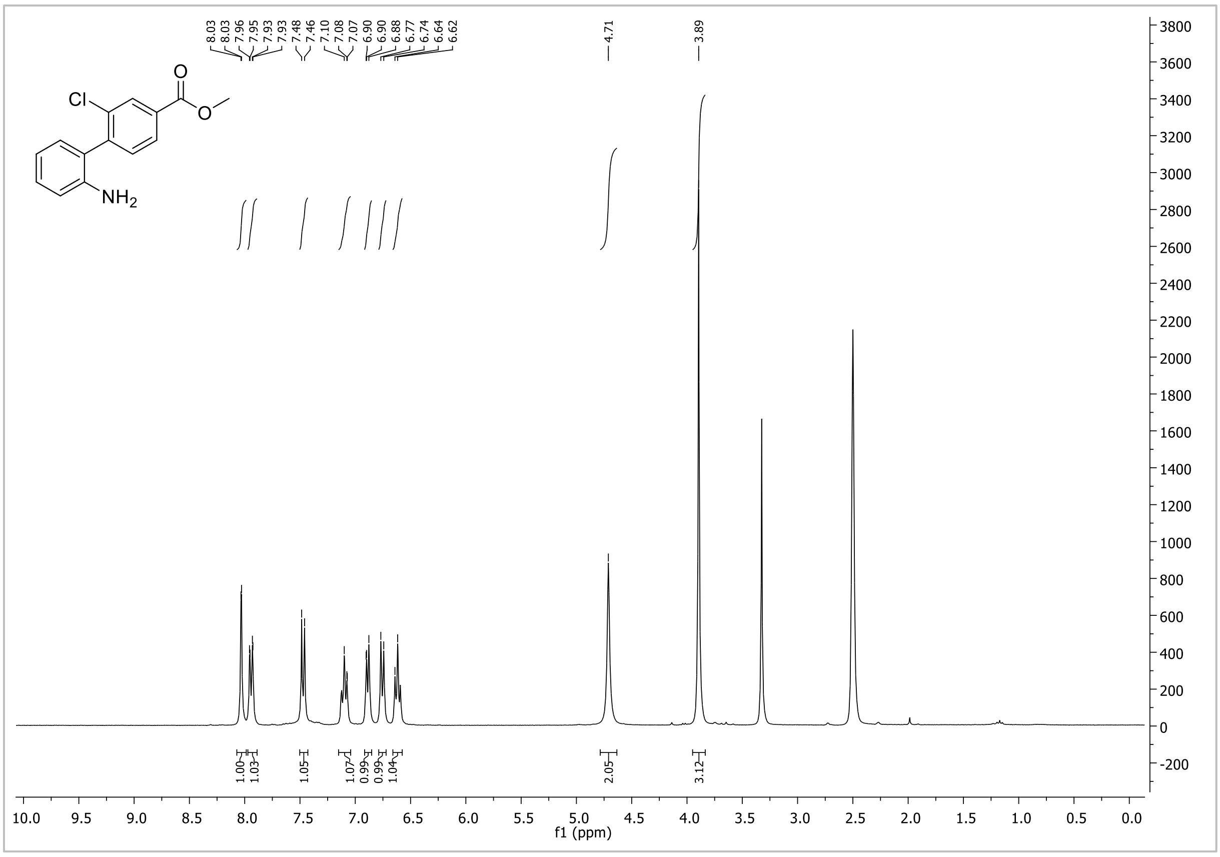

Figure S115. ^{1}H-NMR (300.36 MHz, DMSO-d_6) of compound **28**.

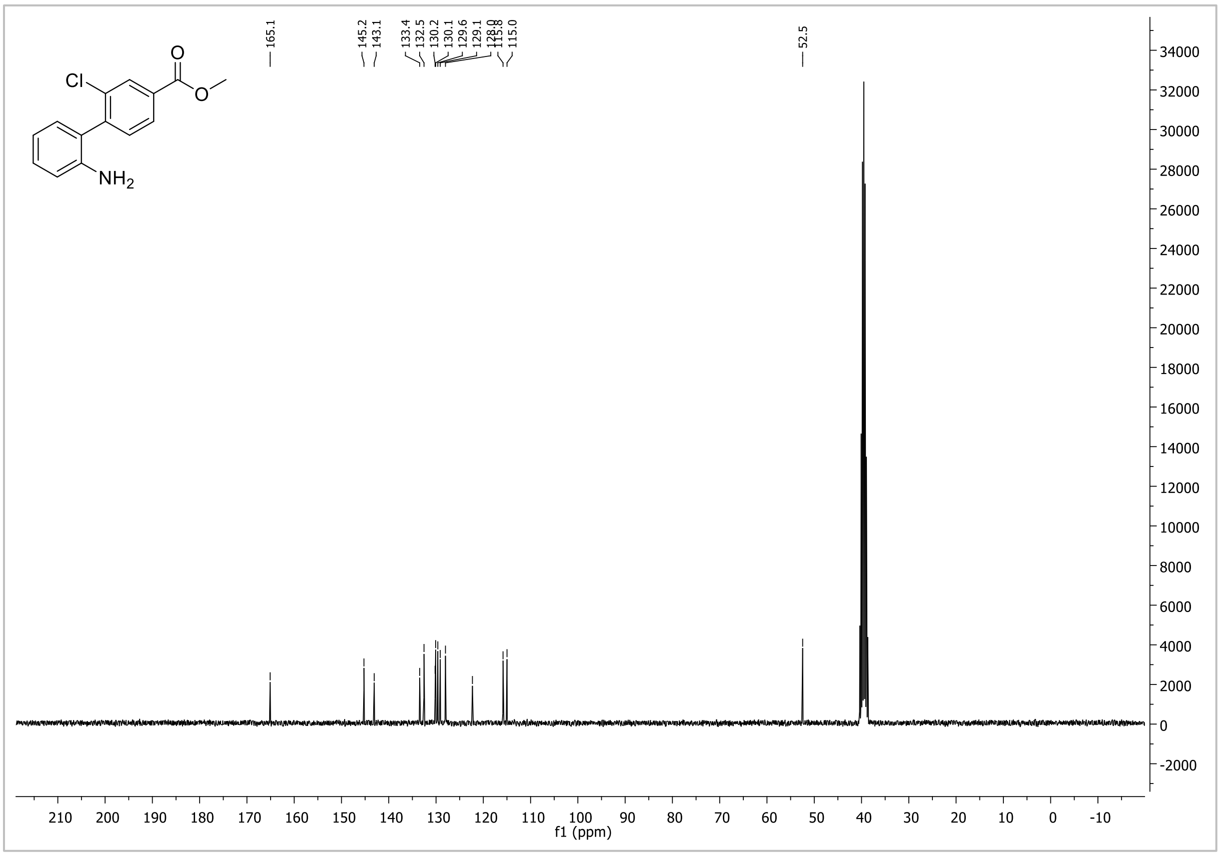

Figure S116. ^{13}C-NMR (75.53 MHz, DMSO-d_6) of compound **28**.

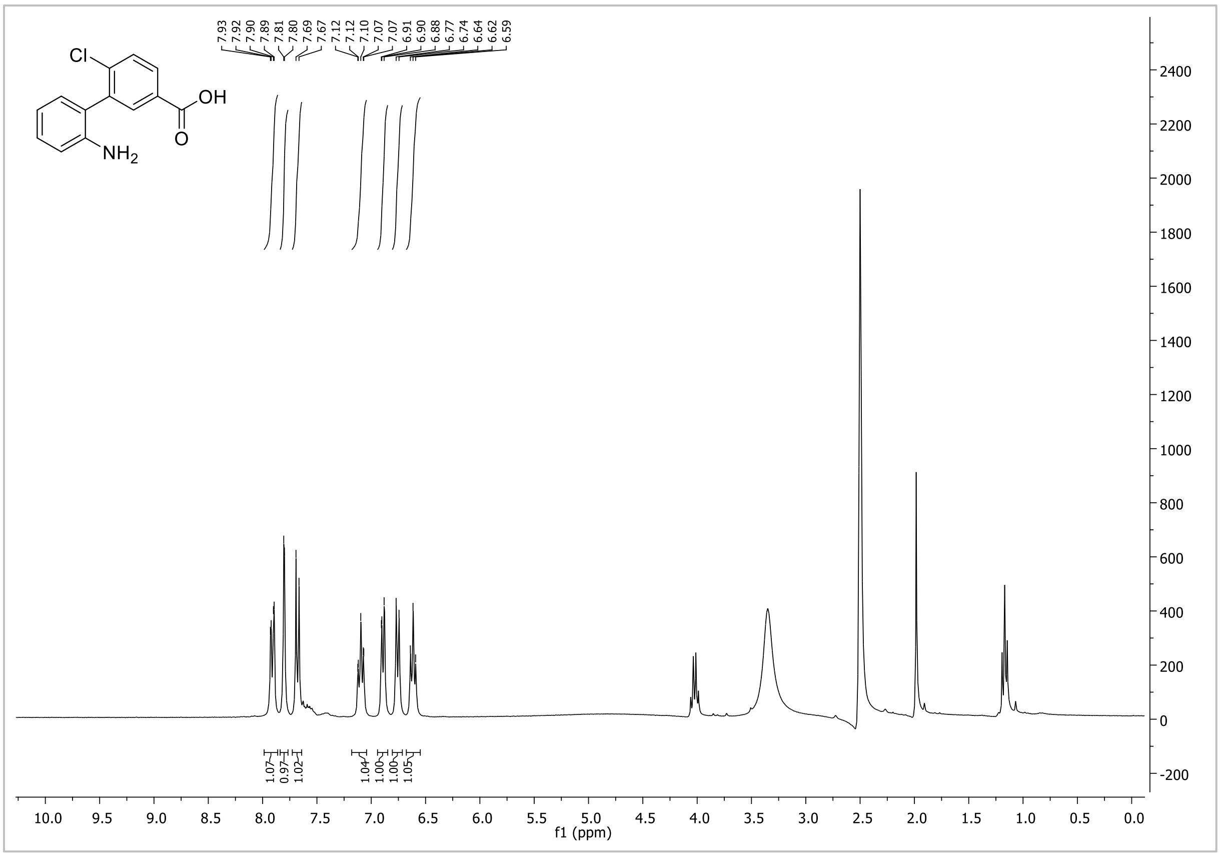

Figure S117. ^{1}H-NMR (300.36 MHz, DMSO-d_6) of compound **9**.

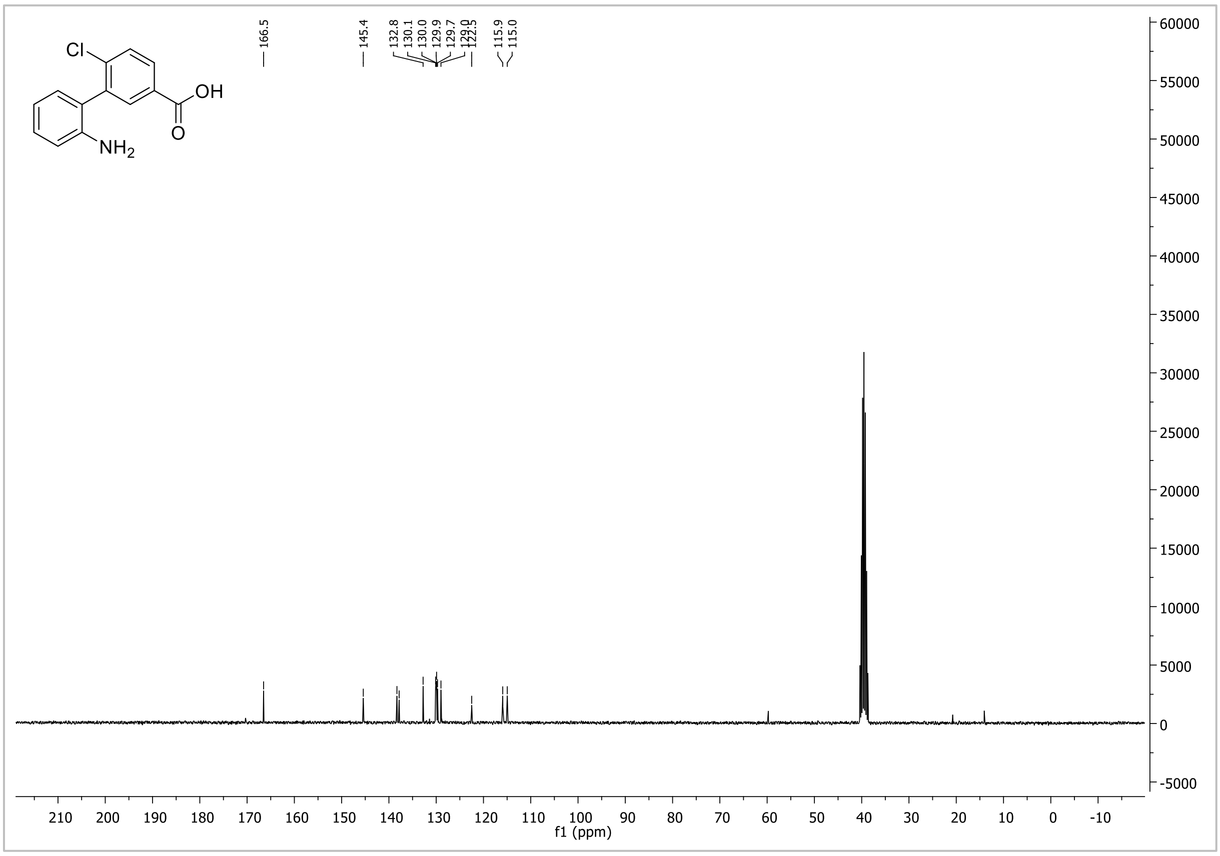

Figure S118. ^{13}C-NMR (75.53 MHz, DMSO-d_6) of compound **9**.

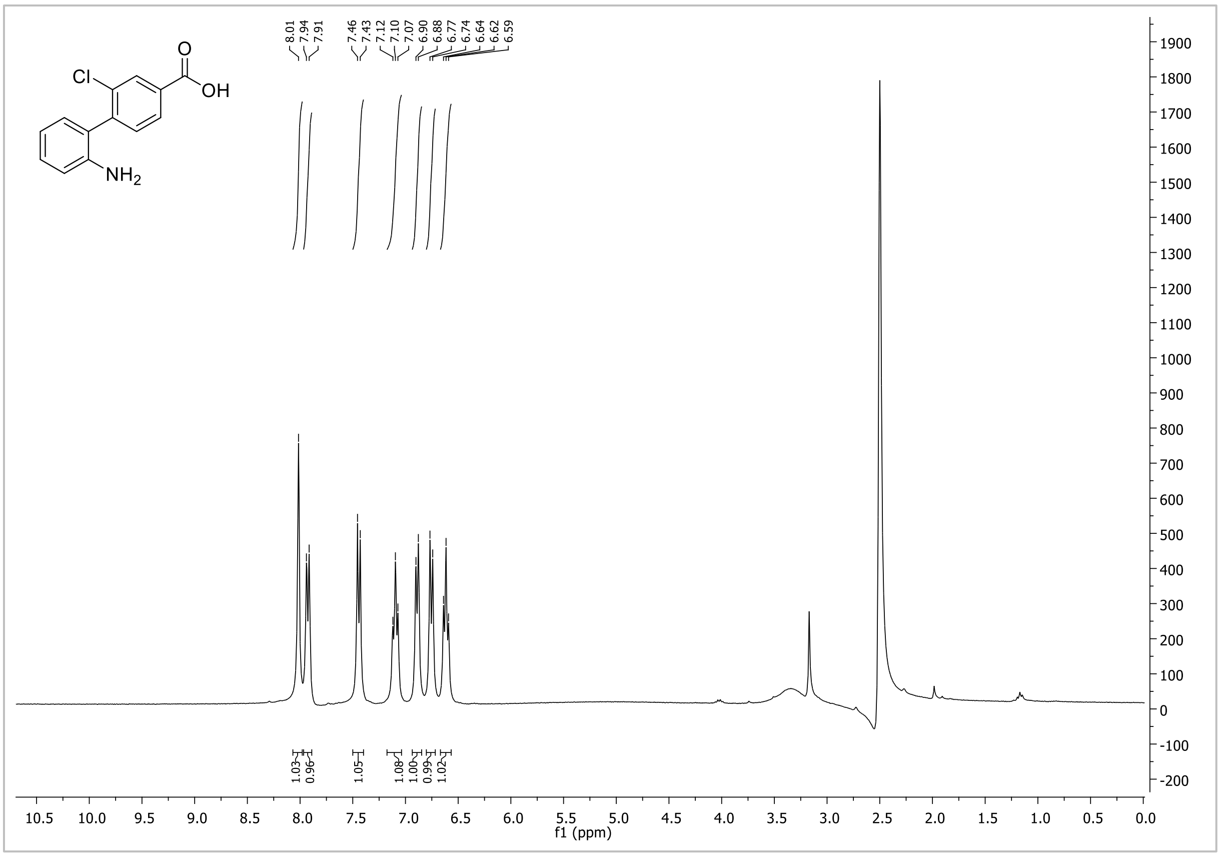

Figure S119. ^{1}H-NMR (300.36 MHz, DMSO-d_6) of compound **10**.

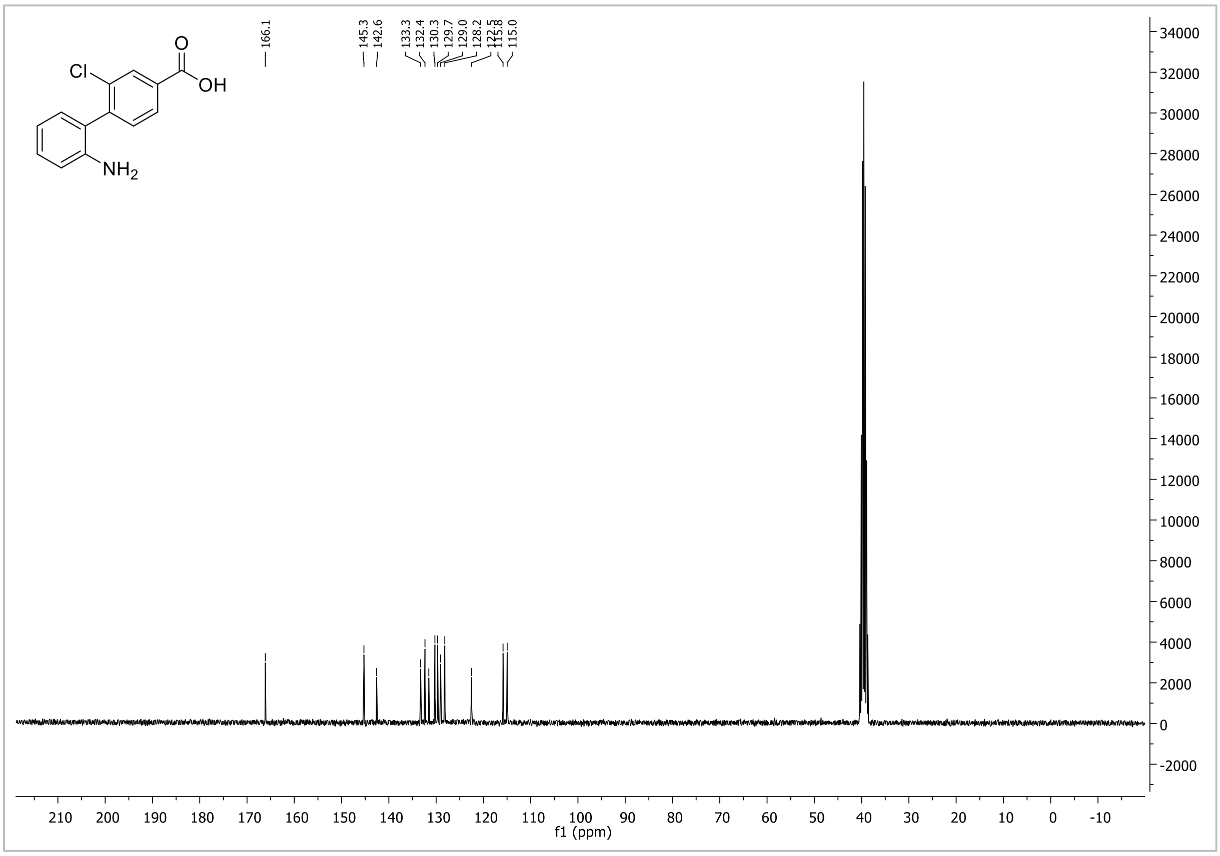

Figure S120. ^{13}C-NMR (75.53 MHz, DMSO-d_6) of compound **10**.

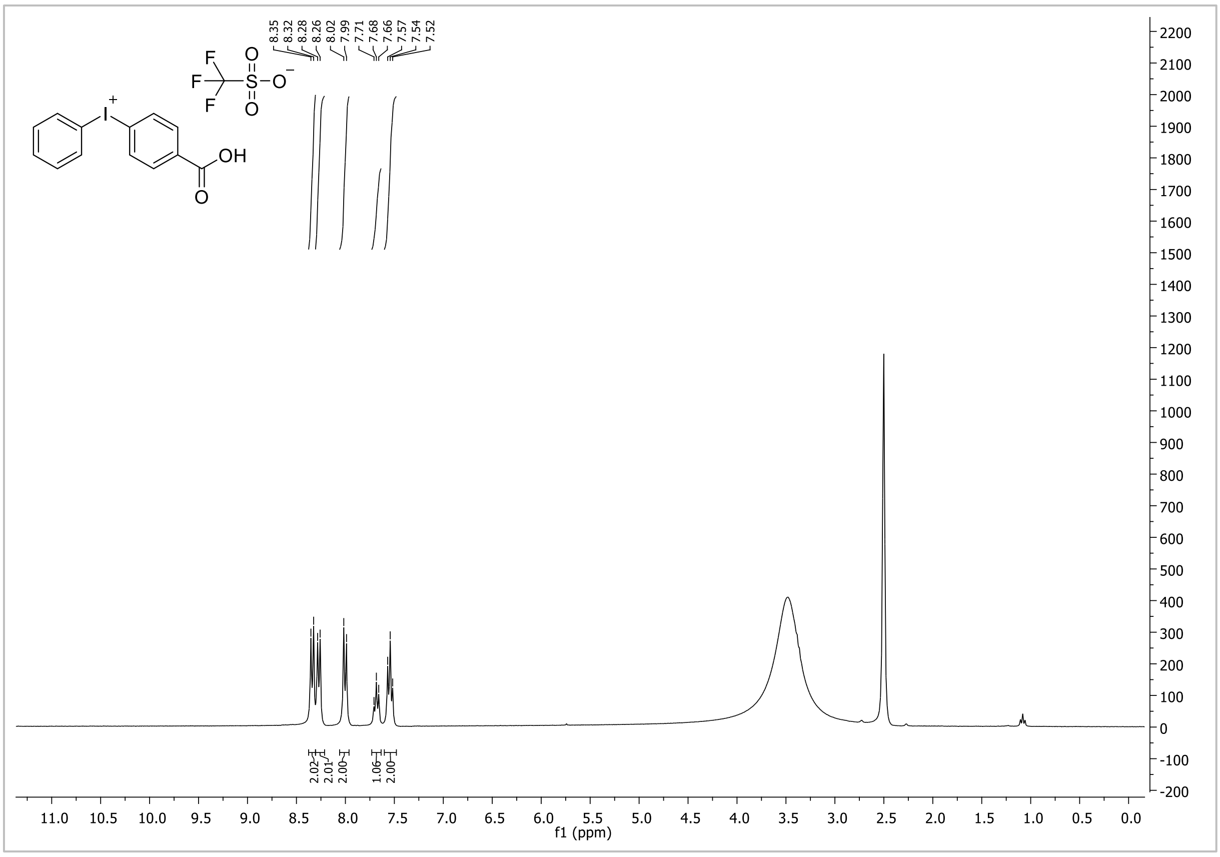

Figure S121. ^{1}H-NMR (300.36 MHz, DMSO-d_6) of compound **1a**.

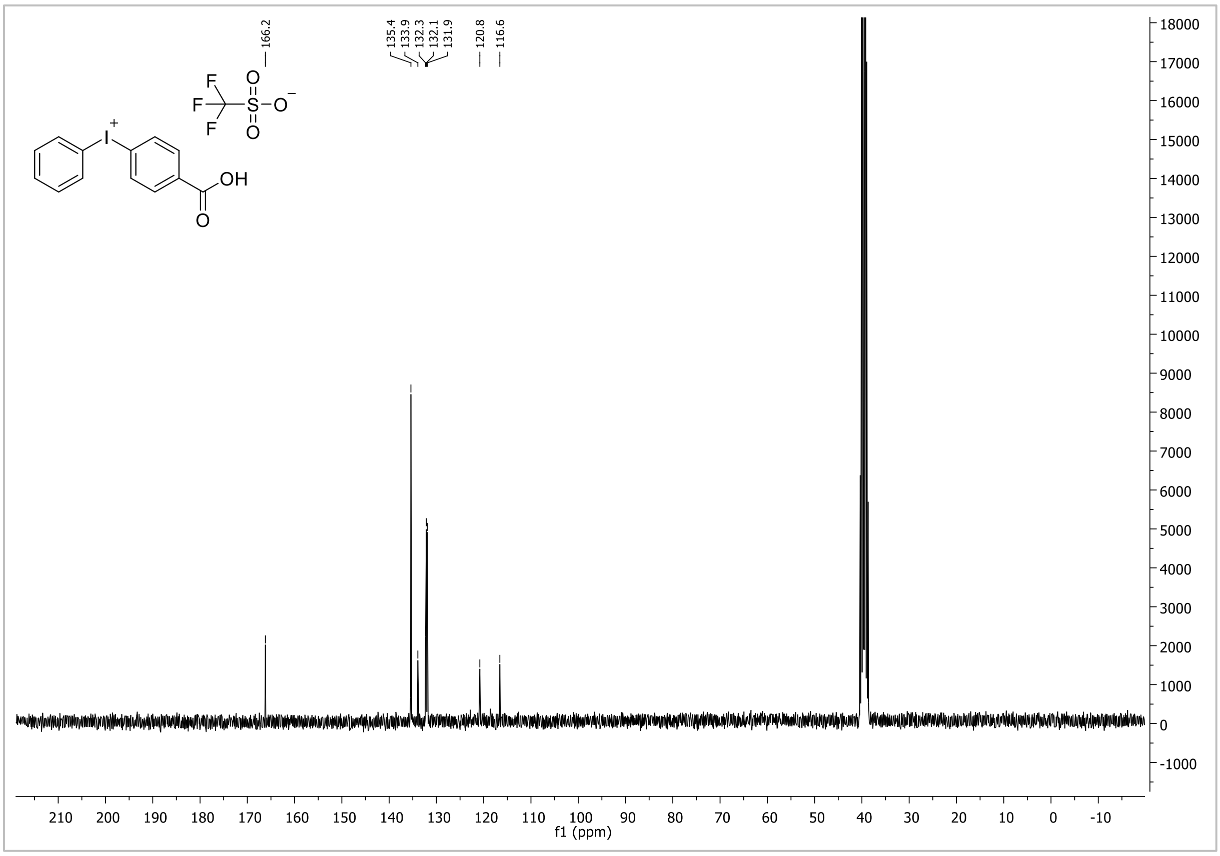

Figure S122. ^{13}C-NMR (75.53 MHz, DMSO-d_6) of compound **1a**.

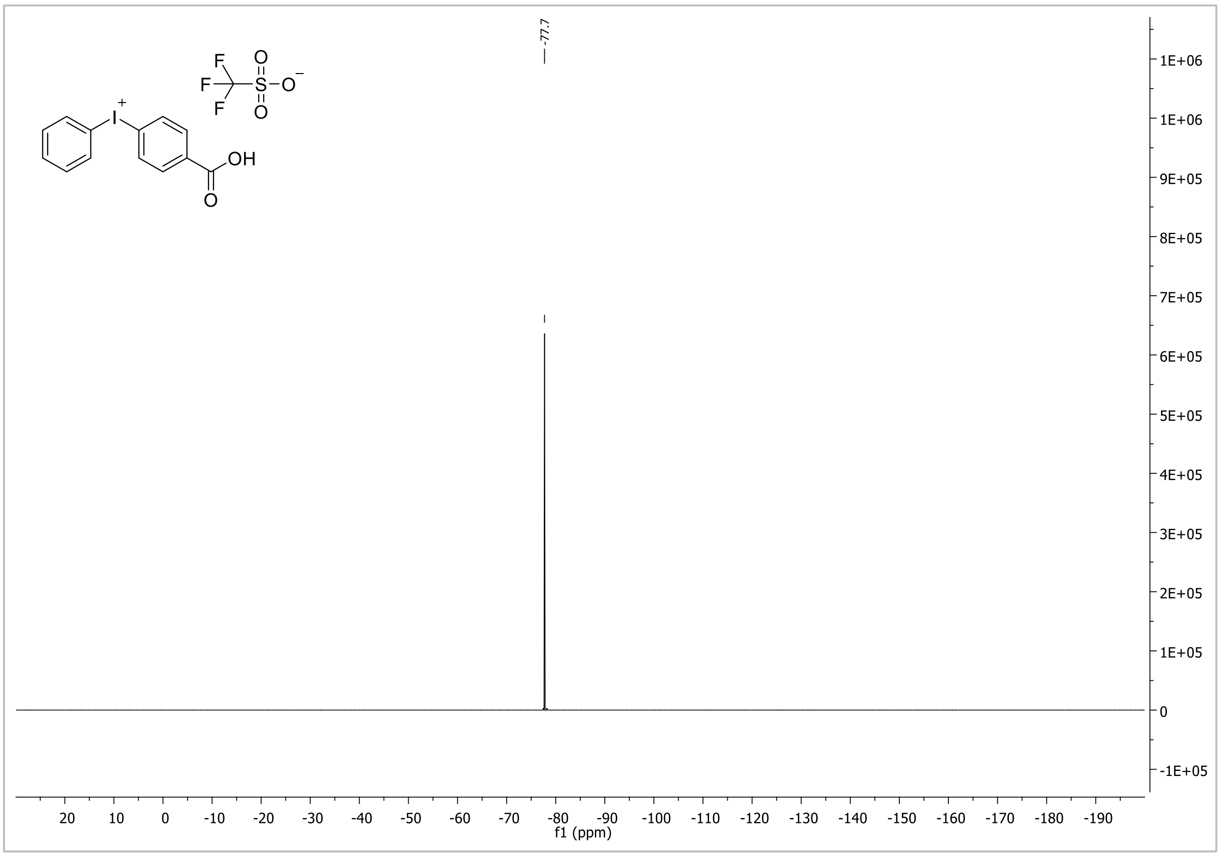

Figure S123. ^{19}F-NMR (470.53 MHz, DMSO-d_6) of compound **1a**.

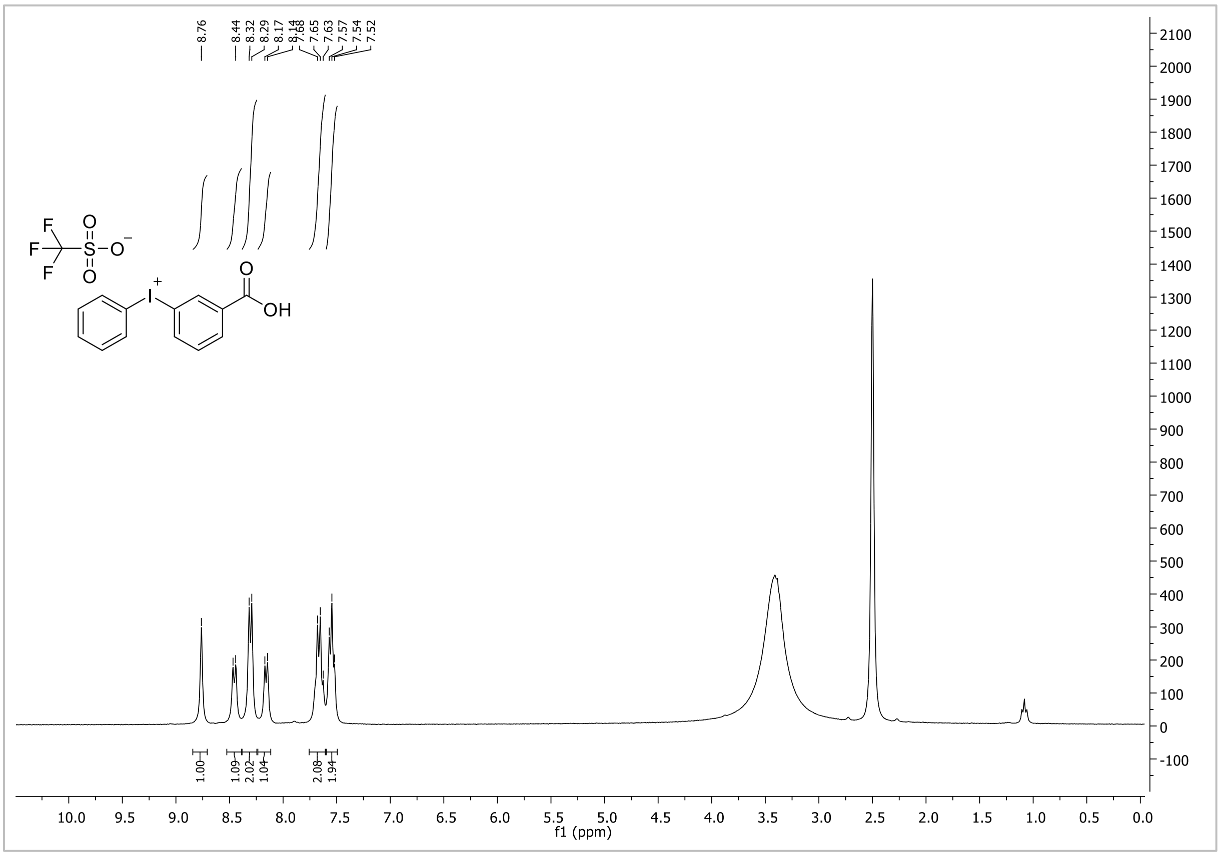

Figure S124. ^{1}H-NMR (300.36 MHz, DMSO-d_6) of compound **1b**.

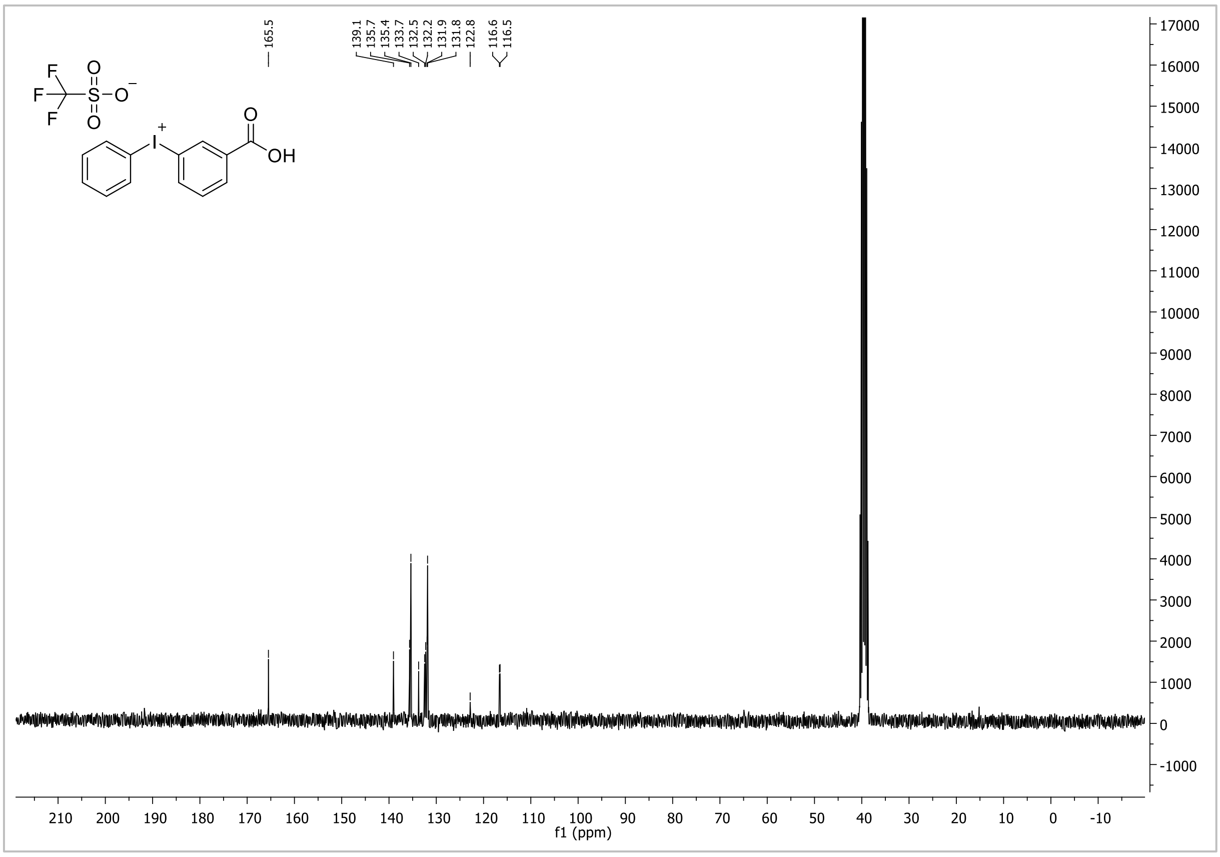

Figure S125. ^{13}C-NMR (75.53 MHz, DMSO-d_6) of compound **1b**.

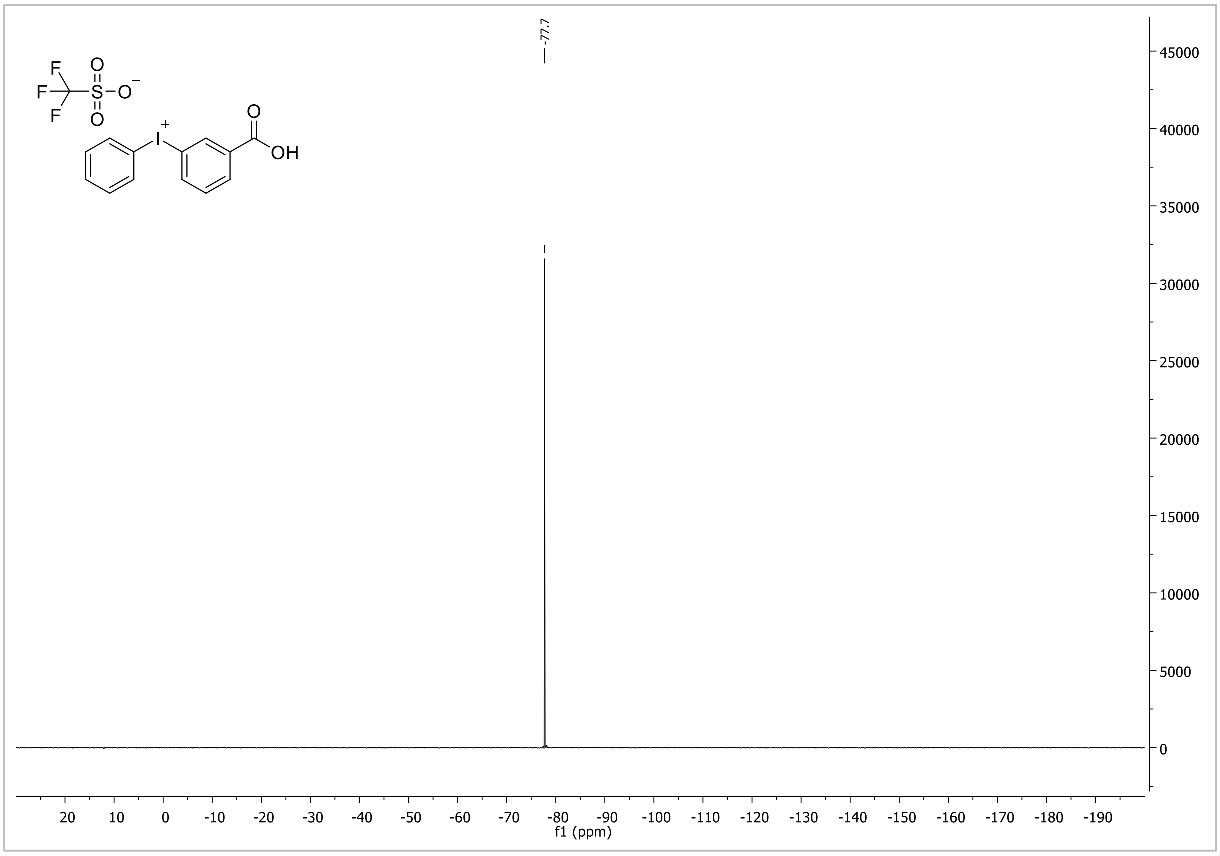

Figure S126. ^{19}F-NMR (470.53 MHz, DMSO-d_6) of compound **1b**.

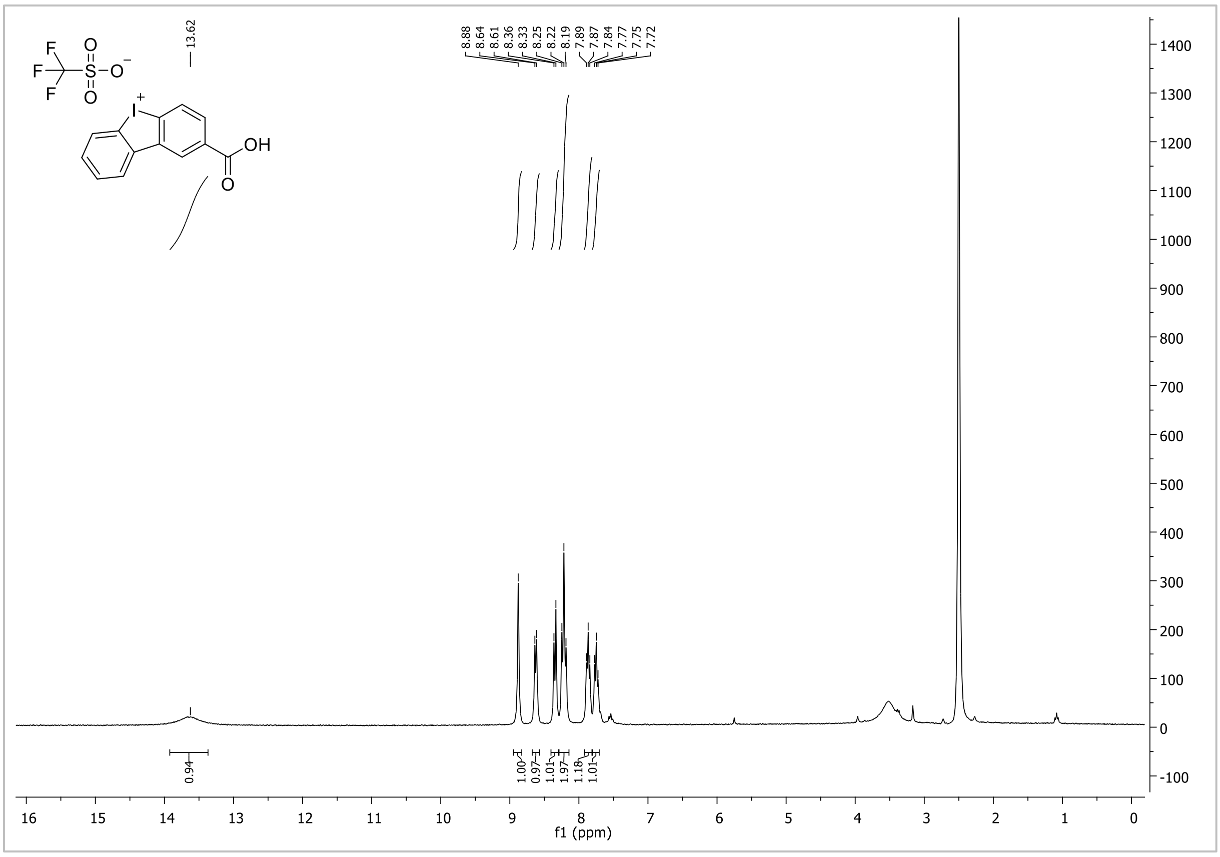

Figure S127. ^{1}H-NMR (300.36 MHz, DMSO-d_6) of compound **1c**.

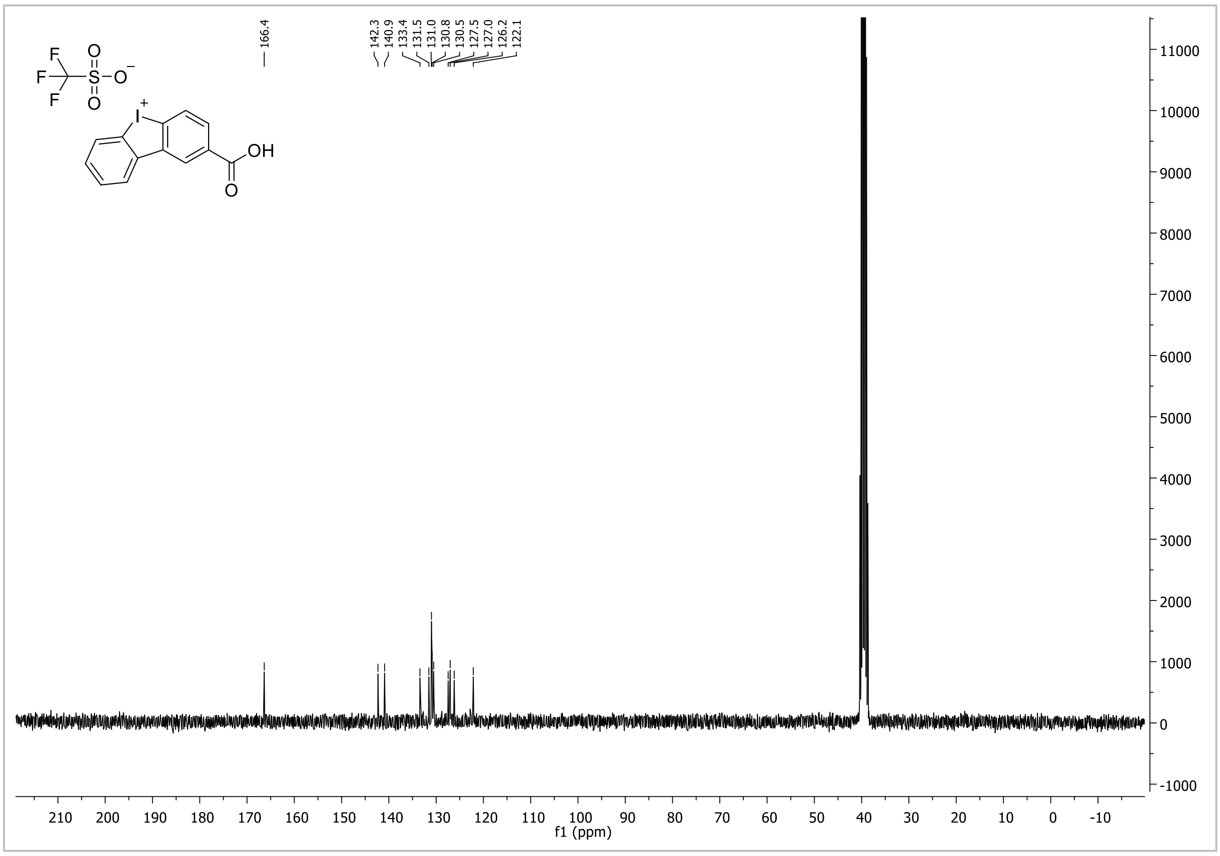

Figure S128. ^{13}C-NMR (75.53 MHz, DMSO-d_6) of compound **1c**.

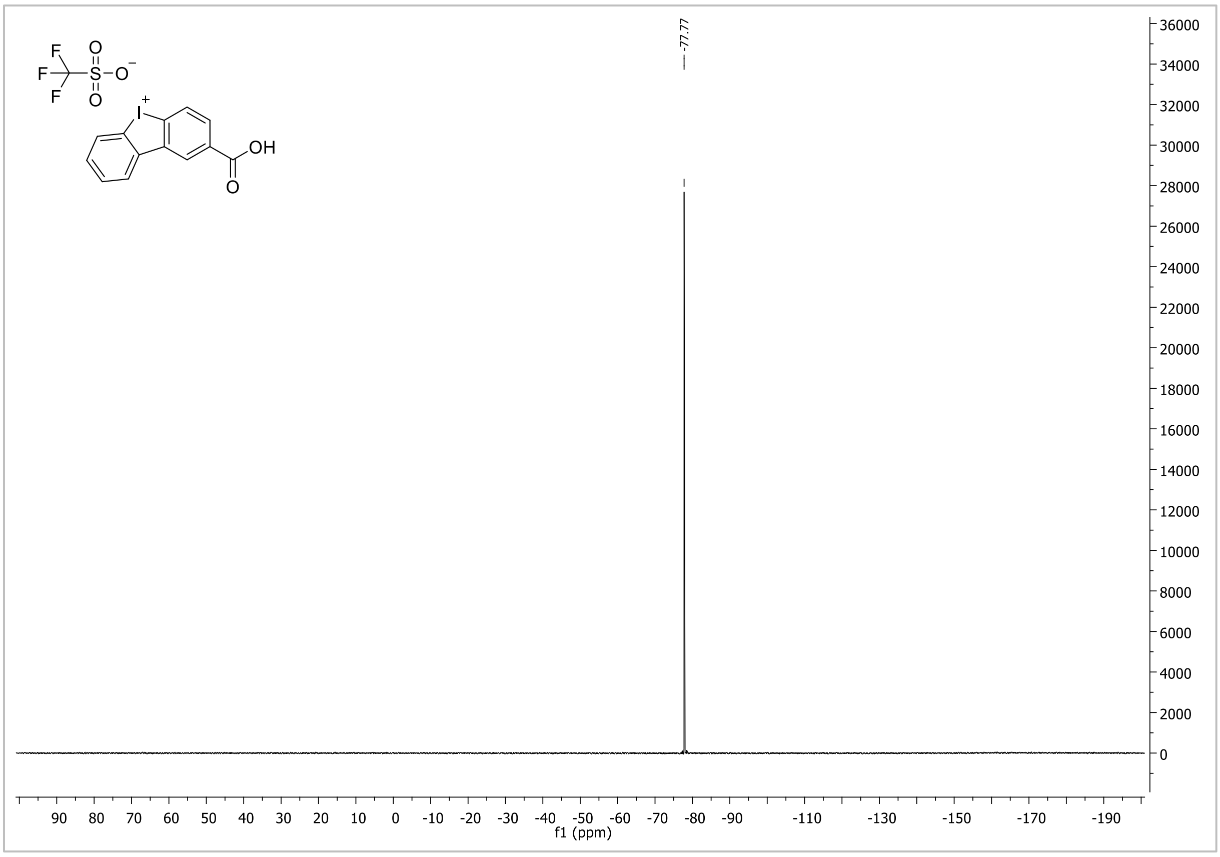

Figure S129. ^{19}F-NMR (282.40 MHz, DMSO-d_6) of compound **1c**.

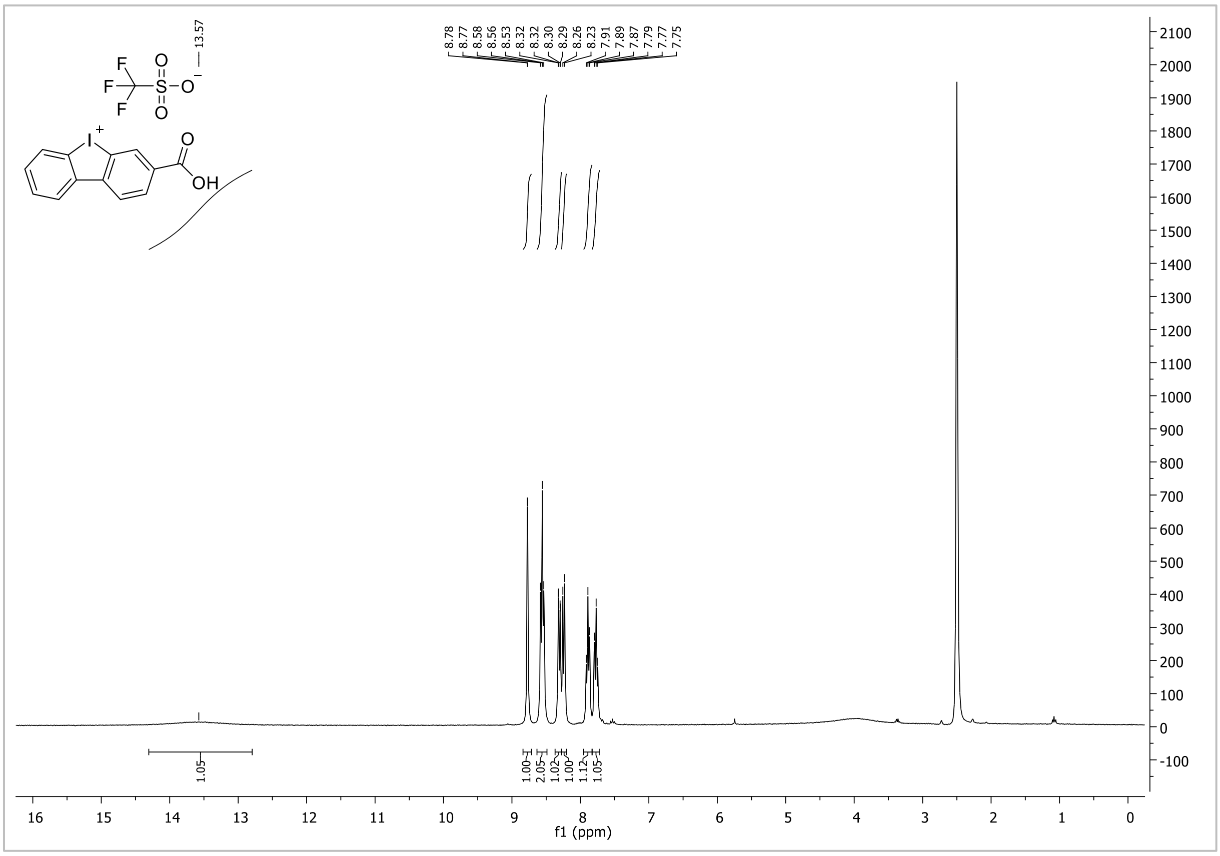

Figure S130. ^{1}H-NMR (300.36 MHz, DMSO-d_6) of compound **1d**.

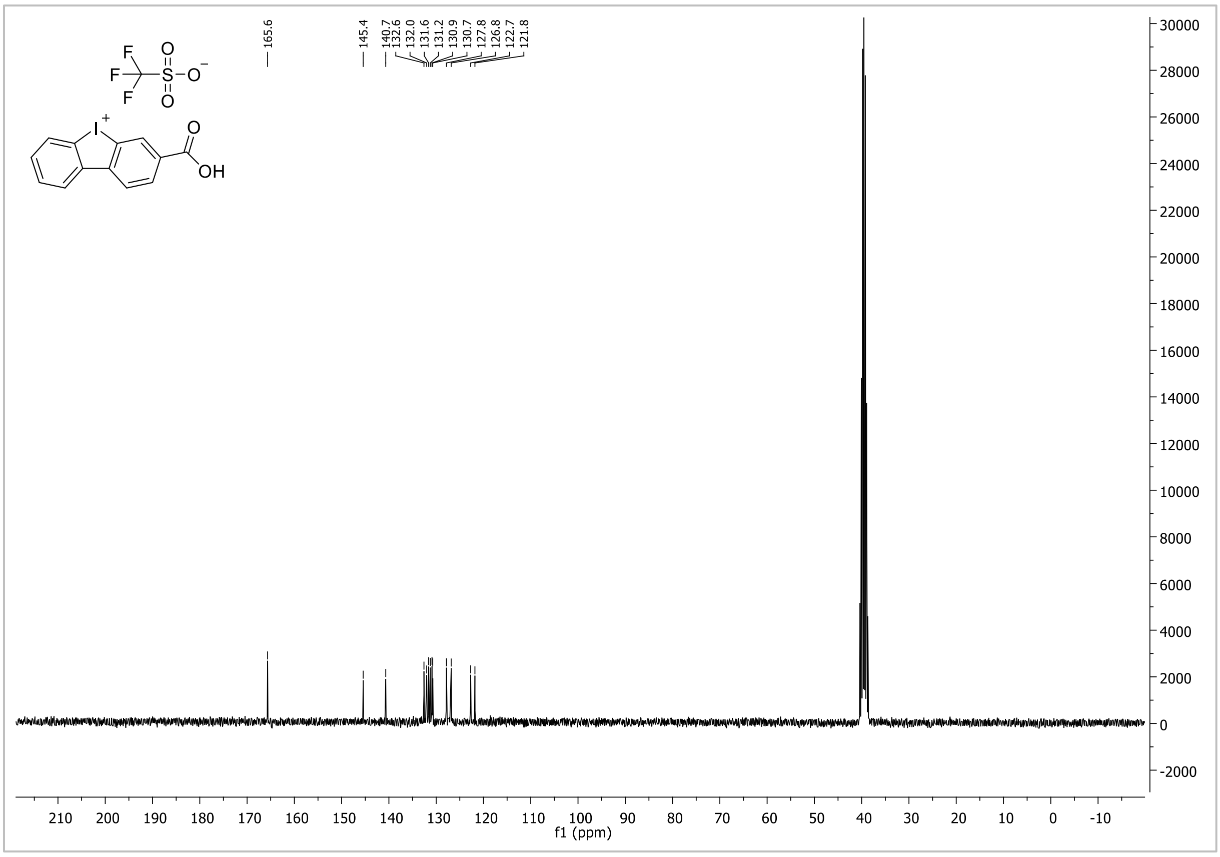

Figure S131. ^{13}C-NMR (75.53 MHz, DMSO-d_6) of compound **1d**.

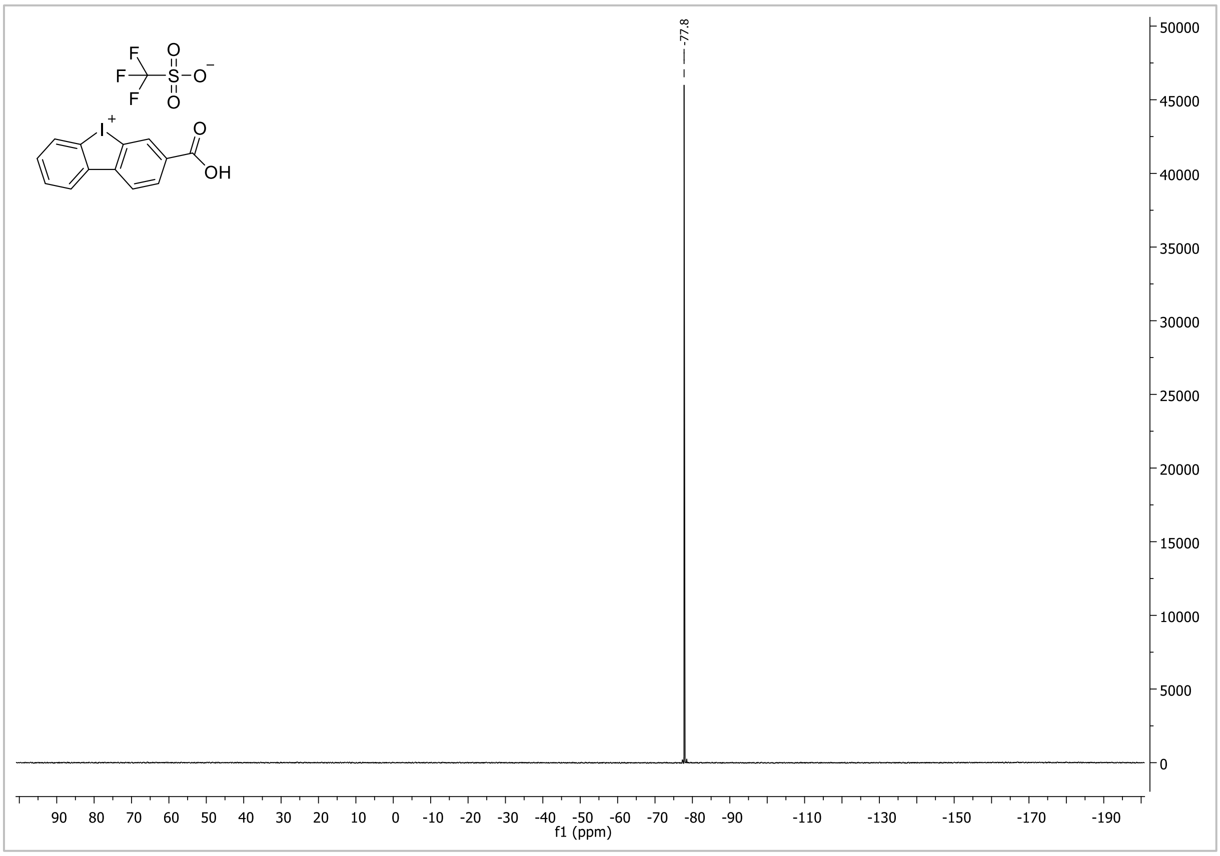

Figure S132. ^{19}F-NMR (282.40 MHz, DMSO-d_6) of compound **1d**.

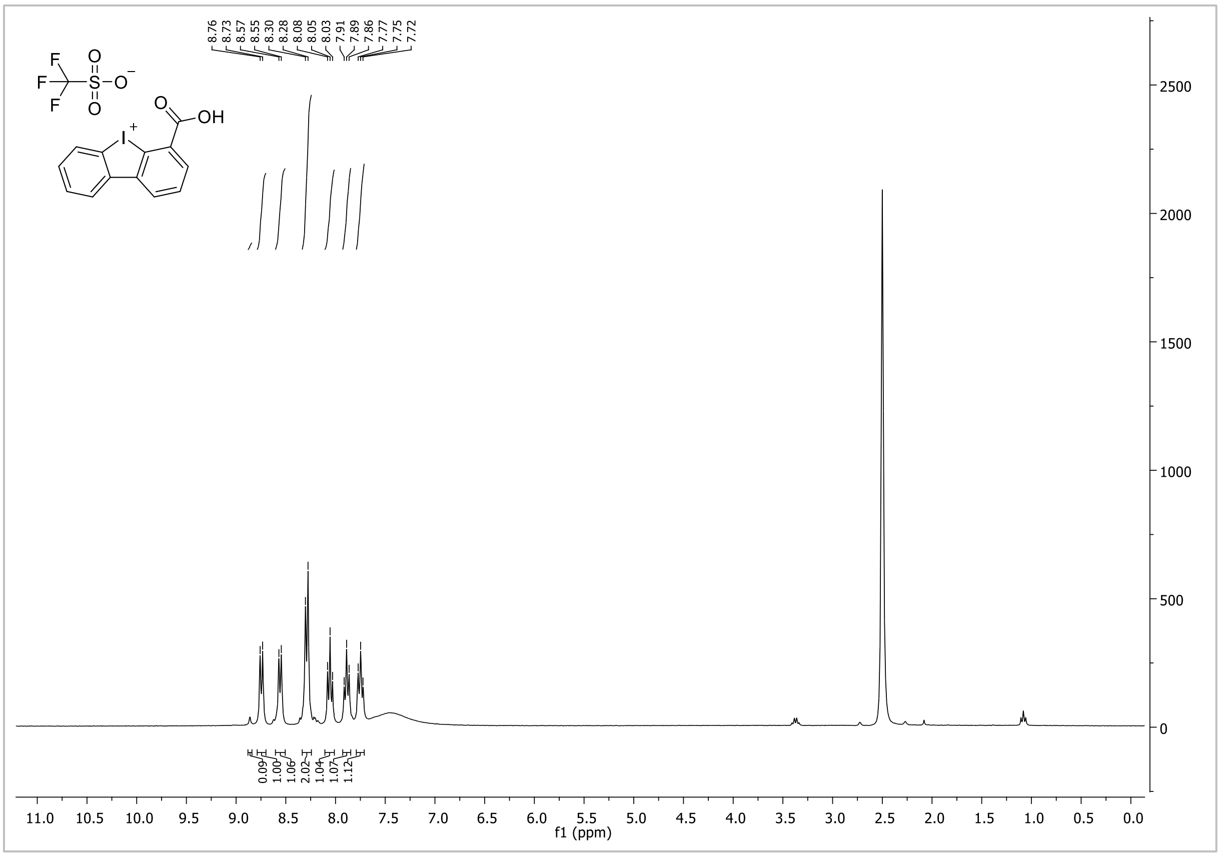

Figure S133. ^{1}H-NMR (300.36 MHz, DMSO-d_6) of compound **1e**.

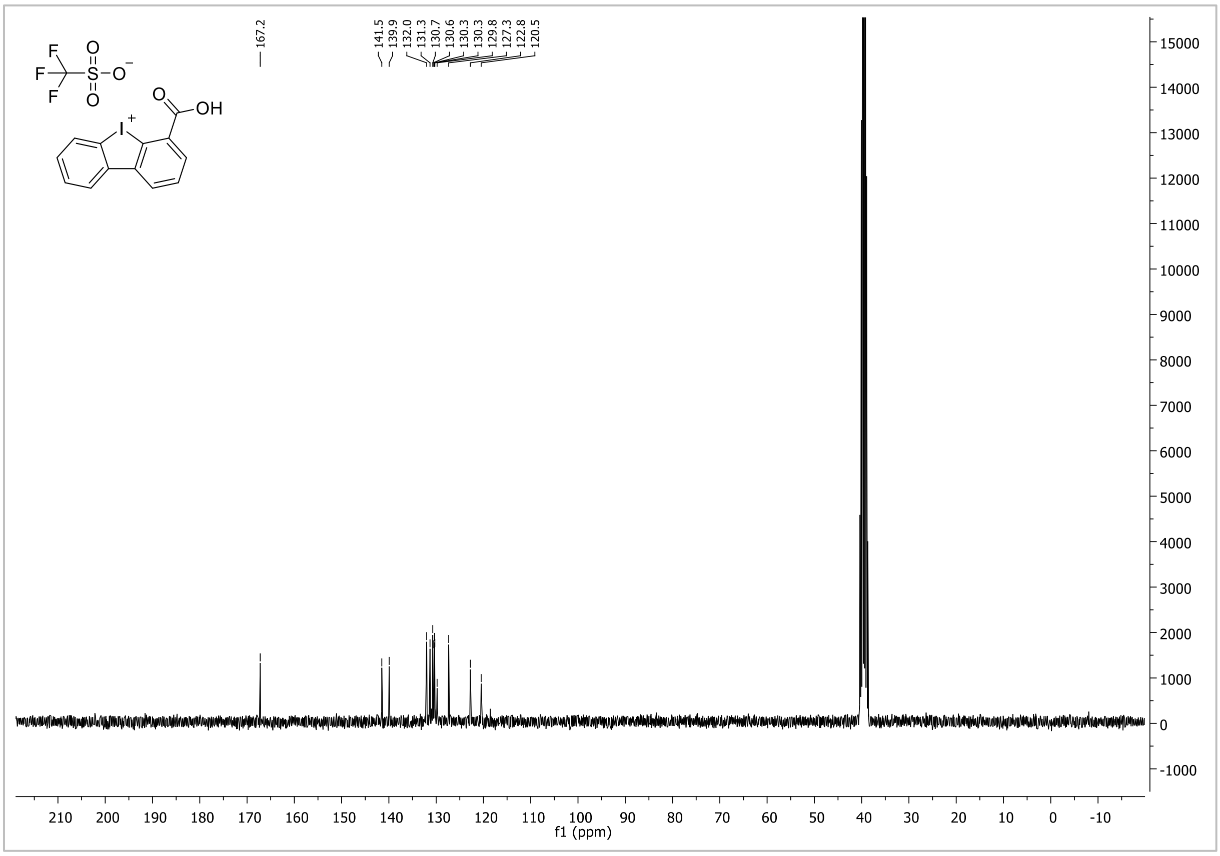

Figure S134. ^{13}C-NMR (75.53 MHz, DMSO-d_6) of compound **1e**.

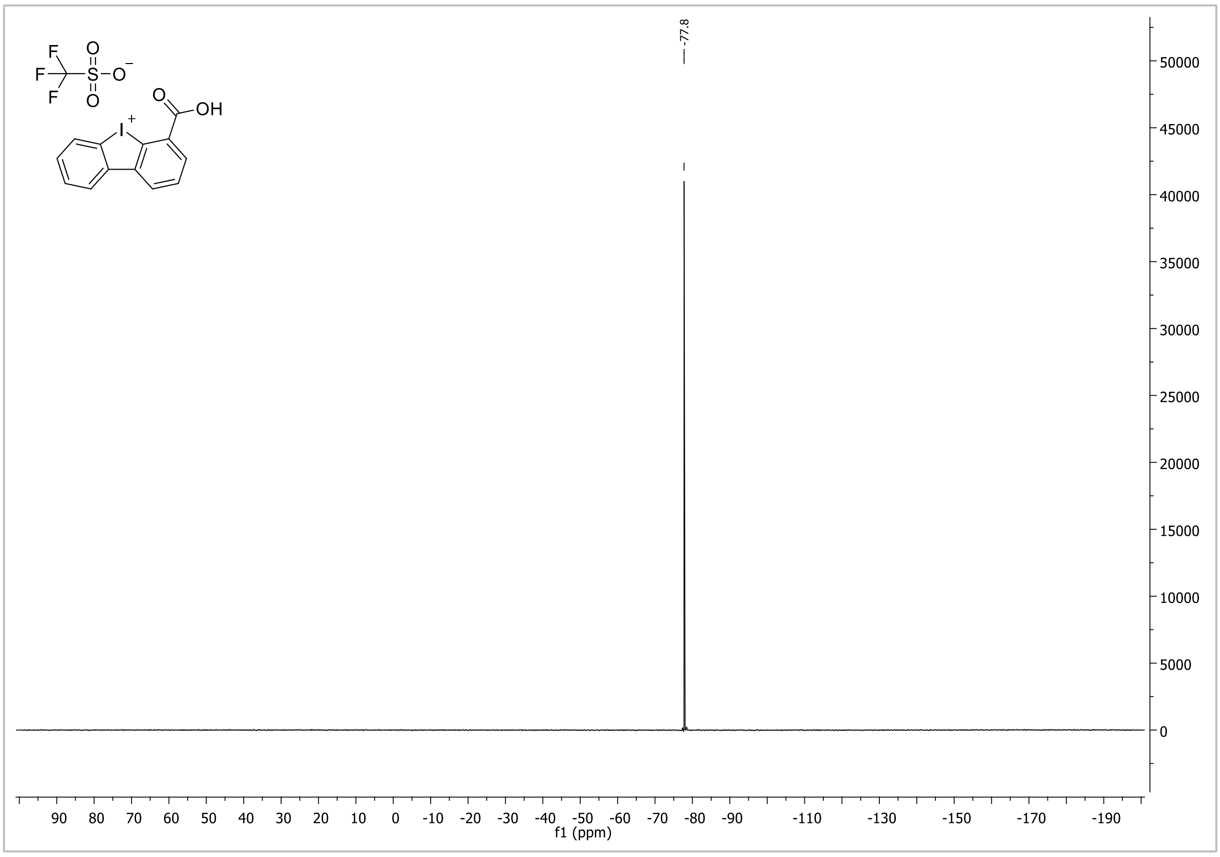

Figure S135. ^{19}F-NMR (470.53 MHz, DMSO-d_6) of compound **1e**.

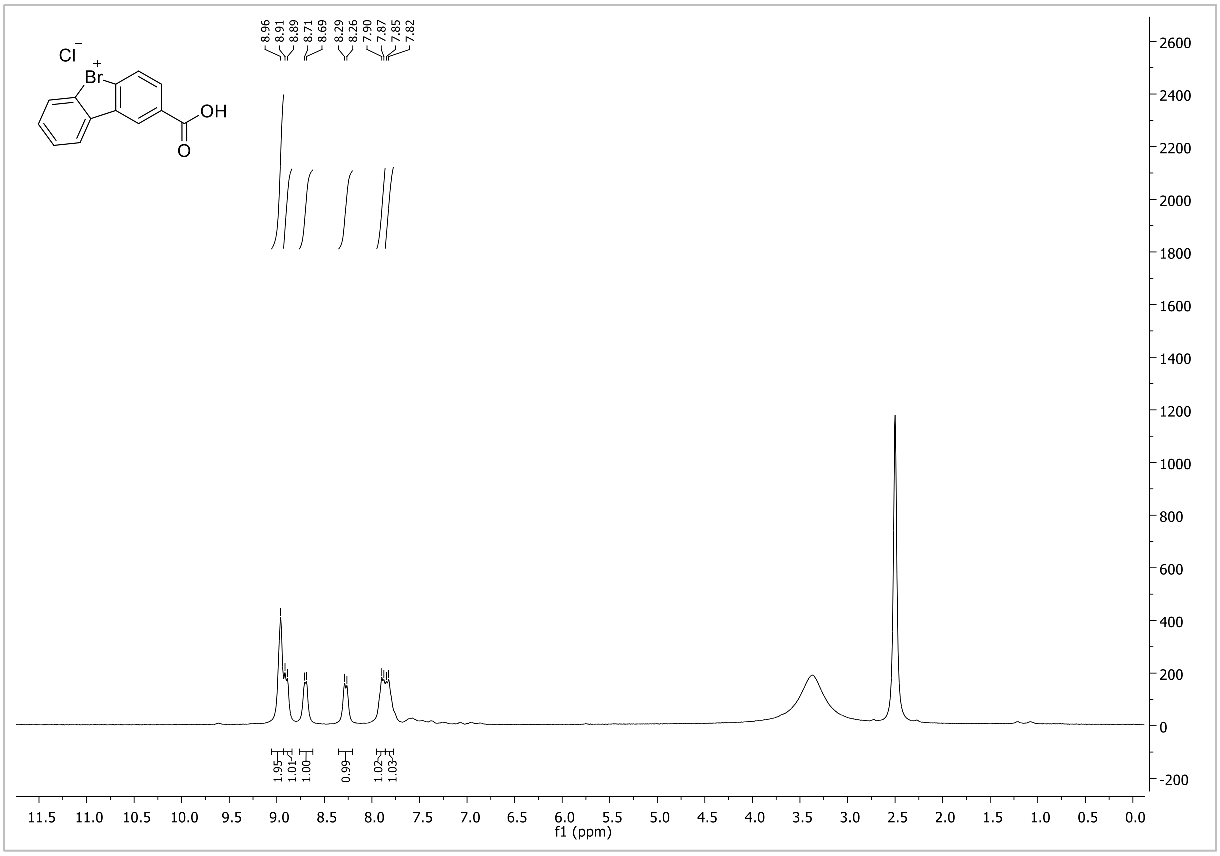

Figure S136. ^{1}H-NMR (300.36 MHz, DMSO-d_6) of compound **11**.

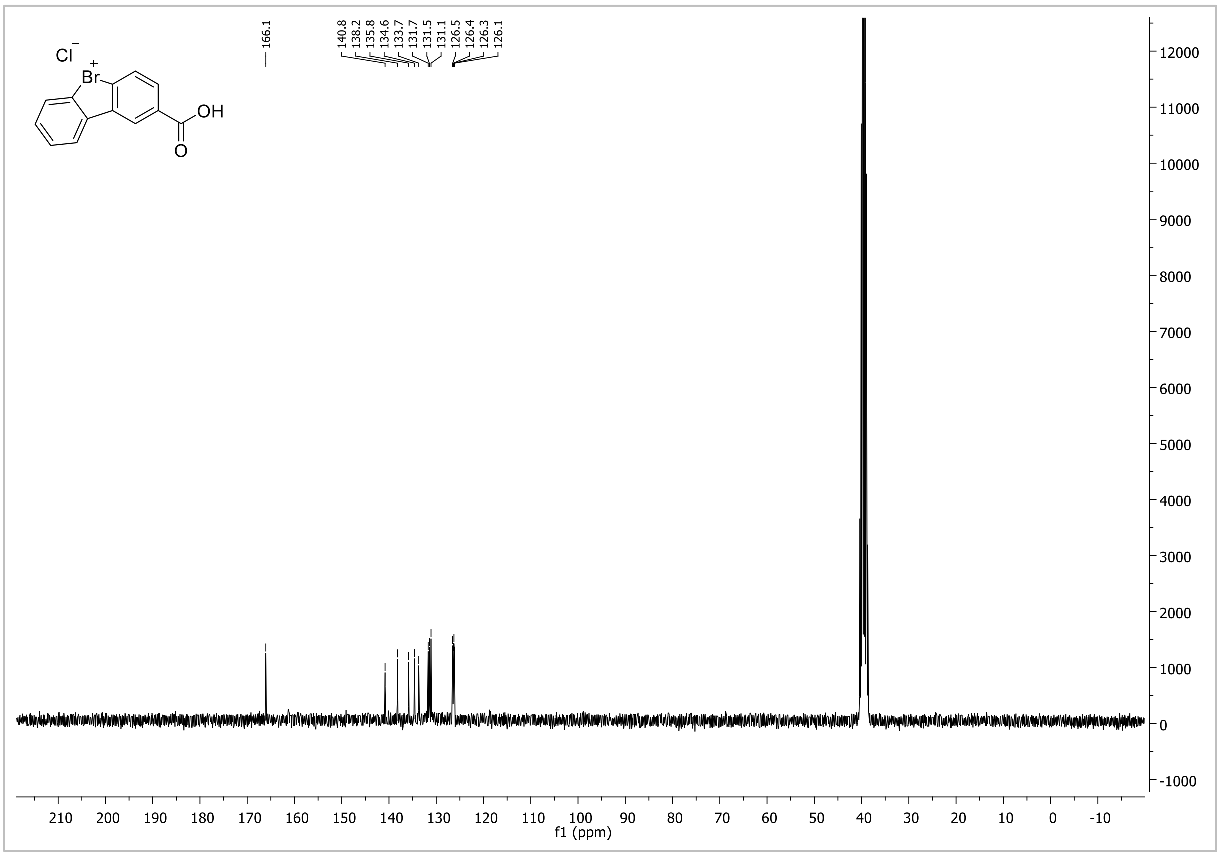

Figure S137. ^{13}C-NMR (75.53 MHz, DMSO-d_6) of compound **11**.

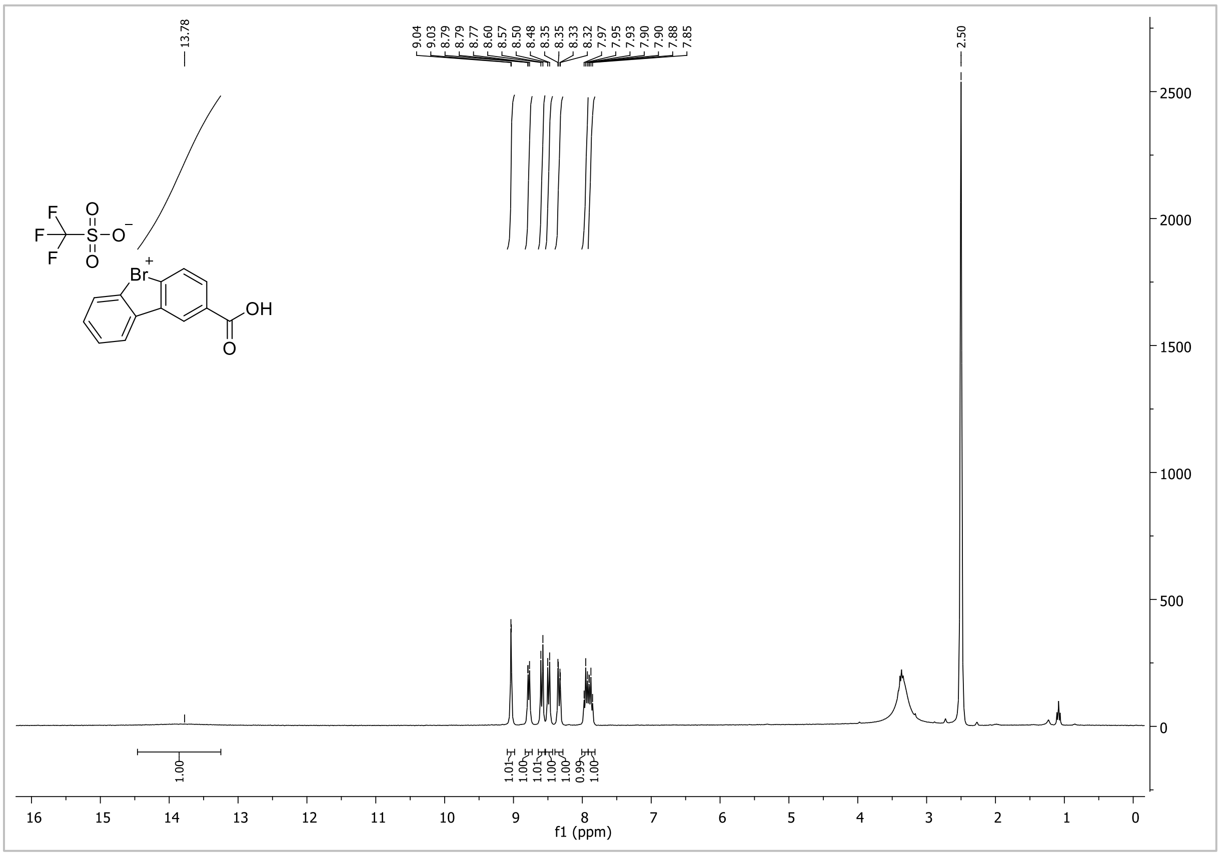

Figure S138. ^{1}H-NMR (300.36 MHz, DMSO-d_6) of compound **1f**.

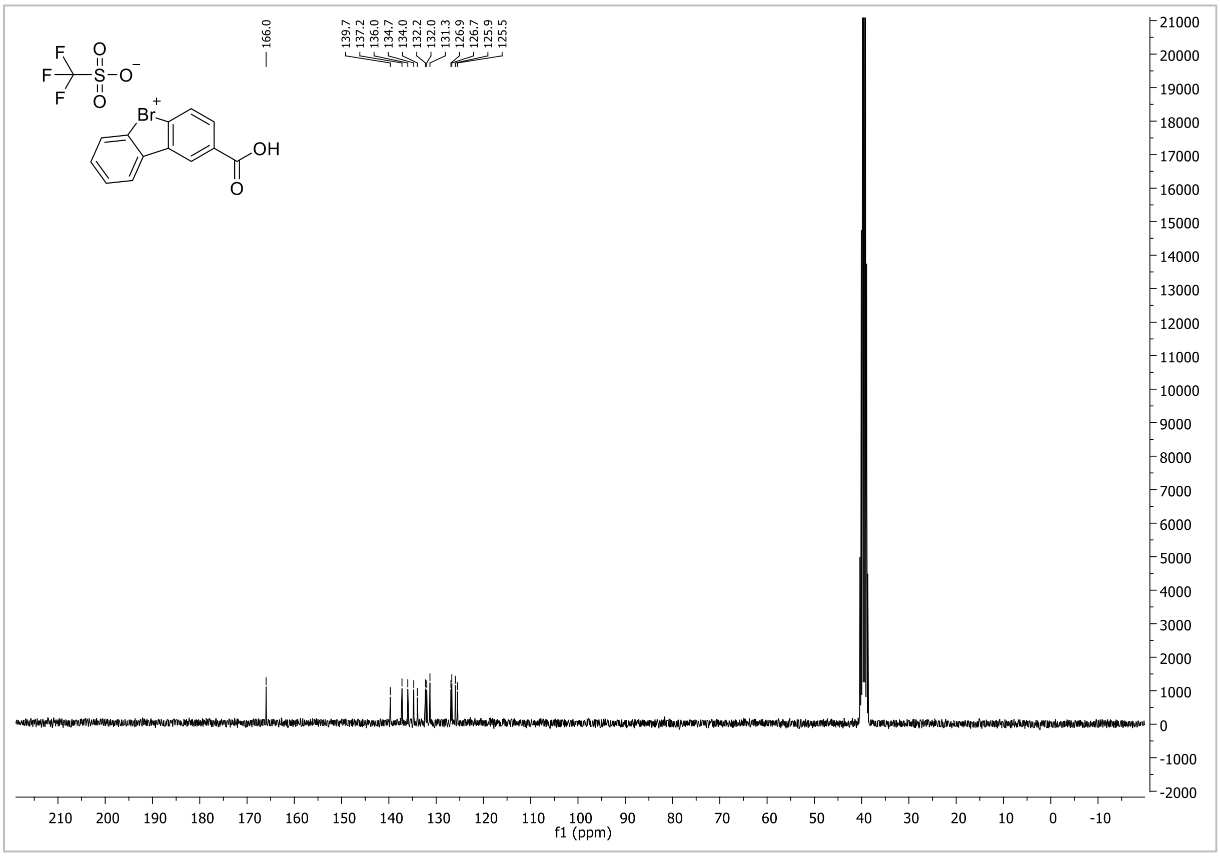

Figure S139. ^{13}C-NMR (75.53 MHz, DMSO-d_6) of compound **1f**.

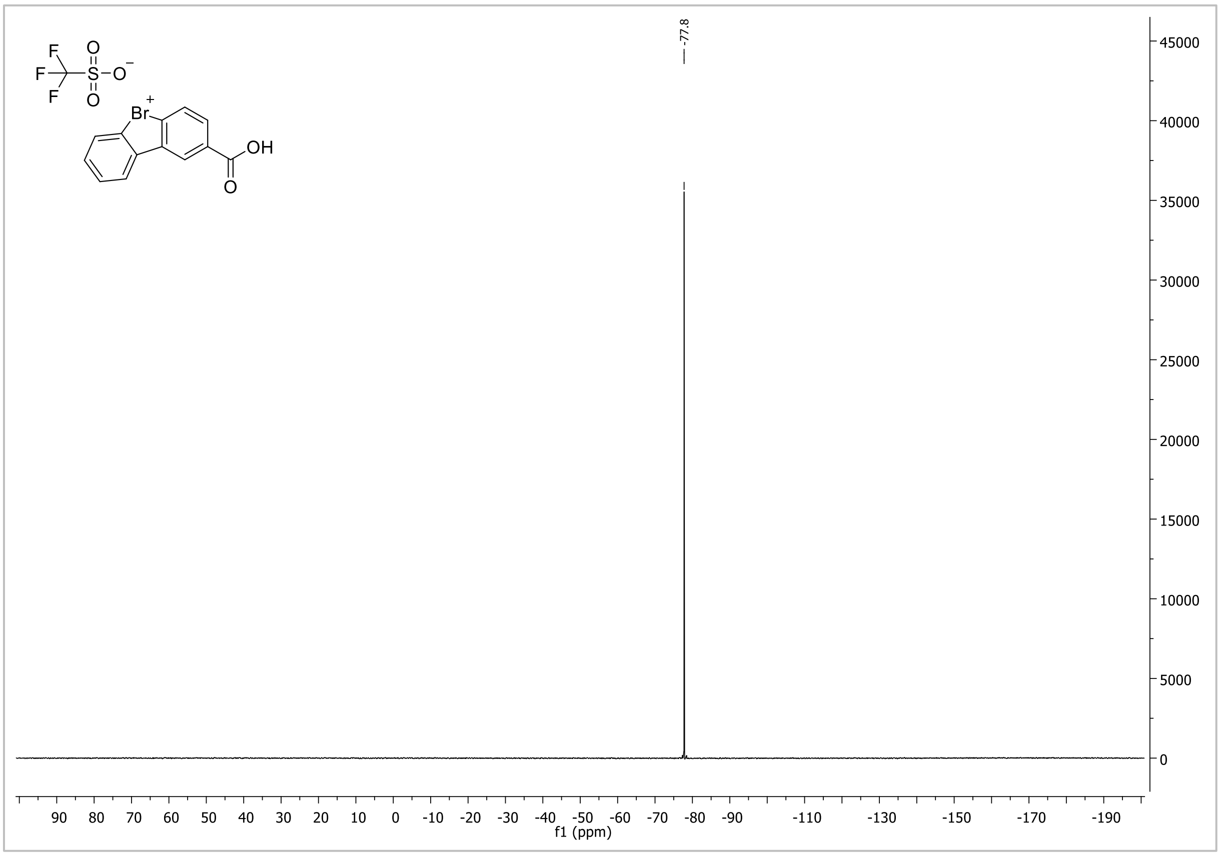

Figure S140. ^{19}F-NMR (282.40 MHz, DMSO-d_6) of compound **1f**.

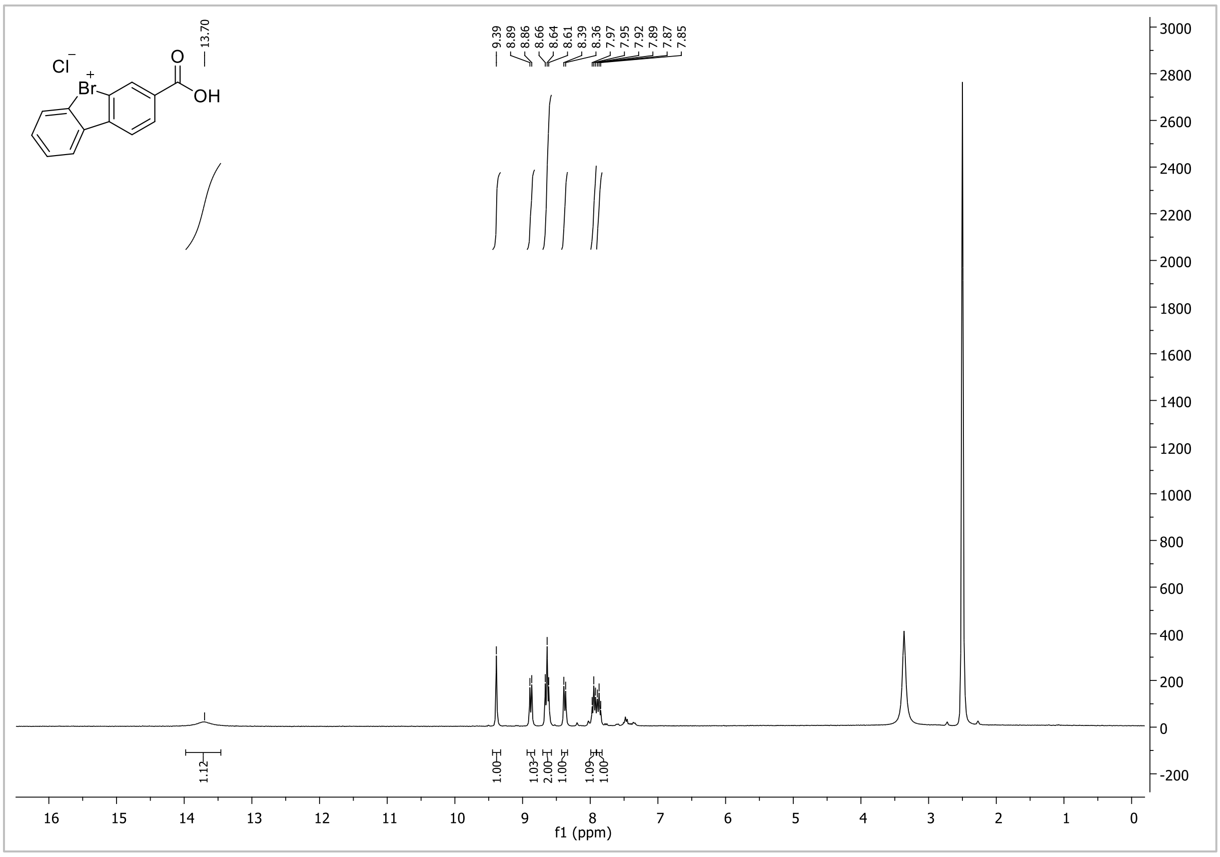

Figure S141. ^{1}H-NMR (300.36 MHz, DMSO-d_6) of compound **12**.

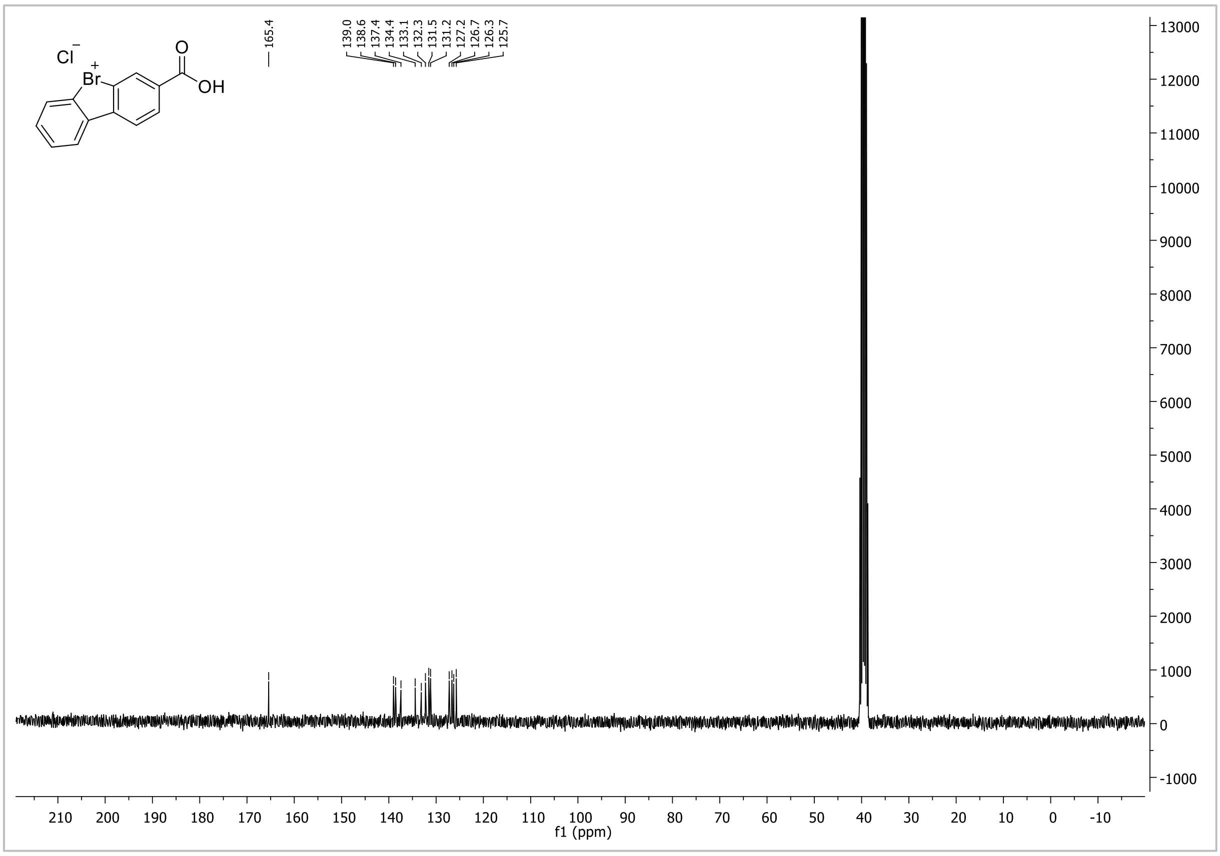

Figure S142. ^{13}C-NMR (75.53 MHz, DMSO-d_6) of compound **12**.

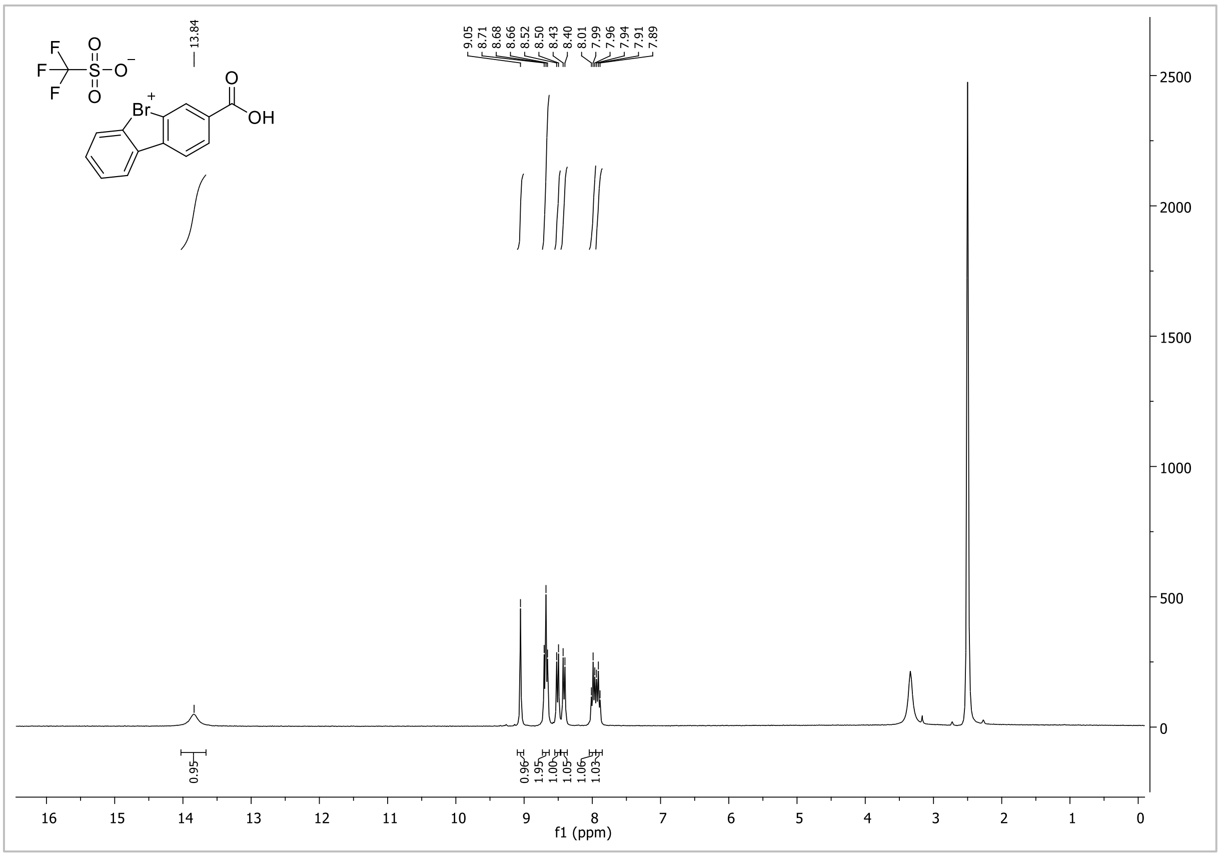

Figure S143. ^{1}H-NMR (300.36 MHz, DMSO-d_6) of compound **1g**.

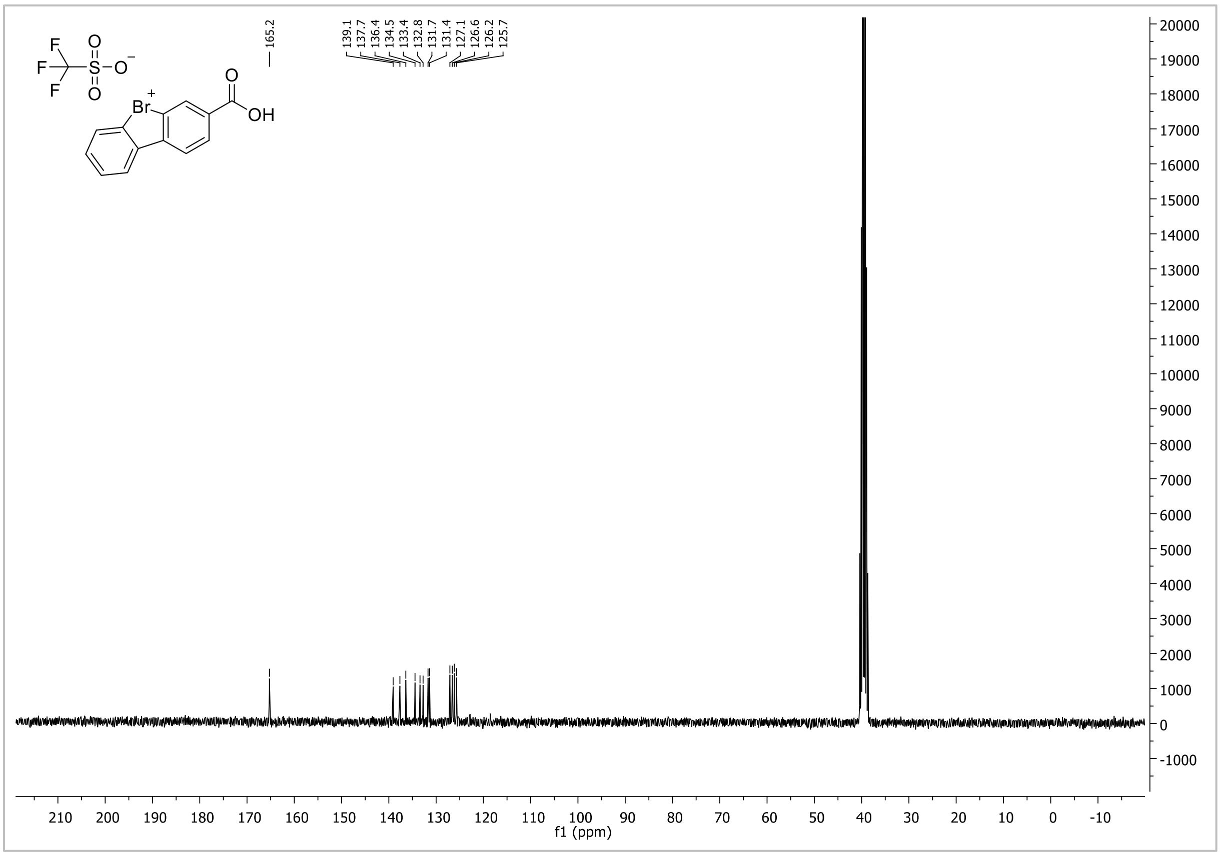

Figure S144. ^{13}C-NMR (75.53 MHz, DMSO-d_6) of compound **1g**.

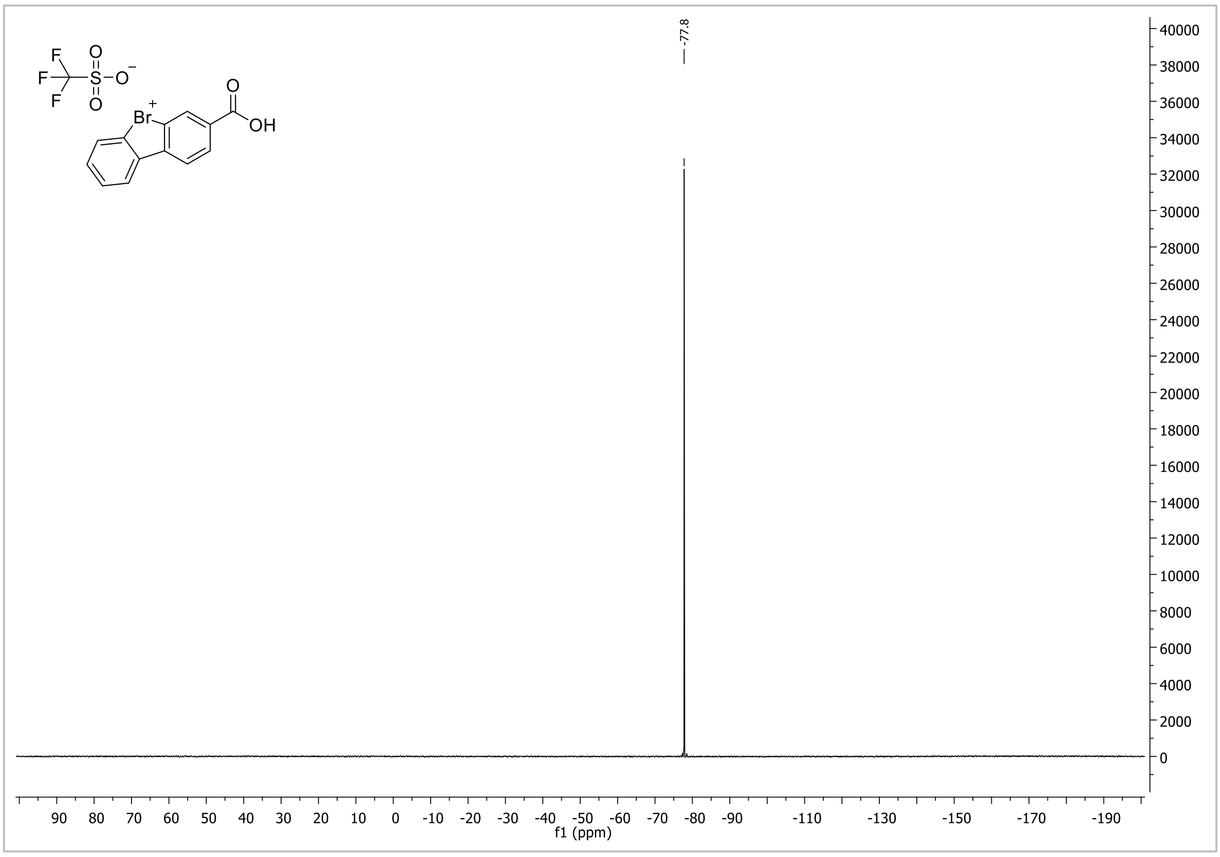

Figure S145. ^{19}F-NMR (282.40 MHz, DMSO-d_6) of compound **1g**.

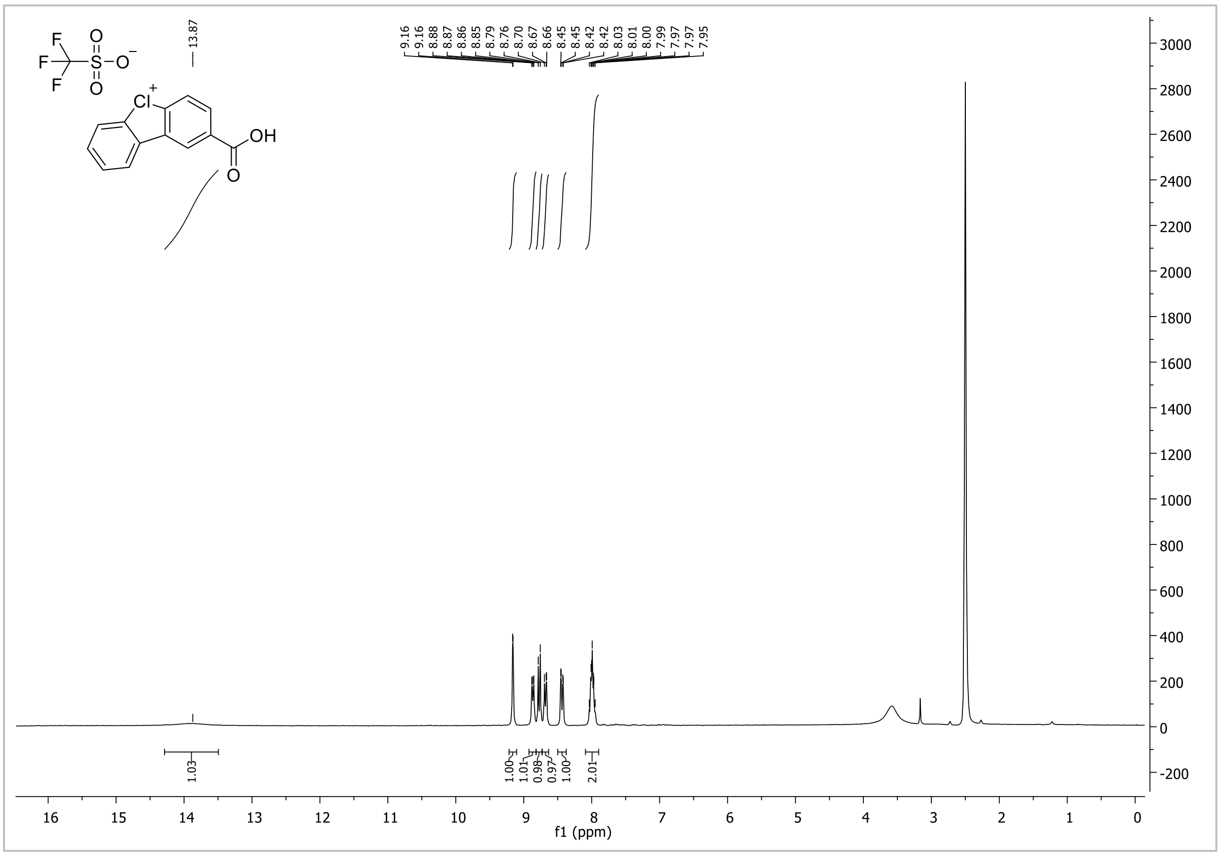

Figure S146. ^{1}H-NMR (300.36 MHz, DMSO-d_6) of compound **1h**.

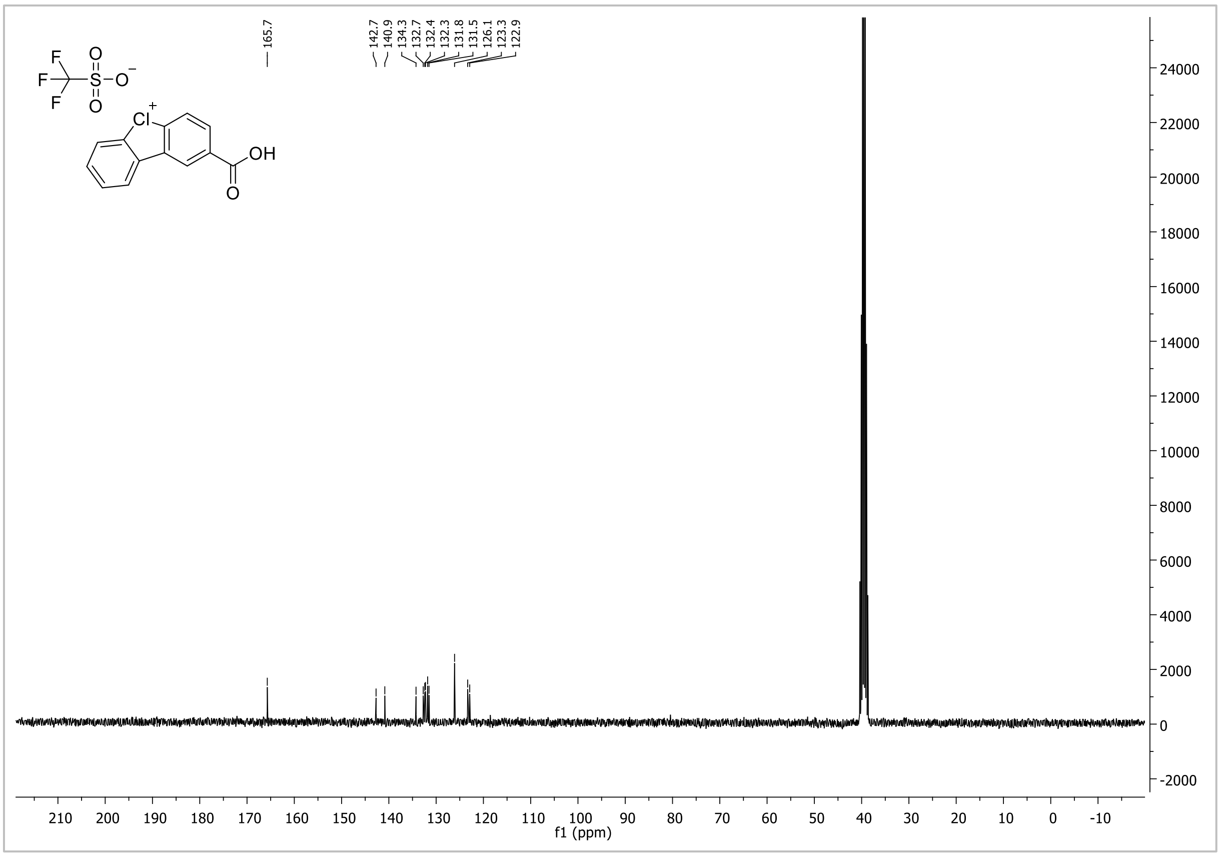

Figure S147. ^{13}C-NMR (75.53 MHz, DMSO-d_6) of compound **1h**.

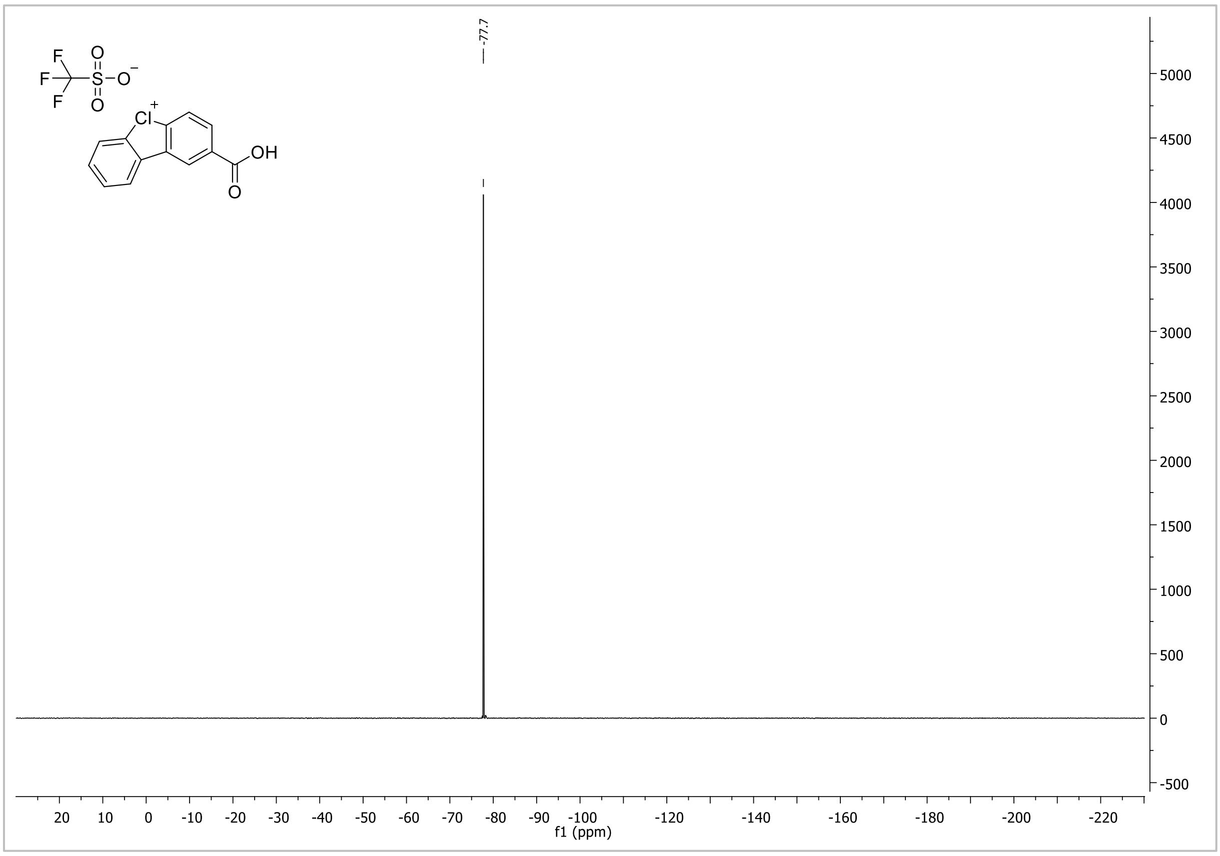

Figure S148. ^{19}F-NMR (470.53 MHz, DMSO-d_6) of compound **1h**.

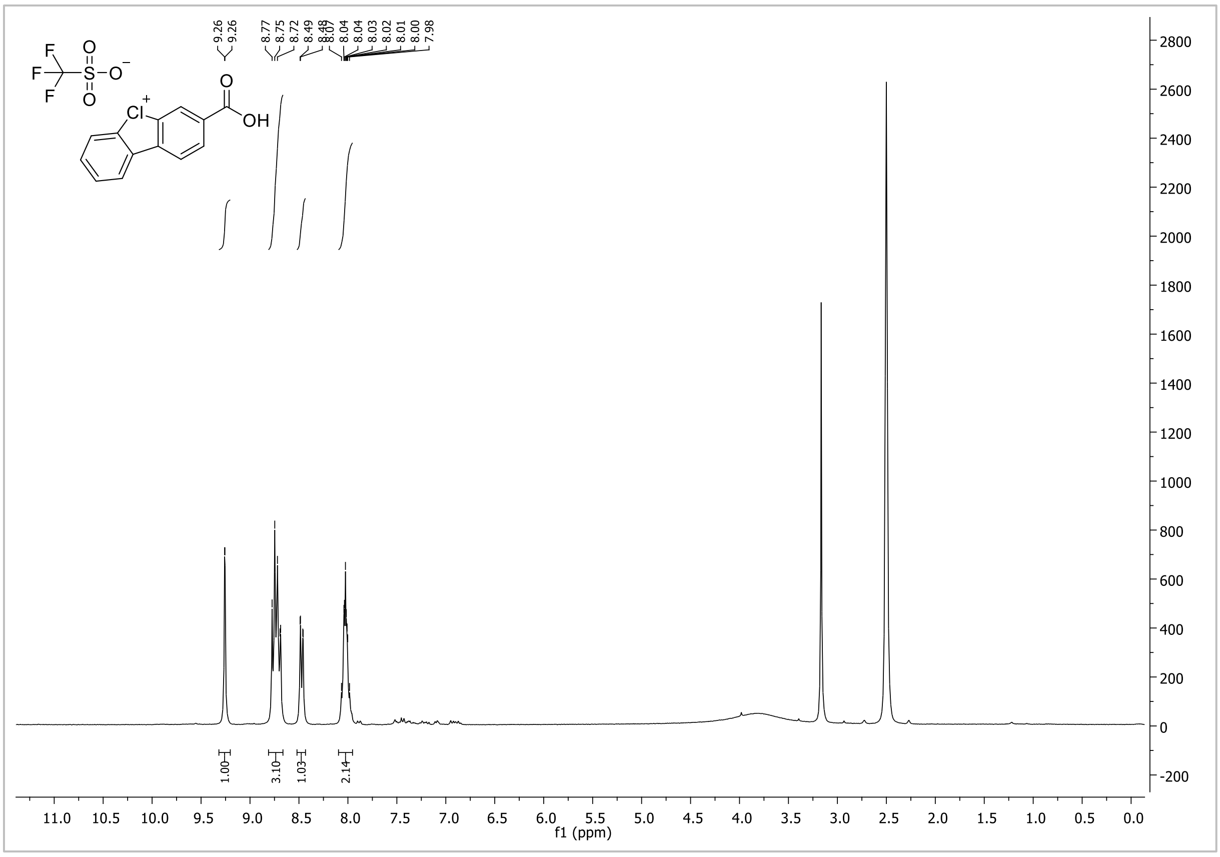

Figure S149. ^{1}H-NMR (300.36 MHz, DMSO-d_6) of compound **1i**.

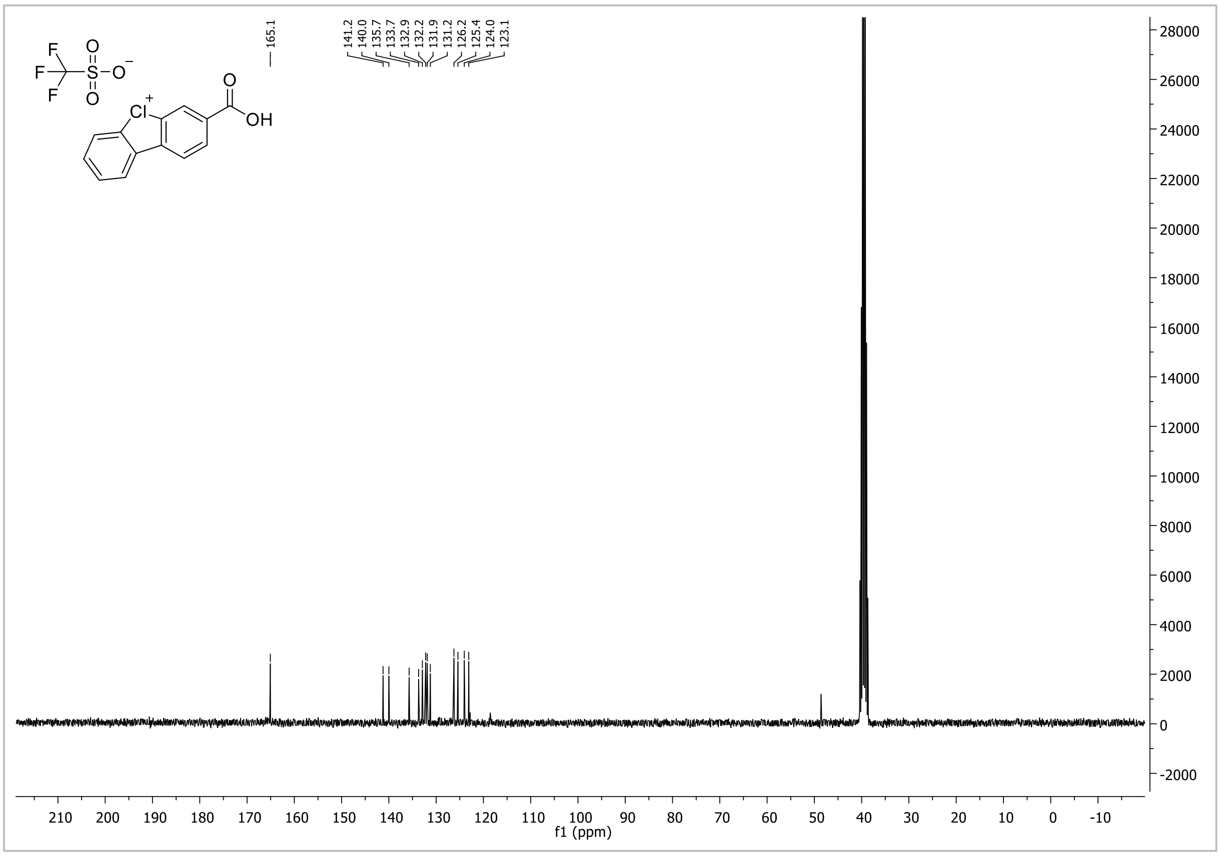

Figure S150. ^{13}C-NMR (75.53 MHz, DMSO-d_6) of compound **1i**.

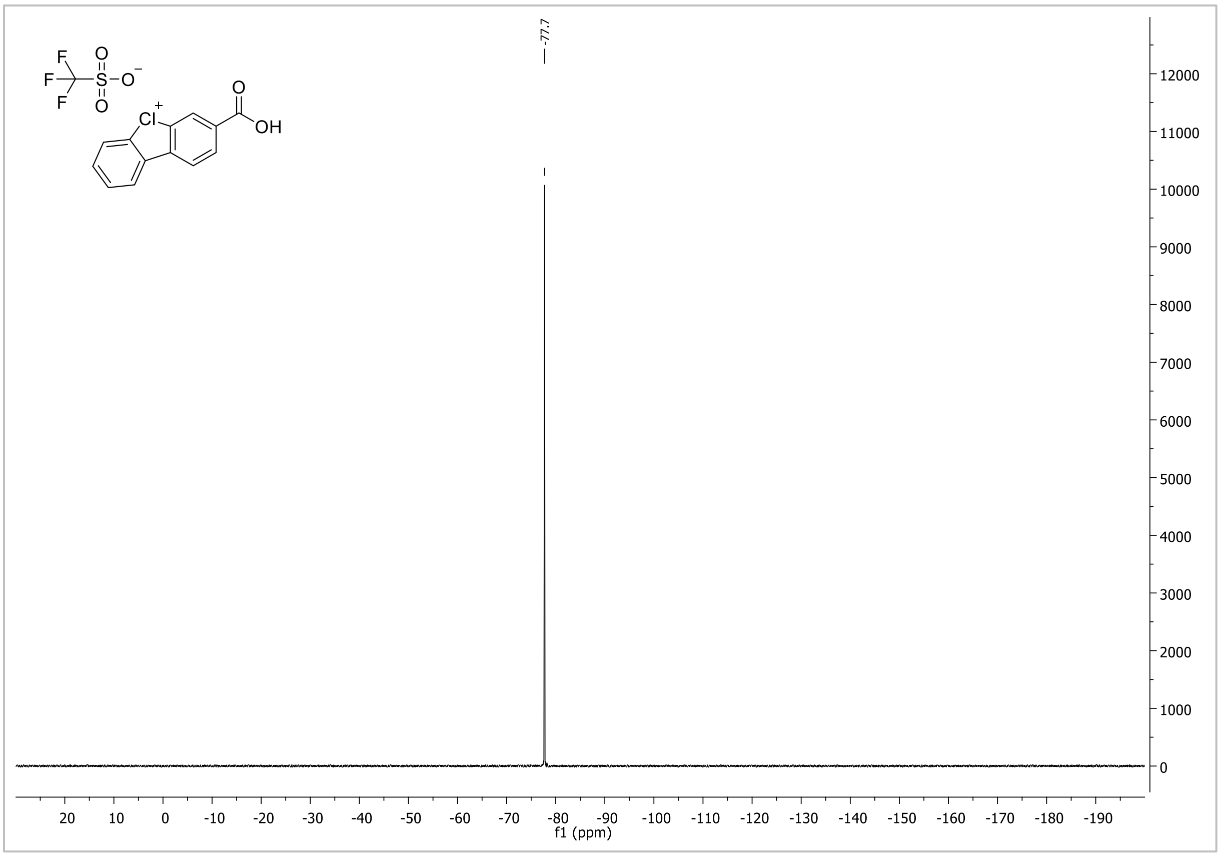

Figure S151. ^{19}F-NMR (470.53 MHz, DMSO-d_6) of compound **1i**.

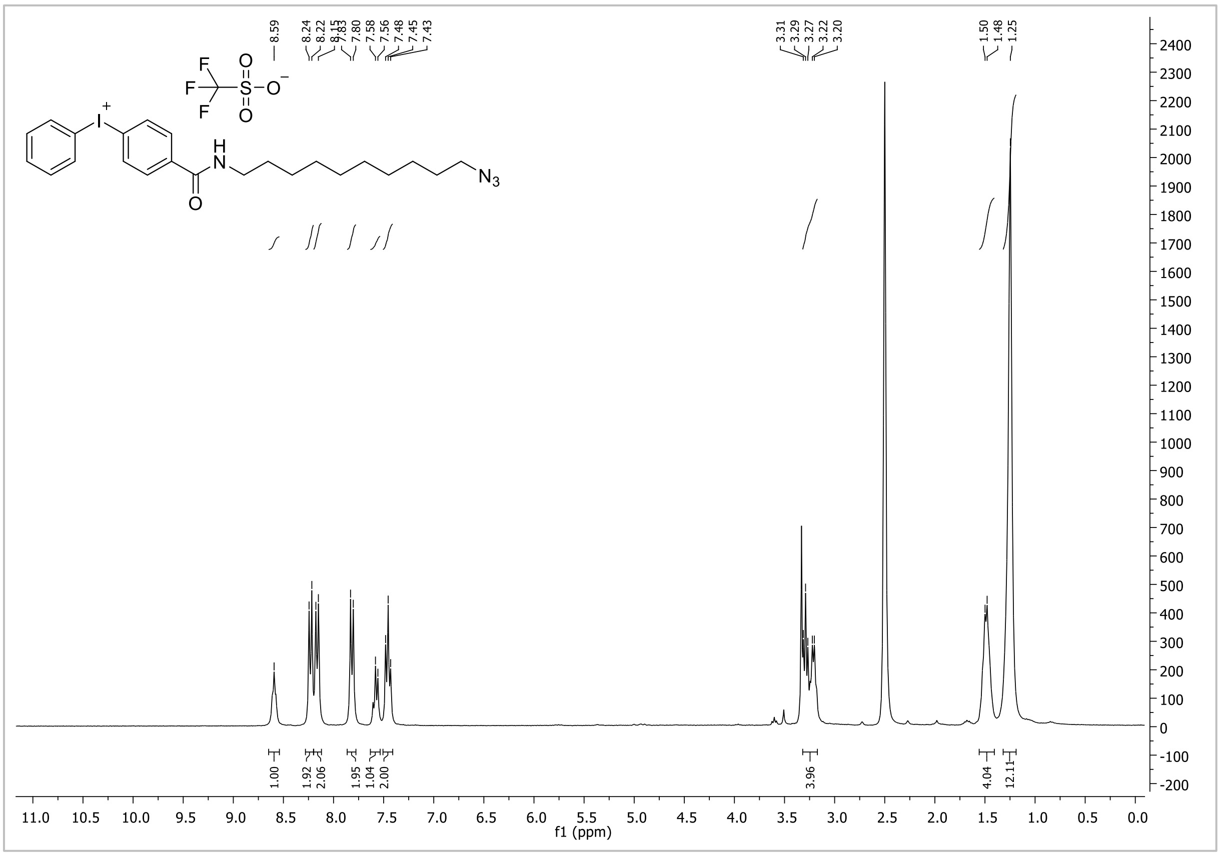

Figure S152. ^{1}H-NMR (300.36 MHz, DMSO-d_6) of compound **3aa**.

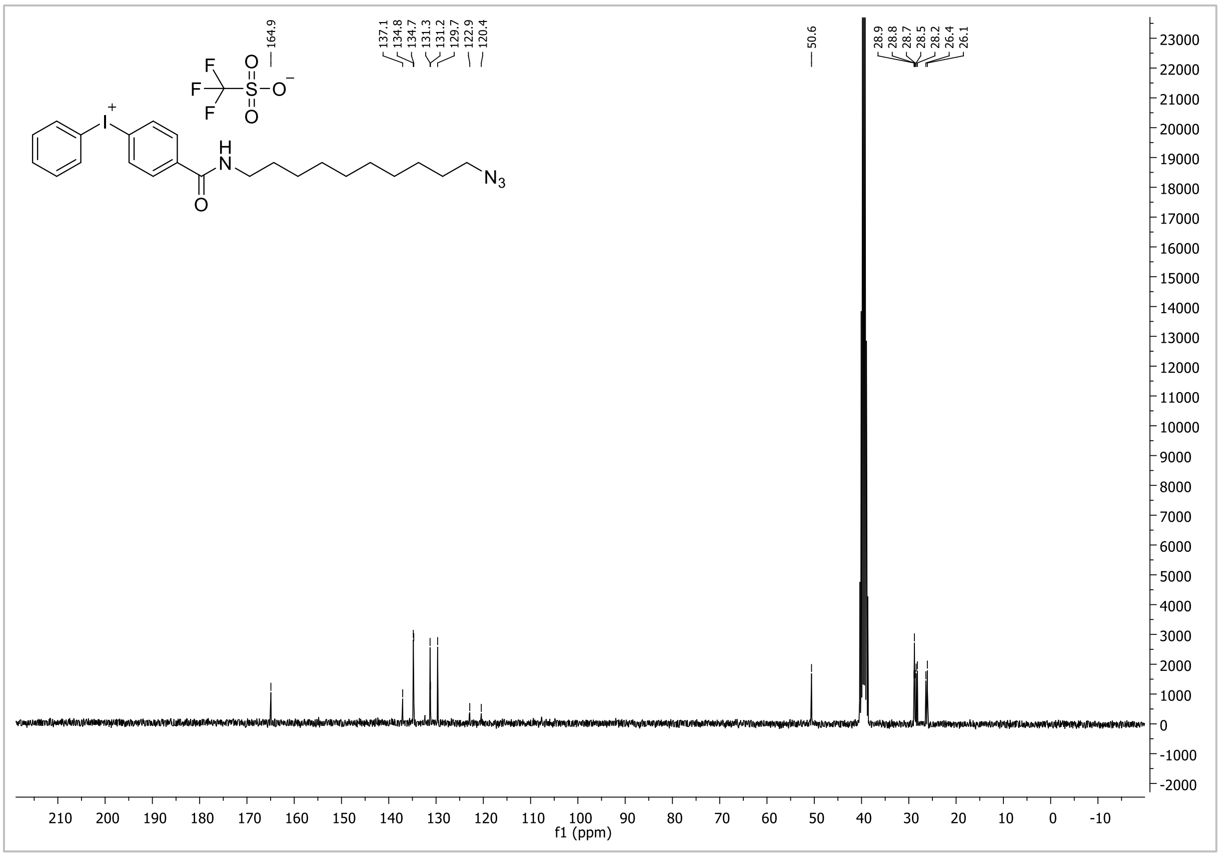

Figure S153. ^{13}C-NMR (75.53 MHz, DMSO-d_6) of compound **3aa**.

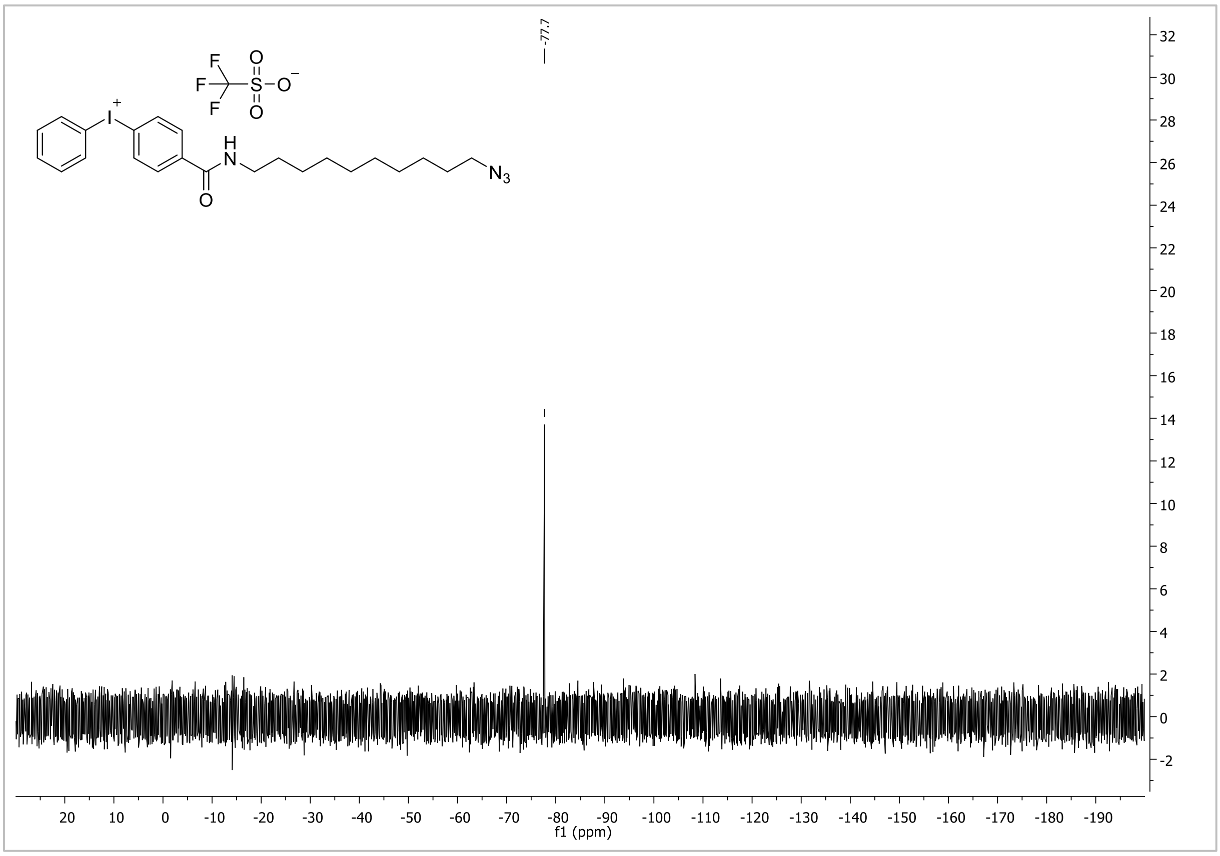

Figure S154. ^{19}F-NMR (470.53 MHz, DMSO-d_6) of compound **3aa**.

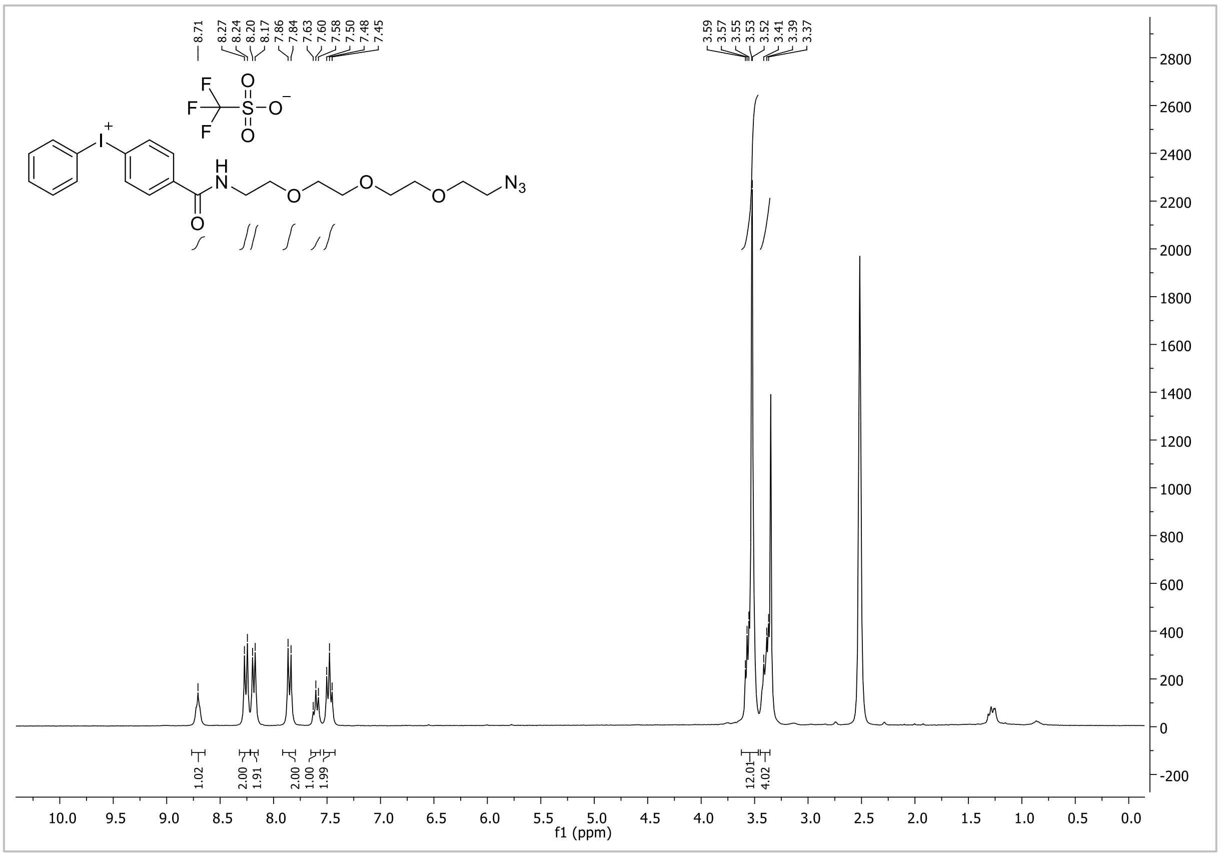

Figure S155. ^{1}H-NMR (300.36 MHz, DMSO-d_6) of compound **3ab**.

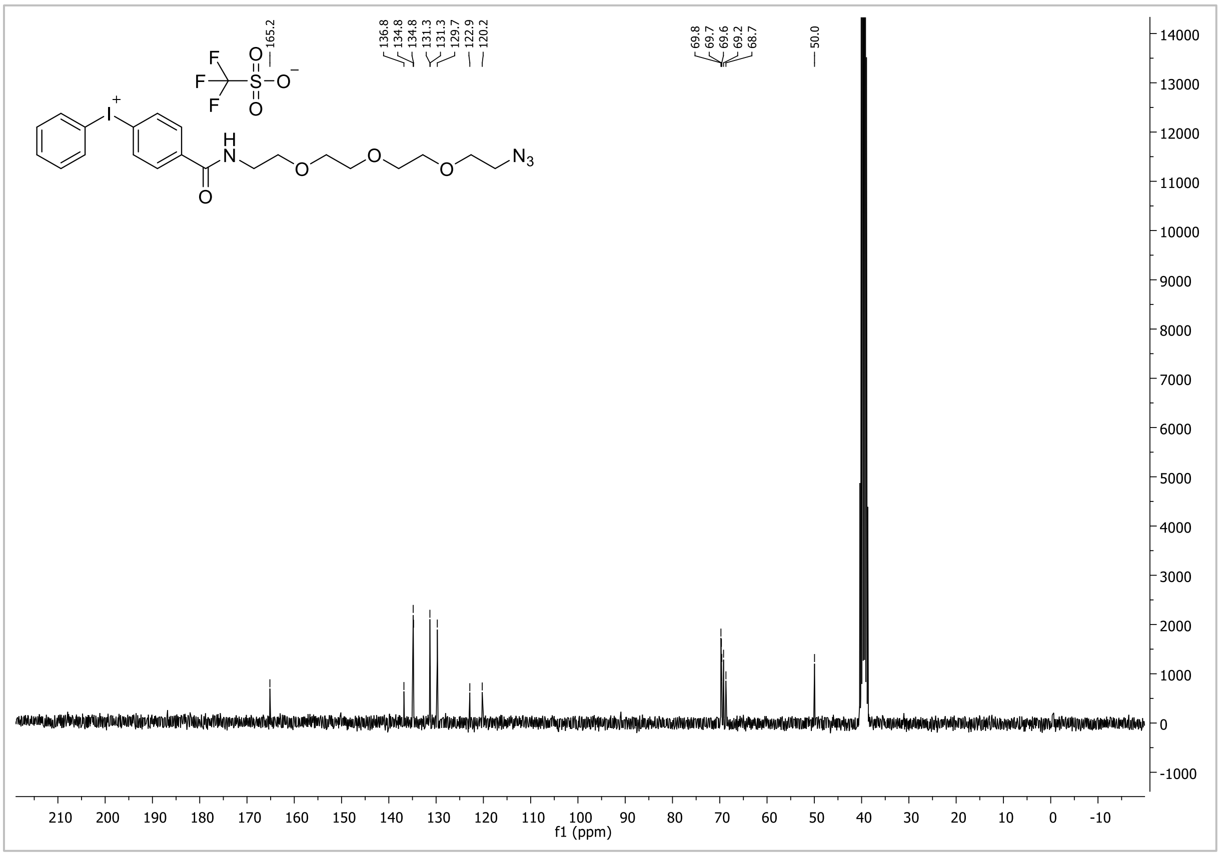

Figure S156. ^{13}C-NMR (75.53 MHz, DMSO-d_6) of compound **3ab**.

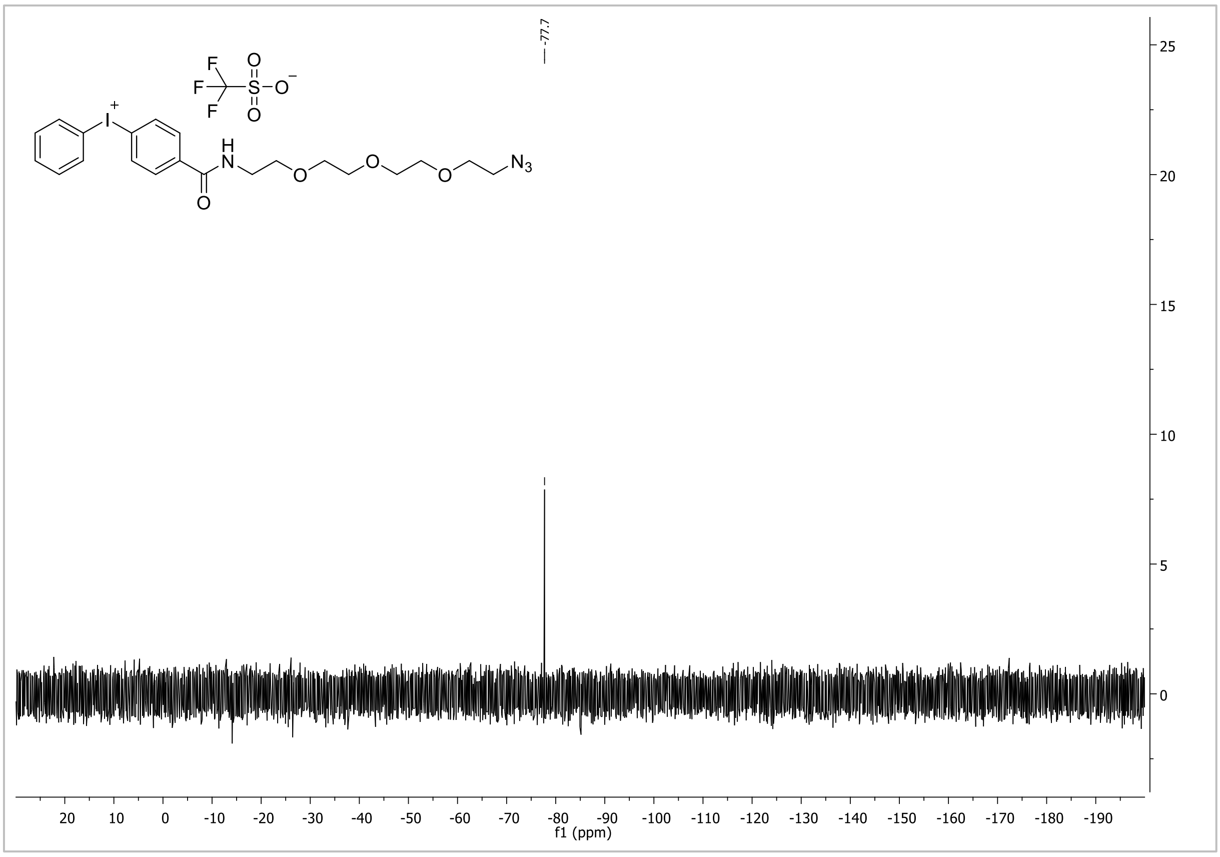

Figure S157. ^{19}F-NMR (470.53 MHz, DMSO-d_6) of compound **3ab**.

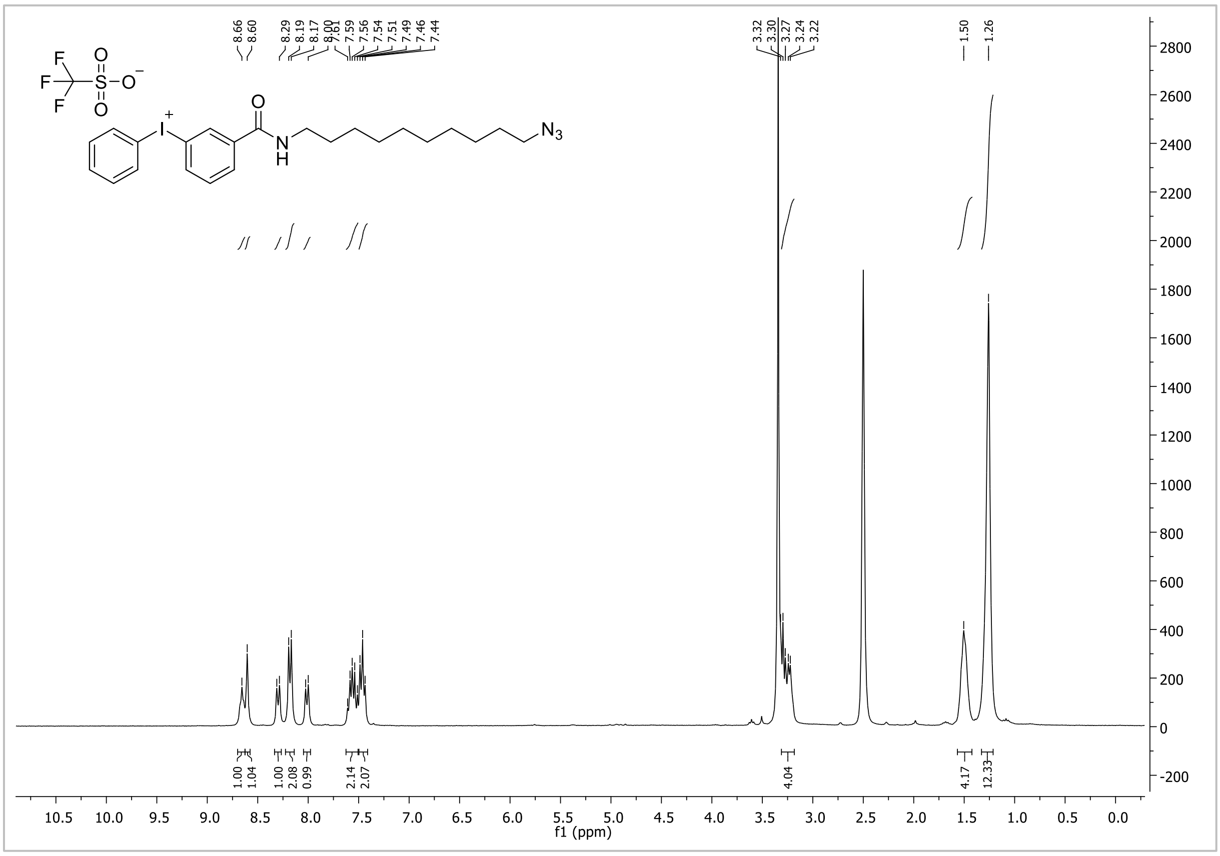

Figure S158. ^{1}H-NMR (300.36 MHz, DMSO-d_6) of compound **3ba**.

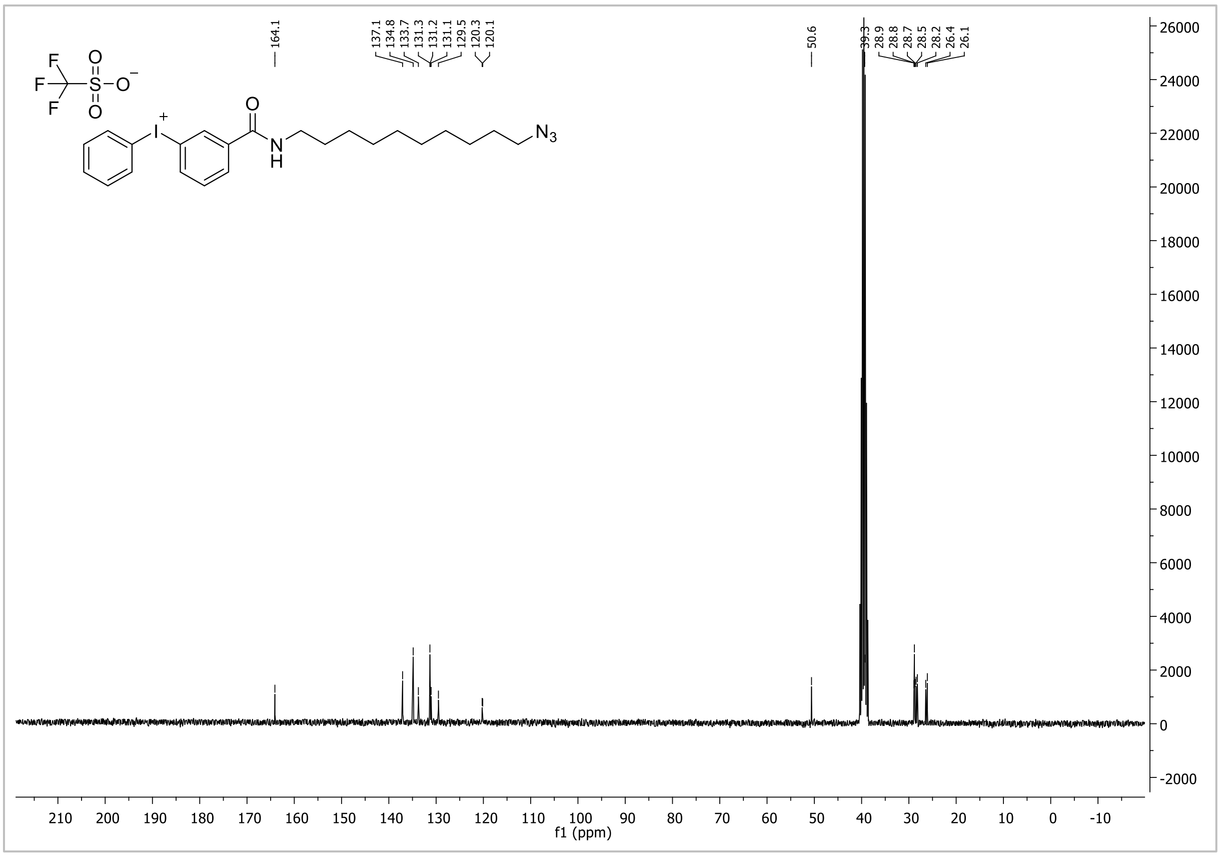

Figure S159. ^{13}C-NMR (75.53 MHz, DMSO-d_6) of compound **3ba**.

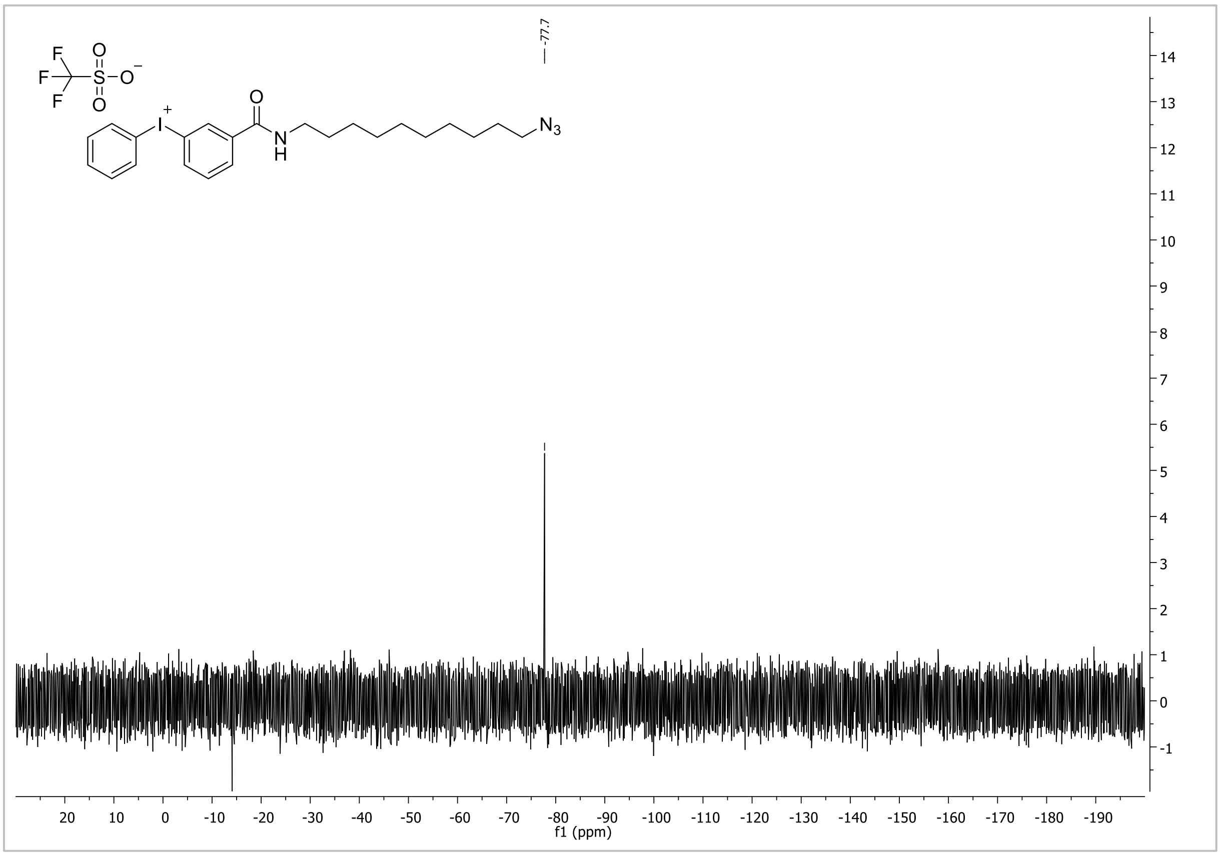

Figure S160. ^{19}F-NMR (470.53 MHz, DMSO-d_6) of compound **3ba**.

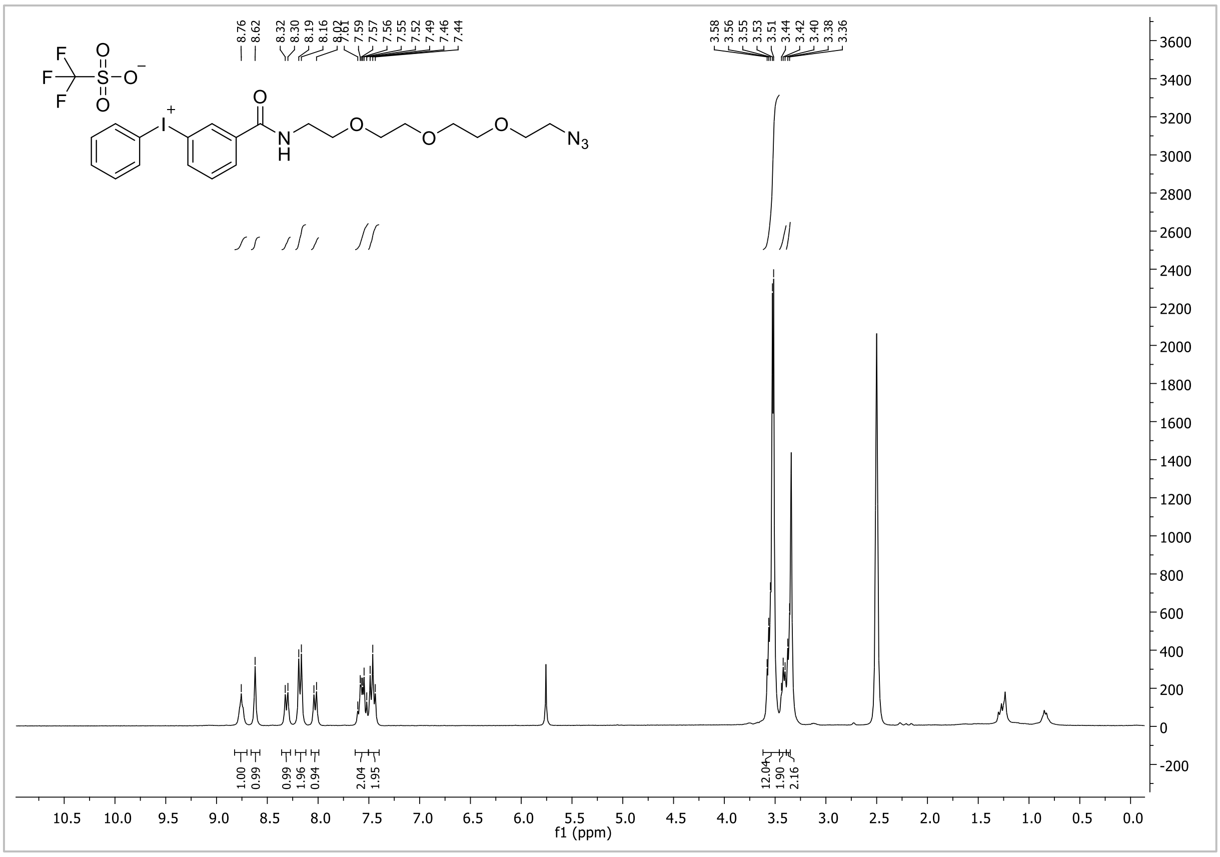

Figure S161. ^{1}H-NMR (300.36 MHz, DMSO-d_6) of compound **3bb**.

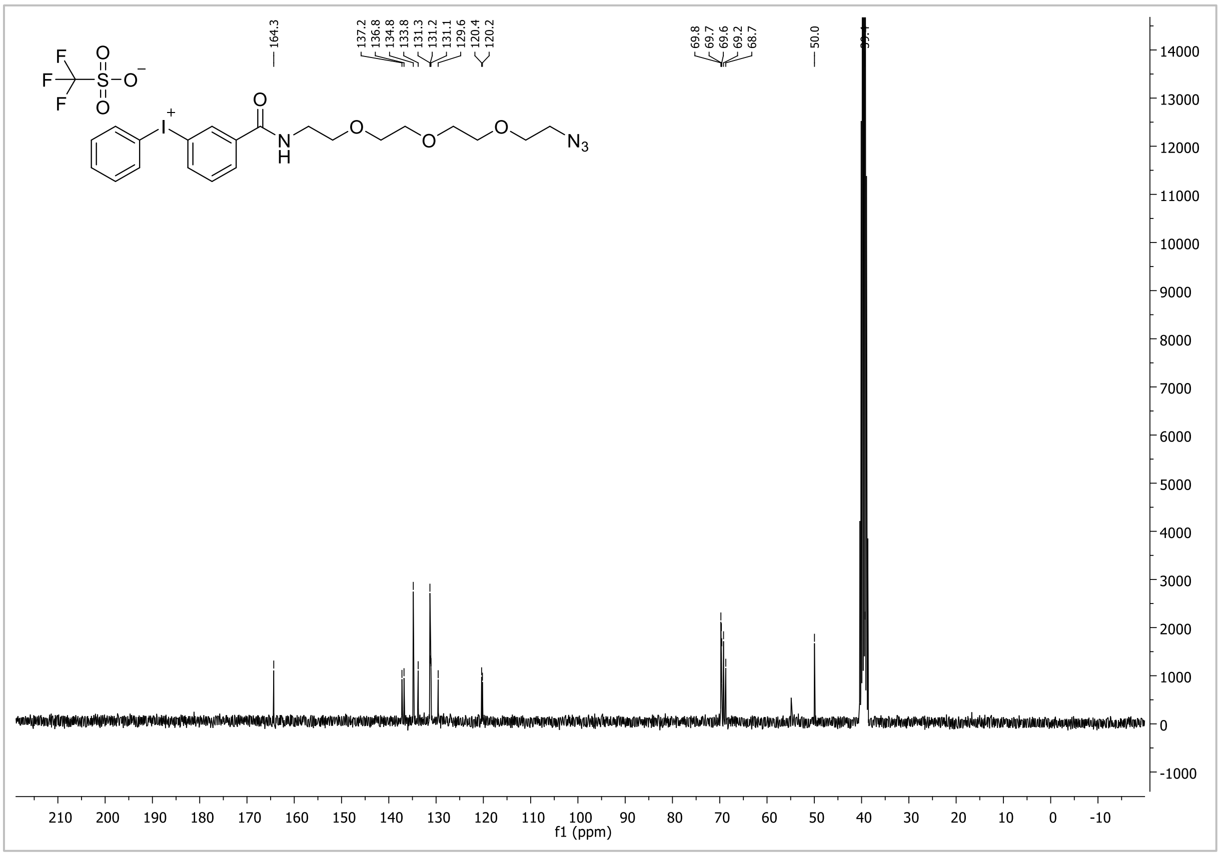

Figure S162. ^{13}C-NMR (75.53 MHz, DMSO-d_6) of compound **3bb**.

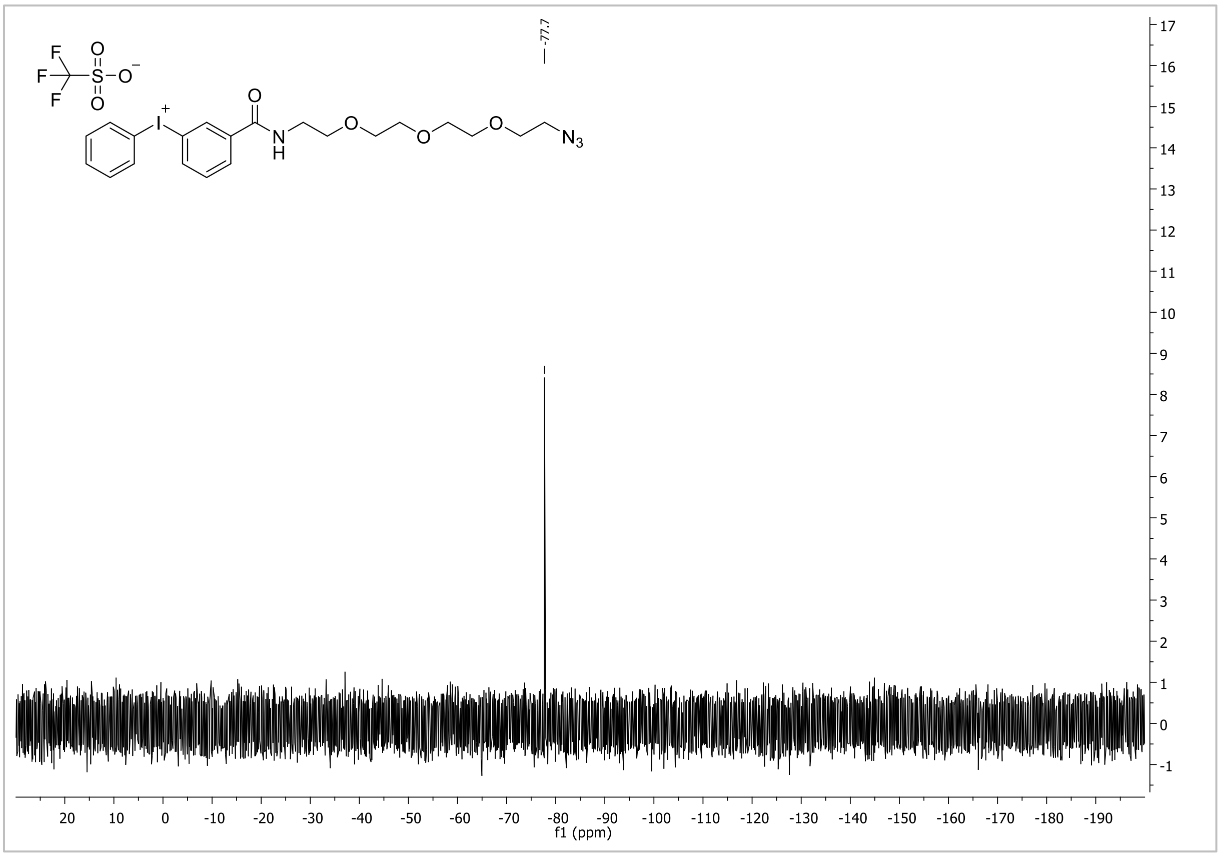

Figure S163. ^{19}F-NMR (470.53 MHz, DMSO-d_6) of compound **3bb**.

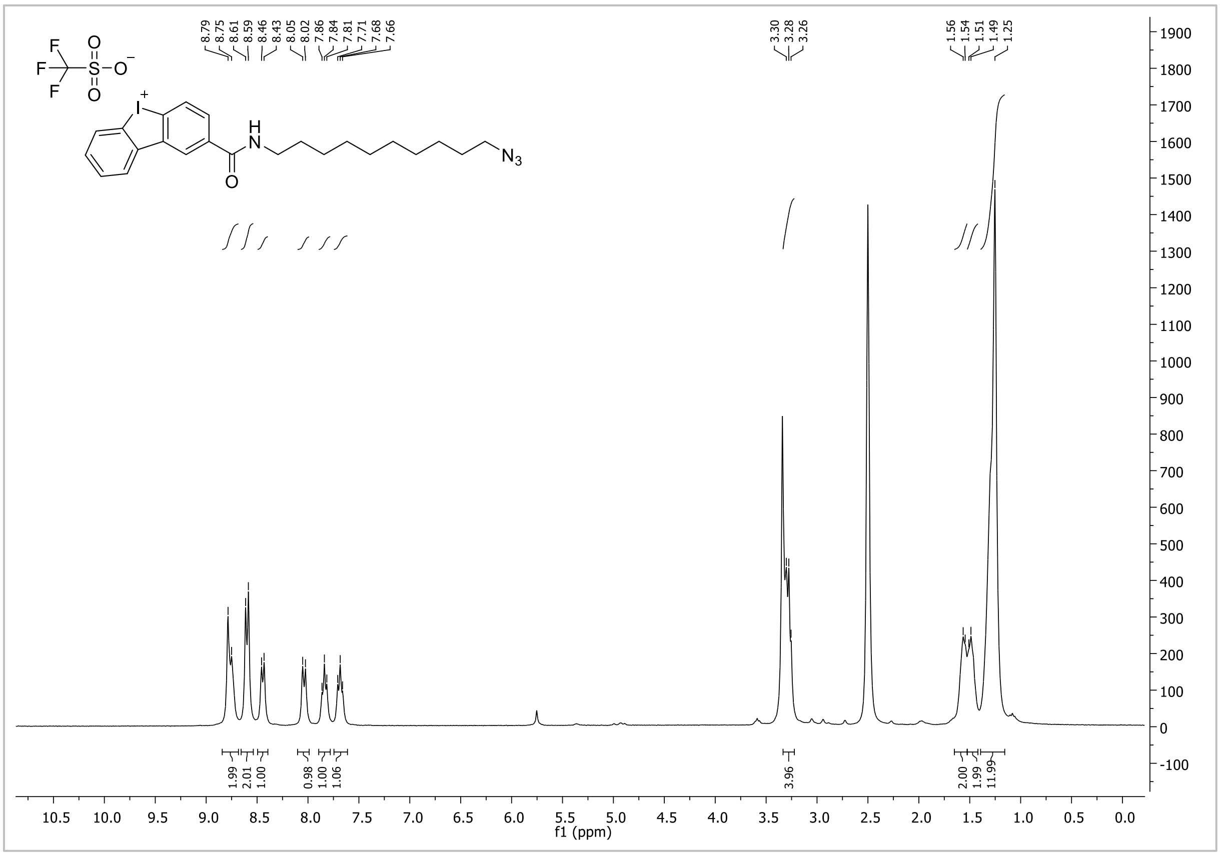

Figure S164. ^{1}H-NMR (300.36 MHz, DMSO-d_6) of compound **3ca**.

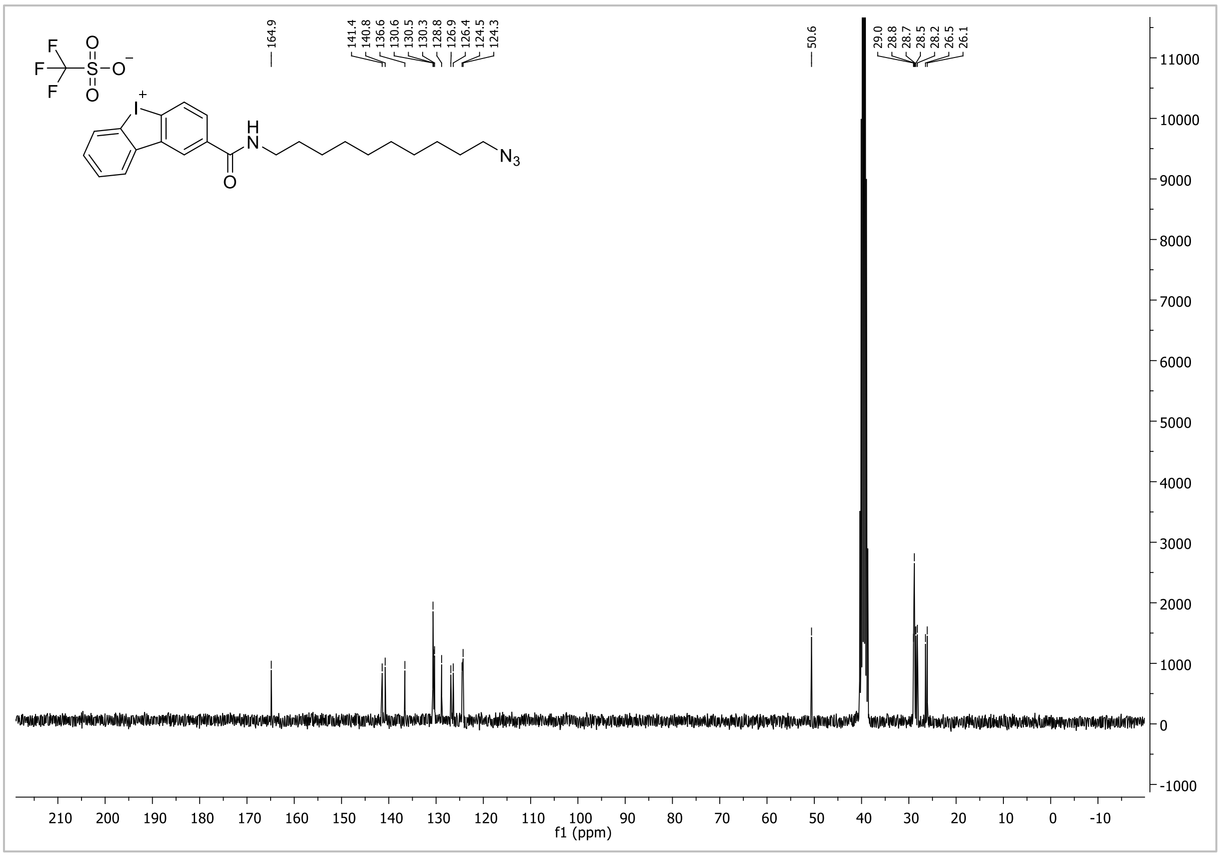

Figure S165. ^{13}C-NMR (75.53 MHz, DMSO-d_6) of compound **3ca**.

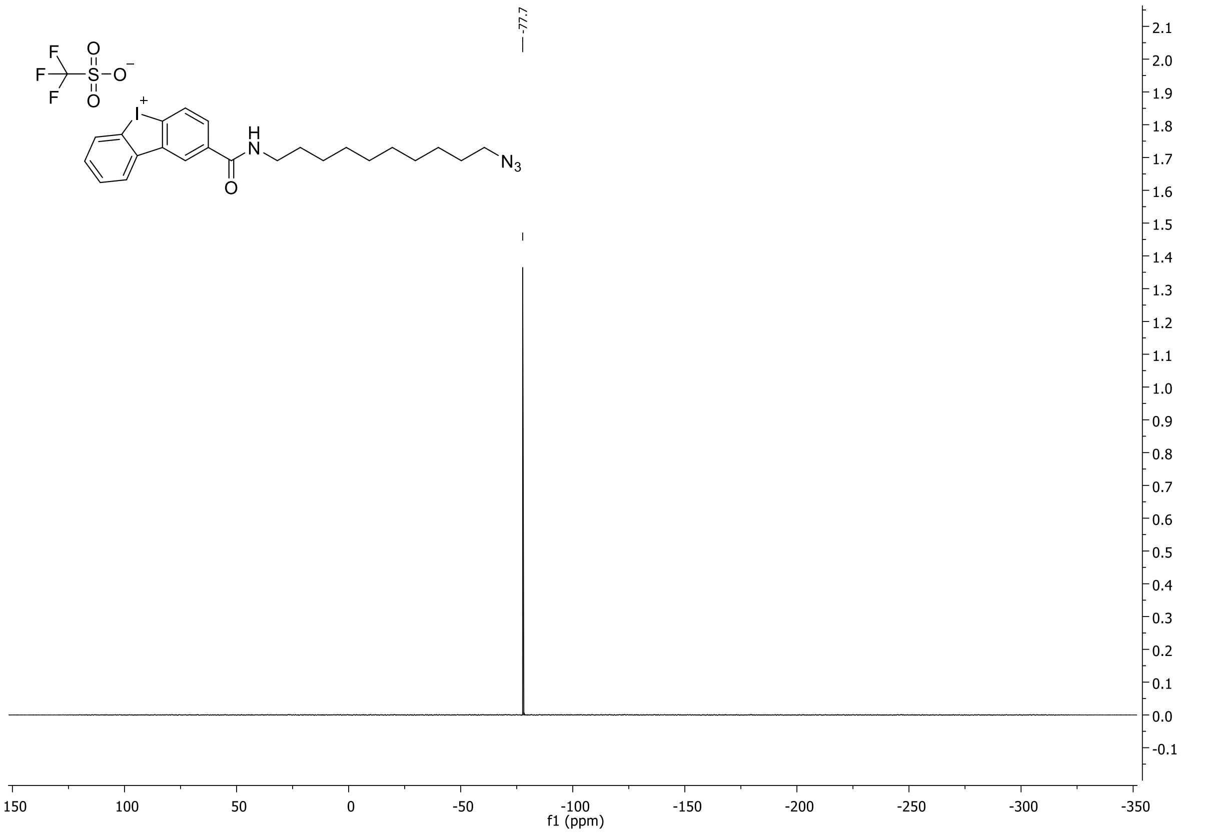

Figure S166. ^{19}F-NMR (376.17 MHz, DMSO-d_6) of compound **3ca**.

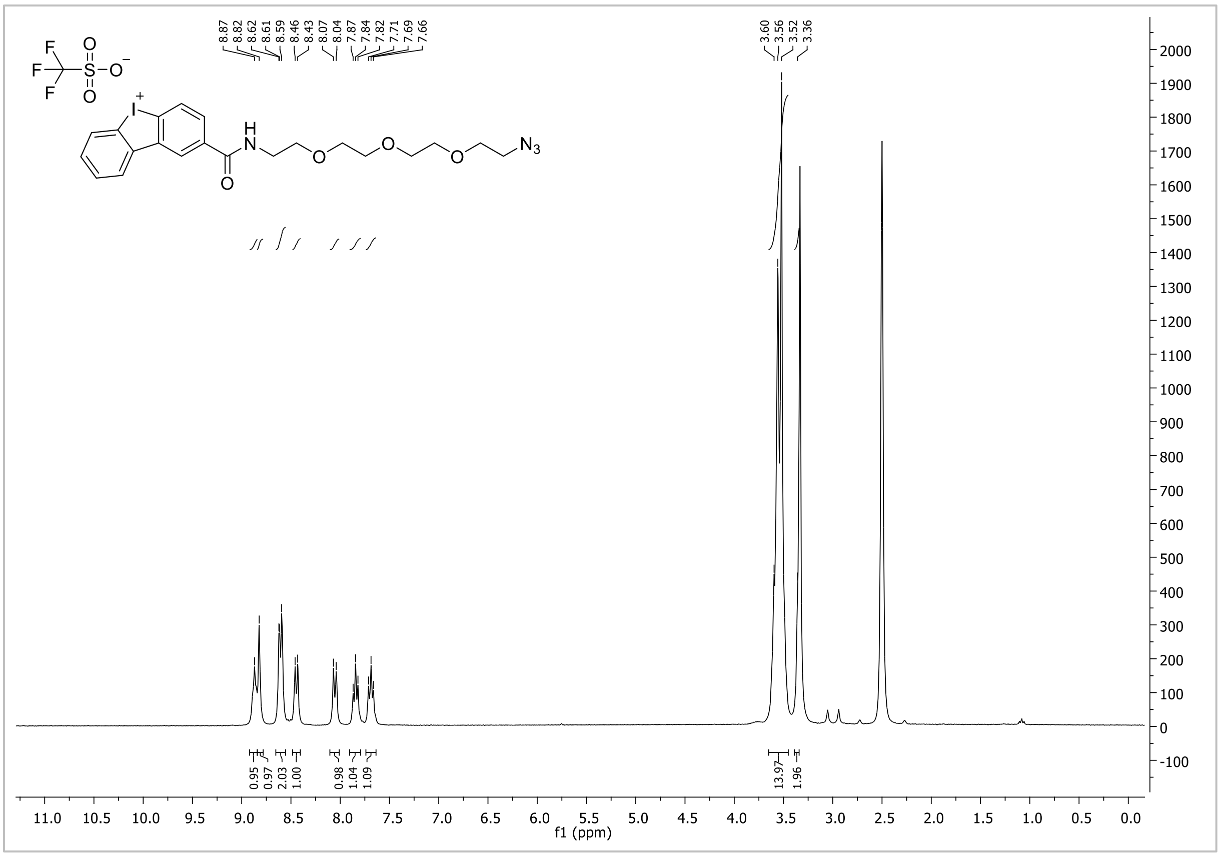

Figure S167. ^{1}H-NMR (300.36 MHz, DMSO-d_6) of compound **3cb**.

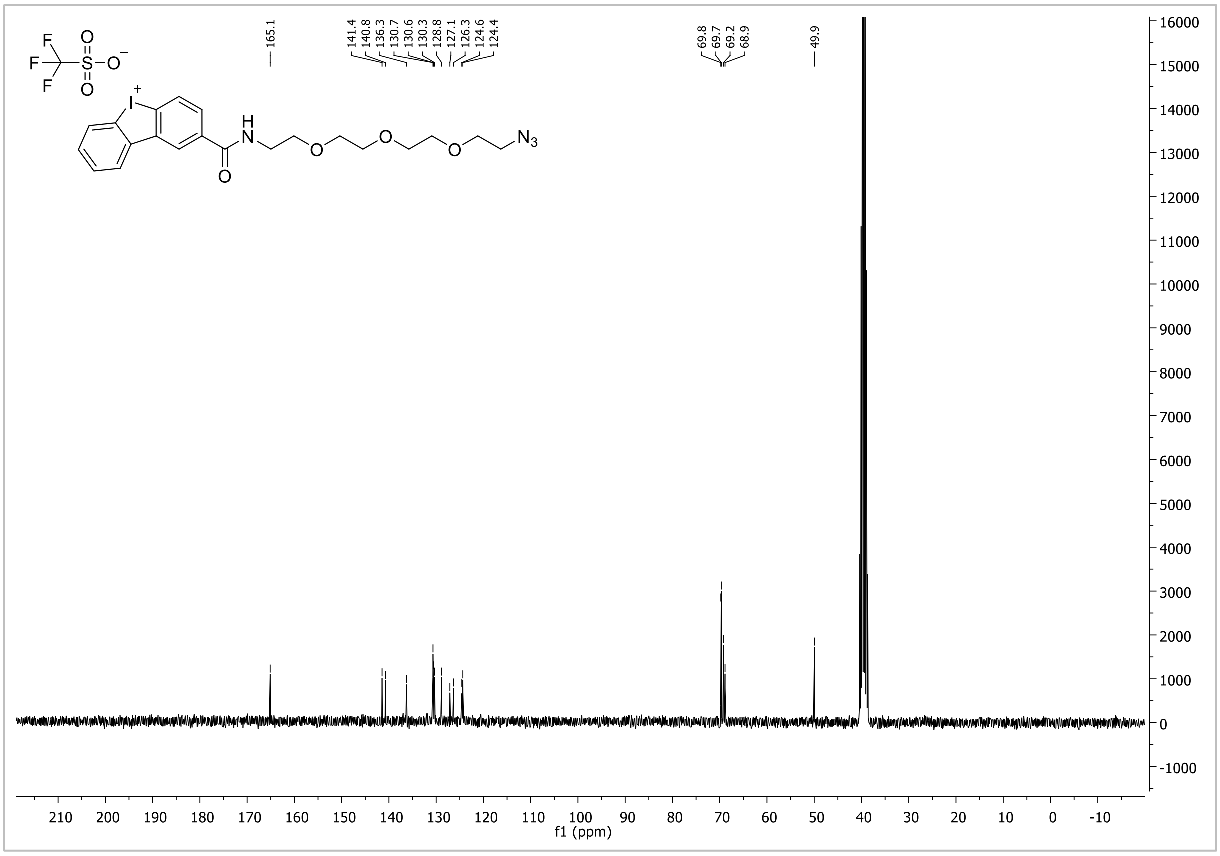

Figure S168. ^{13}C-NMR (75.53 MHz, DMSO-d_6) of compound **3cb**.

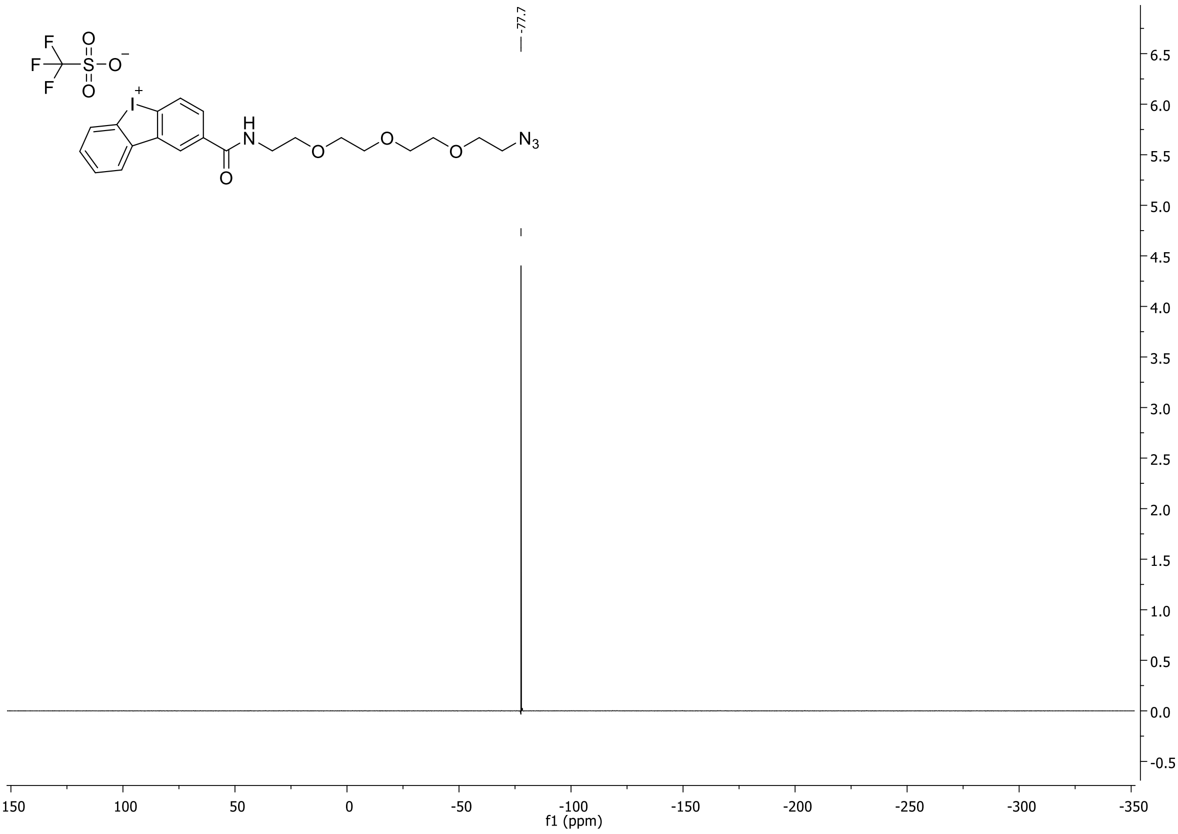

Figure S169. ^{19}F-NMR (376.17 MHz, DMSO-d_6) of compound **3cb**.

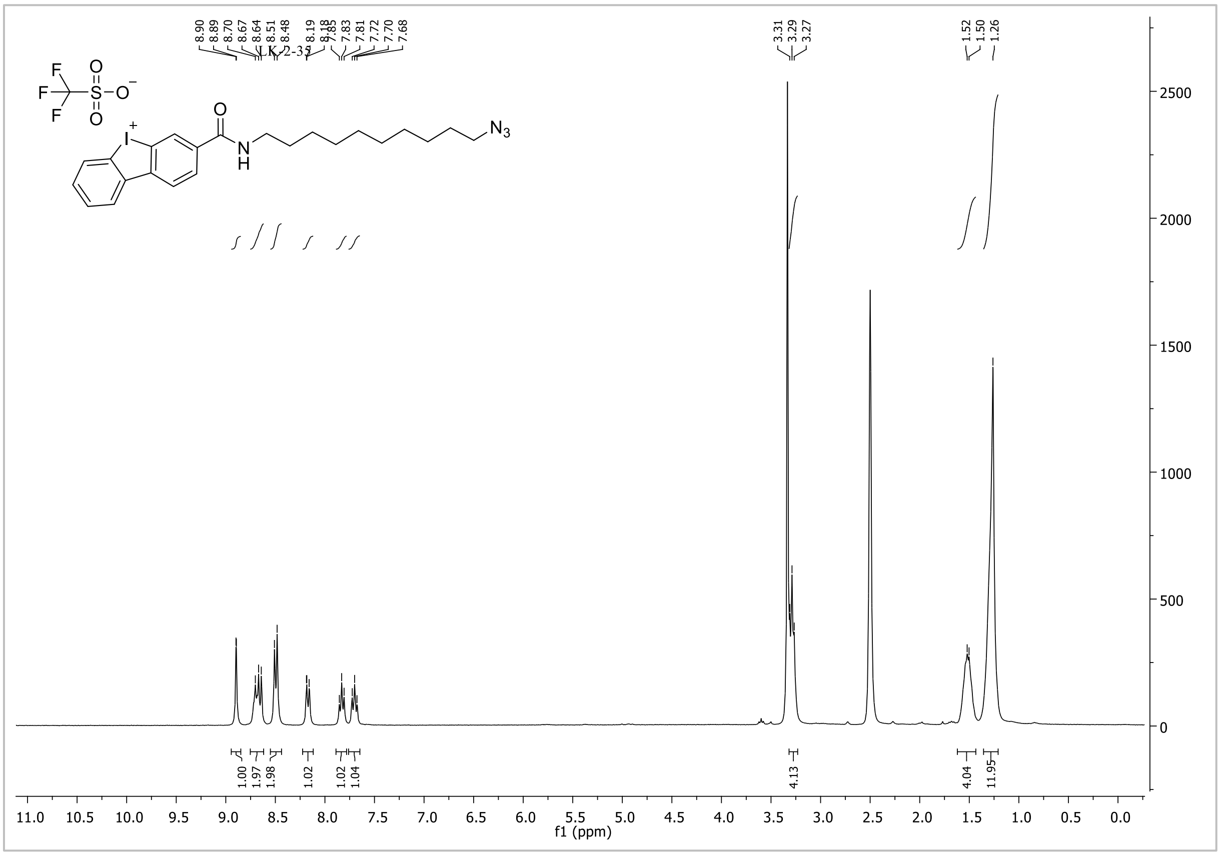

Figure S170. ^{1}H-NMR (300.36 MHz, DMSO-d_6) of compound **3da**.

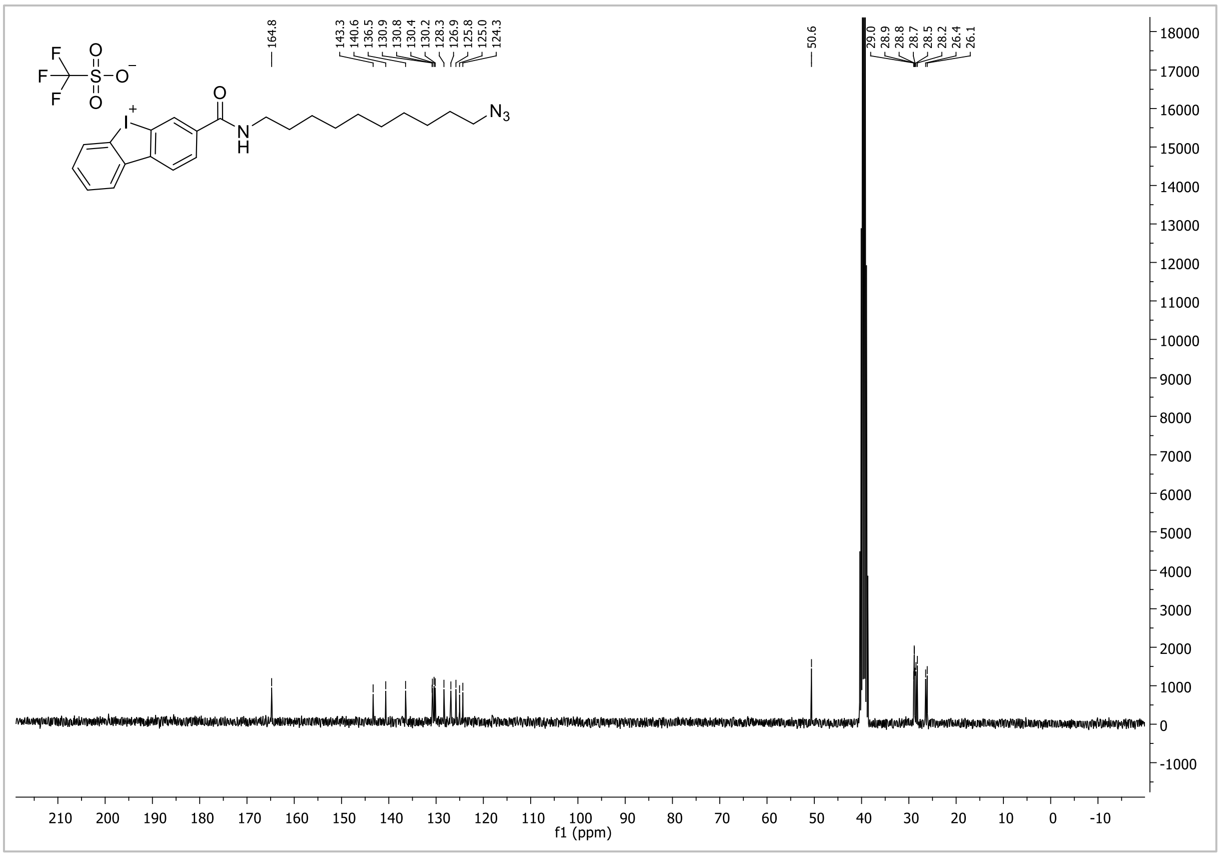

Figure S171. ^{13}C-NMR (75.53 MHz, DMSO-d_6) of compound **3da**.

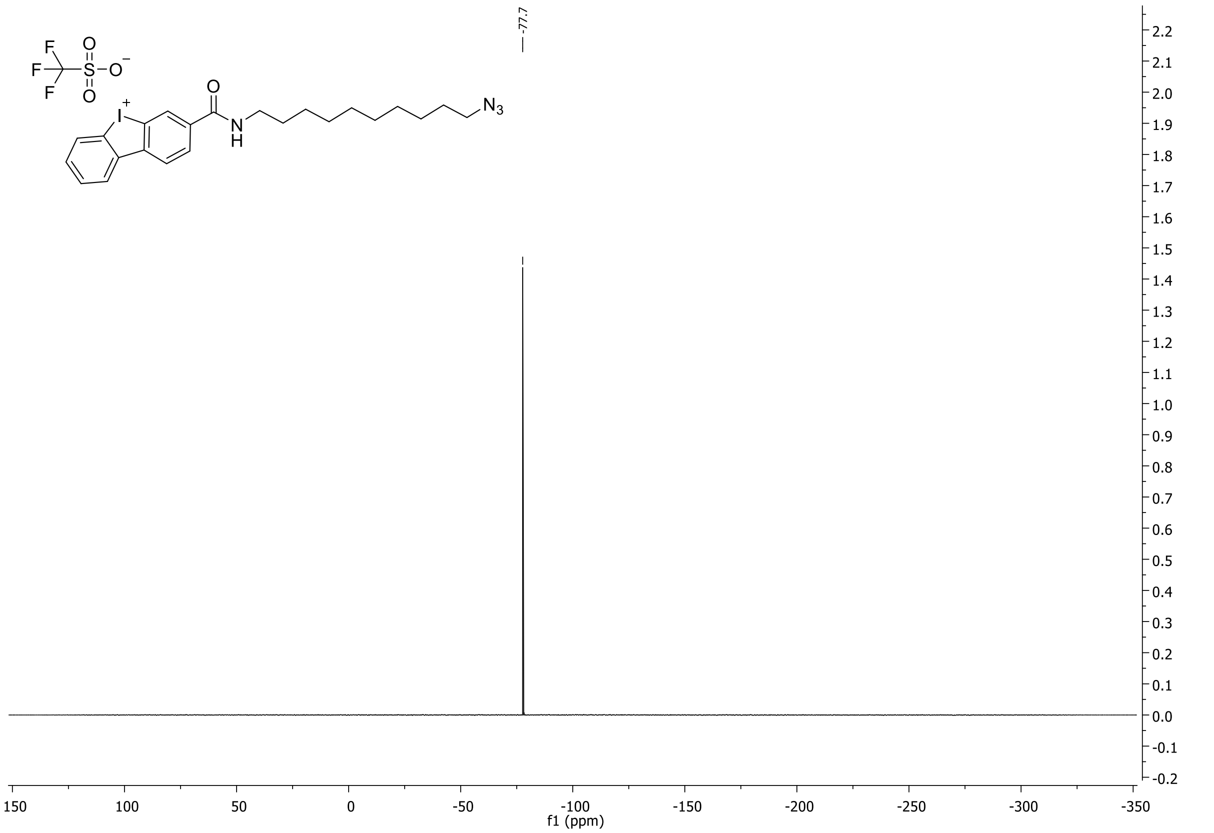

Figure S172. ^{19}F-NMR (376.17 MHz, DMSO-d_6) of compound **3da**.

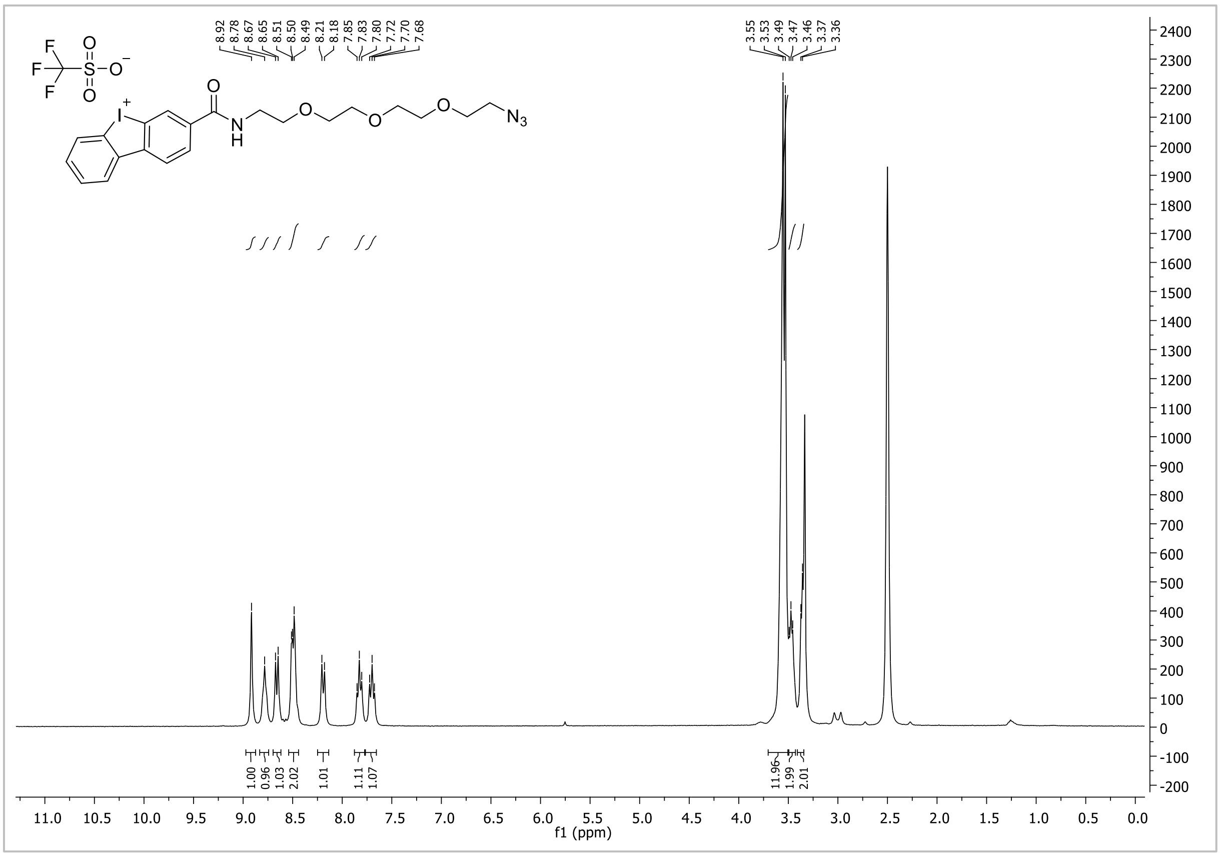

Figure S173. ^{1}H-NMR (300.36 MHz, DMSO-d_6) of compound **3db**.

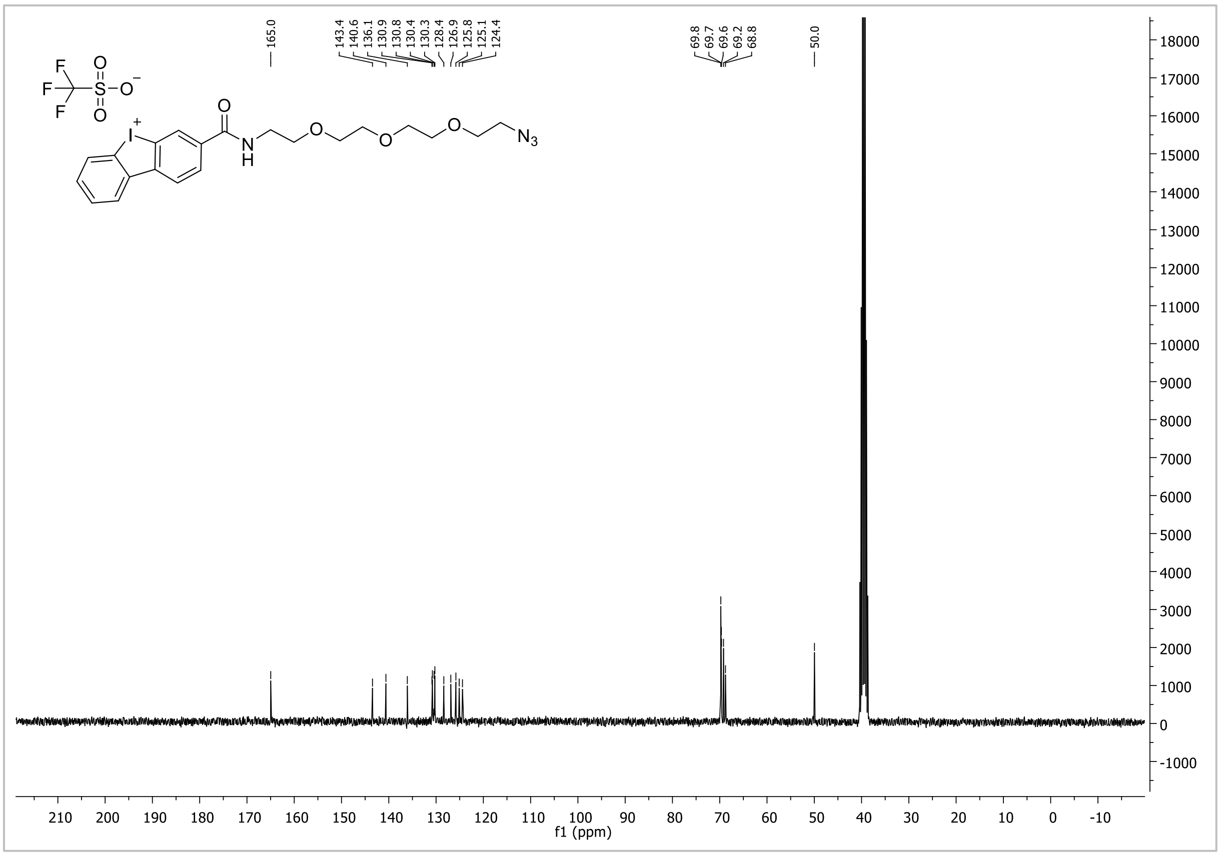

Figure S174. ^{13}C-NMR (75.53 MHz, DMSO-d_6) of compound **3db**.

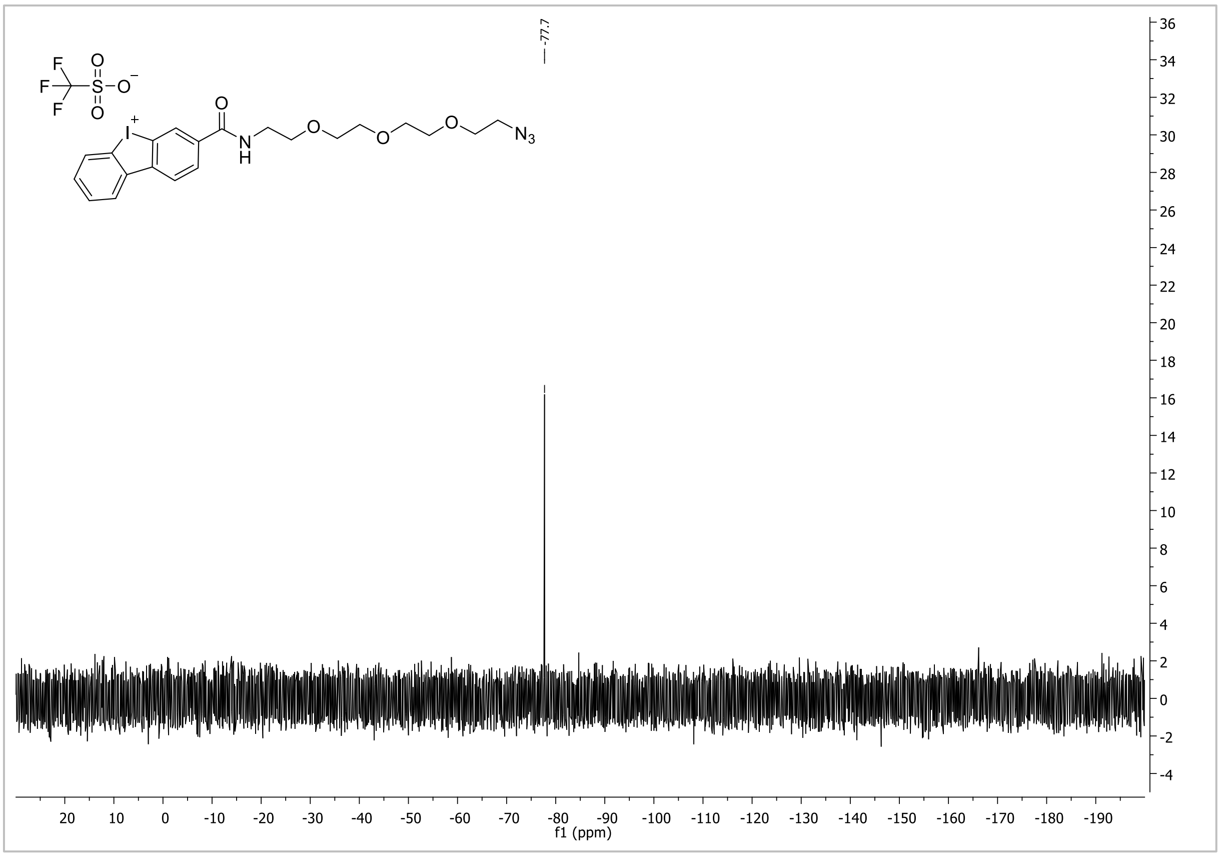

Figure S175. ^{19}F-NMR (470.53 MHz, DMSO-d_6) of compound **3db**.

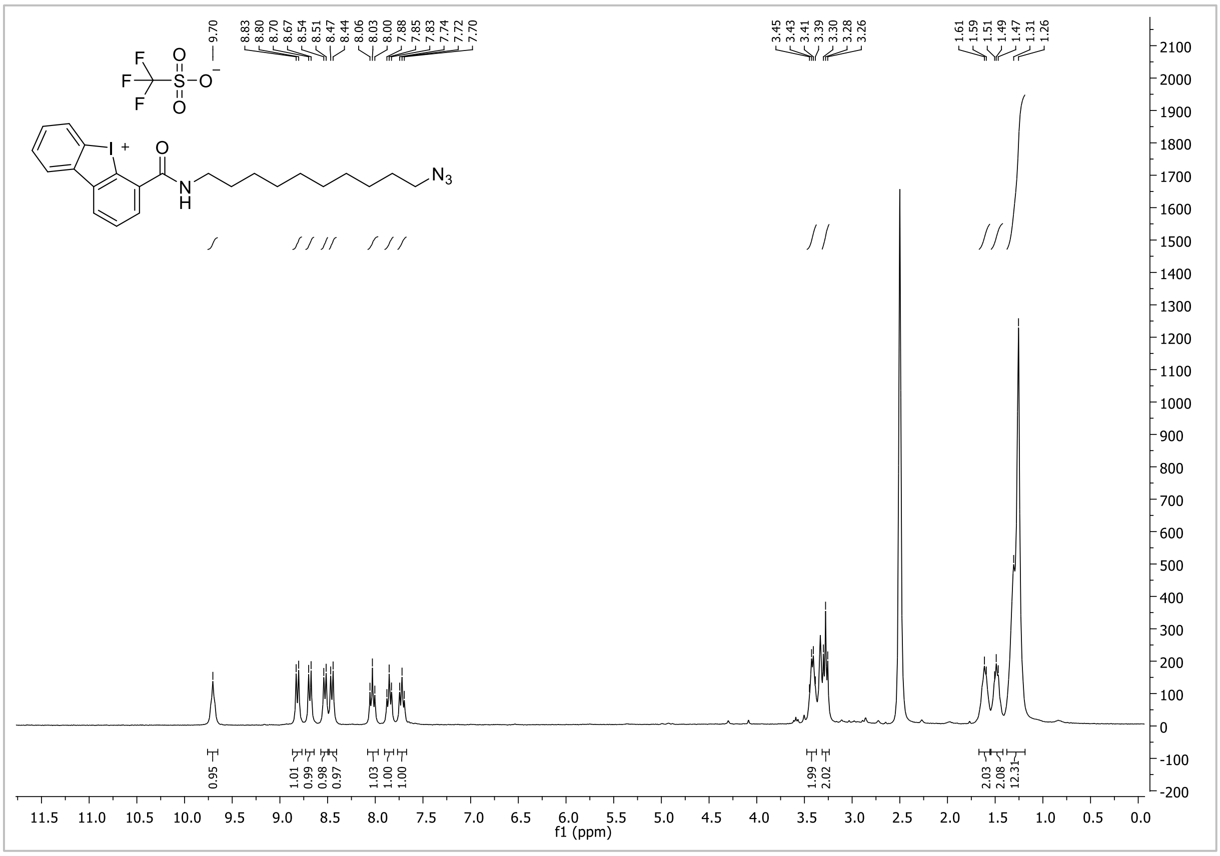

Figure S176. ^{1}H-NMR (300.36 MHz, DMSO-d_6) of compound **3ea**.

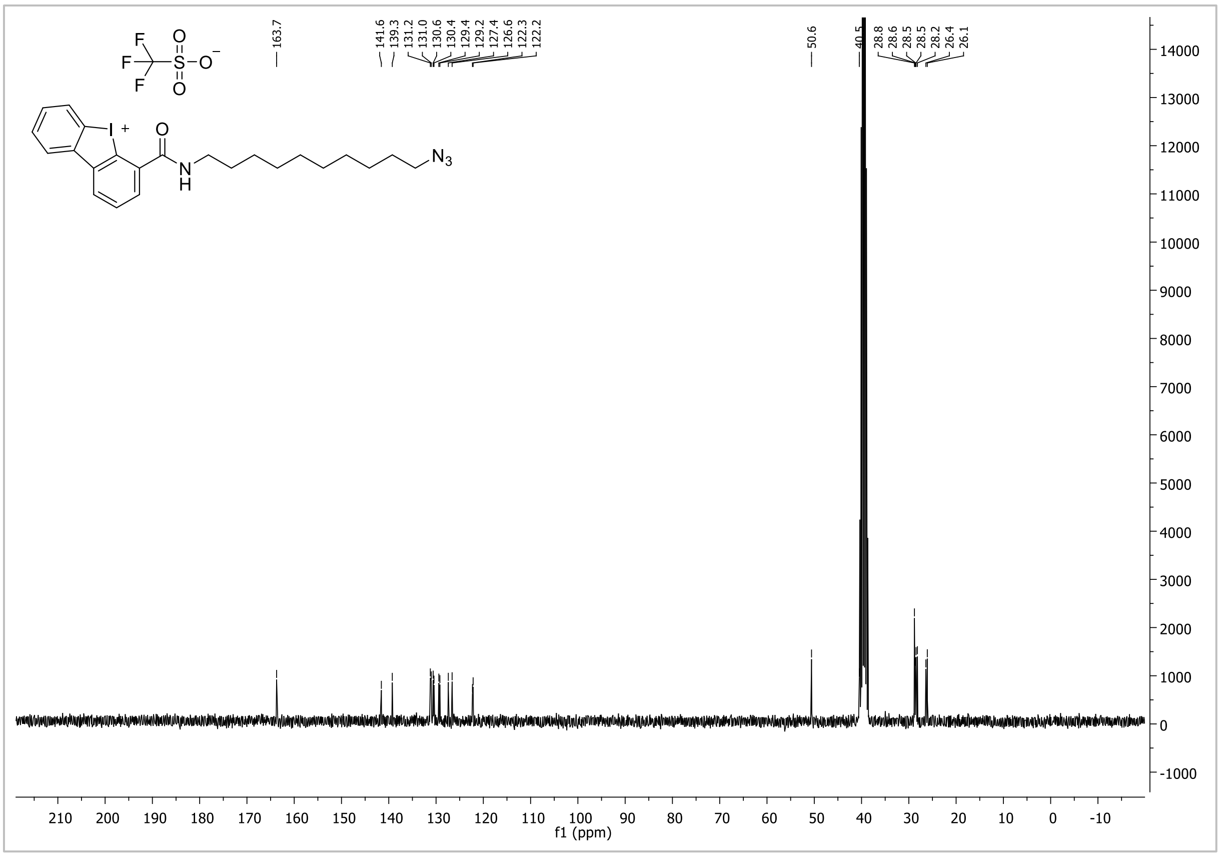

Figure S177. ^{13}C-NMR (75.53 MHz, DMSO-d_6) of compound **3ea**.

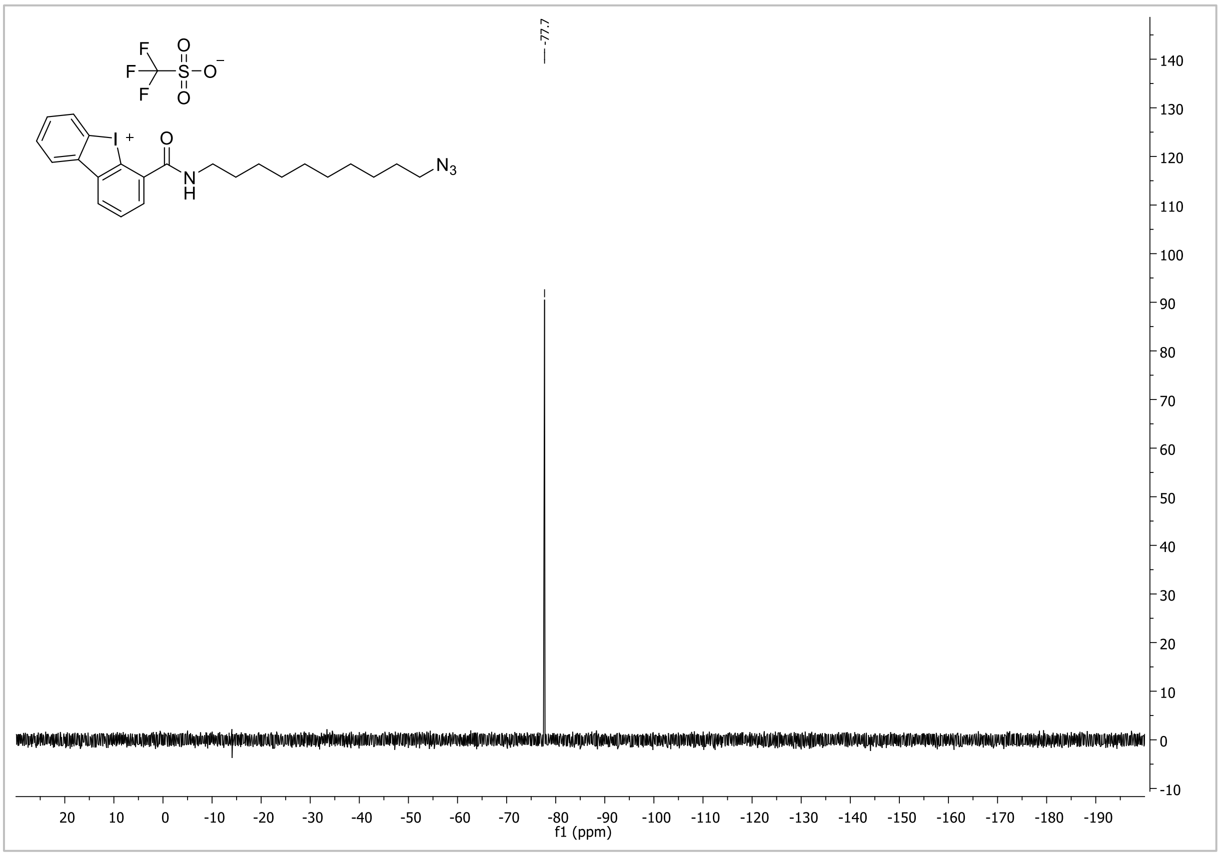

Figure S178. ^{19}F-NMR (470.53 MHz, DMSO-d_6) of compound **3ea**.

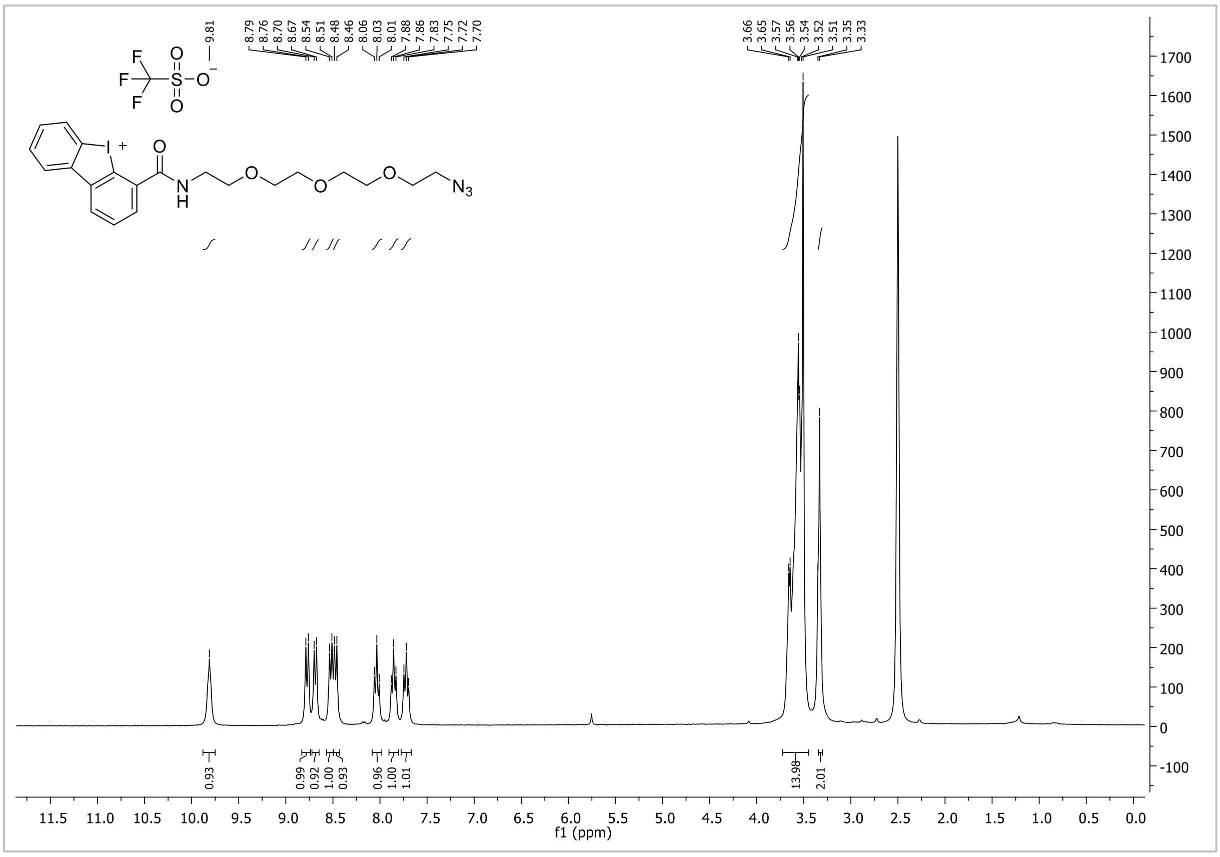

Figure S179. ^{1}H-NMR (300.36 MHz, DMSO-d_6) of compound **3eb**.

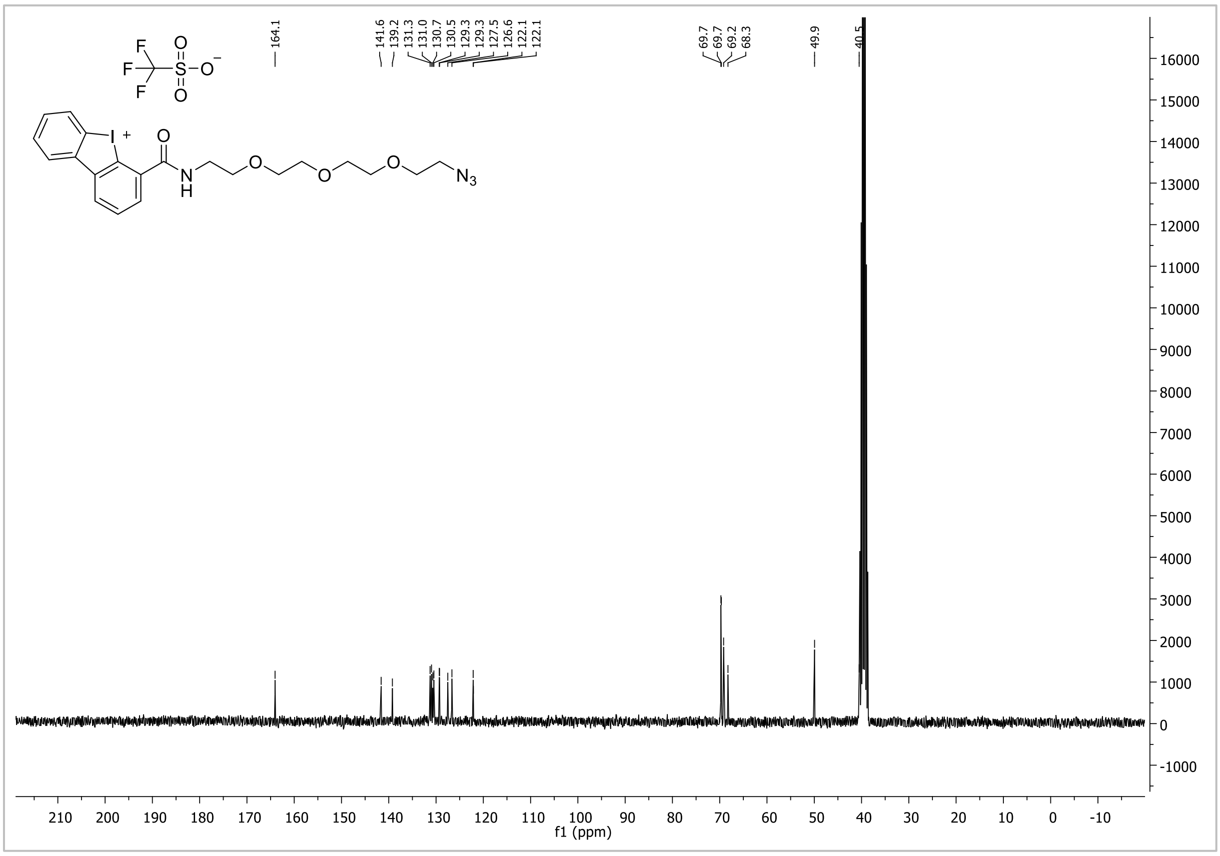

Figure S180. ^{13}C-NMR (75.53 MHz, DMSO-d_6) of compound **3eb**.

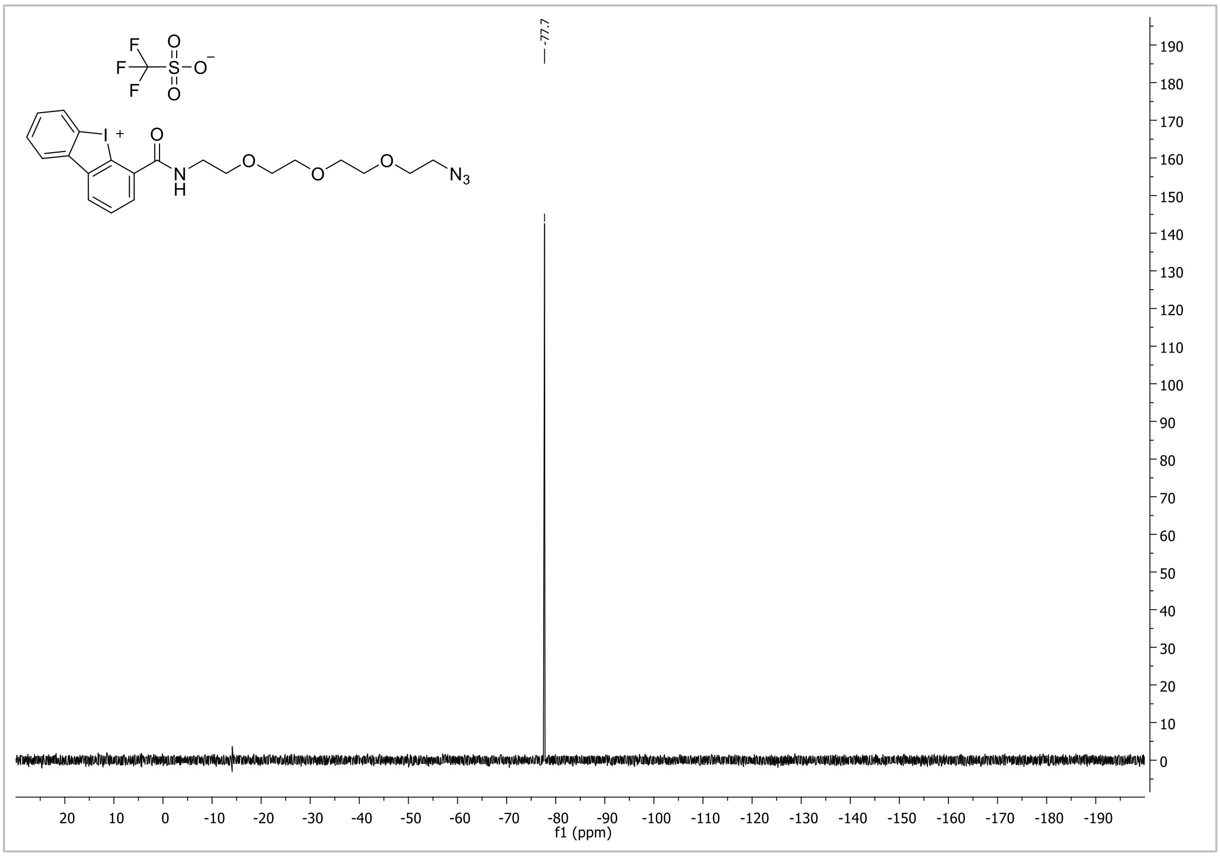

Figure S181. ^{19}F-NMR (470.53 MHz, DMSO-d_6) of compound **3eb**.

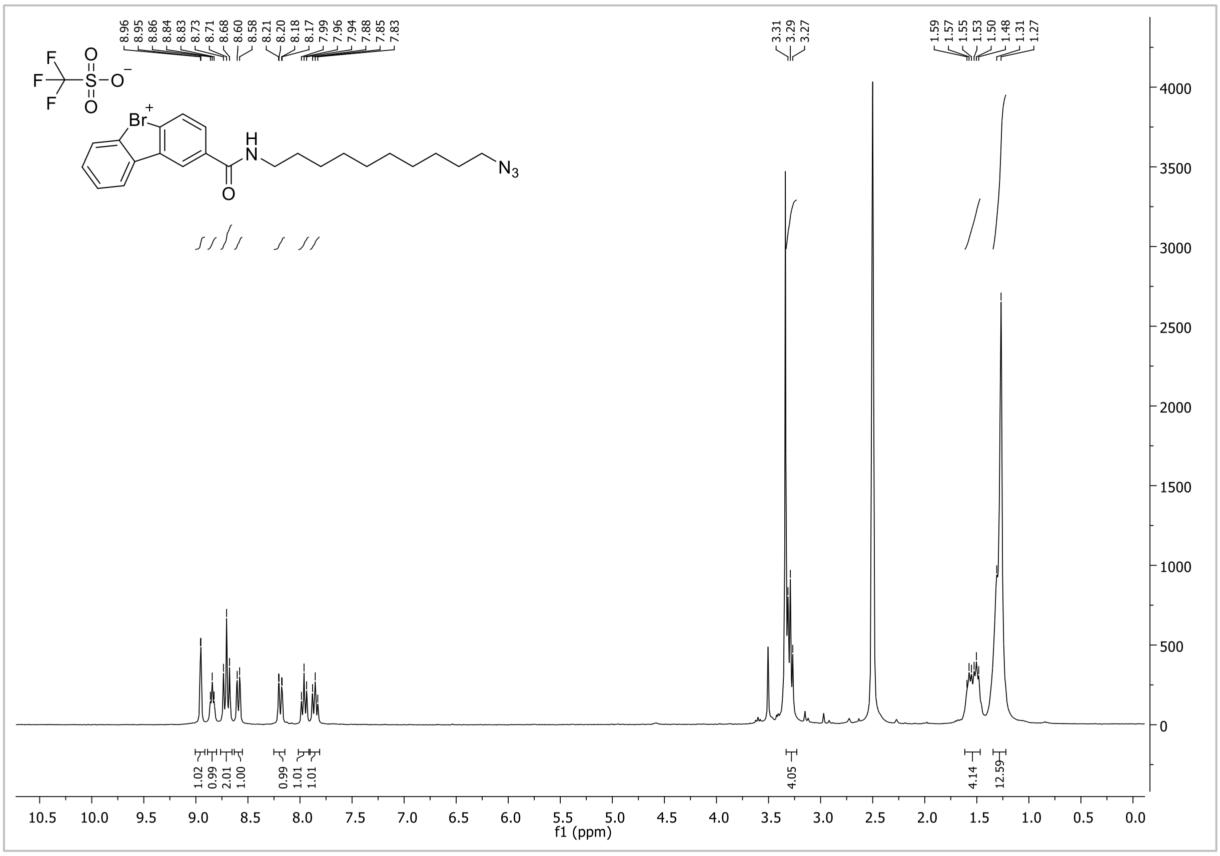

Figure S182. ^{1}H-NMR (300.13 MHz, DMSO-d_6) of compound **3fa**.

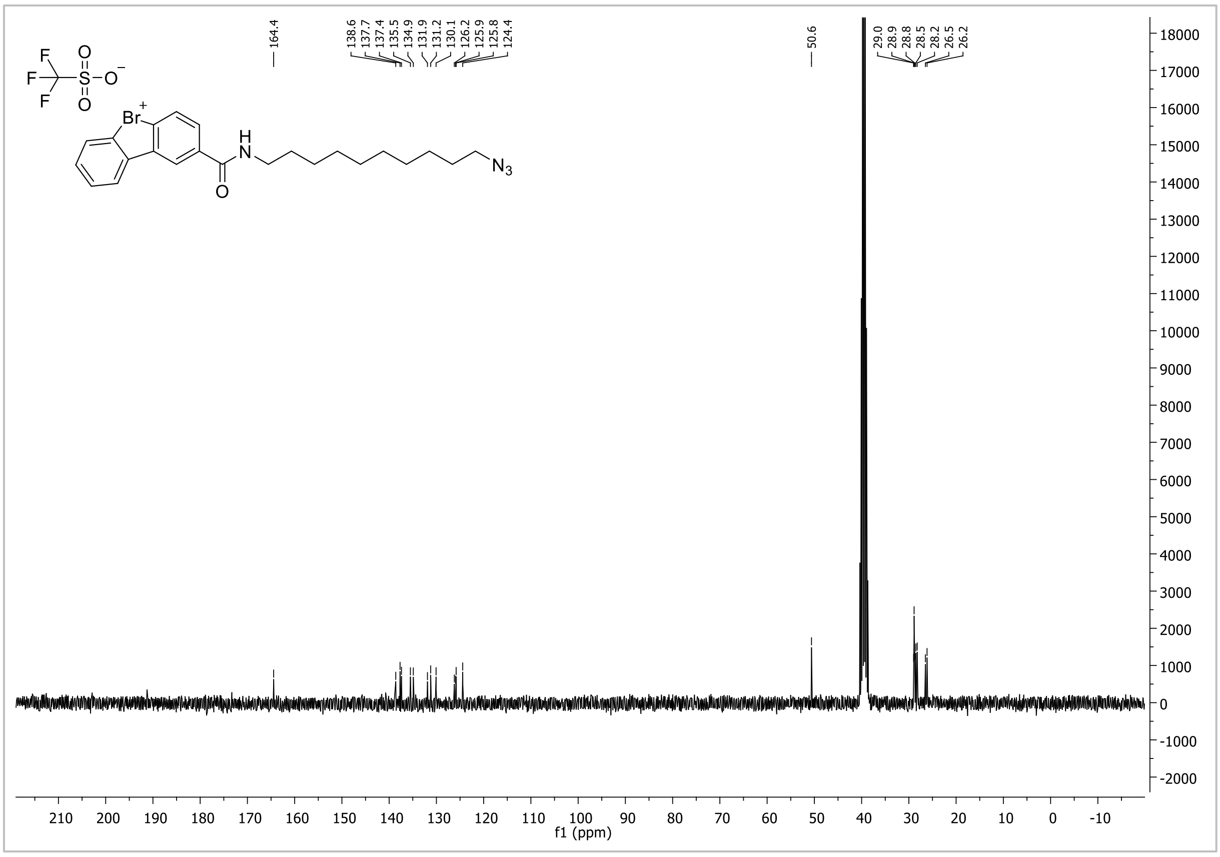

Figure S183. ^{13}C-NMR (75.47 MHz, DMSO-d_6) of compound **3fa**.

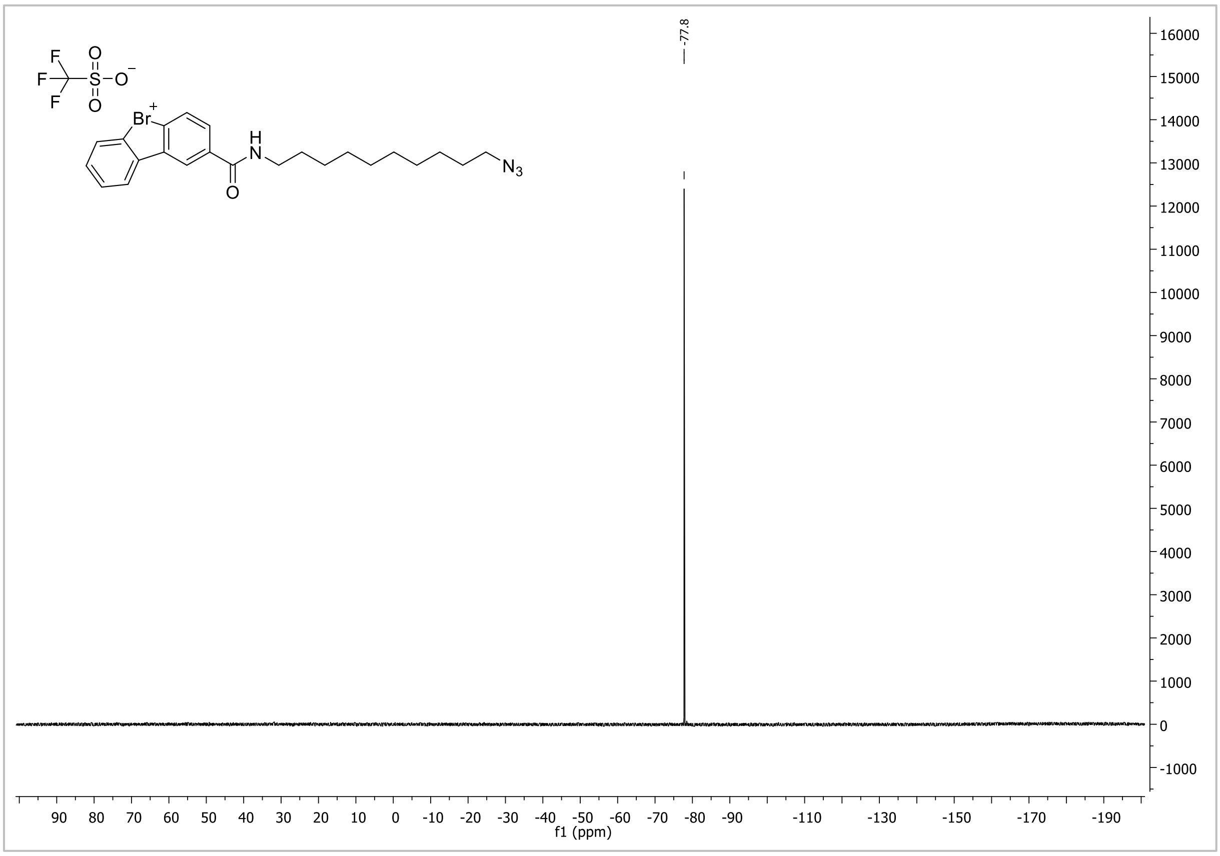

Figure S184. ^{19}F-NMR (282.40 MHz, DMSO-d_6) of compound **3fa**.

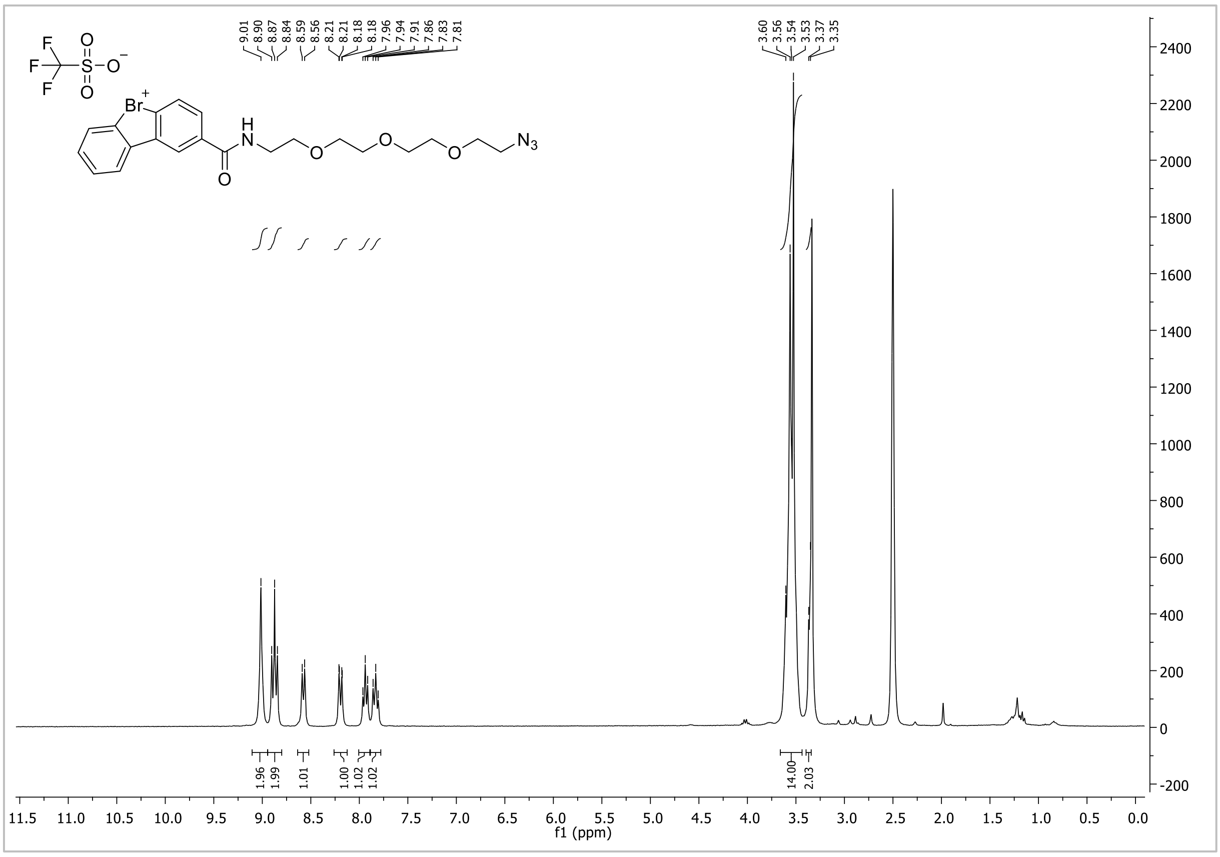

Figure S185. ^{1}H-NMR (300.13 MHz, DMSO-d_6) of compound **3fb**.

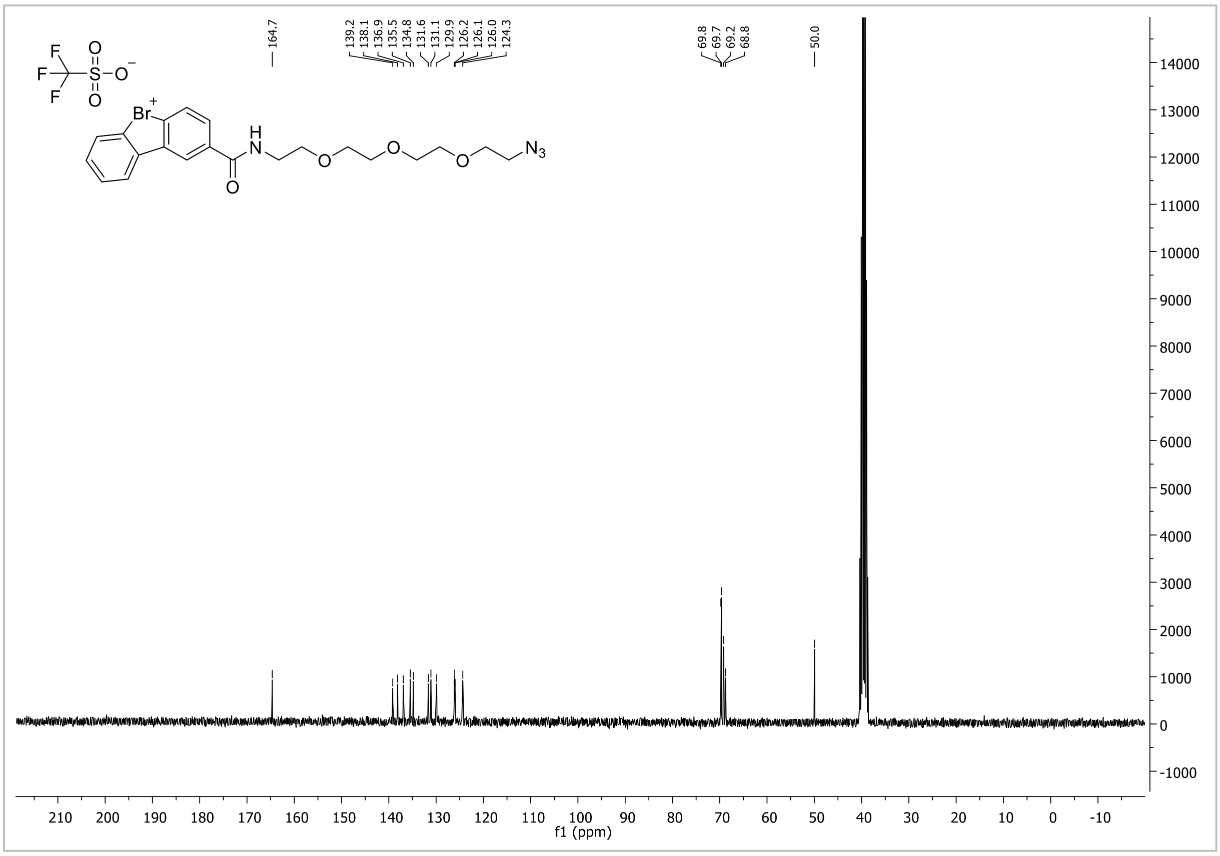

Figure S186. ^{13}C-NMR (75.47 MHz, DMSO-d_6) of compound **3fb**.

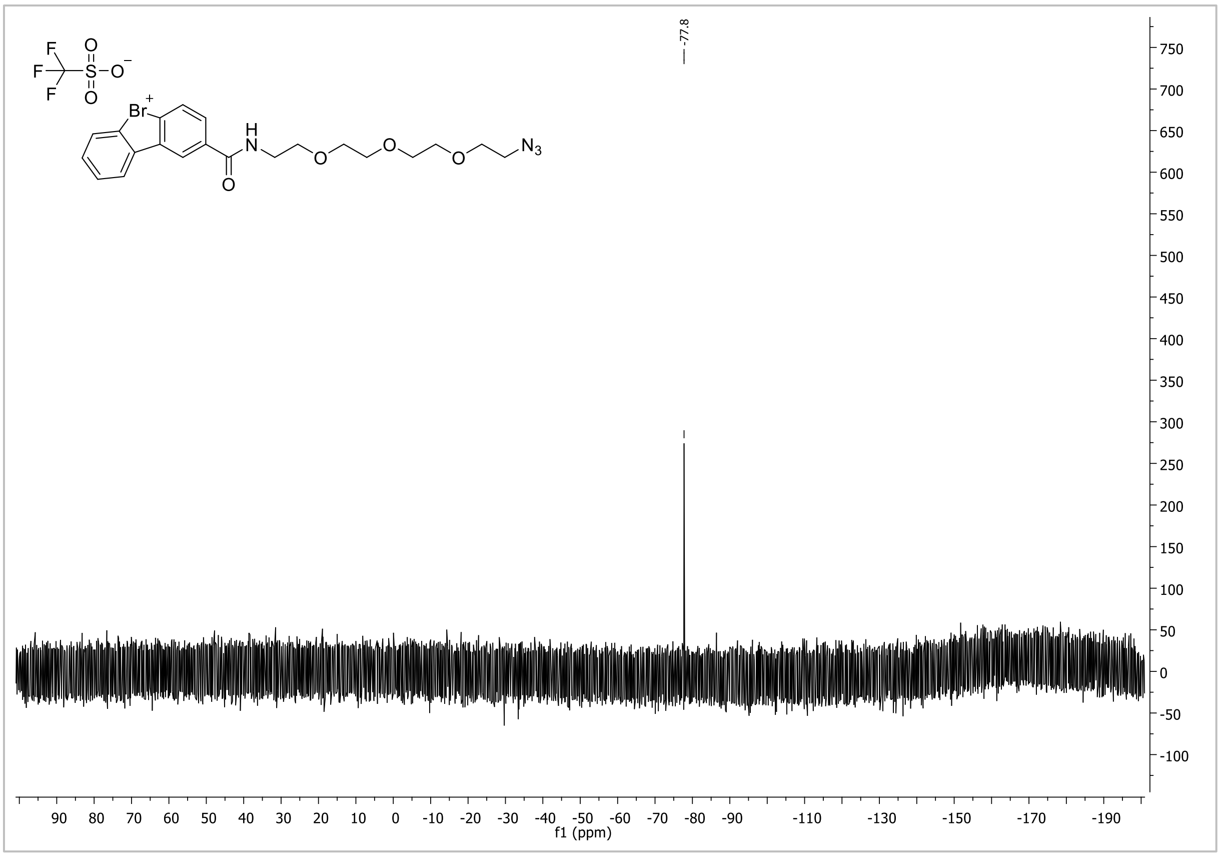

Figure S187. ^{19}F-NMR (282.40 MHz, DMSO-d_6) of compound **3fb**.

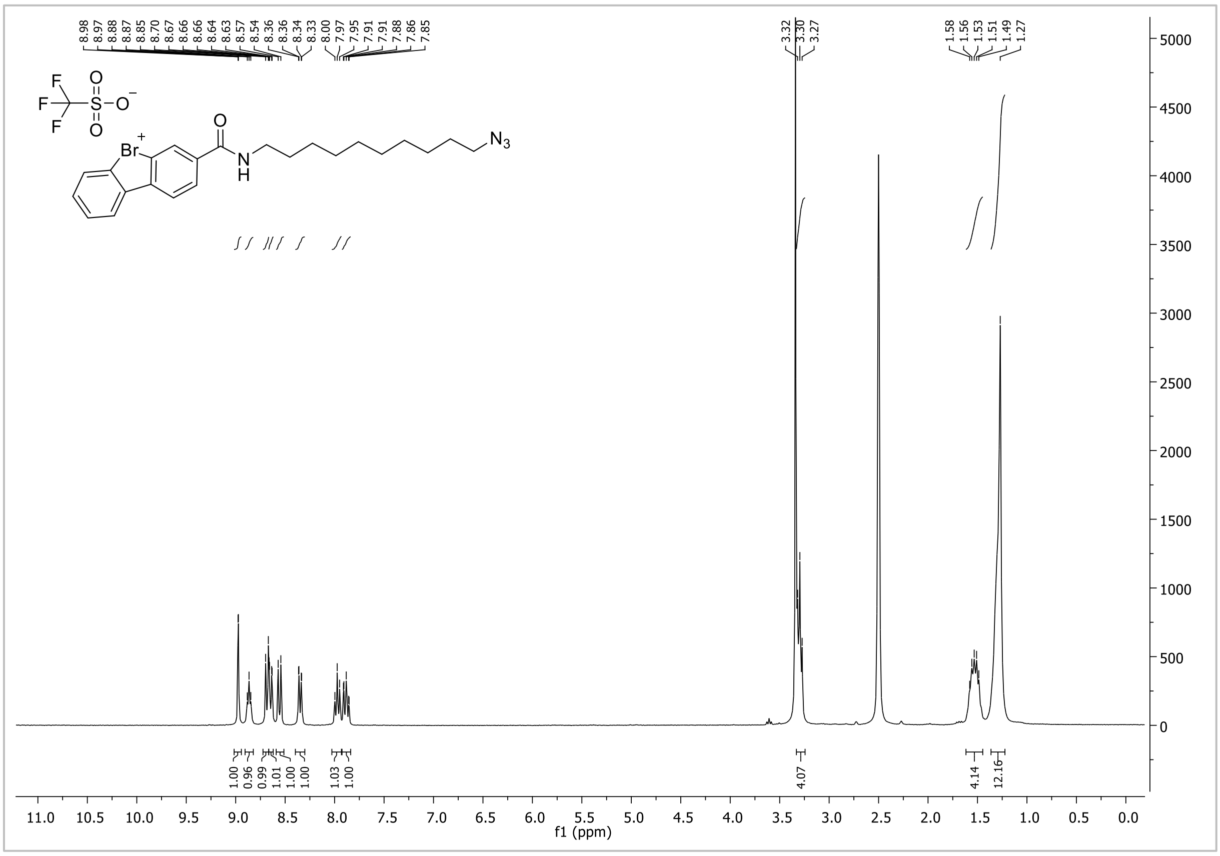

Figure S188. ^{1}H-NMR (300.13 MHz, DMSO-d_6) of compound **3ga**.

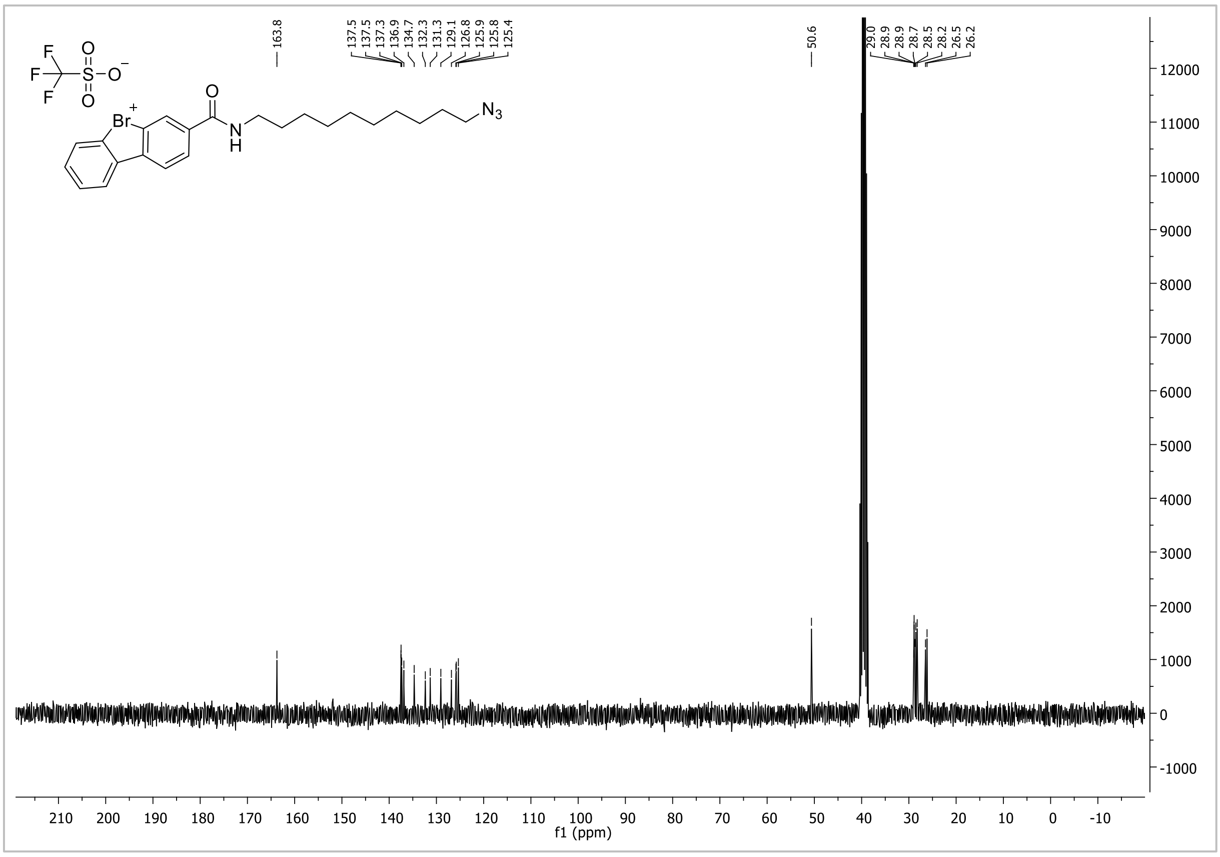

Figure S189. ^{13}C-NMR (75.47 MHz, DMSO-d_6) of compound **3ga**.

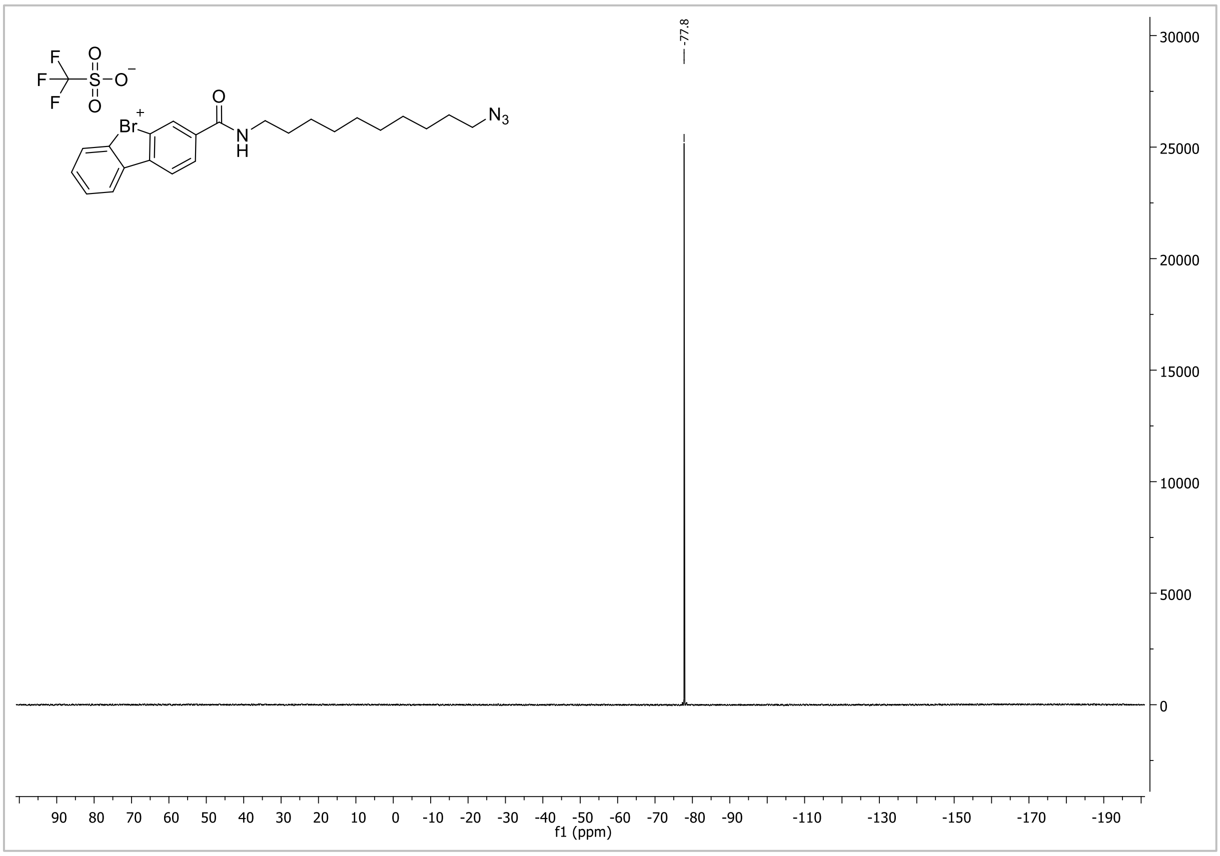

Figure S190. ^{19}F-NMR (282.40 MHz, DMSO-d_6) of compound **3ga**.

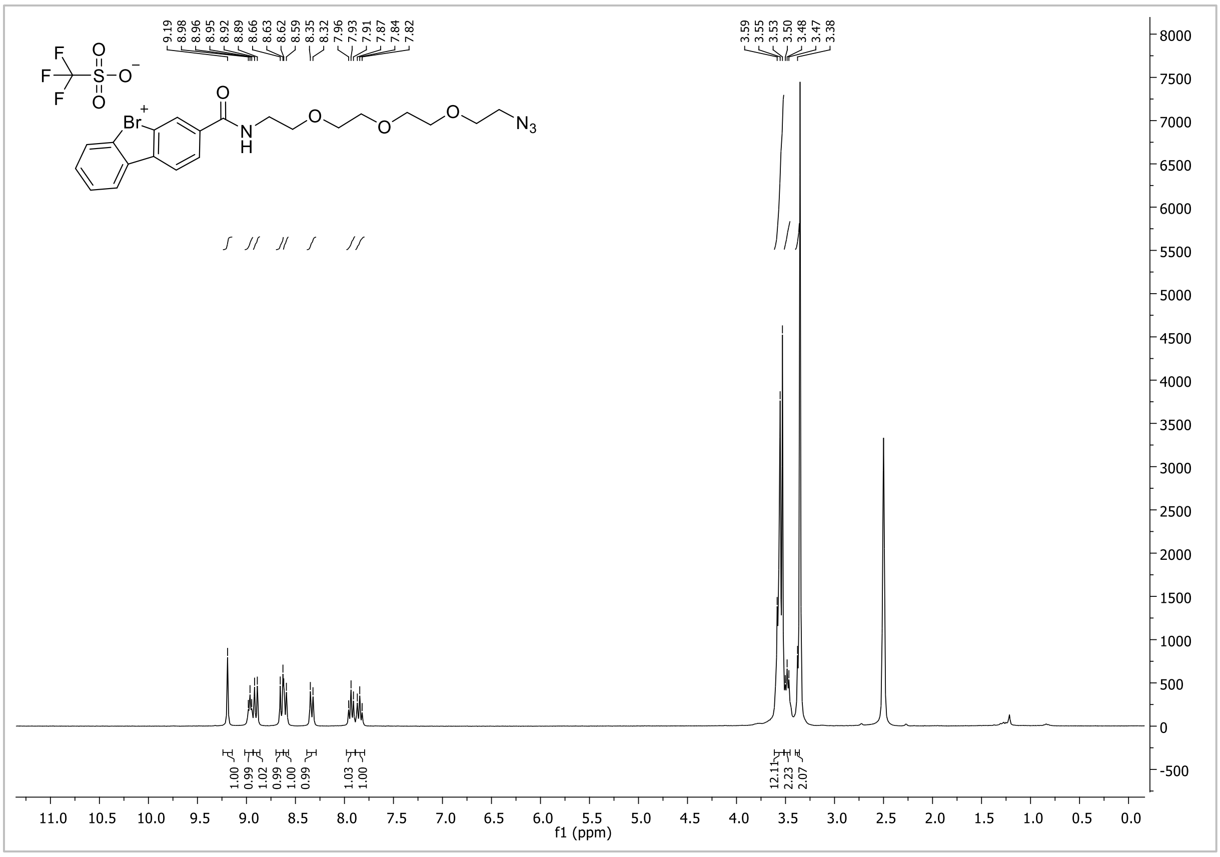

Figure S191. ^{1}H-NMR (300.13 MHz, DMSO-d_6) of compound **3gb**.

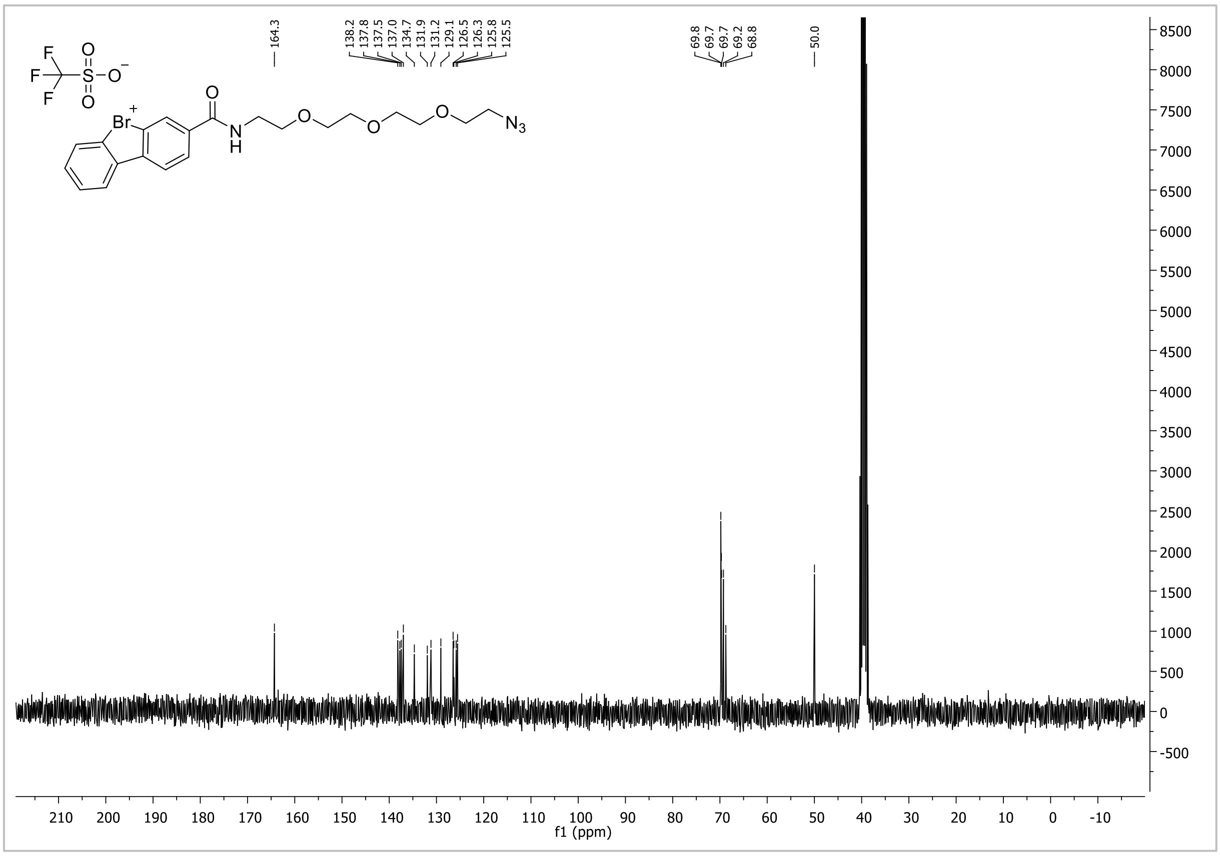

Figure S192. ^{13}C-NMR (75.47 MHz, DMSO-d_6) of compound **3gb**.

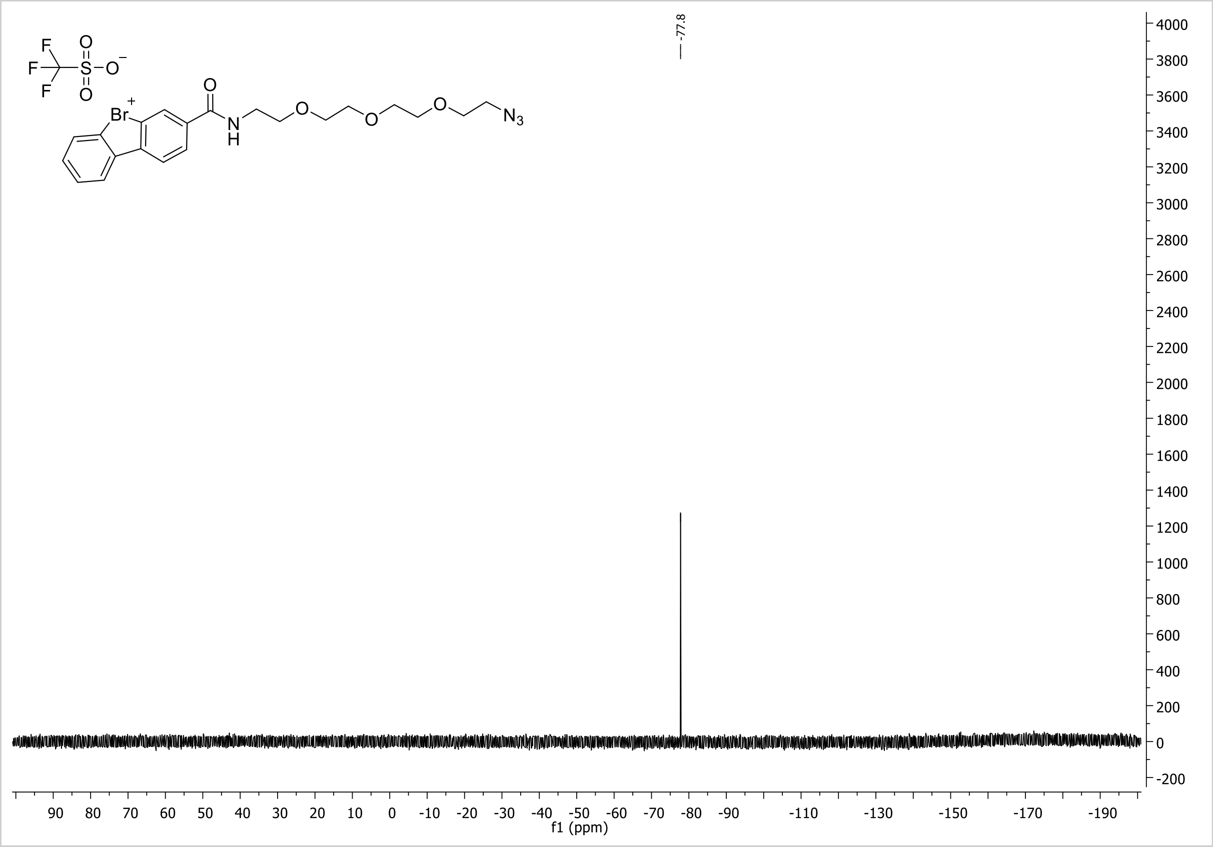

Figure S193. ^{19}F-NMR (282.40 MHz, DMSO-d_6) of compound **3gb**.

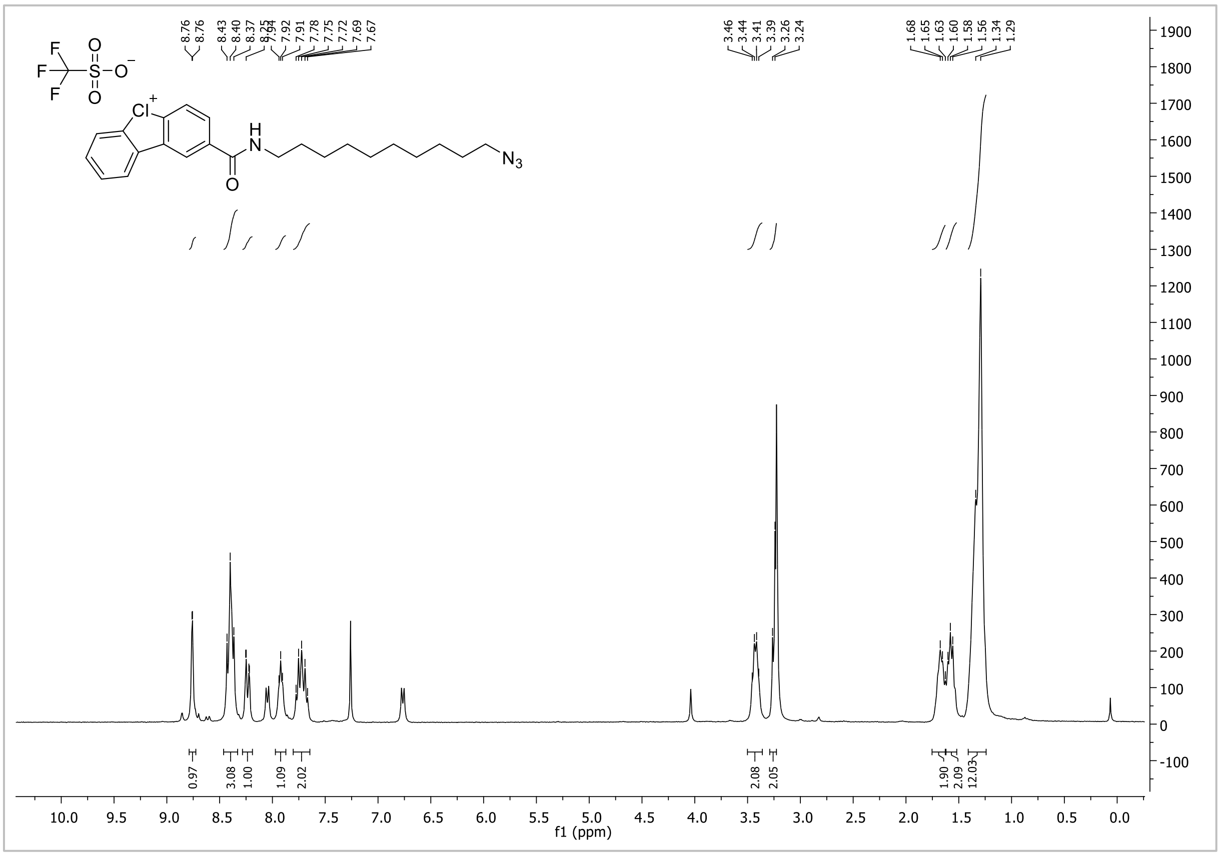

Figure S194. ^{1}H-NMR (300.36 MHz, DMSO-d_6) of compound **3ha**.

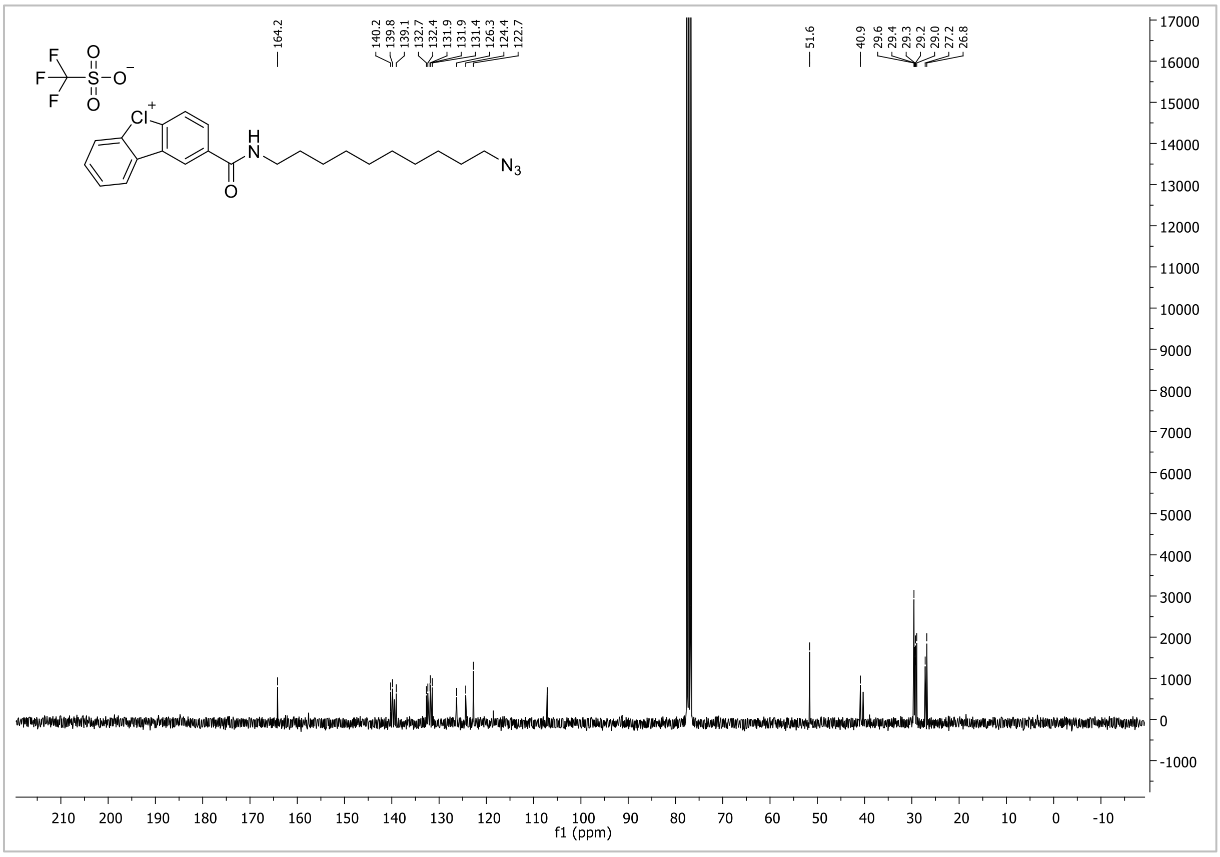

Figure S195. ^{13}C-NMR (75.53 MHz, DMSO-d_6) of compound **3ha**.

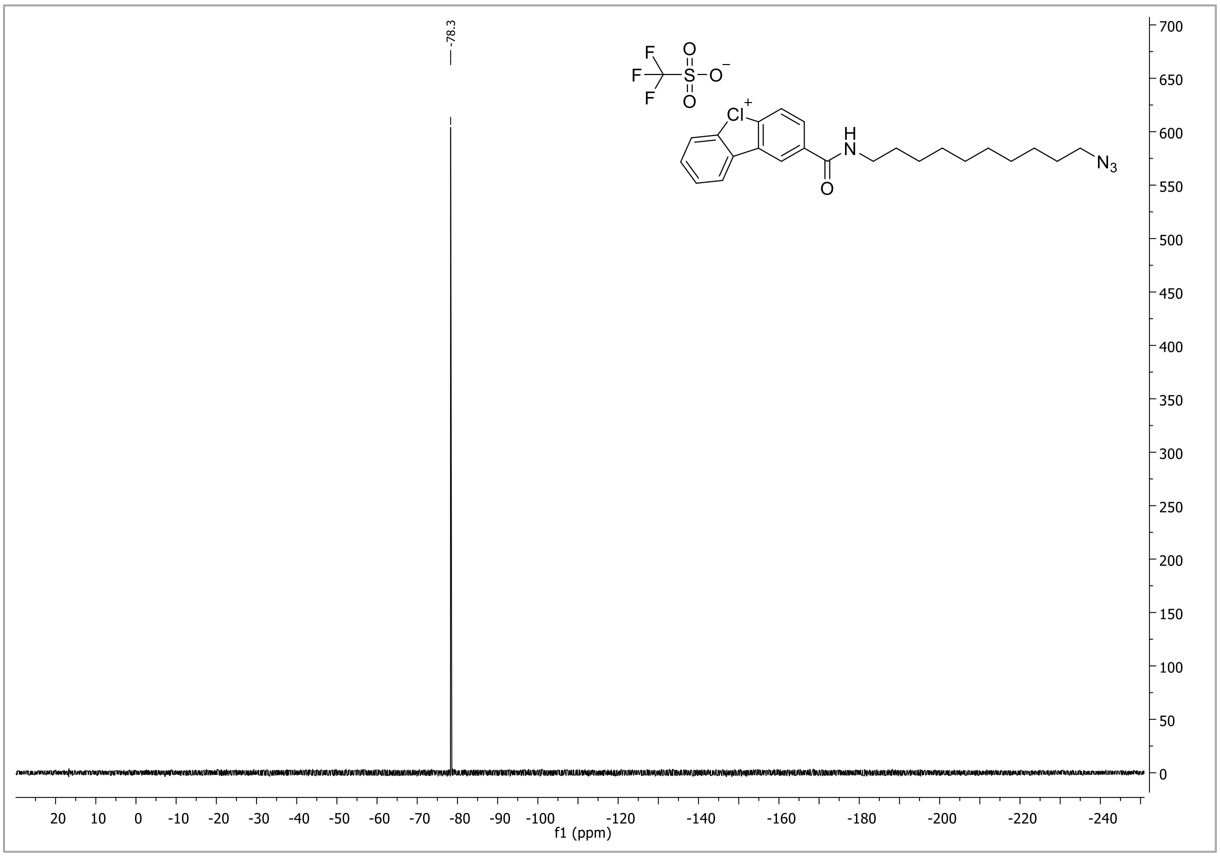

Figure S196. ^{19}F-NMR (470.53 MHz, DMSO-d_6) of compound **3ha**.

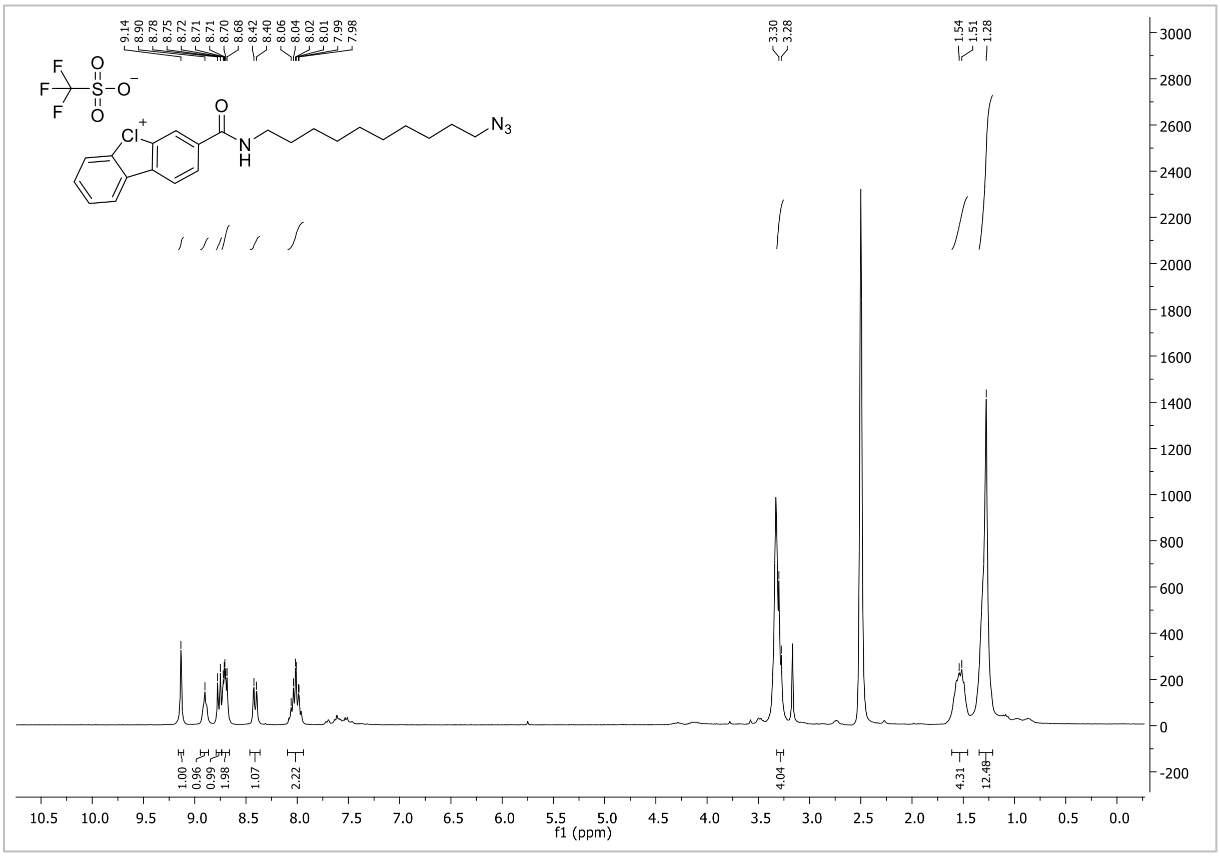

Figure S197. ^{1}H-NMR (300.36 MHz, DMSO-d_6) of compound **3ia**.

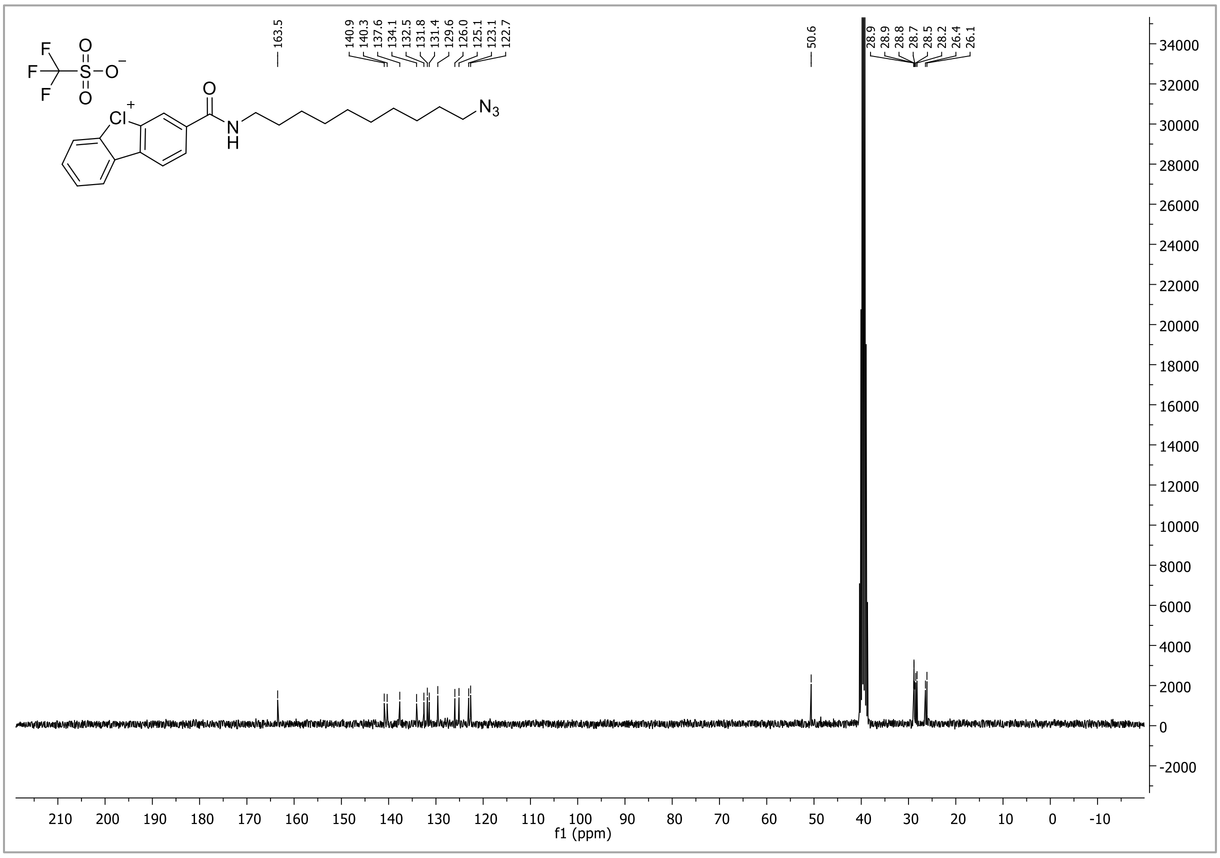

Figure S198. ^{13}C-NMR (75.53 MHz, DMSO-d_6) of compound **3ia**.

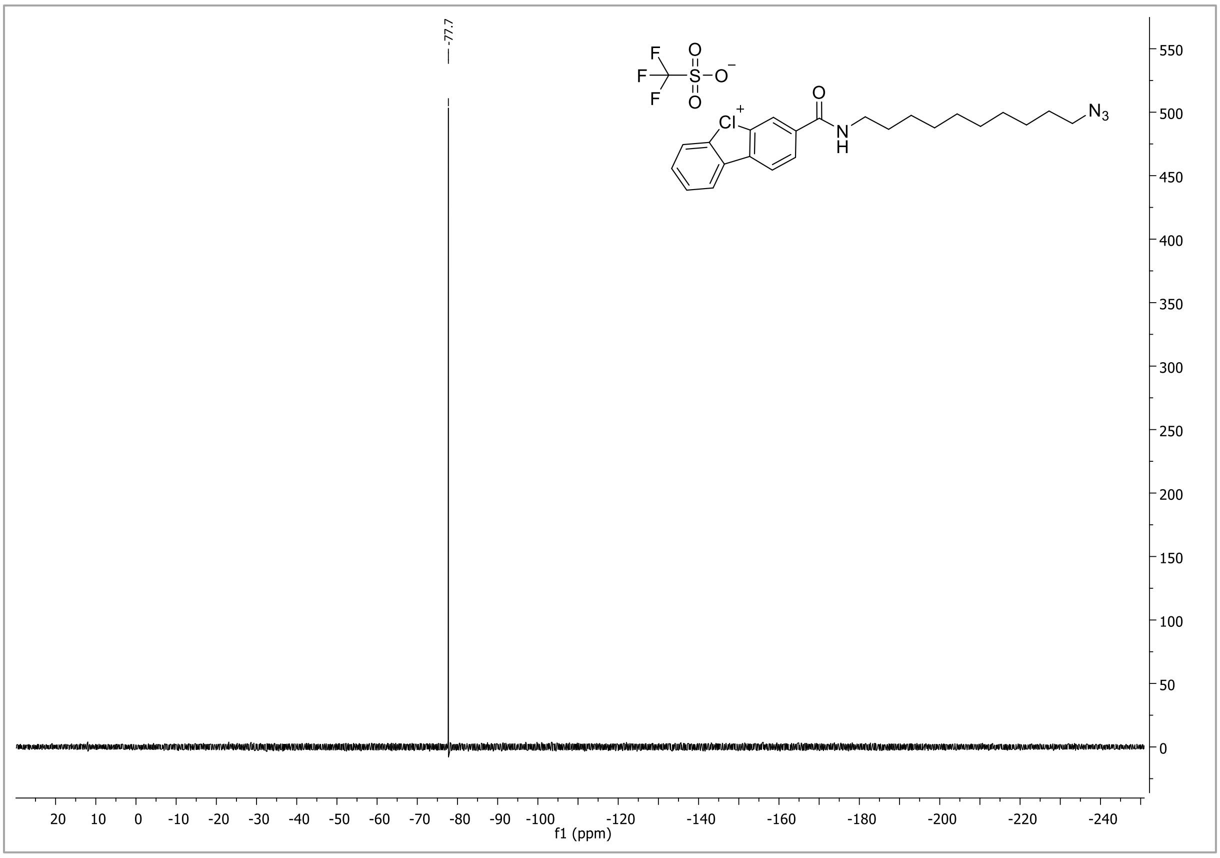

Figure S199. ^{19}F-NMR (470.53 MHz, DMSO-d_6) of compound **3ia**.

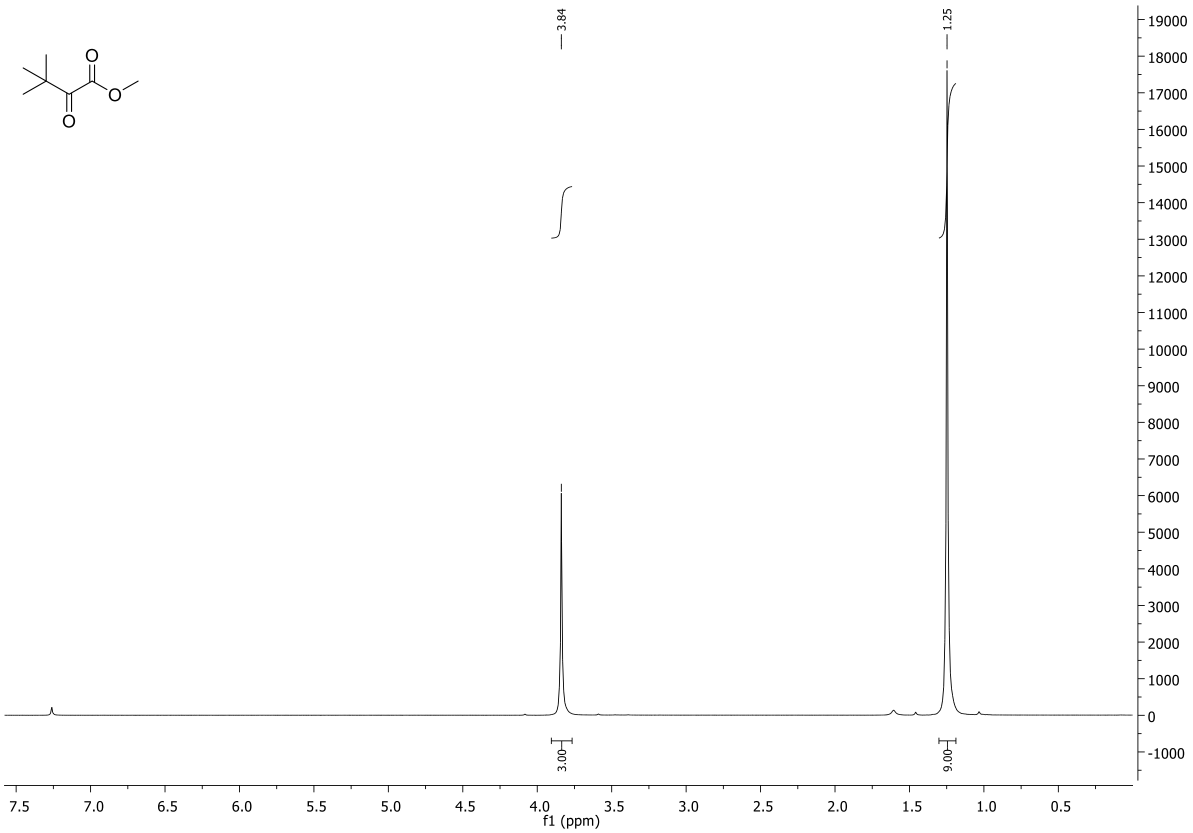

Figure S200. ^{1}H-NMR (300.36 MHz, $CDCl_3$) of compound **29**.

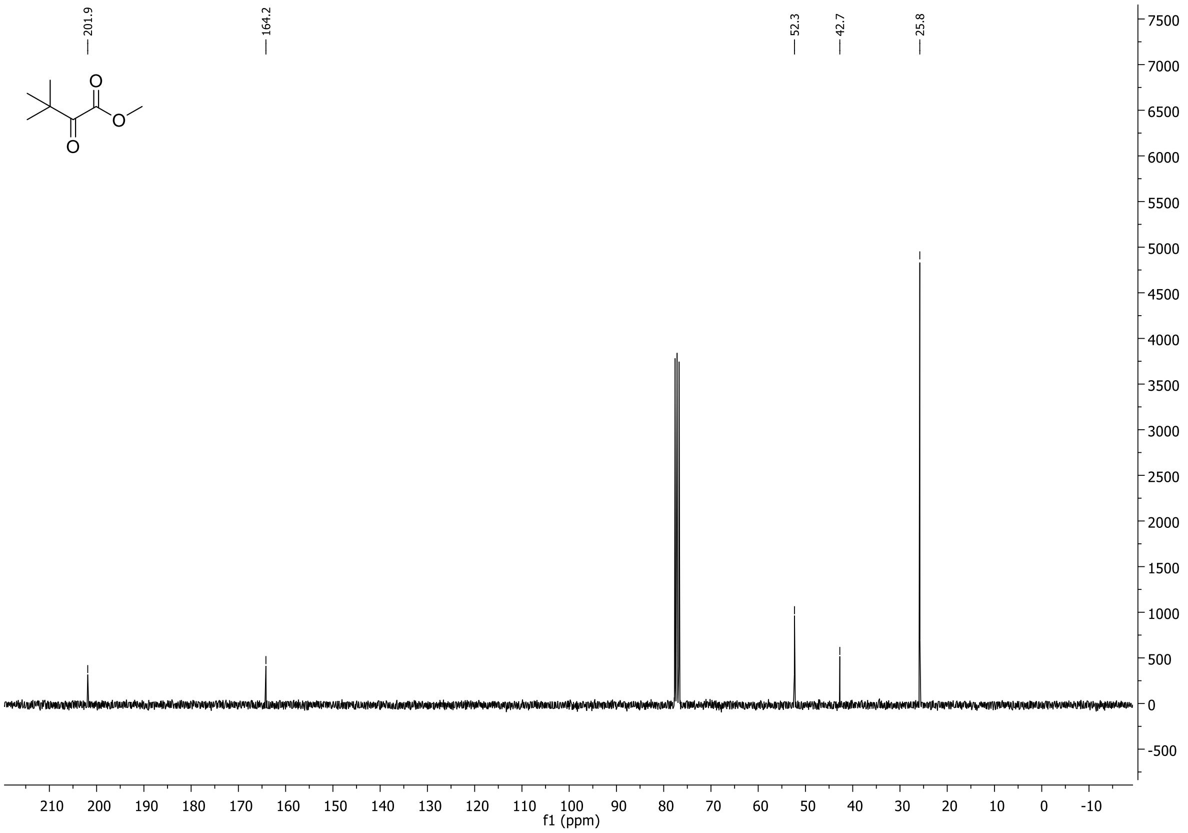

Figure S201. ^{13}C-NMR (75.53 MHz, $CDCl_3$) of compound **29**.

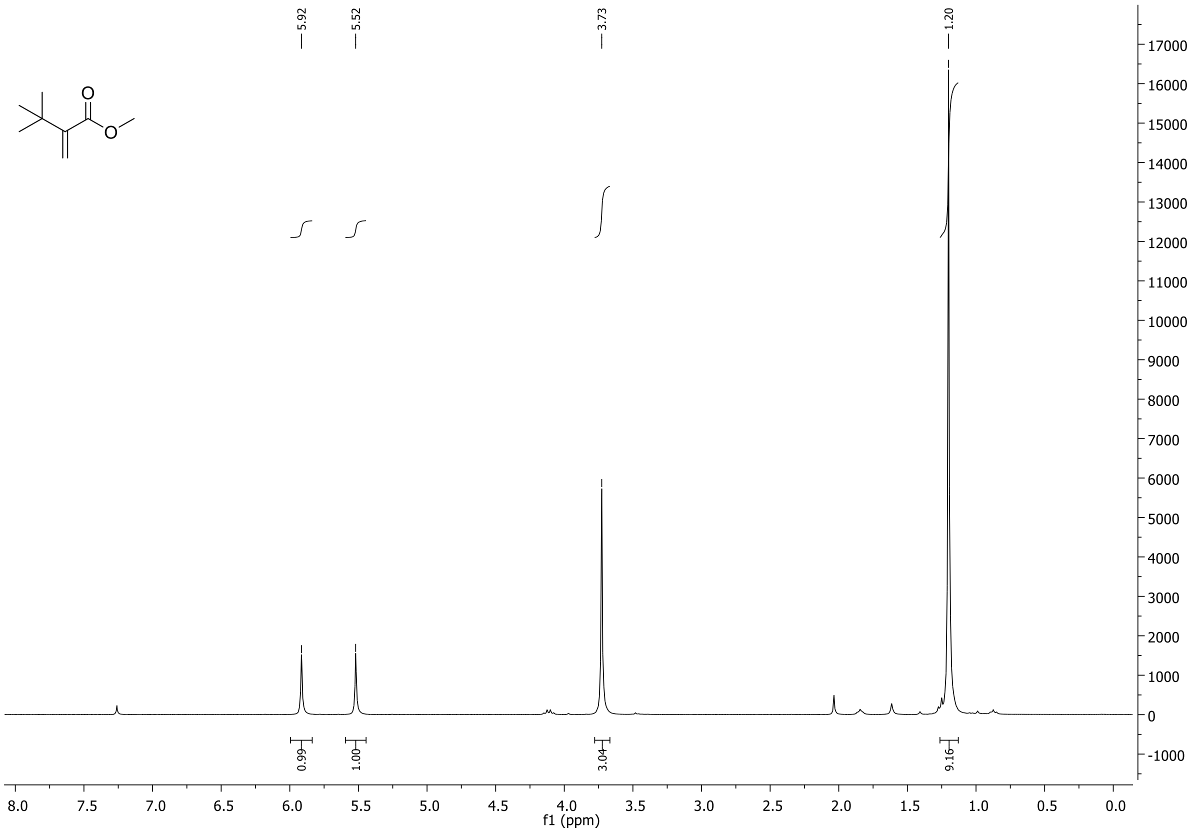

Figure S202. ^{1}H-NMR (300.36 MHz, $CDCl_3$) of compound **30**.

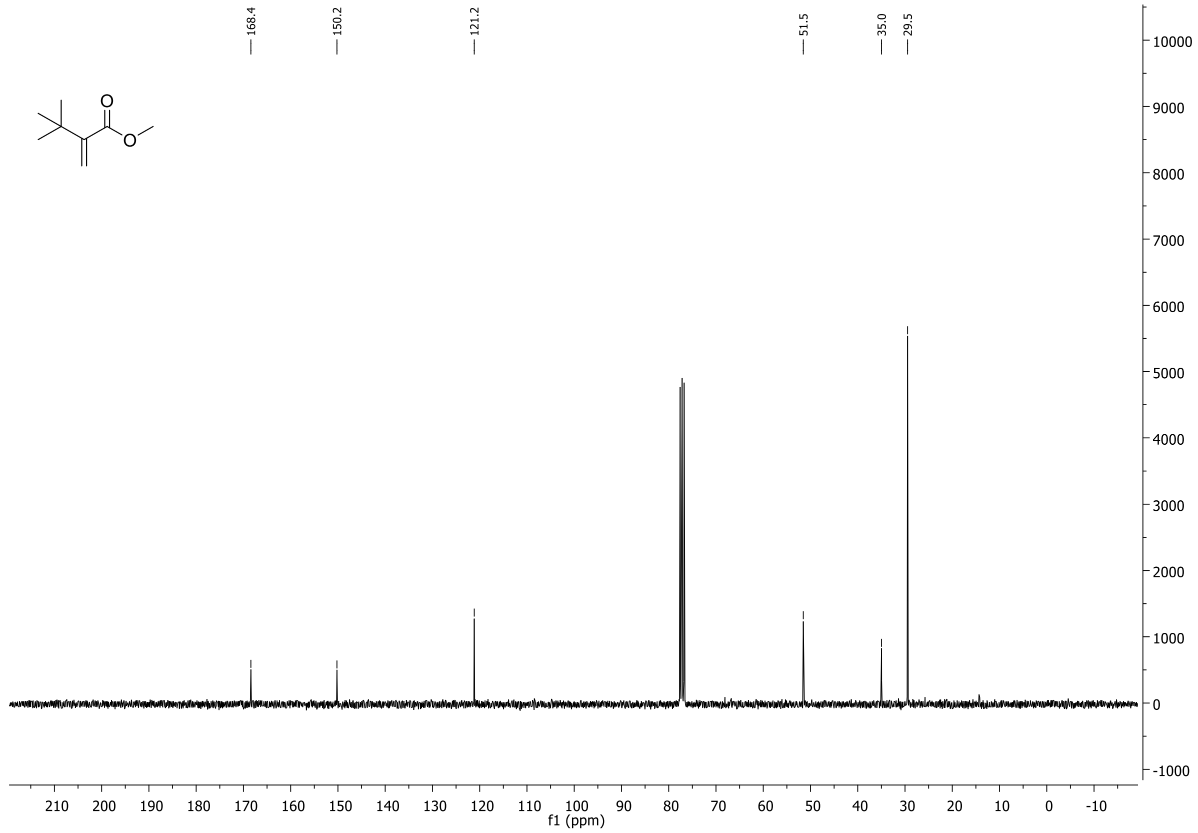

Figure S203. ^{13}C-NMR (75.53 MHz, $CDCl_3$) of compound **30**.
